# Ambient Hydrocarbonylation of Olefins Enabled by Visible‐Light

**DOI:** 10.1002/advs.74323

**Published:** 2026-02-10

**Authors:** Hongchi Liu, Tianze Zhang, Hanmin Huang

**Affiliations:** ^1^ State Key Laboratory of Precision and Intelligent Chemistry, Department of Chemistry University of Science and Technology of China Hefei P. R. China; ^2^ Key Laboratory of Green and Precise Synthetic Chemistry and Applications, Ministry of Education Huaibei Normal University Huaibei P. R. China

**Keywords:** ambient hydrocarbonylation, light irradiation, olefins, palladium catalysis

## Abstract

Hydrocarbonylations of olefins are among the most powerful and widely used processes in the fine and bulk chemical industries, allowing convergent assembly of valuable carbonylated molecules from simple feedstocks. However, an inherent contradictive requirement between olefin insertion and CO insertion causes these reactions to be costly and risky to handle harsh conditions (high temperature and pressurized CO). Here, we establish a photocatalytic approach to resolve the conflicts between the competitive olefin and CO insertion, enabling a palladium‐catalyzed ambient hydrocarbonylation of olefins under irradiation of visible‐light. This versatile, mild and robust platform offers efficient pathways to assemble valuable carboxylic acids, esters, amides, thioesters, and aldehydes under ambient conditions (at room temperature and 1 atm of CO) with excellent regioselectivities through hydroformylation and hydroxycarbonylation, hydroesterification, hydroaminocarbonylation, and hydrothiocarbonylation of simple olefins. Mechanistic studies suggest that concurrent facile olefin insertion and CO insertion via excitation of carbonyl palladium iodide complex is responsible for this activity.

## Graphical abstract

A novel photo‐induced methodology for hydrocarbonylation has been established, resolving the inherent conflicts between competitive olefin and CO insertion in traditional methods that necessitate harsh conditions. Accordingly, an efficient and versatile hydrocarbonylation of olefins enabled by visible‐light irradiation under ambient conditions has been developed, providing a general and robust approach to access valuable carboxylic acids, esters, amides, thioesters, and aldehydes with excellent regioselectivities.

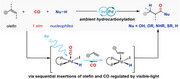

## 1 Introduction

**SCHEME 1:**
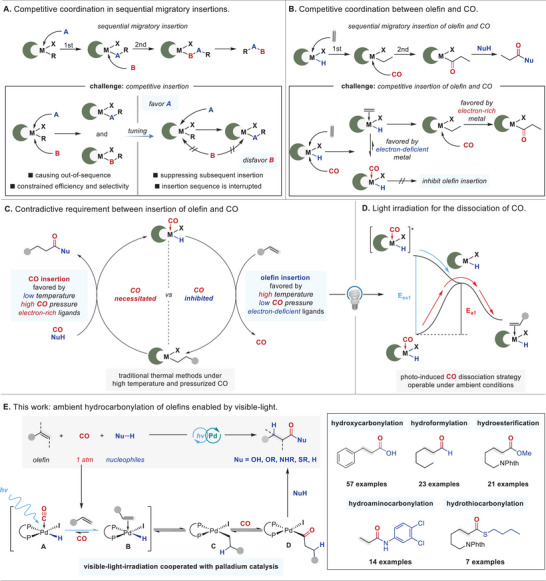
Background and concept.

Transition metal‐mediated migratory insertion represents one of the most pivotal elementary reactions in homogeneous catalysis and plays a fundamental role in numerous transformations with alkene or/and CO as coupling partners [1, 2, 3, 4]. It is believed that the coordination of unsaturated species to metal is a prerequisite for migratory insertion. For catalytic reactions relying on sequential migratory insertion of two different unsaturated species, competitive coordination to the metal center can lead to out‐of‐sequence outcomes, thereby posing significant challenges on the efficiency and selectivity (Scheme 1A) [5, 6]. In principle, tuning the steric and electronic properties of a metal center and its ligands could alter the binding affinity of a metal catalyst, allowing selective coordination and preferential insertion of one unsaturated species over another [7, 8, 9, 10]. However, factors favoring coordination and insertion for one unsaturated species often entail suppressing the other. As a result, force reaction conditions, such as high temperature and pressure, are generally required to balance the contradictive demands, which in turn diminishes the practicality and limits the substrate scope of reactions.

Olefin hydrocarbonylation offers one of the most efficient pathways to assemble valuable carbonylated molecules, including carboxylic acids, esters, amides, and aldehydes, from abundant feedstocks [11, 12, 13, 14, 15, 16, 17, 18, 19, 20]. Mechanistic studies have shown that the sequential insertion of alkene and CO into the in situ generated metal‐hydride species is necessary, with olefin insertion preceding CO insertion as a prerequisite for obtaining the desired reaction outcome [21, 22, 23]. Unfortunately, due to the distinct coordination affinity of CO and olefins, the steric or electronic features of the metal catalyst that favor insertion of olefin into the metal‐hydride complex often inhibit the subsequent CO insertion into the alkylmetal species (Scheme 1B). Moreover, due to CO's high ligation affinity, olefins typically become the underdog when competing with CO for vacant coordination sites under carbonylation conditions, making their insertion more challenging [24]. Two intuitive strategies could help address the challenge: (1) elevating temperature to promote CO dissociation, or (2) employing electron‐deficient ligands to modulate the electronic properties of the metal center and facilitate olefin binding/insertion. However, high temperatures also accelerate the reverse de‐carbonylation of the crucial acylmetal intermediate (formed via CO insertion) [25], necessitating high CO pressure to drive the reaction forward—which in turn suppresses olefin insertion. On the other hand, electron‐deficient ligands hinder CO insertion and also favor decarbonylation process even at relatively lower temperatures, restricting nucleophile selection to highly nucleophilic species (e.g., thiols) to drive the reaction forward by quickly capturing the in situ formed acylpalladium species [26, 27]. In essence, forcing successive olefin/CO insertion through reaction parameter manipulation creates a self‐amplifying temperature‐pressure spiral, until this dilemma reaches a compromised equilibrium under hazardous conditions, reinforcing the ‘stereotypical’ impression of harsh reaction conditions (Scheme 1C), costly pressure‐resistant equipment, and hazardous operational environments [28]. Moreover, the distinct competitive coordination behaviors between CO and olefin across diverse carbonylation reactions necessitates tailored metal/ligand combinations, resulting in a multitude of non‐universal catalyst systems. Therefore, the development of a general, efficient and selective method for olefin hydrocarbonylation proceeded under ambient conditions remains a longstanding and unmet challenge.

As an alternative to thermal activation, light irradiation has proven a valuable method for promoting CO dissociation from carbonyl metal complexes by accessing excited states often with greater efficiency than conventional thermal methods (Scheme 1D) [29, 30, 31, 32, 33]. Such approach not only can provide vacant coordination sites, but also can enhance the Lewis basicity of the metal center. For example, Alexanian and coworker have adopted this strategy to dissociate CO from Co_2_(CO)_8_ to form more basic [Co(CO)_3_]– species for the generation of H[Co(CO)_3_] in the absence of strong acids, which enabled the unmodified Co_2_(CO)_8_ to be utilized as a promising catalyst for promoting the olefin hydrocarbonylations under relatively mild conditions [34, 35]. However, non‐ambient conditions (60°C–90°C, 2–5 atm of CO) and high energy violet light were still required to ensure reactivity and selectivity. Palladium‐catalyzed hydrocarbonylation has been extensively studied and various ligands have been explored to modify the catalyst [11, 12, 13, 14, 15, 16, 17, 18, 19, 20, 21, 22, 23, 24, 25, 26, 27], however, no general protocol that can promote the above reactions under ambient condition was reported. In this context, we envisioned that visible‐light‐irradiation might be also cooperated well with palladium catalysis to overcome the competitive coordination between CO and olefin [36, 37, 38, 39, 40, 41, 42, 43, 44, 45]. As shown in Scheme 1E, the photo‐induced CO dissociation will drive the ligand exchange between the CO and olefin to generate olefin‐coordinated metal hydride species **C**, thereby ensuring the preferential insertion of olefin to form alkylpalladium species **D**. As such, no special ligands that facilitate both the olefin‐ and CO insertion will be required for promoting the entire catalytic cycle. Herein, we report a visible‐light irradiated, palladium‐catalyzed hydrocarbonylation of olefins at room temperature under 1 atm of CO or syngas (CO/H_2_) (Scheme 1E). This mild and general catalytic platform exhibits unprecedented and broad applicability across hydroformylation, hydrocarboxylation, hydroesterification, hydroaminocarbonylation and hydrothiocarbonylation, accommodating diverse olefins and nucleophiles to deliver valuable aldehydes, carboxylic acids, esters, amides and thioesters with excellent regioselectivities.

## 2 Results and Discussion

### 2.1 Reaction Development

**FIGURE 1:**
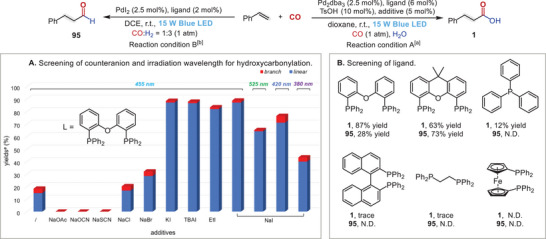
Optimization and Control Studies. (A) Reaction conditions A: olefin (0.4 mmol), CO (1 atm), H_2_O (20 equiv.), Pd_2_dba_3_ (2.5 mol%), ligand (6 mol%), TsOH (10 mol%), additive (5 mol%) in dioxane (2.0 mL) at r.t., irradiated for 24 h. (B) Conditions B: olefins (0.2 mmol), CO:H_2_ = 1:3 (1 atm), PdI_2_ (2.5 mol%), ligand (3 mol%) in DCE (1.0 mL) at r.t., irradiated by 15 W blue LEDs for 24 h. Yields were determined by GC using tetradecane as the internal standard.

To validate our hypothesis, we first focused on evaluating the feasibility of this visible light‐irradiated, palladium‐catalyzed protocol with styrene as a model substrate and water as the coupling partner in the presence of Pd_2_dba_3_ and DPEphos (bis[2‐(diphenylphosphino)phenyl] ether) [42, 43, 44, 45]. Extensive experimentation using this standard substrate (for details, see Figures S1 and S2 and Tables S1–S11) revealed that modulation of the photochemical properties by addition of a suitable coordinating anion was required to achieve high conversion with NaI stood out as the ideal iodide anion source (Figure 1A) [46]. Additionally, replacing blue light with violet or green light resulted in diminished yield and selectivity. Further ligand screening confirmed that DPEphos exhibited optimal performance. Moreover, no product formation was observed in the absence of either TsOH or blue light. The optimized conditions also involved conducting the reaction at room temperature in dioxane, furnishing the phenylpropionic acid 1 in 87% isolated yield (Figure 1B). On the other hand, the ambient hydroformylation of styrene was also established under 1 atm of syngas CO/H_2_ (1:3) by using PdI_2_/Xantphos as catalyst in 1,2‐dichloroethane (DCE), affording 3‐phenylpropanal in 73% yield with >20:1 regioselectivity.

### 2.2 Substrate Scope

With the optimized conditions in hand, we sought to examine the substrate scope of this transformation. Firstly, the adaptability of this strategy to olefins for hydroxycarbonylation was evaluated (Table 1). Aromatic olefins, including styrene and its derivatives as well as 2‐vinylnaphthalene, could afford the desired products (**1**–**12**) in 65%–87% yields with excellent regioselectivities. Simple aliphatic alkenes, such as 1‐octene, vinylcyclohexane, 3,3‐dimethyl‐1‐ butene and allylbenzene could be smoothly converted to the desired products (**13**–**16**) in 79%–85% yields and >20:1 regioselectivities by using more electron‐deficient **L2** as a ligand. Different transformable handles including halides (**17** and **18**), hydroxy (**19**), sulfonyloxy (**20**), acyloxy (**21** and **22**), phenoxy (**23**), ketone (**24** and **25**), carboxylic acid (**26**) and cyano (**27**) were well tolerated at different positions of the aliphatic chains. Moreover, substrates with aliphatic chains bearing imide (**28**), sulfonamide (**29**), various heterocycles (**30**–**32**), sulfonyl (**33**), and phosphonyl (**34**) also achieved good yields. A high degree of chemoselectivity was observed in this transformation, as evidenced by well‐tolerated substrates containing multiple unsaturated bonds (**35**–**37**). In addition, electron‐deficient alkenes such as (perfluorobutyl)ethylene (**38**) and acrylamide (**39**) also performed well under this system, especially using more electron‐rich **L3**. Disubstituted alkenes were also applicable for this transformation. For example, exocyclic alkenes produced the desired products (**40** and **41**) in 93% and 89% yields, while reactivities of other 1,1‐disubstituted alkenes (**42**–**44**) were slightly lower. For 1,2‐disubstituted alkenes, cyclic alkenes afforded the desired products (**45**–**47**) smoothly, while acyclic internal alkenes could undergo chain walking to give the linear products (**48**, **49**) with 5:1 and 7:1 regioselectivities. To validate the applicability of our protocol toward olefins prevalent in natural products and pharmaceuticals, a variety of intricate olefins were investigated and proved compatible, establishing a practical strategy for carbonylation of such bioactive molecules (**50**–**57**) under mild reaction conditions.

**TABLE 1:** Substrate scope of hydroxycarbonylation.^a)^

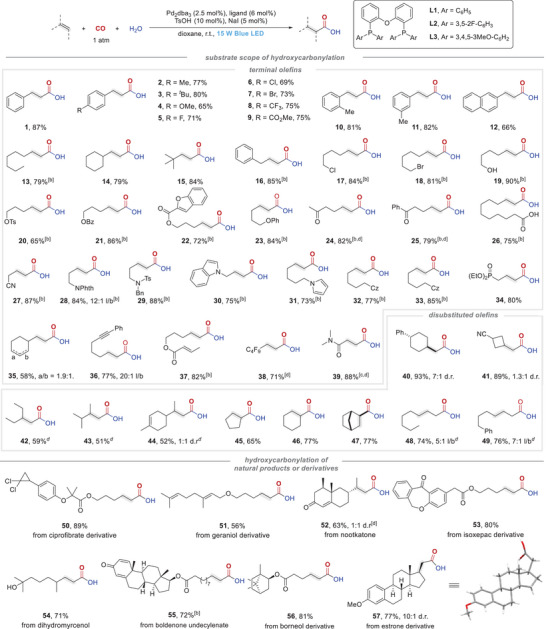

^a)^ Reaction conditions: olefin (0.8 mmol), CO (1 atm), H_2_O (20 equiv.), Pd_2_dba_3_ (2.5 mol%), DPEphos (6 mol%), TsOH (10 mol%), NaI (5 mol%) in dioxane (2.0 mL) at r.t., irradiated by 15 W blue LEDs for 24 h.

^b)^ With ligand **L2**.

^c)^ With ligand **L3**.

^d)^ Irradiated by 15 W blue LEDs for 48 h. Unless otherwise noted, the ratio of l/b > 20:1. Isolated yield.

Next, we examined the adaptability of this strategy to other types of hydrocarbonylation reactions by using 2‐(pent‐4‐enyl)‐isoindoline‐1,3‐dione as the olefin coupling partner (Table 2). First, for the hydroesterification reaction, a series of aliphatic alcohols, spanning primary, secondary, and tertiary alcohols, could be well tolerated in this transformation, providing desired linear esters (**58**–**63**) in 70%–84% yields with excellent regioselectivities using more electron‐deficient **L2** as ligand. Functionalized alcohols, such as ethylene glycol and trifluoroethanol, also exhibited good reactivities to provide desired products (**64** and **65**) in good yields. Due to the relatively weak nucleophilicity, relative lower yields (56%–67%) were obtained for the target phenyl esters (**66**–**68**) when phenols were utilized as coupling partner. *N*‐ Hydroxyimide could also serve as a coupling partner to afford the linear product (**69**) in 54% yield with excellent regioselectivity. Notably, hydroxy‐containing natural products and pharmaceutical agents were also compatible with this reaction to give the corresponding products (**70**–**76**) in 58%–67% yields. In addition, this strategy showed superior performance in hydroaminocarbonylation of olefins. Due to the higher nucleophilicity, both aniline and its derivatives—substituted either on the benzene ring or at the nitrogen atom—produced the target linear amides (**77**–**85**) in 81%–94% yields with excellent regioselectivities. In contrast, due to its strong basicity, aliphatic amine only furnished the desired amide (**86**) in moderate yield. When 4‐methylbenzenesulfonhydrazide was employed as the coupling partner, 87% yield of the desired product (**87**) was observed with >20:1 regioselectivity. It is worth highlighting that this reaction exhibited excellent performance in the late‐stage modification of drug‐containing amine moieties (**88**). Similarly, thiols and thiophenols possessing strong nucleophilicity underwent the desired hydrothiocarbonylation smoothly to afford the linear thioesters (**89**–**95**) in 76%–93% yields with excellent regioselectivities. Additionally, we examined the applicability of hydroformylation across diverse olefins. As expected, good reaction efficiencies as well as excellent regioselectivitie were obtained for styrenes (**96**–**100**). Moreover, a series of aliphatic alkenes reacted smoothly, delivering the desired aldehydes (**101**–**118**) in 60%–90% yields with >20:1 regioselectivities, where functional groups including phenoxy, ester, amide, heterocycle and ketone were well compatible (**101**–**109**). Notably, this protocol also accommodates 1,1‐disubstituted alkenes, delivering the desired products (**110**–**114**) with enhanced efficiency (72%–92% yields). Internal alkenes, including cyclic alkene and acyclic internal alkenes, could also undergo the desired hydroformylation to afford the target linear aldehydes (**115**–**117**) in good yields with good to excellent regioselectivities. The alkene derived from estrone also afforded the desired product (**118**) in 74% yield with good diastereoselectivity. However, the limitation is that the current catalytic protocol is only good for less hindered alkenes, while trisubstituted, tetrasubstituted alkenes and enol ethers were proven to be ineffective for the hydrocarbonylation reactions.

**TABLE 2:** Substrate scope of hydroxycarbonylation.

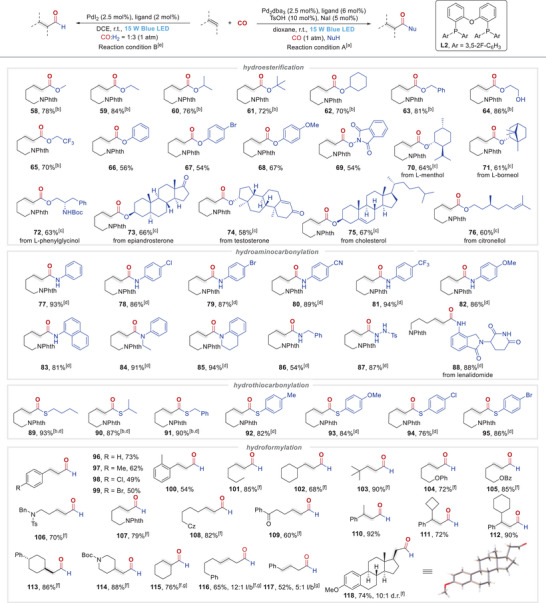

^a)^ Reaction conditions A: olefins (0.4 mmol), CO (1 atm), nucleophile (5.0 equiv.), Pd_2_dba_3_ (2.5 mol%), DPEphos (6 mol%), TsOH (10 mol%), NaI (5 mol%) in dioxane (2.0 mL) at r.t., irradiated by 15 W blue LEDs for 36 h.

^b)^ With ligand **L2**.

^c)^ With nucleophile (2.0 equiv.).

^d)^ With nucleophile (1.5 equiv.), irradiated for 24 h.

^e)^ Reaction conditions B: olefins (0.2 mmol), CO:H_2_ = 1:3 (1 atm), PdI_2_ (2.5 mol%), Xantphos (3 mol%) in DCE (1.0 mL) at r.t., irradiated by 15 W blue LEDs for 24 h.

^f)^ With ligand DPEphos (2 mol%), K_3_PO_4_ (2 mol%) at 10°C.

^g)^ Irradiated for 48 h. Unless otherwise noted, the ratio of l/b > 20:1. Isolated yield.

### 2.3 Synthetic Applications

**FIGURE 2:**
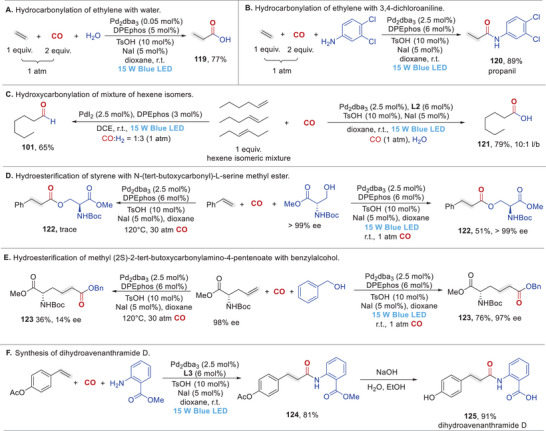
Synthetic applications.

In addition to the demonstrated excellent substrate generality and applicability, we further highlighted the synthetic utility of our strategy through the following transformations (Figure 2). As a cornerstone of the petrochemical industry, the hydrocarbonylation of ethylene represents one of the most valuable transformations. Using our method, a 1:2 ethylene‐CO gas mixture was used as the feedstock, achieving high efficiency to generate the corresponding products under ambient temperature and atmospheric pressure. The propionic acid **119** could be obtained in 77% yield based on ethylene in the presence of 0.1 mol% palladium catalyst (Figure 2A), demonstrating the exceptional efficiency of this hydrocarboxylation. Alternatively, using 3,4‐dichloroaniline as the nucleophile delivered propanil (**120**, a widely used herbicide) in 89% yield (Figure 2B). A mixture composed of equal amounts of all three hexene isomers could be also directly utilized as substrate to produce 1‐heptanoic acid **121** and 1‐heptaldehyde **101** in good yield and selectivity, demonstrating the highly regioconvergent nature of this hydrocarbonylative transformation (Figure 2C). In addition, hydroesterification of styrene with chiral Boc‐L‐serine methyl ester by using our newly developed protocol could afford the corresponding chiral ester **122** in 51% yield and >99% ee. However, almost no desired reaction occurred when the same reaction was conducted under traditional high‐temperature and high‐pressure conditions (Figure 2D). Similarly, Boc‐L‐allylglycine methyl ester also proved to be competent alkene‐substrate for this light‐driven reaction to produce the desired chiral ester **123** in 76% yield with the chiral center fully preserved, while thermal and pressurized conditions led to lower yield and partial racemization, highlighting the superiority of our mild protocol for bioactive substrates (Figure 2E). Finally, hydroaminocarbonylation of 4‐acetoxystyrene with methyl 2‐aminobenzoate under the light‐promoted conditions followed by straightforward hydrolysis, dihydroavenanthramide D (**125**, an antihistamine drug) was obtained in excellent yield, which further demonstrated its synthetic practicality (Figure 2F).

### 2.4 Mechanistic Studies

**FIGURE 3:**
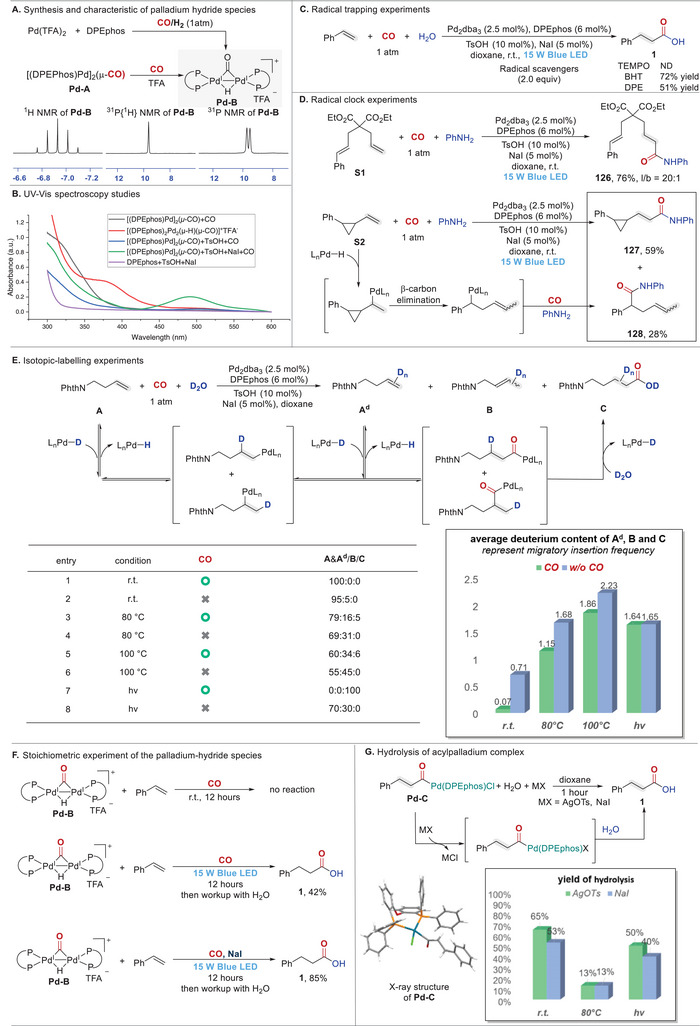
Mechanistic studies.

Mechanistic investigations were conducted to elucidate the role of light in promoting the olefin hydrocarbonylations (for details, see Figures S3–S12). First, the Pd(0) species ([(DPEPhos)Pd]_2_(*µ*‐CO)) (**Pd‐A**) and binuclear Pd(I)‐Pd(I) hydride species [(DPEphos)_2_Pd_2_(*µ*‐H)(*µ*‐CO)]^+^TFA^−^ (**Pd‐B**) were prepared and characterized according to the reported methods (Figure 3A) [20, 37]. We confirmed that **Pd‐B** species can be generated via two distinct pathways: reduction of Pd(II) species under a CO/H_2_ atmosphere, and oxidative addition of acids to Pd(0) species under a CO atmosphere, corresponding to reaction conditions of hydroformylation and hydrocarbonylation respectively. Subsequently, a series of UV‐vis spectra were obtained pertaining to **Pd‐A** and **Pd‐B**. As depicted in Figure 3B, the **Pd‐A** exhibited negligible blue light absorption. In contrast, upon treatment of **Pd‐A** with TsOH, a new absorption band around 500 nm was emerged, which was markedly intensified by subsequent introduction of NaI. In addition, the UV‐Vis spectrum of **Pd‐B** was also measured, showing its main absorption in the violet region with slight absorption in blue light region. These observations indicated that the palladium hydride was most likely to be a photoactivated species. The light absorption strengthening effect of NaI might be ascribed to the iodide‐coordination could promote the binuclear **Pd–B** species to be converted to the active mononuclear palladium species [20]. Furthermore, the enhanced visible‐light absorption could facilitate the photo‐promoted CO dissociation, which could further improve the efficiency of the desired hydrocarbonylation reactions. Given the prevalence of radical intermediates in photochemical carbonylation [36, 37, 38, 39, 40, 41], radical trapping studies were performed to rule out the involvement of radical species in our catalytic system. Introducing radical scavengers (2 equiv.) to the standard reaction failed to intercept alkyl radical intermediates, although the reactivities that led to the target product were somewhat diminished (Figure 3C). In addition, radical clock experiment with unconjugated diene (**S1**) produced the corresponding hydroaminocarbonylation product **126** following the standard reaction course, while no ring‐closing products were detected (Figure 3D). On the other hand, reaction of the three‐membered‐ring‐containing olefin (**S2**) predominantly yielded the linear carbonylation product **127** with only minor amounts of the ring‐opening product **128**, which might be resulted from the β‐carbon elimination of alkylpalladium species.

To further elucidate the inhibitory effect of CO on the migratory insertion of olefin into the palladium‐hydride species and clarify the mechanism of the light‐promotion, we conducted isotopic‐labeling experiments using D_2_O as nucleophile under different conditions (Figure 3E). In theory, the reversible equilibrium between migratory insertion and β‐hydrogen elimination resulted in partial deuteration of substrates and products, where average deuterium content directly correlated well with the migratory insertion efficiency [47]. As such, analysis of average deuterium content of the resulting mixtures under CO and CO‐free conditions could quantitatively assess CO inhibitory effect on migratory insertion of olefins. Under room temperature, an average deuterium content of 0.71 was observed in the resulting mixture without CO. On the contrary, olefin was completely recovered with deuterium nearly absent in the presence of CO, confirming that CO can entirely suppress olefin migratory insertion under mild conditions. At elevated temperatures (80°C/100°C), the average deuterium incorporation in resulting mixtures rose to 1.68/2.23 under CO‐free conditions, and 1.15/1.86 in the presence of CO, demonstrating that high temperature can partially overcome the suppressive effect of CO on olefin insertion. Remarkably, ‌under light irradiation, the deuterium content in the CO‐present system was nearly equal to that of the CO‐free system‌ (1.65 and 1.64, respectively). To provide more direct evidence for light‐induced remission of the inhibitory effect of CO on migratory olefin insertion, we conducted stoichiometric experiments using palladium‐hydride species **Pd–B** with styrene in the presence of CO (Figure 3F). No reaction occurred under dark conditions, leaving both **Pd–B** and styrene entirely retained. In contrast, light irradiation led to the formation of the desired carbonylated product. Upon addition of NaI, the reactivity was significantly enhanced to give the desired product in 85% yield, further exhibiting the promoting role of iodide anions in the photochemical process. These results convergently demonstrated the ‌superior efficacy of photo‐induced CO dissociation ‌in mitigating the suppression of olefin insertion. In addition, to elucidate whether light irradiation can affect the hydrolysis of acylpalladium species, the DPEphos‐ligated acylpalladium complex **Pd‐C** was synthesized and characterized by X‐ray single crystal diffraction analysis [48]. Direct hydrolysis of **Pd‐C** proved to be inefficient, which might be attributed to strong coordination of chloride (Figure S11). Therefore, subsequent hydrolysis studies with Cl^−^ replaced by TsO^−^ or I^−^ (postulated counter ions in our system) were conducted at 25°C, 80°C, and under light irradiation (Figure 3G). Product **1** could be obtained in good yields at room temperature under both dark and light‐irradiated conditions, indicating the light‐insensitive nature of this step. In contrast, elevated temperatures led to significant yield reduction due to the decomposition of the acylpalladium species [25], thereby accentuating the superiority of photo‐activation. Collectively, these findings delineated that light irradiation could bypass harsh thermal and pressure requirements by selectively alleviating the inhibitory effect of CO on the migratory insertion of olefins, thereby ensuring sorting of olefin insertion and CO insertion as the desired sequence.

## 3 Conclusion

In summary, we have established a photocatalytic solution to olefin hydrocarbonylation chemistry, one of the most important classes of transformations in the chemical industry. Here we have focused on the development of a visible‐light mediated paradigm for precisely manipulating elementary reactions, establishing a versatile, mild and robust catalytic platform for olefin hydrocarbonylation at room temperature under 1 atm CO or syngas. A variety of valuable carboxylic acids, esters, amides, thioesters, and aldehydes were produced with excellent regioselectivities from simple alkenes and CO via these newly developed olefin hydrocarbonylation reactions by using a commercially available simple palladium catalyst, underscoring its broad applicability and revolutionary potential for industrialization. The key to achieving this efficient carbonylation under ambient conditions lies in the identification that the complex competitive coordination between CO and olefin can be tamed by light irradiation of the carbonyl palladium iodide intermediate. By leveraging photo‐induced CO dissociation, efficient sorting of olefin and CO insertion as desired sequence was successfully realized. From a broad perspective, the present study should stimulate our ideas for the further development of efficient transition‐metal catalysis by directly manipulating elementary reactions.

## Funding

National Key Research and Development Program of China (2023YFA1507500), and National Natural Science Foundation of China (22525012 and 22350008).

## Conflicts of Interest

The authors declare no conflicts of interest.

## Supporting information




**Supporting File**: advs74323‐sup‐0001‐SuppMat.pdf.

## Data Availability

The data that support the findings of this study are available in the supplementary material of this article.

## References

[1] J. F. Hartwig , Organotransition Metal Chemistry: From Bonding to Catalysis (University Science Books, 2010).

[2] I. P. Beletskaya and A. V. Cheprakov , “The Heck Reaction as a Sharpening Stone of Palladium Catalysis,” Chemical Reviews 100 (2000): 3009–3066, 10.1021/cr9903048. 11749313

[3] H. Mu , L. Pan , D. Song , and Y. Li , “Neutral Nickel Catalysts for Olefin Homo‐ and Copolymerization: Relationships Between Catalyst Structures and Catalytic Properties,” Chemical Reviews 115 (2015): 12091–12137, 10.1021/cr500370f. 26528708

[4] J. Peng , H. Geng , and X. Wu , “The Chemistry of CO: Carbonylation,” Chemistry (Weinheim An Der Bergstrasse, Germany) 5 (2019): 526–552, 10.1016/j.chempr.2018.11.006.

[5] C. Chen , “Designing Catalysts For Olefin Polymerization And Copolymerization: Beyond Electronic and Steric Tuning,” Nature Reviews Chemistry 2 (2018): 6–14, 10.1038/s41570-018-0003-0.

[6] C. Tan and C. Chen , “Emerging Palladium and Nickel Catalysts for Copolymerization of Olefins With Polar Monomers,” Angewandte Chemie International Edition 58 (2019): 7192–7200, 10.1002/anie.201814634. 30719812

[7] F. Aubke and C. Wang , “Carbon Monoxide as a σ‐Donor Ligand in Coordination Chemistry,” Coordination Chemistry Reviews 137 (1994): 483–524, 10.1016/0010-8545(94)03010-N.

[8] G. Frenking and N. Fröhlich , “The Nature of the Bonding in Transition‐Metal Compounds,” Chemical Reviews 100 (2000): 717–774, 10.1021/cr980401l. 11749249

[9] S. S. Stahl , J. L. Thorman , N. de Silva , I. A. Guzei , and R. W. Clark , ““Inverse‐Electron‐Demand” Ligand Substitution in Palladium(0)−Olefin Complexes,” Journal of the American Chemical Society 125 (2003): 12–13, 10.1021/ja028738z. 12515487

[10] J. B. Johnson and T. Rovis , “More Than Bystanders: The Effect of Olefins on Transition‐Metal‐Catalyzed Cross‐Coupling Reactions,” Angewandte Chemie International Edition 47 (2008): 840–871, 10.1002/anie.200700278. 18081111

[11] G. Kiss , “Palladium‐Catalyzed Reppe Carbonylation,” Chemical Reviews 101 (2001): 3435–3456, 10.1021/cr010328q. 11840990

[12] A. Brennführer , H. Neumann , and M. Beller , “Palladium‐Catalyzed Carbonylation Reactions of Alkenes and Alkynes,” Chemcatchem 1 (2009): 28–41, 10.1002/cctc.200900062. 19431166

[13] R. Franke , D. Selent , and A. Börner , “Applied Hydroformylation,” Chemical Reviews 112 (2012): 5675–5732, 10.1021/cr3001803. 22937803

[14] S. Cai , H. Zhang , and H. Huang , “Transition‐Metal‐Catalyzed Hydroaminocarbonylations of Alkenes and Alkynes,” Trends in Chemistry 3 (2021): 218–230, 10.1016/j.trechm.2020.11.006.

[15] C. P. Folster , R. P. Harkins , S. Lo , J. D. Sachs , and I. A. Tonks , “Development and Applications Of Selective Hydroesterification Reactions,” Trends in Chemistry 3 (2021): 469–484, 10.1016/j.trechm.2021.03.002.

[16] X. Fang , R. Jackstell , and M. Beller , “Selective Palladium‐Catalyzed Aminocarbonylation of Olefins With Aromatic Amines and Nitroarenes,” Angewandte Chemie International Edition 52 (2013): 14089–14093, 10.1002/anie.201308455. 24214903

[17] G. Zhang , B. Gao , and H. Huang , “Palladium‐Catalyzed Hydroaminocarbonylation of Alkenes With Amines: A Strategy to Overcome the Basicity Barrier Imparted by Aliphatic Amines,” Angewandte Chemie International Edition 54 (2015): 7657–7661, 10.1002/anie.201502405. 25959632

[18] K. Dong , X. Fang , S. Gülak , et al., “Highly Active and Efficient Catalysts For Alkoxycarbonylation Of Alkenes,” Nature Communications 8 (2017): 14117, 10.1038/ncomms14117. PMC5288498 28120947

[19] R. Sang , P. Kucmierczyk , R. Dühren , et al., “Synthesis of Carboxylic Acids by Palladium‐Catalyzed Hydroxycarbonylation,” Angewandte Chemie International Edition 58 (2019): 14365–14373, 10.1002/anie.201908451. 31390131

[20] Y. Zhang , S. Torker , M. Sigrist , N. Bregović , and P. Dydio , “Binuclear Pd(I)–Pd(I) Catalysis Assisted by Iodide Ligands for Selective Hydroformylation of Alkenes and Alkynes,” Journal of the American Chemical Society 142 (2020): 18251–18265, 10.1021/jacs.0c09254. 33035057

[21] P. Roesle , L. Caporaso , M. Schnitte , V. Goldbach , L. Cavallo , and S. Mecking , “A Comprehensive Mechanistic Picture of the Isomerizing Alkoxycarbonylation of Plant Oils,” Journal of the American Chemical Society 136 (2014): 16871–16881, 10.1021/ja508447d. 25415929

[22] H. Li , K. Dong , H. Jiao , H. Neumann , R. Jackstell , and M. Beller , “The Scope And Mechanism of Palladium‐Catalysed Markovnikov Alkoxycarbonylation of Alkenes,” Nature Chemistry 8 (2016): 1159–1166, 10.1038/nchem.2586. 27874861

[23] K. Zhao , H. Wang , T. Li , et al., “Identification of a Potent Palladium‐Aryldiphosphine Catalytic System for High‐Performance Carbonylation of Alkenes,” Nature Communications 15 (2024): 2016. 10.1038/s41467-024-46286-9 PMC10914764 38443382

[24] S. Gallarati , P. Dingwall , J. A. Fuentes , M. Bühl , and M. L. Clarke , “Understanding Catalyst Structure–Selectivity Relationships in Pd‐Catalyzed Enantioselective Methoxycarbonylation of Styrene,” Organometallics 39 (2020): 4544–4556, 10.1021/acs.organomet.0c00613.

[25] H. Lu , T. Yu , P. Xu , and H. Wei , “Selective Decarbonylation via Transition‐Metal‐Catalyzed Carbon–Carbon Bond Cleavage,” Chemical Reviews 121 (2021): 365–411, 10.1021/acs.chemrev.0c00153. 32543866

[26] X. Wang , B. Wang , X. Yin , et al., “Palladium‐Catalyzed Enantioselective Thiocarbonylation of Styrenes,” Angewandte Chemie International Edition 58 (2019): 12264–12270, 10.1002/anie.201905905. 31267622

[27] W. Yu , J. Han , D. Fang , M. Wang , and J. Liao , “Palladium‐Catalyzed Linear Hydrothiocarbonylation of Unactivated Terminal Alkenes: Synthesis of Aliphatic Thioesters,” Organic Letters 23 (2021): 2482–2487, 10.1021/acs.orglett.1c00406. 33711895

[28] Q. Tian , X. Yin , R. Sun , X. F. Wu , and Y. Li , “The Lower the Better: Efficient Carbonylative Reactions Under Atmospheric Pressure of Carbon Monoxide,” Coordination Chemistry Reviews 475 (2023): 214900, 10.1016/j.ccr.2022.214900.

[29] J. K. Hoyano , A. D. McMaster , and W. A. G. Graham , “Activation of Methane by Iridium Complexes,” Journal of the American Chemical Society 105 (1983): 7190–7191, 10.1021/ja00362a039.

[30] A. J. Kunin and R. Eisenberg , “Photochemical Carbonylation of Benzene By Iridium(I) and Rhodium(I) Square‐Planar Complexes,” Journal of the American Chemical Society 108 (1986): 535–536, 10.1021/ja00263a045. 22175490

[31] A. A. Bengali , R. H. Schultz , C. B. Moore , and R. G. Bergman , “Activation of the C‐H Bonds in Neopentane and Neopentane‐d12 by (.eta.5‐C5(CH_3_)_5_)Rh(CO)_2_: Spectroscopic and Temporal Resolution of Rhodium‐Krypton and Rhodium‐Alkane Complex Intermediates,” Journal of the American Chemical Society 116 (1994): 9585–9589, 10.1021/ja00100a024.

[32] J. J. Turner , M. W. George , M. Poliakoff , and R. N. Perutz , “Photochemistry of Transition Metal Carbonyls,” Chemical Society Reviews 51 (2022): 5300–5329, 10.1039/D1CS00826A. 35708003

[33] R. M. Jay , M. R. Coates , H. Zhao , et al., “Photochemical Formation and Electronic Structure of an Alkane σ‐Complex From Time‐Resolved Optical and X‐ray Absorption Spectroscopy,” Journal of the American Chemical Society 146 (2024): 14000–14011, 10.1021/jacs.4c02077. 38713061 PMC11117182

[34] M. S. Faculak , A. M. Veatch , and E. J. Alexanian , “Cobalt‐Catalyzed Synthesis Of Amides From Alkenes and Amines Promoted by Light,” Science 383 (2024): 77–81, 10.1126/science.adk2312. 38175889 PMC10799253

[35] M. S. Faculak , M. R. Rodriguez , and E. J. Alexanian , “Cobalt‐Catalyzed Syntheses of Esters and Carboxylic Acids From Alkenes Promoted by Light,” Journal of the American Chemical Society 147 (2025): 14060–14064, 10.1021/jacs.5c03769. 40257115 PMC12122085

[36] S. Sumino , A. Fusano , T. Fukuyama , and I. Ryu , “Carbonylation Reactions of Alkyl Iodides Through the Interplay of Carbon Radicals and Pd Catalysts,” Accounts of Chemical Research 47 (2014): 1563–1574, 10.1021/ar500035q. 24712759

[37] G. M. Torres , Y. Liu , and B. A. Arndtsen , “A Dual Light‐Driven Palladium Catalyst: Breaking the Barriers in Carbonylation Reactions,” Science 368 (2020): 318–323, 10.1126/science.aba5901. 32299954

[38] B. Cai , H. W. Cheo , T. Liu , and J. Wu , “Light‐Promoted Organic Transformations Utilizing Carbon‐Based Gas Molecules as Feedstocks,” Angewandte Chemie International Edition 60 (2021): 18950–18980, 10.1002/anie.202010710. 33002315

[39] Y. Liu , C. Zhou , M. Jiang , and B. A. Arndtsen , “Versatile Palladium‐Catalyzed Approach to Acyl Fluorides and Carbonylations by Combining Visible Light‐ and Ligand‐Driven Operations,” Journal of the American Chemical Society 144 (2022): 9413–9420, 10.1021/jacs.2c01951. 35587132

[40] C. S. MacNeil , L. N. Mendelsohn , T. P. Pabst , G. Hierlmeier , and P. J. Chirik , “Alcohol Synthesis by Cobalt‐Catalyzed Visible‐Light‐Driven Reductive Hydroformylation,” Journal of the American Chemical Society 144 (2022): 19219–19224, 10.1021/jacs.2c07745. 36240429

[41] K. El Chami , Y. Liu , M. A. Belahouane , Y. Ma , P. Lagueux‐Tremblay , and B. A. Arndtsen , Angewandte Chemie International Edition 62 (2023): 202213297. 10.1002/anie.202213297 36576428

[42] P. Chuentragool , D. Kurandina , and V. Gevorgyan , “Catalysis With Palladium Complexes Photoexcited by Visible Light,” Angewandte Chemie International Edition 58 (2019): 11586–11598, 10.1002/anie.201813523. 30600875 PMC6606406

[43] Z. Liu and X. Chen , “Light‐Induced Excited‐State Palladium Catalysis for Challenging Couplings,” Chemistry (Weinheim An Der Bergstrasse, Germany) 6 (2020): 1219–1221, 10.1016/j.chempr.2020.05.015.

[44] P. Boehm , S. Roediger , A. Bismuto , and B. Morandi , “Palladium‐Catalyzed Chlorocarbonylation of Aryl (Pseudo)Halides Through In Situ Generation of Carbon Monoxide,” Angewandte Chemie International Edition 59 (2020): 17887–17896, 10.1002/anie.202005891. 32628290

[45] S. Sarkar , K. P. S. Cheung , and V. Gevorgyan , “Recent Advances In Visible Light Induced Palladium Catalysis,” Angewandte Chemie International Edition 63 (2024): 202311972. 10.1002/anie.202311972 PMC10922525 37957126

[46] C. Che and S. Lai , “Structural and Spectroscopic Evidence for Weak Metal–Metal Interactions and Metal–Substrate Exciplex Formations in d10 Metal Complexes,” Coordination Chemistry Reviews 249 (2005): 1296–1309, 10.1016/j.ccr.2004.11.026.

[47] G. Cera , N. Della Ca' , and G. Maestri , “Palladium(0)/Benzoic Acid Catalysis Merges Sequences With D_2_O‐Promoted Labelling of C–H Bonds,” Chemical Scie 10 (2019): 10297–10304, 10.1039/C9SC03682B. PMC6979390 32110316

[48] Deposition number 2472279 (for **57**), CCDC 2480723 (for **118**) and 2472280 (for **Pd‐C**), contains the crystallographic data for this paper (for details, see Figure S13–S15, Tables S12–S14). These data are provided free of charge by the joint Cambridge Crystallographic Data Centre and Fachinformationszentrum Karlsruhe Access Structures service.

