# Supplementary material for: Ambient Hydrocarbonylation of Olefins Enabled by Visible‐Light
Source: Adv Sci (Weinh). 2026 Feb 10;13(22):e74323. doi: 10.1002/advs.74323 (PMC13088343; doi:10.1002/advs.74323)
Supplement: Supplementary file 1 — Supporting File: advs74323‐sup‐0001‐SuppMat.pdf. [file ADVS-13-e74323-s001.pdf]

Supplementary Information for

Ambient Hydrocarbonylation of Olefins Enabled by Visible-Light

Hongchi Liu[1], Tianze Zhang[1] and Hanmin Huang[1,2]*

[1]State Key Laboratory of Precision and Intelligent Chemistry, Department of Chemistry, University of Science and Technology of China; Hefei, 230026, P. R. China.

[2]Key Laboratory of Green and Precise Synthetic Chemistry and Applications, Ministry of Education, Huaibei Normal University; Huaibei, 235000, P. R. China.

*Corresponding author: hanmin@ustc.edu.cn,

Table of contents

1. General information 3
2. Optimization of the reaction conditions 5
    2.1. Optimization of the reaction conditions for hydroxycarbonylation 5
    2.1. Optimization of the reaction conditions for hydroformylation 10
3. Procedure for the synthesis of modified DPEphos ligands 13
    3.1. Procedure A: synthesis of modified DPEphos ligands 13
    3.2. Experimental characterization data for modified DPEphos ligands 14
4. General procedure and spectral data of the products 16
    4.1.General procedure for preparation of products 16
    4.2. Characterization of products 18
5. Synthetic applications 50
    5.1. Hydrocarbonylation of ethylene 50
    5.2. Hydrocarbonylation of mixture of hexene isomers 51
    5.3. Hydrocarbonylation with bioactive molecules 52
    5.4. Synthesize of dihydroavenanthramide D 54
6. Mechanistic investigations 55
    6.1. Synthesis and characteristic of palladium hydride species 55
    6.2. UV-Vis absorption spectroscopy studies 58
    6.3. Radical trapping experiment 58
    6.4. Radical clock experiment 59
    6.5. Isotopic-labelling experiment 61
    6.6 Stoichiometric experiment of the palladium-hydride species 63
    6.7 Hydrolysis of acylpalladium complex 65
7. X-ray crystallographic data 67
    7.1. X-ray crystallographic data of 56 67
    7.2. X-ray crystallographic data of 117 69
    7.3. X-ray crystal structure of (DPEphos)Pd($COCH_2CH_2Ph$)Cl 71
8. NMR spectra 73
9. Copies of HPLC 356
10. References 358

1. General information

Methods:

All non-aqueous reactions and manipulations were using standard Schlenk techniques. Flash column chromatography was performed using 200-300 mesh silica gels. Thin layer chromatography was used for product detection using silica gel-coated plates, with visualization effected *via* exposure to UV Light (λ_{ex} = 254 nm) or staining with iodine or bromocresol green.

Materials and reagents:

All the reagents and solvents were purchased from commercial suppliers including Energy Chemical, Bidepharm, Adamas and used as received. Anhydrous diethyl ether, acetone and toluene were dried and degassed by standard methods and stored under N_2 atmosphere. Other anhydrous solvents (CH_3CN (acetonitrile), THF (tetrahydrofuran), DCM (dichloromethane), DMF (*N,N*-dimethylformamide), NMP (*N*-methyl-2-pyrrolidone), DMSO (dimethyl sulfoxide),) were purchased from Energy Chemical, stored under N_2 atmosphere and degassed by standard methods before using. Deuterated solvents were purchased from Energy Chemical.

Instrumentation:

Nuclear magnetic resonance spectra (^1H NMR, ^{13}C NMR, ^{19}F NMR, and ^{31}P NMR) were recorded on BRUKER Avance III 400 or 500 MHz NMR spectrometers. Chemical shifts are reported in parts per million (ppm, δ), downfield from tetramethylsilane (TMS, δ = 0.00 ppm) and are referenced to residual solvent ($CDCl_3$, δ = 7.26 ppm (^1H) and 77.16 ppm (^{13}C)). Coupling constants (*J*) were reported in Hertz (Hz) and referred to apparent peak multiplications. Data for ^1H NMR spectra were reported as follows: chemical shift (ppm), multiplicity (s = singlet, d = doublet, t = triplet, q = quartet, dd = doublet of doublets, td = triplet of doublets, m = multiplet, brs = broad singlet), coupling constants (Hz) and integration. High resolution mass spectra (HRMS) were recorded on Bruker MicroTOF-QII mass instrument (ESI). UV-Vis absorption spectroscopy were recorded on Shimadzu UV3600 I plus UV-Vis-NIR Spectrophotometer.

Abbreviations:

Me = methyl, Et = ethyl, ^{i}Pr = isopropyl, ^{t}Bu = *tert*-butyl, Cy = cyclohexyl, Bn = benzyl, Ph = phenyl, Ar = aryl, Bz = Benzoyl, Ts = tosyl, Ph = Phenyl, PhthN = Phthalimido, Cz, carbazolyl, C_4F_9, 2,2,3,3,4,4,5,5,5-nonafluorobutyl, BocHN = tert-butyl carbamate;

DME = 1,2-dimethoxyethane, Et_2O = diethyl ether, EtOAc = ethyl acetate, MeOH = methanol, EtOH = ethanol, DCM = dichloromethane, THF = tetrahydrofuran, DMF = *N,N*-dimethylformamide, NMP = *N*-methyl-2-pyrrolidone, DMSO = dimethyl sulfoxide;

DPEphos = (oxybis(2,1-phenylene))bis(diphenylphosphane);

equiv. = equivalents, g = grams, mg = milligrams, aq. = aqueous, wt.% = weight%, N.D. = Not detected, dr = diastereomer ratio, rr = regioisomer ratio.

Reaction setup:

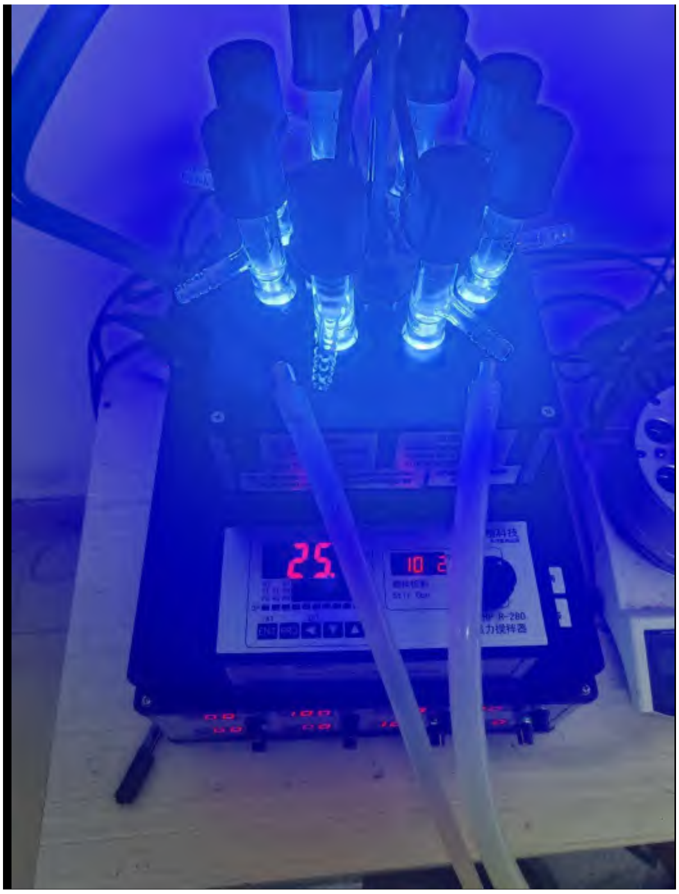

Figure S1. General reaction setup: The Young-type tubes were irradiated using 15W LED lamps, with the temperature controlled at 25°C through circulating cooling.

2. Optimization of the reaction conditions

2.1. Optimization of the reaction conditions for hydroxycarbonylation

To a flame-dried Young-type tube was added palladium precursor (0.020 mmol, 5 mol% of palladium) and ligand (0.024 mmol, 6 mol%). The tube was evacuated and refilled with N_2, then olefin (0.40 mmol, 1.0 equiv.), H_2O (144 mg, 8 mmol, 20 equiv.), acid (0.040 mmol, 10 mol%), additive (0.020 mmol, 5 mol%) and solvent (2.0 mL) were added under the N_2 atmosphere. After that, the resulting mixture was degassed with the freeze-thaw method, introduced 1 atm CO and irradiated under 15W 455 nm blue LEDs for 20 hours. After completion, the reaction mixture was concentrated under reduced pressure. The residue was purified by silica gel column chromatography to afford the desired product.

Table S1. Screening of solvent.

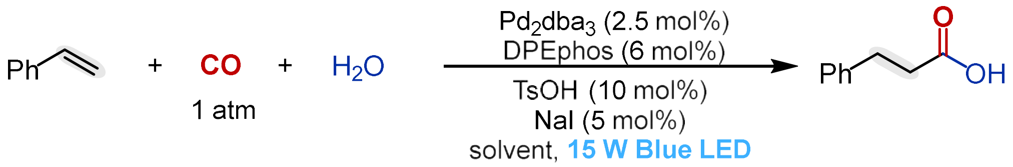

entry	solvent	yield (%)	l/b
1	dioxane	89(87*)	> 20:1
2	THF	81	> 20:1
3	DME	51	> 20:1
4	anisole	86	> 20:1
5	CH_3CN	65	> 20:1
6	acetone	80	> 20:1
7	DMF	41	> 20:1
8	NMP	63	> 20:1
9	DMSO	57	12:1

Reaction conditions: Styrene (0.4 mmol), CO (1 atm), H_2O (20 equiv.), Pd_2dba_3 (2.5 mol%), DPEphos (6 mol%), TsOH (10 mol%), NaI (5 mol%) in 2.0 mL solvent, r.t., 455 nm blue LED for 24 h. Determined by GC using tetradecane as the internal standard. *Isolated yield.

Table S2. Screening of palladium precursor.

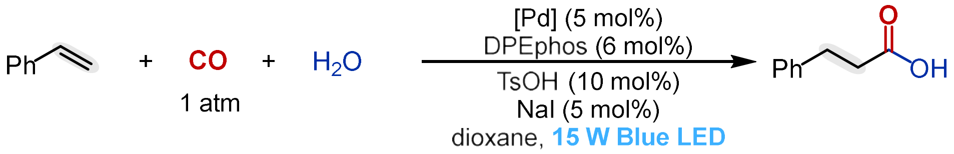

entry	palladium precursor	yield (%)	l/b
1	Pd_2dba_3	89(87*)	> 20:1
2	$Pd(PPh_3)_4$	57	> 20:1
3	$Pd(OAc)_2$	73	> 20:1
4	$Pd(acac)_2$	87	> 20:1
5	$Pd(CH_3CN)Cl_2$	67	> 20:1
6	$[Pd(allyl)Cl]_2$	78	> 20:1
7	$PdBr_2$	38	> 20:1
8	$Pd(cod)Br_2$	47	> 20:1
9	PdI_2	17	> 20:1

Reaction conditions: Styrene (0.4 mmol), CO (1 atm), H_2O (20 equiv.), [Pd] (5 mol% of palladium), DPEphos (6 mol%), TsOH (10 mol%), NaI (5 mol%) in 2.0 mL dioxane, r.t., 455 nm blue LED for 24 h. Determined by GC using tetradecane as the internal standard. *Isolated yield.

Table S3. Screening of ligand**.**

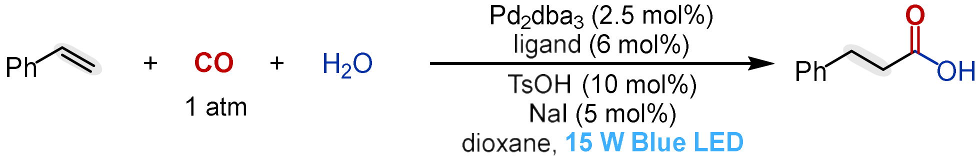

entry	ligand	yield (%)	l/b
1	DPEphos	89(87*)	> 20:1
2	Xantphos	63	> 20:1
3	DPPE	trace	-
4	PPh_3	12	> 20:1
5	BINAP	trace	-
6	DPPF	N.D.	-

Reaction conditions: Styrene (0.4 mmol), CO (1 atm), H_2O (20 equiv.), Pd_2dba_3 (2.5 mol%), ligand (6 mol%), TsOH (10 mol%), NaI (5 mol%) in 2.0 mL dioxane, r.t., 455 nm blue LED for 24 h. Determined by GC using tetradecane as the internal standard. *Isolated yield.

Table S4. Screening of acid.

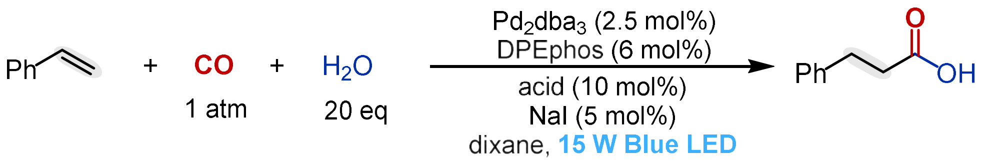

entry	acid	yield (%)	l/b
1	TsOH	89(87*)	> 20:1
2	$PhSO_3H$	67	> 20:1
3	$MeSO_3H$	69	> 20:1
4	(1R)-(-)-10-Camphorsulfonic acid	68	> 20:1
5	HCl	73	> 20:1
6	AcOH	67	> 20:1
7	TfOH	67	> 20:1

Reaction conditions: Styrene (0.4 mmol), CO (1 atm), H_2O (20 equiv.), Pd_2dba_3 (5 mol%), DPEphos (6 mol%), acid (10 mol%), NaI (5 mol%) in 2.0 mL dioxane, r.t., 455 nm blue LED for 24 h. Determined by GC using tetradecane as the internal standard. *Isolated yield.

Table S5. Screening of additives.

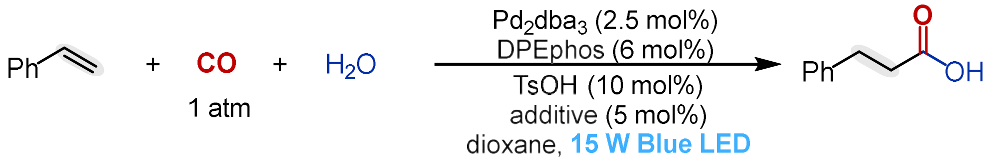

entry	additive	yield (%)	l/b
1	/	18	4.3:1
2	NaI	89(87*)	> 20:1
3	KI	88	> 20:1
4	TBAI	87	> 20:1
5	EtI	83	> 20:1
6	NaCl	20	5:1
7	NaBr	32	8:1
8	NaOAc	trace	-
7	NaOCN	N.D.	-
8	NaSCN	N.D.	-

Reaction conditions: Styrene (0.4 mmol), CO (1 atm), H_2O (20 equiv.), Pd_2dba_3 (5 mol%), DPEphos (6 mol%), TsOH (10 mol%), additive (5 mol%) in 2.0 mL dioxane, r.t., 455 nm blue LED for 24 h. Determined by GC using tetradecane as the internal standard. *Isolated yield.

Table S6. Optimization of irradiation wavelength.

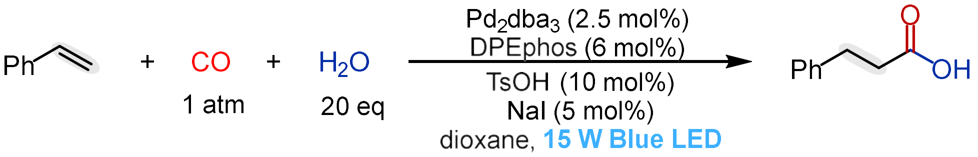

entry	irradiation wavelength	yield (%)	l/b
1	525 nm LED	63	> 20:1
2	420 nm LED	76	13:1
3	380 nm LED	43	12:1
4	dark	N.D.	-

Reaction conditions: Styrene (0.4 mmol), CO (1 atm), H_2O (20 equiv.), Pd_2dba_3 (5 mol%), DPEphos (6 mol%), TsOH (10 mol%), NaI (5 mol%) in 2.0 mL dioxane 455 nm blue LED for 24 h. Determined by GC using tetradecane as the internal standard.

Table S7. Optimization of modified DPEphos with 1-octene.

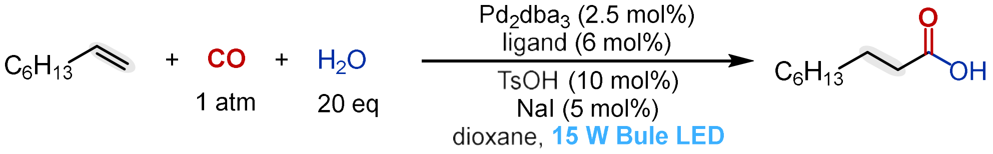

entry	ligand	yield (%)	l/b
1	DPEphos	79	10:1
2	**L2**	80(79*)	> 20:1
3	**L3**	60	7.5:1
4	**L4**	79	20:1
5	**L5**	80	8.6:1

Reaction conditions: 1-octene (0.4 mmol), CO (1 atm), H_2O (20 equiv.), Pd_2dba_3 (2.5 mol%), ligand (6 mol%), TsOH (10 mol%), NaI (5 mol%) in 2.0 mL dioxane 455 nm blue LED for 24 h. Determined by GC using tetradecane as the internal standard.
*Isolated yield.

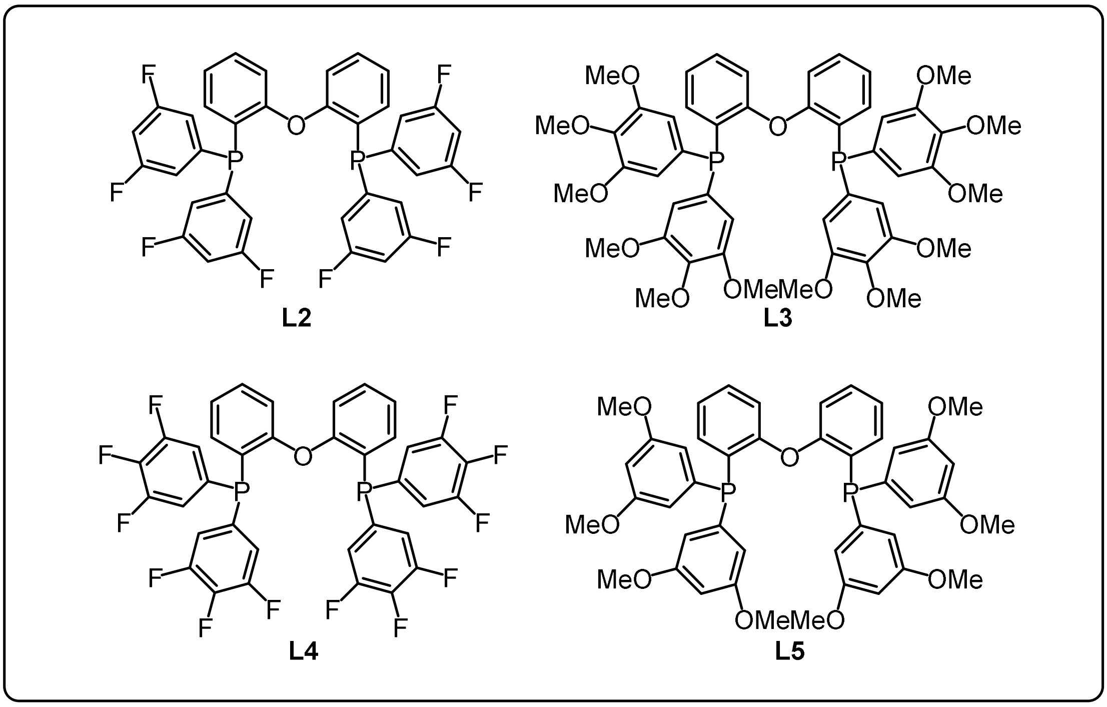

Figure S2. Structures of modified DPEphos ligands

2.1. Optimization of the reaction conditions for hydroformylation

To a flame-dried Young-type tube was added palladium precursor (0.005 mmol, 2.5 mol% of palladium) and ligand (0.004 mmol, 2 mol%). The tube was evacuated and refilled with N_2, then olefin (0.20 mmol, 1.0 equiv.) and solvent (1.0 mL) were added under the N_2 atmosphere. After that, the resulting mixture was degassed with the freeze-thaw method, introduced 1 atm CO/H_2 and irradiated under 15W 455 nm blue LEDs for 20 hours. After completion, the yield and regioselectivity were measured by GC-FID using tetradecane as the internal standard.

Table S8. Screening of solvent.

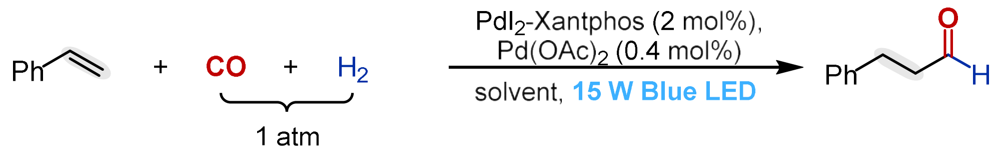

entry	solvent	yield (%)	l/b
1	DCE	51	> 20:1
2	DCM	41	> 20:1
3	THF	N.D.	-
4	dioxane	N.D.	-
5	DME	N.D.	-
6	CH_3CN	44	> 20:1
7	acetone	23	> 20:1
8	Toluene	N.D.	-
9	DMA	N.D.	-

Reaction conditions: Styrene (0.2 mmol), CO/H_2 = 1:3 (1 atm), PdI_2-Xantphos (2 mol%), $Pd(OAc)_2$ (0.4 mol%), 1.0 mL solvent, r.t., 455 nm blue LED for 10 h. Determined by GC using tetradecane as the internal standard.

Table S9. Optimization of the CO/H_2 ratio.

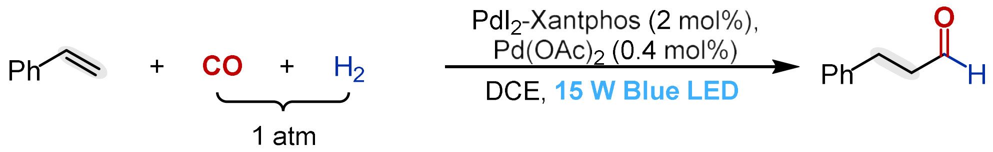

entry	CO/H_2	yield (%)	l/b
1	1:3	52	> 20:1
2	1:2	41	> 20:1
3	1:1	28	> 20:1
4	2:1	28	> 20:1
5	3:1	18	> 20:1

Reaction conditions: Styrene (0.2 mmol), CO/H_2 = x:y (1 atm), PdI_2-Xantphos (2 mol%), $Pd(OAc)_2$ (0.4 mol%), 1.0 mL DCE, r.t., 455 nm blue LED for 10 h. Determined by GC using tetradecane as the internal standard.

Table S10. Screening of ligand**.**

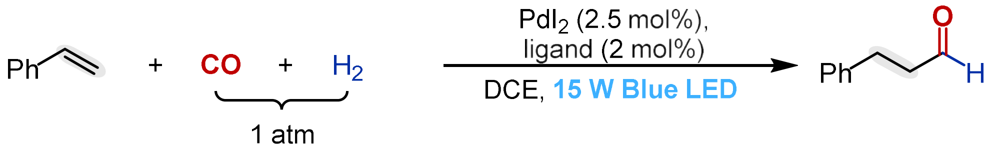

entry	ligand	yield (%)	l/b
1	Xantphos	73	> 20:1
2	DPEphos	28	> 20:1
3	DPPE	N.D.	-
4	PPh_3	N.D.	-
5	BINAP	N.D.	-
6	DPPF	N.D.	-

Reaction conditions: Styrene (0.2 mmol), CO/H_2 = 1:3 (1 atm), PdI_2 (2.5 mol%), ligand (2 mol%), 1.0 mL DCE, r.t., 455 nm blue LED for 10 h. Determined by GC using tetradecane as the internal standard.

Table S11. Screening of palladium precursor.

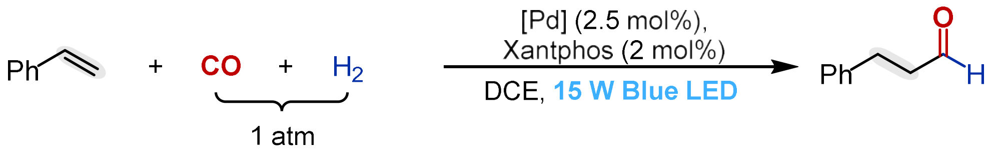

entry	palladium precursor	yield (%)	l/b
1	PdI_2	73	> 20:1
2	Pd_2dba_3	N.D.	-
3	$Pd(OAc)_2$	N.D.	-
4	$Pd(acac)_2$	N.D.	-
5	$PdCl_2$	N.D.	-
6	$[Pd(allyl)Cl]_2$	N.D.	-
7	$PdBr_2$	N.D.	-
8	$Pd(cod)Br_2$	N.D.	-

Reaction conditions: Styrene (0.2 mmol), CO/H_2 = 1:3 (1 atm), [Pd] (2.5 mol%), Xantphos (2 mol%), 1.0 mL DCE, r.t., 455 nm blue LED for 10 h. Determined by GC using tetradecane as the internal standard.

3. Procedure for the synthesis of modified DPEphos ligands

3.1. Procedure A: synthesis of modified DPEphos ligands

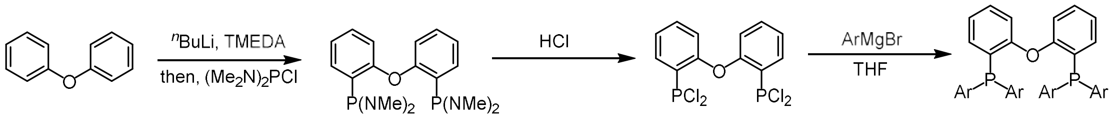

The compound was prepared according to the literature[1]. Into a flask charged with n-BuLi (25.08 ml, 48.4 mmol, 1.93M) placed in a water bath at 23 °C, TMEDA (7.31 ml, 48.8 mmol) was added dropwise with rapid stirring. After addition is finished, the mixture was placed in an ice-water bath, and a THF (11.75 ml) solution of phenyl ether (3.73 ml, 23.50 mmol) was added dropwise with stirring. The mixture was warmed up and stirred at rt for 16 hours and then cooled to –78°C. A THF (23.50 ml) solution of bis(diethylamino)chlorophosphine (10.20 g, 48.4 mmol) was added to the above reaction mixture over 1 hour with stirring at –78 °C. After addition was complete, the resulitng mixture was warmed and allowed to stir at 23 °C for 16 hours. The mixture was filtered under N_2 in the glovebox, and then concentrated under vacuum to give the product (12.19 g, 23.50 mmol) as a viscous deep red liquid which was used in the next step without further purification.

Crude 1,1'-(oxybis(2,1-phenylene))bis(*N,N,N',N'*-tetraethylphosphinediamine) (12.19 g, 23.50 mmol) was dissolved in Et_2O and cooled to –78 °C. Hydrochloric acid (2M in Et_2O) (150 ml, 300 mmol) was added to the reaction mixture at –78 °C over 2 hours. The reaction mixture was then warmed up and stirred at 23 °C for 12 h. The mixture was filtered under N_2 with a Schlenk filter, and then concentrated under vacuum to give a yellow oil, which was then carried on to the next step without purification.

Part of the crude (oxybis(2,1-phenylene))bis(dichlorophosphine) (2.00 g, 5.38 mmol) was dissolved in THF (Volume: 90 ml) and the reaction cooled down to –78 °C. Grignard reagent, 1M solution in THF (37.6 ml, 37.6 mmol) was then added batchwise over 1 hour and the reaction was allowed to stir at –78 °C for 1 hour. The temperature was then raised to 0°C and the reaction allowed to stir for 2 hours. The reaction was then allowed to warm to 23°C and allowed to stir for 16 hours.

The reaction was then cooled to 0°C and then quenched by the addition of saturated aqueous NH_4Cl (25 mL). The mixture was then warmed to room temperature and then extracted with Et_2O (2x60 mL) and CH_2Cl_2 (50 ml) sequentially. The combined organic layers were dried with Na_2SO_4 and then concentrated under reduced pressure. The crude product was then purified by by flash chromatography on silica gel to afford the ligand as a white solid.

3.2. Experimental characterization data for modified DPEphos ligands

(oxybis(2,1-phenylene))bis(bis(3,5-difluorophenyl)phosphane) (L2)

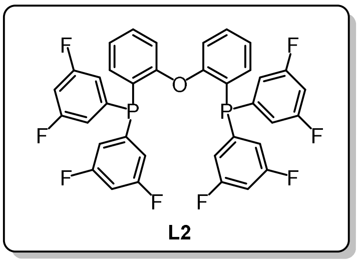

The title compound was prepared according to **Procedure A** and purified by column chromatography (PE/EA = 50:1) as a white solid (1.65 g, 47% yield). **^{1}H NMR** (400 MHz, $CDCl_3$) δ 7.34 (td, *J* = 7.8, 1.7 Hz, 2H), 7.07 (td, *J* = 7.5, 1.0 Hz, 2H), 6.82 – 6.65 (m, 16H). **^{13}C NMR** (126 MHz, $CDCl_3$) δ 163.9 (t, *J* = 10.9 Hz), 161.9 (t, *J* = 10.9 Hz), 139.9 (dt, *J* = 17.4, 6.4 Hz), 134.0 (d, *J* = 3.5 Hz), 131.6, 125.8 (d, *J* = 15.2 Hz), 124.6, 118.1, 116.0 (td, *J* = 20.5, 19.7, 5.7 Hz), 104.8 (t, *J* = 25.3 Hz). **^{31}P NMR** (202 MHz, $CDCl_3$) δ -12.4. **^{19}F NMR** (471 MHz, $CDCl_3$) δ -108.7. **HRMS** (ESI) calcd. for $C_{36}H_{21}OF_8P_2$ $[M+H]^+$: 683.0934, found: 683.0930

(oxybis(2,1-phenylene))bis(bis(3,4,5-trimethoxyphenyl)phosphane) (L3)

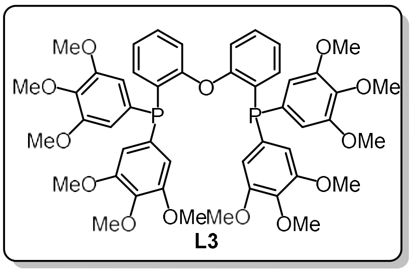

The title compound was prepared according to **Procedure A** and purified by column chromatography (PE/EA = 2:1) as a white solid (1.79 g, 37% yield). **^{1}H NMR** (400 MHz, $CDCl_3$) δ 7.20 (t, *J* = 7.7 Hz, 2H), 7.00 (t, *J* = 7.5 Hz, 2H), 6.83 (dd, *J* = 8.0, 3.9 Hz, 2H), 6.66 (dd, *J* = 8.1, 3.9 Hz, 2H), 6.50 (d, *J* = 7.5 Hz, 8H), 3.84 (s, 12H), 3.71 (s, 24H). **^{13}C NMR** (101 MHz, $CDCl_3$) δ 153.1 (d, *J* = 1.7 Hz), 153.1, 153.0 (d, *J* = 1.7 Hz), 138.5, 133.8, 131.6, 131.5, 130.2, 123.8, 117.6, 110.8 (d, *J* = 23.8 Hz), 60.9, 56.1. **^{31}P NMR** (162 MHz, $CDCl_3$) δ -11.2. **HRMS** (ESI) calcd. for $C_{48}H_{53}O_{13}P_2$ $[M+H]^+$: 899.2956, found: 899.2932

(oxybis(2,1-phenylene))bis(bis(3,4,5-trifluorophenyl)phosphane) (L4)

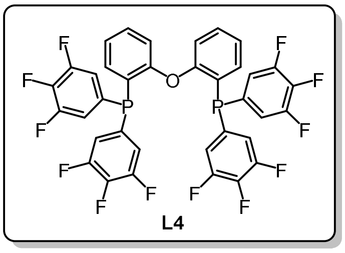

The title compound was prepared according to **Procedure A** and purified by column chromatography (PE/EA = 50:1) as a white solid (1.58 g, 39% yield). **^{1}H NMR** (400 MHz, $CDCl_3$) δ 7.38 (td, *J* = 7.8, 1.7 Hz, 2H), 7.12 (td, *J* = 7.5, 1.1 Hz, 2H), 6.95 – 6.60 (m, 12H). **^{13}C NMR** (126 MHz, $CDCl_3$) δ 158.3 (d, *J* = 17.0 Hz), 152.4 (td, *J* = 10.3, 2.6 Hz), 150.3 (dt, *J* = 10.0, 5.4 Hz), 141.4 (t, *J* = 15.2 Hz), 139.3 (t, *J* = 15.1 Hz), 133.8 (d, *J* = 3.6 Hz), 131.9, 124.9, 118.2, 117.4 (ddd, *J* = 23.0, 15.9, 4.9 Hz). **^{31}P NMR** (202 MHz, $CDCl_3$) δ -11.9. **^{19}F NMR** (471 MHz, $CDCl_3$) δ -132.5 (d, *J* = 20.1 Hz), -157.5. **HRMS** (ESI) calcd. for $C_{36}H_{17}OF_{12}P_2$ $[M+H]^+$: 755.0558, found: 755.0554

(oxybis(2,1-phenylene))bis(bis(3,5-dimethoxyphenyl)phosphane) (L5)

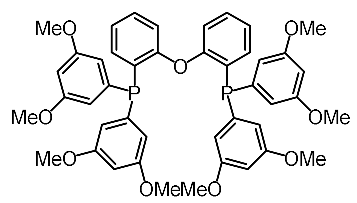

L5

The title compound was prepared according to **Procedure A** and purified by column chromatography (PE/EA = 4:1) as a white solid (1.88 g, 45% yield). **^{1}H NMR** (400 MHz, $CDCl_3$) δ 7.23 – 7.16 (m, 2H), 6.96 (td, *J* = 7.4, 1.1 Hz, 2H), 6.81 (ddd, *J* = 7.6, 4.0, 1.7 Hz, 2H), 6.72 (ddd, *J* = 8.2, 4.6, 1.1 Hz, 2H), 6.39 (dd, *J* = 8.1, 2.3 Hz, 8H), 6.35 (t, *J* = 2.3 Hz, 4H), 3.69 (s, 24H). **^{13}C NMR** (101 MHz, $CDCl_3$) δ 160.4 (d, *J* = 9.6 Hz), 159.3 (d, *J* = 18.7 Hz), 138.7 (d, *J* = 12.4 Hz), 133.9, 130.3, 128.5 (d, *J* = 15.8 Hz), 123.7, 118.0, 111.5 (d, *J* = 22.3 Hz), 101.0. **^{31}P NMR** (162 MHz, $CDCl_3$) δ -12.4. **HRMS** (ESI) calcd. for $C_{44}H_{45}O_9P_2$ $[M+H]^+$: 779.2533, found: 779.2524

4. General procedure and spectral data of the products

4.1.General procedure for preparation of products

General procedure A:

To a flame-dried Young-type tube was added Pd_2dba_3 (9.1 mg, 0.010 mmol, 2.5 mol%) and ligand (0.024 mmol, 6 mol%). The tube was evacuated and refilled with N_2, then olefin (0.40 mmol, 1.0 equiv.), H_2O (144 mg, 8 mmol, 20 equiv.), TsOH·H_2O (7.6 mg, 0.040 mmol, 10 mol%), NaI (3.0 mg, 0.020 mmol, 5 mol%) and dioxane (2.0 mL) were added under the N_2 atmosphere. After that, the resulting mixture was degassed with the freeze-thaw method, introduced 1 atm CO and irradiated under 15W 455 nm blue LEDs at 25°C for 20 hours. Two parallel reactions were set up. After completion, the reaction mixture was concentrated under reduced pressure. The residue was purified by silica gel column chromatography to afford the desired product. The regioselectivity of the product was determined by ^1H NMR analysis.

General procedure B:

To a flame-dried Young-type tube was added Pd_2dba_3 (9.1 mg, 0.010 mmol, 2.5 mol%) and ligand (0.024 mmol, 6 mol%). The tube was evacuated and refilled with N_2, then 2-(pent-4-en-1-yl)isoindoline-1,3-dione (86 mg, 0.40 mmol, 1.0 equiv.), alcohol (2 mmol, 5 equiv.), TsOH·H_2O (7.6 mg, 0.040 mmol, 10 mol%), NaI (3.0 mg, 0.020 mmol, 5 mol%) and dioxane (2.0 mL) were added under the N_2 atmosphere. After that, the resulting mixture was degassed with the freeze-thaw method, introduced 1 atm CO and irradiated under 15W 455 nm blue LEDs at 25°C for 20 hours. After completion, the reaction mixture was concentrated under reduced pressure. The residue was purified by silica gel column chromatography to afford the desired product. The regioselectivity of the product was determined by ^1H NMR analysis.

General procedure C:

To a flame-dried Young-type tube was added Pd_2dba_3 (9.1 mg, 0.010 mmol, 2.5 mol%) and DPEphos (13 mg 0.024 mmol, 6 mol%). The tube was evacuated and refilled with N_2, then 2-(pent-4-en-1-yl)isoindoline-1,3-dione (86 mg, 0.40 mmol, 1.0 equiv.), alcohol (0.8 mmol, 2 equiv.), TsOH·H_2O (7.6 mg, 0.040 mmol, 10 mol%), NaI (3.0 mg, 0.020 mmol, 5 mol%) and dioxane (2.0 mL) were added under the N_2 atmosphere. After that, the resulting mixture was degassed with the freeze-thaw method, introduced 1 atm CO and irradiated under 15W 455 nm blue LEDs at 25°C for 20 hours. After completion, the reaction mixture was concentrated under reduced pressure. The residue was purified by silica gel column chromatography to afford the desired product. The regioselectivity of the product was determined by ^1H NMR analysis.

General procedure D:

To a flame-dried Young-type tube was added Pd_2dba_3 (9.1 mg, 0.010 mmol, 2.5 mol%) and ligand (0.024 mmol, 6 mol%). The tube was evacuated and refilled with N_2,

then 2-(pent-4-en-1-yl)isoindoline-1,3-dione (86 mg, 0.40 mmol, 1.0 equiv.), amine/thiol (0.60 mmol, 1.5 equiv.), TsOH·H_2O (7.6 mg, 0.040 mmol, 10 mol%), NaI (3.0 mg, 0.020 mmol, 5 mol%) and dioxane (2.0 mL) were added under the N_2 atmosphere. After that, the resulting mixture was degassed with the freeze-thaw method, introduced 1 atm CO and irradiated under 15W 455 nm blue LEDs at 25°C for 20 hours. After completion, the reaction mixture was concentrated under reduced pressure. The residue was purified by silica gel column chromatography to afford the desired product. The regioselectivity of the product was determined by ^{1}H NMR analysis.

General procedure E:

To a flame-dried Young-type tube was added PdI_2 (1.8 mg, 0.005 mmol, 2.5 mol%) and Xantphos (2.3 mg, 0.004 mmol, 2 mol%). The tube was evacuated and refilled with N_2, then olefin (0.20 mmol, 1.0 equiv.) and DCE (1.0 mL) were added under the N_2 atmosphere. After that, the resulting mixture was degassed with the freeze-thaw method, introduced 1 atm gas mixture (CO:H_2 = 1:3) and irradiated under 15W 455 nm blue LEDs at 25°C for 24 hours. Two parallel reactions were set up. After completion, the regioselectivity were measured by GC-FID using tetradecane as the internal standard. The reaction mixture was concentrated under reduced pressure and purified by silica gel column chromatography to afford the desired product.

General procedure F:

To a flame-dried Young-type tube was added PdI_2 (1.8 mg, 0.005 mmol, 2.5 mol%) DPEphos (3.2 mg, 0.006 mmol, 3 mol%) and K_3PO_4 (0.8 mg, 0.004 mmol, 2 mol%). The tube was evacuated and refilled with N_2, then olefin (0.20 mmol, 1.0 equiv.) and DCE (1.0 mL) were added under the N_2 atmosphere. After that, the resulting mixture was degassed with the freeze-thaw method, introduced 1 atm gas mixture (CO:H_2 = 1:3) and irradiated under 15W 455 nm blue LEDs at 10°C for 24 hours. Two parallel reactions were set up. After completion, the regioselectivity were measured by GC-FID using tetradecane as the internal standard. The reaction mixture was concentrated under reduced pressure and purified by silica gel column chromatography to afford the desired product.

4.2. Characterization of products

3-phenylpropanoic acid (1)

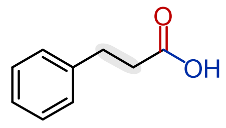

1

The title compound was prepared according to **general procedure A** using DPEphos as ligand and purified by column chromatography (PE/EA = 5:1) as a white solid (104 mg, 87% yield, l/b > 20:1). **^{1}H NMR** (500 MHz, $CDCl_3$) δ 10.42 (s, 1H), 7.32 – 7.26 (m, 2H), 7.24 – 7.17 (m, 3H), 2.95 (t, *J* = 7.8 Hz, 2H), 2.68 (t, *J* = 7.8 Hz, 2H). **^{13}C NMR** (126 MHz, $CDCl_3$) δ 179.5, 140.3, 128.7, 128.4, 126.5, 35.8, 30.7.

The structure was confirmed according to the precedents reported in the literature.[15]

3-(*p*-tolyl)propanoic acid (2)

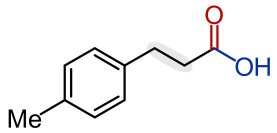

2

The title compound was prepared according to **general procedure A** using DPEphos as ligand and purified by column chromatography (PE/EA = 5:1) as a white solid (101 mg, 77% yield, l/b > 20:1). **^{1}H NMR** (400 MHz, $CDCl_3$) δ 11.17 (s, 1H), 7.20 – 7.00 (m, 4H), 2.91 (td, *J* = 7.9, 6.6, 3.7 Hz, 2H), 2.66 (tdd, *J* = 7.4, 3.4, 1.7 Hz, 2H), 2.38 – 2.23 (m, 3H). **^{13}C NMR** (101 MHz, $CDCl_3$) δ 179.5, 137.1, 135.9, 129.3, 128.2, 35.8, 30.2, 21.1.

The structure was confirmed according to the precedents reported in the literature.[20]

3-(4-(tert-butyl)phenyl)propanoic acid (3)

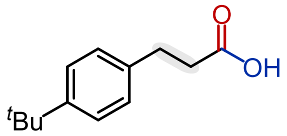

3

The title compound was prepared according to **general procedure A** using DPEphos as ligand and purified by column chromatography (PE/EA = 100:1) as a white solid (132 mg, 80% yield, l/b > 20:1). **^{1}H NMR** (400 MHz, $CDCl_3$) δ 11.55 (s, 1H), 7.33 – 7.29 (m, 2H), 7.15 – 7.10 (m, 2H), 2.96 – 2.87 (m, 2H), 2.70 – 2.61 (m, 2H), 1.29 (s, 9H). **^{13}C NMR** (101 MHz, $CDCl_3$) δ 179.4, 149.2, 137.1, 127.9, 125.5, 35.6, 34.4, 31.4, 30.0.

The structure was confirmed according to the precedents reported in the literature.[2]4

3-(4-methoxyphenyl)propanoic acid (4)

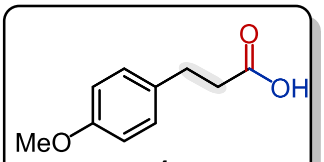

4

The title compound was prepared according to **general procedure A** using DPEphos as ligand and purified by column chromatography (PE/EA = 4:1) as a white solid (95 mg, 65% yield, l/b > 20:1). **^{1}H NMR** (400 MHz, $CDCl_3$) δ 11.09 (s, 1H), 7.16 – 7.09 (m, 2H), 6.87 – 6.79 (m, 2H), 3.77 (s, 3H), 2.89 (t, *J* = 7.7 Hz, 2H), 2.64 (t, *J* = 7.8 Hz, 2H). **^{13}C NMR** (101 MHz, $CDCl_3$) δ 179.5, 158.1, 132.3, 129.3, 114.0, 55.3, 36.0, 29.8.

The structure was confirmed according to the precedents reported in the literature.[20]

3-(4-fluorophenyl)propanoic acid (5)

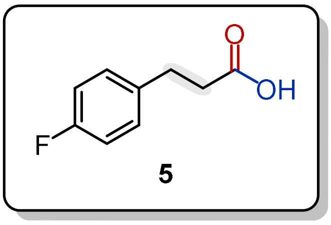

The title compound was prepared according to **general procedure A** using DPEphos as ligand and purified by column chromatography (PE/EA = 5:1) as a white solid (96 mg, 71% yield, l/b > 20:1). ^{1}**H NMR** (400 MHz, $CDCl_3$) δ 11.89 (s, 1H), 7.20 – 7.10 (m, 2H), 7.00 – 6.91 (m, 2H), 2.91 (t, *J* = 7.7 Hz, 2H), 2.64 (t, *J* = 7.7 Hz, 2H). ^{13}**C NMR** (101 MHz, $CDCl_3$) δ 179.6, 161.6 (d, *J* = 244.2 Hz), 135.8 (d, *J* = 3.2 Hz), 129.8 (d, *J* = 7.9 Hz), 115.4 (d, *J* = 21.2 Hz)., 35.8, 29.8. ^{19}**F NMR** (471 MHz, $CDCl_3$) δ -116.79.
The structure was confirmed according to the precedents reported in the literature.[20]

3-(4-chlorophenyl)propanoic acid (6)

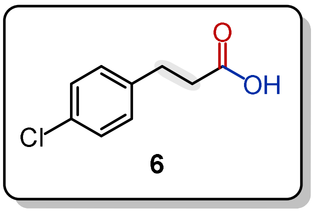

The title compound was prepared according to **general procedure A** using DPEphos as ligand and purified by column chromatography (PE/EA = 5:1) as a white solid (101 mg, 69% yield, l/b > 20:1). ^{1}**H NMR** (500 MHz, $CDCl_3$) δ 10.51 (s, 1H), 7.25 (d, *J* = 8.2 Hz, 2H), 7.13 (d, *J* = 8.0 Hz, 2H), 2.92 (t, *J* = 7.6 Hz, 2H), 2.67 (t, *J* = 7.6 Hz, 2H). ^{13}**C NMR** (126 MHz, $CDCl_3$) δ 179.2, 138.7, 132.3, 129.8, 128.8, 35.7, 30.0.
The structure was confirmed according to the precedents reported in the literature.[20]

3-(4-bromophenyl)propanoic acid (7)

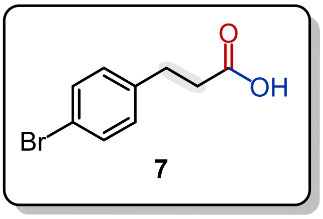

The title compound was prepared according to **general procedure A** using DPEphos as ligand and purified by column chromatography (PE/EA = 5:1) as a white solid (133 mg, 73% yield, l/b > 20:1). ^{1}**H NMR** (400 MHz, $CDCl_3$) δ 11.13 (s, 1H), 7.45 – 7.37 (m, 2H), 7.12 – 7.05 (m, 2H), 2.90 (t, *J* = 7.6 Hz, 2H), 2.66 (t, *J* = 7.6 Hz, 2H). ^{13}**C NMR** (101 MHz, $CDCl_3$) δ 179.0, 139.1, 131.7, 130.1, 120.2, 35.4, 29.9.
The structure was confirmed according to the precedents reported in the literature.[21]

3-(4-(trifluoromethyl)phenyl)propanoic acid (8)

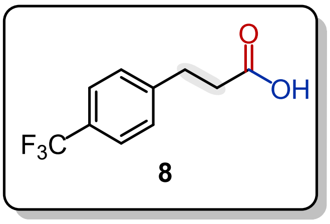

The title compound was prepared according to **general procedure A** using DPEphos as ligand and purified by column chromatography (PE/EA = 5:1) as a white solid (131 mg, 75% yield, l/b > 20:1). ^{1}**H NMR** (400 MHz, $CDCl_3$) δ 11.47 (s, 1H), 7.56 (d, *J* = 8.0 Hz, 2H), 7.32 (s, 2H), 3.02 (t, *J* = 7.6 Hz, 2H), 2.72 (t, *J* = 7.6 Hz, 2H). ^{13}**C NMR** (101 MHz, $CDCl_3$) δ 179.1, 144.2 (d, *J* = 1.4 Hz), 125.5 (d, *J* = 3.8 Hz), 125.5 (d, *J* = 11.3 Hz), 122.9 (t, *J* = 271.8 Hz), 35.1, 30.2. ^{19}**F NMR** (376 MHz, $CDCl_3$) δ - 62.40.
The structure was confirmed according to the precedents reported in the literature.[2]4

3-(4-(methoxycarbonyl)phenyl)propanoic acid (9)

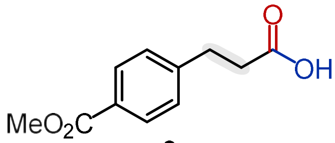

9

The title compound was prepared according to **general procedure A** using DPEphos as ligand and purified by column chromatography (PE/EA = 3:1) as a white solid (125 mg, 75% yield, l/b > 20:1). **^{1}H NMR** (500 MHz, $CDCl_3$) δ 10.38 (s, 1H), 7.97 (d, *J* = 7.9 Hz, 2H), 7.28 (d, *J* = 7.8 Hz, 2H), 3.90 (s, 3H), 3.01 (t, *J* = 7.8 Hz, 2H), 2.71 (t, *J* = 7.7 Hz, 2H). **^{13}C NMR** (126 MHz, $CDCl_3$) δ 178.6, 167.1, 145.6, 129.9, 128.4, 52.1, 35.2, 30.5.

The structure was confirmed according to the precedents reported in the literature.[20]

3-(*o*-tolyl)propanoic acid (10)

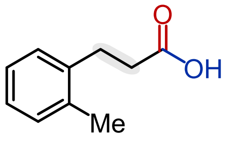

10

The title compound was prepared according to **general procedure A** using DPEphos as ligand and purified by column chromatography (PE/EA = 5:1) as a white solid (106 mg, 81% yield, l/b > 20:1). **^{1}H NMR** (400 MHz, $CDCl_3$) δ 11.75 (s, 1H), 7.22 – 7.05 (m, 4H), 2.94 (dd, *J* = 9.0, 7.0 Hz, 2H), 2.63 (dd, *J* = 9.0, 7.0 Hz, 2H), 2.31 (s, 3H). **^{13}C NMR** (101 MHz, $CDCl_3$) δ 179.8, 138.3, 136.0, 130.4, 128.5, 126.6, 126.3, 34.5, 28.0, 19.3.

The structure was confirmed according to the precedents reported in the literature.[20]

3-(*m*-tolyl)propanoic acid (11)

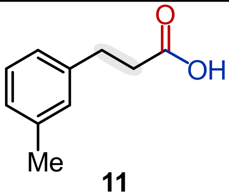

11

The title compound was prepared according to **general procedure A** using DPEphos as ligand and purified by column chromatography (PE/EA = 5:1) as a white solid (108 mg, 82% yield, l/b > 20:1). **^{1}H NMR** (400 MHz, $CDCl_3$) δ 11.16 (s, 1H), 7.19 (t, *J* = 7.7 Hz, 1H), 7.06 – 6.96 (m, 3H), 2.92 (t, *J* = 7.8 Hz, 2H), 2.68 (t, *J* = 7.8 Hz, 2H), 2.33 (s, 3H). **^{13}C NMR** (101 MHz, $CDCl_3$) δ 179.1, 140.1, 138.2, 129.1, 128.5, 127.1, 125.3, 35.6, 30.5, 21.4.

The structure was confirmed according to the precedents reported in the literature.[11]

3-(naphthalen-2-yl)propanoic acid (12)

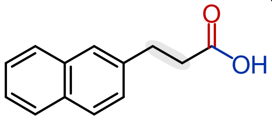

12

The title compound was prepared according to **general procedure A** using DPEphos as ligand and purified by column chromatography (PE/EA = 100:1) as a white solid (106 mg, 66% yield, l/b > 20:1). **^{1}H NMR** (400 MHz, $CDCl_3$) δ 11.30 (s, 1H), 7.83 – 7.73 (m, 3H), 7.66 – 7.62 (m, 1H), 7.50 – 7.37 (m, 2H), 7.32 (dd, *J* = 8.5, 1.8 Hz, 1H), 3.11 (t, *J* = 7.8 Hz, 2H), 2.89 – 2.68 (m, 2H). **^{13}C NMR** (101 MHz, $CDCl_3$) δ 179.3, 137.7, 133.6, 132.2, 128.3, 127.7, 127.6, 126.9, 126.5, 126.1, 125.5, 35.6, 30.7.

The structure was confirmed according to the precedents reported in the literature.[2]4

nonanoic acid (13)

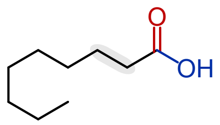

13

The title compound was prepared according to **general procedure A** using **L2** as ligand and purified by column chromatography (PE/EA = 20:1) as a colourless oil (100 mg, 79% yield, l/b > 20:1). **^{1}H NMR** (400 MHz, $CDCl_3$) δ 9.43 (s, 1H), 2.35 (t, *J* = 7.5 Hz, 2H), 1.64 (q, *J* = 7.4 Hz, 2H), 1.36 – 1.23 (m, 10H), 0.92 – 0.83 (m, 3H). **^{13}C NMR** (101 MHz, $CDCl_3$) δ 180.4, 34.1, 31.8, 29.2, 29.1, 29.1, 24.7, 22.7, 14.1. **HRMS** (ESI) calcd. for $C_9H_{19}O_2$ $[M+H]^+$: 159.1380, found: 159.1377

The structure was confirmed according to the precedents reported in the literature.[15]

3-cyclohexylpropanoic acid (14)

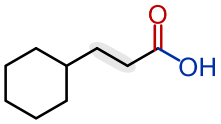

14

The title compound was prepared according to **general procedure A** using DPEphos as ligand and purified by column chromatography (PE/EA = 20:1) as a colourless oil (98 mg, 79% yield, l/b > 20:1). **^{1}H NMR** (400 MHz, $CDCl_3$) δ 11.71 (s, 1H), 2.35 – 2.21 (m, 2H), 1.71 – 1.53 (m, 5H), 1.46 (dt, *J* = 8.6, 7.0 Hz, 2H), 1.12 (dddt, *J* = 25.2, 15.3, 9.7, 3.4 Hz, 4H), 0.89 – 0.75 (m, 2H). **^{13}C NMR** (101 MHz, $CDCl_3$) δ 180.6, 37.1, 32.9, 32.1, 31.7, 26.5, 26.2.

The structure was confirmed according to the precedents reported in the literature.[24]

4,4-dimethylpentanoic acid (15)

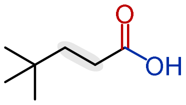

15

The title compound was prepared according to **general procedure A** using DPEphos as ligand and purified by column chromatography (PE/EA = 20:1) as a colourless oil (87 mg, 84% yield, l/b > 20:1). **^{1}H NMR** (400 MHz, $CDCl_3$) δ 11.74 (s, 1H), 2.36 – 2.10 (m, 2H), 1.66 – 1.36 (m, 2H), 0.83 (s, 9H). **^{13}C NMR** (101 MHz, $CDCl_3$) δ 181.1, 38.3, 29.9, 29.0.

The structure was confirmed according to the precedents reported in the literature.[15]

4-phenylbutanoic acid (16)

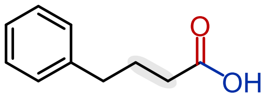

16

The title compound was prepared according to **general procedure A** using **L2** as ligand and purified by column chromatography (PE/EA = 5:1) as a colourless oil (112 mg, 85% yield, l/b > 20:1). **^{1}H NMR** (400 MHz, $CDCl_3$) δ 11.00 (s, 1H), 7.31 – 7.23 (m, 2H), 7.17 (m, 3H), 2.74 – 2.58 (m, 2H), 2.35 (t, *J* = 7.5 Hz, 2H), 1.95 (m, 2H). **^{13}C NMR** (101 MHz, $CDCl_3$) δ 180.0, 141.2, 128.5, 128.5, 126.1, 35.0, 33.4, 26.2.

The structure was confirmed according to the precedents reported in the literature.[15]

7-chloroheptanoic acid (17)

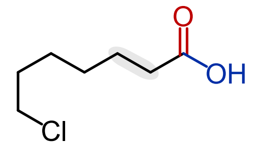

17

The title compound was prepared according to **general procedure A** using **L2** as ligand and purified by column chromatography (PE/EA = 5:1) as a colourless oil (110 mg, 84% yield, l/b > 20:1). **^{1}H NMR** (400 MHz, $CDCl_3$) δ 3.54 (t, J = 6.7 Hz, 2H), 2.37 (t, J = 7.4 Hz, 2H), 1.78 (dq, J = 10.9, 6.8 Hz, 2H), 1.66 (p, J = 7.4 Hz, 2H), 1.54 – 1.32 (m, 4H). **^{13}C NMR** (101 MHz, $CDCl_3$) δ 180.0, 45.0, 33.9, 32.3, 28.3, 26.5, 24.5.

The structure was confirmed according to the precedents reported in the literature.[25]

8-bromooctanoic acid (18)

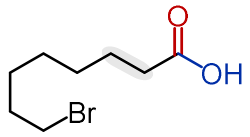

18

The title compound was prepared according to **general procedure A** using **L2** as ligand and purified by column chromatography (PE/EA = 5:1) as a colourless oil (161 mg, 81% yield, l/b > 20:1). **^{1}H NMR** (400 MHz, $CDCl_3$) δ 3.41 (t, J = 6.8 Hz, 2H), 2.36 (t, J = 7.5 Hz, 2H), 1.86 (dt, J = 14.4, 6.9 Hz, 2H), 1.70 – 1.60 (m, 2H), 1.49 – 1.40 (m, 2H), 1.39 – 1.30 (m, 4H). **^{13}C NMR** δ 180.3, 34.0, 33.9, 32.7, 28.8, 28.4, 28.0, 24.5.

The structure was confirmed according to the precedents reported in the literature.[16]

7-hydroxyheptanoic acid (19)

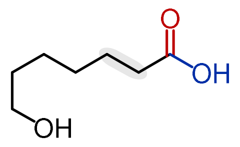

19

The title compound was prepared according to **general procedure A** using **L2** as ligand and purified by column chromatography (PE/EA = 2:1) as a colourless oil (105 mg, 90% yield, l/b > 20:1). **^{1}H NMR** (400 MHz, $CDCl_3$) δ 5.82 (s, 2H), 3.64 (t, J = 6.6 Hz, 2H), 2.35 (t, J = 7.5 Hz, 2H), 1.70 – 1.52 (m, 4H), 1.38 (p, J = 3.6 Hz, 4H). **^{13}C NMR** (101 MHz, $CDCl_3$) δ 179.1, 62.7, 34.0, 32.3, 28.8, 25.3, 24.6. **HRMS** (ESI) calcd. for $C_7H_{15}O_3$ $[M+H]^+$: 147.1016, found: 147.1018

6-(tosyloxy)hexanoic acid (20)

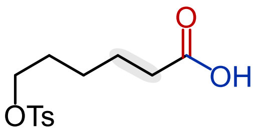

20

The title compound was prepared according to **general procedure A** using **L2** as ligand and purified by column chromatography (PE/EA = 4:1) as a white solid (149 mg, 65% yield, l/b > 20:1). **^{1}H NMR** (400 MHz, $CDCl_3$) δ 8.59 (s, 1H), 7.79 (d, J = 8.0 Hz, 2H), 7.35 (d, J = 8.0 Hz, 2H), 4.03 (t, J = 6.4 Hz, 2H), 2.45 (s, 3H), 2.31 (td, J = 7.4, 1.1 Hz, 2H), 1.74 – 1.52 (m, 4H), 1.48 – 1.31 (m, 2H). **^{13}C NMR** (101 MHz, $CDCl_3$) δ 179.6, 144.8, 133.0, 129.9, 127.9, 70.2, 33.7, 28.5, 24.9, 23.9, 21.7. **HRMS** (ESI) calcd. for $C_{13}H_{18}O_5SNa$ $[M+Na]^+$: 309.0767, found: 309.0773

6-(benzoyloxy)hexanoic acid (21)

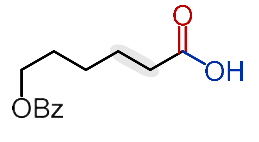

21

The title compound was prepared according to **general procedure A** using **L2** as ligand and purified by column chromatography (PE/EA = 4:1) as a white solid (163 mg, 86% yield, l/b = 20:1). **^{1}H NMR** (400 MHz, $CDCl_3$) δ 11.29 (s, 1H), 8.12 – 8.01 (m, 2H), 7.60 – 7.49 (m, 1H), 7.48 – 7.38 (m, 2H), 4.32 (t, *J* = 6.6 Hz, 2H), 2.39 (t, *J* = 7.4 Hz, 2H), 1.87 – 1.65 (m, 4H), 1.57 – 1.44 (m, 2H). **^{13}C NMR** (101 MHz, $CDCl_3$) δ 179.6, 166.7, 132.9, 130.3, 129.6, 128.4, 64.7, 33.9, 28.4, 25.6, 24.3. **HRMS** (ESI) calcd. for $C_{13}H_{16}O_4Na$ $[M+Na]^+$: 259.0941, found: 259.0945

6-((benzofuran-2-carbonyl)oxy)hexanoic acid (22)

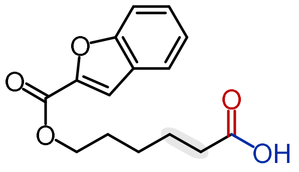

22

The title compound was prepared according to **general procedure A** using **L2** as ligand and purified by column chromatography (PE/EA = 4:1) as a white solid (158 mg, 72% yield, l/b > 20:1). **^{1}H NMR** (400 MHz, $CDCl_3$) δ 7.68 (d, *J* = 7.8 Hz, 1H), 7.60 (d, *J* = 8.4 Hz, 1H), 7.53 (s, 1H), 7.49 – 7.40 (m, 1H), 7.30 (dd, *J* = 15.0, 7.6 Hz, 1H), 4.39 (t, *J* = 6.6 Hz, 2H), 2.40 (t, *J* = 7.4 Hz, 2H), 1.92 – 1.66 (m, 4H), 1.64 – 1.44 (m, 2H). **^{13}C NMR** (101 MHz, $CDCl_3$) δ 179.7, 159.7, 155.7, 145.5, 127.6, 127.0, 123.8, 122.8, 113.9, 112.4, 65.2, 33.9, 28.4, 25.4, 24.3.**HRMS** (ESI) calcd. for $C_{15}H_{16}O_5Na$ $[M+Na]^+$: 299.0890, found: 299.0892

7-phenoxyheptanoic acid (23)

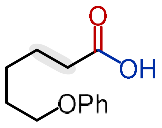

23

The title compound was prepared according to **general procedure A** using **L2** as ligand and purified by column chromatography (PE/EA = 5:1) as a white solid (149 mg, 84% yield, l/b > 20:1). **^{1}H NMR** (400 MHz, $CDCl_3$) δ 11.6 (s, 1H), 7.3 – 7.2 (m, 2H), 7.0 – 6.8 (m, 3H), 3.9 (t, *J* = 6.4 Hz, 2H), 2.4 (t, *J* = 7.5 Hz, 2H), 1.8 – 1.8 (m, 2H), 1.7 (dq, *J* = 15.3, 7.6, 6.9 Hz, 2H), 1.5 (dddd, *J* = 14.8, 9.4, 6.5, 3.3 Hz, 2H). **^{13}C NMR** δ 180.2, 159.0, 129.5, 120.6, 114.5, 67.5, 34.1, 29.0, 25.7, 24.5.
The structure was confirmed according to the precedents reported in the literature.[23]

6-oxoheptanoic acid (24)

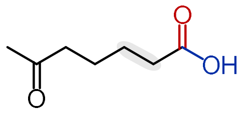

24

The title compound was prepared according to **general procedure A** using **L2** as ligand for 48 hours and purified by column chromatography (PE/EA = 2:1) as a colourless oil (95mg, 82% yield, l/b > 20:1). **^{1}H NMR** (400 MHz, $CDCl_3$) δ 8.59 (s, 1H), 2.49 (td, *J* = 6.7, 6.2, 3.6 Hz, 2H), 2.41 – 2.33 (m, 2H), 2.16 (s, 3H), 1.71 – 1.54 (m, *J* = 3.6 Hz, 4H). **^{13}C NMR** (101 MHz, $CDCl_3$) δ 209.2, 178.9, 43.2, 33.8, 29.9, 24.1, 23.0. **HRMS** (ESI) calcd. for $C_7H_{13}O_3$ $[M+H]^+$: 145.0859, found: 145.0864

6-oxo-6-phenylhexanoic acid (25)

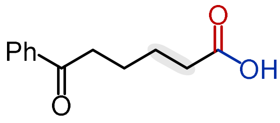

25

The title compound was prepared according to **general procedure A** using **L2** as ligand for 48 hours and purified by column chromatography (PE/EA = 3:1) as a white solid (130 mg, 79% yield, l/b > 20:1). **^{1}H NMR** (500 MHz, $CDCl_3$) δ 11.11 (s, 1H), 7.95 (dt, *J* = 8.5, 1.7 Hz, 2H), 7.55 (tt, *J* = 6.9, 1.4 Hz, 1H), 7.49 – 7.42 (m, 2H), 3.00 (dd, *J* = 8.2, 6.2 Hz, 2H), 2.42 (td, *J* = 7.2, 1.9 Hz, 2H), 1.85 – 1.68 (m, 4H). **^{13}C NMR** (101 MHz, $CDCl_3$) δ 199.9, 179.7, 136.9, 133.1, 128.6, 128.0, 38.1, 33.9, 24.3, 23.5. **HRMS** (ESI) calcd. for $C_{12}H_{14}O_3Na$ $[M+Na]^+$: 229.0835, found: 229.0841

dodecanedioic acid (26)

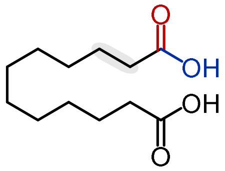

26

The title compound was prepared according to **general procedure A** and using **L2** as ligand purified by column chromatography (PE/EA = 1:1) as a white solid (138 mg, 75% yield, l/b > 20:1). **^{1}H NMR** (500 MHz, DMSO-d_6) δ 12.01 (s, 2H), 2.20 (t, *J* = 7.4 Hz, 4H), 1.50 (p, *J* = 7.0 Hz, 4H), 1.31 – 1.22 (m, 12H). **^{13}C NMR** (101 MHz, DMSO-d_6) δ 175.0, 34.1, 29.3, 29.2, 29.0, 25.0. **HRMS** (ESI) calcd. for $C_{12}H_{22}O_4Na$ $[M+Na]^+$: 253.1410, found: 253.1417

4-cyanobutanoic acid (27)

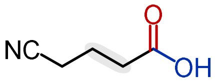

27

The title compound was prepared according to **general procedure A** using **L2** as ligand and purified by column chromatography (PE/EA = 3:1) as a colourless oil (79 mg, 87% yield, l/b > 20:1). **^{1}H NMR** (400 MHz, $CDCl_3$) δ 9.06 (s, 1H), 2.56 (t, *J* = 7.1 Hz, 2H), 2.49 (t, *J* = 7.1 Hz, 2H), 2.05 – 1.95 (m, 2H). **^{13}C NMR** (101 MHz, $CDCl_3$) δ 177.6, 118.9, 32.1, 20.4, 16.5.**HRMS** (ESI) calcd. for $C_5H_8NO_2$ $[M+H]^+$: 114.0550, found: 114.0545

5-(1,3-dioxoisoindolin-2-yl)pentanoic acid (28)

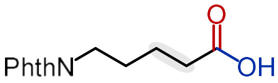

28

The title compound was prepared according to **general procedure A** using **L2** as ligand and purified by column chromatography (PE/EA = 2:1) as a white solid (83 mg, 84% yield, l/b = 12:1). **^{1}H NMR** (400 MHz, $CDCl_3$) δ 7.85 (dd, *J* = 5.4, 3.1 Hz, 2H), 7.72 (dd, *J* = 5.5, 3.0 Hz, 2H), 3.71 (t, *J* = 6.8 Hz, 2H), 2.41 (t, *J* = 7.1 Hz, 2H), 1.80 – 1.64 (m, 4H). **^{13}C NMR** (101 MHz, $CDCl_3$) δ 179.2, 168.5, 134.0, 132.0, 123.3, 37.4, 33.4, 27.9, 21.8. **HRMS** (ESI) calcd. for $C_{13}H_{13}NO_4Na$ $[M+Na]^+$: 270.0737, found: 270.0743

5-((*N*-benzyl-4-methylphenyl)sulfonamido)pentanoic acid (29)

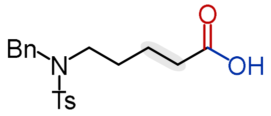

29

The title compound was prepared according to **general procedure A** using **L2** as ligand and purified by column chromatography (PE/EA = 3:1) as a white solid (255 mg, 88% yield, l/b > 20:1). **^{1}H NMR** (400 MHz, $CDCl_3$) δ 7.80 – 7.65 (m, 2H), 7.35 – 7.30 (m, 3H), 7.28 (d, J = 4.6 Hz, 4H), 4.29 (s, 2H), 3.09 (t, J = 7.2 Hz, 2H), 2.44 (s, 3H), 2.16 (t, J = 7.1 Hz, 2H), 1.54 – 1.27 (m, 4H). **^{13}C NMR** (101 MHz, $CDCl_3$) δ 179.0, 143.4, 136.8, 136.4, 129.8, 128.6, 128.3, 127.9, 127.2, 52.2, 47.7, 33.2, 27.4, 21.6, 21.5. **HRMS** (ESI) calcd. for $C_{19}H_{23}NO_4SNa$ $[M+Na]^+$: 384.1240, found: 384.1244

4-(1*H*-indol-1-yl)butanoic acid (30)

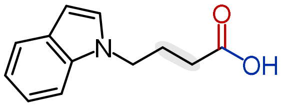

30

The title compound was prepared according to **general procedure A** using **L2** as ligand and purified by column chromatography (PE/EA = 5:1) as a white solid (122 mg, 75% yield, l/b > 20:1). **^{1}H NMR** (500 MHz, $CDCl_3$) δ 7.63 (dt, J = 7.9, 1.0 Hz, 1H), 7.34 (dd, J = 8.3, 1.0 Hz, 1H), 7.21 (ddd, J = 8.2, 7.0, 1.2 Hz, 1H), 7.10 (ddd, J = 8.0, 7.0, 1.0 Hz, 1H), 7.08 (d, J = 3.1 Hz, 1H), 6.50 (dd, J = 3.2, 0.9 Hz, 1H), 4.21 (t, J = 6.9 Hz, 2H), 2.34 (t, J = 7.2 Hz, 2H), 2.16 (p, J = 7.0 Hz, 2H). **^{13}C NMR** (126 MHz, $CDCl_3$) δ 178.7, 135.9, 128.7, 127.8, 121.6, 121.1, 119.5, 109.3, 101.5, 45.2, 30.9, 25.1. **HRMS** (ESI) calcd. for $C_{12}H_{13}NO_2Na$ $[M+Na]^+$: 226.0838, found: 226.0835

7-(1*H*-pyrrol-1-yl)heptanoic acid (31)

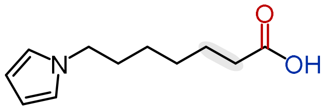

31

The title compound was prepared according to **general procedure A** using **L2** as ligand and purified by column chromatography (PE/EA = 5:1) as a white solid (105 mg, 73% yield, l/b > 20:1). **^{1}H NMR** (500 MHz, $CDCl_3$) δ 10.94 (s, 1H), 6.62 (t, J = 2.2 Hz, 2H), 6.11 (t, J = 2.2 Hz, 2H), 3.83 (t, J = 7.1 Hz, 2H), 2.31 (t, J = 7.5 Hz, 2H), 1.74 (p, J = 7.2 Hz, 2H), 1.60 (p, J = 7.4 Hz, 2H), 1.44 – 1.22 (m, 4H). **^{13}C NMR** (126 MHz, $CDCl_3$) δ 180.4, 120.5, 107.9, 49.5, 34.1, 31.4, 28.7, 26.5, 24.6. **HRMS** (ESI) calcd. for $C_{11}H_{18}NO_2$ $[M+H]^+$: 196.1332, found: 196.1326

6-(9*H*-carbazol-9-yl)hexanoic acid (32)

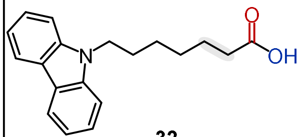

32

The title compound was prepared according to **general procedure A** using **L2** as ligand and purified by column chromatography (PE/EA = 5:1) as a white solid (182 mg, 77% yield, l/b > 20:1). **^{1}H NMR** (400 MHz, $CDCl_3$) δ 8.08 (dt, J = 7.8, 1.0 Hz, 2H), 7.44 (ddd, J = 8.3, 7.0, 1.2 Hz, 2H), 7.36 (d, J = 8.1 Hz, 2H), 7.25 – 7.17 (m, 2H), 4.24 (t, J = 7.1 Hz, 2H), 2.28 (t, J = 7.4 Hz, 2H), 1.89 – 1.77 (m, 2H), 1.56 (t, J = 7.2 Hz, 2H), 1.42 – 1.23 (m, 4H). **^{13}C NMR** (101 MHz, $CDCl_3$) δ 180.2, 140.4, 125.7, 122.9, 120.4, 118.8, 108.7, 42.9, 34.0, 28.8, 28.8, 27.0, 24.5.

The structure was confirmed according to the precedents reported in the literatur[21].

7-tosylheptanoic acid (33)

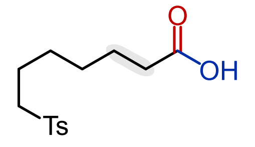

33

The title compound was prepared according to **general procedure A** using **L2** as ligand and purified by column chromatography (PE/EA = 3:1) as a white solid (192 mg, 85% yield, l/b > 20:1). **^{1}H NMR** (400 MHz, $CDCl_3$) δ 7.86 – 7.69 (m, 2H), 7.36 (d, *J* = 8.0 Hz, 2H), 3.12 – 2.93 (m, 2H), 2.45 (s, 3H), 2.32 (t, *J* = 7.4 Hz, 2H), 1.81 – 1.51 (m, 4H), 1.49 – 1.24 (m, 4H). **^{13}C NMR** (101 MHz, $CDCl_3$) δ 179.6, 144.7, 136.1, 129.9, 128.1, 56.2, 33.8, 28.4, 27.9, 24.2, 22.6, 21.6. **HRMS** (ESI) calcd. for $C_{14}H_{20}O_4SNa$ $[M+Na]^+$: 307.0975, found: 307.0984

4-(diethoxyphosphoryl)butanoic acid (34)

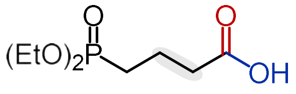

34

The title compound was prepared according to **general procedure A** using DPEphos as ligand and purified by column chromatography (PE/EA = 1:1) as a white solid (125 mg, 80% yield, l/b > 20:1). **^{1}H NMR** (400 MHz, $CDCl_3$) δ 8.39 (s, 1H), 4.25 – 3.93 (m, 4H), 2.44 (t, *J* = 6.9 Hz, 2H), 2.04 – 1.76 (m, 4H), 1.33 (t, *J* = 7.1 Hz, 6H). **^{13}C NMR** (101 MHz, $CDCl_3$) δ 61.9 (d, *J* = 6.5 Hz), 34.1 (d, *J* = 16.3 Hz), 24.6 (d, *J* = 141.5 Hz), 17.9 (d, *J* = 4.8 Hz), 16.4 (d, *J* = 6.1 Hz). **^{31}P NMR** (162 MHz, $CDCl_3$) δ 31.92. **HRMS** (ESI) calcd. for $C_8H_{17}O_5PNa$ $[M+Na]^+$: 247.0706, found: 247.0715

3-(cyclohex-3-en-1-yl)propanoic acid (35)

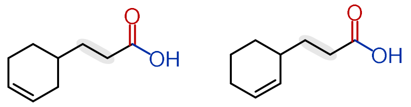

35 35'

35:35' = 1.9:1

The title compound was prepared according to **general procedure A** using DPEphos as ligand and purified by column chromatography (PE/EA = 20:1) as a colourless oil (71 mg, 58% yield, l/b > 20:1, a/b = 1.9:1). **^{1}H NMR** (400 MHz, $CDCl_3$) δ 11.70 (s, 1H), 5.78 – 5.60 (m, 1.6H, **35 + 35'**), 5.54 (dq, *J* = 10.1, 2.5 Hz, 0H, 35'), 2.45 – 2.34 (m, 2H), 2.16 – 2.01 (m, 2H), 1.97 (ddp, *J* = 10.2, 5.2, 2.5 Hz, 1H), 1.81 – 1.51 (m, 5H), 1.31 – 1.15 (m, 1H). **^{13}C NMR** (101 MHz, $CDCl_3$) δ 180.8, 180.8, 130.7, 127.8, 127.0, 126.1, 34.5, 33.0, 31.8, 31.6, 31.4, 31.2, 30.9, 28.6, 28.5, 25.3, 25.1, 21.3.

The structure was confirmed according to the precedents reported in the literature.[4, 14]

8-phenyloct-7-ynoic acid (36)

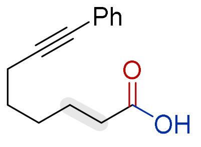

36

The title compound was prepared according to **general procedure A** using DPEphos as ligand and purified by column chromatography (PE/EA = 5:1) as a colourless oil (132 mg, 77% yield, l/b = 20:1). **^{1}H NMR** (400 MHz, $CDCl_3$) δ 11.67 (s, 1H), 7.42 – 7.37 (m, 2H), 7.31 – 7.24 (m, 3H), 2.40 (dt, *J* = 13.3, 7.2 Hz, 4H), 1.76 – 1.59 (m, 4H), 1.57 – 1.45 (m, 2H). **^{13}C NMR**

(101 MHz, $CDCl_3$) δ 180.4, 131.6, 128.2, 127.6, 124.0, 89.9, 80.9, 34.0, 28.4, 28.3, 24.2, 19.3. **HRMS** (ESI) calcd. for $C_{14}H_{15}O_2$ $[M-H]^-$: 215.1078, found: 215.1092

(*E*)-6-(but-2-enoyloxy)hexanoic acid (37)

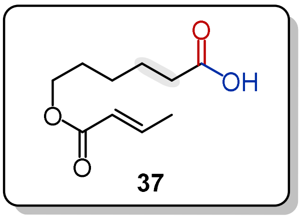

The title compound was prepared according to **general procedure A** using **L2** as ligand and purified by column chromatography (PE/EA = 5:1) as a colourless oil (131 mg, 82% yield, l/b > 20:1). **^{1}H NMR** (400 MHz, $CDCl_3$) δ 9.07 (s, 1H), 6.98 (dq, *J* = 15.6, 6.9 Hz, 1H), 5.85 (dq, *J* = 15.5, 1.8 Hz, 1H), 4.13 (t, *J* = 6.6 Hz, 2H), 2.37 (t, *J* = 7.5 Hz, 2H), 1.88 (dd, *J* = 6.9, 1.8 Hz, 3H), 1.68 (pd, *J* = 7.1, 2.7 Hz, 4H), 1.44 (ttd, *J* = 10.1, 7.6, 6.7, 3.4 Hz, 2H). **^{13}C NMR** (101 MHz, $CDCl_3$) δ 179.2, 166.7, 144.7, 122.7, 63.9, 33.8, 28.4, 25.5, 24.3, 18.0. **HRMS** (ESI) calcd. for $C_{10}H_{16}O_4Na$ $[M+Na]^+$: 223.0941, found: 223.0941

4,4,5,5,6,6,7,7,7-nonafluoroheptanoic acid (38)

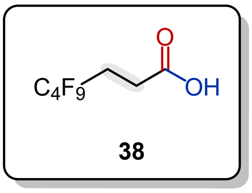

The title compound was prepared according to **general procedure A** using DPEphos as ligand for 48 hours and purified by column chromatography (PE/EA = 5:1) as a white solid (166 mg, 71% yield, l/b > 20:1). **^{1}H NMR** (400 MHz, $CDCl_3$) δ 11.90 (s, 1H), 2.70 (dd, *J* = 8.8, 6.8 Hz, 2H), 2.57 – 2.37 (m, 2H). **^{13}C NMR** (101 MHz, $CDCl_3$) δ 177.8, 122.2 – 104.4 (m), 26.0 (t, *J* = 22.2 Hz), 25.2 (t, *J* = 4.4 Hz). **^{19}F NMR** (376 MHz, $CDCl_3$) δ -81.5 (ddd, *J* = 9.2, 6.5, 3.2 Hz), -115.5 (ddt, *J* = 12.8, 7.6, 4.1 Hz), -124.8 (pq, *J* = 9.6, 6.2, 5.2 Hz), -126.4 (tt, *J* = 17.9, 5.7 Hz). **HRMS** (ESI) calcd. for $C_7H_6O_2F_9$ $[M+H]^+$: 293.0219, found: 293.0229

4-(dimethylamino)-4-oxobutanoic acid (39)

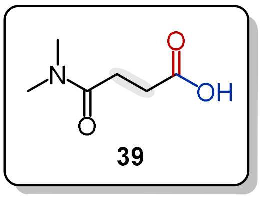

The title compound was prepared according to **general procedure A** using **L3** as ligand for 48 hours and purified by column chromatography (PE/EA = 1:1) as a white solid (102 mg, 88% yield, l/b > 20:1). **^{1}H NMR** (400 MHz, $CDCl_3$) δ 9.45 (s, 1H), 3.00 (s, 3H), 2.91 (s, 3H), 2.70 – 2.53 (m, 4H). **^{13}C NMR** (101 MHz, $CDCl_3$) δ 176.5, 172.1, 37.3, 35.7, 29.5, 28.1. **HRMS** (ESI) calcd. for $C_6H_{11}NO_3Na$ $[M+Na]^+$: 168.0631, found: 168.0638

***trans*-2-(4-phenylcyclohexyl)acetic acid (40)**

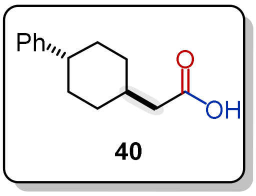

The title compound was prepared according to **general procedure A** using DPEphos as ligand and purified by column chromatography (PE/EA = 5:1) as a white solid (163 mg, 93% yield, l/b > 20:1, d.r. = 7:1.). **^{1}H NMR** (400 MHz, $CDCl_3$) δ 12.27 – 11.74 (m, 1H), 7.30 – 7.24 (m, 2H), 7.23 – 7.12 (m, 3H), 2.45 (tt, *J* = 12.2, 3.6 Hz, 1H), 2.28 (d, *J* = 6.9 Hz, 2H), 1.95 – 1.86 (m, 4H), 1.79 – 1.61 (m, 1H), 1.50 (qd, *J* = 13.3, 12.8, 3.5 Hz, 2H), 1.24 – 1.08 (m, 2H). **^{13}C NMR** (101 MHz, $CDCl_3$) δ 179.8, 147.3, 128.4, 126.9, 126.0, 44.1, 41.9, 34.4, 33.9, 33.2. **HRMS** (ESI)

calcd. for $C_{14}H_{19}O_2$ $[M+H]^+$: 219.1380, found: 219.1384

***trans*-2-(3-cyanocyclobutyl)acetic acid (41)**

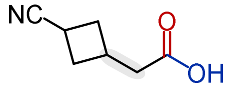

41

The title compound was prepared according to **general procedure A** using DPEphos as ligand and purified by column chromatography (PE/EA = 2:1) as a colourless oil (99 mg, 89% yield, l/b > 20:1, d.r. = 1.3:1). **^{1}H NMR** (500 MHz, $CDCl_3$) δ 11.19 (s, 1H), 3.13 (td, *J* = 9.3, 4.6 Hz, 0.44H, minor), 3.07 – 2.94 (m, 1H), 2.72 (dd, *J* = 15.8, 7.9 Hz, 0.57H, major), 2.66 – 2.45 (m, 4H), 2.15 (ddd, *J* = 31.2, 15.5, 8.3 Hz, 2H). **^{13}C NMR** (126 MHz, $CDCl_3$) δ 177.6, 177.5, 122.84, 121.82, 40.0, 39.2, 32.7, 31.3, 29.2, 29.0, 19.4, 18.4. **HRMS** (ESI) calcd. for $C_7H_{10}NO_2$ $[M+H]^+$: 140.0706, found: 140.0709.

3-ethylpentanoic acid (42)

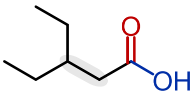

42

The title compound was prepared according to **general procedure A** using DPEphos as ligand for 48 hours and purified by column chromatography (PE/EA = 5:1) as a colourless oil (61 mg, 59% yield, l/b > 20:1). **^{1}H NMR** (400 MHz, $CDCl_3$) δ 11.30 (s, 1H), 2.28 (dd, *J* = 7.0, 0.8 Hz, 2H), 1.76 (dq, *J* = 13.0, 6.5 Hz, 1H), 1.46 – 1.30 (m, 4H), 0.88 (td, *J* = 7.4, 0.8 Hz, 6H). **^{13}C NMR** (101 MHz, $CDCl_3$) δ 180.6, 38.3, 37.7, 25.7, 10.8. **HRMS** (ESI) calcd. for $C_7H_{15}O_2$ $[M+H]^+$: 131.1067, found: 131.1073.

3,4-dimethylpentanoic acid (43)

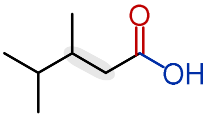

43

The title compound was prepared according to **general procedure A** using DPEphos as ligand for 48 hours and purified by column chromatography (PE/EA = 5:1) as a colourless oil (53 mg, 51% yield, l/b > 20:1). **^{1}H NMR** (400 MHz, $CDCl_3$) δ 11.68 (s, 1H), 2.40 (dd, *J* = 15.0, 5.2 Hz, 1H), 2.11 (dd, *J* = 14.9, 9.2 Hz, 1H), 1.89 (dddt, *J* = 11.2, 6.8, 4.5, 1.8 Hz, 1H), 1.62 (pd, *J* = 6.8, 4.8 Hz, 1H), 0.92 (d, *J* = 6.8 Hz, 3H), 0.89 (d, *J* = 6.9 Hz, 3H), 0.86 (d, *J* = 6.8 Hz, 3H). **^{13}C NMR** (101 MHz, $CDCl_3$) δ 180.7, 39.0, 35.7, 32.0, 19.8, 18.2, 15.8.

The structure was confirmed according to the precedents reported in the literature.[15]

3-(4-methylcyclohex-3-en-1-yl)butanoic acid (44)

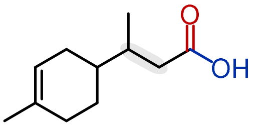

44

The title compound was prepared according to **general procedure A** using DPEphos as ligand for 48 hours and purified by column chromatography (PE/EA = 5:1) as a colourless oil (76 mg, 52% yield, l/b > 20:1, d.r. = 1:1). **^{1}H NMR** (400 MHz, $CDCl_3$) δ 11.27 (s, 1H), 5.36 (dt, *J* = 5.6, 1.9 Hz, 1H), 2.46 (dd, *J* = 15.0, 5.1 Hz, 1H), 2.14 (ddd, *J* = 15.1, 9.1, 3.9 Hz, 1H), 2.08 – 1.87 (m, 4H), 1.74 (ddddd, *J* = 22.4, 12.0, 9.6, 4.6, 2.5 Hz, 2H), 1.64 (d, *J* = 2.4 Hz, 3H), 1.44 (dtq, *J* = 13.1, 7.8, 2.4 Hz, 1H), 1.31 – 1.21 (m, 1H), 0.96 (dd, *J* = 6.9, 5.4

Hz, 3H). **^{13}C NMR** (101 MHz, $CDCl_3$) δ 180.55, 180.52, 134.04, 134.02, 120.53, 120.49, 39.2, 39.0, 38.3, 38.2, 34.5, 34.3, 30.7, 30.6, 29.1, 27.7, 26.8, 25.5, 23.4, 16.6, 16.3. **HRMS** (ESI) calcd. for $C_{11}H_{19}O_2$ $[M+H]^+$: 183.1380, found: 183.1378

cyclopentanecarboxylic acid (45)

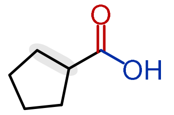

45

The title compound was prepared according to **general procedure A** using DPEphos as ligand for 48 hours and purified by column chromatography (PE/EA = 50:1) as a colourless oil (59 mg, 65% yield). **^{1}H NMR** (500 MHz, $CDCl_3$) δ 11.71 (s, 1H), 2.77 (p, J = 8.0 Hz, 1H), 1.94 (dt, J = 7.7, 3.5 Hz, 1H), 1.90 (dt, J = 7.6, 3.5 Hz, 1H), 1.83 (dq, J = 12.6, 7.7 Hz, 2H), 1.71 (tdd, J = 10.6, 7.7, 5.0 Hz, 2H), 1.60 (dtd, J = 11.8, 7.4, 3.2 Hz, 2H). **^{13}C NMR** (126 MHz, $CDCl_3$) δ 183.8, 43.7, 30.0, 25.9. **HRMS** (ESI) calcd. for $C_6H_{11}O_2$ $[M+H]^+$: 115.0754, found: 115.0755

cyclohexanecarboxylic acid (46)

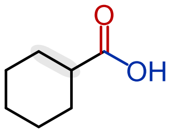

46

The title compound was prepared according to **general procedure A** using DPEphos as ligand for 48 hours and purified by column chromatography (PE/EA = 50:1) as a colourless oil (77 mg, 77% yield). **^{1}H NMR** (400 MHz, $CDCl_3$) δ 11.84 (s, 1H), 2.33 (tt, J = 11.2, 3.7 Hz, 1H), 2.01 – 1.88 (m, 2H), 1.76 (dp, J = 13.1, 3.2, 2.6 Hz, 2H), 1.68 – 1.59 (m, 1H), 1.45 (qd, J = 11.6, 3.4 Hz, 2H), 1.36 – 1.16 (m, 3H). **^{13}C NMR** (101 MHz, $CDCl_3$) δ 183.0, 43.0, 28.8, 25.7, 25.3. **HRMS** (ESI) calcd. for $C_7H_{13}O_2$ $[M+H]^+$: 129.0910, found: 129.0914

bicyclo[2.2.1]heptane-2-carboxylic acid (47)

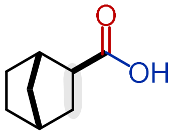

47

The title compound was prepared according to **general procedure A** using DPEphos as ligand for 48 hours and purified by column chromatography (PE/EA = 50:1) as a colourless oil (86 mg, 77% yield, d.r. > 20:1). **^{1}H NMR** (400 MHz, $CDCl_3$) δ 12.22 – 11.75 (m, 1H), 2.54 (d, J = 3.9 Hz, 1H), 2.35 (dd, J = 9.3, 5.3 Hz, 1H), 2.29 (t, J = 4.3 Hz, 1H), 1.84 (dtd, J = 12.4, 4.8, 2.7 Hz, 1H), 1.62 – 1.41 (m, 4H), 1.29 – 1.22 (m, 1H), 1.22 – 1.11 (m, 2H). **^{13}C NMR** (101 MHz, $CDCl_3$) δ 183.0, 46.5, 41.0, 36.6, 36.1, 34.1, 29.5, 28.7. **HRMS** (ESI) calcd. for $C_8H_{13}O_2$ $[M+H]^+$: 141.0910, found: 141.0912

The structure was confirmed according to the precedents reported in the literature.[2]

nonanoic acid (48)

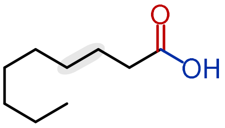

48

The title compound was prepared according to **general procedure A** using **L2** as ligand for 48 hours and purified by column chromatography (PE/EA = 20:1) as a colourless oil (93 mg, 74% yield, l/b = 5:1). **^{1}H NMR** (400 MHz, $CDCl_3$) δ 11.70 (s, 1H), 2.27 (t, J = 7.5 Hz, 2H), 1.56 (q, J = 7.5 Hz, 2H), 1.27 – 1.16 (m, 10H), 0.82 – 0.77 (m, 3H). **^{13}C NMR** (101 MHz, $CDCl_3$) δ 180.8, 34.2, 31.8, 29.2, 29.12, 29.07, 24.7, 22.7, 14.1, 14.1. **HRMS** (ESI) calcd. for $C_9H_{19}O_2$ $[M+H]^+$:

159.1380, found: 159.1385

6-phenylhexanoic acid (49)

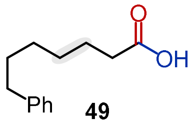

49

The title compound was prepared according to **general procedure A** using **L2** as ligand for 48 hours and purified by column chromatography (PE/EA = 10:1) as a colourless oil (133 mg, 76% yield, l/b = 7:1). **^{1}H NMR** (400 MHz, $CDCl_3$) δ 11.90 (s, 1H), 7.25 (dd, *J* = 8.2, 6.9 Hz, 2H), 7.17 – 7.13 (m, 3H), 2.59 (d, *J* = 7.5 Hz, 2H), 2.32 (t, *J* = 7.5 Hz, 2H), 1.60 (tq, *J* = 10.9, 3.9, 3.4 Hz, 4H), 1.39 – 1.31 (m, 4H). **^{13}C NMR** (101 MHz, $CDCl_3$) δ 180.8, 142.7, 128.5, 128.4, 125.7, 36.0, 34.2, 31.4, 29.0, 24.7.
The structure was confirmed according to the precedents reported in the literature.[14]

6-((2-(4-(2,2-dichlorocyclopropyl)phenoxy)-2-methylpropanoyl)oxy)hexanoic acid (50)

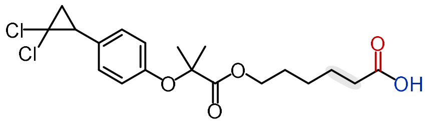

50

The title compound was prepared according to **general procedure A** using DPEphos as ligand and purified by column chromatography (PE/EA = 3:1) as a white solid (286 mg, 89% yield, l/b > 20:1). **^{1}H NMR** (400 MHz, $CDCl_3$) δ 11.21 (s, 1H), 7.17 – 7.06 (m, 2H), 6.84 – 6.75 (m, 2H), 4.15 (t, *J* = 6.5 Hz, 2H), 2.82 (dd, *J* = 10.7, 8.3 Hz, 1H), 2.28 (t, *J* = 7.5 Hz, 2H), 1.93 (dd, *J* = 10.8, 7.5 Hz, 1H), 1.77 (dd, *J* = 8.4, 7.4 Hz, 1H), 1.67 – 1.53 (m, 10H), 1.33 – 1.21 (m, 2H). **^{13}C NMR** (101 MHz, $CDCl_3$) δ 179.90, 174.28, 154.99, 129.65, 128.01, 118.39, 118.32, 79.11, 65.16, 60.94, 34.78, 33.83, 28.07, 25.79, 25.44, 25.24, 24.10. ^{13}C NMR (101 MHz, $CDCl_3$) δ 179.9, 174.3, 155.0, 129.7, 128.0, 118.4, 118.3, 79.1, 65.2, 60.9, 34.8, 33.8, 28.1, 25.8, 25.4, 25.2, 24.1. **HRMS** (ESI) calcd. for $C_{19}H_{24}Cl_2O_5Na$ $[M+Na]^+$: 425.0893, found: 425.0898

(*E*)-7-((3,7-dimethylocta-2,6-dien-1-yl)oxy)-7-oxoheptanoic acid (51)

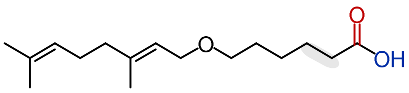

51

The title compound was prepared according to **general procedure A** using DPEphos as ligand and purified by column chromatography (PE/EA = 5:1) as a white solid (126 mg, 56% yield, l/b > 20:1). **^{1}H NMR** (400 MHz, $CDCl_3$) δ 10.83 (s, 1H), 5.34 (tq, *J* = 6.8, 1.4 Hz, 1H), 5.09 (tdd, *J* = 6.9, 3.0, 1.5 Hz, 1H), 3.98 (d, *J* = 6.8 Hz, 2H), 3.42 (t, *J* = 6.6 Hz, 2H), 2.36 (t, *J* = 7.5 Hz, 2H), 2.16 – 1.99 (m, 4H), 1.72 – 1.55 (m, 13H), 1.48 – 1.35 (m, 2H). **^{13}C NMR** (101 MHz, $CDCl_3$) δ 179.7, 140.0, 131.5, 124.0, 120.9, 69.8, 67.2, 39.6, 34.0, 29.4, 26.3, 25.74, 25.68, 24.5, 17.7, 16.4. **HRMS** (ESI) calcd. for $C_{16}H_{28}O_3Na$ $[M+Na]^+$: 291.1931, found: 291.1943

(*S*)-3-((2*R*,8*R*,8a*S*)-8,8a-dimethyl-6-oxo-1,2,3,4,6,7,8,8a-octahydronaphthalen-2-yl)butanoic acid (52)

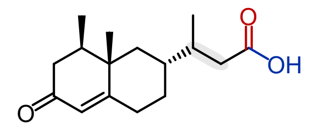

52

The title compound was prepared according to **general procedure A** using DPEphos as ligand for 48 hours and purified by column chromatography (PE/EA = 5:1) as a white solid (133 mg, 63% yield, l/b > 20:1, d.r. = 1:1). **^{1}H NMR** (400 MHz, $CDCl_3$) δ 11.32 (s, 1H), 5.79 (d, *J* = 1.7 Hz, 1H), 2.53 – 2.33 (m, 3H), 2.31 – 2.12 (m, 3H), 2.08 – 1.79 (m, 4H), 1.79 – 1.68 (m, 1H), 1.20 (ddt, *J* = 16.2, 12.8, 6.0 Hz, 1H), 1.08 (d, *J* = 2.2 Hz, 3H), 1.03 – 0.87 (m, 7H). **^{13}C NMR** (101 MHz, $CDCl_3$) δ 200.4, 178.60, 178.55, 171.7, 124.40, 124.37, 42.6, 41.8, 41.0, 40.42, 40.39, 39.3, 39.21, 39.19, 38.8, 37.2, 37.1, 34.5, 33.0, 32.9, 30.0, 28.5, 16.90, 16.89, 16.6, 16.2, 14.92, 14.90. **HRMS** (ESI) calcd. for $C_{16}H_{24}O_3Na$ $[M+Na]^+$: 287.1618, found: 287.1620

7-((11-oxo-6,11-dihydrodibenzo[b,e]oxepine-2-carbonyl)oxy)heptanoic acid (53)

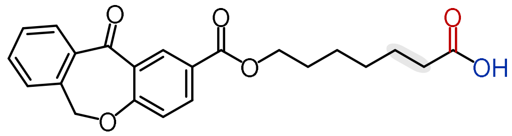

53

The title compound was prepared according to **general procedure A** using DPEphos as ligand and purified by column chromatography (PE/EA = 5:1) as a white solid (245 mg, 80% yield, l/b > 20:1). **^{1}H NMR** (400 MHz, $CDCl_3$) δ 8.12 (dd, *J* = 7.3, 2.4 Hz, 1H), 7.89 (dd, *J* = 7.6, 1.4 Hz, 1H), 7.56 (td, *J* = 7.5, 1.4 Hz, 1H), 7.48 (dd, *J* = 7.6, 1.3 Hz, 1H), 7.42 (dd, *J* = 8.5, 2.5 Hz, 1H), 7.36 (dd, *J* = 7.5, 1.3 Hz, 1H), 7.03 (dd, *J* = 8.4, 4.5 Hz, 1H), 5.18 (s, 2H), 4.10 (t, *J* = 6.5 Hz, 2H), 3.64 (s, 2H), 2.35 (t, *J* = 7.4 Hz, 2H), 1.64 (ddt, *J* = 10.7, 7.5, 3.4 Hz, 4H), 1.46 – 1.33 (m, 2H). **^{13}C NMR** (101 MHz, $CDCl_3$) δ 191.1, 179.3, 171.6, 160.5, 140.4, 136.4, 135.6, 132.8, 132.4, 129.5, 129.3, 127.9, 127.8, 125.1, 121.1, 73.6, 64.8, 40.3, 33.8, 28.2, 25.4, 24.2. **HRMS** (ESI) calcd. for $C_{22}H_{22}O_6Na$ $[M+Na]^+$: 405.1309, found: 405.1313.

8-hydroxy-4,8-dimethylnonanoic acid (54)

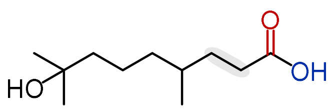

54

The title compound was prepared according to **general procedure A** using DPEphos as ligand and purified by column chromatography (PE/EA = 2:1) as a white solid (114 mg, 71% yield, l/b > 20:1). **^{1}H NMR** (400 MHz, $CDCl_3$) δ 6.65 (s, 2H), 2.43 – 2.24 (m, 2H), 1.76 – 1.62 (m, 1H), 1.53 – 1.40 (m, 4H), 1.33 (dddd, *J* = 22.7, 12.8, 8.6, 3.6 Hz, 3H), 1.21 (s, 6H), 1.18 – 1.08 (m, 1H), 0.89 (d, *J* = 6.3 Hz, 3H). **^{13}C NMR** (101 MHz, $CDCl_3$) δ 178.7, 71.5, 43.8, 37.1, 32.2, 31.9, 31.6, 28.9, 28.8, 21.5, 19.2. **HRMS** (ESI) calcd. for $C_{11}H_{22}O_3Na$ $[M+Na]^+$: 225.1461, found: 225.1456.

13-(((8*R*,9*S*,10*R*,13*S*,14*S*,17*S*)-10,13-dimethyl-3-oxo-6,7,8,9,10,11,12,13,14,15,16,17-dodecahydro-3*H*-cyclopenta[a]phenanthren-17-yl)oxy)-13-oxotridecanoic acid (55)

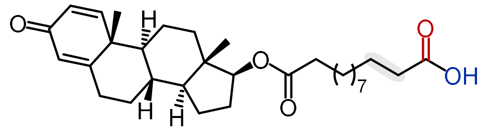

55

The title compound was prepared according to **general procedure A** using **L2** as ligand and purified by column chromatography (PE/EA = 2:1) as a white solid (288 mg, 72% yield, l/b > 20:1). **^{1}H NMR** (400 MHz, $CDCl_3$) δ 7.06 (d, *J* = 10.2 Hz, 1H), 6.25 (dd, *J* = 10.1, 1.9 Hz, 1H), 6.09 (t, *J* = 1.7 Hz, 1H), 4.59 (dd, *J* = 9.2, 7.7 Hz, 1H), 2.52 – 2.43 (m, 1H), 2.41 – 2.26 (m, 5H), 2.23 – 2.13 (m, 1H), 1.97 (dtd, *J* = 15.8, 5.9, 5.4, 2.5 Hz, 1H), 1.83 – 1.69 (m, 3H), 1.68 – 1.57 (m, 6H), 1.54 – 1.48 (m, 1H), 1.44 – 1.38 (m, 1H), 1.35 – 1.27 (m, 12H), 1.24 (s, 3H), 1.19 (dd, *J* = 3.6, 2.2 Hz, 1H), 1.05 (dddd, *J* = 15.4, 12.7, 8.5, 5.2 Hz, 3H), 0.87 (s, 3H). **^{13}C NMR** (101 MHz, $CDCl_3$) δ 186.5, 179.3, 173.9, 169.2, 156.0, 127.5, 123.9, 82.0, 52.2, 49.9, 43.6, 42.8, 36.5, 35.3, 34.5, 34.0, 33.1, 32.8, 29.7, 29.4, 29.3, 29.2, 29.10, 29.05, 27.5, 25.1, 24.7, 23.7, 22.4, 18.7, 12.2. **HRMS** (ESI) calcd. for $C_{31}H_{46}O_5Na$ $[M+Na]^+$: 521.3237, found: 521.3253.

6-oxo-6-(((1*R*,2*S*,4*R*)-1,7,7-trimethylbicyclo[2.2.1]heptan-2-yl)oxy)hexanoic acid (56)

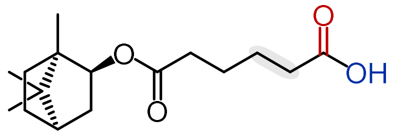

56

The title compound was prepared according to **general procedure A** using DPEphos as ligand and purified by column chromatography (PE/EA = 4:1) as a white solid (183 mg, 81% yield, l/b > 20:1). **^{1}H NMR** (400 MHz, $CDCl_3$) δ 10.98 (s, 1H), 4.89 (ddd, *J* = 10.0, 3.5, 2.0 Hz, 1H), 2.45 – 2.29 (m, 5H), 1.92 (ddd, *J* = 12.3, 9.2, 4.5 Hz, 1H), 1.80 – 1.73 (m, 1H), 1.69 (dq, *J* = 9.0, 4.7, 4.1 Hz, 5H), 1.35 – 1.18 (m, 2H), 0.95 (dd, *J* = 13.8, 3.4 Hz, 1H), 0.90 (s, 3H), 0.87 (s, 3H), 0.83 (s, 3H). **^{13}C NMR** (101 MHz, $CDCl_3$) δ 179.3, 173.7, 79.9, 48.7, 47.7, 44.8, 36.8, 34.2, 33.6, 28.0, 27.1, 24.4, 24.1, 19.7, 18.8, 13.5. **HRMS** (ESI) calcd. for $C_{16}H_{26}O_4Na$ $[M+Na]^+$: 305.1723, found: 305.1731.

2-((8*S*,9*S*,13*R*,14*S*,17*R*)-3-methoxy-13-methyl-7,8,9,11,12,13,14,15,16,17-decahydro-6*H*-cyclopenta[a]phenanthren-17-yl)acetic acid (57)

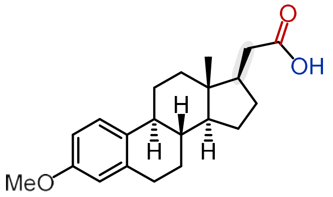

57

The title compound was prepared according to **general procedure A** using DPEphos as ligand and purified by column chromatography (PE/EA = 5:1) as a white solid (201 mg, 77% yield, l/b > 20:1 d.r. = 10:1). **^{1}H NMR** (500 MHz, $CDCl_3$) δ 11.58 (s, 1H), 7.16 (dd, *J* = 8.8, 2.5 Hz, 1H), 6.67 (dq, *J* = 8.2, 2.6 Hz, 1H), 6.60 (t, *J* = 3.0 Hz, 1H), 3.80 – 3.70 (m, 3H), 2.91 – 2.76 (m, 2H), 2.43 (dt, *J* = 15.1, 4.0 Hz, 1H), 2.31 – 2.23 (m, 1H), 2.17 (td, *J* = 12.3, 11.2, 6.1 Hz, 2H), 2.00 (dtd, *J* = 13.7, 9.7, 8.8, 4.3 Hz, 1H), 1.87 (tq, *J* = 10.1, 5.3, 4.6 Hz, 2H), 1.80 (dd, *J* = 12.4, 3.3 Hz, 1H), 1.77 – 1.69 (m, 1H), 1.49 – 1.18 (m, 7H), 0.76 – 0.46 (m,

3H). **^{13}C NMR** (126 MHz, $CDCl_3$) δ 180.8, 157.5, 138.1, 132.8, 126.4, 113.9, 111.5, 55.2, 54.4, 46.9, 44.1, 42.6, 39.0, 37.4, 35.3, 30.0, 28.4, 27.9, 26.5, 24.4, 12.7. **HRMS** (ESI) calcd. for $C_{21}H_{28}O_3Na$ $[M+Na]^+$: 351.1931, found: 351.1938.

methyl 6-(1,3-dioxoisoindolin-2-yl)hexanoate (58)

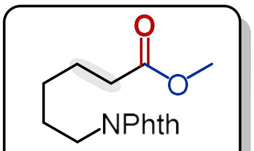

58

The title compound was prepared according to **general procedure B** using **L2** as ligand and purified by column chromatography (PE/EA = 20:1) as a white solid (86 mg, 78% yield, l/b > 20:1). **^{1}H NMR** (400 MHz, $CDCl_3$) δ 7.90 – 7.77 (m, 2H), 7.79 – 7.69 (m, 2H), 3.71 – 3.67 (m, 2H), 3.66 (s, 3H), 2.32 (t, J = 7.5 Hz, 2H), 1.80 – 1.61 (m, 4H), 1.48 – 1.32 (m, 2H). **^{13}C NMR** (101 MHz, $CDCl_3$) δ 174.0, 168.5, 133.9, 132.1, 123.2, 51.5, 37.8, 33.9, 28.3, 26.4, 24.5.
HRMS (ESI) calcd. for $C_{15}H_{17}NO_4Na$ $[M+Na]^+$: 298.1050, found: 298.1052.

ethyl 6-(1,3-dioxoisoindolin-2-yl)hexanoate (59)

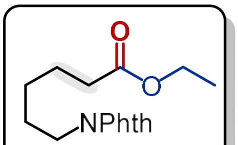

59

The title compound was prepared according to **general procedure B** using **L2** as ligand and purified by column chromatography (PE/EA = 20:1) as a white solid (97 mg, 84% yield, l/b > 20:1). **^{1}H NMR** (400 MHz, $CDCl_3$) δ 7.75 (dd, J = 5.4, 3.1 Hz, 2H), 7.63 (dd, J = 5.5, 3.0 Hz, 2H), 4.03 (q, J = 7.2 Hz, 2H), 3.60 (t, J = 7.3 Hz, 2H), 2.21 (t, J = 7.5 Hz, 2H), 1.61 (dp, J = 10.7, 7.5 Hz, 4H), 1.36 – 1.24 (m, 2H), 1.16 (t, J = 7.1 Hz, 3H). **^{13}C NMR** (101 MHz, $CDCl_3$) δ 173.5, 168.3, 133.9, 132.1, 123.1, 60.2, 37.7, 34.1, 28.3, 26.3, 24.5, 14.2. **HRMS** (ESI) calcd. for $C_{16}H_{19}NO_4Na$ $[M+Na]^+$: 312.1206, found: 312.1213.

isopropyl 6-(1,3-dioxoisoindolin-2-yl)hexanoate (60)

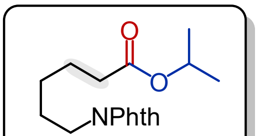

60

The title compound was prepared according to **general procedure B** using **L2** as ligand and purified by column chromatography (PE/EA = 20:1) as a white solid (92 mg, 76% yield, l/b = 16:1). **^{1}H NMR** (400 MHz, $CDCl_3$) δ 7.84 (dd, J = 5.4, 3.1 Hz, 2H), 7.72 (dd, J = 5.5, 3.0 Hz, 2H), 5.00 (h, J = 6.3 Hz, 1H), 3.75 – 3.62 (m, 2H), 2.27 (t, J = 7.5 Hz, 2H), 1.77 – 1.60 (m, 4H), 1.46 – 1.33 (m, 2H), 1.22 (d, J = 6.3 Hz, 6H). **^{13}C NMR** (101 MHz, $CDCl_3$) δ 173.0, 168.4, 133.9, 132.1, 123.1, 67.4, 37.8, 34.4, 28.3, 26.3, 24.5, 21.8. **HRMS** (ESI) calcd. for $C_{17}H_{21}NO_4Na$ $[M+Na]^+$: 326.1363, found: 326.1362.

tert-butyl 6-(1,3-dioxoisoindolin-2-yl)hexanoate (61)

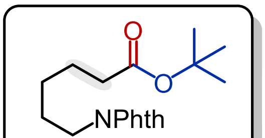

61

The title compound was prepared according to **general procedure B** using **L2** as ligand and purified by column chromatography (PE/EA = 20:1) as a white solid (91 mg, 72% yield, l/b > 20:1). **^{1}H NMR** δ 7.84 (dd, J = 5.4, 3.1 Hz, 2H), 7.72 (dd, J = 5.4, 3.0 Hz, 2H), 3.69 (t, J = 7.3 Hz, 2H), 2.21 (t, J = 7.5 Hz, 2H), 1.70 (p, J = 7.6 Hz, 2H), 1.67 – 1.59 (m, 2H), 1.43 (s, 9H), 1.37 (tdd, J

= 10.1, 7.9, 4.4 Hz, 2H). **^{13}C NMR** (126 MHz, $CDCl_3$) δ 173.0, 168.4, 133.9, 132.1, 123.2, 80.1, 37.8, 35.3, 28.3, 28.1, 26.3, 24.6. **HRMS** (ESI) calcd. for $C_{18}H_{23}NO_4Na$ $[M+Na]^+$: 340.1520, found: 340.1526.

cyclohexyl 6-(1,3-dioxoisoindolin-2-yl)hexanoate (62)

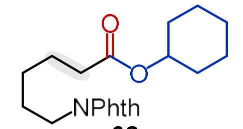

62

The title compound was prepared according to **general procedure B** using **L2** as ligand and purified by column chromatography (PE/EA = 20:1) as a white solid (96 mg, 70% yield, l/b > 20:1). **^{1}H NMR** (400 MHz, $CDCl_3$) δ 7.91 – 7.81 (m, 2H), 7.76 – 7.67 (m, 2H), 4.87 – 4.60 (m, 1H), 3.69 (t, J = 7.3 Hz, 2H), 2.28 (t, J = 7.4 Hz, 2H), 1.86 – 1.78 (m, 2H), 1.76 – 1.62 (m, 6H), 1.57 – 1.49 (m, 1H), 1.44 – 1.19 (m, 7H). **^{13}C NMR** (101 MHz, $CDCl_3$) δ 172.9, 168.4, 133.9, 132.1, 123.2, 123.1, 72.4, 37.8, 34.5, 31.6, 28.3, 26.3, 25.4, 24.6, 23.7. **HRMS** (ESI) calcd. for $C_{20}H_{25}NO_4Na$ $[M+Na]^+$: 366.1676, found: 366.1677.

benzyl 6-(1,3-dioxoisoindolin-2-yl)hexanoate (63)

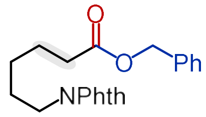

63

The title compound was prepared according to **general procedure B** using **L2** as ligand and purified by column chromatography (PE/EA = 20:1) as a white solid (146 mg, 81% yield, l/b > 20:1). **^{1}H NMR** (400 MHz, $CDCl_3$) δ 7.87 – 7.77 (m, 2H), 7.75 – 7.64 (m, 2H), 7.40 – 7.26 (m, 5H), 5.10 (s, 2H), 3.66 (t, J = 7.3 Hz, 2H), 2.36 (t, J = 7.5 Hz, 2H), 1.69 (pd, J = 7.5, 3.2 Hz, 4H), 1.44 – 1.31 (m, 2H). **^{13}C NMR** (101 MHz, $CDCl_3$) δ 173.2, 168.3, 136.1, 133.9, 132.1, 128.5, 128.2, 123.1, 66.1, 37.7, 34.1, 28.3, 26.3, 24.5. **HRMS** (ESI) calcd. for $C_{21}H_{22}NO_4$ $[M+H]^+$: 352.1543, found: 352.1543.

2-hydroxyethyl 6-(1,3-dioxoisoindolin-2-yl)hexanoate (64)

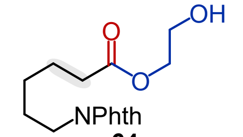

64

The title compound was prepared according to **general procedure B** using **L2** as ligand and purified by column chromatography (PE/EA = 2:1) as a white solid (105 mg, 86% yield, l/b > 20:1). **^{1}H NMR** (400 MHz, $CDCl_3$) δ 7.85 (dd, J = 5.5, 3.1 Hz, 2H), 7.77 – 7.64 (m, 2H), 4.29 – 4.14 (m, 2H), 3.88 – 3.74 (m, 2H), 3.69 (t, J = 7.2 Hz, 2H), 2.36 (t, J = 7.4 Hz, 2H), 2.24 (d, J = 33.7 Hz, 1H), 1.70 (pd, J = 7.3, 1.5 Hz, 4H), 1.47 – 1.32 (m, 2H). **^{13}C NMR** (101 MHz, $CDCl_3$) δ 173.9, 168.5, 134.0, 132.1, 123.2, 66.0, 61.2, 37.7, 34.0, 28.2, 26.2, 24.4. **HRMS** (ESI) calcd. for $C_{16}H_{19}NO_5Na$ $[M+Na]^+$: 328.1155, found: 328.1159.

2,2,2-trifluoroethyl 6-(1,3-dioxoisoindolin-2-yl)hexanoate (65)

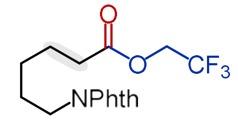

65

The title compound was prepared according to **general procedure B** using **L2** as ligand and purified by column chromatography (PE/EA = 20:1) as a white solid (96 mg, 70% yield, l/b > 20:1). **^{1}H NMR** (400 MHz, $CDCl_3$) δ 7.84 (dt, J = 7.0, 3.5 Hz, 2H), 7.77 – 7.67 (m, 2H), 4.46 (q, J = 8.5 Hz, 2H), 3.69 (t, J = 7.2 Hz, 2H), 2.43 (t, J = 7.5 Hz, 2H), 1.78 – 1.64 (m, 4H), 1.47 – 1.34 (m,

2H). **^{13}C NMR** (101 MHz, $CDCl_3$) δ 171.8, 168.4, 133.9, 132.1, 123.2, 123.0 (q, J = 277.2 Hz), 60.2 (q, J = 36.6 Hz), 37.7, 33.4, 28.2, 26.2, 24.2. **^{19}F NMR** (376 MHz, $CDCl_3$) δ -73.8. **HRMS** (ESI) calcd. for $C_{16}H_{16}F_3NO_4Na$ $[M+Na]^+$: 366.0924, found: 366.0928.

phenyl 6-(1,3-dioxoisoindolin-2-yl)hexanoate (66)

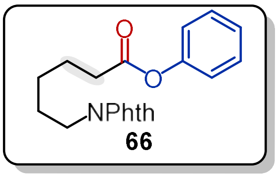

The title compound was prepared according to **general procedure B** using DPEphos as ligand and purified by column chromatography (PE/EA = 10:1) as a white solid (76 mg, 56% yield, l/b > 20:1). **^{1}H NMR** (500 MHz, $CDCl_3$) δ 7.87 – 7.81 (m, 2H), 7.74 – 7.67 (m, 2H), 7.36 (t, J = 7.9 Hz, 2H), 7.21 (t, J = 7.4 Hz, 1H), 7.09 – 7.02 (m, 2H), 3.72 (t, J = 7.2 Hz, 2H), 2.57 (t, J = 7.5 Hz, 2H), 1.86 – 1.71 (m, 4H), 1.55 – 1.40 (m, 2H). **^{13}C NMR** (126 MHz, $CDCl_3$) δ 172.0, 168.5, 150.7, 134.0, 132.1, 129.4, 125.8, 123.3, 123.2, 121.6, 37.8, 34.2, 28.3, 26.3, 24.5. **HRMS** (ESI) calcd. for $C_{20}H_{19}NO_4Na$ $[M+Na]^+$: 360.1206, found: 360.1204.

4-bromophenyl 6-(1,3-dioxoisoindolin-2-yl)hexanoate (67)

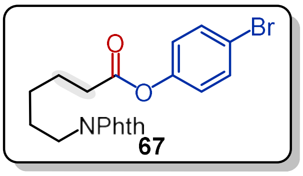

The title compound was prepared according to **general procedure B** using DPEphos as ligand and purified by column chromatography (PE/EA = 10:1) as a white solid (90 mg, 54% yield, l/b > 20:1). **^{1}H NMR** (400 MHz, $CDCl_3$) δ 7.89 – 7.80 (m, 2H), 7.76 – 7.67 (m, 2H), 7.50 – 7.42 (m, 2H), 7.00 – 6.91 (m, 2H), 3.71 (t, J = 7.2 Hz, 2H), 2.55 (t, J = 7.4 Hz, 2H), 1.77 (ddt, J = 17.2, 15.0, 7.5 Hz, 4H), 1.52 – 1.40 (m, 2H). **^{13}C NMR** (101 MHz, $CDCl_3$) δ 171.7, 168.5, 149.7, 134.0, 132.4, 132.1, 123.4, 123.2, 118.8, 37.7, 34.1, 28.3, 26.3, 24.4. **HRMS** (ESI) calcd. for $C_{20}H_{18}NO_4BrNa$ $[M+Na]^+$: 438.0311, found: 438.0315.

4-methoxyphenyl 6-(1,3-dioxoisoindolin-2-yl)hexanoate (68)

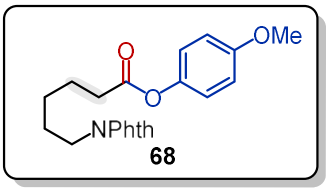

The title compound was prepared according to **general procedure B** using DPEphos as ligand and purified by column chromatography (PE/EA = 5:1) as a white solid (97 mg, 67% yield, l/b > 20:1). **^{1}H NMR** (400 MHz, $CDCl_3$) δ 7.87 – 7.79 (m, 2H), 7.74 – 7.65 (m, 2H), 7.01 – 6.93 (m, 2H), 6.92 – 6.81 (m, 2H), 3.77 (s, 3H), 3.71 (t, J = 7.2 Hz, 2H), 2.54 (t, J = 7.5 Hz, 2H), 1.77 (dddd, J = 22.6, 15.0, 8.3, 3.9 Hz, 4H), 1.53 – 1.40 (m, 2H). **^{13}C NMR** (101 MHz, $CDCl_3$) δ 172.4, 168.4, 157.2, 144.2, 133.9, 132.1, 123.2, 122.3, 114.4, 55.6, 37.8, 34.1, 28.3, 26.3, 24.5. **HRMS** (ESI) calcd. for $C_{21}H_{21}NO_5Na$ $[M+Na]^+$: 390.1312, found: 390.1322.

1,3-dioxoisoindolin-2-yl 6-(1,3-dioxoisoindolin-2-yl)hexanoate (69)

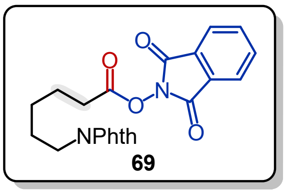

The title compound was prepared according to **general procedure B** using DPEphos as ligand and purified by column chromatography (PE/EA = 1:1) as a white solid (88 mg, 54% yield, l/b > 20:1). **^{1}H NMR** (400 MHz, $CDCl_3$) δ 7.92 – 7.76

(m, 6H), 7.74 – 7.67 (m, 2H), 3.73 (t, *J* = 7.2 Hz, 2H), 2.68 (t, *J* = 7.4 Hz, 2H), 1.81 (m, 4H), 1.60 – 1.46 (m, 2H).**^{13}C NMR** (101 MHz, $CDCl_3$) δ 169.4, 168.4, 161.9, 134.8, 133.9, 132.1, 128.9, 123.9, 123.2, 37.6, 30.8, 28.1, 26.0, 24.2. **HRMS** (ESI) calcd. for $C_{22}H_{18}N_2O_6Na$ $[M+Na]^+$: 429.1057, found: 429.1068.

(1*R*,2*S*,5*R*)-2-isopropyl-5-methylcyclohexyl 6-(1,3-dioxoisoindolin-2-yl)hexanoate (70)

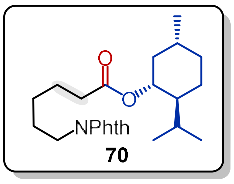

The title compound was prepared according to **general procedure C** and purified by column chromatography (PE/EA = 20:1) as a white solid (105 mg, 64% yield, l/b > 20:1). **^{1}H NMR** (400 MHz, $CDCl_3$) δ 7.81 (dtt, *J* = 8.5, 5.5, 3.1 Hz, 2H), 7.69 (ddq, *J* = 7.6, 5.0, 2.3 Hz, 2H), 4.63 (tt, *J* = 11.0, 5.6 Hz, 1H), 3.66 (p, *J* = 7.1 Hz, 2H), 2.26 (dt, *J* = 11.2, 7.2 Hz, 2H), 1.92 (d, *J* = 11.6 Hz, 1H), 1.86 – 1.77 (m, 1H), 1.73 – 1.57 (m, 6H), 1.44 (s, 1H), 1.35 (dq, *J* = 14.9, 7.2, 5.7 Hz, 3H), 1.01 (dt, *J* = 12.1, 6.4 Hz, 1H), 0.96 – 0.80 (m, 8H), 0.76 – 0.67 (m, 3H). **^{13}C NMR** (101 MHz, $CDCl_3$) δ 168.4, 133.9, 132.1, 123.2, 73.9, 47.0, 40.9, 37.8, 34.5, 34.2, 31.4, 28.3, 26.4, 26.2, 24.7, 23.4, 22.0, 20.8, 16.3. **HRMS** (ESI) calcd. for $C_{24}H_{33}NO_4Na$ $[M+Na]^+$: 422.2302, found: 422.2304

(1*S*,2*R*,4*S*)-1,7,7-trimethylbicyclo[2.2.1]heptan-2-yl 6-(1,3-dioxoisoindolin-2-yl)hexanoate (71)

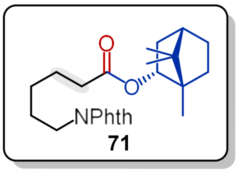

The title compound was prepared according to **general procedure C** and purified by column chromatography (PE/EA = 20:1) as a white solid (101 mg, 61% yield, l/b > 20:1). **^{1}H NMR** (400 MHz, $CDCl_3$) δ 7.90 – 7.79 (m, 2H), 7.76 – 7.66 (m, 2H), 4.87 (ddq, *J* = 9.6, 4.1, 2.0 Hz, 1H), 3.69 (tt, *J* = 7.3, 1.9 Hz, 2H), 2.33 (tt, *J* = 7.5, 1.9 Hz, 3H), 1.97 – 1.85 (m, 1H), 1.79 – 1.61 (m, 6H), 1.46 – 1.35 (m, 2H), 1.33 – 1.17 (m, 2H), 0.98 – 0.92 (m, 1H), 0.89 (t, *J* = 2.0 Hz, 3H), 0.86 (t, *J* = 2.0 Hz, 3H), 0.80 (t, *J* = 1.9 Hz, 3H). **^{13}C NMR** (101 MHz, $CDCl_3$) δ 173.8, 168.4, 133.9, 132.1, 123.2, 79.6, 48.7, 47.8, 44.9, 37.8, 36.8, 34.5, 28.3, 28.0, 27.1, 26.4, 24.7, 19.7, 18.8, 13.5. **HRMS** (ESI) calcd. for $C_{24}H_{31}NO_4Na$ $[M+Na]^+$: 420.2145, found: 420.2150

(*S*)-2-((tert-butoxycarbonyl)amino)-3-phenylpropyl 6-(1,3-dioxoisoindolin-2-yl)hexanoate (72)

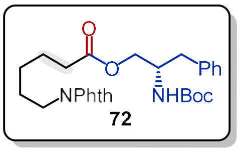

The title compound was prepared according to **general procedure C** and purified by column chromatography (PE/EA = 10:1) as a white solid (128 mg, 63% yield, l/b > 20:1). **^{1}H NMR** (400 MHz, $CDCl_3$) δ 7.83 (dt, *J* = 5.6, 2.8 Hz, 2H), 7.70 (dt, *J* = 5.5, 2.7 Hz, 2H), 7.33 – 7.26 (m, 2H), 7.25 – 7.12 (m, 3H), 4.78 (d, *J* = 8.8 Hz, 1H), 4.08 (d, *J* = 12.4 Hz, 1H), 4.05 – 3.97 (m, 2H), 3.69 (td, *J* = 7.2, 2.2 Hz, 2H), 2.93 – 2.73 (m, 2H), 2.35 (td, *J* = 7.5, 2.1 Hz, 2H), 1.71 (tt, *J* = 8.1, 6.1 Hz, 4H), 1.49 – 1.32 (m, 11H). **^{13}C NMR** (101 MHz, $CDCl_3$) δ 173.3, 168.4, 155.2, 137.3, 133.9, 132.1, 129.3, 128.6, 126.6, 123.2, 79.5, 64.9, 50.7, 37.9, 37.7, 33.9, 28.35, 28.26, 26.3, 24.4. **HRMS** (ESI) calcd. for $C_{28}H_{34}N_2O_6Na$ $[M+Na]^+$: 517.2309, found: 517.2316.

(3*S*,5*S*,8*R*,9*S*,10*S*,13*S*,14*S*)-10,13-dimethyl-17-oxohexadecahydro-1*H*-cyclopenta[a]phenanthren-3-yl 6-(1,3-dioxoisoindolin-2-yl) hexanoate (73)

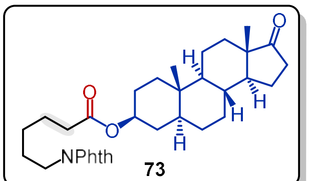

73

The title compound was prepared according to **general procedure C** and purified by column chromatography (PE/EA = 5:1) as a white solid (141 mg, 66% yield, l/b > 20:1). **^{1}H NMR** (500 MHz, $CDCl_3$) δ 7.90 – 7.79 (m, 2H), 7.79 – 7.68 (m, 2H), 4.68 (tt, *J* = 11.4, 4.9 Hz, 1H), 3.68 (t, *J* = 7.2 Hz, 2H), 2.51 – 2.38 (m, 1H), 2.27 (t, *J* = 7.4 Hz, 2H), 2.17 – 2.01 (m, 1H), 2.01 – 1.91 (m, 1H), 1.83 – 1.76 (m, 3H), 1.76 – 1.63 (m, 6H), 1.62 – 1.45 (m, 4H), 1.42 – 1.12 (m, 9H), 1.10 – 0.95 (m, 2H), 0.85 (d, *J* = 6.6 Hz, 6H), 0.77 – 0.67 (m, 1H). **^{13}C NMR** (126 MHz, $CDCl_3$) δ 172.9, 168.3, 133.9, 132.1, 123.1, 73.2, 54.2, 51.3, 47.7, 44.6, 37.7, 36.6, 35.8, 35.6, 34.9, 34.4, 33.9, 31.5, 30.7, 28.24, 28.21, 27.4, 26.3, 24.5, 21.7, 20.4, 13.8, 12.2. **HRMS** (ESI) calcd. for $C_{33}H_{43}NO_5Na$ $[M+Na]^+$: 556.3033, found: 556.3035

(8*S*,9*R*,10*S*,13*R*,14*R*,17*R*)-10,13-dimethyl-3-oxo-2,3,6,7,8,9,10,11,12,13,14,15,16,17-tetradecahydro-1*H*-cyclopenta[a]phenanthren-17-yl 6-(1,3-dioxoisoindolin-2-yl)hexanoate (74)

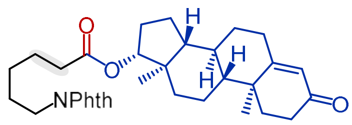

74

The title compound was prepared according to **general procedure C** and purified by column chromatography (PE/EA = 10:1) as a white solid (130 mg, 58% yield, l/b > 20:1). **^{1}H NMR** (500 MHz, $CDCl_3$) δ 7.90 – 7.79 (m, 2H), 7.78 – 7.67 (m, 2H), 5.73 (s, 1H), 4.59 (dd, *J* = 9.2, 7.8 Hz, 1H), 3.69 (t, *J* = 7.3 Hz, 2H), 2.49 – 2.25 (m, 6H), 2.22 – 2.10 (m, 1H), 2.06 – 1.99 (m, 1H), 1.88 – 1.82 (m, 1H), 1.79 – 1.62 (m, 7H), 1.61 – 1.52 (m, 2H), 1.52 – 1.43 (m, 1H), 1.44 – 1.28 (m, 4H), 1.19 (s, 3H), 1.18 – 1.13 (m, 1H), 1.10 – 0.98 (m, 2H), 0.94 (ddd, *J* = 12.2, 10.6, 4.1 Hz, 1H), 0.82 (s, 3H). **^{13}C NMR** (126 MHz, $CDCl_3$) δ 199.6, 173.5, 171.1, 168.4, 133.9, 132.1, 123.9, 123.2, 82.3, 53.7, 50.2, 42.5, 38.6, 37.8, 36.6, 35.7, 35.4, 34.3, 33.9, 32.7, 31.5, 28.3, 27.5, 26.4, 24.6, 23.5, 20.5, 17.4, 12.1. **HRMS** (ESI) calcd. for $C_{33}H_{41}NO_5Na$ $[M+Na]^+$: 554.2877, found: 554.2883

(3*S*,8*S*,9*S*,10*R*,13*R*,14*S*,17*R*)-10,13-dimethyl-17-((*R*)-6-methylheptan-2-yl)-2,3,4,7,8,9,10,11,12,13,14,15,16,17-tetradecahydro-1*H*-cyclopenta[a]phenanthren-3-yl 6-(1,3-dioxoisoindolin-2-yl)hexanoate (75)

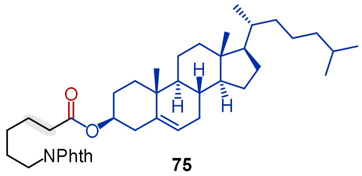

75

The title compound was prepared according to **general procedure C** and purified by column chromatography (PE/EA = 10:1) as a white solid (170 mg, 67% yield, l/b > 20:1). **^{1}H NMR** (500 MHz, $CDCl_3$) δ 7.87 – 7.78 (m, 2H), 7.77 – 7.65 (m, 2H), 5.39 – 5.31 (m, 1H), 4.59 (dtt, *J* = 11.1, 7.5, 4.2 Hz, 1H), 3.68 (t, *J* = 7.2 Hz, 2H), 2.28 (t, *J* = 7.4 Hz, 4H), 2.06 – 1.92 (m, 2H), 1.89 – 1.79 (m, 3H), 1.69 (dt, *J* = 15.6, 7.9 Hz, 4H), 1.60 – 1.32 (m, 12H), 1.31 – 1.21 (m, 2H), 1.13 (tdd, *J* = 18.4, 11.4, 6.5 Hz, 6H), 1.04 – 0.97 (m, 5H), 0.96 – 0.90 (m, 4H), 0.86 (dd, *J* = 6.6, 2.3 Hz, 6H), 0.67 (s, 3H).

^{13}C NMR (126 MHz, $CDCl_3$) δ 172.9, 168.3, 139.6, 133.9, 132.1, 123.2, 122.6, 73.7, 56.7, 56.1, 50.0, 42.3, 39.7, 39.5, 38.1, 37.8, 37.0, 36.6, 36.2, 35.8, 34.4, 31.9, 31.8, 28.3, 28.2, 28.0, 27.8, 26.3, 24.6, 24.3, 23.8, 22.9, 22.6, 21.0, 19.3, 18.7, 11.9. **HRMS** (ESI) calcd. for $C_{41}H_{59}NO_4Na$ $[M+Na]^+$: 652.4336, found: 652.4345

(*S*)-3,7-dimethyloct-6-en-1-yl 6-(1,3-dioxoisoindolin-2-yl)hexanoate (76)

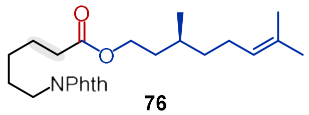

76

The title compound was prepared according to **general procedure C** and purified by column chromatography (PE/EA = 20:1) as a white solid (96 mg, 60% yield, l/b > 20:1). **^{1}H NMR** (500 MHz, $CDCl_3$) δ 7.96 – 7.78 (m, 2H), 7.77 – 7.67 (m, 2H), 5.08 (dddd, *J* = 8.5, 7.1, 2.9, 1.5 Hz, 1H), 4.16 – 3.99 (m, 2H), 3.69 (t, *J* = 7.3 Hz, 2H), 2.30 (t, *J* = 7.5 Hz, 2H), 2.07 – 1.90 (m, 2H), 1.75 – 1.63 (m, 8H), 1.60 (d, *J* = 1.4 Hz, 3H), 1.56 – 1.50 (m, 1H), 1.47 – 1.29 (m, 4H), 1.24 – 1.10 (m, 1H), 0.91 (d, *J* = 6.6 Hz, 3H). **^{13}C NMR** (126 MHz, $CDCl_3$) δ 173.6, 168.4, 133.9, 132.1, 131.3, 124.6, 123.18, 123.15, 62.8, 37.7, 36.9, 35.4, 34.1, 29.4, 28.3, 26.4, 25.7, 25.4, 24.5, 19.4, 17.7. **HRMS** (ESI) calcd. for $C_{24}H_{33}NO_4Na$ $[M+Na]^+$: 422.2302, found: 422.2306

6-(1,3-dioxoisoindolin-2-yl)-*N*-phenylhexanamide (77)

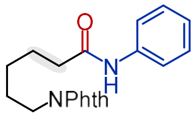

77

The title compound was prepared according to **general procedure D** using DPEphos as ligand and purified by column chromatography (PE/EA = 4:1) as a white solid (125 mg, 93% yield, l/b > 20:1). **^{1}H NMR** (400 MHz, $CDCl_3$) δ 7.81 (dt, *J* = 7.3, 3.7 Hz, 2H), 7.70 (td, *J* = 5.2, 2.0 Hz, 2H), 7.63 (d, *J* = 18.1 Hz, 1H), 7.56 – 7.45 (m, 2H), 7.28 (t, *J* = 7.8 Hz, 2H), 7.07 (t, *J* = 7.4 Hz, 1H), 3.68 (t, *J* = 7.2 Hz, 2H), 2.35 (t, *J* = 7.5 Hz, 2H), 1.88 – 1.62 (m, 4H), 1.41 (h, *J* = 7.4, 6.5 Hz, 2H). **^{13}C NMR** (101 MHz, $CDCl_3$) δ 171.3, 168.5, 138.1, 134.0, 132.1, 128.9, 124.1, 123.2, 119.9, 37.7, 37.4, 28.2, 26.3, 25.0. **HRMS** (ESI) calcd. for $C_{20}H_{20}N_2O_3Na$ $[M+Na]^+$: 359.1366, found: 359.1367

***N*-(4-chlorophenyl)-6-(1,3-dioxoisoindolin-2-yl)hexanamide (78)**

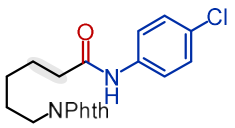

78

The title compound was prepared according to **general procedure D** using DPEphos as ligand and purified by column chromatography (PE/EA = 3:1) as a white solid (128 mg, 86% yield, l/b > 20:1). **^{1}H NMR** (400 MHz, $CDCl_3$) δ 7.86 – 7.77 (m, 2H), 7.75 – 7.66 (m, 2H), 7.59 (s, 1H), 7.52 – 7.44 (m, 2H), 7.29 – 7.20 (m, 2H), 3.69 (t, *J* = 7.2 Hz, 2H), 2.35 (t, *J* = 7.5 Hz, 2H), 1.84 – 1.66 (m, 4H), 1.41 (tt, *J* = 9.8, 6.3 Hz, 2H). **^{13}C NMR** (101 MHz, $CDCl_3$) δ 171.2, 168.6, 136.6, 134.0, 132.0, 129.0, 128.9, 123.2, 121.1, 37.6, 37.3, 28.2, 26.2, 24.8. **HRMS** (ESI) calcd. for $C_{20}H_{19}ClN_2O_3Na$ $[M+Na]^+$: 393.0976, found: 393.0985

N-(4-bromophenyl)-6-(1,3-dioxoisoindolin-2-yl)hexanamide (79)

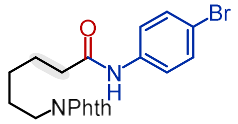

79

The title compound was prepared according to **general procedure D** using DPEphos as ligand and purified by column chromatography (PE/EA = 3:1) as a white solid (144 mg, 87% yield, l/b > 20:1). **^{1}H NMR** (500 MHz, $CDCl_3$) δ 7.80 (dt, J = 7.7, 3.9 Hz, 2H), 7.75 – 7.68 (m, 3H), 7.43 (d, J = 8.5 Hz, 2H), 7.38 (d, J = 8.5 Hz, 2H), 3.68 (t, J = 7.2 Hz, 2H), 2.34 (t, J = 7.5 Hz, 2H), 1.76 (p, J = 7.7 Hz, 2H), 1.73 – 1.67 (m, 2H), 1.39 (p, J = 7.9 Hz, 2H). **^{13}C NMR** (101 MHz, $CDCl_3$) δ 171.2, 168.6, 137.1, 134.0, 132.0, 131.9, 123.2, 121.4, 116.6, 37.6, 37.4, 28.2, 26.2, 24.8. **HRMS** (ESI) calcd. for $C_{20}H_{19}BrN_2O_3Na$ $[M+Na]^+$: 437.0471, found: 437.0483

N-(4-cyanophenyl)-6-(1,3-dioxoisoindolin-2-yl)hexanamide (80)

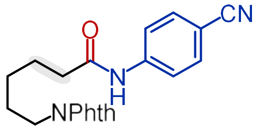

80

The title compound was prepared according to **general procedure D** using DPEphos as ligand and purified by column chromatography (PE/EA = 2:1) as a white solid (129 mg, 89% yield, l/b > 20:1). **^{1}H NMR** (400 MHz, $CDCl_3$) δ 7.97 (s, 1H), 7.88 – 7.78 (m, 2H), 7.78 – 7.66 (m, 4H), 7.63 – 7.55 (m, 2H), 3.69 (t, J = 7.2 Hz, 2H), 2.41 (t, J = 7.5 Hz, 2H), 1.76 (dp, J = 29.1, 7.5 Hz, 4H), 1.48 – 1.36 (m, 2H). **^{13}C NMR** (101 MHz, $CDCl_3$) δ 171.6, 168.6, 142.2, 134.1, 133.3, 132.0, 123.2, 119.5, 119.0, 106.8, 53.5, 37.5, 28.1, 26.1, 24.6. **HRMS** (ESI) calcd. for $C_{21}H_{19}N_3O_3Na$ $[M+Na]^+$: 384.1319, found: 384.1327

6-(1,3-dioxoisoindolin-2-yl)-N-(4-(trifluoromethyl)phenyl)hexanamide (81)

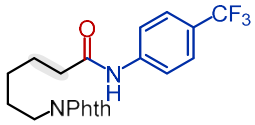

81

The title compound was prepared according to **general procedure D** using DPEphos as ligand and purified by column chromatography (PE/EA = 3:1) as a white solid (172 mg, 94% yield, l/b > 20:1). **^{1}H NMR** (400 MHz, $CDCl_3$) δ 7.80 (dt, J = 7.3, 3.7 Hz, 2H), 7.74 – 7.60 (m, 5H), 7.55 (d, J = 8.4 Hz, 2H), 3.70 (t, J = 7.1 Hz, 2H), 2.39 (t, J = 7.5 Hz, 2H), 1.81 (q, J = 7.6 Hz, 2H), 1.74 (dd, J = 14.4, 7.1 Hz, 2H), 1.43 (tt, J = 9.1, 6.2 Hz, 2H). **^{13}C NMR** (101 MHz, $CDCl_3$) δ 171.3, 168.6, 141.0, 136.3, 134.0, 132.0, 126.2 (q, J = 2.6 Hz), 124.1 (q, J = 260.4 Hz), 123.2, 119.3, 37.5, 37.4, 28.1, 26.1, 24.7. **^{19}F NMR** (376 MHz, $CDCl_3$) δ -62.1. **HRMS** (ESI) calcd. for $C_{21}H_{19}F_3N_2O_3Na$ $[M+Na]^+$: 427.1240, found: 427.1246

6-(1,3-dioxoisoindolin-2-yl)-N-(4-methoxyphenyl)hexanamide (82)

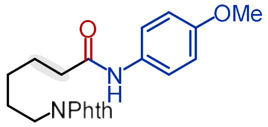

82

The title compound was prepared according to **general procedure D** using DPEphos as ligand and purified by column chromatography (PE/EA = 3:1) as a white solid (126 mg, 86% yield, l/b > 20:1). **^{1}H NMR** (400 MHz, $CDCl_3$) δ 7.81 (dd, J = 5.5, 3.1 Hz, 2H), 7.70 (dd, J = 5.5, 3.1 Hz, 2H), 7.54 (s, 1H), 7.44 – 7.37 (m, 2H), 6.92 – 6.75 (m, 2H), 3.77 (s, 3H), 3.68 (t, J = 7.2 Hz, 2H), 2.32 (t, J = 7.5 Hz, 2H), 1.74 (dq, J = 22.7, 7.6 Hz, 4H), 1.41 (tt, J = 9.8, 6.4 Hz, 2H). **^{13}C NMR** (101 MHz, $CDCl_3$) δ 171.1, 168.5, 156.3, 133.9, 132.1, 131.2, 123.2, 121.8, 114.0, 55.5, 37.7, 37.2, 28.3, 26.3, 25.1. **HRMS** (ESI) calcd. for $C_{21}H_{22}N_2O_4Na$

[M+Na]$^+$: 389.1472, found: 389.1486

6-(1,3-dioxoisoindolin-2-yl)-*N*-(naphthalen-1-yl)hexanamide (83)

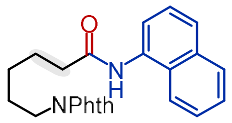

83

The title compound was prepared according to **general procedure D** using DPEphos as ligand and purified by column chromatography (PE/EA = 3:1) as a white solid (125 mg, 81% yield, l/b > 20:1). **^{1}H NMR** (500 MHz, DMSO-d$_6$) δ 9.87 (s, 1H), 8.07 – 8.02 (m, 1H), 7.98 – 7.90 (m, 1H), 7.90 – 7.85 (m, 2H), 7.85 – 7.80 (m, 2H), 7.75 (d, *J* = 8.2 Hz, 1H), 7.64 (d, *J* = 7.5 Hz, 1H), 7.58 – 7.50 (m, 2H), 7.47 (t, *J* = 7.8 Hz, 1H), 3.62 (t, *J* = 7.0 Hz, 2H), 2.49 (q, *J* = 7.4 Hz, 2H), 1.77 – 1.60 (m, 4H), 1.40 (h, *J* = 7.5, 6.4 Hz, 2H). **^{13}C NMR** (126 MHz, DMSO-d$_6$) δ 172.3, 168.4, 134.8, 134.1, 134.1, 132.0, 128.5, 128.3, 126.4, 126.2, 126.0, 125.5, 123.5, 123.2, 122.2, 37.9, 36.2, 28.3, 26.5, 25.4. **HRMS** (ESI) calcd. for $C_{24}H_{22}N_2O_3Na$ [M+Na]$^+$: 409.1523, found: 409.1532

6-(1,3-dioxoisoindolin-2-yl)-*N*-ethyl-*N*-phenylhexanamide (84)

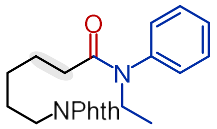

84

The title compound was prepared according to **general procedure D** using DPEphos as ligand and purified by column chromatography (PE/EA = 5:1) as a white solid (132 mg, 91% yield, l/b > 20:1). **^{1}H NMR** (400 MHz, CDCl$_3$) δ 7.82 (m, 2H), 7.71 (m, 2H), 7.41 (m, 2H), 7.37 – 7.29 (m, 1H), 7.14 (m, 2H), 3.77 – 3.67 (m, 2H), 3.62 (t, *J* = 7.2 Hz, 2H), 2.00 (t, *J* = 7.5 Hz, 2H), 1.59 (m, 4H), 1.32 – 1.17 (m, 2H), 1.09 (tt, *J* = 7.2, 1.3 Hz, 3H). **^{13}C NMR** (101 MHz, CDCl$_3$) δ 172.3, 168.4, 142.5, 133.9, 132.1, 129.7, 128.5, 127.8, 123.1, 43.9, 37.9, 34.2, 28.4, 26.6, 25.1, 13.1. **HRMS** (ESI) calcd. for $C_{22}H_{24}N_2O_3Na$ [M+Na]$^+$: 387.1679, found: 387.1685

2-(6-(3,4-dihydroquinolin-1(2*H*)-yl)-6-oxohexyl)isoindoline-1,3-dione (85)

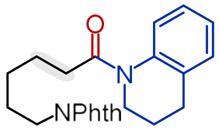

85

The title compound was prepared according to **general procedure D** using DPEphos as ligand and purified by column chromatography (PE/EA = 5:1) as a white solid (141 mg, 94% yield, l/b > 20:1). **^{1}H NMR** (400 MHz, CDCl$_3$) δ 7.88 – 7.77 (m, 2H), 7.71 (m, 2H), 7.14 (m, 4H), 3.77 (t, *J* = 6.6 Hz, 2H), 3.65 (t, *J* = 7.2 Hz, 2H), 2.71 (t, *J* = 6.7 Hz, 2H), 2.49 (t, *J* = 7.5 Hz, 2H), 1.95 (p, *J* = 6.7 Hz, 2H), 1.69 (m, 4H), 1.32 (m, 2H). **^{13}C NMR** (101 MHz, CDCl$_3$) δ 172.7, 168.4, 133.9, 132.1, 128.5, 126.1, 125.2, 124.7, 123.2, 37.9, 34.3, 28.4, 26.8, 26.6, 25.5, 24.2. **HRMS** (ESI) calcd. for $C_{23}H_{24}N_2O_3Na$ [M+Na]$^+$: 399.1679, found: 399.1684

***N*-benzyl-6-(1,3-dioxoisoindolin-2-yl)hexanamide (86)**

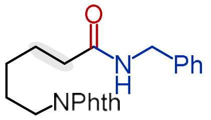

86

The title compound was prepared according to **general procedure D** using DPEphos as ligand and purified by column chromatography (PE/EA = 1:1) as a white solid (76 mg, 54% yield, l/b > 20:1). **^{1}H NMR** (500 MHz, CDCl$_3$) δ 7.87 – 7.76 (m, 2H), 7.74 – 7.66 (m, 2H), 7.31 – 7.26 (m, 2H), 7.26 – 7.22 (m, 3H), 6.11 (t, *J* = 5.8 Hz, 1H), 4.39 (d, *J* = 5.7 Hz, 2H), 3.64 (t, *J* = 7.2 Hz, 2H), 2.20 (t,

J = 7.5 Hz, 2H), 1.77 – 1.61 (m, 4H), 1.46 – 1.30 (m, 2H). **^{13}C NMR** (126 MHz, $CDCl_3$) δ 172.7, 168.4, 138.4, 133.9, 132.1, 128.6, 127.7, 127.4, 123.2, 43.5, 37.7, 36.4, 28.3, 26.5, 25.2. **HRMS** (ESI) calcd. for $C_{21}H_{22}N_2O_3Na$ $[M+Na]^+$: 373.1523, found: 373.1524

***N'*-(6-(1,3-dioxoisoindolin-2-yl)hexanoyl)-4-methylbenzenesulfonohydrazide (87)**

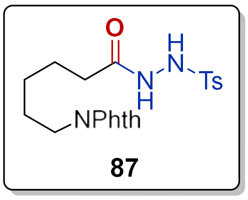

The title compound was prepared according to **general procedure D** using DPEphos as ligand and purified by column chromatography (PE/EA = 1:1) as a white solid (149 mg, 87% yield, l/b > 20:1). **^{1}H NMR** (400 MHz, DMSO-d_6) δ 9.97 (d, J = 3.7 Hz, 1H), 9.70 (d, J = 3.6 Hz, 1H), 7.85 (dtt, J = 9.1, 6.7, 3.4 Hz, 4H), 7.68 (d, J = 8.0 Hz, 2H), 7.36 (d, J = 8.0 Hz, 2H), 3.52 (t, J = 7.1 Hz, 2H), 2.39 (s, 3H), 1.95 (t, J = 7.2 Hz, 2H), 1.52 (h, J = 7.5, 6.9 Hz, 2H), 1.37 (dp, J = 14.9, 7.6 Hz, 2H), 1.10 (ddt, J = 15.2, 11.3, 6.3 Hz, 2H). **^{13}C NMR** (101 MHz, DMSO-d_6) δ 171.1, 168.4, 143.6, 136.4, 134.8, 132.1, 129.7, 128.3, 123.5, 37.7, 33.1, 28.2, 26.0, 24.9, 21.5. **HRMS** (ESI) calcd. for $C_{21}H_{24}N_3O_5S$ $[M+H]^+$: 430.1431, found: 430.1446

6-(1,3-dioxoisoindolin-2-yl)-*N*-(2-(2,6-dioxopiperidin-3-yl)-1-oxoisoindolin-4-yl)hexanamide (88)

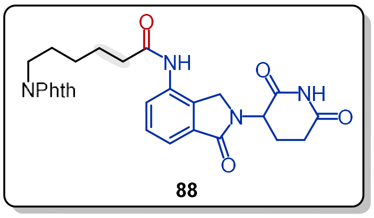

The title compound was prepared according to **general procedure D** using DPEphos as ligand and purified by column chromatography (PE/EA = 2:1) as a white solid (181 mg, 88% yield, l/b > 20:1). **^{1}H NMR** (400 MHz, $CDCl_3$) δ 9.72 (s, 1H), 7.94 – 7.72 (m, 5H), 7.50 – 7.39 (m, 2H), 5.71 (s, 2H), 5.12 (dd, J = 13.2, 5.1 Hz, 1H), 4.38 – 4.23 (m, 2H), 3.54 (t, J = 7.0 Hz, 2H), 2.95 – 2.82 (m, 1H), 2.61 – 2.53 (m, 1H), 2.31 (t, J = 7.2 Hz, 2H), 1.99 (ddd, J = 11.1, 5.5, 3.3 Hz, 1H), 1.59 (t, J = 7.6 Hz, 4H), 1.34 – 1.18 (m, 2H). **^{13}C NMR** (101 MHz, $CDCl_3$) δ 178.1, 176.4, 176.3, 173.2, 173.1, 139.6, 139.0, 138.9, 137.9, 136.8, 133.8, 130.4, 128.2, 124.2, 60.1, 56.7, 42.5, 40.8, 36.4, 33.0, 31.1, 29.8, 27.9. **HRMS** (ESI) calcd. for $C_{27}H_{26}N_4O_6Na$ $[M+Na]^+$: 525.1745, found: 525.1750

***S*-butyl 6-(1,3-dioxoisoindolin-2-yl)hexanethioate (89)**

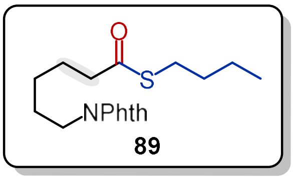

The title compound was prepared according to **general procedure D** using **L2** as ligand and purified by column chromatography (PE/EA = 20:1) as a white solid (124 mg, 93% yield, l/b > 20:1). **^{1}H NMR** (400 MHz, $CDCl_3$) δ 7.90 – 7.79 (m, 2H), 7.76 – 7.68 (m, 2H), 3.68 (t, J = 7.3 Hz, 2H), 2.88 – 2.82 (m, 2H), 2.60 – 2.51 (m, 2H), 1.77 – 1.64 (m, 4H), 1.59 – 1.47 (m, 2H), 1.46 – 1.30 (m, 4H), 0.90 (t, J = 7.3 Hz, 3H). **^{13}C NMR** (101 MHz, $CDCl_3$) δ 199.5, 168.4, 133.9, 132.1, 123.2, 43.8, 37.7, 31.6, 28.5, 28.3, 26.2, 25.2, 22.0, 13.6. **HRMS** (ESI) calcd. for $C_{18}H_{23}NO_3SNa$ $[M+Na]^+$: 356.1291, found: 356.1298

***S*-isopropyl 6-(1,3-dioxoisoindolin-2-yl)hexanethioat (90)**

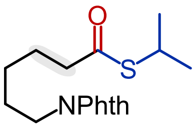

90

The title compound was prepared according to **general procedure D** using **L2** as ligand and purified by column chromatography (PE/EA = 20:1) as a white solid (111 mg, 87% yield, l/b > 20:1). **^{1}H NMR** (400 MHz, $CDCl_3$) δ 7.89 – 7.80 (m, 2H), 7.77 – 7.69 (m, 2H), 3.74 – 3.54 (m, 3H), 2.51 (t, *J* = 7.5 Hz, 2H), 1.77 – 1.64 (m, 4H), 1.45 – 1.33 (m, 2H), 1.27 (d, *J* = 6.9 Hz, 6H). **^{13}C NMR** (101 MHz, $CDCl_3$) δ 199.5, 168.4, 133.9, 132.1, 123.2, 43.8, 37.7, 34.5, 28.3, 26.1, 25.1, 23.0. **HRMS** (ESI) calcd. for $C_{17}H_{21}NO_3SNa$ $[M+Na]^+$: 342.1134, found: 342.1142

***S*-benzyl 6-(1,3-dioxoisoindolin-2-yl)hexanethioate (91)**

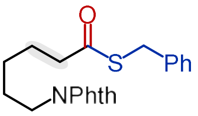

91

The title compound was prepared according to **general procedure D** using **L2** as ligand and purified by column chromatography (PE/EA = 20:1) as a white solid (132 mg, 90% yield, l/b > 20:1). **^{1}H NMR** (500 MHz, $CDCl_3$) δ 7.88 – 7.78 (m, 2H), 7.72 – 7.65 (m, 2H), 7.31 – 7.24 (m, 4H), 7.21 (ddt, *J* = 8.5, 6.8, 2.5 Hz, 1H), 4.08 (s, 2H), 3.65 (t, *J* = 7.3 Hz, 2H), 2.55 (t, *J* = 7.5 Hz, 2H), 1.77 – 1.63 (m, 4H), 1.43 – 1.32 (m, 2H). **^{13}C NMR** (101 MHz, $CDCl_3$) δ 198.6, 168.4, 137.6, 133.9, 132.1, 128.8, 128.6, 127.2, 123.2, 43.5, 37.7, 33.2, 28.3, 26.2, 25.1. **HRMS** (ESI) calcd. for $C_{21}H_{21}NO_3SNa$ $[M+Na]^+$: 390.1134, found: 390.1140

***S*-(*p*-tolyl) 6-(1,3-dioxoisoindolin-2-yl)hexanethioate (92)**

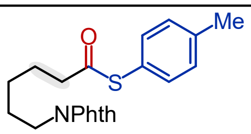

92

The title compound was prepared according to **general procedure D** using DPEphos as ligand and purified by column chromatography (PE/EA = 20:1) as a white solid (120 mg, 82% yield, l/b > 20:1). **^{1}H NMR** (500 MHz, $CDCl_3$) δ 7.88 – 7.78 (m, 2H), 7.75 – 7.66 (m, 2H), 7.30 – 7.23 (m, 2H), 7.19 (d, *J* = 8.0 Hz, 2H), 3.68 (t, *J* = 7.2 Hz, 2H), 2.63 (t, *J* = 7.4 Hz, 2H), 2.35 (s, 3H), 1.82 – 1.65 (m, 4H), 1.46 – 1.37 (m, 2H). **^{13}C NMR** (101 MHz, $CDCl_3$) δ 197.8, 168.4, 139.6, 134.5, 134.0, 133.9, 132.1, 130.0, 124.3, 123.2, 43.3, 37.7, 28.3, 26.2, 25.1, 21.4. **HRMS** (ESI) calcd. for $C_{21}H_{21}NO_3SNa$ $[M+Na]^+$: 390.1134, found: 390.1135

***S*-(4-methoxyphenyl) 6-(1,3-dioxoisoindolin-2-yl)hexanethioate (93)**

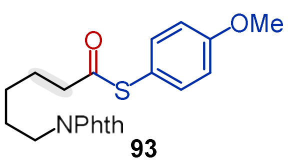

93

The title compound was prepared according to **general procedure D** using DPEphos as ligand and purified by column chromatography (PE/EA = 10:1) as a white solid (128 mg, 84% yield, l/b > 20:1). **^{1}H NMR** (500 MHz, $CDCl_3$) δ 7.85 – 7.80 (m, 2H), 7.73 – 7.67 (m, 2H), 7.31 – 7.26 (m, 2H), 6.94 – 6.88 (m, 2H), 3.80 (s, 3H), 3.68 (t, *J* = 7.2 Hz, 2H), 2.63 (t, *J* = 7.4 Hz, 2H), 1.72 (dp, *J* = 20.2, 7.5 Hz, 4H), 1.41 (tdd, *J* = 10.7, 8.4, 4.3 Hz, 2H). **^{13}C NMR** (101 MHz, $CDCl_3$) δ 198.4, 168.5, 160.6, 136.1, 133.9, 132.1, 123.2, 114.8, 55.4, 43.1, 37.7, 28.3, 26.2, 25.1. **HRMS** (ESI) calcd. for $C_{21}H_{21}NO_4SNa$ $[M+Na]^+$: 406.1083, found: 406.1085

***S*-(4-chlorophenyl) 6-(1,3-dioxoisoindolin-2-yl)hexanethioate (94)**

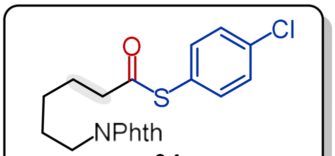

94

The title compound was prepared according to **general procedure D** using DPEphos as ligand and purified by column chromatography (PE/EA = 20:1) as a white solid (118 mg, 76% yield, l/b > 20:1). **^{1}H NMR** δ 7.83 (dt, *J* = 7.1, 3.6 Hz, 2H), 7.70 (dd, *J* = 5.5, 3.0 Hz, 2H), 7.38 – 7.26 (m, 4H), 3.69 (t, *J* = 7.2 Hz, 2H), 2.66 (t, *J* = 7.4 Hz, 2H), 1.82 – 1.65 (m, 4H), 1.48 – 1.36 (m, 2H). **^{13}C NMR** (101 MHz, $CDCl_3$) δ 196.8, 168.5, 135.7, 134.0, 132.1, 129.4, 126.2, 123.2, 43.4, 37.7, 28.2, 26.1, 25.0. **HRMS** (ESI) calcd. for $C_{20}H_{18}ClNO_3SNa$ $[M+Na]^+$: 410.0588, found: 410.0590

***S*-(4-bromophenyl) 6-(1,3-dioxoisoindolin-2-yl)hexanethioate (95)**

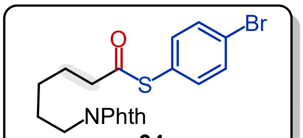

94

The title compound was prepared according to **general procedure D** using DPEphos as ligand and purified by column chromatography (PE/EA = 20:1) as a white solid (149 mg, 86% yield, l/b > 20:1). **^{1}H NMR** (500 MHz, $CDCl_3$) δ 7.85 – 7.78 (m, 2H), 7.73 – 7.66 (m, 2H), 7.54 – 7.46 (m, 2H), 7.28 – 7.20 (m, 2H), 3.68 (t, *J* = 7.2 Hz, 2H), 2.65 (t, *J* = 7.5 Hz, 2H), 1.82 – 1.66 (m, 4H), 1.47 – 1.37 (m, 2H). **^{13}C NMR** (101 MHz, $CDCl_3$) δ 196.6, 196.6, 185.5, 168.4, 135.9, 134.0, 132.4, 132.1, 126.9, 124.0, 123.2, 43.4, 37.7, 28.2, 26.1, 25.0. **HRMS** (ESI) calcd. for $C_{20}H_{18}BrNO_3SNa$ $[M+Na]^+$: 454.0083, found: 454.0091.

3-phenylpropanal (96)

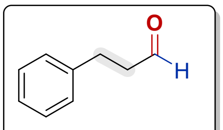

96

The title compound was prepared according to **general procedure E** using Xantphos as ligand and purified by column chromatography (PE/EA = 100:1) as a colourless oil. The yield and regioselectivity were measured by GC-FID (73 % yield, l/b > 20:1). **^{1}H NMR** (400 MHz, $CDCl_3$) δ 9.82 (t, *J* = 1.4 Hz, 1H), 7.34 – 7.27 (m, 2H), 7.21 (td, *J* = 7.3, 6.8, 1.6 Hz, 3H), 2.96 (t, *J* = 7.6 Hz, 2H), 2.82 – 2.76 (m, 2H). **^{13}C NMR** (101 MHz, $CDCl_3$) δ 201.8, 140.4, 128.7, 128.4, 126.4, 45.4, 28.2.
The structure was confirmed according to the precedents reported in the literature.[7]

3-(p-tolyl)propanal (97)

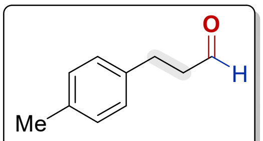

97

The title compound was prepared according to **general procedure E** using Xantphos as ligand and purified by column chromatography (PE/EA = 100:1) as a colourless oil (37 mg, 62 % yield, l/b > 20:1). **^{1}H NMR** (400 MHz, $CDCl_3$) δ 9.82 (t, *J* = 1.5 Hz, 1H), 7.20 – 7.01 (m, 4H), 2.92 (t, *J* = 7.5 Hz, 2H), 2.76 (td, *J* = 7.8, 1.3 Hz, 2H), 2.32 (s, 3H). **^{13}C NMR** (101 MHz, $CDCl_3$) δ 201.9, 137.2, 135.9, 129.3, 128.2, 45.5, 27.7, 21.0.
The structure was confirmed according to the precedents reported in the literature.[10]

3-(4-chlorophenyl)propanal (98)

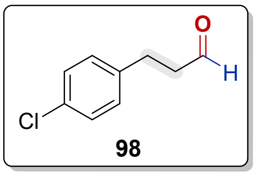

The title compound was prepared according to **general procedure E** using Xantphos as ligand and purified by column chromatography (PE/EA = 100:1) as a colourless oil (33 mg, 49 % yield, l/b > 20:1). **^{1}H NMR** (400 MHz, $CDCl_3$) δ 9.81 (d, J = 1.3 Hz, 1H), 7.30 – 7.23 (m, 2H), 7.17 – 7.04 (m, 2H), 2.93 (t, J = 7.4 Hz, 2H), 2.78 (t, J = 7.4 Hz, 2H). **^{13}C NMR** (101 MHz, $CDCl_3$) δ 201.2, 138.8, 132.1, 129.7, 128.7, 45.2, 27.4.

The structure was confirmed according to the precedents reported in the literature.[7]

3-(4-bromophenyl)propanal (99)

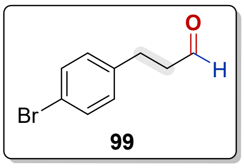

The title compound was prepared according to **general procedure E** using Xantphos as ligand and purified by column chromatography (PE/EA = 100:1) as a colourless oil (43 mg, 50 % yield, l/b > 20:1). **^{1}H NMR** (500 MHz, $CDCl_3$) δ 9.81 (t, J = 1.3 Hz, 1H), 7.53 – 7.35 (m, 2H), 7.15 – 7.02 (m, 2H), 2.91 (t, J = 7.4 Hz, 2H), 2.77 (tt, J = 7.6, 1.0 Hz, 2H). **^{13}C NMR** (126 MHz, $CDCl_3$) δ 201.2, 139.5, 131.8, 130.2, 120.2, 45.2, 27.6.

The structure was confirmed according to the precedents reported in the literature.[7]

3-(o-tolyl)propanal (100)

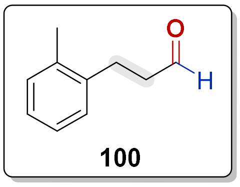

The title compound was prepared according to **general procedure E** using Xantphos as ligand and purified by column chromatography (PE/EA = 100:1) as a colourless oil (32 mg, 54 % yield, l/b > 20:1). **^{1}H NMR** (400 MHz, $CDCl_3$) δ 9.85 (t, J = 1.4 Hz, 1H), 7.20 – 7.00 (m, 4H), 3.01 – 2.90 (m, 2H), 2.81 – 2.69 (m, 2H), 2.32 (s, 3H). **^{13}C NMR** (101 MHz, $CDCl_3$) δ 201.8, 138.6, 136.1, 130.5, 128.6, 126.6, 126.4, 44.2, 25.6, 19.4.

The structure was confirmed according to the precedents reported in the literature.[10]

heptanal (101)

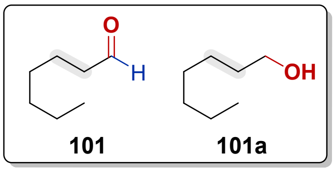

The title compound was prepared according to **general procedure F** using DPEphos as ligand. The yield and regioselectivity were measured by GC-FID (85 % yield, l/b > 20:1). To confirm the structure, the compound was reduced with $NaBH_4$ to give **100a** and purified by column chromatography (PE/EA = 10:1) as a colourless oil. **^{1}H NMR** (400 MHz, $CDCl_3$) δ 3.64 (t, J = 6.6 Hz, 2H), 1.61 – 1.53 (m, 2H), 1.37 – 1.27 (m, 8H), 0.93 – 0.82 (m, 3H). **^{13}C NMR** (101 MHz, $CDCl_3$) δ 63.2, 32.9, 32.0, 29.2, 25.8, 22.7, 14.2.

The structure was confirmed according to the precedents reported in the literature.[22]

3-cyclohexylpropanal (102)

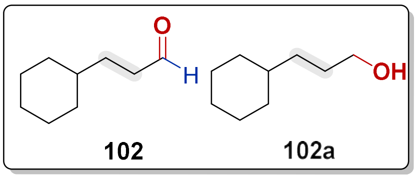

The title compound was prepared according to **general procedure F** using DPEphos as ligand. The yield and regioselectivity were measured by GC-FID (68 % yield, l/b > 20:1). To confirm the structure, the compound was reduced with $NaBH_4$ to give **101a** and purified by column chromatography (PE/EA = 10:1) as a colourless oil. **^{1}H NMR** (400 MHz, $CDCl_3$) δ 3.63 (t, J = 6.7 Hz, 2H), 1.78 – 1.61 (m, 5H), 1.60 – 1.54 (m, 2H), 1.30 – 1.15 (m, 6H), 0.92 (d, J = 3.1 Hz, 2H). **^{13}C NMR** (101 MHz, $CDCl_3$) δ 63.5, 37.5, 33.4, 33.4, 30.2, 26.7, 26.4.

The structure was confirmed according to the precedents reported in the literature.[18]

4,4-dimethylpentanal (103)

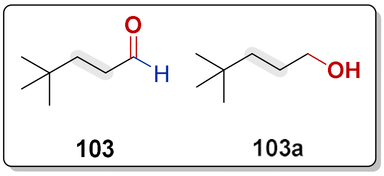

The title compound was prepared according to **general procedure F** using DPEphos as ligand. The yield and regioselectivity were measured by GC-FID (90 % yield, l/b > 20:1). To confirm the structure, the compound was reduced with $NaBH_4$ to give **102a** and purified by column chromatography (PE/EA = 10:1) as a colourless oil. **^{1}H NMR** (400 MHz, $CDCl_3$) δ 3.63 (t, J = 6.7 Hz, 2H), 1.58 – 1.51 (m, 2H), 1.26 – 1.20 (m, 2H), 0.89 (s, 9H). **^{13}C NMR** (101 MHz, $CDCl_3$) δ 64.0, 39.9, 30.1, 29.4, 28.0.

The structure was confirmed according to the precedents reported in the literature.[6]

6-phenoxyhexanal (104)

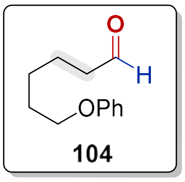

The title compound was prepared according to **general procedure F** using DPEphos as ligand and purified by column chromatography (PE/EA = 30:1) as a colourless oil (56 mg, 72 % yield, l/b > 20:1). **^{1}H NMR** (400 MHz, $CDCl_3$) δ 9.77 (t, J = 2.1 Hz, 1H), 7.34 – 7.20 (m, 2H), 6.98 – 6.75 (m, 3H), 3.95 (tt, J = 8.7, 4.3 Hz, 2H), 2.47 (tt, J = 7.4, 2.1 Hz, 2H), 1.87 – 1.75 (m, 2H), 1.75 – 1.65 (m, 2H), 1.52 (dtd, J = 9.9, 6.2, 4.6, 2.0 Hz, 2H). **^{13}C NMR** (101 MHz, $CDCl_3$) δ 202.7, 159.1, 129.5, 120.7, 114.6, 67.5, 43.9, 29.2, 25.8, 21.9.

The structure was confirmed according to the precedents reported in the literature.[3]

6-oxohexyl benzoate (105)

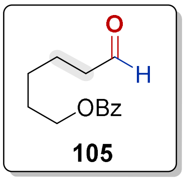

The title compound was prepared according to **general procedure F** using DPEphos as ligand and purified by column chromatography (PE/EA = 20:1) as a colourless oil (75 mg, 85 % yield, l/b > 20:1). **^{1}H NMR** (400 MHz, $CDCl_3$) δ 9.78 (t, J = 1.7 Hz, 1H), 8.10 – 7.98 (m, 2H), 7.62 – 7.52 (m, 1H), 7.50 – 7.37 (m, 2H), 4.33 (t, J = 6.6 Hz, 2H), 2.48 (td, J = 7.3, 1.7 Hz, 2H), 1.80 (dq, J = 8.2, 6.7 Hz, 2H), 1.76 – 1.68 (m, 2H), 1.56 – 1.46 (m, 2H). **^{13}C NMR** (101 MHz, $CDCl_3$) δ 202.5, 166.8, 133.0, 130.5, 129.7, 128.5, 64.8, 43.9, 28.7, 25.8, 21.8.

The structure was confirmed according to the precedents reported in the literature.[26]

N-benzyl-4-methyl-N-(5-oxopentyl)benzenesulfonamide (106)

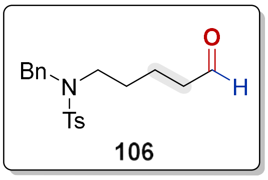

The title compound was prepared according to **general procedure F** using DPEphos as ligand and purified by column chromatography (PE/EA = 10:1) as a colourless oil (97 mg, 70 % yield, l/b > 20:1). **^{1}H NMR** (400 MHz, $CDCl_3$) δ 9.59 (t, J = 1.6 Hz, 1H), 7.89 – 7.62 (m, 2H), 7.46 – 7.24 (m, 7H), 4.28 (s, 2H), 3.08 (t, J = 7.2 Hz, 2H), 2.44 (s, 3H), 2.24 (td, J = 7.1, 1.6 Hz, 2H), 1.48 – 1.38 (m, 2H), 1.37 – 1.30 (m, 2H). **^{13}C NMR** (101 MHz, $CDCl_3$) δ 202.2, 143.5, 136.8, 136.6, 129.9, 128.7, 128.5, 128.0, 127.3, 52.5, 48.0, 43.2, 27.7, 21.7, 19.1. **HRMS** (ESI) calcd. for $C_{19}H_{24}NO_3S$ $[M+H]^+$: 346.1471, found: 346.1467.

6-(1,3-dioxoisoindolin-2-yl)hexanal (107)

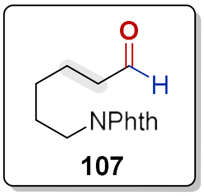

The title compound was prepared according to **general procedure F** using DPEphos as ligand and purified by column chromatography (PE/EA = 10:1) as a colourless oil (77 mg, 79 % yield, l/b > 20:1). **^{1}H NMR** (500 MHz, $CDCl_3$) δ 9.76 (d, J = 1.8 Hz, 1H), 7.91 – 7.81 (m, 2H), 7.77 – 7.68 (m, 2H), 3.69 (t, J = 7.2 Hz, 2H), 2.45 (td, J = 7.3, 1.7 Hz, 2H), 1.69 (dq, J = 10.7, 7.5 Hz, 4H), 1.44 – 1.35 (m, 2H). **^{13}C NMR** (126 MHz, $CDCl_3$) δ 202.6, 168.6, 134.1, 132.2, 123.3, 43.8, 37.8, 28.5, 26.4, 21.7.
The structure was confirmed according to the precedents reported in the literature.[12]

7-(9H-carbazol-9-yl)heptanal (108)

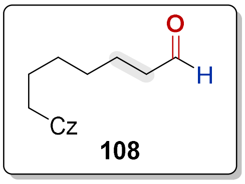

The title compound was prepared according to **general procedure F** using DPEphos as ligand and purified by column chromatography (PE/EA = 30:1) as a colourless oil (92 mg, 82 % yield, l/b > 20:1). **^{1}H NMR** (400 MHz, $CDCl_3$) δ 9.69 (t, J = 1.7 Hz, 1H), 8.09 (dt, J = 7.8, 1.0 Hz, 2H), 7.45 (ddd, J = 8.2, 7.1, 1.2 Hz, 2H), 7.38 (dt, J = 8.2, 1.0 Hz, 2H), 7.22 (ddd, J = 7.9, 7.0, 1.1 Hz, 2H), 4.27 (t, J = 7.1 Hz, 2H), 2.34 (td, J = 7.3, 1.7 Hz, 2H), 1.86 (p, J = 7.2 Hz, 2H), 1.59 – 1.49 (m, 2H), 1.41 – 1.30 (m, 4H). **^{13}C NMR** (101 MHz, $CDCl_3$) δ 202.7, 140.5, 125.7, 122.9, 120.5, 118.9, 108.7, 43.8, 43.0, 29.0, 28.9, 27.2, 22.0. **HRMS** (ESI) calcd. for $C_{19}H_{21}NO$ $[M+H]^+$: 280.1696, found: 280.1696.

6-oxo-6-phenylhexanal (109)

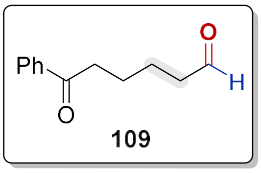

The title compound was prepared according to **general procedure F** using DPEphos as ligand and purified by column chromatography (PE/EA = 20:1) as a colourless oil (46 mg, 60 % yield, l/b > 20:1). **^{1}H NMR** (500 MHz, $CDCl_3$) δ 9.79 (t, J = 1.6 Hz, 1H), 8.06 – 7.92 (m, 2H), 7.65 – 7.54 (m, 1H), 7.54 – 7.42 (m, 2H), 3.02 (t, J = 6.9 Hz, 2H), 2.52 (td, J = 7.1, 1.6 Hz, 2H), 1.90 – 1.72 (m, 4H). **^{13}C NMR** (126 MHz, $CDCl_3$) δ 202.5, 199.9, 137.0, 133.2, 128.8, 128.1, 43.9, 38.3, 23.7, 21.8.
The structure was confirmed according to the precedents reported in the literature.[19]

3-phenylbutanal (110)

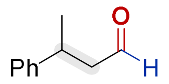

110

The title compound was prepared according to **general procedure E** using Xantphos as ligand and purified by column chromatography (PE/EA = 100:1) as a colourless oil (54 mg, 92 % yield, l/b > 20:1). **^{1}H NMR** (400 MHz, $CDCl_3$) δ 9.71 (t, *J* = 2.0 Hz, 1H), 7.31 (dd, *J* = 8.3, 6.9 Hz, 2H), 7.25 – 7.18 (m, 3H), 3.36 (h, *J* = 7.1 Hz, 1H), 2.82 – 2.62 (m, 2H), 1.32 (d, *J* = 7.0 Hz, 3H). **^{13}C NMR** (101 MHz, $CDCl_3$) δ 202.1, 145.6, 128.8, 126.9, 126.7, 51.8, 34.4, 22.3.

The structure was confirmed according to the precedents reported in the literature.[9]

3-cyclobutyl-3-phenylpropanal (111)

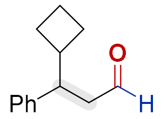

111

The title compound was prepared according to **general procedure E** using Xantphos as ligand and purified by column chromatography (PE/EA = 100:1) as a colourless oil (54 mg, 72 % yield, l/b > 20:1). **^{1}H NMR** (400 MHz, $CDCl_3$) δ 9.63 (t, *J* = 2.3 Hz, 1H), 7.29 (dd, *J* = 8.2, 6.8 Hz, 2H), 7.23 – 7.14 (m, 3H), 3.09 (ddd, *J* = 10.4, 8.3, 6.1 Hz, 1H), 2.71 – 2.61 (m, 2H), 2.51 (dq, *J* = 13.0, 7.8 Hz, 1H), 2.09 (tdt, *J* = 11.2, 8.1, 3.6 Hz, 1H), 1.82 – 1.68 (m, 4H), 1.62 (d, *J* = 10.2 Hz, 1H). **^{13}C NMR** (101 MHz, $CDCl_3$) δ 202.5, 142.3, 128.7, 127.8, 126.7, 47.9, 47.1, 41.5, 27.7, 26.9, 17.5.

The structure was confirmed according to the precedents reported in the literature.[13]

3-cyclohexyl-3-phenylpropanal (112)

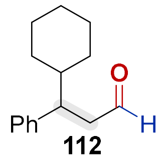

112

The title compound was prepared according to **general procedure E** using Xantphos as ligand and purified by column chromatography (PE/EA = 100:1) as a colourless oil (78 mg, 90 % yield, l/b > 20:1). **^{1}H NMR** (400 MHz, $CDCl_3$) δ 9.59 (t, *J* = 2.3 Hz, 1H), 7.28 (dd, *J* = 8.2, 6.7 Hz, 2H), 7.23 – 7.17 (m, 1H), 7.14 (dd, *J* = 7.1, 1.6 Hz, 2H), 2.97 (ddd, *J* = 9.6, 7.5, 5.3 Hz, 1H), 2.83 (ddd, *J* = 16.4, 5.4, 1.9 Hz, 1H), 2.72 (ddd, *J* = 16.4, 9.6, 2.6 Hz, 1H), 1.84 – 1.70 (m, 2H), 1.62 (tdd, *J* = 10.4, 5.9, 3.4 Hz, 2H), 1.48 (dddd, *J* = 16.0, 13.6, 6.4, 3.0 Hz, 2H), 1.22 (qt, *J* = 12.6, 3.2 Hz, 1H), 1.14 – 1.01 (m, 2H), 0.94 (qd, *J* = 12.2, 3.3 Hz, 1H), 0.81 (qd, *J* = 11.8, 10.6, 5.7 Hz, 1H). **^{13}C NMR** (101 MHz, $CDCl_3$) δ 202.9, 142.9, 128.5, 128.4, 126.6, 47.2, 46.3, 43.2, 31.2, 30.8, 26.5, 26.4.

The structure was confirmed according to the precedents reported in the literature.[13]

2-(4-phenylcyclohexyl)acetaldehyde (113)

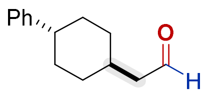

113

The title compound was prepared according to **general procedure F** using DPEphos as ligand and purified by column chromatography (PE/EA = 100:1) as a colourless oil (69 mg, 86 % yield, l/b > 20:1, d.r. = 20:1). **^{1}H NMR** (400 MHz, $CDCl_3$) δ 9.80 (t, *J* = 2.2 Hz, 1H), 7.30 (dd, *J* = 8.2, 6.9 Hz, 2H), 7.20 (dt, *J* = 7.9, 1.9 Hz, 3H), 2.48 (tt, *J* = 12.3, 3.3 Hz, 1H), 2.37 (dd, *J* = 6.7, 2.3 Hz, 2H), 1.91 (ddd, *J* = 15.1, 9.4, 3.3 Hz, 4H), 1.58 (d, *J* = 3.2 Hz, 1H), 1.57 – 1.46 (m, 2H), 1.20 (qd, *J* =

13.6, 12.7, 4.2 Hz, 2H). **^{13}C NMR** (101 MHz, $CDCl_3$) δ 202.9, 147.3, 128.5, 126.9, 126.1, 51.4, 44.1, 34.0, 33.5, 32.4. **HRMS** (ESI) calcd. for $C_{14}H_{19}O$ $[M+H]^+$: 203.1430, found: 203.1426.

tert-butyl 4-(2-oxoethyl)piperidine-1-carboxylate (114)

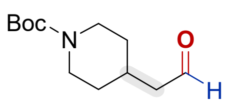

114

The title compound was prepared according to **general procedure F** using DPEphos as ligand and purified by column chromatography (PE/EA = 20:1) as a colourless oil (80 mg, 88 % yield, l/b > 20:1). **^{1}H NMR** (400 MHz, $CDCl_3$) δ 9.78 (t, J = 1.8 Hz, 1H), 4.08 (s, 2H), 2.72 (d, J = 13.4 Hz, 2H), 2.39 (dd, J = 6.8, 1.8 Hz, 2H), 2.06 (dddd, J = 18.3, 11.5, 6.8, 3.2 Hz, 1H), 1.75 – 1.65 (m, 2H), 1.45 (s, 9H), 1.23 – 1.13 (m, 2H). **^{13}C NMR** (101 MHz, $CDCl_3$) δ 201.7, 154.9, 79.5, 50.5, 43.8, 32.0, 30.8, 28.5.
The structure was confirmed according to the precedents reported in the literature.[8]

cyclohexanecarbaldehyde (115)

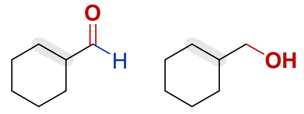

115 115a

The title compound was prepared according to **general procedure F** using DPEphos as ligand for 48 hours. The yield and regioselectivity were measured by GC-FID (76 % yield, l/b > 20:1). To confirm the structure, the compound was reduced with $NaBH_4$ to give **114a** and purified by column chromatography (PE/EA = 10:1) as a colourless oil. **^{1}H NMR** (400 MHz, $CDCl_3$) δ 3.44 (d, J = 6.4 Hz, 2H), 1.82 – 1.65 (m, 5H), 1.48 (dddt, J = 14.7, 8.2, 6.4, 3.3 Hz, 1H), 1.30 – 1.08 (m, 3H), 0.93 (ddd, J = 21.0, 10.5, 2.7 Hz, 2H). **^{13}C NMR** (101 MHz, $CDCl_3$) δ 68.9, 40.6, 29.7, 26.7, 26.0.
The structure was confirmed according to the precedents reported in the literature.[6]

7-phenylheptanal (116)

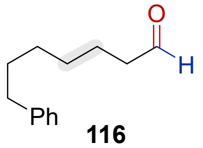

116

The title compound was prepared according to **general procedure F** using DPEphos as ligand for 48 hours and purified by column chromatography (PE/EA = 20:1) as a colourless oil (49 mg, 65 % yield, l/b = 12:1). **^{1}H NMR** (400 MHz, $CDCl_3$) δ 9.75 (t, J = 1.8 Hz, 1H), 7.30 – 7.24 (m, 2H), 7.17 (td, J = 6.4, 1.7 Hz, 3H), 2.60 (dd, J = 8.6, 6.8 Hz, 2H), 2.41 (td, J = 7.4, 1.9 Hz, 2H), 1.66 – 1.58 (m, 4H), 1.41 – 1.29 (m, 4H). **^{13}C NMR** (101 MHz, $CDCl_3$) δ 203.0, 142.7, 128.5, 128.4, 125.8, 44.0, 36.0, 31.4, 29.1, 29.1, 22.1.
The structure was confirmed according to the precedents reported in the literature.[27]

4-phenylbutanal (117)

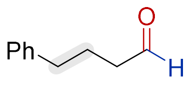

117

The title compound was prepared according to **general procedure E** using Xantphos as ligand for 48 hours and purified by column chromatography (PE/EA = 20:1) as a colourless oil (30 mg, 52 % yield, l/b = 5:1). **^{1}H NMR** (400 MHz, $CDCl_3$) δ 9.76 (t, J = 1.6 Hz, 1H), 7.32 – 7.27 (m, 2H), 7.23 – 7.15 (m, 3H), 2.76 – 2.60 (m, 2H), 2.46 (td, J = 7.4,

1.7 Hz, 2H), 2.06 – 1.92 (m, 2H). **^{13}C NMR** (101 MHz, $CDCl_3$) δ 202.5, 141.3, 128.6, 128.4, 126.2, 43.3, 35.1, 23.8.
The structure was confirmed according to the precedents reported in the literature.[5]

2-((8*S*,9*S*,13*R*,14*S*,17*R*)-3-methoxy-13-methyl-7,8,9,11,12,13,14,15,16,17-decahydro-6*H*-cyclopenta[*a*]phenanthren-17-yl)acetaldehyde (118)

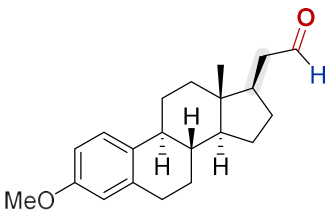

118

The title compound was prepared according to **general procedure F** using DPEphos as ligand and purified by column chromatography (PE/EA = 20:1) as a white solid (100 mg, 74 % yield, l/b > 20:1, d.r. = 10:1). **^{1}H NMR** (400 MHz, $CDCl_3$) δ 9.85 – 9.72 (m, 1H), 7.20 (d, J = 8.6 Hz, 1H), 6.71 (dd, J = 8.6, 2.8 Hz, 1H), 6.63 (d, J = 2.8 Hz, 1H), 3.77 (s, 3H), 2.94 – 2.80 (m, 2H), 2.66 – 2.47 (m, 1H), 2.34 – 2.17 (m, 3H), 2.06 – 1.85 (m, 3H), 1.79 (dt, J = 12.7, 3.5 Hz, 2H), 1.51 – 1.37 (m, 3H), 1.36 – 1.22 (m, 4H), 0.64 (s, 3H). **^{13}C NMR** (101 MHz, $CDCl_3$) δ 203.3, 157.5, 138.1, 132.8, 126.4, 113.9, 111.5, 55.3, 54.3, 45.4, 44.8, 44.1, 42.6, 38.9, 37.6, 30.0, 28.4, 27.9, 26.4, 24.5, 12.9. **HRMS** (ESI) calcd. for $C_{21}H_{29}O_2$ $[M+H]^+$: 313.2162, found: 313.2157.

5. Synthetic applications

5.1. Hydrocarbonylation of ethylene

5.1.1. Hydroxycarbonylation of ethylene

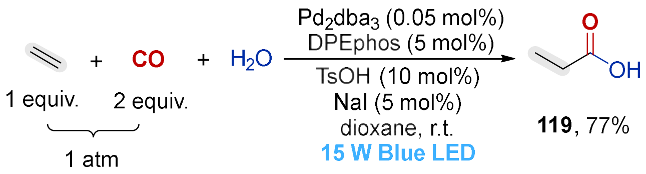

To a flame-dried Young-type tube was added Pd_2dba_3 (30 μL of 0.005M solvent in dioxane, 0.00015 mmol) and DPEphos (8.1 mg, 0.015 mmol). The tube was evacuated and refilled with N_2, then H_2O (144 mg, 8 mmol), TsOH·H_2O (5.7 mg, 0.030 mmol), NaI (2.3 mg, 0.015 mmol) and dioxane (1.0 mL) were added under the N_2 atmosphere. After that, the resulting mixture was degassed with the freeze-thaw method, introduced 1 atm gas mixture (ethylene:CO = 1:2) and irradiated under 15W 455 nm blue LEDs for 20 hours. After completion, the reaction mixture was concentrated under reduced pressure. Weighing the Young-type tube before and after introducing the gas mixture determined that 0.30 mmol of ethylene was injected. Using n-tetradecane as an internal standard, gas chromatography analysis showed a yield of 77%.

5.1.2. Hydraminocarbonylation of ethylene

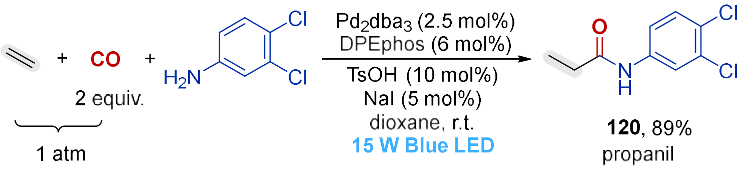

To a flame-dried Young-type tube was added Pd_2dba_3 (9.1 mg, 0.010 mmol) and DPEphos (13 mg, 0.060 mmol). The tube was evacuated and refilled with N_2, then H_2O (144 mg, 0.6 mmol), TsOH·H_2O (7.6 mg, 0.040 mmol), NaI (3.0 mg, 0.020 mmol) and dioxane (2.0 mL) were added under the N_2 atmosphere. After that, the resulting mixture was degassed with the freeze-thaw method, introduced 1 atm gas mixture (ethylene:CO = 1:2) and irradiated under 15W 455 nm blue LEDs for 20 hours. After completion, the reaction mixture was concentrated under reduced pressure. Weighing the Young-type tube before and after introducing the gas mixture determined that 0.26 mmol of ethylene was injected. After completion, the reaction mixture was concentrated under reduced pressure. The residue was purified by silica gel column chromatography to afford the desired product in a yield of 89% (52 mg).

N-(3,4-dichlorophenyl)propionamide (120)

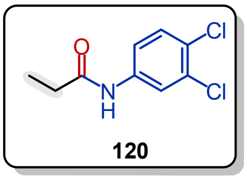

^{1}H NMR (400 MHz, $CDCl_3$) δ 7.80 – 7.73 (m, 1H), 7.38 – 7.29 (m, 2H), 2.40 (qd, *J* = 7.5, 2.5 Hz, 2H), 1.27 – 1.20 (m, 3H). **^{13}C NMR** (101 MHz, $CDCl_3$) δ 172.1, 137.4, 132.7, 130.5, 127.3, 121.4, 118.9, 30.7, 9.5. **HRMS** (ESI) calcd. for $C_9H_9Cl_2NONa$ $[M+Na]^+$: 239.9953, found: 239.9962

5.2. Hydrocarbonylation of mixture of hexene isomers

5.1.1. Hydroxycarbonylation of hexene isomers

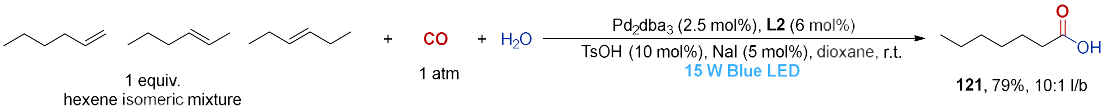

To a flame-dried Young-type tube was added Pd_2dba_3 (9.1 mg, 0.010 mmol, 2.5 mol%) and **L2** (16 mg, 0.024 mmol, 6 mol%). The tube was evacuated and refilled with N_2, then mixture of hexene (0.40 mmol, 1.0 equiv., 1:1:1), H_2O (144 mg, 8 mmol, 20 equiv.), $TsOH{\cdot}H_2O$ (7.6 mg, 0.040 mmol, 10 mol%), NaI (3.0 mg, 0.020 mmol, 5 mol%) and dioxane (2.0 mL) were added under the N_2 atmosphere. After that, the resulting mixture was degassed with the freeze-thaw method, introduced 1 atm CO and irradiated under 15W 455 nm blue LEDs for 20 hours. Two parallel reactions were set up. After completion, the reaction mixture was concentrated under reduced pressure. The residue was purified by silica gel column chromatography to afford the desired product in a yield of 79% (82 mg) and l/b = 10:1.

1-heptanoic acid (121)

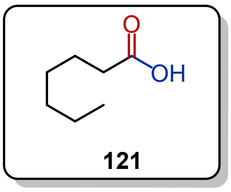

^{1}H NMR (400 MHz, $CDCl_3$) δ 11.54 (s, 1H), 2.35 (dd, *J* = 8.7, 6.4 Hz, 2H), 1.69 – 1.57 (m, 2H), 1.32 (qdd, *J* = 9.5, 7.3, 5.7, 2.6 Hz, 6H), 0.93 – 0.82 (m, 3H). **^{13}C NMR** (101 MHz, $CDCl_3$) δ 180.6, 34.2, 31.4, 28.7, 24.6, 22.5, 14.0.

The structure was confirmed according to the precedents reported in the literature.[15]

5.2.2. Hydroformylation of ethylene

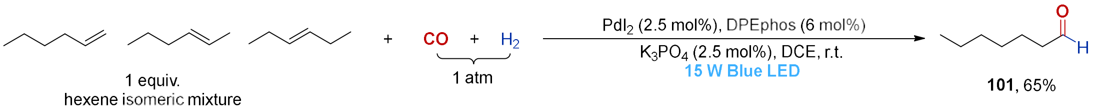

To a flame-dried Young-type tube was added PdI_2 (1.8 mg, 0.005 mmol, 2.5 mol%) DPEphos (3.2 mg, 0.006 mmol, 3 mol%) and K_3PO_4 (0.8 mg, 0.004 mmol, 2 mol%). The tube was evacuated and refilled with N_2, then mixture of hexene (0.20 mmol, 1.0 equiv., 1:1:1) and DCE (1.0 mL) were added under the N_2 atmosphere. After that, the resulting mixture was degassed with the freeze-thaw method, introduced 1 atm gas mixture ($CO:H_2$ = 1:3) and irradiated under 15W 455 nm blue LEDs at 25°C for 48

hours. Two parallel reactions were set up. After completion, the yield and regioselectivity of **101** were measured by GC-FID (65 % yield, l/b > 20:1).

5.3. Hydrocarbonylation with bioactive molecules

5.3.1. Hydroesterification of styrene with methyl (tert-butoxycarbonyl)-L-serinate

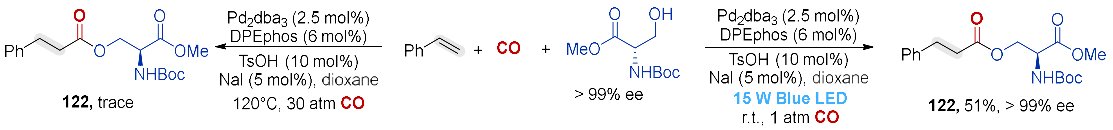

To a flame-dried Young-type tube was added Pd_2dba_3 (9.1 mg, 0.010 mmol, 2.5 mol%) and DPEphos (13 mg, 0.024 mmol, 6 mol%). The tube was evacuated and refilled with N_2, then styrene (42 mg, 0.40 mmol, 1.0 equiv.), methyl (tert-butoxycarbonyl)-L-serinate (175 mg, 0.8 mmol, 2 equiv.), $TsOH \cdot H_2O$ (7.6 mg, 0.040 mmol, 10 mol%), NaI (3.0 mg, 0.020 mmol, 5 mol%) and dioxane (2.0 mL) were added under the N_2 atmosphere. After that, the resulting mixture was degassed with the freeze-thaw method, introduced 1 atm CO and irradiated under 15W 455 nm blue LEDs for 20 hours. After completion, the reaction mixture was concentrated under reduced pressure. The residue was purified by silica gel column chromatography to afford the desired product in a yield of 51% (72 mg, l/b > 20:1) and ee > 99%.
Pd_2dba_3 (9.1 mg, 0.010 mmol, 2.5 mol%) and DPEphos (13 mg, 0.024 mmol, 6 mol%), styrene (42 mg, 0.40 mmol, 1.0 equiv.), methyl (tert-butoxycarbonyl)-L-serinate (175 mg, 2 mmol, 5 equiv.), $TsOH \cdot H_2O$ (7.6 mg, 0.040 mmol, 10 mol%), NaI (3.0 mg, 0.020 mmol, 5 mol%) and dioxane (2.0 mL) were added to a dry glass vessel. The glass vessel was put into an autoclave. Then, the autoclave was purged and charged with CO (30 atm). The reaction mixture was stirred at 120 °C for 24 hours. Only trace of product was detected by TLC.

methyl *N*-(tert-butoxycarbonyl)-*O*-(3-phenylpropanoyl)-L-serinate (122)

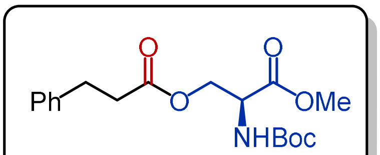

122

^{1}H NMR (400 MHz, $CDCl_3$) δ 7.32 – 7.27 (m, 2H), 7.23 – 7.16 (m, 3H), 5.22 (d, *J* = 8.5 Hz, 1H), 4.56 (dt, *J* = 8.2, 3.5 Hz, 1H), 4.43 (dd, *J* = 11.2, 4.1 Hz, 1H), 4.32 (dd, *J* = 11.2, 3.5 Hz, 1H), 3.73 (s, 3H), 2.98 – 2.86 (m, 2H), 2.69 – 2.57 (m, 2H), 1.46 (s, 9H). **^{13}C NMR** (101 MHz, $CDCl_3$) δ 172.4, 170.3, 155.2, 140.2, 128.6, 128.2, 126.4, 80.4, 64.3, 52.7, 35.5, 30.7, 28.3. **HRMS** (ESI) calcd. for $C_{18}H_{25}NO_6Na$ $[M+Na]^+$: 374.1574, found: 374.1578. The ee value was determined to be > 99% ee. [Determined by HPLC with a Chiralcel OD-H column (hexane: isopropanol = 90:10, 1 mL/min, 220 nm, 25 °C); t (major) = 11.5 min, t (minor) = 14.5 min]. $[\alpha]_D^{20}$ = 5.0 (CH_2Cl_2, *c* 0.10).

5.3.2. Hydroesterification of methyl (2*S*)-2-tert-butoxycarbonylamino-4-pentenoate

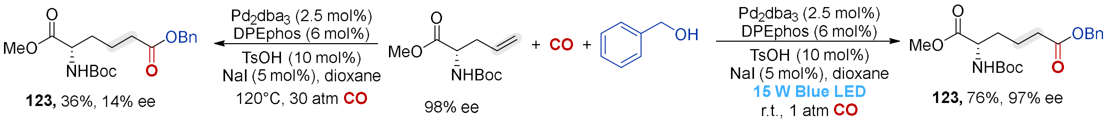

To a flame-dried Young-type tube was added Pd_2dba_3 (9.1 mg, 0.010 mmol, 2.5 mol%) and DPEphos (13 mg, 0.024 mmol, 6 mol%). The tube was evacuated and refilled with N_2, then (2S)-2-tert-butoxycarbonylamino-4-pentenoate (92 mg, 0.40 mmol, 1.0 equiv.), benzyl alcohol (216 mg, 2 mmol, 5 equiv.), TsOH·H_2O (7.6 mg, 0.040 mmol, 10 mol%), NaI (3.0 mg, 0.020 mmol, 5 mol%) and dioxane (2.0 mL) were added under the N_2 atmosphere. After that, the resulting mixture was degassed with the freeze-thaw method, introduced 1 atm CO and irradiated under 15W 455 nm blue LEDs for 20 hours. After completion, the reaction mixture was concentrated under reduced pressure. The residue was purified by silica gel column chromatography to afford the desired product in a yield of 79% (116mg, l/b > 20:1) and ee of 97%.

To a ampoule was added Pd_2dba_3 (9.1 mg, 0.010 mmol, 2.5 mol%) and DPEphos (13 mg, 0.024 mmol, 6 mol%), (2S)-2-tert-butoxycarbonylamino-4-pentenoate (92 mg, 0.40 mmol, 1.0 equiv.), benzyl alcohol (216 mg, 2 mmol, 5 equiv.), TsOH·H_2O (7.6 mg, 0.040 mmol, 10 mol%), NaI (3.0 mg, 0.020 mmol, 5 mol%) and dioxane (2.0 mL) were added to a dry glass vessel. The glass vessel was put into an autoclave. Then, the autoclave was purged and charged with CO (30 atm). The reaction mixture was stirred at 120 °C for 24 hours. After the reaction finished, the autoclave was cooled to room temperature and the pressure was carefully released in the fume hood. The reaction mixture was concentrated under reduced pressure. The residue was purified by silica gel column chromatography to afford the desired product in a yield of 36% (31 mg, l/b > 20:1) and ee of 14%.

6-benzyl 1-methyl (*S*)-2-((tert-butoxycarbonyl)amino)hexanedioate (123)

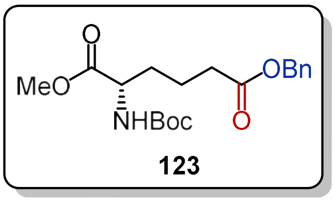

^{1}H NMR (400 MHz, $CDCl_3$) δ 7.40 – 7.27 (m, 5H), 5.15 (d, *J* = 8.5 Hz, 1H), 5.11 (s, 2H), 4.30 (td, *J* = 7.7, 4.8 Hz, 1H), 3.71 (s, 3H), 2.39 (td, *J* = 7.1, 2.9 Hz, 2H), 1.89 – 1.78 (m, 1H), 1.75 – 1.60 (m, 3H), 1.44 (s, 9H). **^{13}C NMR** (101 MHz, $CDCl_3$) δ 173.1, 172.9, 155.4, 135.9, 128.6, 128.25, 128.22, 79.9, 66.3, 53.1, 52.3, 33.6, 32.0, 28.3, 20.8. **HRMS** (ESI) calcd. for $C_{19}H_{27}NO_6Na$ $[M+Na]^+$: 388.1731, found: 388.1743.The ee value was determined to be 97% ee. [Determined by HPLC with a Chiralcel OD-H column (hexane: isopropanol = 90:10, 1 mL/min, 229 nm, 25 °C); t (minor) = 8.9 min, t (major) = 9.3 min]. $[\alpha]_D^{20}$ = 52.7 (CH_2Cl_2, *c* 0.10).

5.4. Synthesize of dihydroavenanthramide D

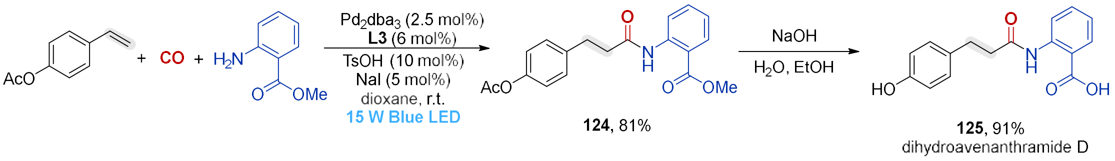

To a flame-dried Young-type tube was added Pd_2dba_3 (9.1 mg, 0.010 mmol, 2.5 mol%) and **L3** (22 mg, 0.024 mmol, 6 mol%). The tube was evacuated and refilled with N_2, then 4-vinylphenyl acetate (65 mg, 0.40 mmol, 1.0 equiv.), methyl 2-aminobenzoate (91 mg, 0.6 mmol, 20 equiv.), TsOH·H_2O (7.6 mg, 0.040 mmol, 10 mol%), NaI (3.0 mg, 0.020 mmol, 5 mol%) and dioxane (2.0 mL) were added under the N_2 atmosphere. After that, the resulting mixture was degassed with the freeze-thaw method, introduced 1 atm CO and irradiated under 15W 455 nm blue LEDs for 24 hours. Two parallel reactions were set up. After completion, the reaction mixture was concentrated under reduced pressure. The residue was purified by silica gel column chromatography to afford the intermediate product **124** in a yield of 81% (226 mg). Subsequently, 5 mL of ethanol and 5 mL of NaOH (10% wt., aq.) were added. After refluxing overnight, the mixture was extracted with ethyl acetate (EA, 5 mL × 3). The solvent was removed under vacuum, and the target product **125** was obtained in 91% yield (188 mg) and l/b > 20:1.

methyl 2-(3-(4-acetoxyphenyl)propanamido)benzoate (124)

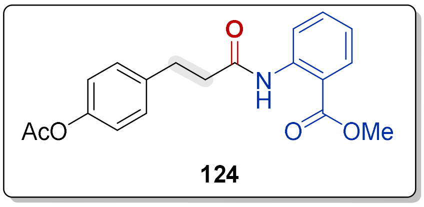

^{1}H NMR (400 MHz, $CDCl_3$) δ 11.09 (s, 1H), 8.72 (dd, J = 8.5, 1.2 Hz, 1H), 8.01 (dd, J = 8.0, 1.6 Hz, 1H), 7.53 (ddd, J = 8.7, 7.2, 1.7 Hz, 1H), 7.29 – 7.24 (m, 2H), 7.06 (ddd, J = 8.4, 7.4, 1.2 Hz, 1H), 7.04 – 6.96 (m, 2H), 3.90 (s, 3H), 3.07 (dd, J = 8.9, 6.8 Hz, 2H), 2.75 (dd, J = 8.6, 7.0 Hz, 2H), 2.27 (s, 3H). **^{13}C NMR** (101 MHz, $CDCl_3$) δ 170.9, 169.6, 168.7, 149.1, 141.5, 138.2, 134.7, 130.8, 129.4, 122.5, 121.6, 120.4, 114.8, 52.3, 40.1, 30.7, 21.1. **HRMS** (ESI) calcd. for $C_{19}H_{20}NO_5$ $[M+H]^+$: 342.1336, found: 342.1345

dihydroavenanthramide D (125)

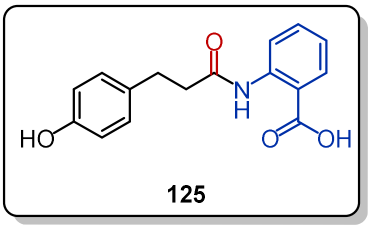

^{1}H NMR (500 MHz, DMSO-d_6) δ 13.58 (s, 1H), 11.12 (d, J = 8.4 Hz, 1H), 9.15 (s, 1H), 8.48 (d, J = 8.4 Hz, 1H), 7.96 (dd, J = 7.9, 1.8 Hz, 1H), 7.57 (td, J = 8.0, 7.5, 1.7 Hz, 1H), 7.13 (t, J = 7.6 Hz, 1H), 7.08 – 6.99 (m, 2H), 6.66 (dq, J = 9.6, 3.2 Hz, 2H), 2.83 (t, J = 7.6 Hz, 2H), 2.63 (t, J = 7.7 Hz, 2H). **^{13}C NMR** (126 MHz, DMSO-d_6) δ 171.1, 170.0, 156.0, 141.3, 134.5, 131.5, 131.2, 129.6, 123.0, 120.4, 116.8, 115.6, 40.2, 30.3. **HRMS** (ESI) calcd. for $C_{16}H_{15}NO_4Na$ $[M+Na]^+$: 308.0893, found: 308.0904

6. Mechanistic investigations

6.1. Synthesis and characteristic of palladium hydride species

6.1.1. Synthesis of palladium hydride complex via reduction of Pd(II)

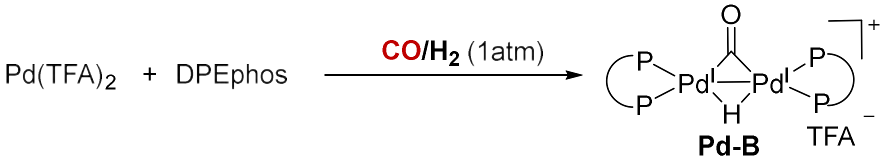

In a nitrogen-filled glovebox, a 25 mL Young-type tube was charged with $Pd(TFA)_2$ (531 mg, 1.6 mmol), DPEphos (947 mg, 1.76 mmol), and CH_2Cl_2 (8 mL). After that, the resulting mixture was degassed with the freeze-thaw method, introduced 1 atm CO/H_2 (1:1) mixture gas and heated to 60 °C for 4 h. Then, the tube was allowed to cool to room temperature and transferred to a glovebox. The reaction mixture was concentrated to ~2 mL under reduced pressure, followed by the addition of hexane (20 mL) to precipitate the complex. The red solid was isolated by filtration, washed with hexane (3×10 mL) and dried under high vacuum. The title compound was obtained in 52% yield (596 mg).

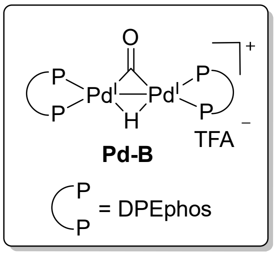

^{1}H NMR (500 MHz, CD_2Cl_2) δ 7.24 – 7.05 (m, 28H), 6.98 (t, J = 7.6 Hz, 16H), 6.85 – 6.76 (m, 8H), 6.51 – 6.40 (m, 4H), -6.93 (p, J = 42.2 Hz, 1H). **^{13}C NMR** (126 MHz, CD_2Cl_2) δ 224.6 (t, J = 34.5 Hz), 157.9 (t, J = 4.4 Hz), 134.3, 134.1, 132.2, 130.6 (m), 130.5, 128.6, 125.0, 123.6 (m), 120.4. **^{31}P NMR** (202 MHz, CD_2Cl_2) δ 9.6. **^{19}F NMR** (471 MHz, CD_2Cl_2) δ -76.2. **HRMS** (ESI) calcd. for $C_{73}H_{57}O_3P_4Pd_2$ $[M]^+$: 1317.1328, found: 1317.1355

6.1.2. Synthesis of palladium hydride complex via oxidative addition of Pd(0)

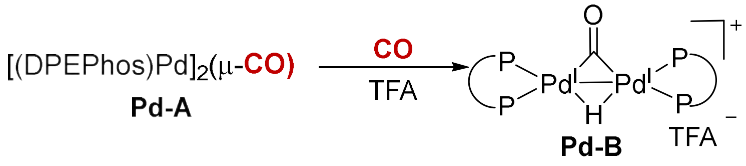

$[(DPEphos)Pd]_2(\mu\text{-}CO)$ was synthesized according to the method recorded in the literature.[17] Weigh 70 mg (0.05 mmol, 1 equiv.) of $[(DPEphos)Pd]_2(\mu\text{-}CO)$ and 5.7 mg (0.05 mmol, 1equiv.) of TFA into an NMR tube, add 0.75 mL of CD_2Cl_2, and then test by ^{1}H, ^{31}P and ^{19}F NMR. Characteristic chemical shifts attributable to the palladium hydride species were identified, tentatively assigned as **Pd-B**.

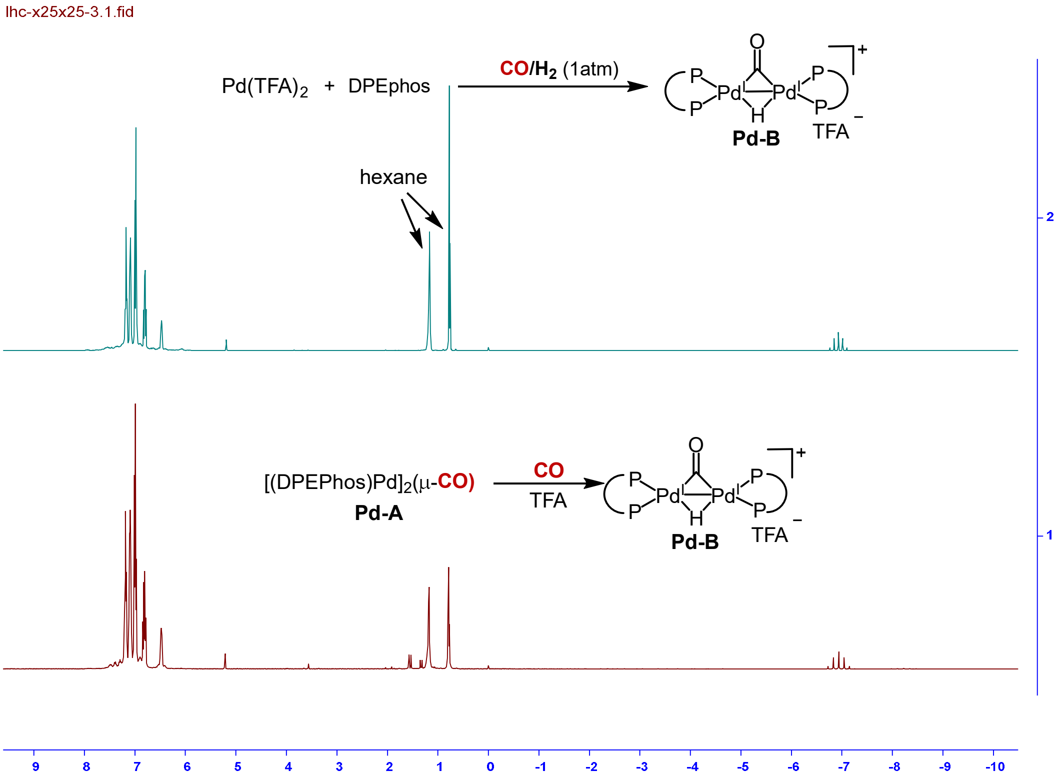

Figure S3. ^{1}H NMR of **Pd-B** prepared via two pathways.

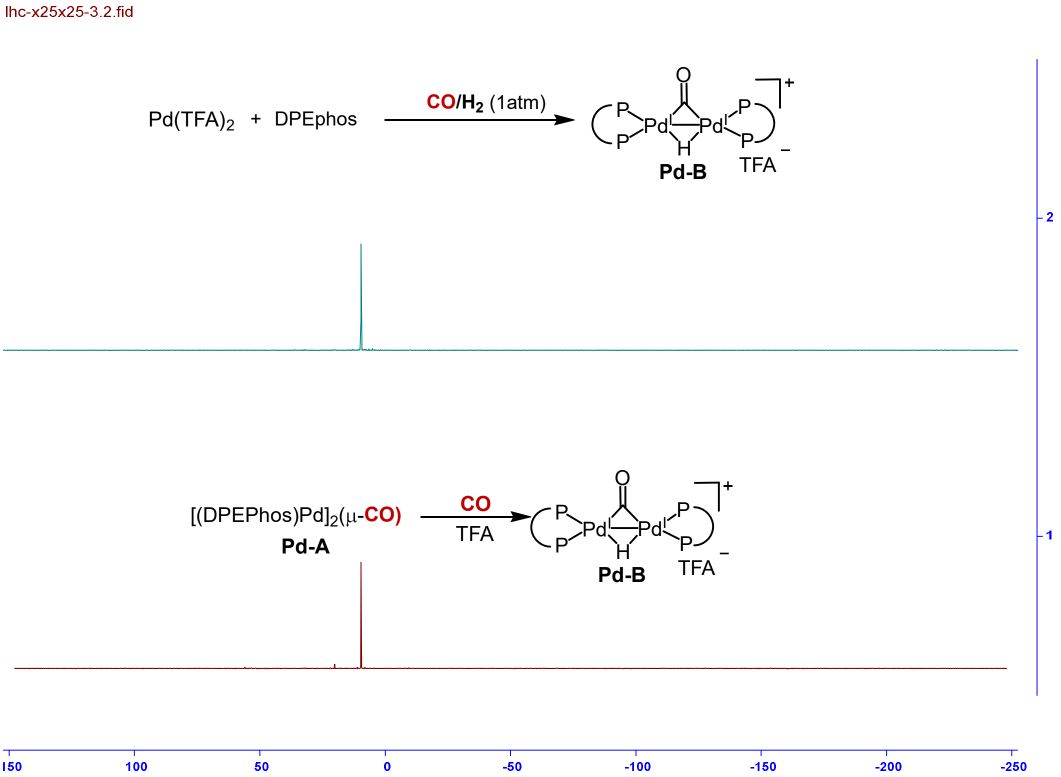

Figure S4. ^{31}P NMR of **Pd-B** prepared via two pathways.

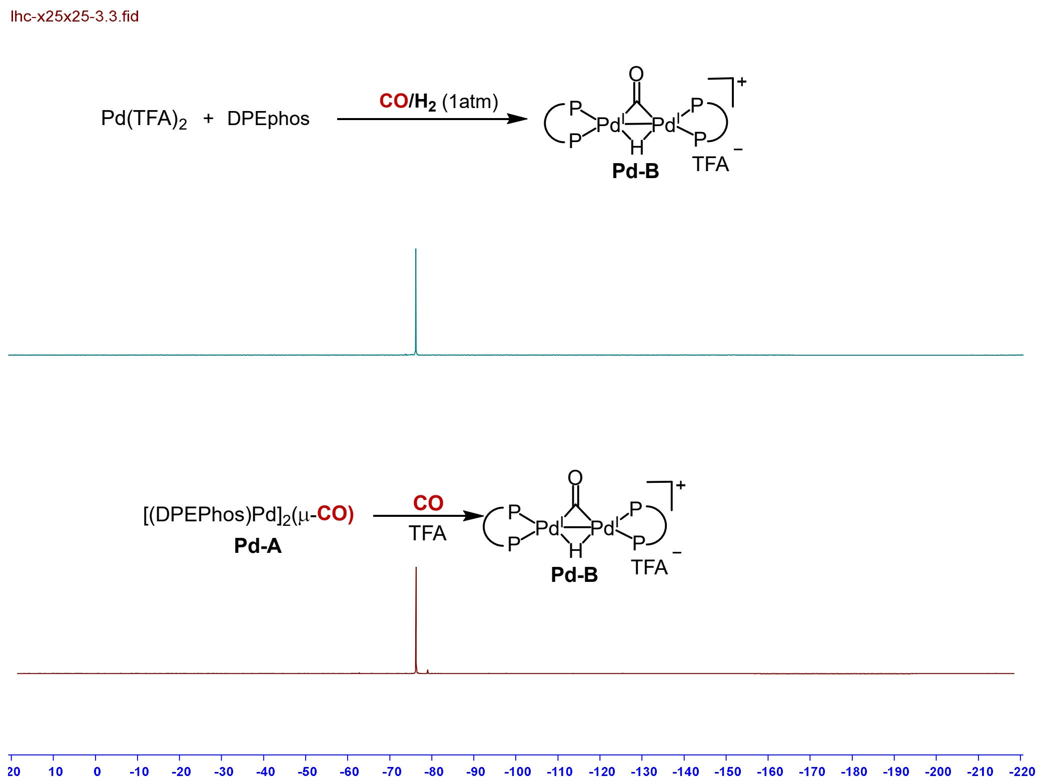

Figure S5. ^{19}F NMR of **Pd-B** prepared via two pathways.

6.2. UV-Vis absorption spectroscopy studies

Six portions of 0.2 mmol [(DPEphos)Pd]$_2$(μ-CO) were each dissolved in 2 mL of dioxane. To one portion was added 0.2 mmol TsOH, and to other portions was added 0.2 mmol TsOH and NaX. For each solution, 20 μL was transferred to a cuvette, diluted to 2 mL, and purged with CO for 1 minute. Separately, 0.2 mmol DPEphos, TsOH, and NaI were dissolved in 2 mL of dioxane, and 20 μL of this solution was transferred to a cuvette and diluted to 2 mL. UV-Vis absorption spectroscopy studies using a Shimadzu UV3600 I plus UV-Vis-NIR spectrophotometer from 300 nm to 600 nm.

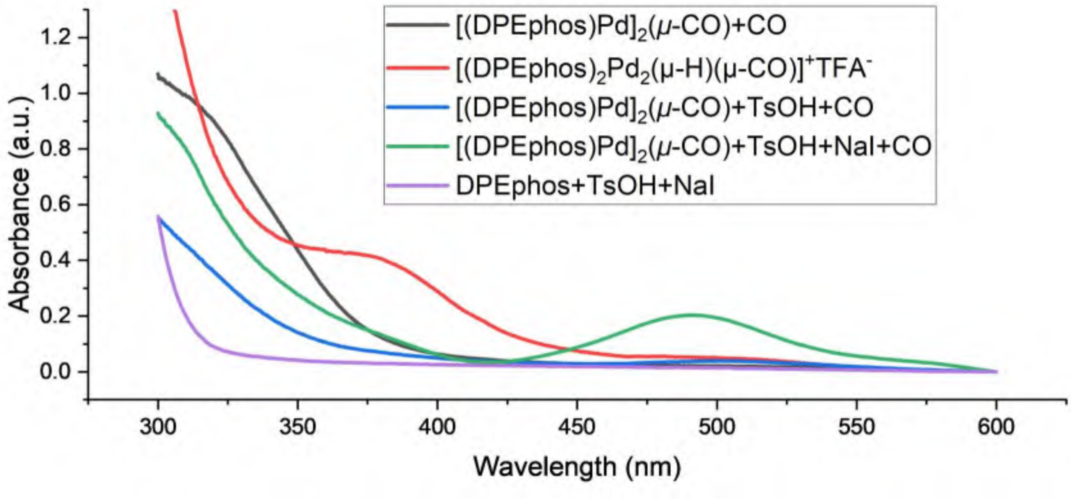

Figure S6. UV-Vis absorption spectroscopy in dioxane.

6.3. Radical trapping experiment

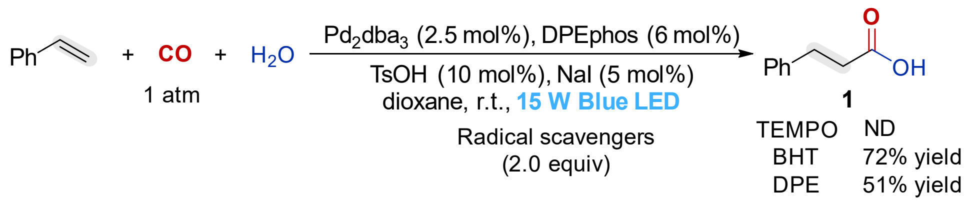

To a flame-dried Young-type tube was added Pd$_2$dba$_3$ (9.1 mg, 0.010 mmol, 2.5 mol%) and DPEphos (13 mg, 0.024 mmol, 6 mol%). The tube was evacuated and refilled with N$_2$, then styrene (42 mg, 0.40 mmol, 1.0 equiv.), H$_2$O (144 mg, 8 mmol, 20 equiv.), TsOH·H$_2$O (7.6 mg, 0.040 mmol, 10 mol%), NaI (3.0 mg, 0.020 mmol, 5 mol%), radical scavengers (2.0 equiv) and dioxane (2.0 mL) were added under the N$_2$ atmosphere. After that, the resulting mixture was degassed with the freeze-thaw method, introduced 1 atm CO and irradiated under 15W 455 nm blue LEDs for 20 hours. After completion, using n-tetradecane as an internal standard, gas chromatography analysis showed yieldes of 0% (TEMPO), 72%(BHT) and 51%(DPE).

6.4. Radical clock experiment

6.4.1. Ring-closing experiment

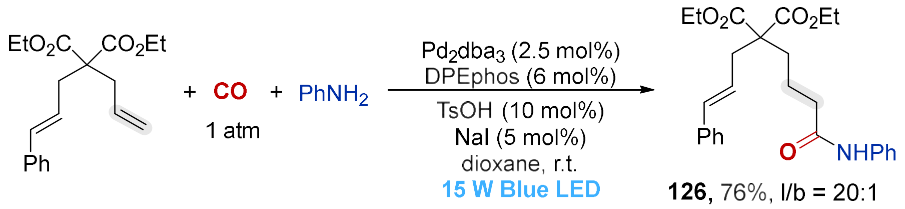

To a flame-dried Young-type tube was added Pd_2dba_3 (9.1 mg, 0.010 mmol, 2.5 mol%) and DPEphos (13 mg, 0.024 mmol, 6 mol%). The tube was evacuated and refilled with N_2, then diethyl 2-allyl-2-cinnamylmalonate (126 mg, 0.40 mmol, 1.0 equiv.), amiline (56 mg, 0.60 mmol, 1.5 equiv.), $TsOH·H_2O$ (7.6 mg, 0.040 mmol, 10 mol%), NaI (3.0 mg, 0.020 mmol, 5 mol%) and dioxane (2.0 mL) were added under the N_2 atmosphere. After that, the resulting mixture was degassed with the freeze-thaw method, introduced 1 atm CO and irradiated under 15W 455 nm blue LEDs for 20 hours. After completion, the reaction mixture was concentrated under reduced pressure. The residue was purified by silica gel column chromatography to afford the desired product **102** in a 76% yield (133 mg).

diethyl 2-cinnamyl-2-(4-oxo-4-(phenylamino)butyl)malonate (126)

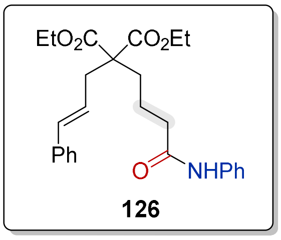

^{1}H NMR (500 MHz, $CDCl_3$) δ 7.58 (s, 1H), 7.55 – 7.48 (m, 2H), 7.31 – 7.25 (m, 6H), 7.23 – 7.17 (m, 1H), 7.08 (t, J = 7.4 Hz, 1H), 6.45 (d, J = 15.7 Hz, 1H), 6.05 (dt, J = 15.4, 7.6 Hz, 1H), 4.20 (qd, J = 7.2, 3.0 Hz, 4H), 2.83 (dd, J = 7.6, 1.3 Hz, 2H), 2.36 (t, J = 7.2 Hz, 2H), 2.02 – 1.90 (m, 2H), 1.72 (qd, J = 7.4, 4.5 Hz, 2H), 1.25 (t, J = 7.1 Hz, 6H). **^{13}C NMR** (126 MHz, $CDCl_3$) δ 171.2, 170.5, 138.0, 137.0, 134.0, 129.0, 128.5, 127.4, 126.3, 124.2, 123.9, 119.9, 61.5, 57.7, 37.4, 36.6, 32.2, 20.1, 14.20, 14.18. **HRMS** (ESI) calcd. for $C_{26}H_{32}NO_5$ $[M+H]^+$: 438.2275, found: 438.2293

6.4.2. Ring-opening experiment

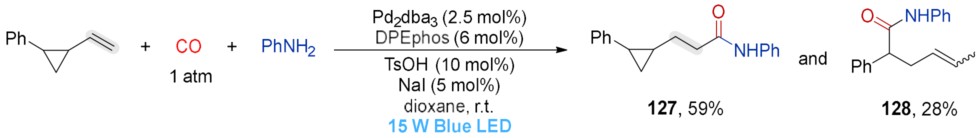

To a flame-dried Young-type tube was added Pd_2dba_3 (9.1 mg, 0.010 mmol, 2.5 mol%) and DPEphos (13 mg, 0.024 mmol, 6 mol%). The tube was evacuated and refilled with N_2, then (2-vinylcyclopropyl)benzene (58 mg, 0.40 mmol, 1.0 equiv.), amiline (56 mg, 0.60 mmol, 1.5 equiv.), $TsOH·H_2O$ (7.6 mg, 0.040 mmol, 10 mol%), NaI (3.0 mg, 0.020 mmol, 5 mol%) and dioxane (2.0 mL) were added under the N_2 atmosphere. After that, the resulting mixture was degassed with the freeze-thaw method, introduced 1 atm CO and irradiated under 15W 455 nm blue LEDs for 20 hours. After completion, the reaction mixture was concentrated under reduced pressure. The residue

was purified by silica gel column chromatography to afford the desired product **127** and **128** in 59% (63 mg) and 28% (30 mg) yield respectively.

diethyl 2-cinnamyl-2-(4-oxo-4-(phenylamino)butyl)malonate (127)

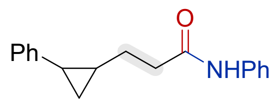

127

^{1}H NMR (500 MHz, $CDCl_3$) δ 7.93 (s, 1H), 7.48 – 7.41 (m, 2H), 7.26 – 7.16 (m, 4H), 7.12 – 7.07 (m, 1H), 7.07 – 7.02 (m, 1H), 6.99 – 6.93 (m, 2H), 2.43 (t, *J* = 7.5 Hz, 2H), 1.75 (ddt, *J* = 23.2, 14.0, 6.9 Hz, 2H), 1.63 (tt, *J* = 9.2, 5.0 Hz, 1H), 1.07 – 0.97 (m, 1H), 0.87 (dt, *J* = 8.4, 4.9 Hz, 1H), 0.76 (dt, *J* = 8.7, 5.2 Hz, 1H). **^{13}C NMR** (126 MHz, $CDCl_3$) δ 171.7, 143.4, 138.1, 129.0, 128.4, 125.6, 125.5, 124.3, 120.2, 37.5, 30.4, 23.2, 16.2. **HRMS** (ESI) calcd. for $C_{18}H_{19}NONa$ $[M+Na]^+$: 288.1359, found: 288.1364

diethyl 2-cinnamyl-2-(4-oxo-4-(phenylamino)butyl)malonate (128)

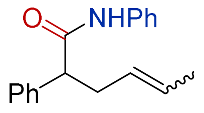

128

^{1}H NMR (500 MHz, $CDCl_3$) δ 7.36 (dd, *J* = 15.2, 8.0 Hz, 2H), 7.29 (q, *J* = 7.0 Hz, 3H), 7.26 – 7.18 (m, 4H), 7.09 – 6.98 (m, 1H), 5.78 – 5.46 (m, 2H), 4.34 – 3.79 (m, 1H), 2.81 – 2.42 (m, 2H), 1.74 – 1.60 (m, 3H). **^{13}C NMR** (126 MHz, $CDCl_3$) δ 169.8, 169.6, 143.9, 143.5, 137.8, 133.1, 132.4, 129.0, 128.94, 128.92, 128.84, 128.79, 127.5, 127.2, 126.7, 126.6, 126.2, 125.8, 125.6, 124.32, 124.29, 120.12, 120.05, 45.3, 45.2, 44.5, 40.0, 18.0, 13.2. **HRMS** (ESI) calcd. for $C_{18}H_{20}NO$ $[M+H]^+$: 266.1539, found: 266.1541

6.5. Isotopic-labelling experiment

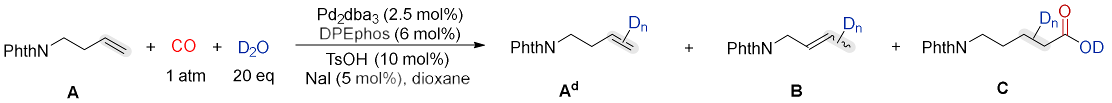

To a flame-dried Young-type tube was added Pd_2dba_3 (4.5 mg, 0.005 mmol, 2.5 mol%) and DPEphos (6.5 mg, 0.012 mmol, 6 mol%). The tube was evacuated and refilled with N_2, then 2-(but-3-en-1-yl)isoindoline-1,3-dione (40 mg, 0.20 mmol, 1.0 equiv.), D_2O (80 mg, 4 mmol, 20 equiv.), $TsOH \cdot H_2O$ (3.8 mg, 0.020 mmol, 10 mol%), NaI (1.5 mg, 0.010 mmol, 5 mol%) and dioxane (1.0 mL) were added under the N_2 atmosphere. After that, the resulting mixture was degassed with the freeze-thaw method, introduced 1 atm CO and stirred under r.t./80 °C/100 °C/blue LEDs for 20 hours. After completion, the reaction mixture was concentrated under reduced pressure and analyzed by ^1H NMR.

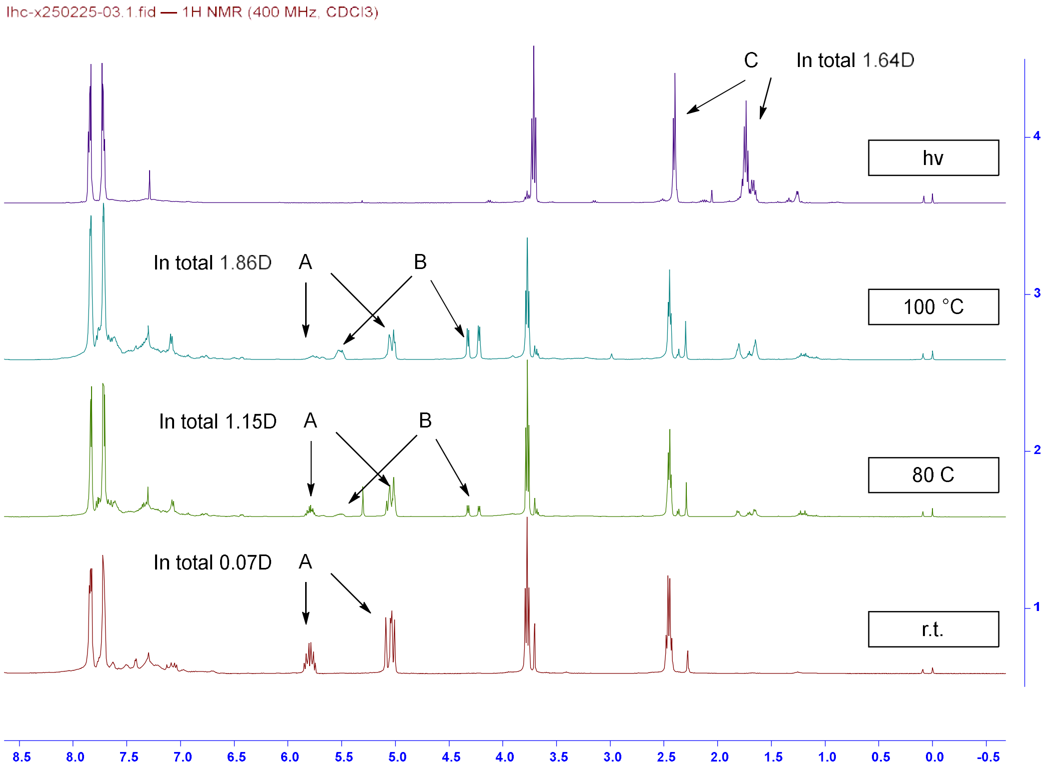

Figure S7. Isotopic-labelling experiment with 1 atm CO.

To a flame-dried Young-type tube was added Pd_2dba_3 (4.5 mg, 0.005 mmol, 2.5 mol%) and DPEphos (6.5 mg, 0.012 mmol, 6 mol%). The tube was evacuated and refilled with N_2, then 2-(but-3-en-1-yl)isoindoline-1,3-dione (40 mg, 0.20 mmol, 1.0 equiv.), D_2O (80 mg, 4 mmol, 20 equiv.), $TsOH \cdot H_2O$ (3.8 mg, 0.020 mmol, 10 mol%), NaI (1.5 mg, 0.010 mmol, 5 mol%) and dioxane (1.0 mL) were added under the N_2 atmosphere. After that, the resulting mixture was degassed with the freeze-thaw method, then stirred under r.t./80 °C/100 °C/ blue LEDs for 20 hours. After completion, the reaction mixture was concentrated under reduced pressure and analyzed by ^1H NMR.

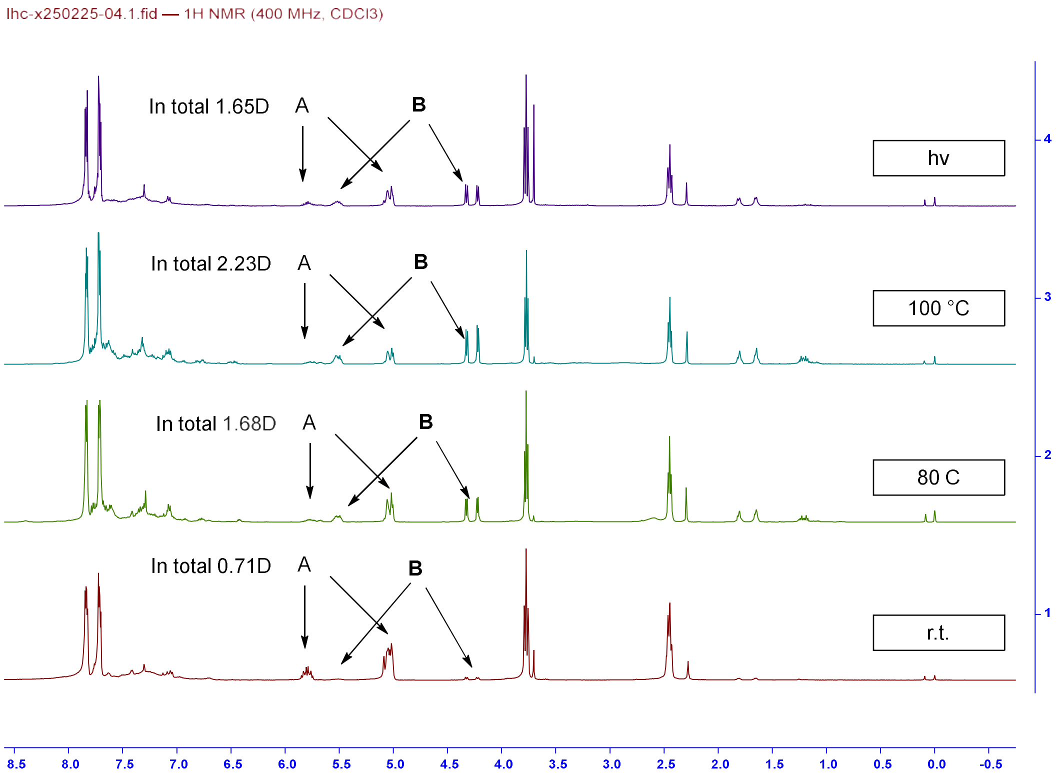

Figure S8. Isotopic-labelling experiment without CO.

6.6 Stoichiometric experiment of the palladium-hydride species

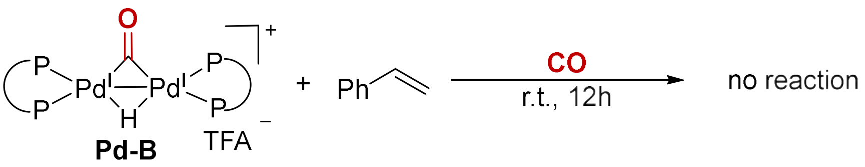

In a nitrogen-filled glovebox, a 25 mL Young-type tube was charged with **Pd-B** (72 mg, 0.05 mmol, 1.0 equiv.), styrene (10.4 mg, 2.0 equiv.) and $CDCl_3$ (0.5 mL). After that, the resulting mixture was degassed with the freeze-thaw method, introduced 1 atm CO and stirred under under r.t. for 12 hours. After completion, the reaction mixture analyzed by ^{1}H NMR using 1,3,5-trimethoxybenzene as internal standard. Both **Pd–B** and styrene were unreacted

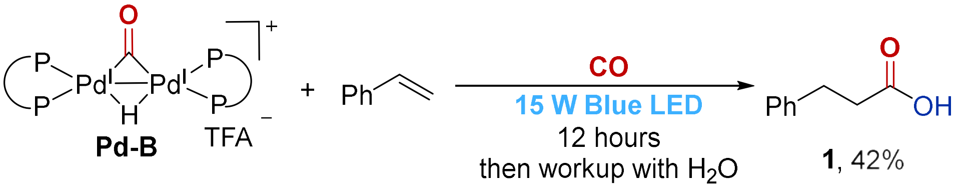

In a nitrogen-filled glovebox, a 25 mL Young-type tube was charged with **Pd-B** (72 mg, 0.05 mmol, 1.0 equiv.), styrene (10.4 mg, 2.0 equiv.) and $CDCl_3$ (0.5 mL). After that, the resulting mixture was degassed with the freeze-thaw method, introduced 1 atm CO and stirred under 455 nm blue LEDs for 12 hours. Then H_2O (18 mg, 20 equiv.) was added and the reaction mixture was stirred under under 60 °C for 1 hour. After completion, the reaction mixture analyzed by ^{1}H NMR and GC using 1,3,5-trimethoxybenzene as internal standard. Product **1** was detected in 42% yield by GC.

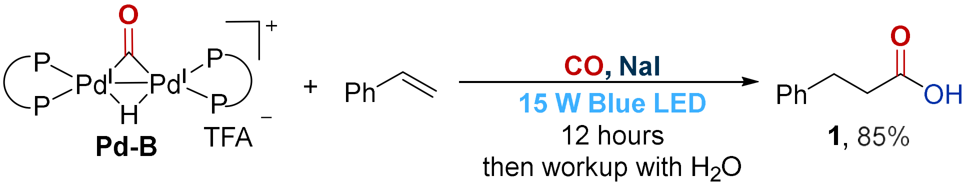

In a nitrogen-filled glovebox, a 25 mL Young-type tube was charged with **Pd-B** (72 mg, 0.05 mmol, 1.0 equiv.), styrene (10.4 mg, 2.0 equiv.), NaI (14.9 mg, 2.0 equiv.) and $CDCl_3$ (0.5 mL). After that, the resulting mixture was degassed with the freeze-thaw method, introduced 1 atm CO and stirred under under 455 nm blue LEDs for 12 hours. Then H_2O (18 mg, 20 equiv.) was added and the reaction mixture was stirred under under 60 °C for 1 hour. After completion, the reaction mixture analyzed by ^{1}H NMR and GC using 1,3,5-trimethoxybenzene as internal standard. Product **1** was detected in 85% yield by GC.

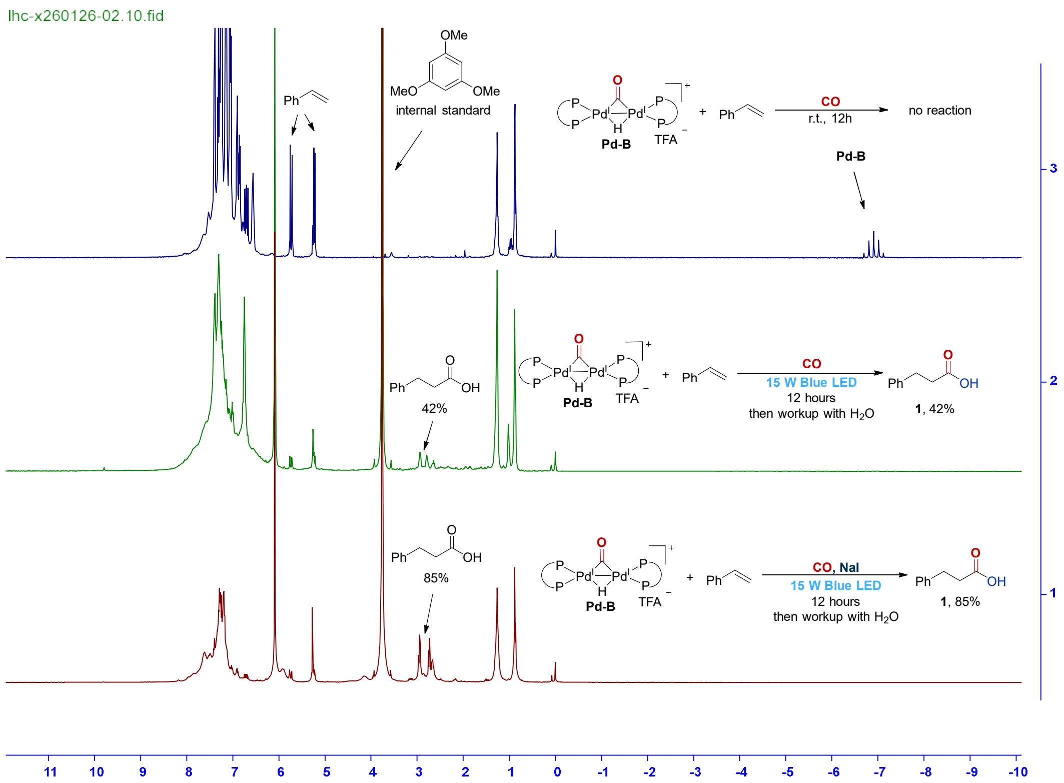

Figure S9. Stoichiometric experiment of the palladium-hydride species.

6.7 Hydrolysis of acylpalladium complex

6.7.1. Synthesis of acylpalladium complex

To a flame-dried Young-type tube was added $Pd(PPh_3)_4$ (1.16g, 1.0 mmol, 1.0 equiv.) and DPEphos (1.07 g, 2.0 mmol, 2.0 equiv.). The tube was evacuated and refilled with N_2, then hydrocinnamoyl chloride (337 mg, 2.0 mmol, 2.0 equiv.) and toluene (4.0 mL) were added under the N_2 atmosphere. After that, the resulting mixture was stirred under r.t. for 12 hours. After completion, 20 mL diethyl ether was added and stirred for 2h and remove the solvent by decantation. The solid was washed with diethyl ether (5 mL × 3) and concentrated under reduced pressure to afford the desired **(DPEphos)Pd(COCH$_2$CH$_2$Ph)Cl (Pd-C)** in a 40% yield (325 mg).

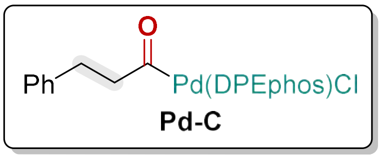

^{1}H NMR (400 MHz, $CDCl_3$) δ 7.51 (t, J = 9.1 Hz, 8H), 7.32 (dt, J = 15.2, 7.1 Hz, 12H), 7.21 (s, 2H), 7.17 – 7.12 (m, 2H), 7.11 – 7.05 (m, 1H), 6.97 – 6.59 (m, 8H), 2.85 (s, 2H), 2.36 (s, 2H). **^{13}C NMR** (101 MHz, $CDCl_3$) δ 231.86 (d, J = 58.9 Hz), 158.21 (d, J = 9.0 Hz), 141.8, 134.7, 134.6, 134.2, 131.7, 131.0, 130.2, 128.4, 128.35, 128.25, 128.1, 125.5, 124.5, 53.30 (t, J = 25.5 Hz), 31.8. **^{31}P NMR** (202 MHz, $CDCl_3$) δ 10.08, 2.72.

6.7.2. Hydrolysis of acylpalladium complex

To a flame-dried Young-type tube was added **Pd-C** (16 mg, 0.010 mmol, 1.0 equiv.). The tube was evacuated and refilled with N_2, then dioxane (1.0 mL) were added under the N_2 atmosphere. After that, the resulting mixture was degassed with the freeze-thaw method, then stirred under r.t. for 20 hours. After completion, the reaction mixture was concentrated under reduced pressure and analyzed by ^{1}H NMR using 1,3,5-trimethoxybenzene as internal standard. Most of **Pd-C** is unreacted.

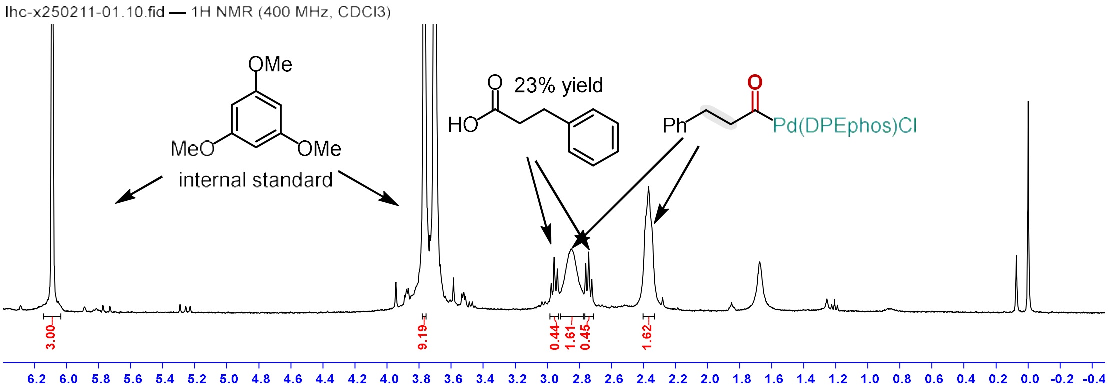

Figure S10. Hydrolysis of acylpalladium complex

To a flame-dried Young-type tube was added **Pd-C** (16 mg, 0.010 mmol, 1.0 equiv.) and TsOAg/NaI (0.020 mmol, 2.0 equiv.). The tube was evacuated and refilled with N_2, then dioxane (1.0 mL) were added under the N_2 atmosphere. After that, the resulting mixture was degassed with the freeze-thaw method, then stirred under r.t./80 °C/455 nm blue LEDs for 20 hours. After completion, the reaction mixture was concentrated

under reduced pressure and analyzed by ^{1}H NMR using 1,3,5-trimethoxybenzene as internal standard.

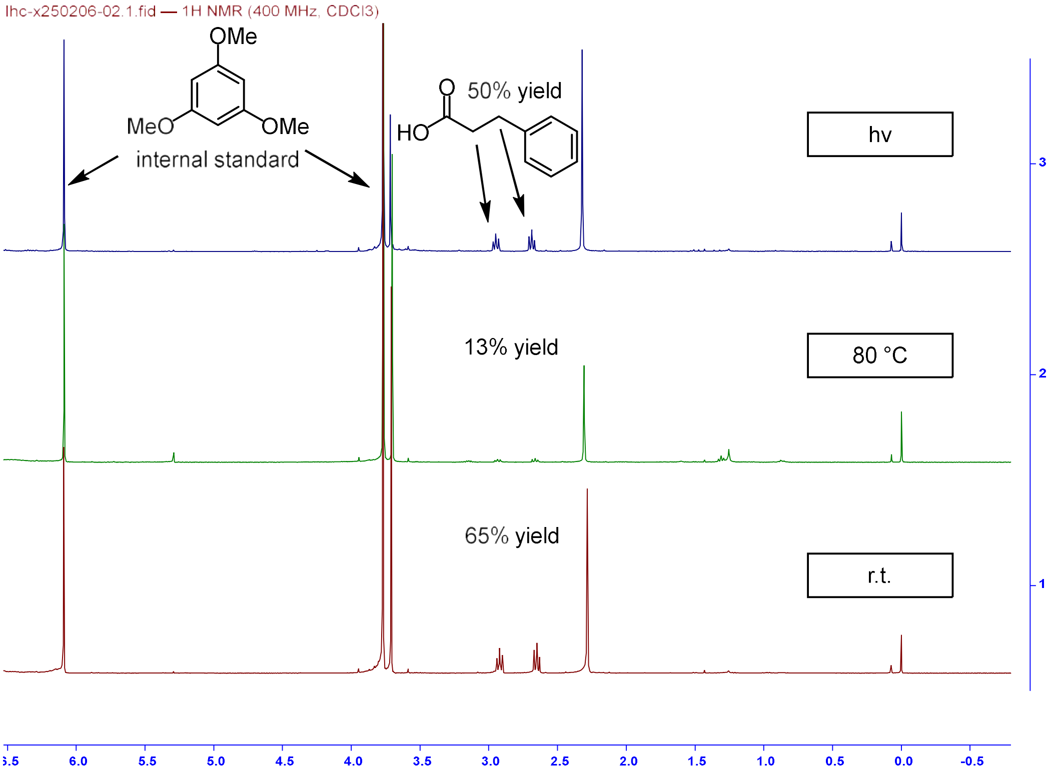

Figure S11. Hydrolysis of acylpalladium complex with TsOAg.

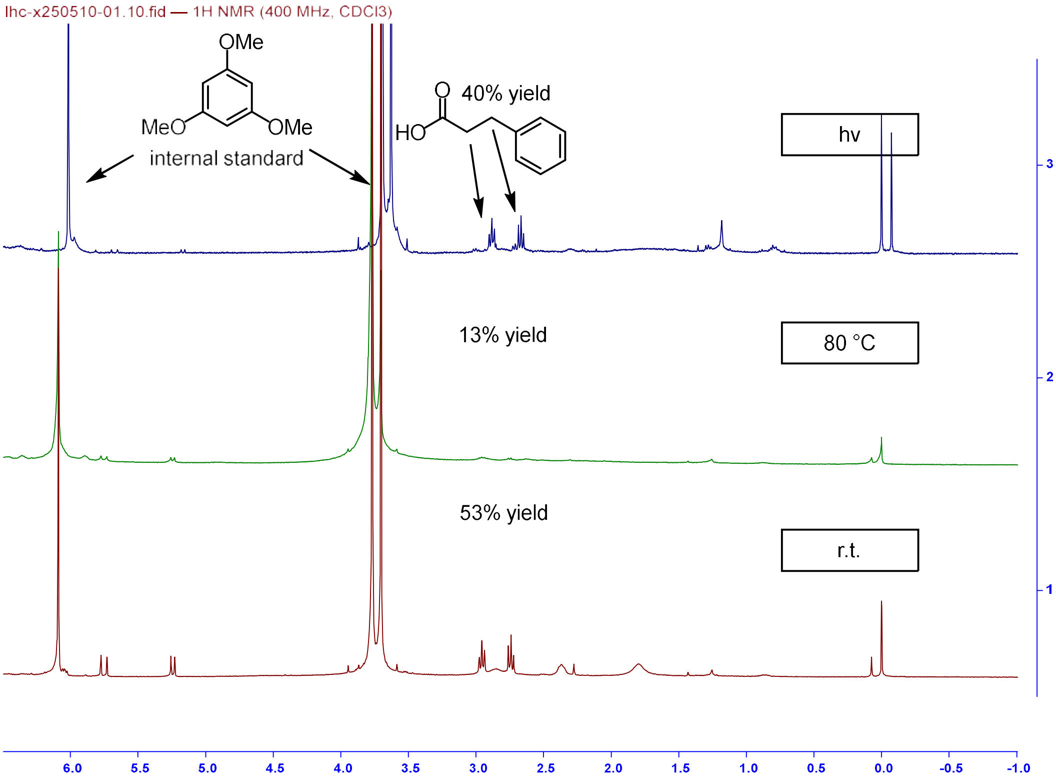

Figure S12. Hydrolysis of acylpalladium complex with NaI.

7. X-ray crystallographic data

7.1. X-ray crystallographic data of 57

A colorless crystal of acylpalladium specie **57** suitable for X-ray analysis was obtained by slow evaporation of CH_2Cl_2 and petroleum ether solution of **56.** The diffraction data were collected on a Rigaku SuperNova diffractometer equipped with an Atlas S2 CCD detector with Cu Kα radiation. The crystal was kept at 300 K during data collection. Details of crystal and data collection parameters are summarized in **Table S12**. Using Olex2, the structure was solved with the SHELXT structure solution program using Intrinsic Phasing and refined with the ShelXL refinement package using Least Squares. As the product is derived from a commercial chiral precursor estrone, the absolute configuration was deemed unnecessary for this study. Friedel pairs were processed without merging, and the Flack parameter was omitted during refinement. A structure drawing of **57** is shown in **Figure S13**.

Table S12. Summary of crystallographic data of **57**

Identification code	LHC-250411-1_auto
Empirical formula	$C_{21}H_{28}O_3$
Formula weight	328.43
Temperature/K	300
Crystal system	monoclinic
Space group	C2
a/Å	12.5080(9)
b/Å	6.8499(6)
c/Å	21.8066(18)
α/°	90
β/°	103.041(8)
γ/°	90
Volume/Å^3	1820.2(3)
Z	4
ρ_{calc}g/cm^3	1.199
μ/mm^{-1}	0.619
F(000)	712.0
Crystal size/mm^3	0.2 × 0.15 × 0.1
Radiation	Cu Kα (λ = 1.54184)
2Θ range for data collection/°	8.324 to 144.932
Index ranges	$-15 \leq h \leq 13, -8 \leq k \leq 7, -26 \leq l \leq 21$
Reflections collected	3371
Independent reflections	2540 [R_{int} = 0.0301, R_{sigma} = 0.0368]
Data/restraints/parameters	2540/1/222
Goodness-of-fit on F^2	1.053
Final R indexes [I>=2σ (I)]	R_1 = 0.0572, wR_2 = 0.1516

Final R indexes [all data]	$R_1 = 0.0641$, $wR_2 = 0.1629$
Largest diff. peak/hole / e Å^{-3}	0.29/-0.24
Flack parameter	0.4(4)

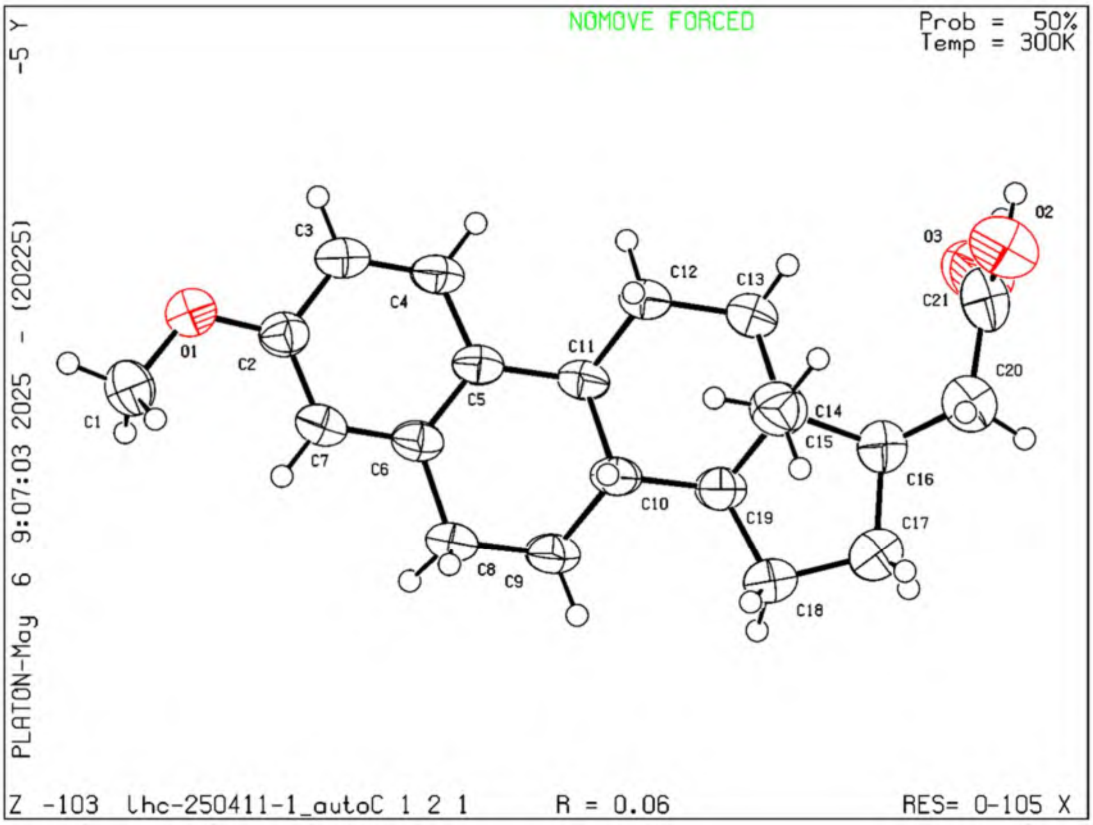

Figure S13. Structure drawing of **57**. Thermal ellipsoids are drawn at the 50% probability level.

7.2. X-ray crystallographic data of 118

A colorless crystal of acylpalladium specie **118** suitable for X-ray analysis was obtained by slow evaporation of CH_2Cl_2 and petroleum ether solution of **118.** The diffraction data were collected on a Rigaku SuperNova diffractometer equipped with an Atlas S2 CCD detector with Cu Kα radiation. The crystal was kept at 300 K during data collection. Details of crystal and data collection parameters are summarized in **Table S13**. Using Olex2, the structure was solved with the SHELXT structure solution program using Intrinsic Phasing and refined with the ShelXL refinement package using Least Squares. A structure drawing of **118** is shown in **Figure S14**.

Table S13. Summary of crystallographic data of **118**

Identification code	LHC-250812_auto
Empirical formula	$C_{21}H_{28}O_2$
Formula weight	312.43
Temperature/K	293(2)
Crystal system	monoclinic
Space group	$P2_1$
a/Å	11.8106(2)
b/Å	6.29650(10)
c/Å	12.5546(2)
α/°	90
β/°	110.576(2)
γ/°	90
Volume/Å^3	874.07(3)
Z	2
ρ_{calc}g/cm^3	1.187
μ/mm^{-1}	0.575
F(000)	340.0
Crystal size/mm^3	0.2 × 0.15 × 0.1
Radiation	Cu Kα (λ = 1.54184)
2Θ range for data collection/°	7.522 to 145.866
Index ranges	-14 ≤ h ≤ 13, -7 ≤ k ≤ 6, -15 ≤ l ≤ 15
Reflections collected	15779
Independent reflections	3248 [R_{int} = 0.0291, R_{sigma} = 0.0199]
Data/restraints/parameters	3248/1/211
Goodness-of-fit on F^2	1.084
Final R indexes [I>=2σ (I)]	R_1 = 0.0440, wR_2 = 0.1221
Final R indexes [all data]	R_1 = 0.0460, wR_2 = 0.1249
Largest diff. peak/hole / e Å^{-3}	0.17/-0.15
Flack parameter	0.09(17)

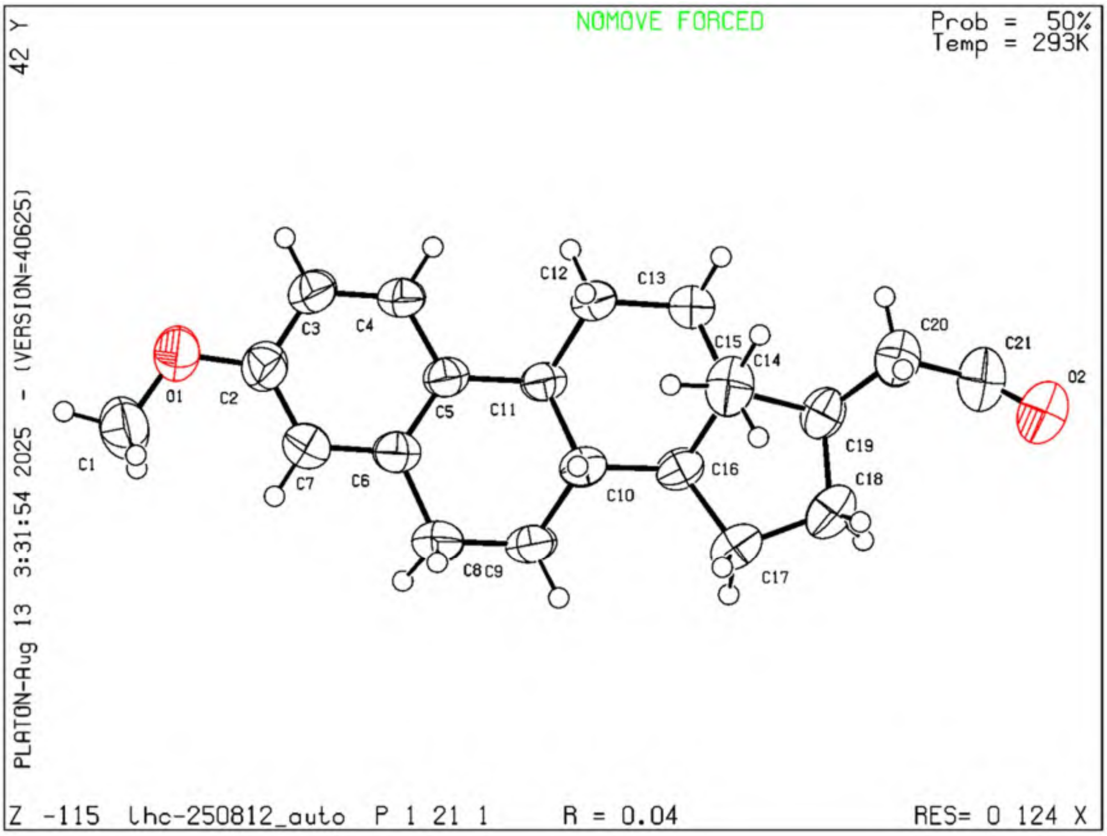

Figure S14. Structure drawing of **118**. Thermal ellipsoids are drawn at the 50% probability level.

7.3. X-ray crystal structure of (DPEphos)Pd($COCH_2CH_2Ph$)Cl

A colorless crystal of acylpalladium species (DPEphos)Pd($COCH_2CH_2Ph$)Cl (**Pd-C**) suitable for X-ray analysis was obtained by slow evaporation of CH_2Cl_2 and petroleum ether solution of acylpalladium specie **Pd-C.** The diffraction data were collected on a Rigaku SuperNova diffractometer equipped with an Atlas S2 CCD detector with Cu Kα radiation. The crystal was kept at 300 K during data collection. Details of crystal and data collection parameters are summarized in **Table S14**. Using Olex2, the structure was solved with the SHELXT structure solution program using Intrinsic Phasing and refined with the ShelXL refinement package using Least Squares. A structure drawing of **5** is shown in **Figure S15**.

Table S14. Summary of crystallographic data of **Pd-C**

Identification code	LHC-250222_auto
Empirical formula	$C_{90}H_{74}Cl_2O_4P_4Pd_2$
Formula weight	1627.07
Temperature/K	300
Crystal system	monoclinic
Space group	Pc
a/Å	12.9483(2)
b/Å	19.1616(2)
c/Å	15.8606(2)
α/°	90
β/°	98.8980(10)
γ/°	90
Volume/Å^3	3887.82(9)
Z	2
ρ_{calc}g/cm^3	1.390
μ/mm^{-1}	5.543
F(000)	1664.0
Crystal size/mm^3	0.2 × 0.15 × 0.1
Radiation	Cu Kα (λ = 1.54184)
2Θ range for data collection/°	7.288 to 145.93
Index ranges	$-15 \leqslant h \leqslant 7, -22 \leqslant k \leqslant 23, -19 \leqslant l \leqslant 19$
Reflections collected	16063
Independent reflections	8850 [R_{int} = 0.0421, R_{sigma} = 0.0589]
Data/restraints/parameters	8850/14/919
Goodness-of-fit on F^2	1.026
Final R indexes [I>=2σ (I)]	R_1 = 0.0420, wR_2 = 0.0990
Final R indexes [all data]	R_1 = 0.0493, wR_2 = 0.1060

Largest diff. peak/hole / e Å^{-3}	0.58/-0.93
Flack parameter	-0.008(8)

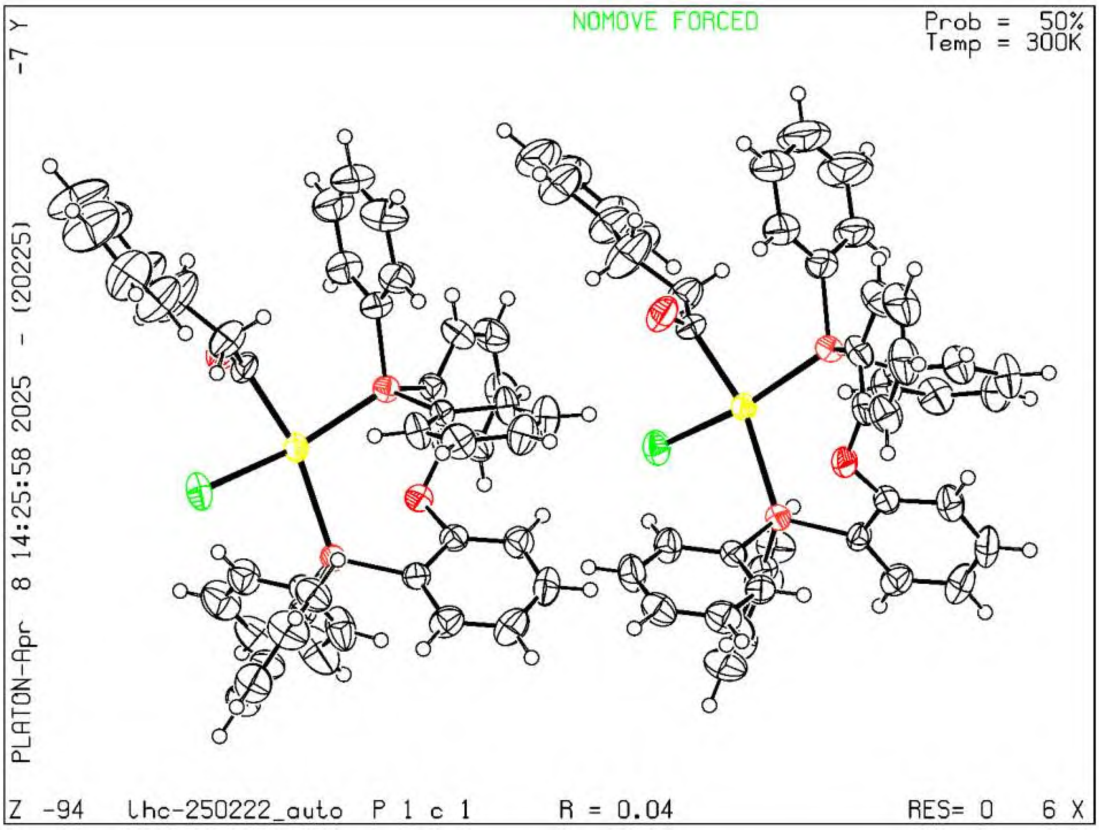

Figure S15. Structure drawing of **Pd-C**. Thermal ellipsoids are drawn at the 50% probability level.

8. NMR spectra

^{1}H NMR spectra for L2

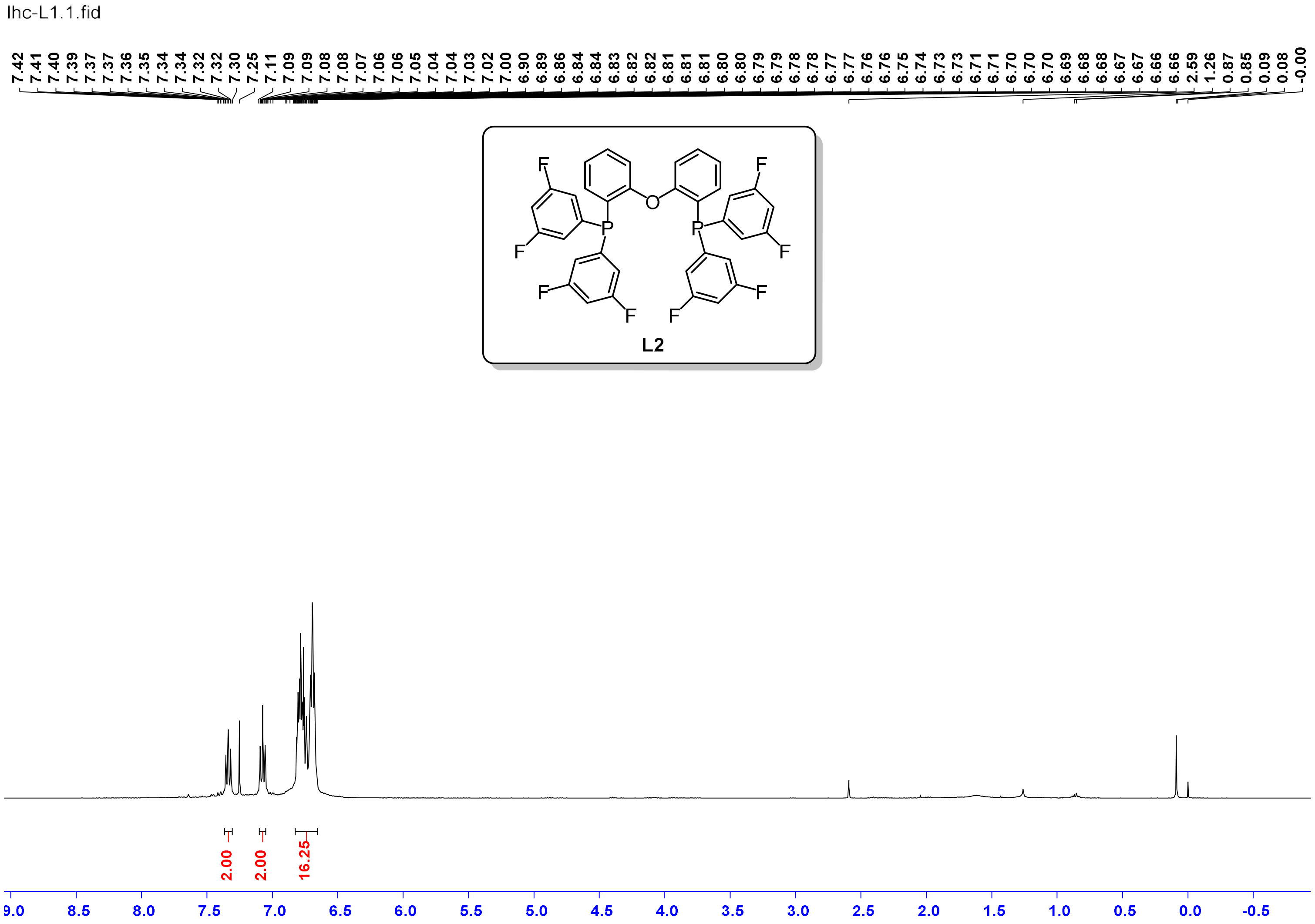

^{13}C NMR spectra for L2

lhc-x250526-1.2.fid — 1H NMR (400 MHz, CDCl3)

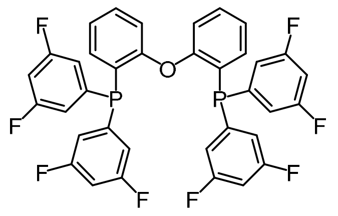

L2

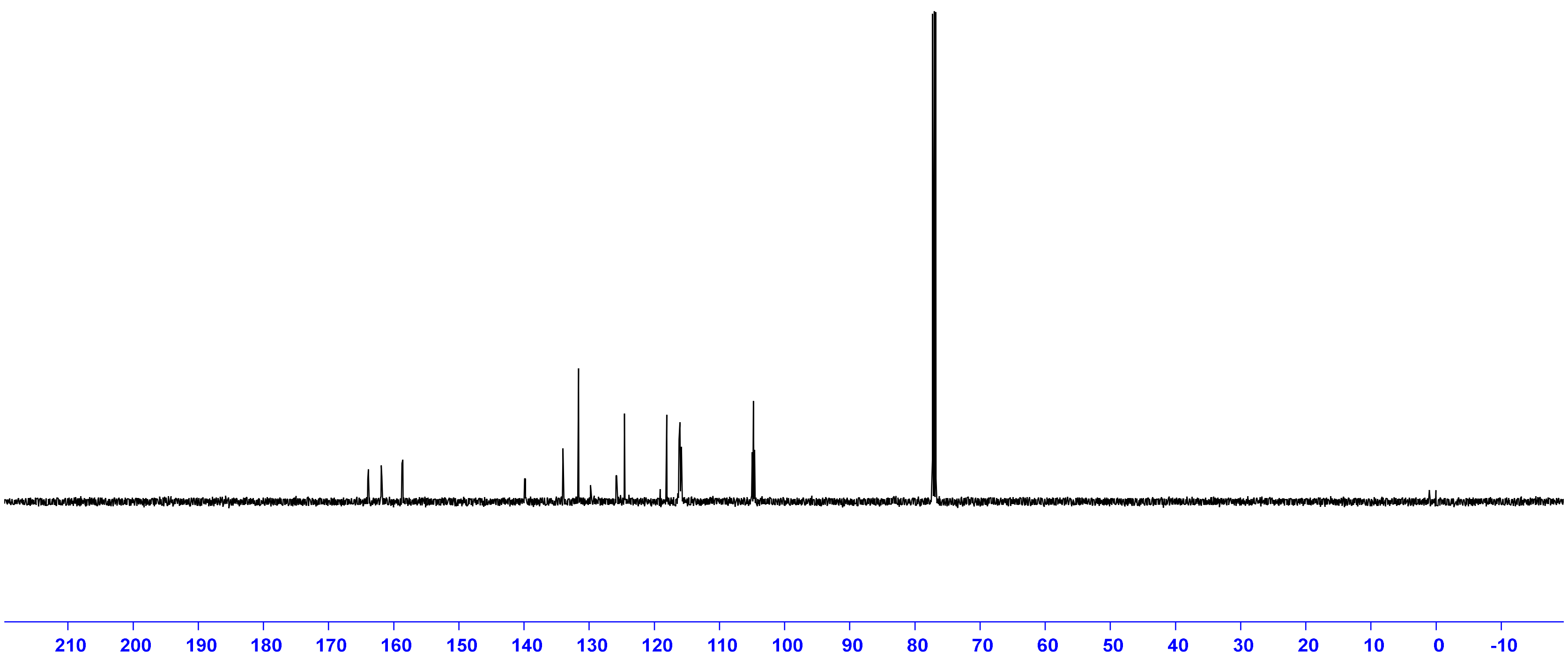

^{31}P NMR spectra for L2

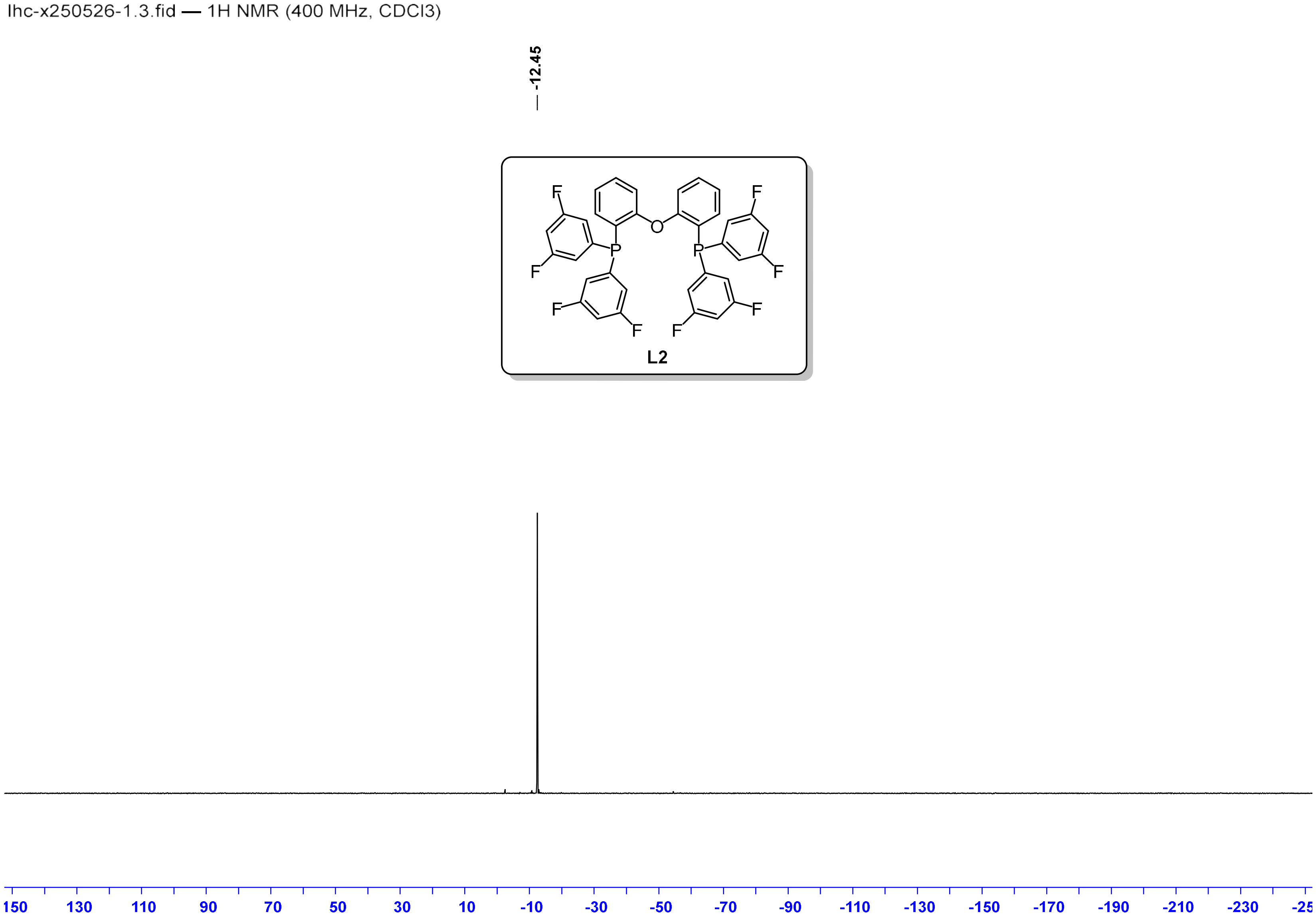

^{19}F NMR spectra for L2

lhc-x250526-1.4.fid — 1H NMR (400 MHz, CDCl3)

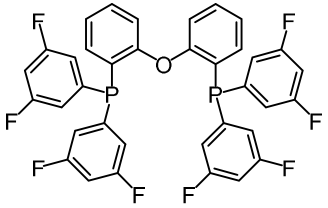

L2

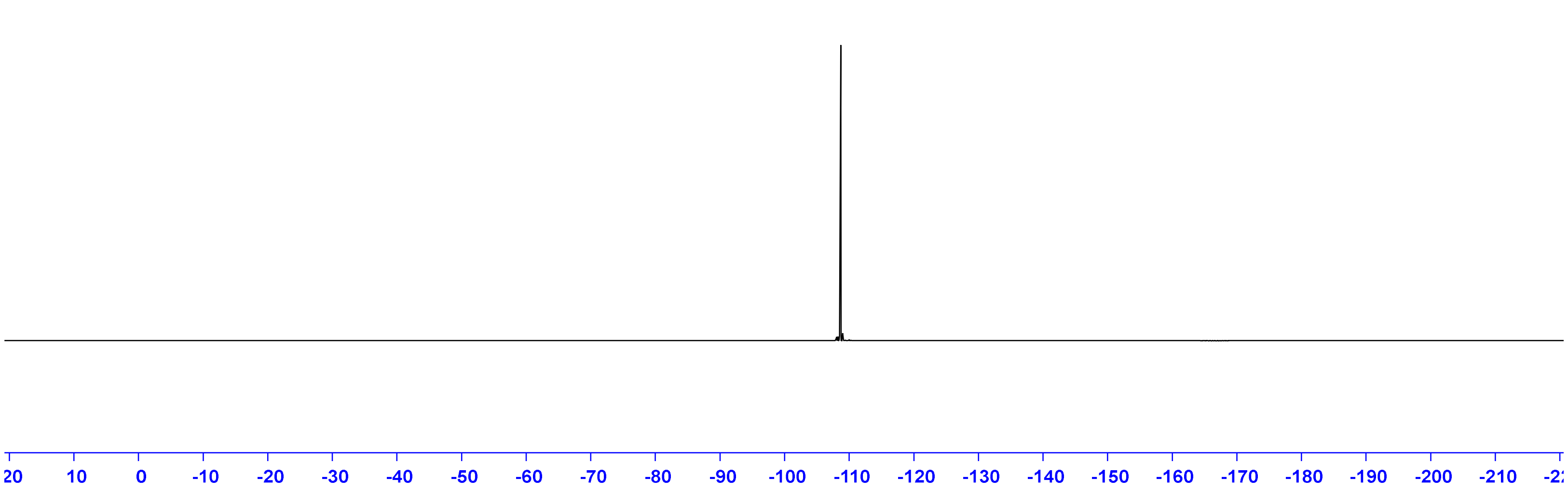

^{1}H NMR spectra for L3

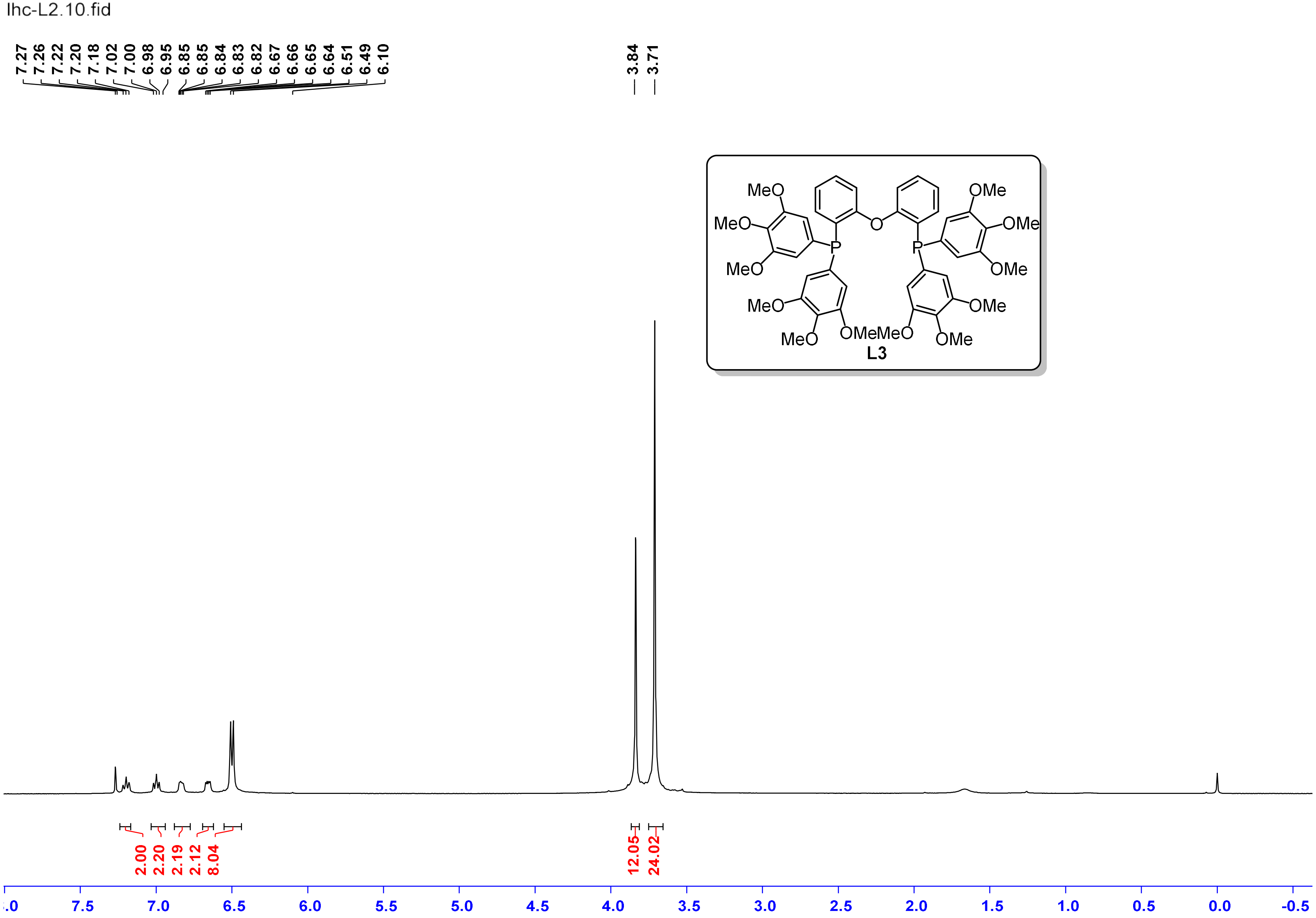

^{13}C NMR spectra for L3

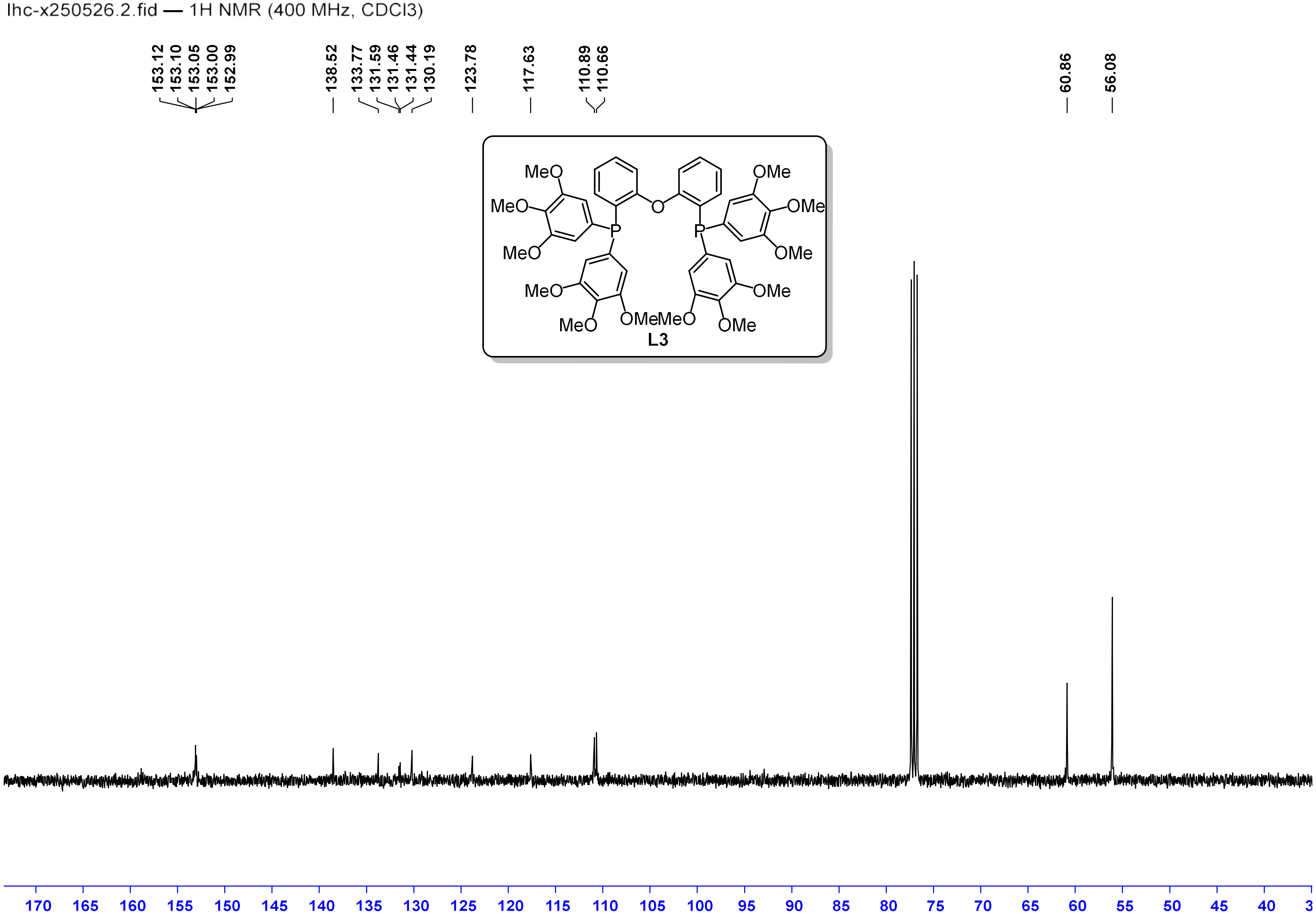

L3

^{31}P NMR spectra for L3

lhc-L2.12.fid

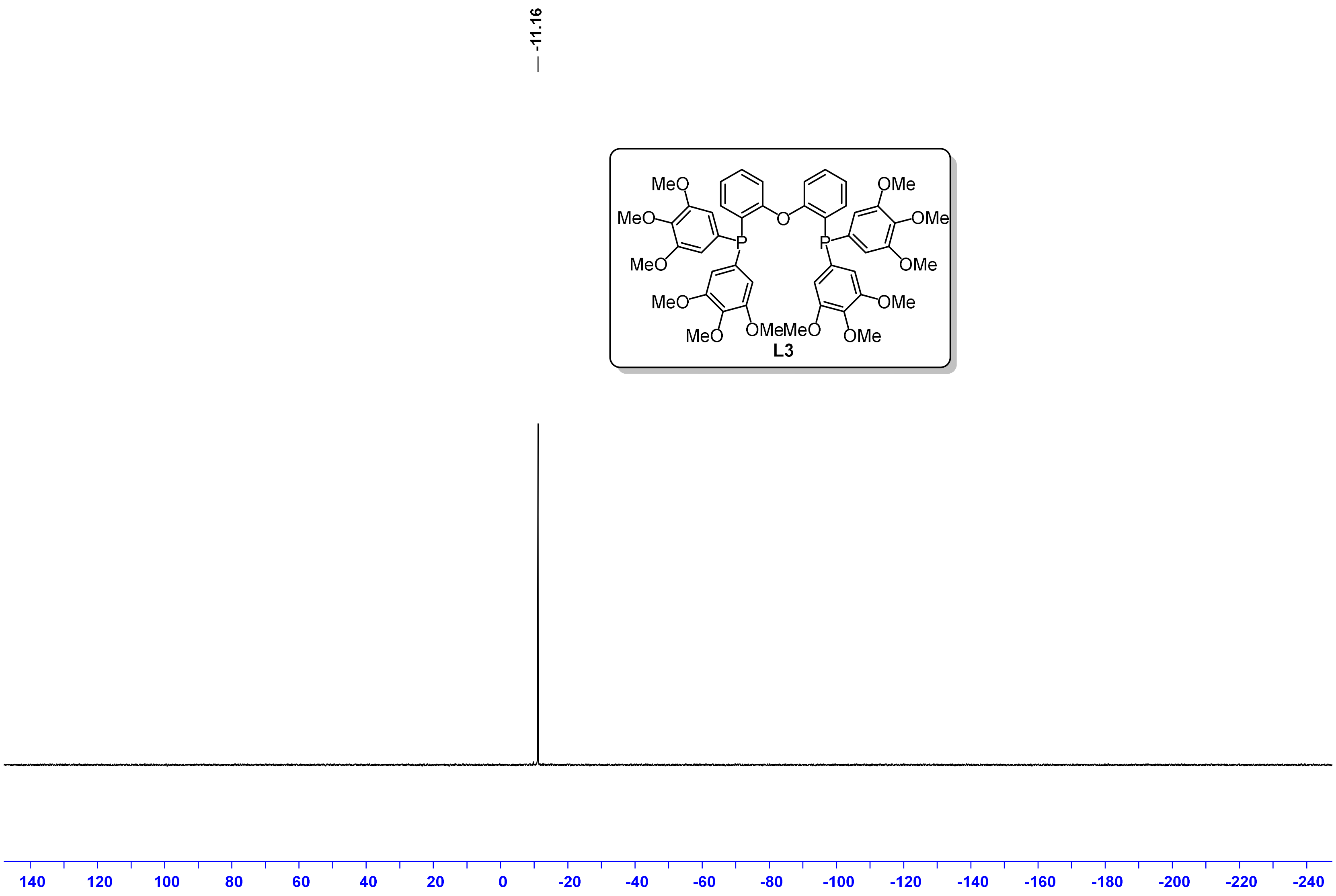

^{1}H NMR spectra for L4

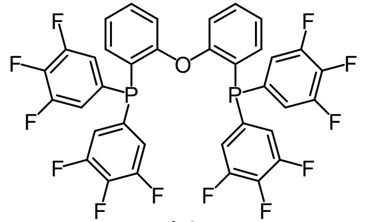

L4

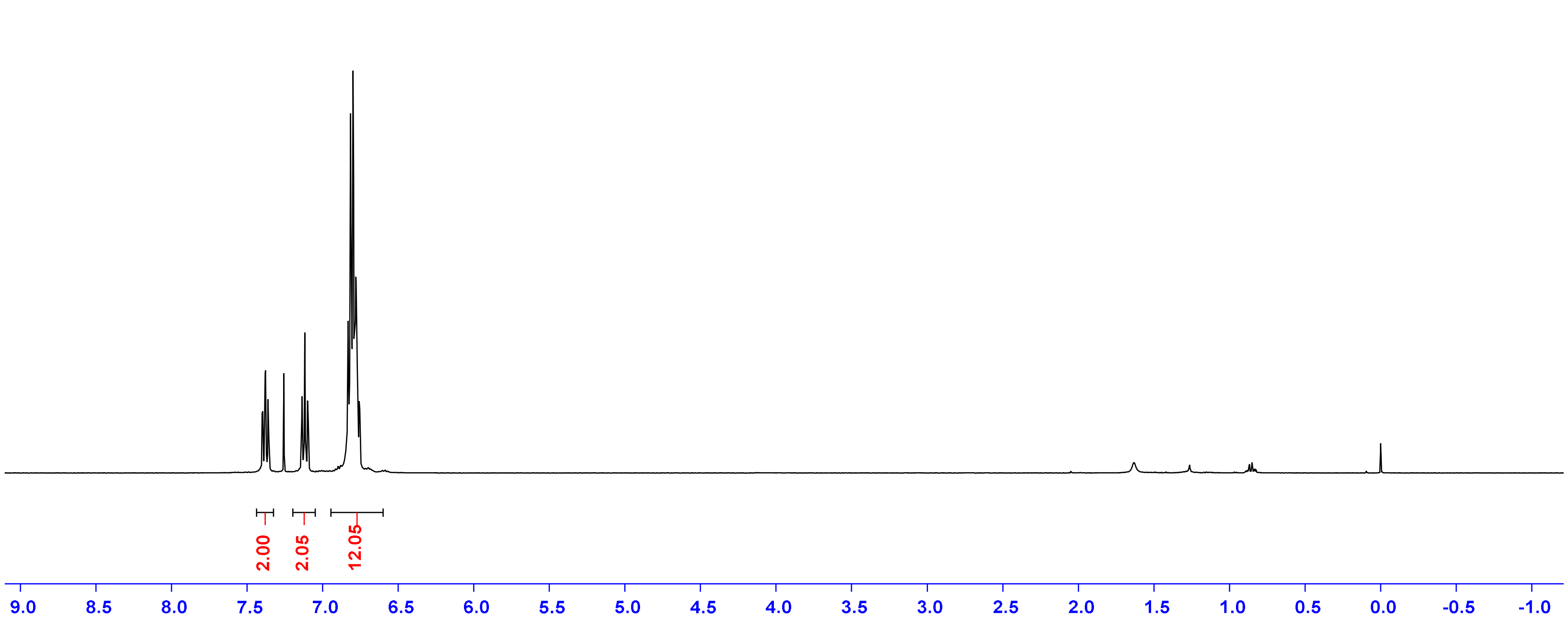

^{13}C NMR spectra for L4

lhc-x250526-2.2.fid — 1H NMR (400 MHz, CDCl3)

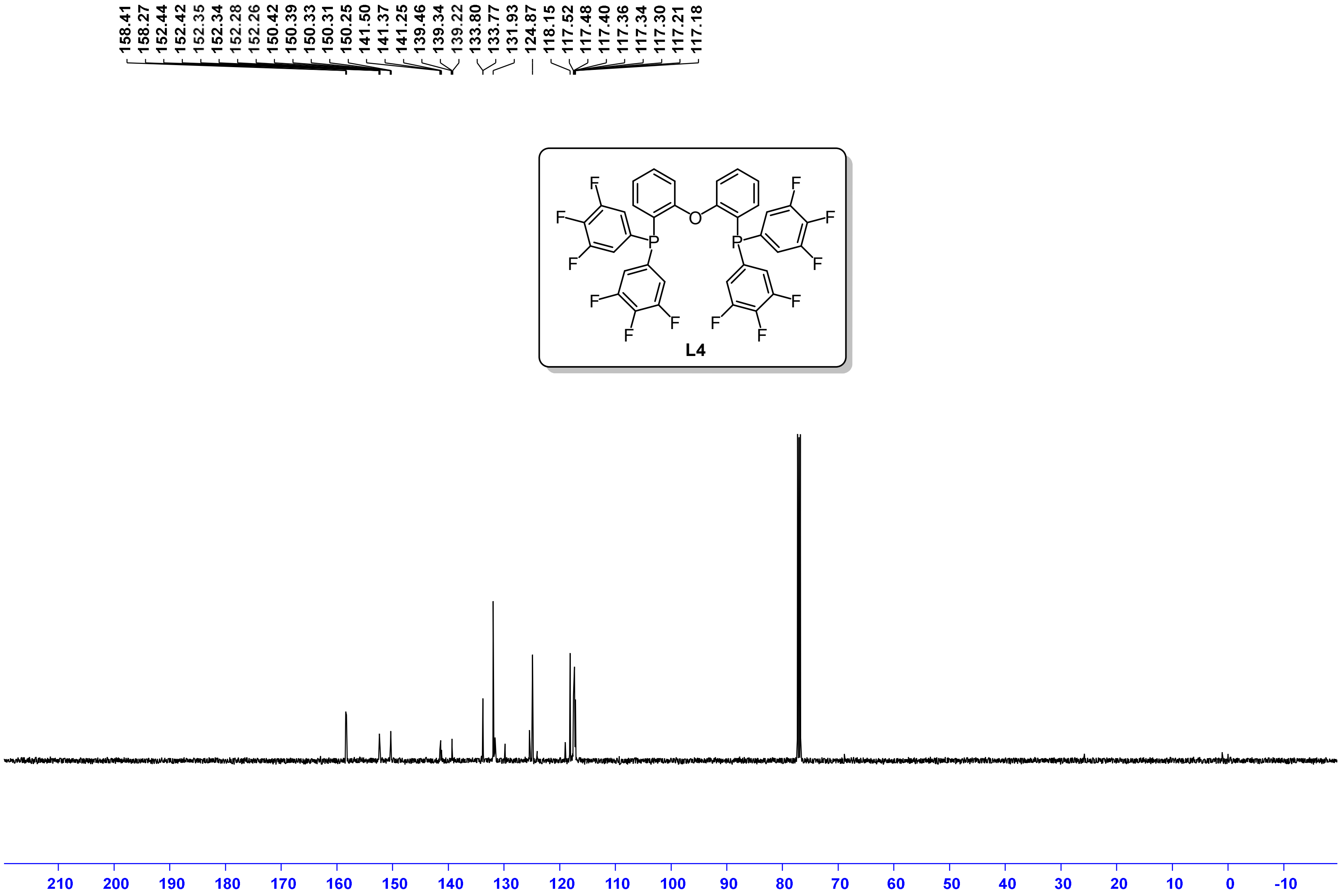

^{31}P NMR spectra for L4

lhc-x250526-2.3.fid — 1H NMR (400 MHz, CDCl3)

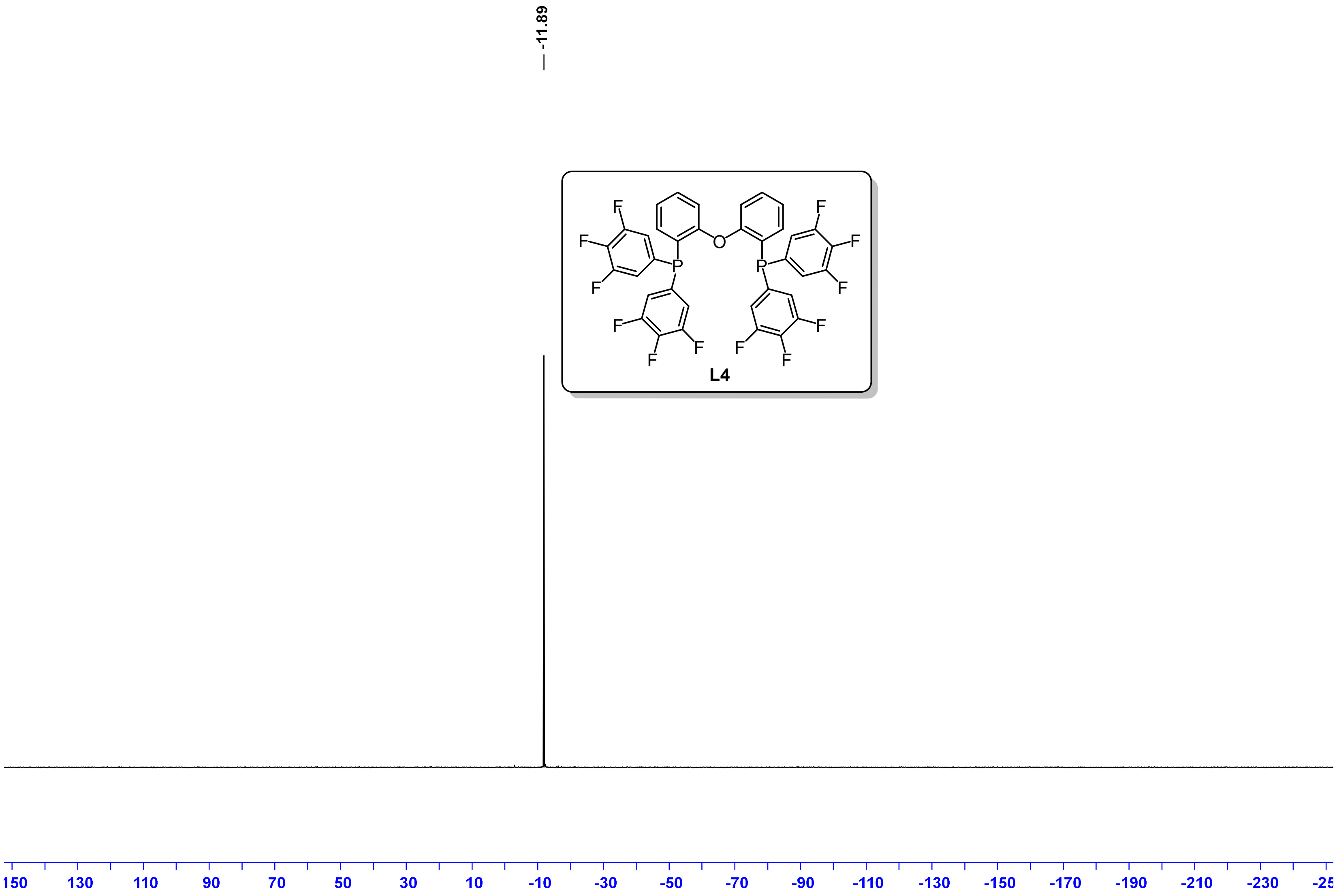

^{19}F NMR spectra for L4

lhc-x250526-2.4.fid — 1H NMR (400 MHz, CDCl3)

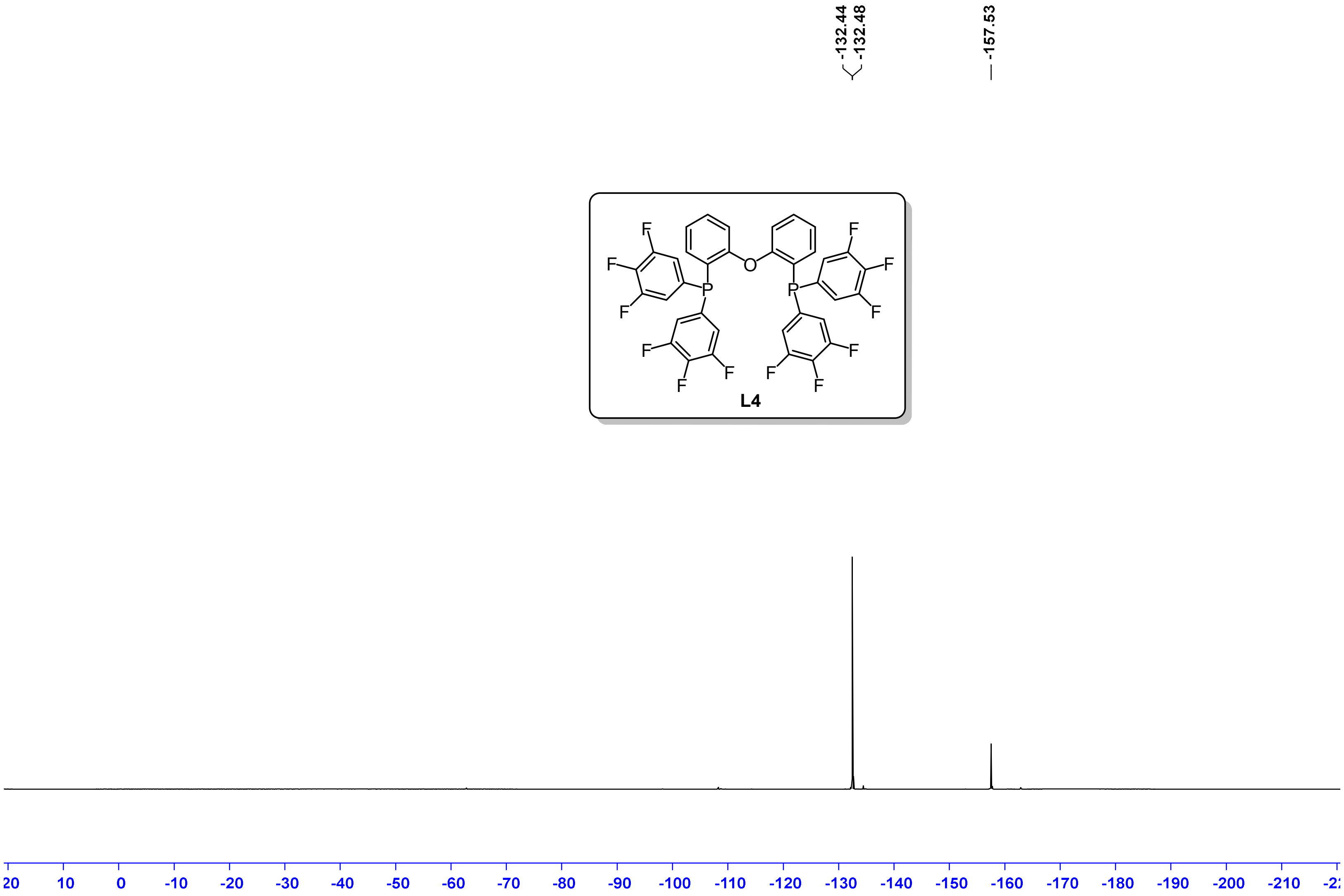

^{1}H NMR spectra for L5

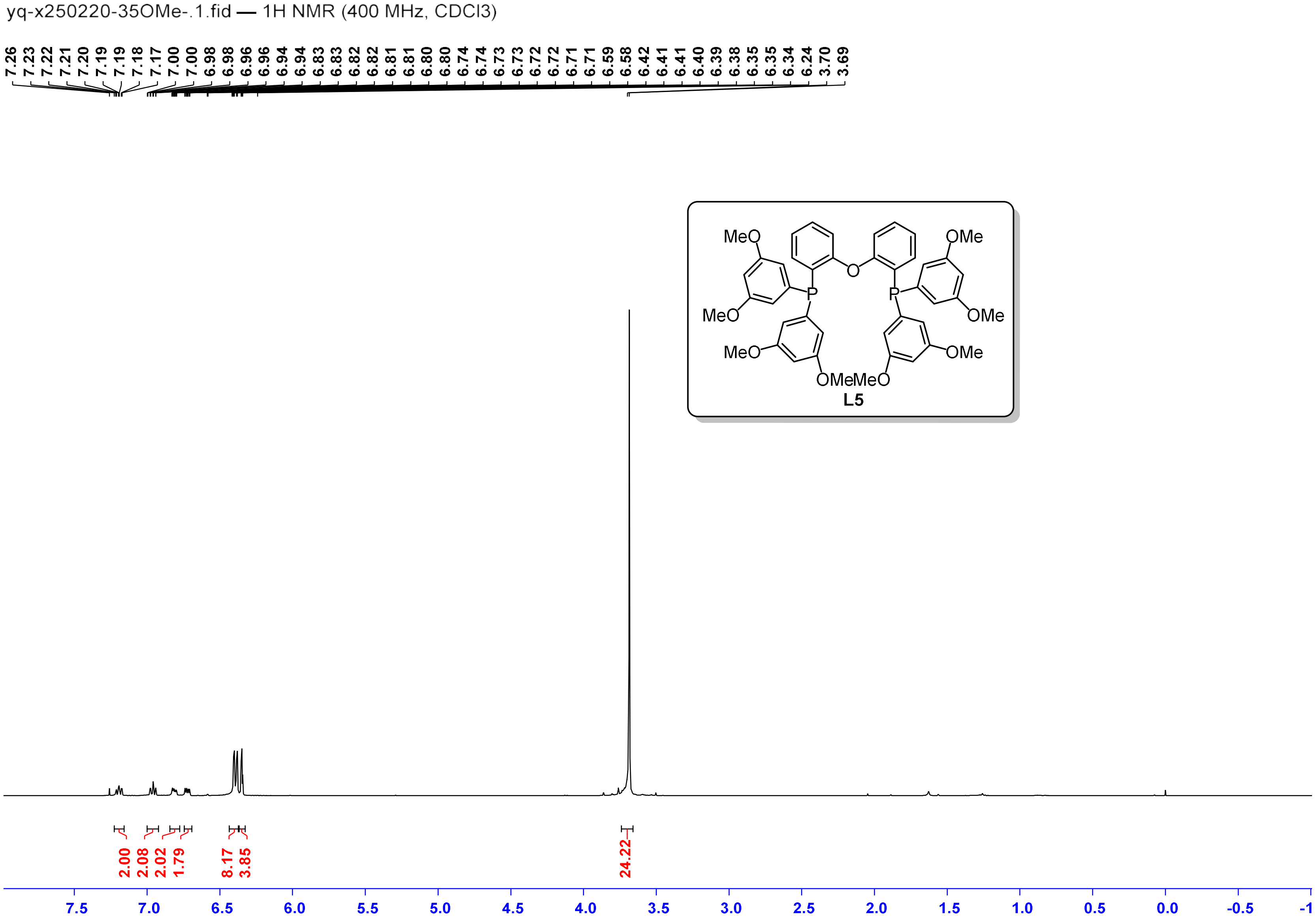

^{13}C NMR spectra for L5

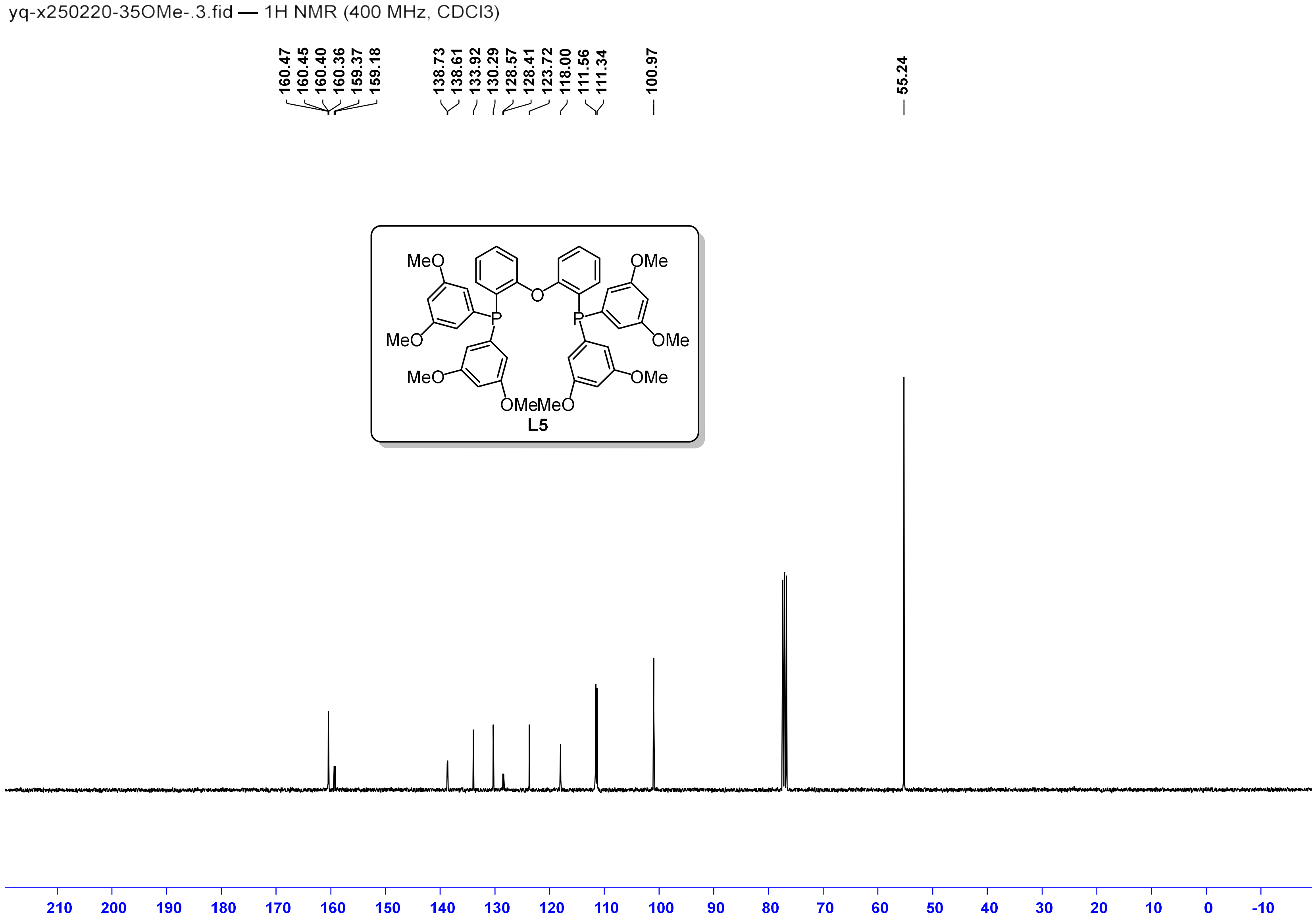

^{31}P NMR spectra for L5

yq-x250220-35OMe-.2.fid — 1H NMR (400 MHz, CDCl3)

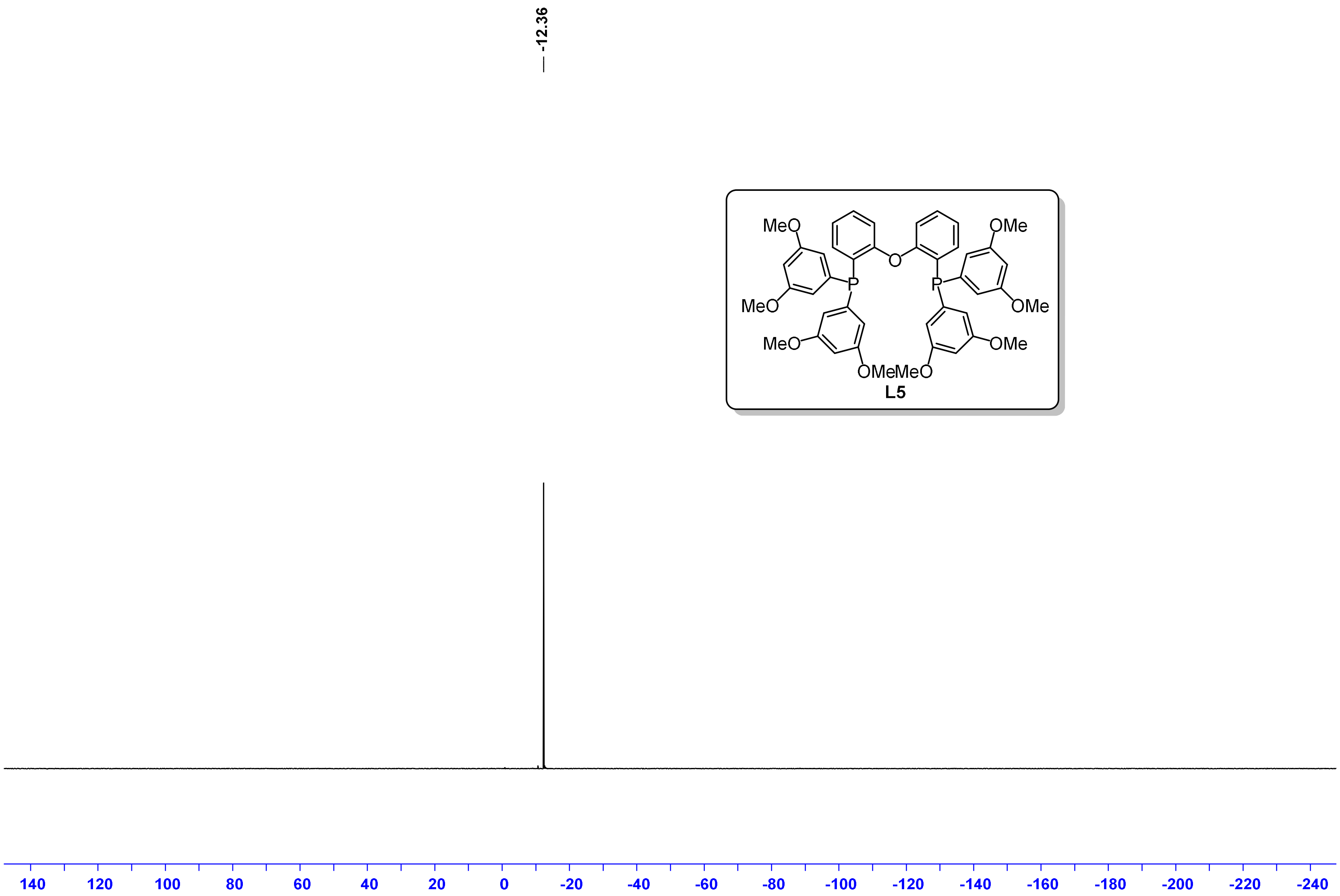

^{1}H NMR spectra for 1

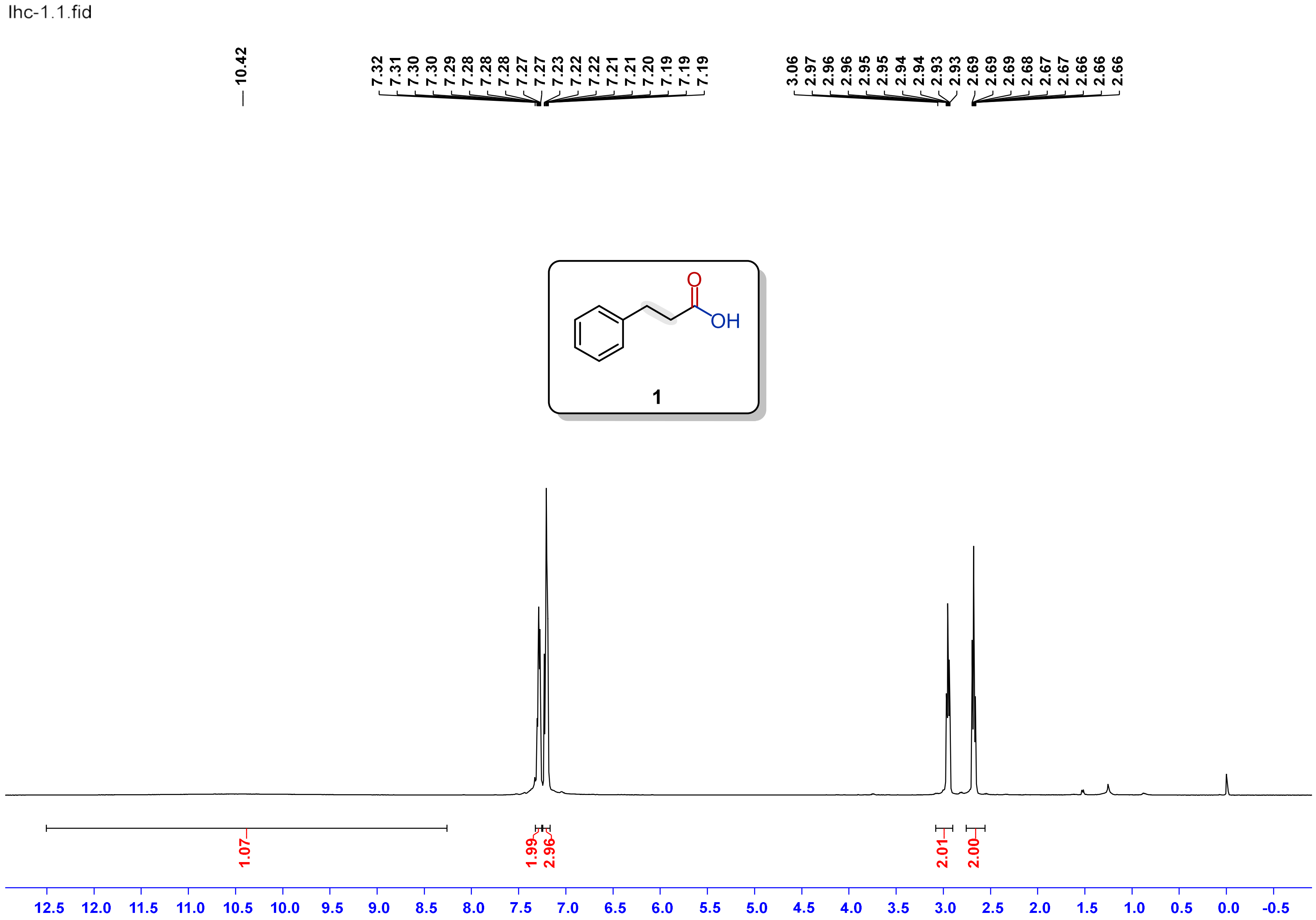

^{13}C NMR spectra for 1

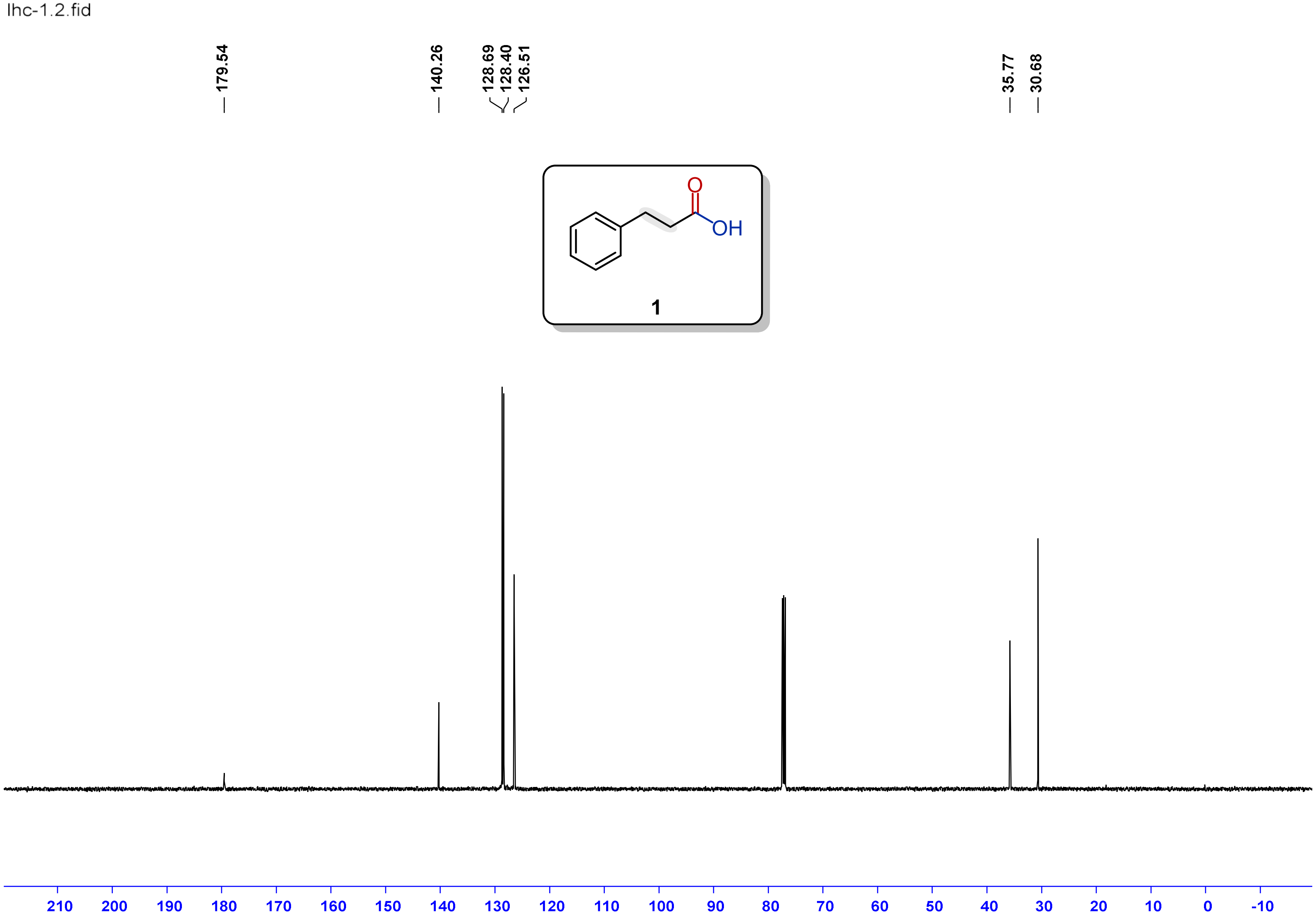

^{1}H NMR spectra for 2

hc-x24z06-1-1.1.fid — 1H NMR (400 MHz, CDCl3)

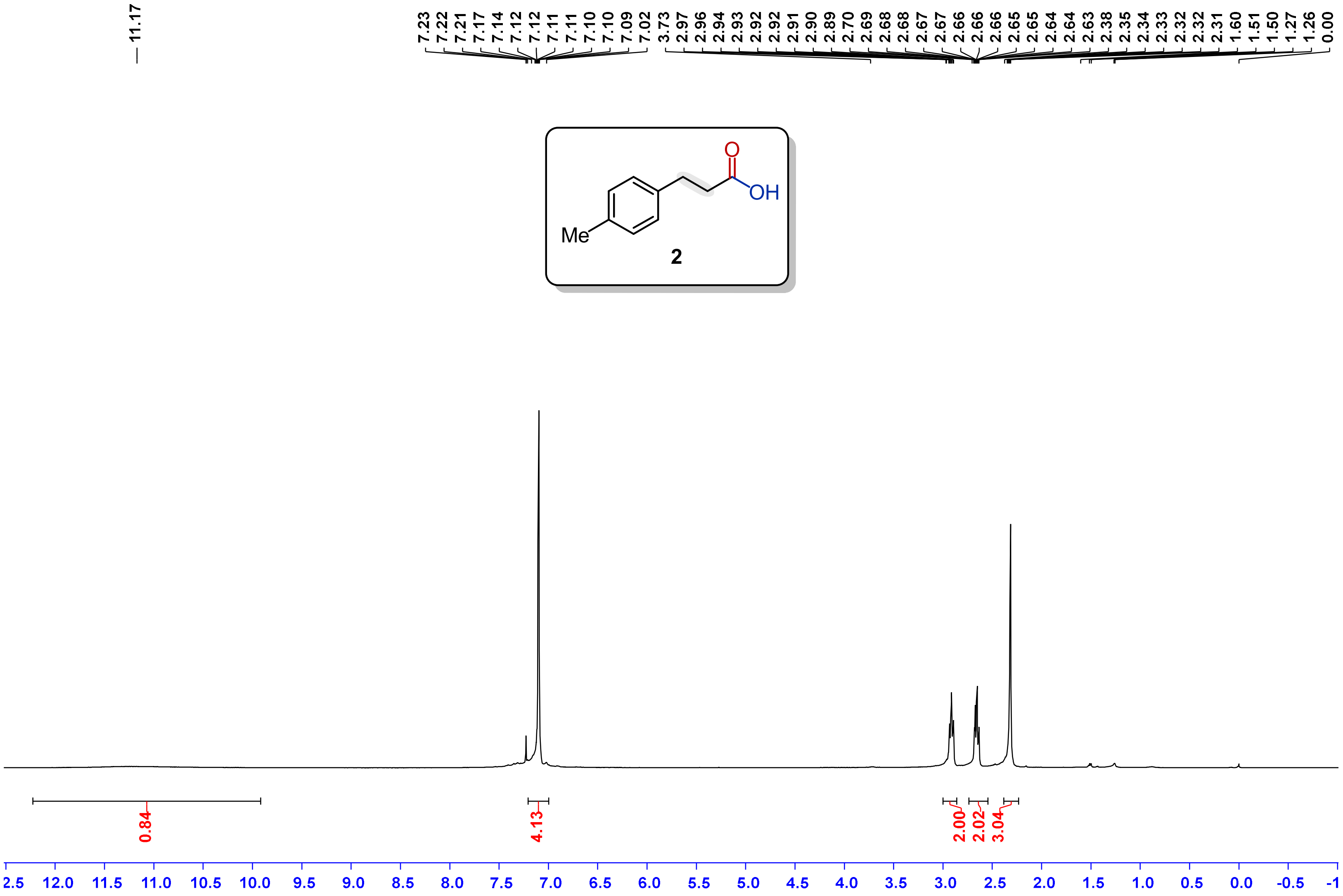

^{13}C NMR spectra for 2

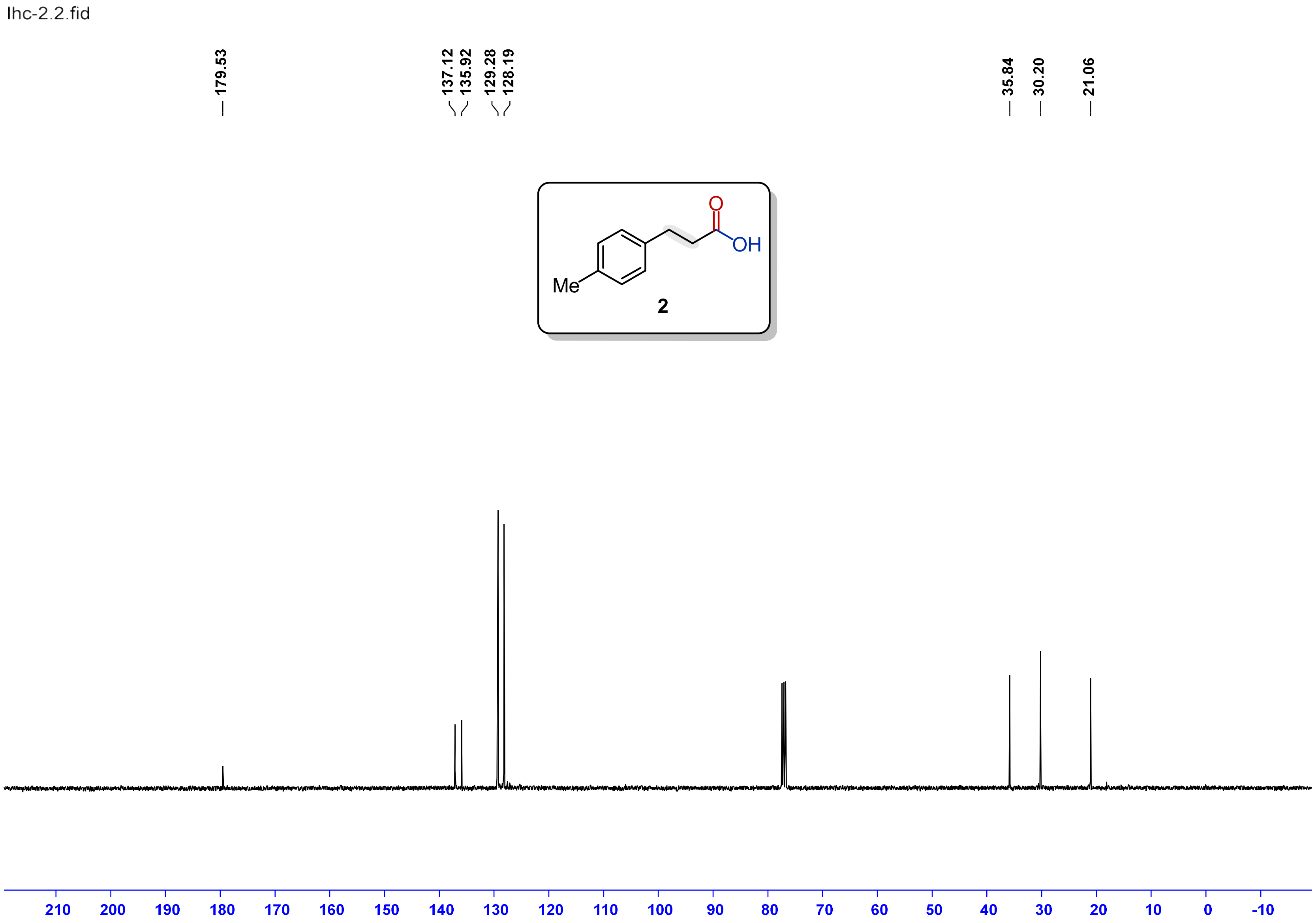

^{1}H NMR spectra for 3

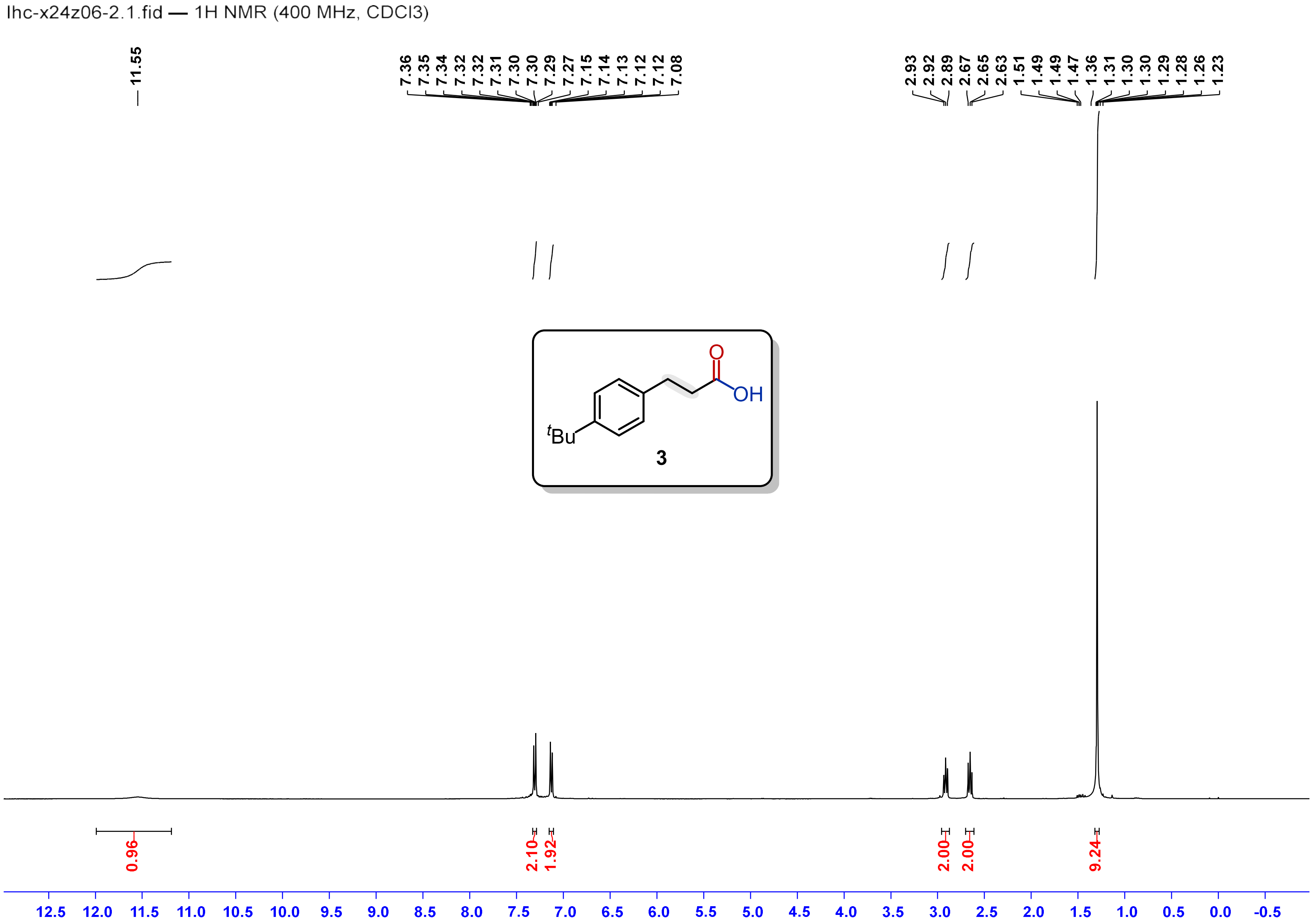

^{13}C NMR spectra for 3

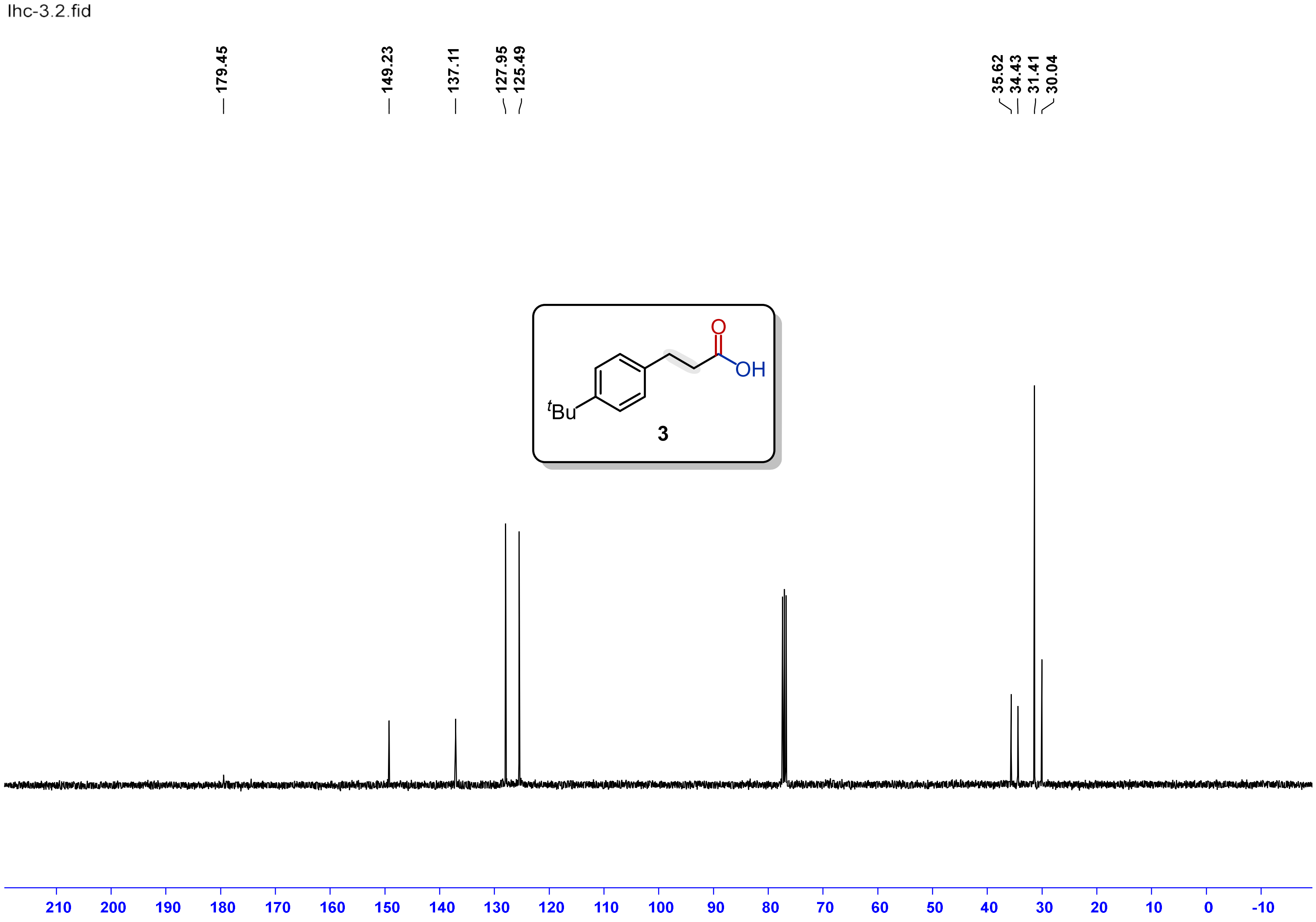

^{1}H NMR spectra for 4

lhc-x250526-1.10.fid — 1H NMR (400 MHz, CDCl3)

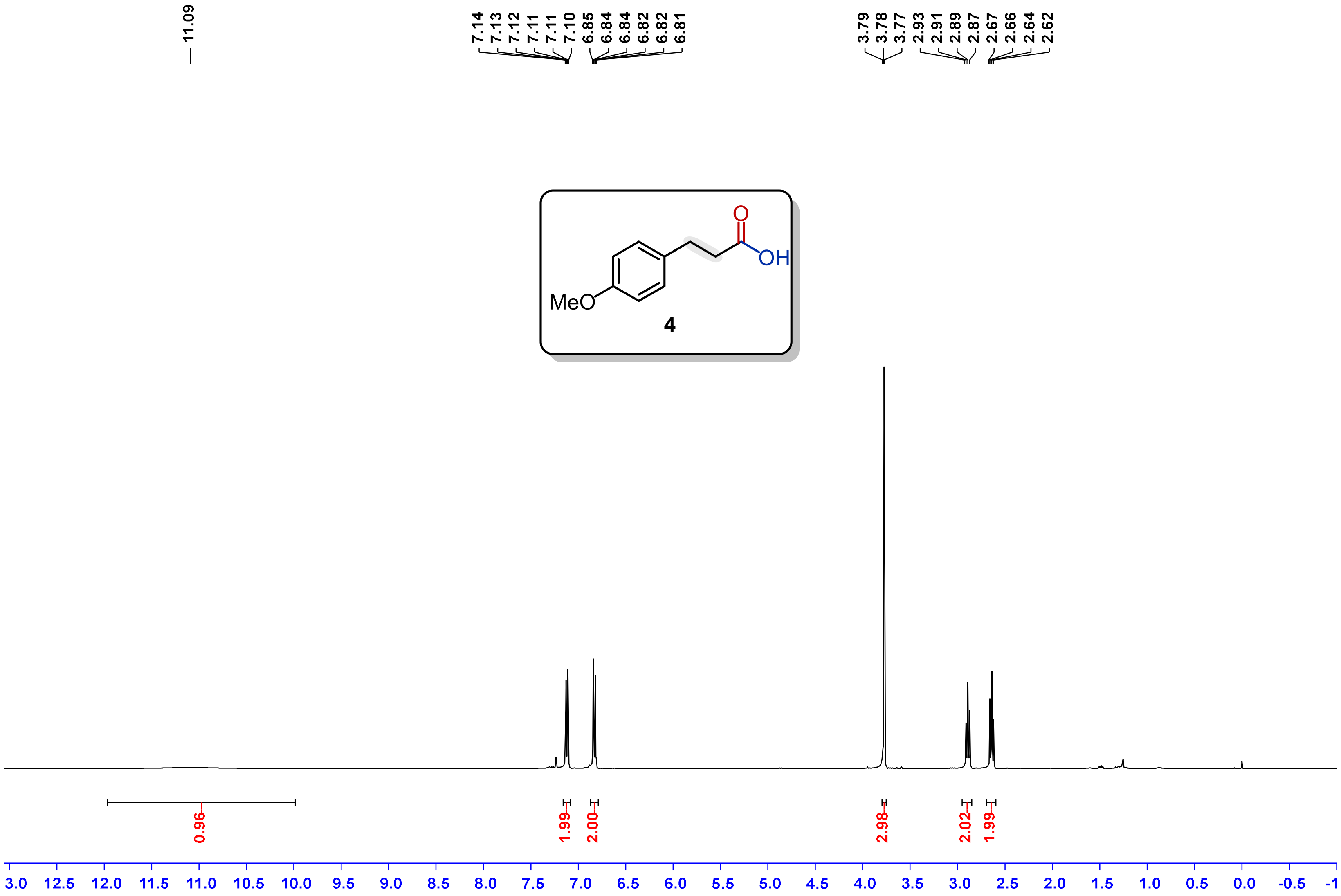

^{13}C NMR spectra for 4

lhc-x250526-1.11.fid — 1H NMR (400 MHz, CDCl3)

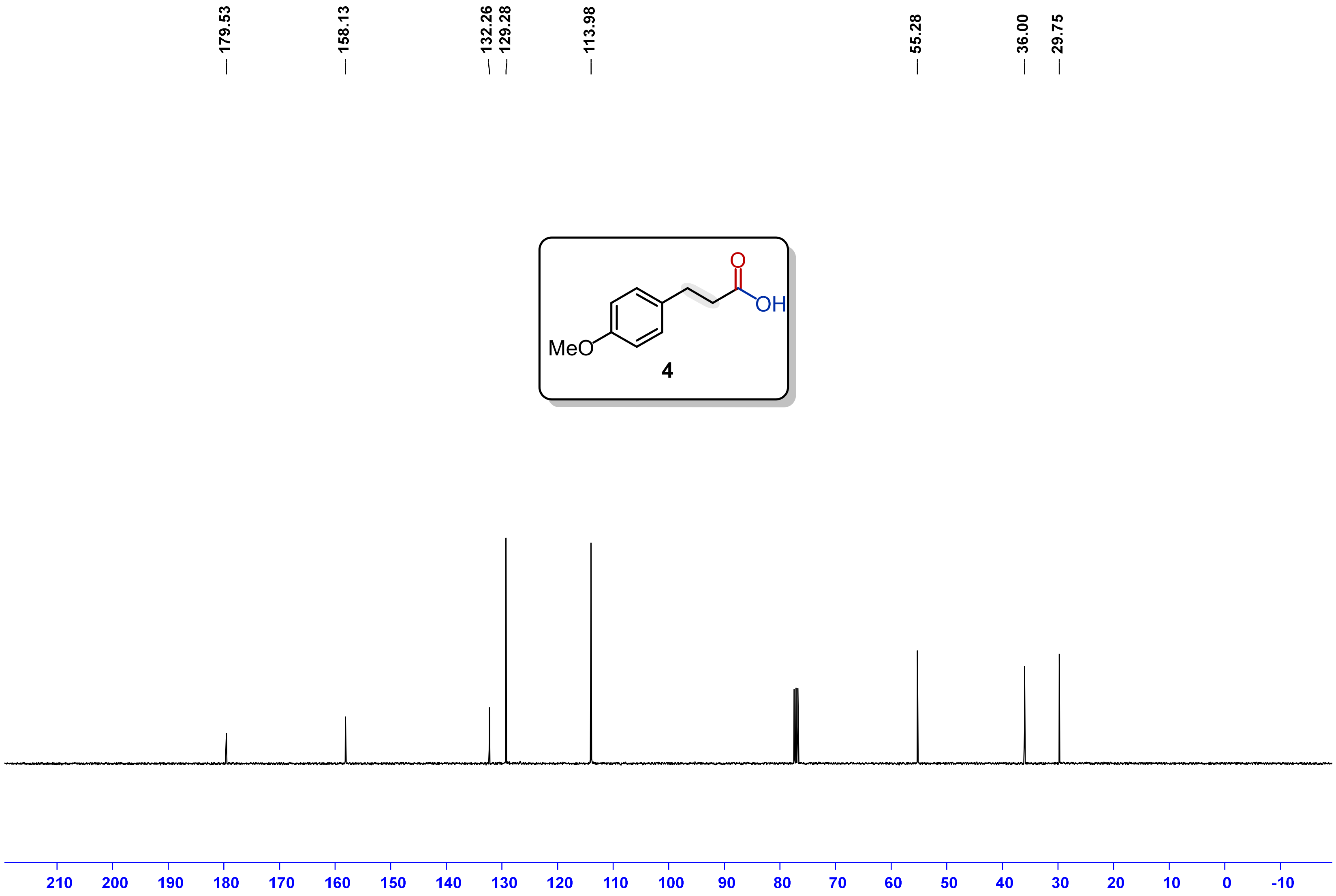

^{1}H NMR spectra for 5

lhc-x24z10-2.1.fid — 1H NMR (400 MHz, CDCl3)

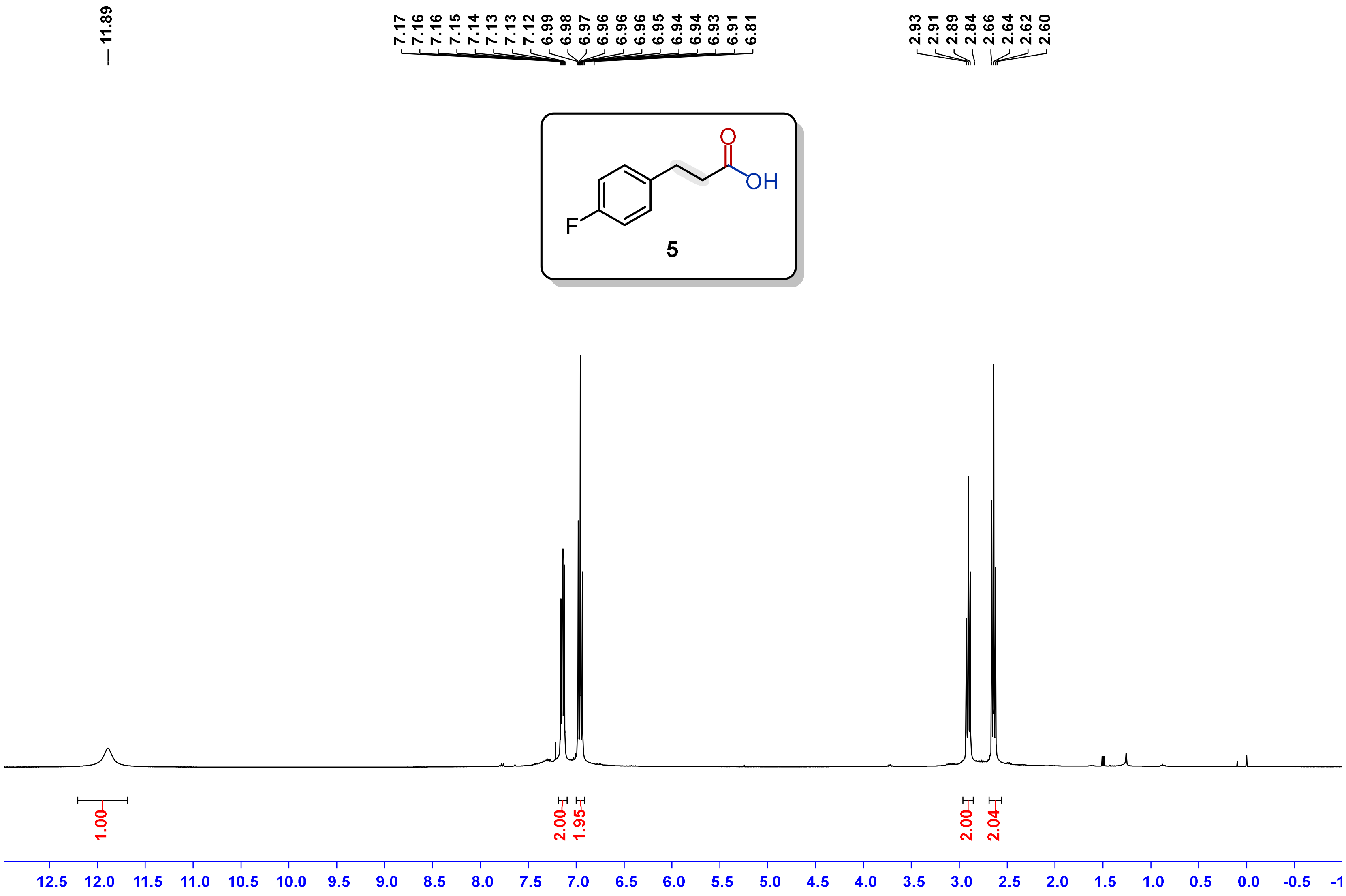

^{13}C NMR spectra for 5

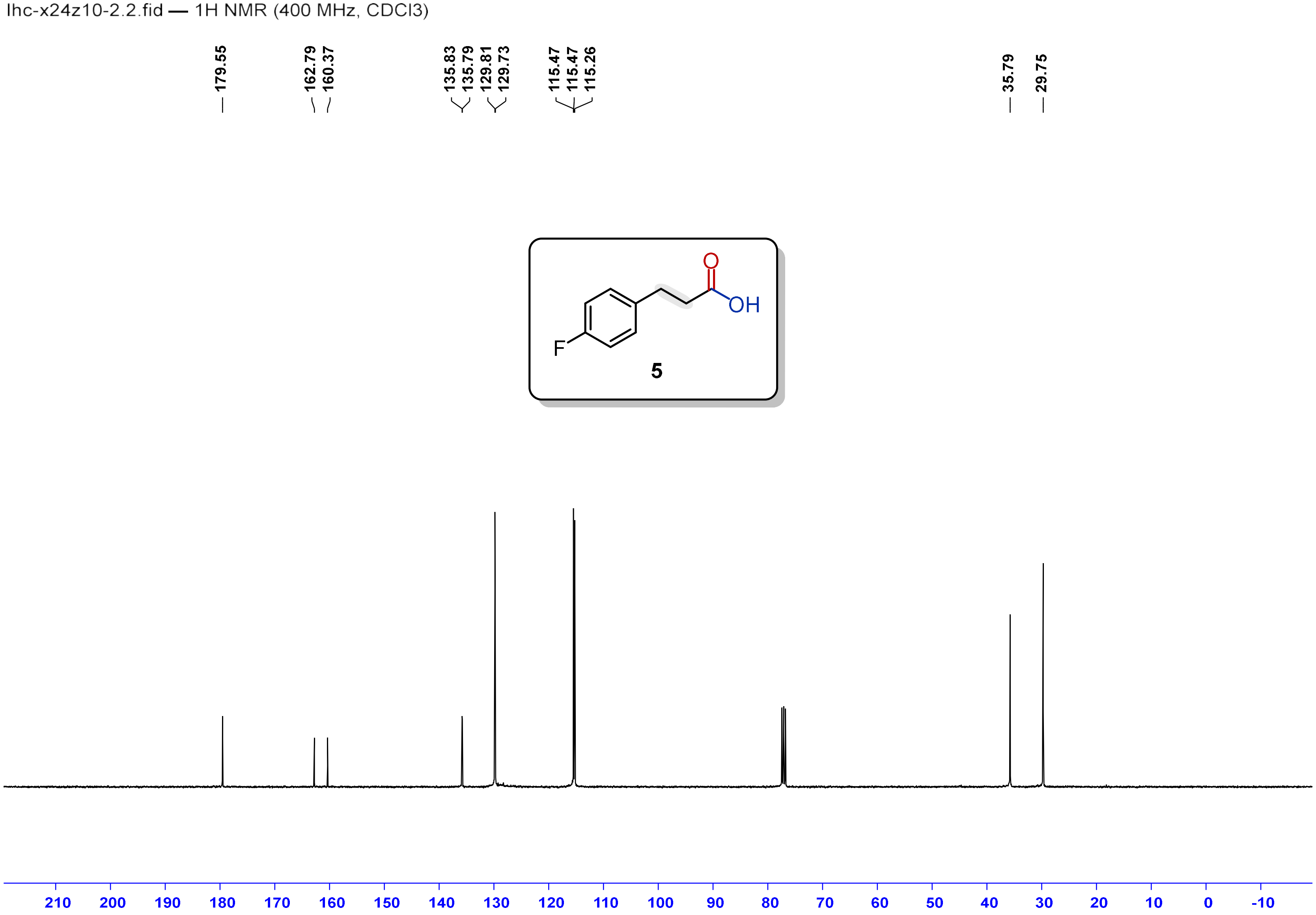

^{19}F NMR spectra for 5

lhc-x24z10-2-F.1.fid — 1H NMR (400 MHz, CDCl3)

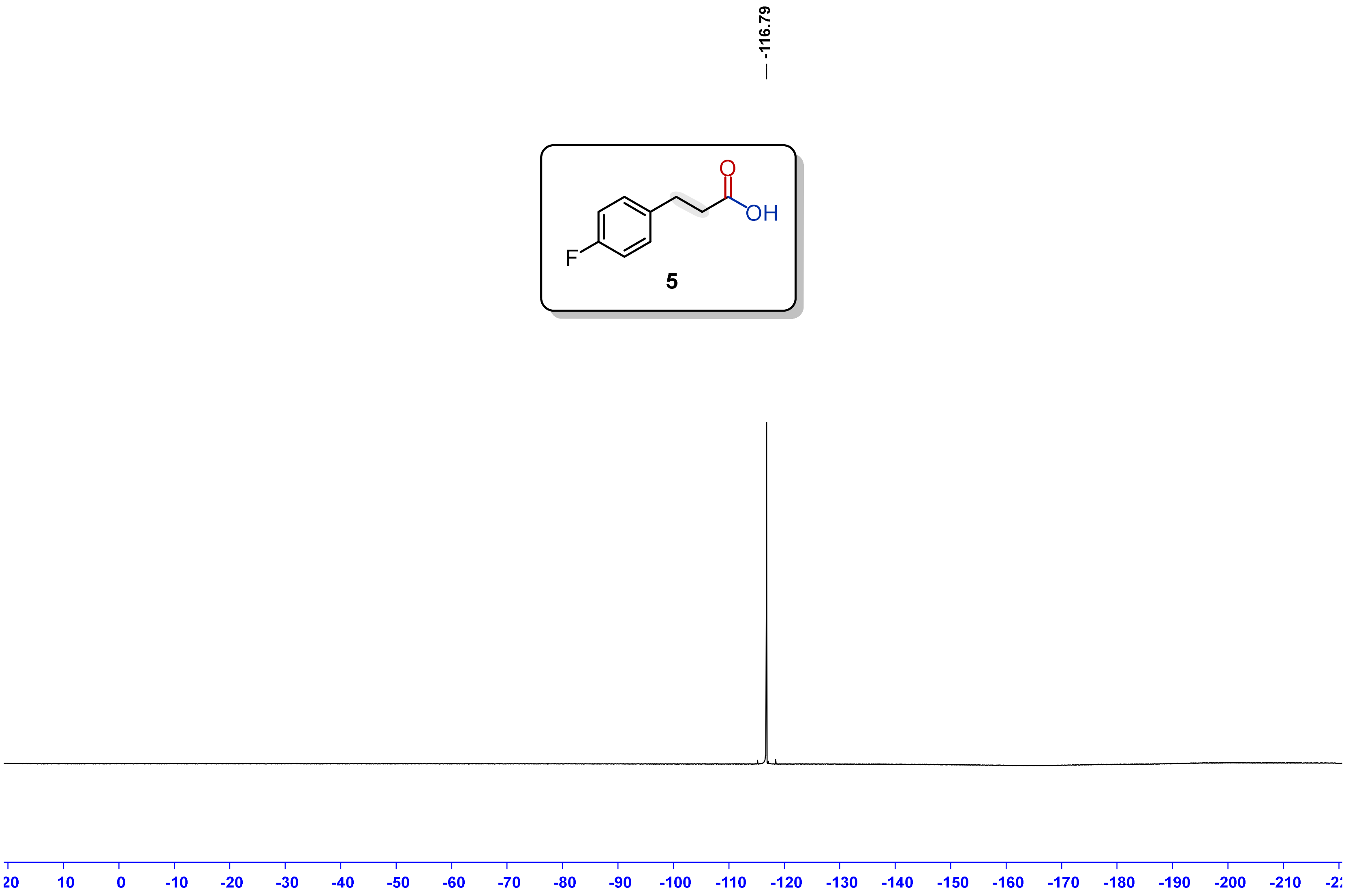

^{1}H NMR spectra for 6

lhc-x250701-1.1.fid

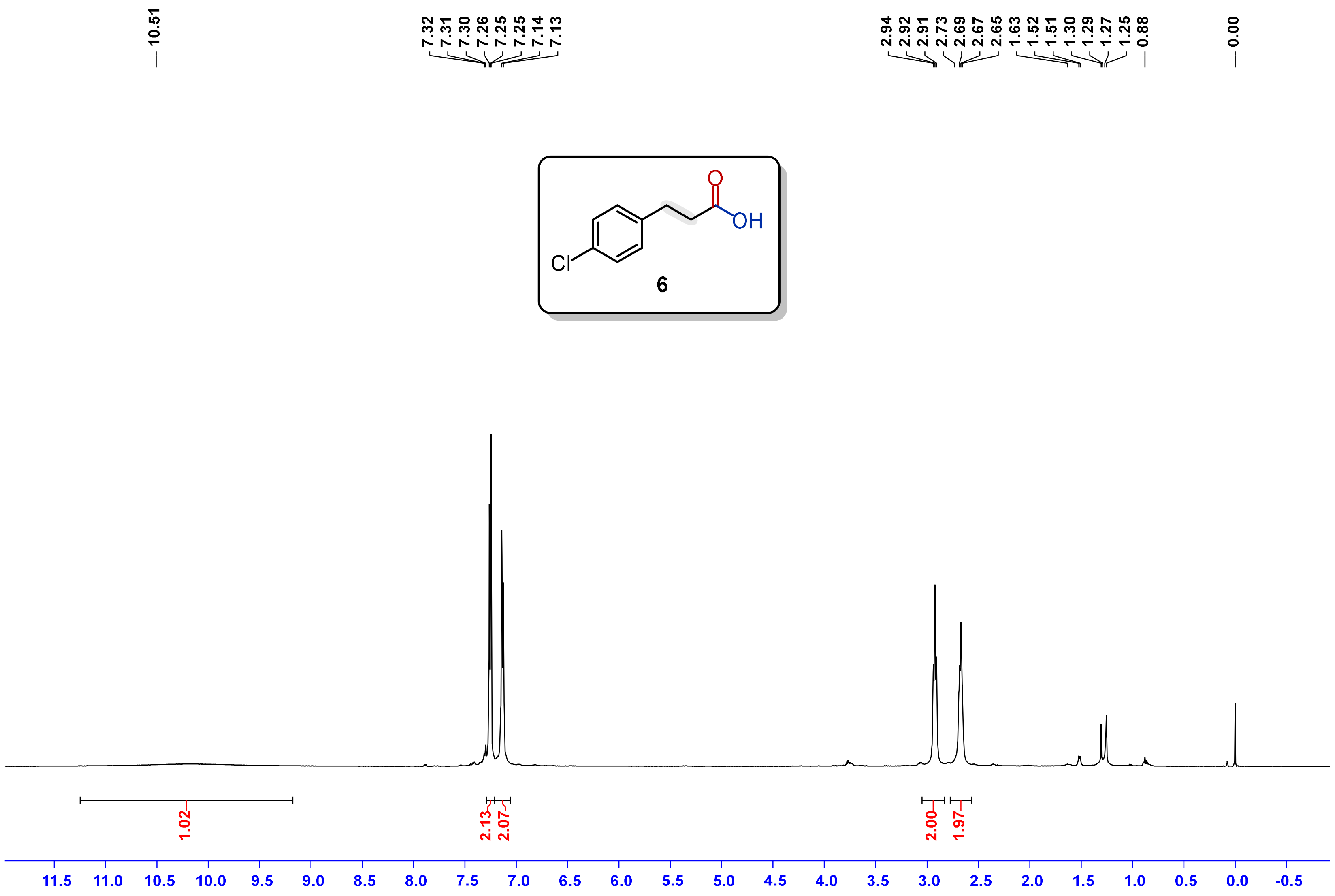

^{13}C NMR spectra for 6

lhc-x250701-1.2.fid

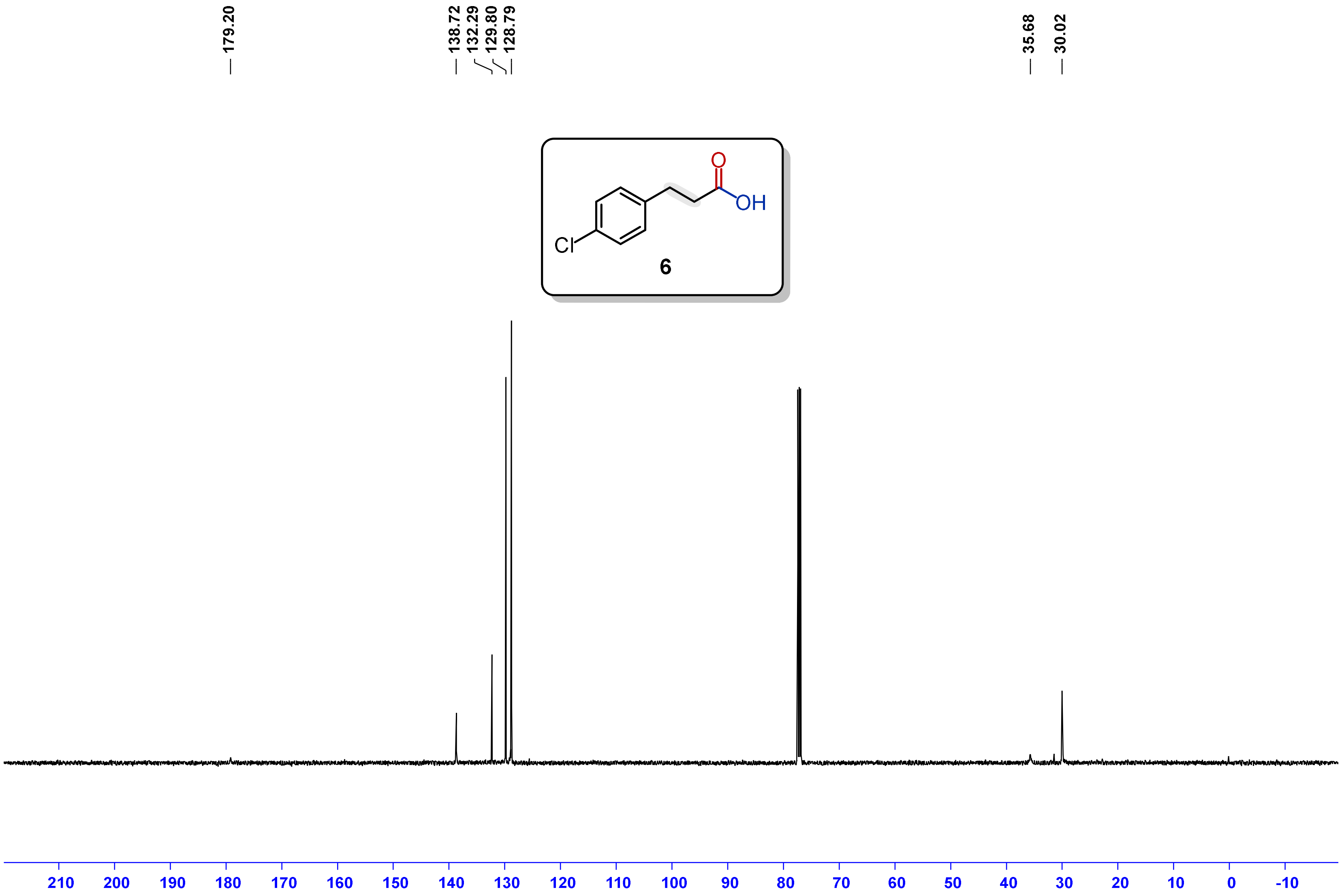

^{1}H NMR spectra for 7

lhc-x250309-2.1.fid — 1H NMR (400 MHz, CDCl3)

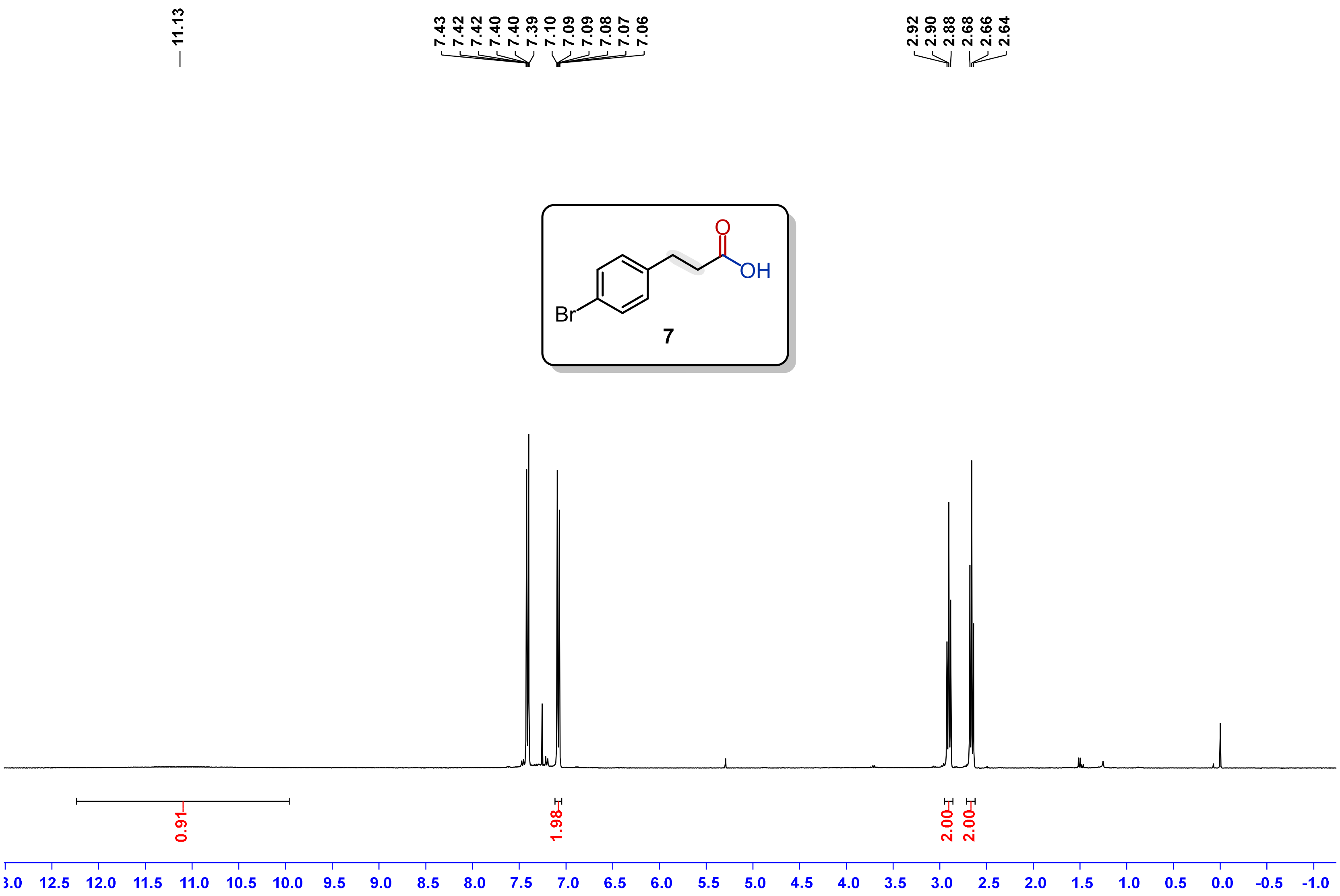

^{13}C NMR spectra for 7

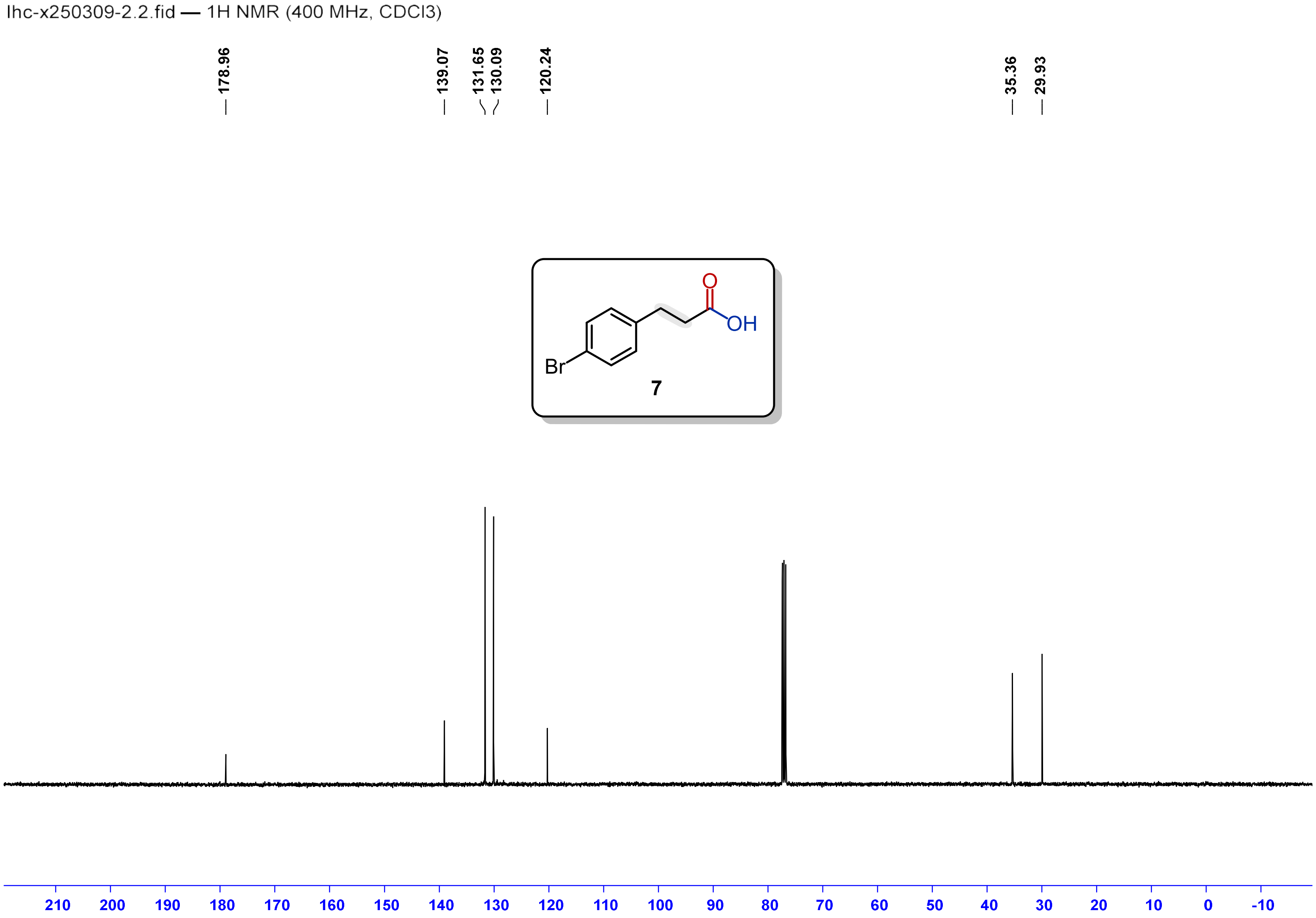

^{1}H NMR spectra for 8

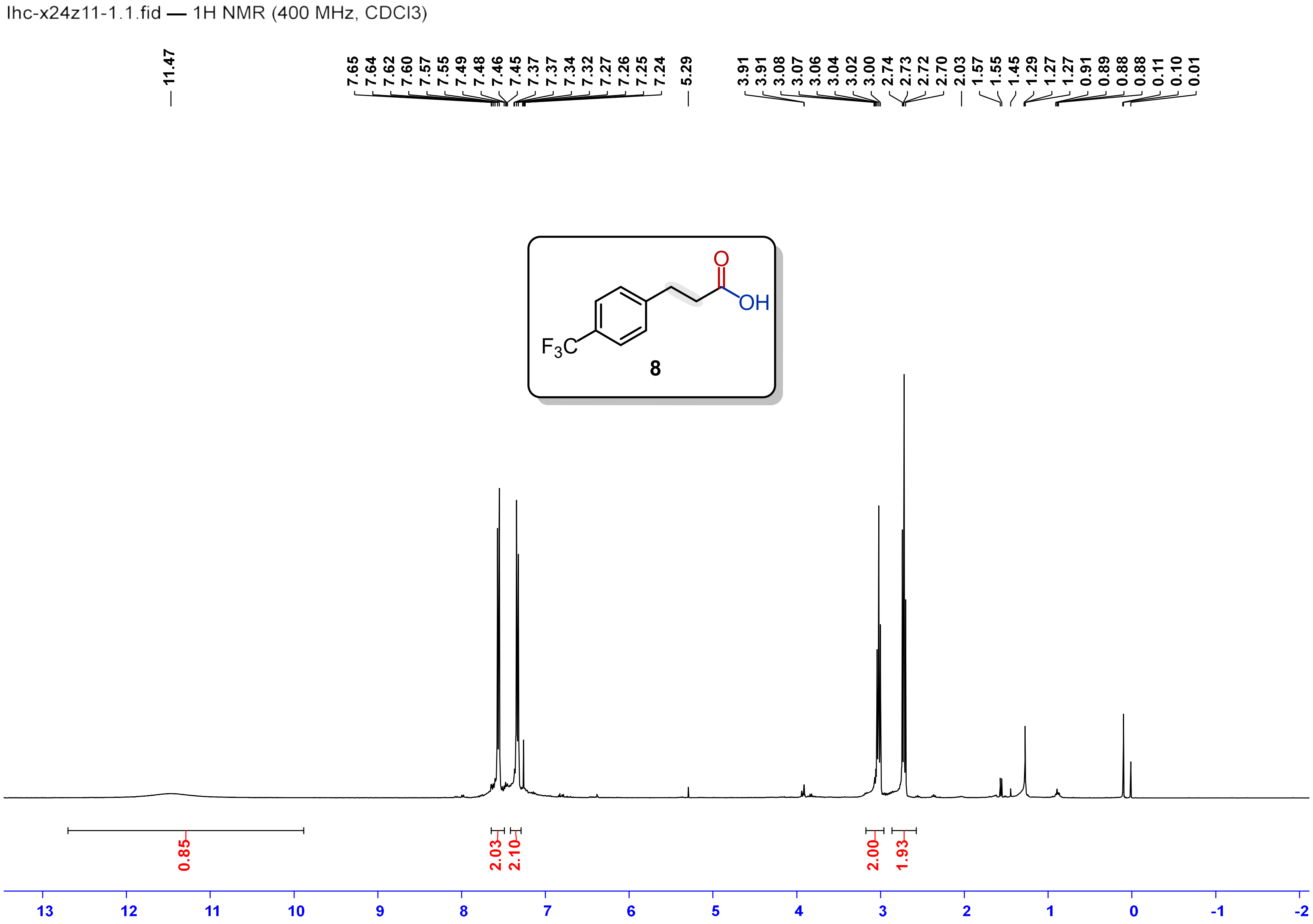

^{13}C NMR spectra for 8

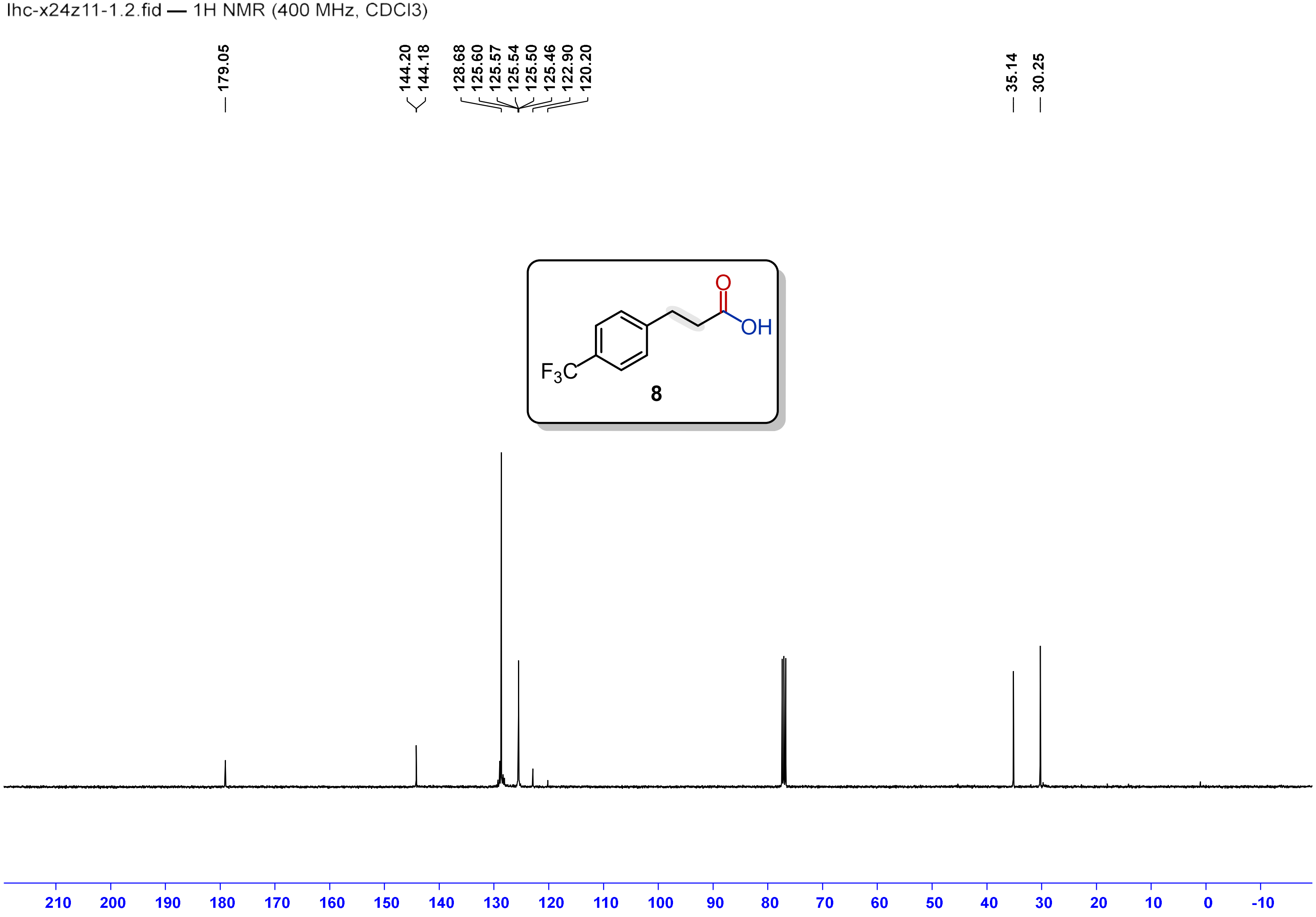

^{19}F NMR spectra for 8

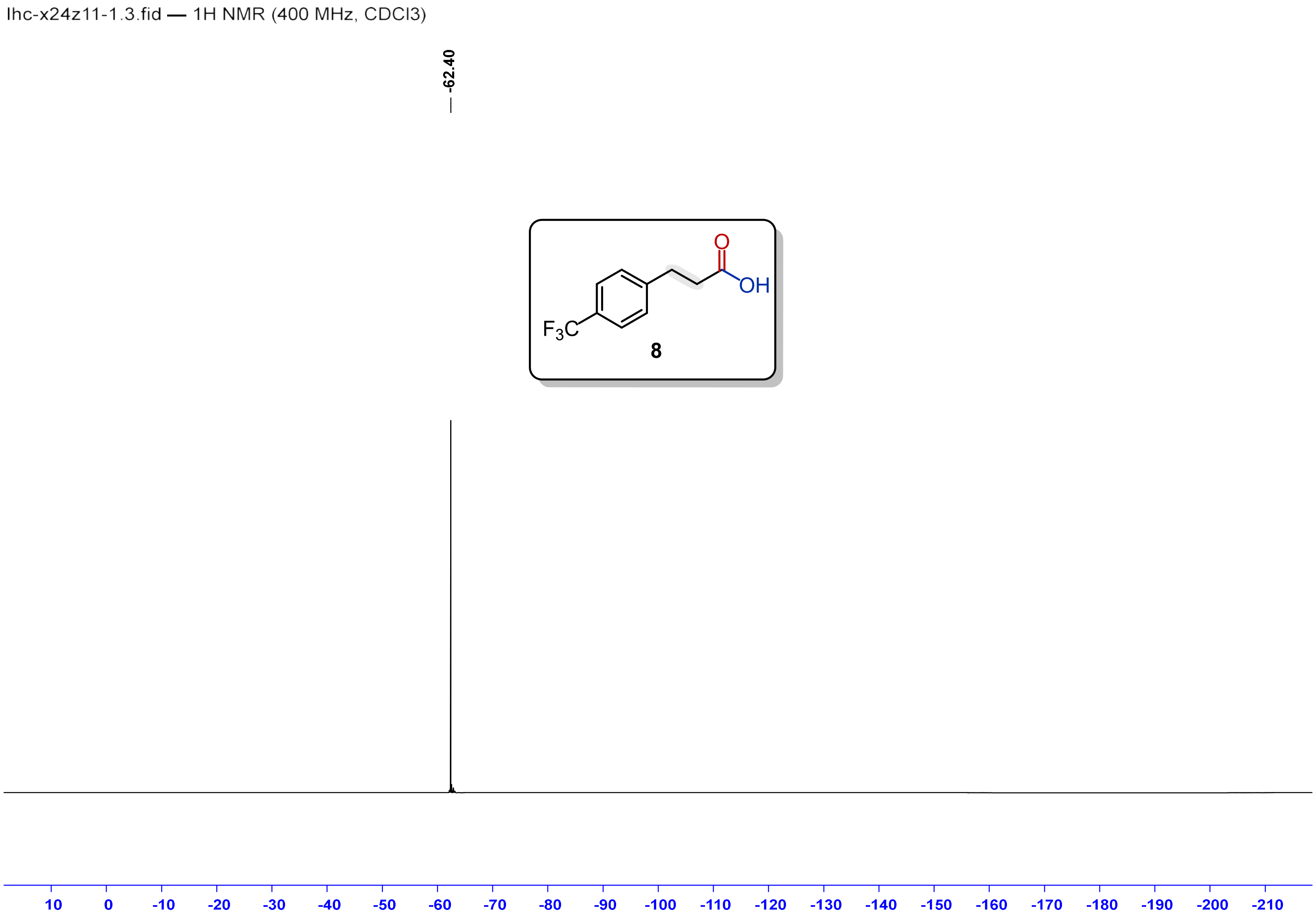

^{1}H NMR spectra for 9

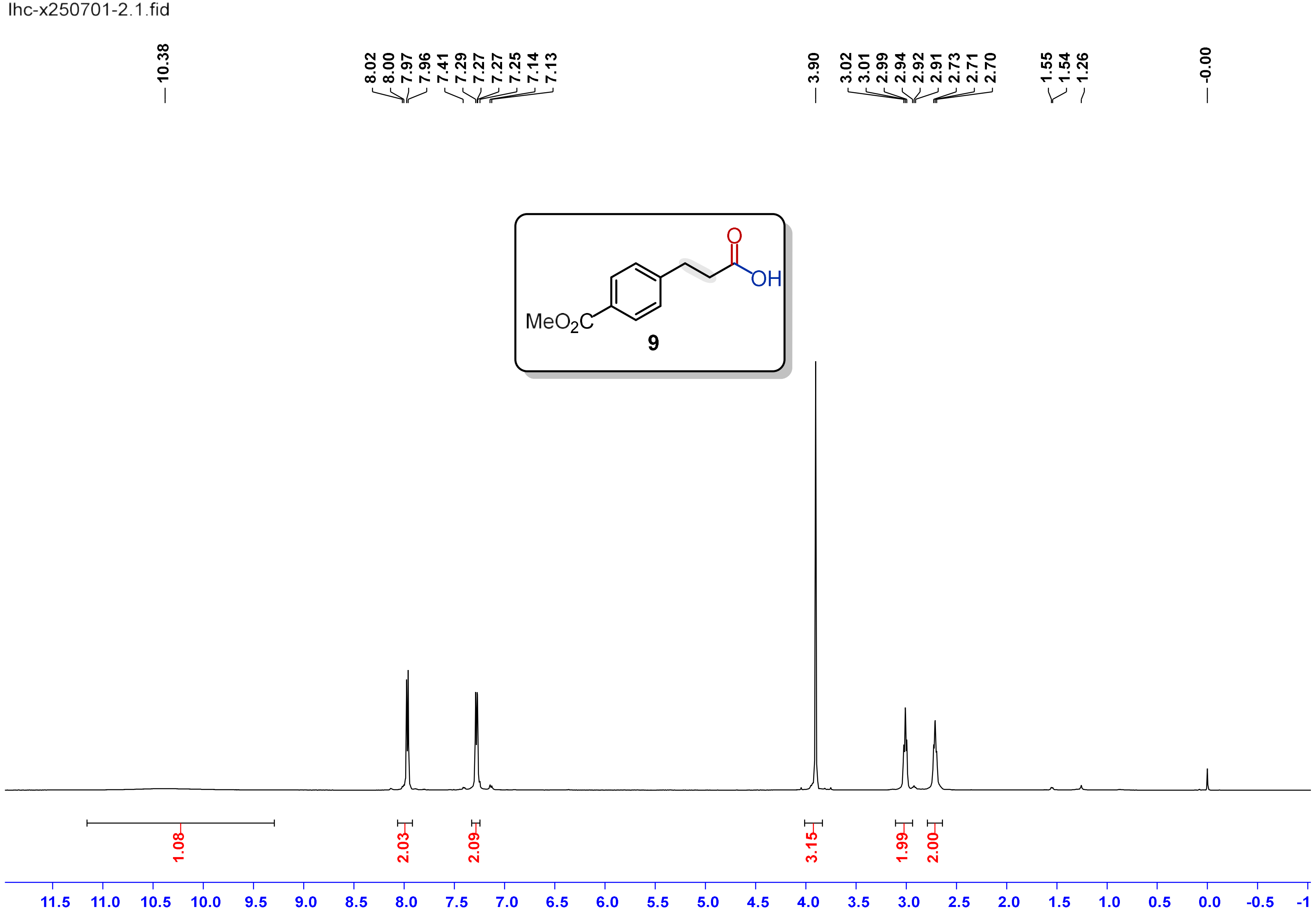

^{13}C NMR spectra for 9

lhc-x250701-2.2.fid

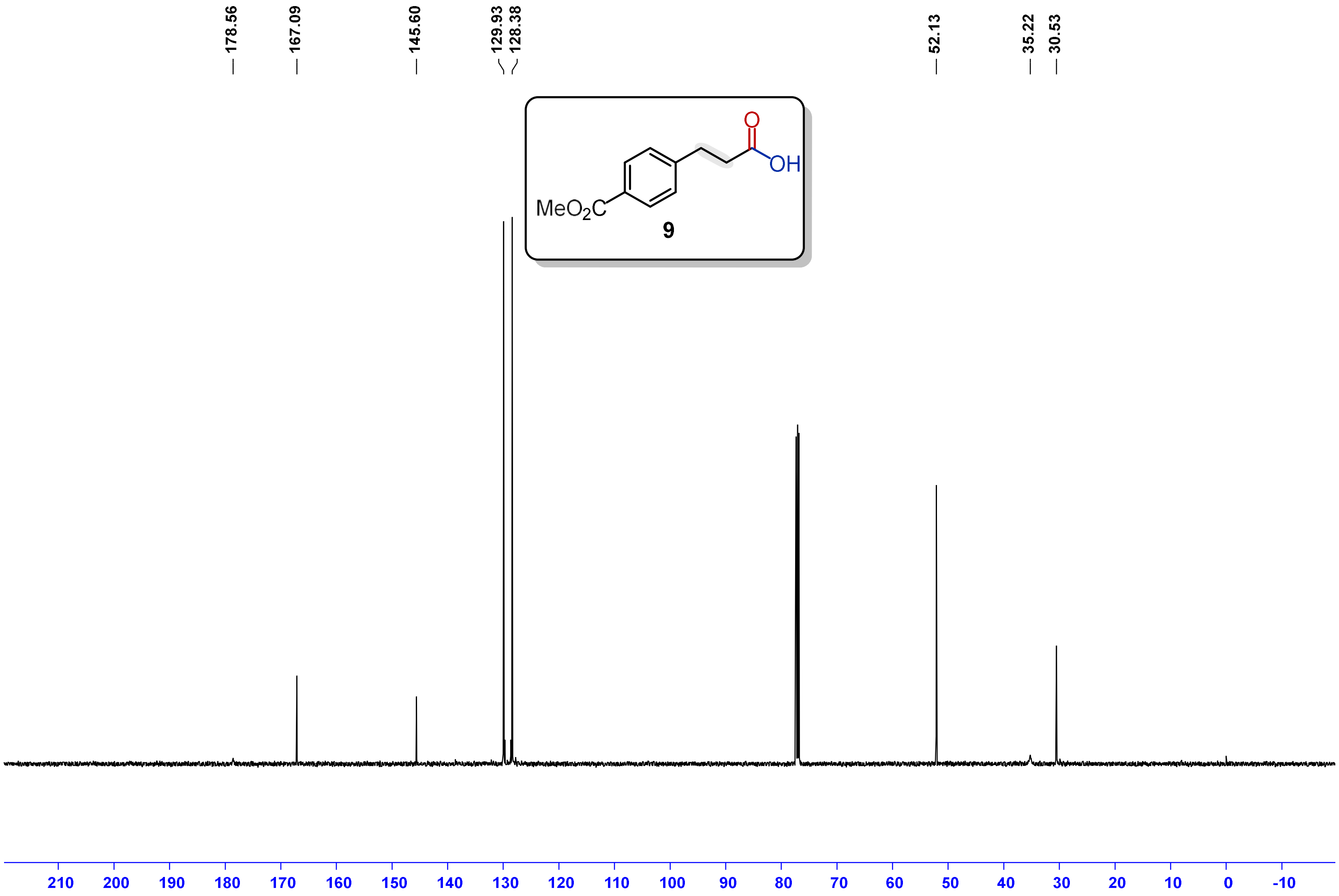

^{1}H NMR spectra for 10

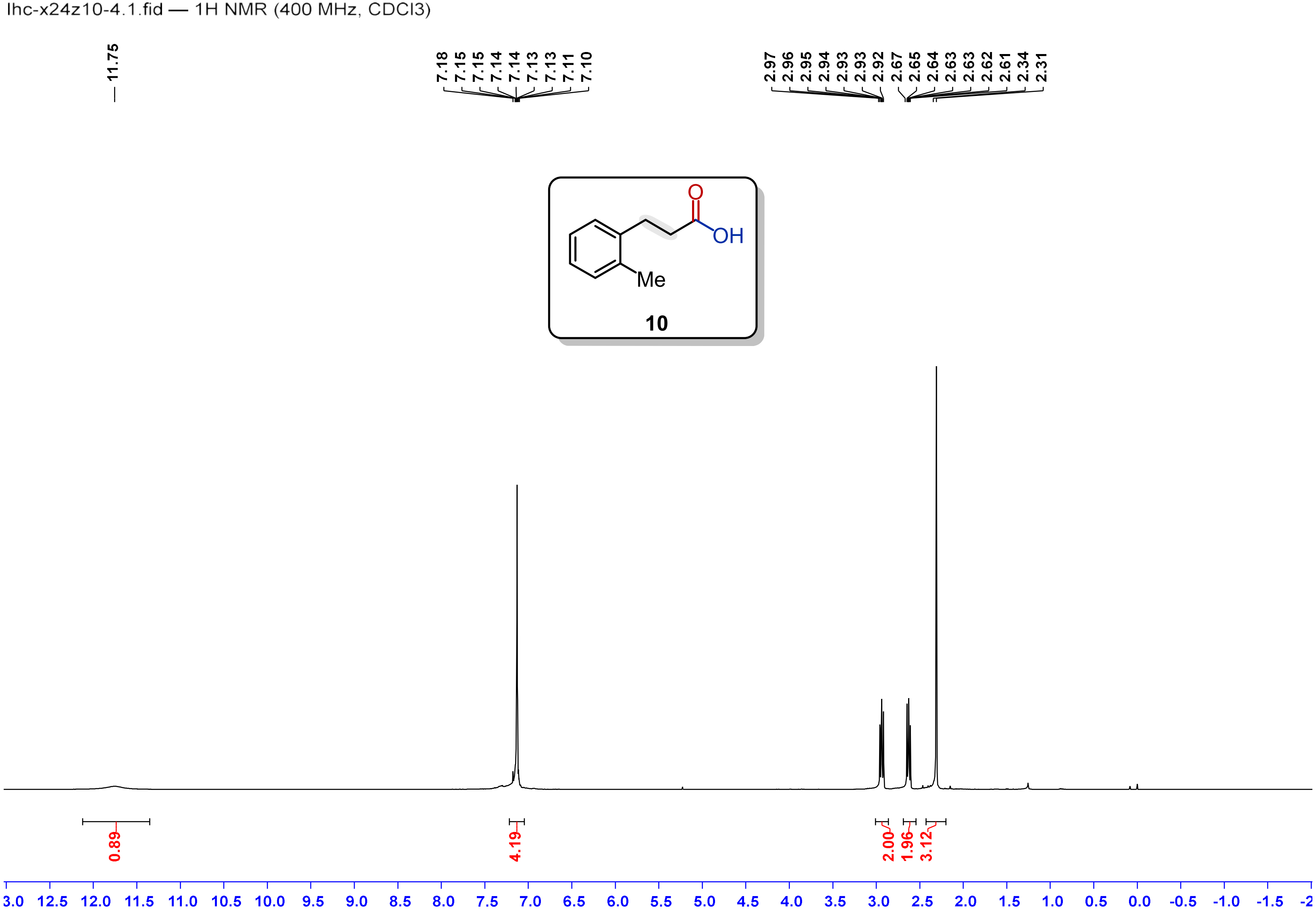

^{13}C NMR spectra for 10

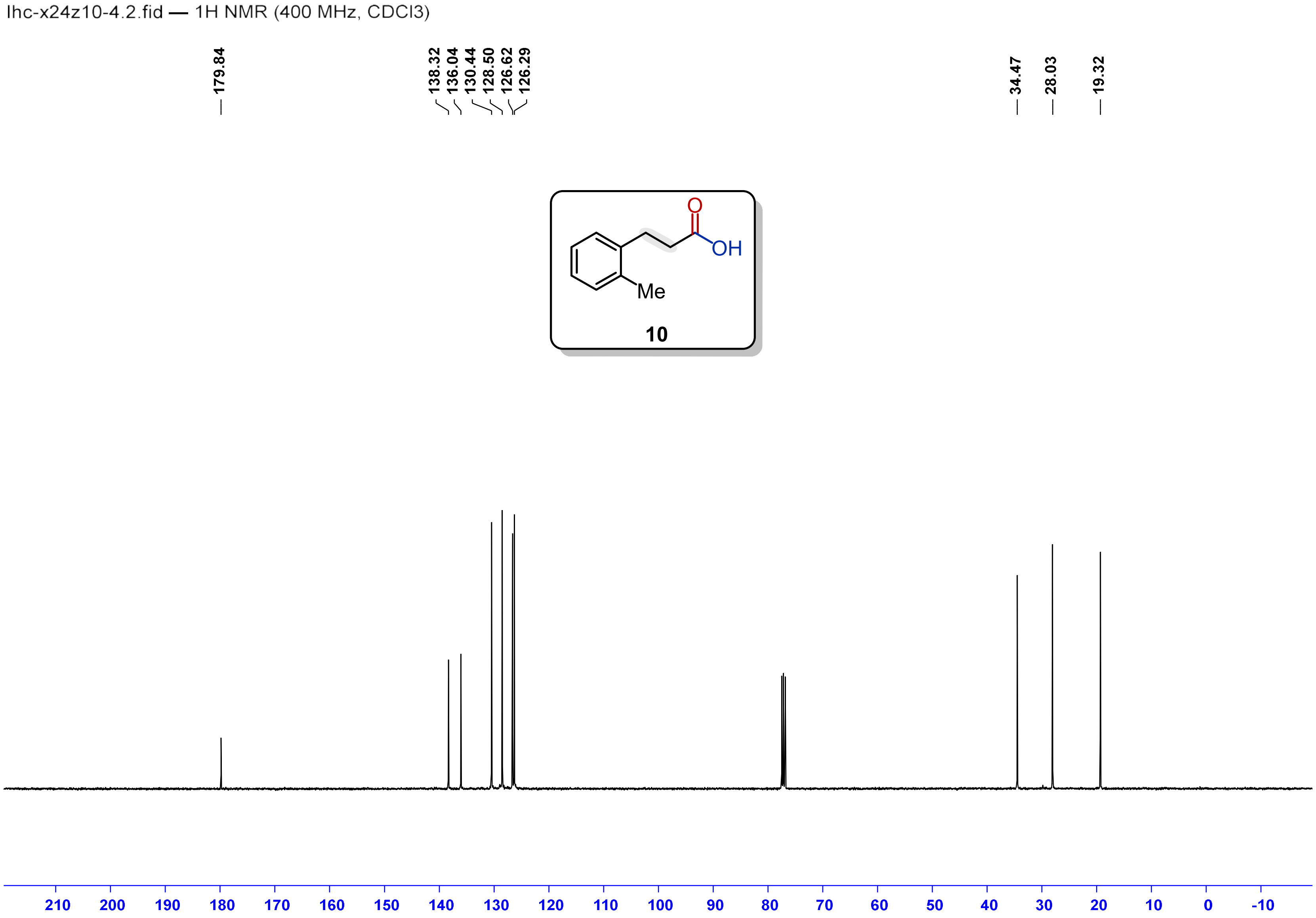

^{1}H NMR spectra for 11

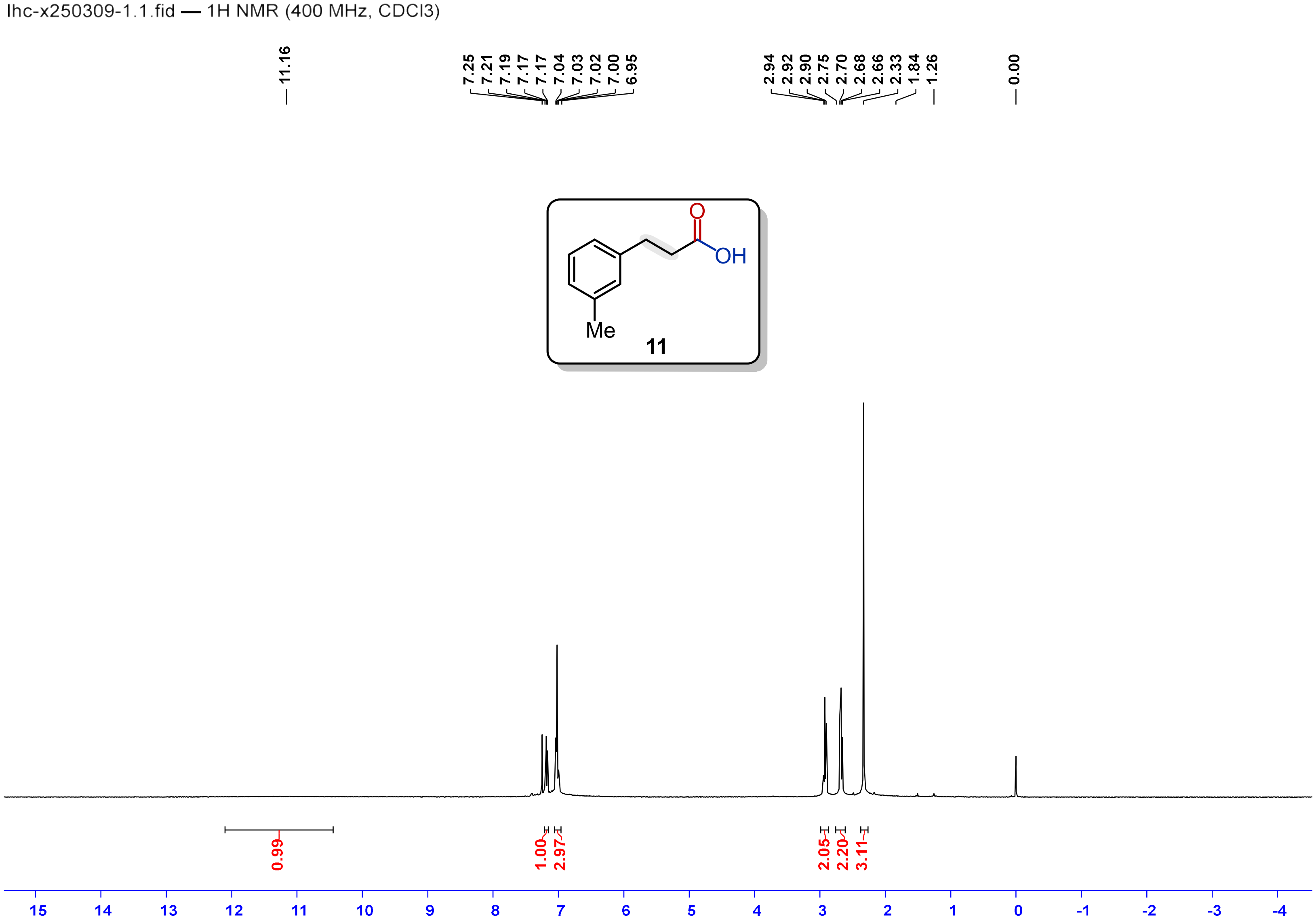

^{13}C NMR spectra for 11

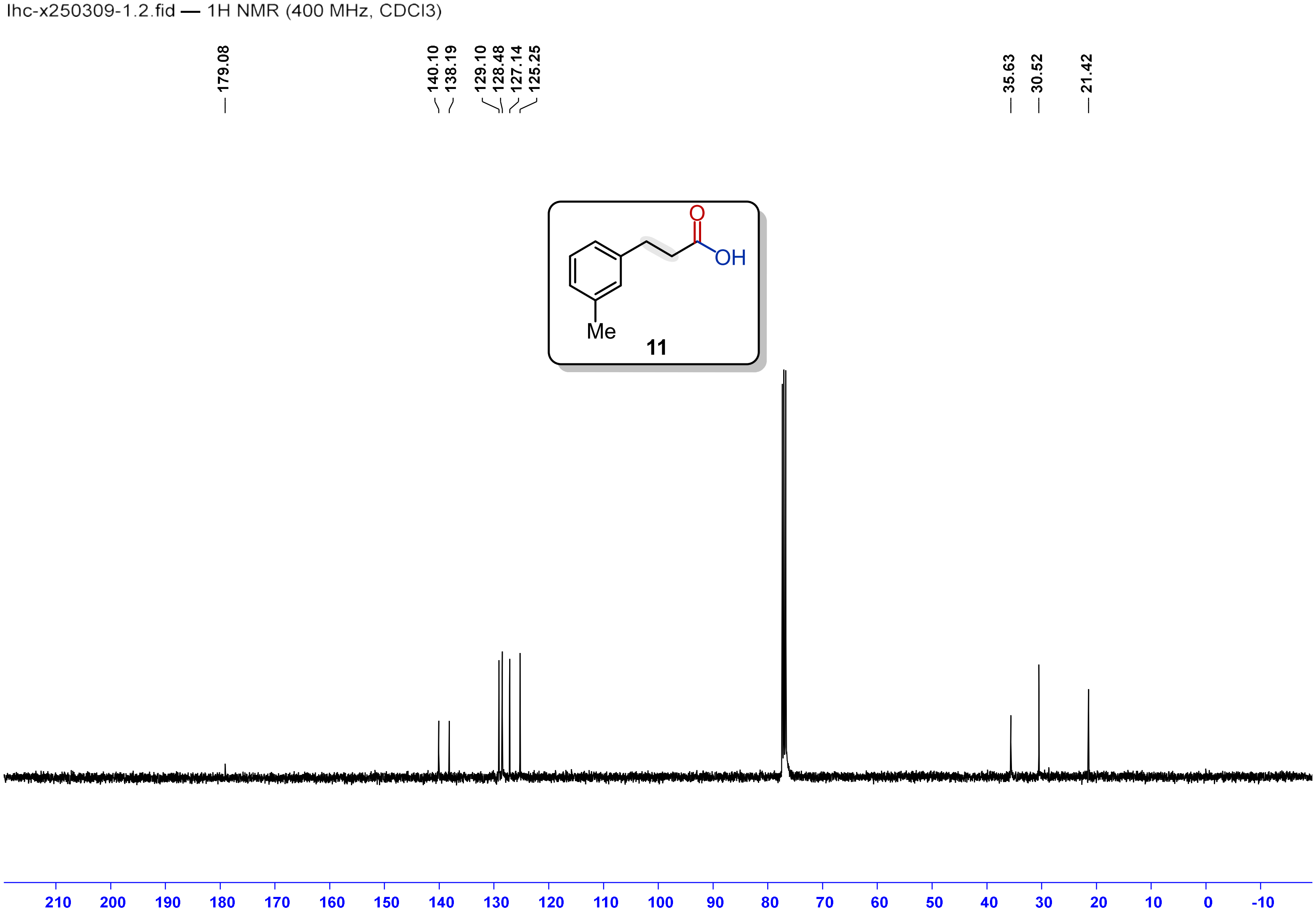

^{1}H NMR spectra for 12

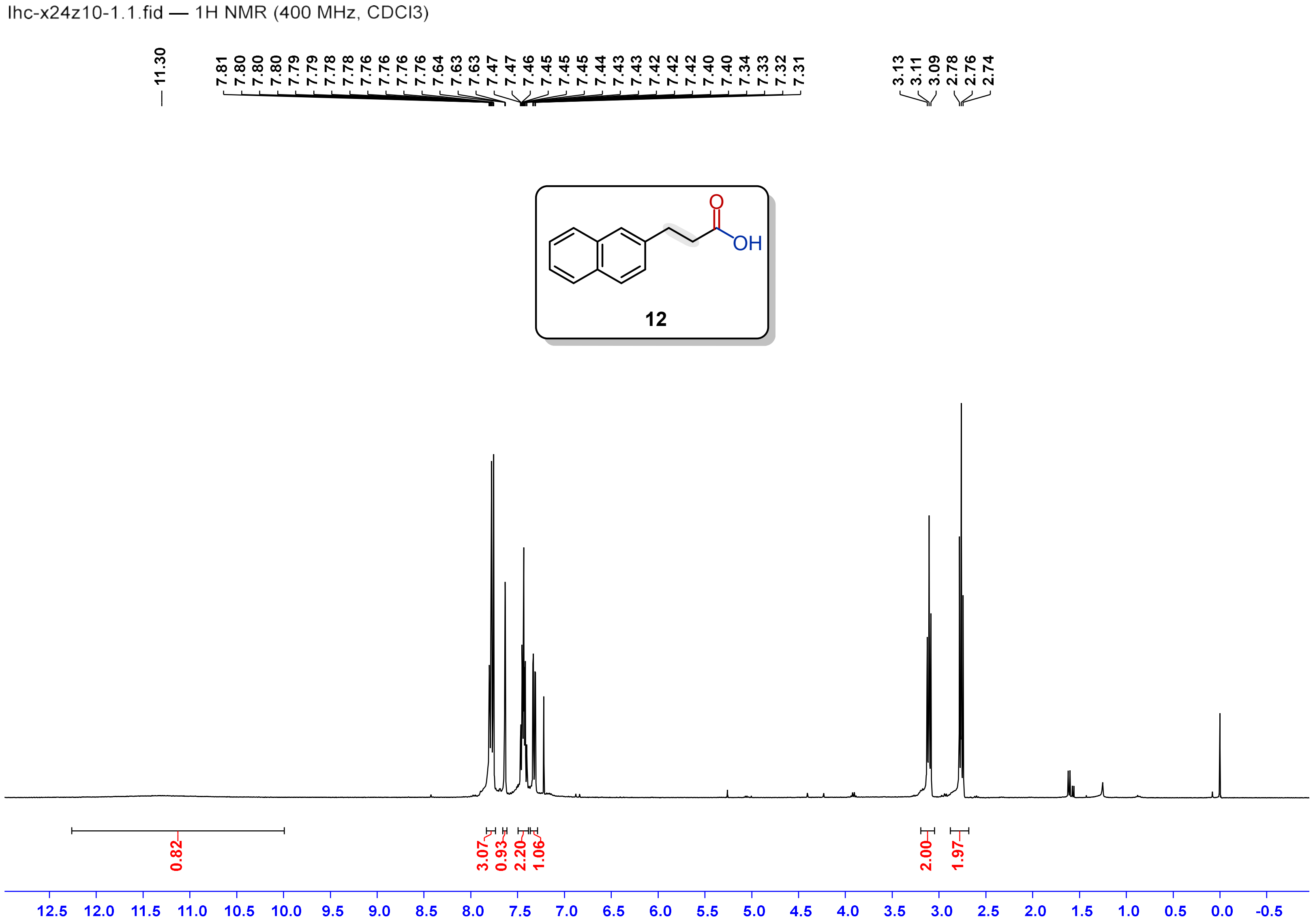

^{13}C NMR spectra for 12

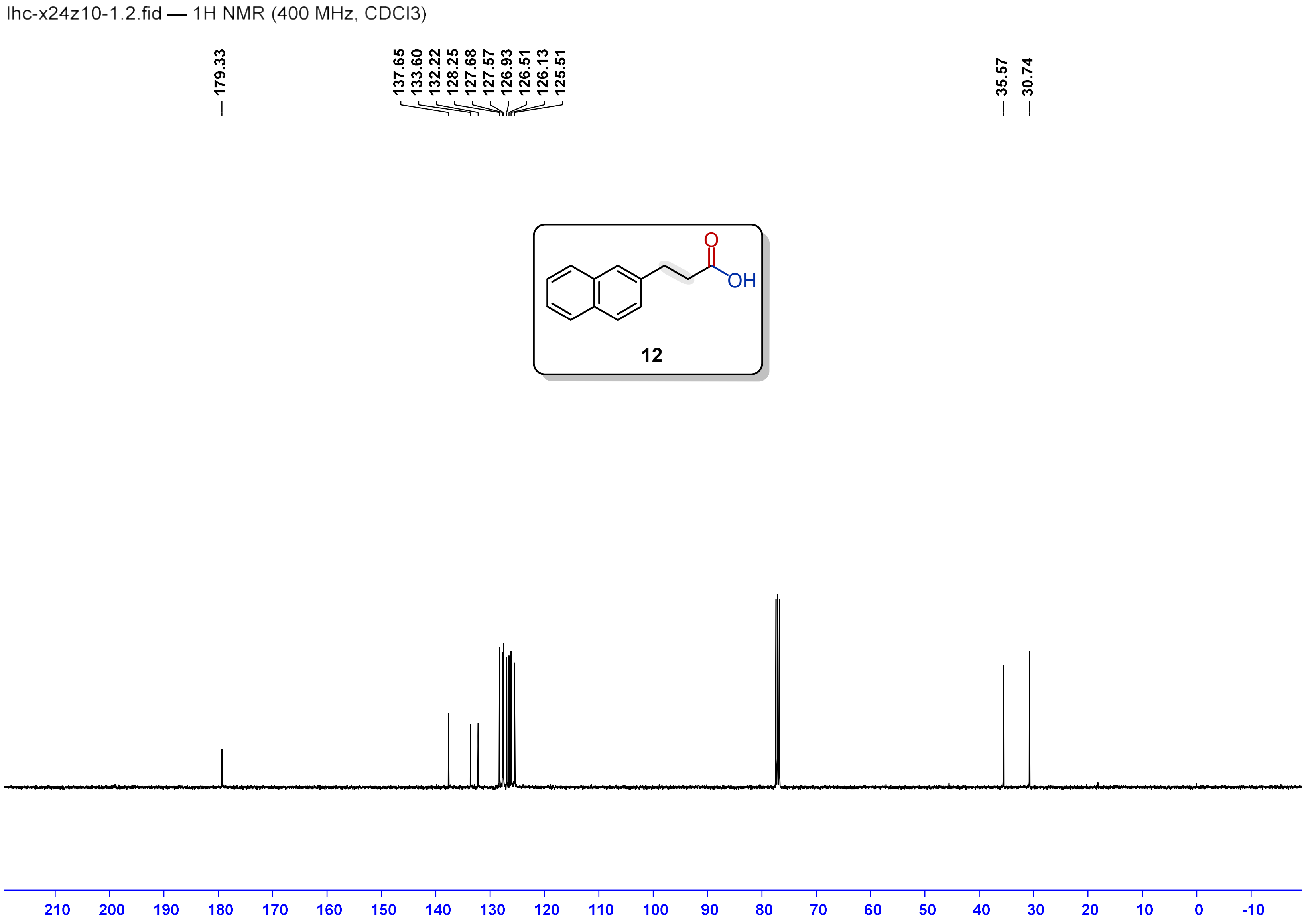

^{1}H NMR spectra for 13

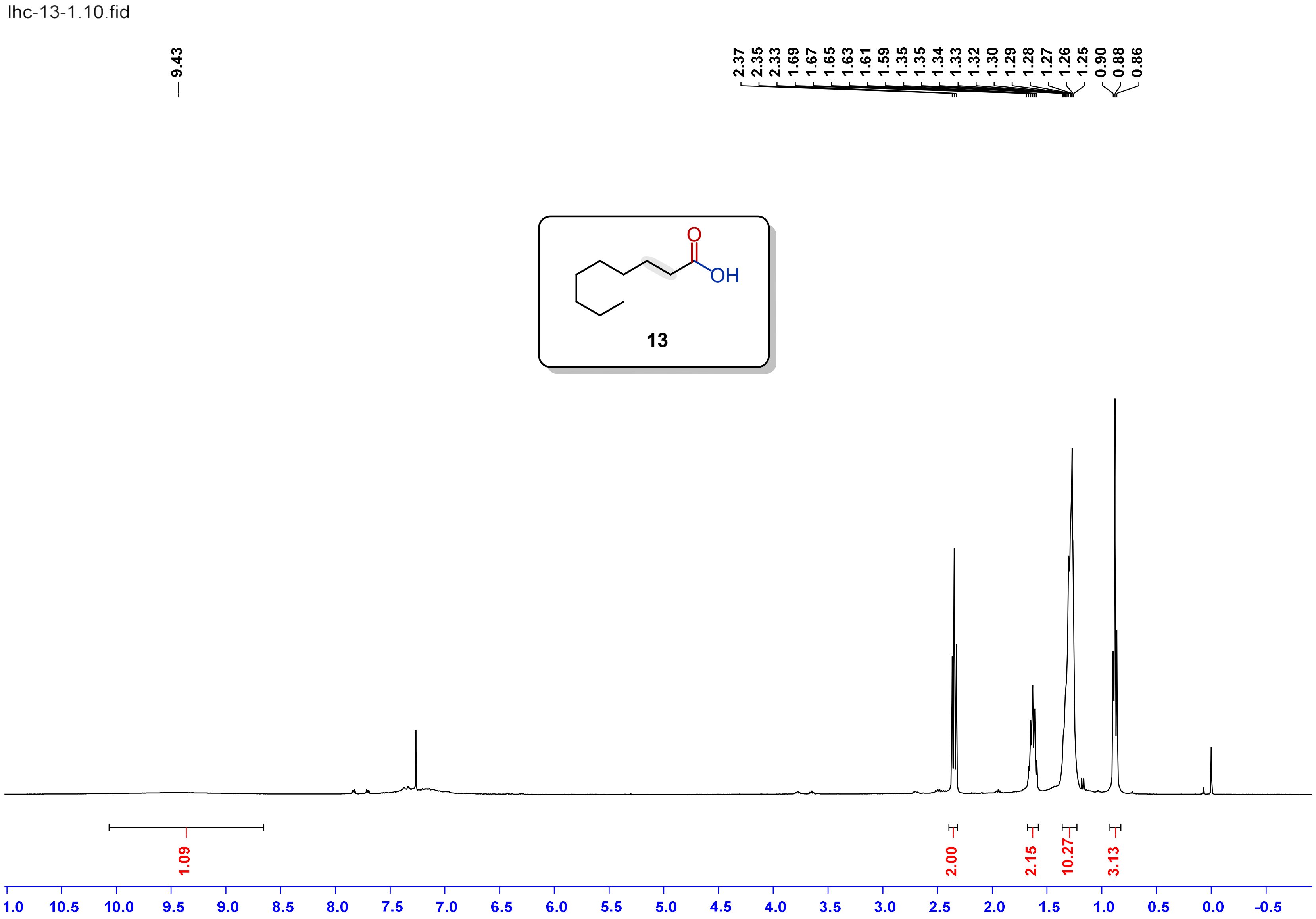

^{13}C NMR spectra for 13

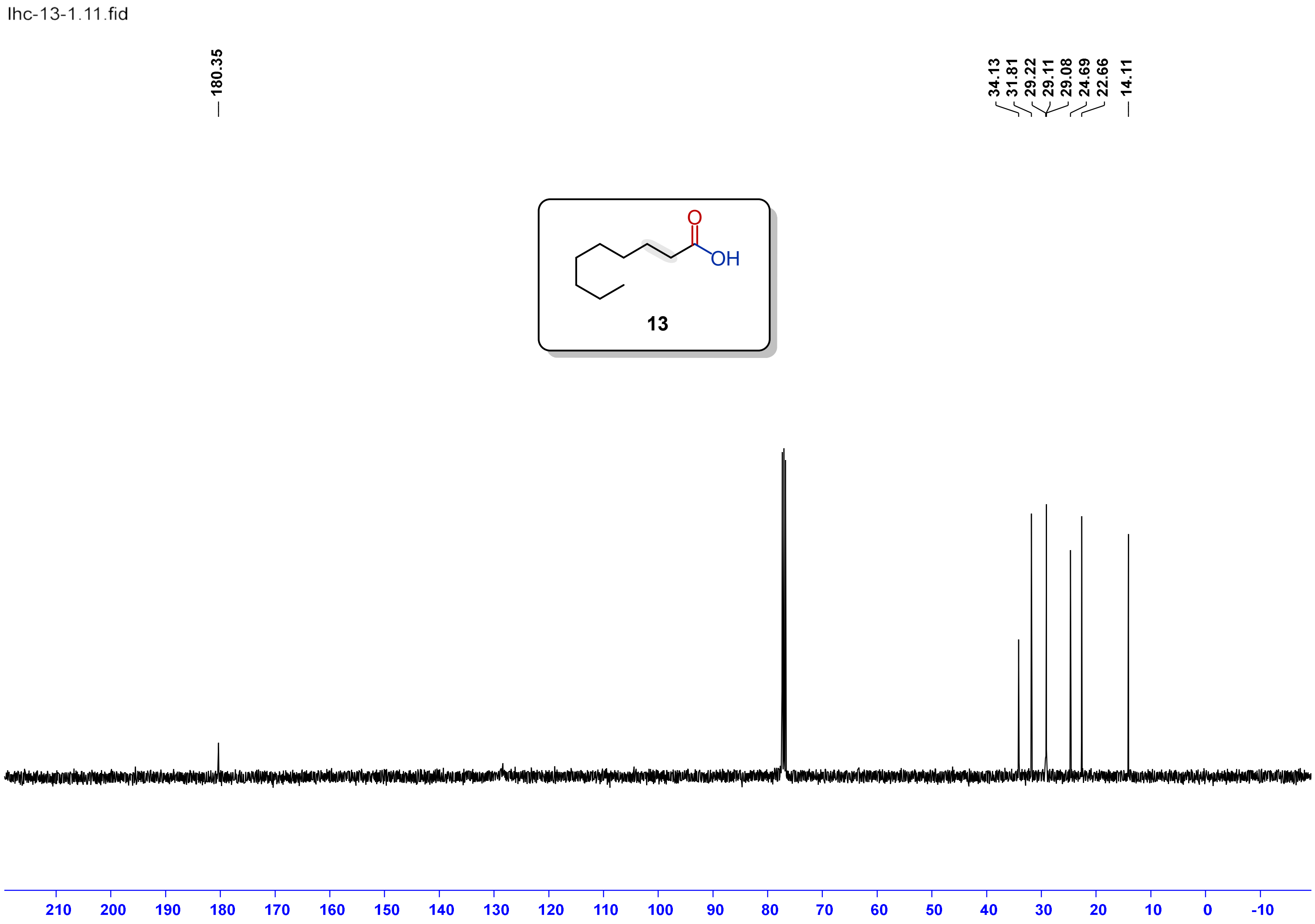

^{1}H NMR spectra for 14

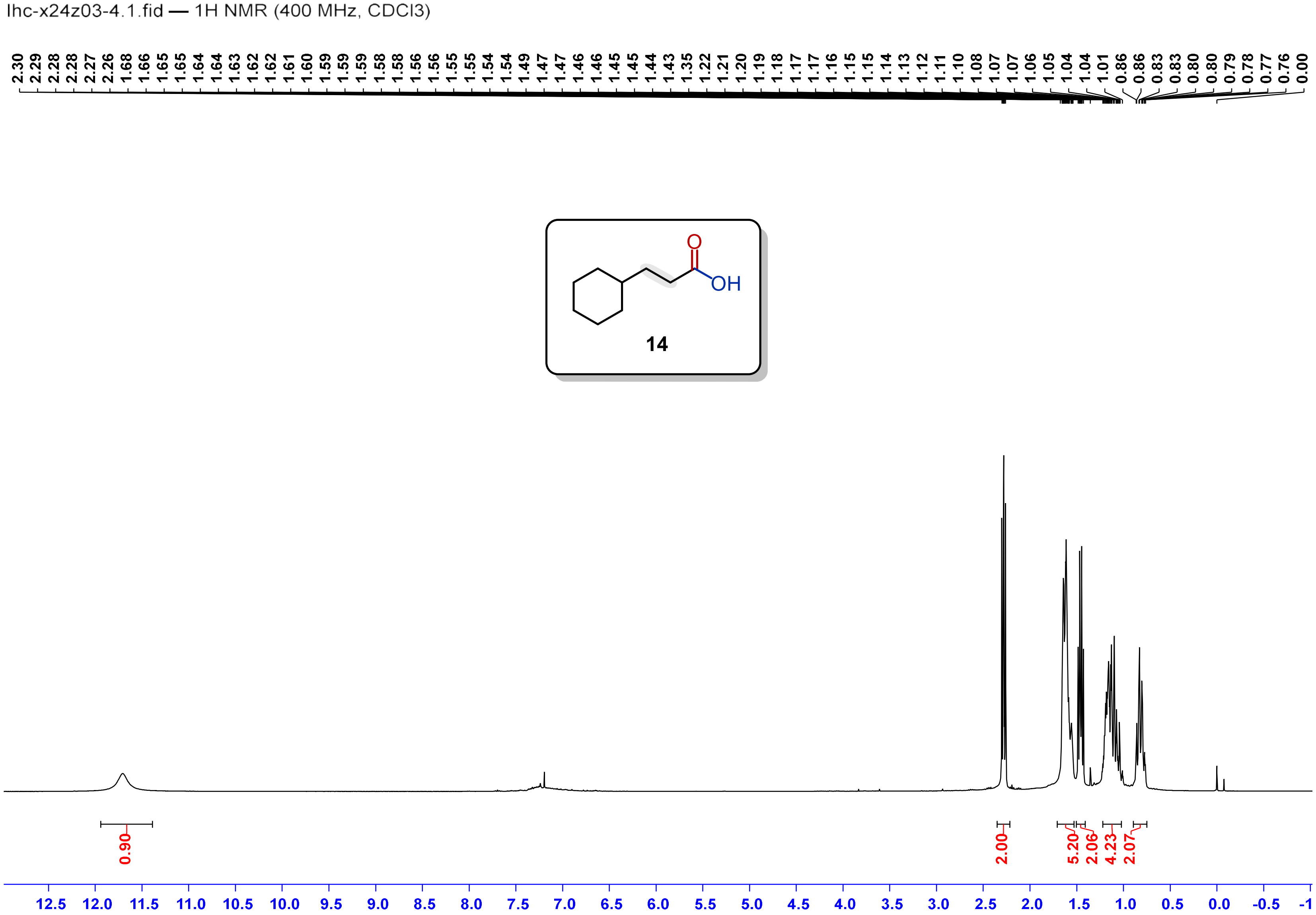

^{13}C NMR spectra for 14

lhc-14.2.fid

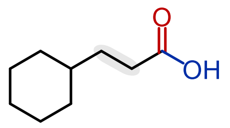

14

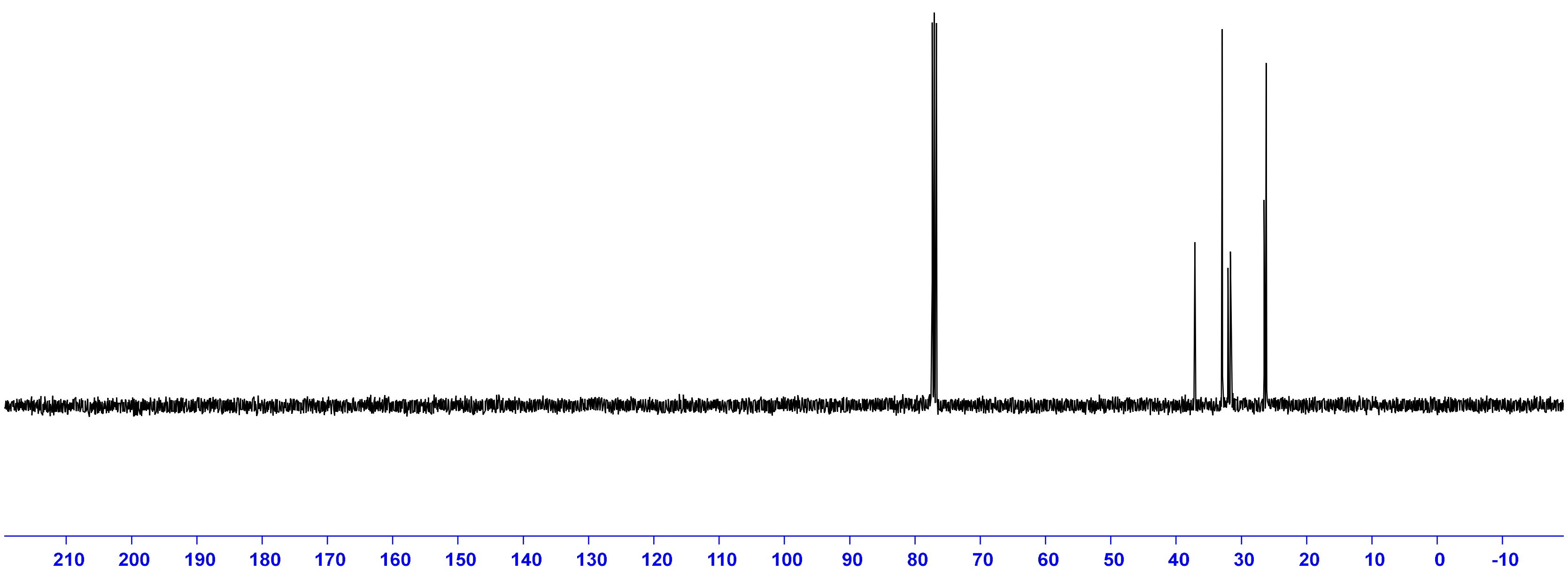

^{1}H NMR spectra for 15

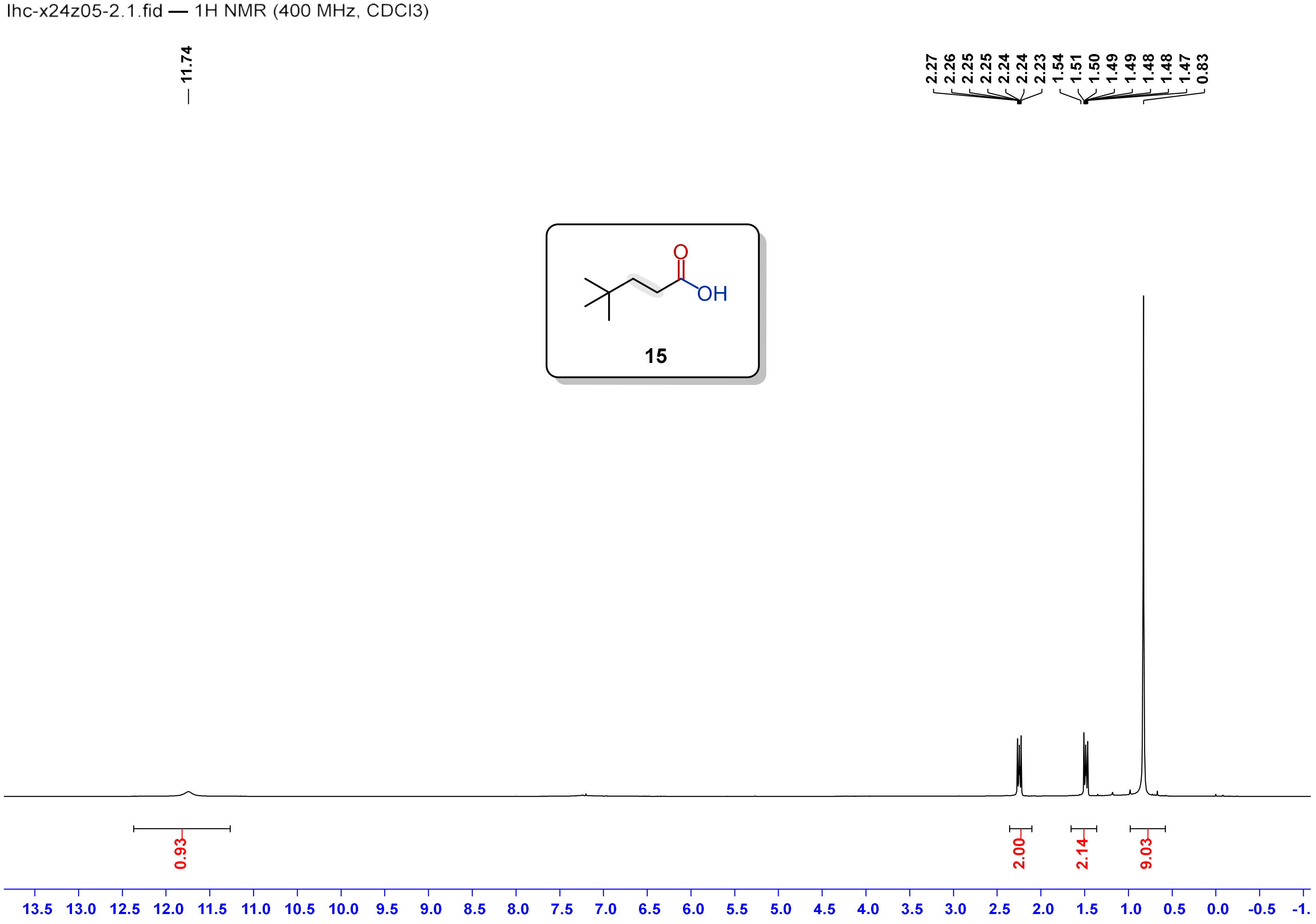

^{13}C NMR spectra for 15

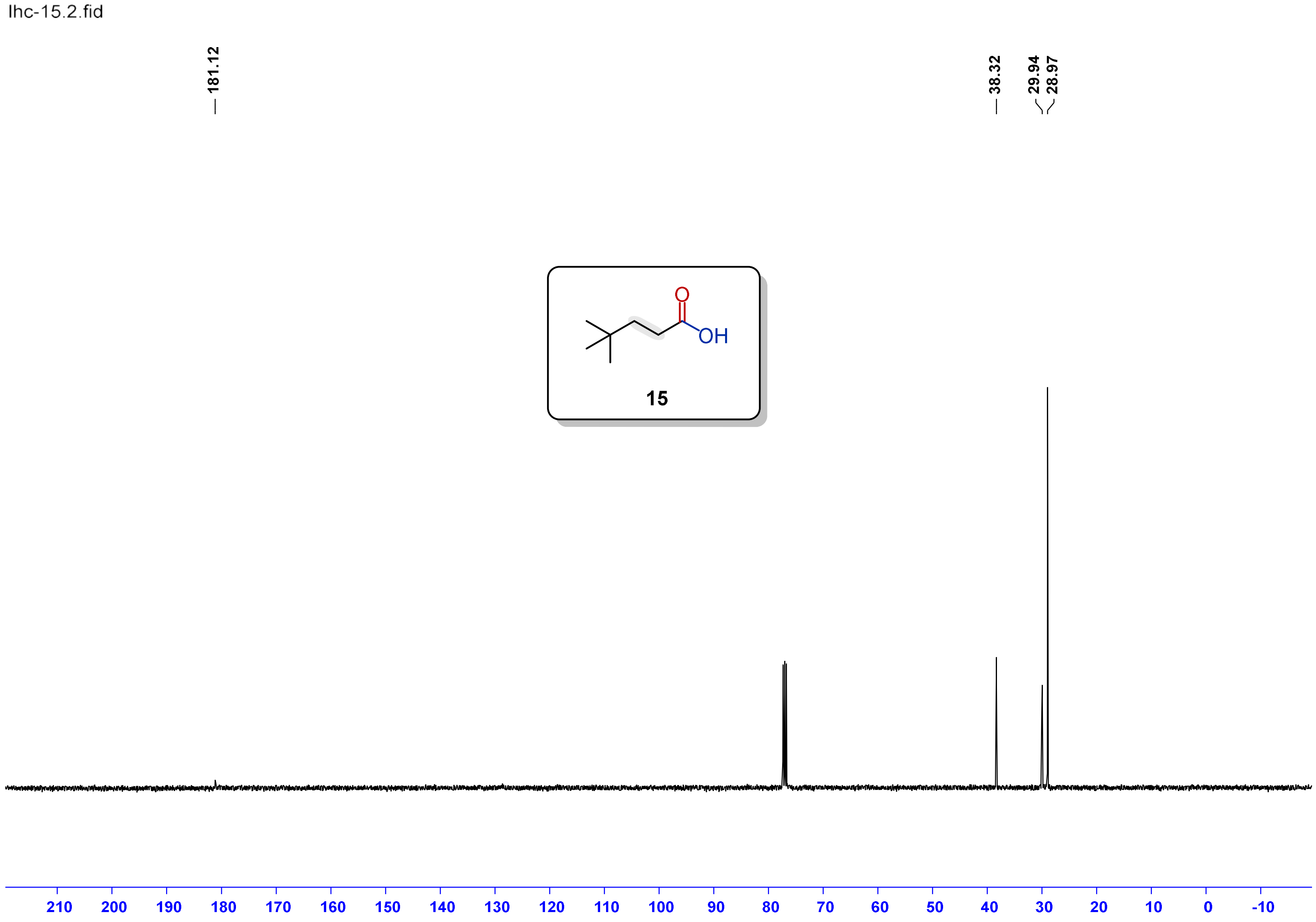

^{1}H NMR spectra for 16

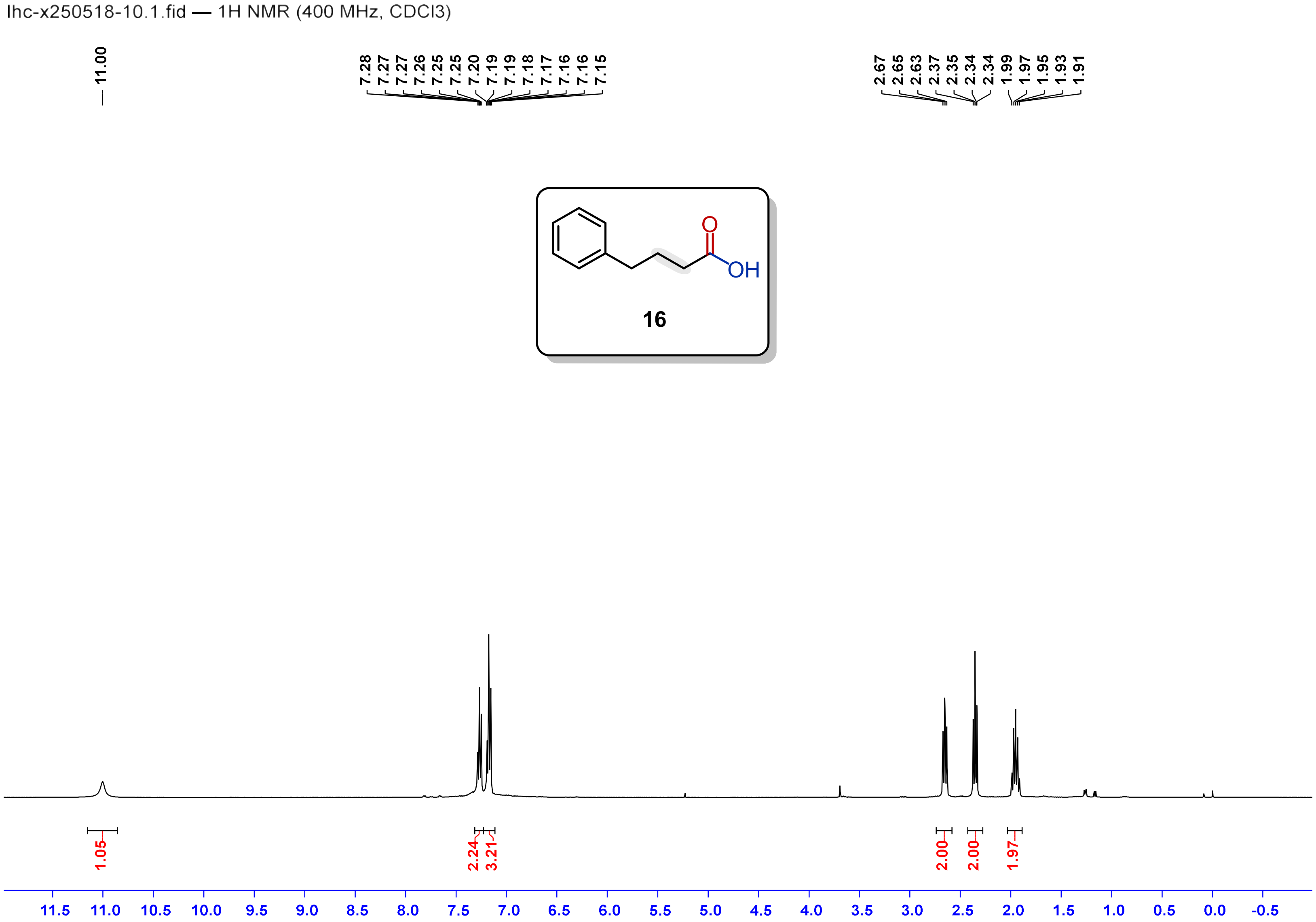

^{13}C NMR spectra for 16

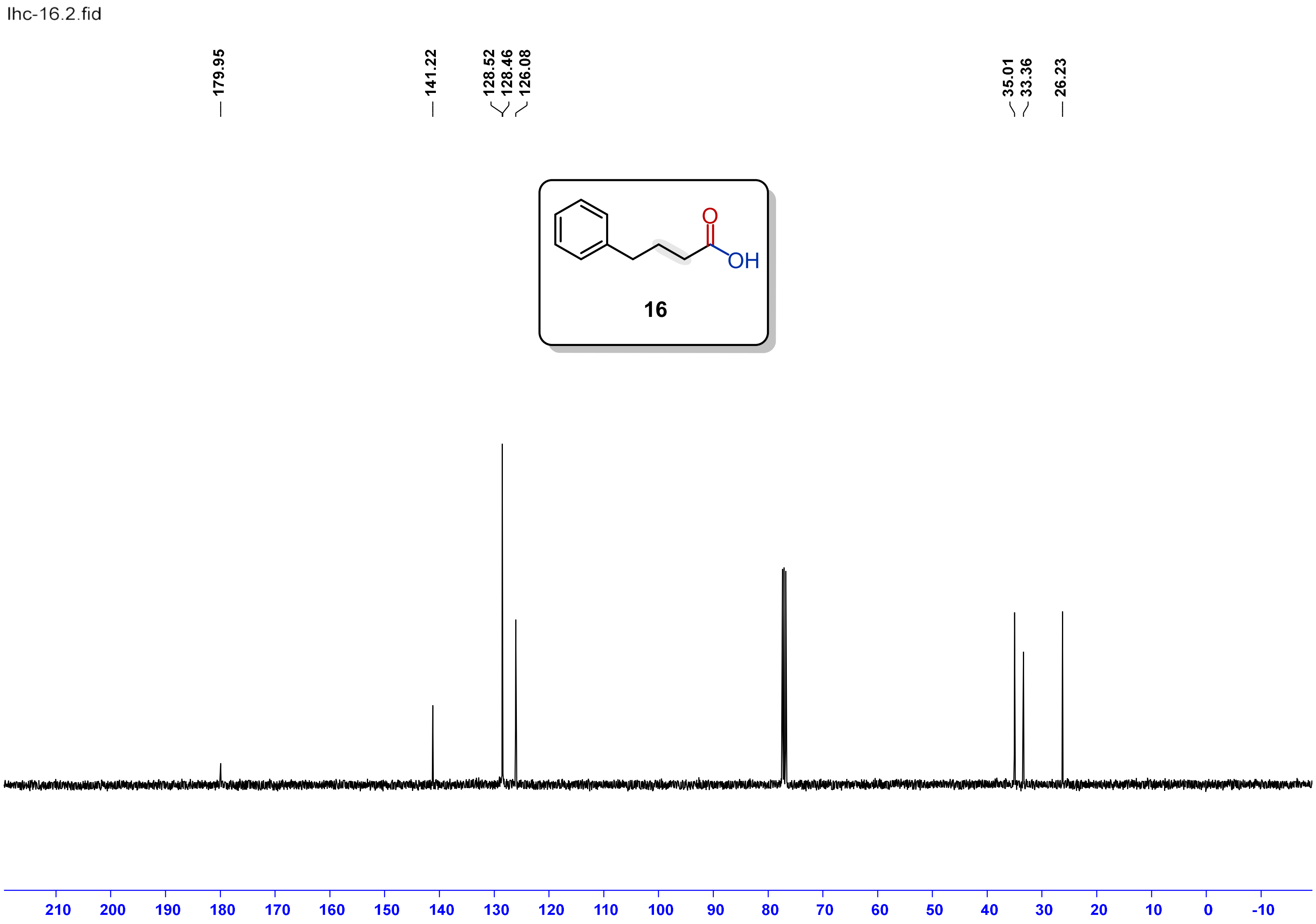

^{1}H NMR spectra for 17

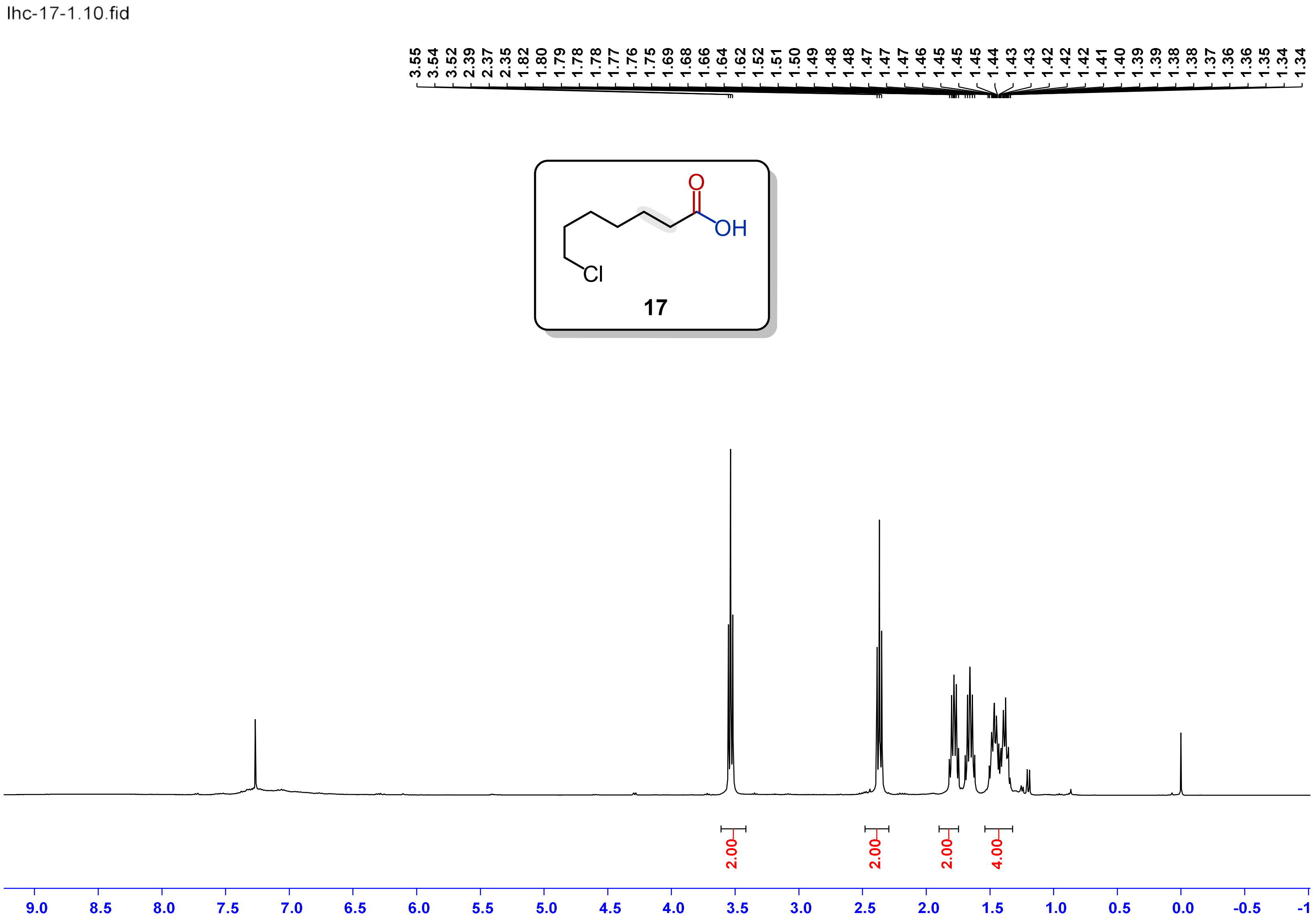

^{13}C NMR spectra for 17

lhc-17-1.11.fid

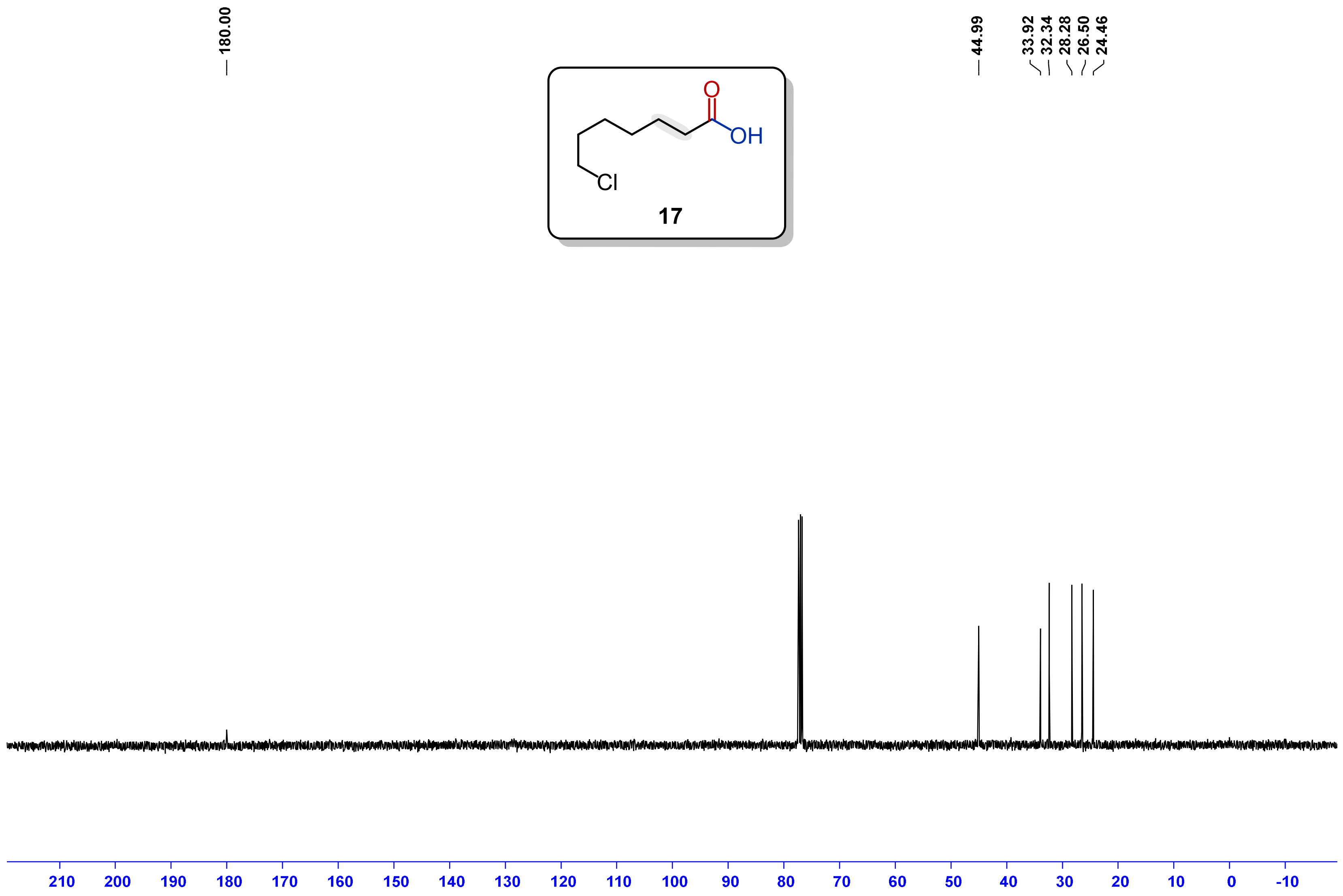

^{1}H NMR spectra for 18

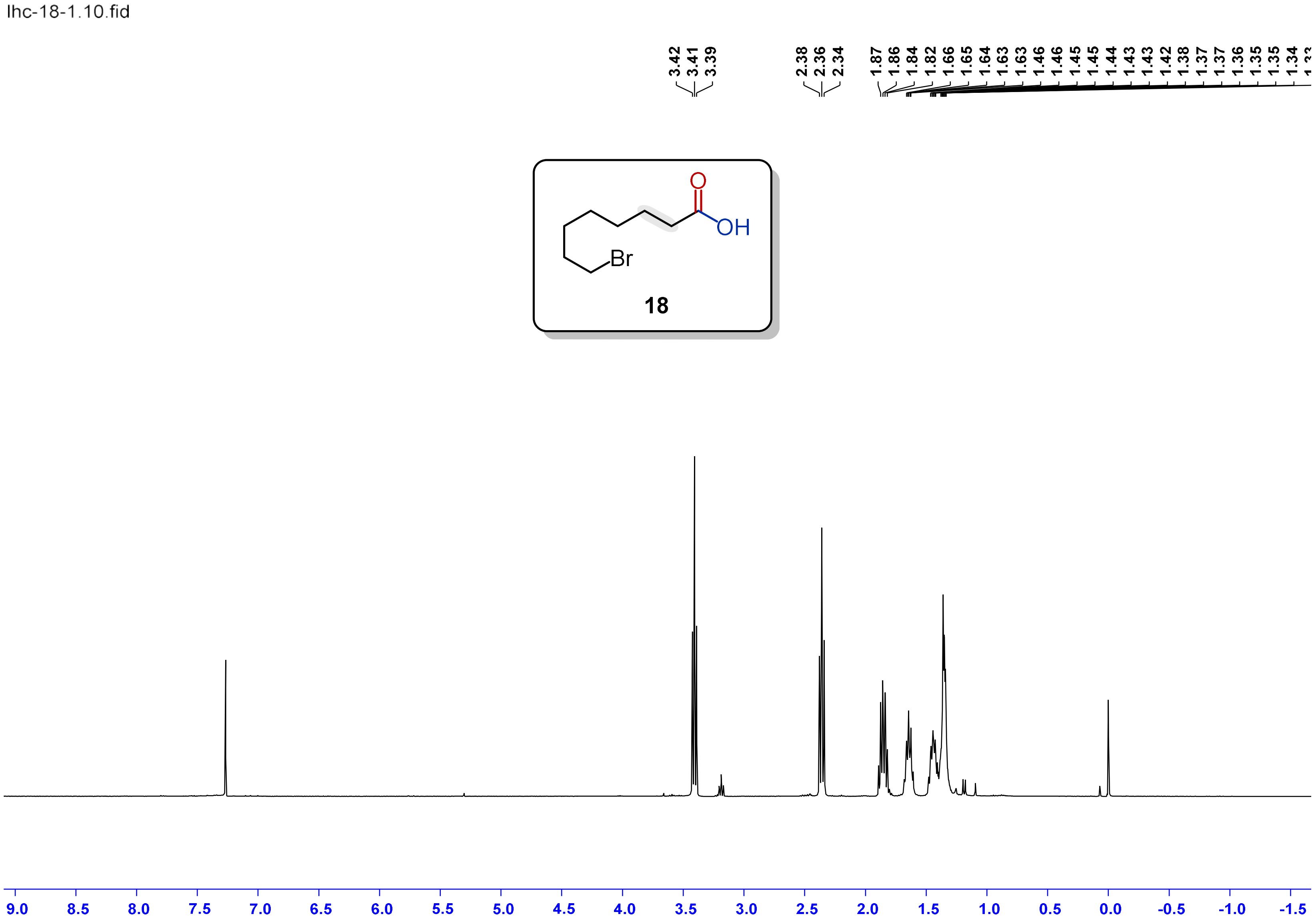

^{13}C NMR spectra for 18

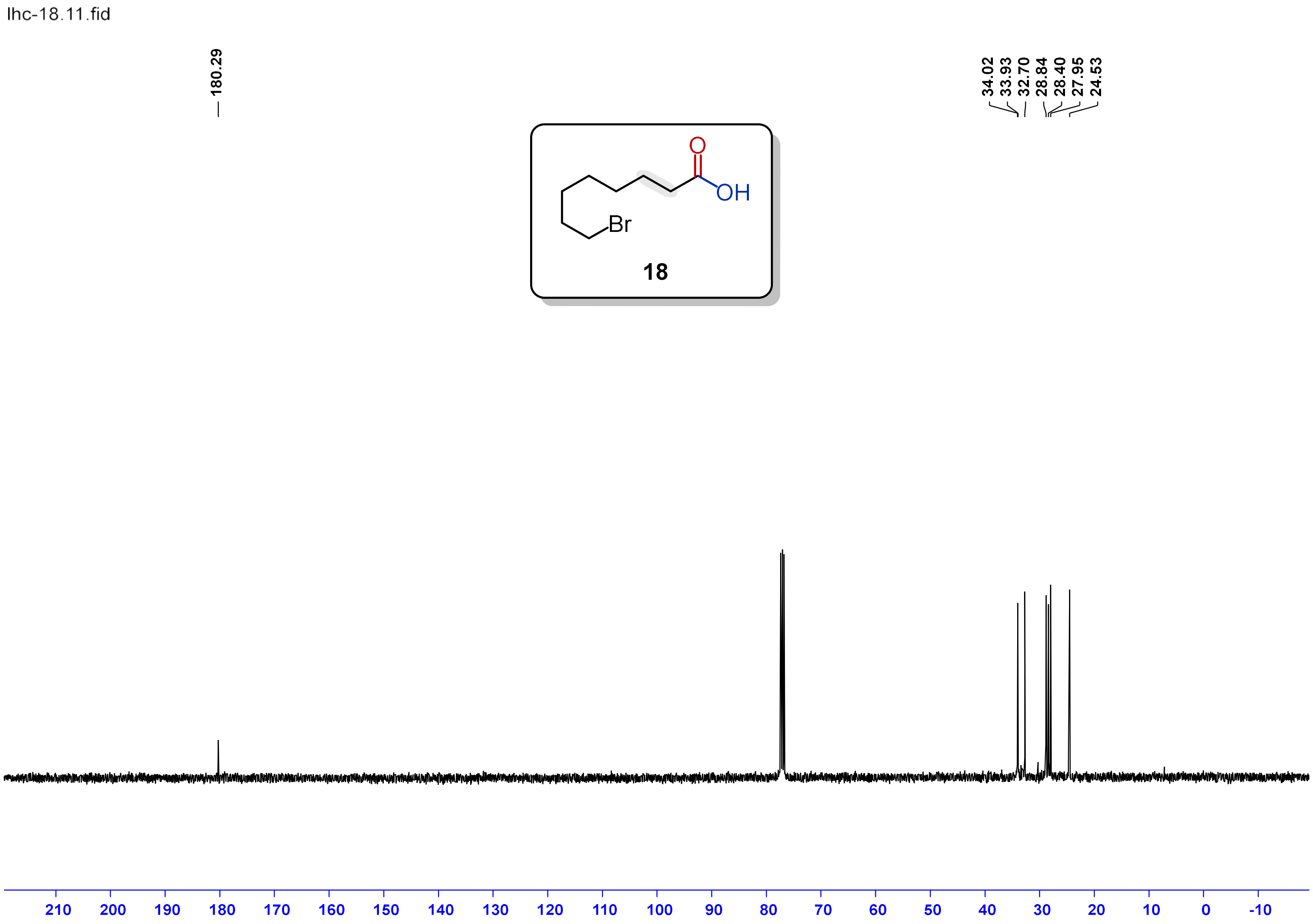

^{1}H NMR spectra for 19

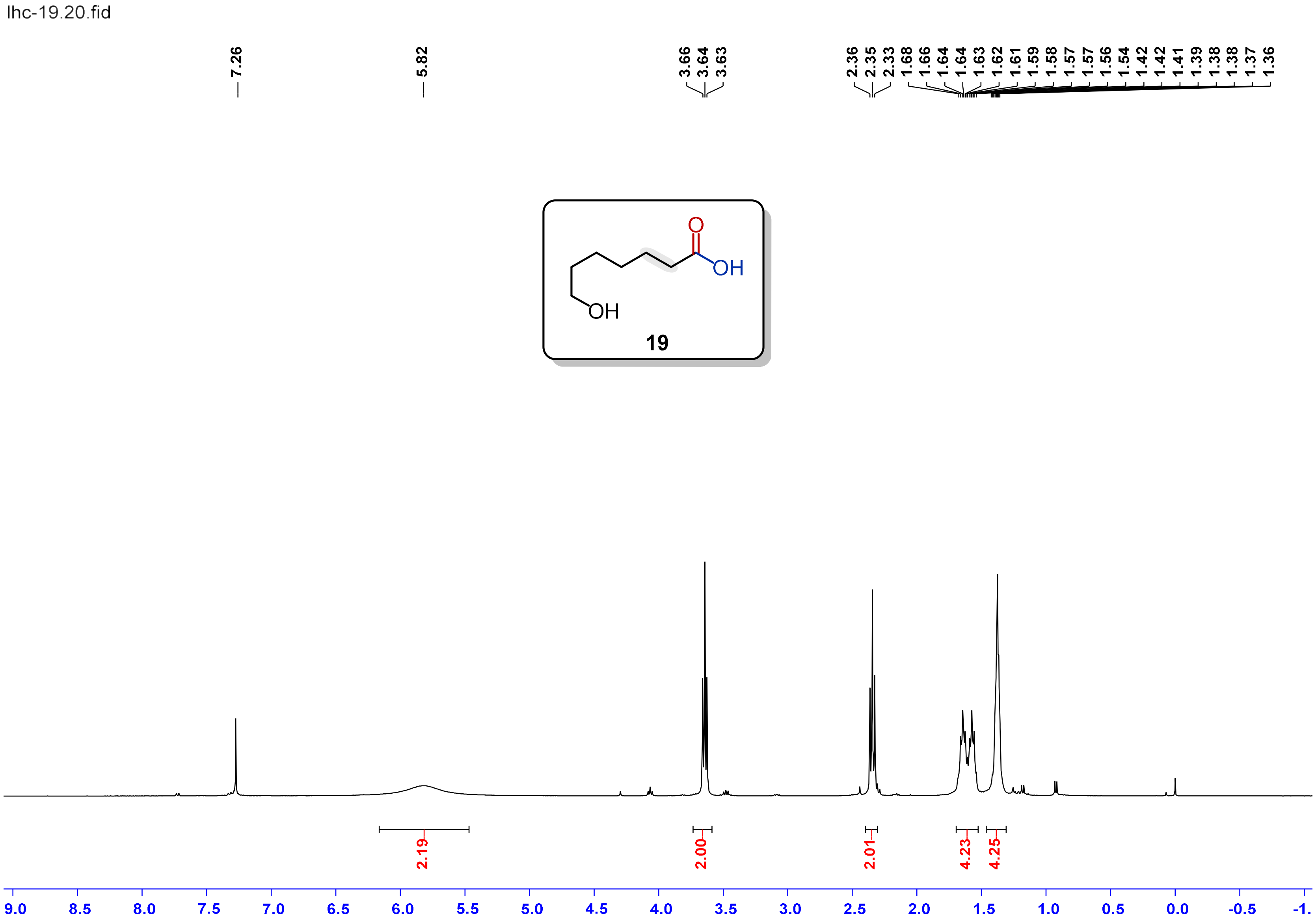

^{13}C NMR spectra for 19

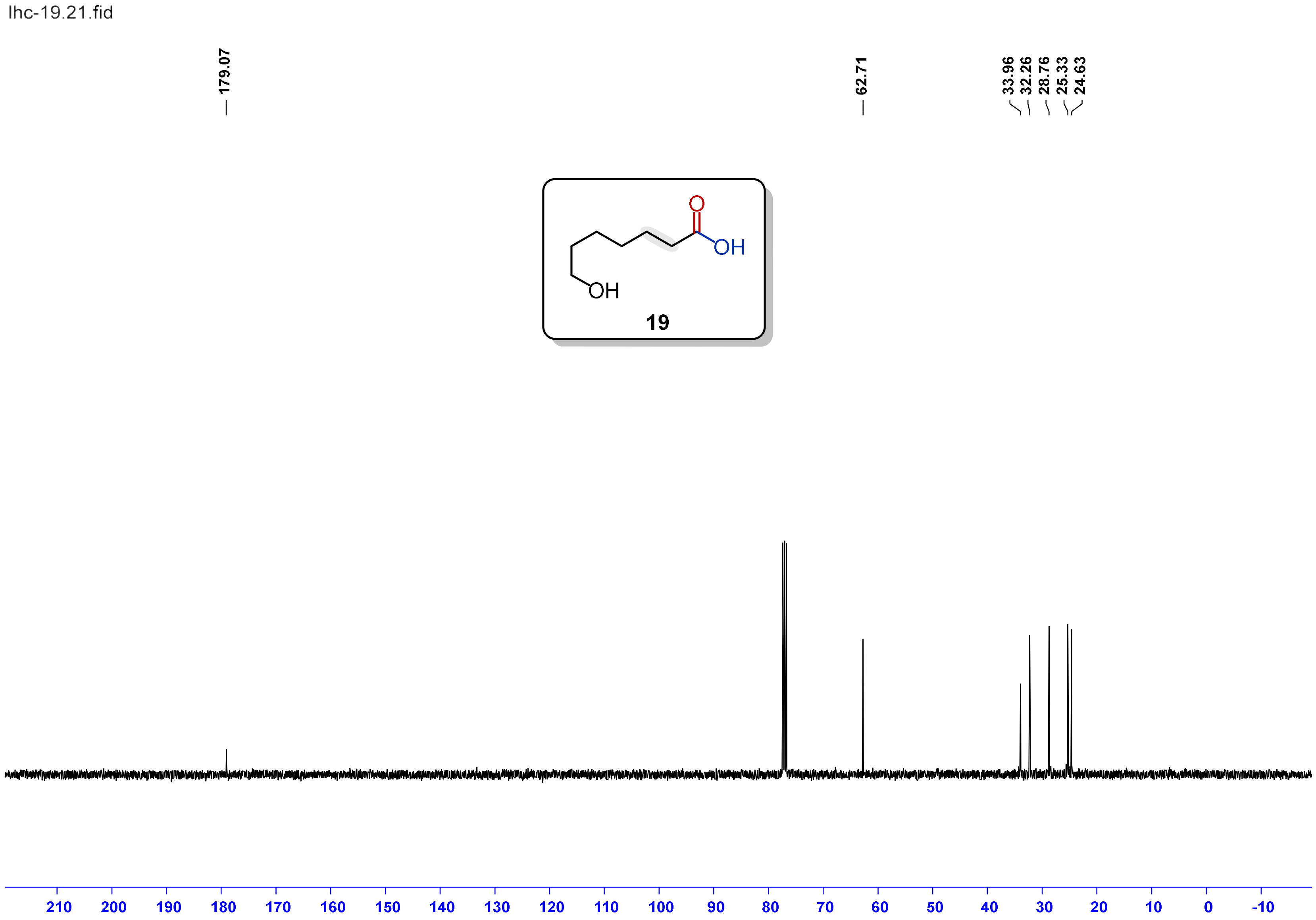

^{1}H NMR spectra for 20

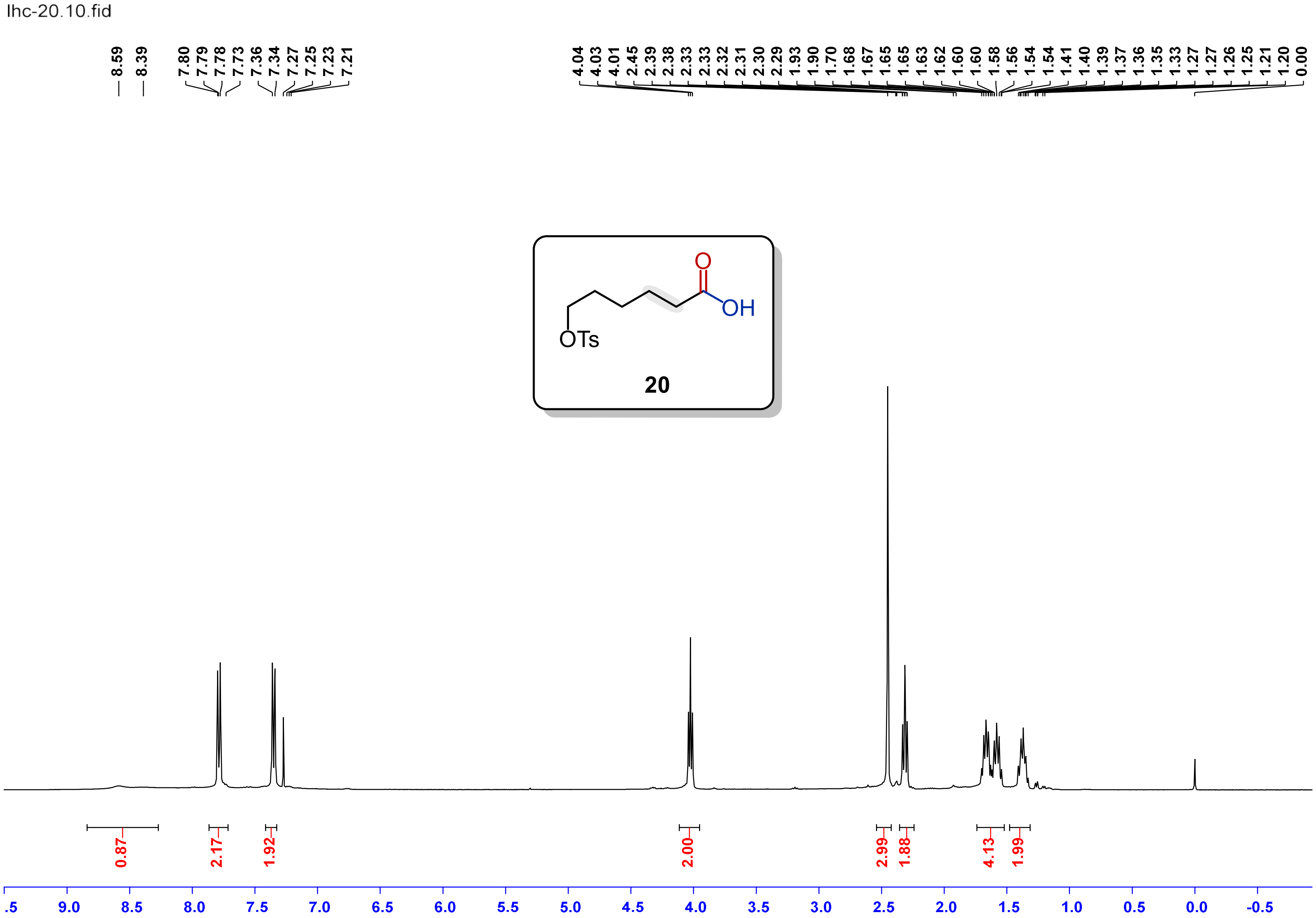

^{13}C NMR spectra for 20

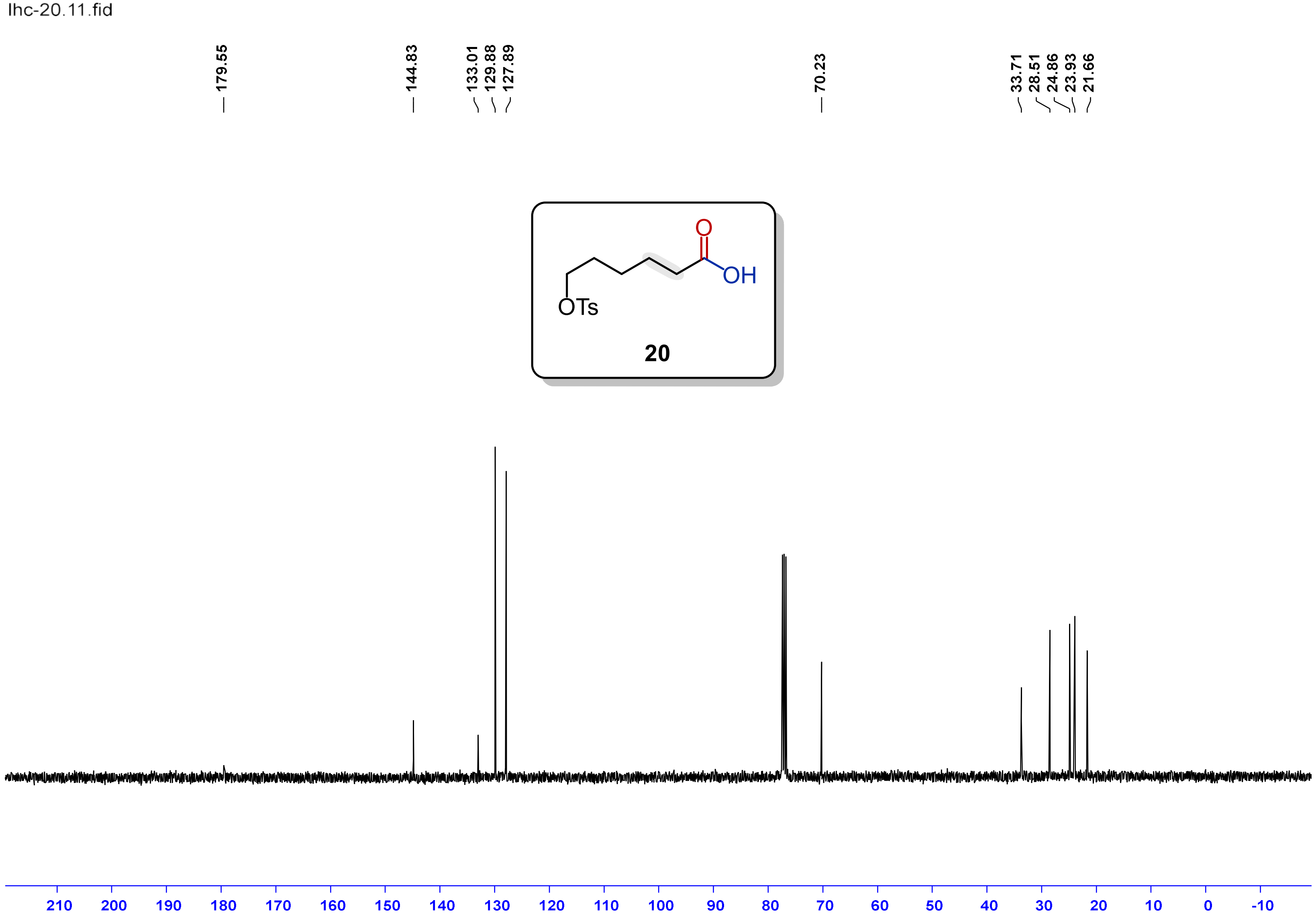

^{1}H NMR spectra for 21

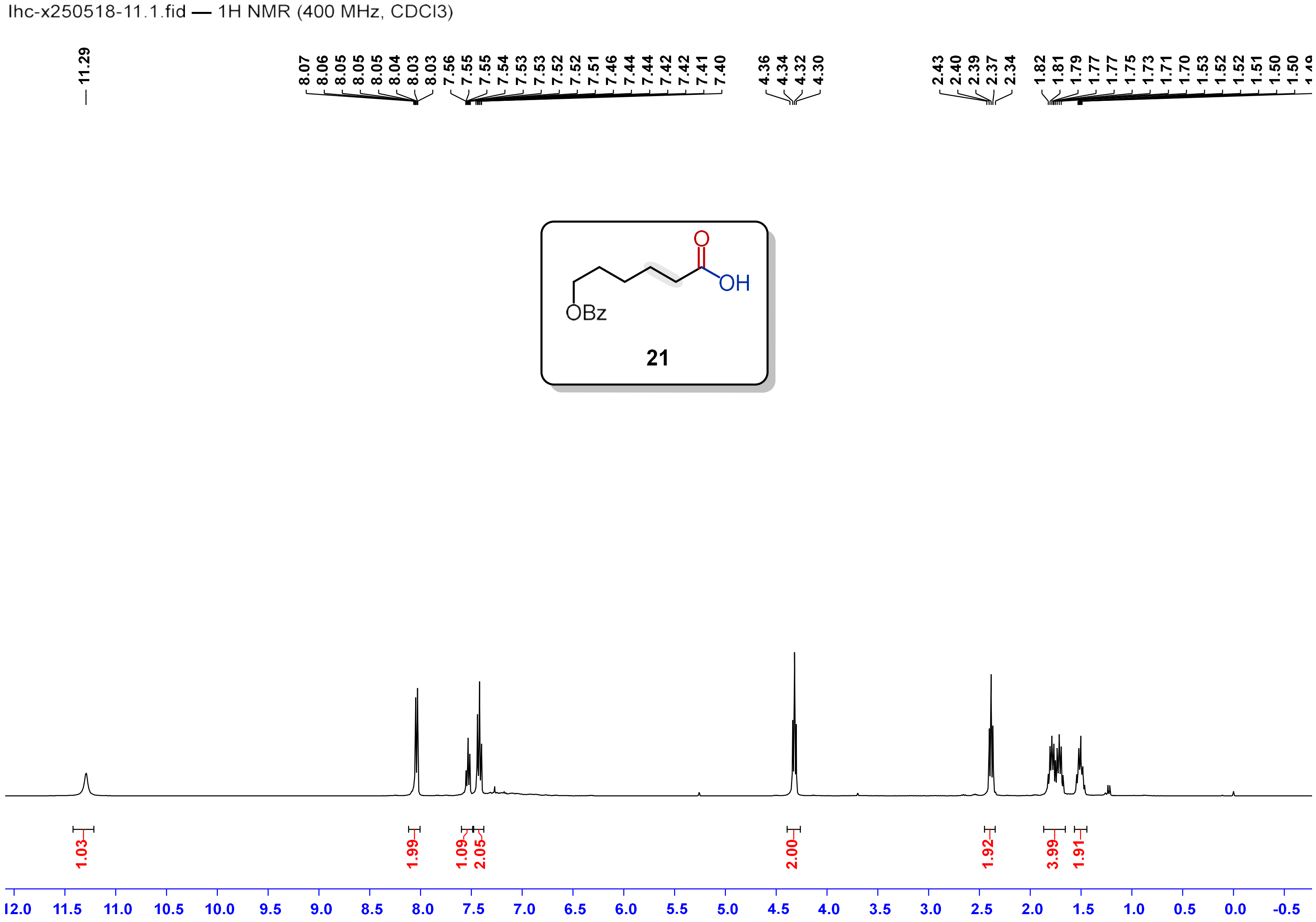

^{13}C NMR spectra for 21

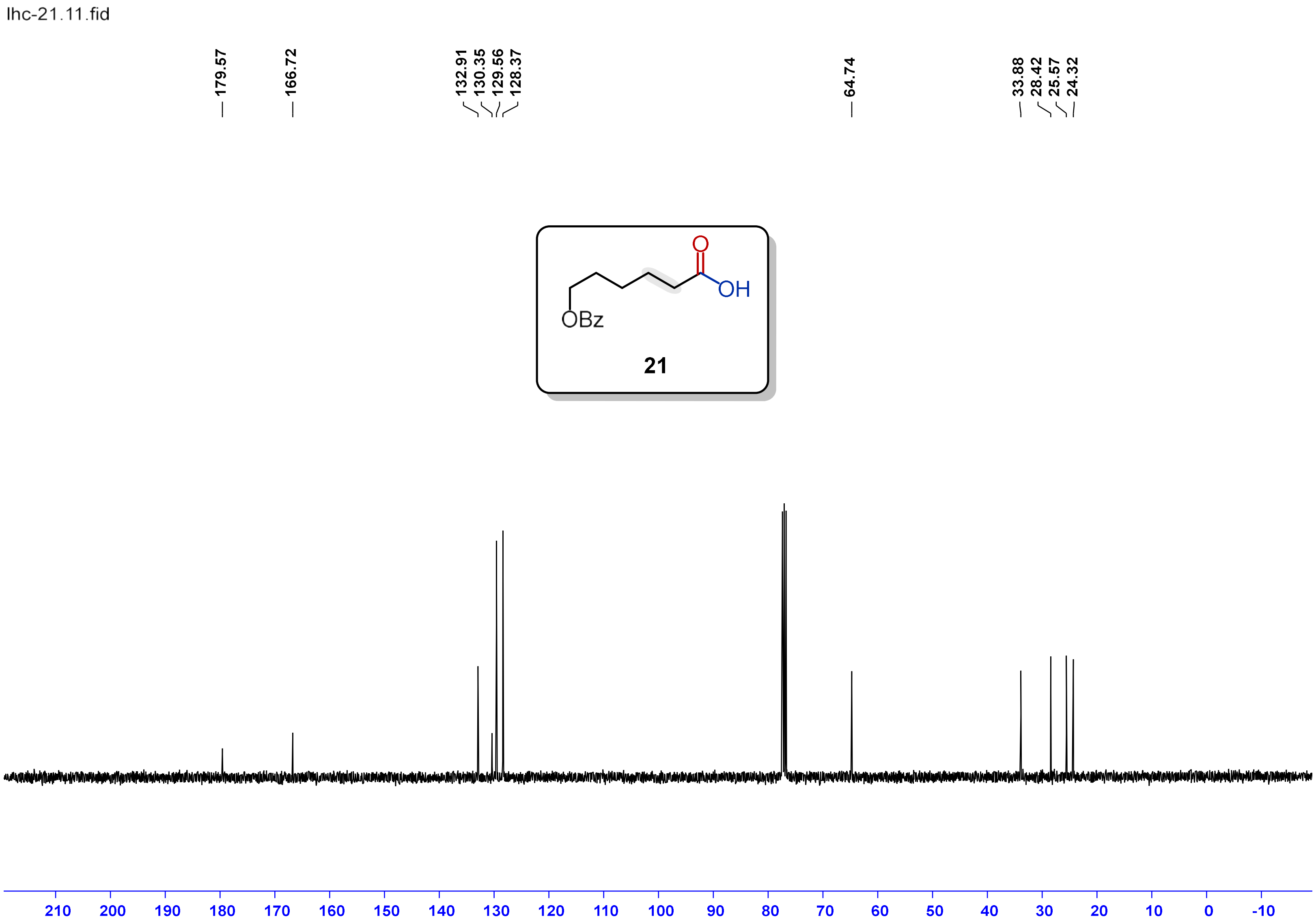

^{1}H NMR spectra for 22

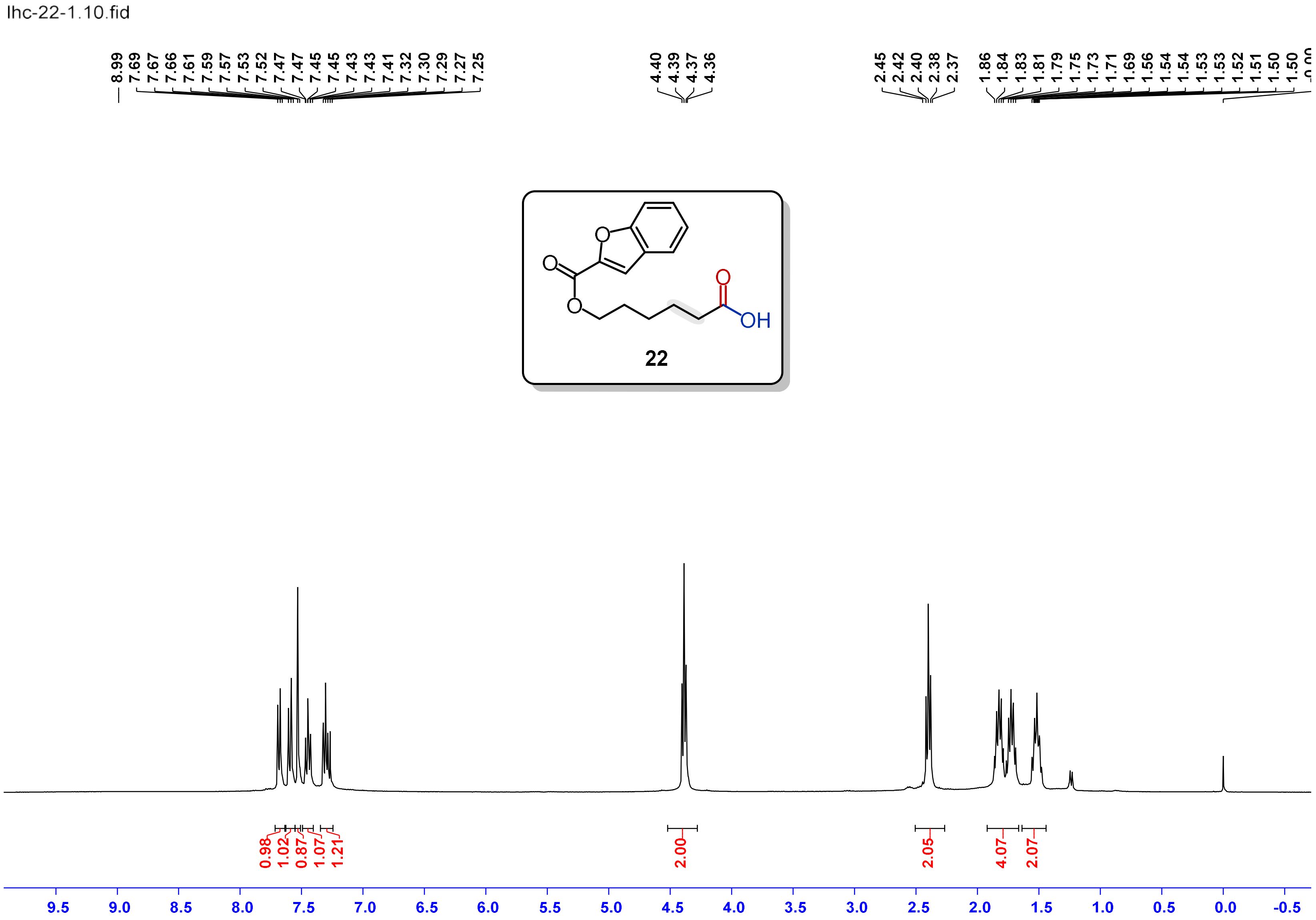

22

^{13}C NMR spectra for 22

lhc-22-1.11.fid

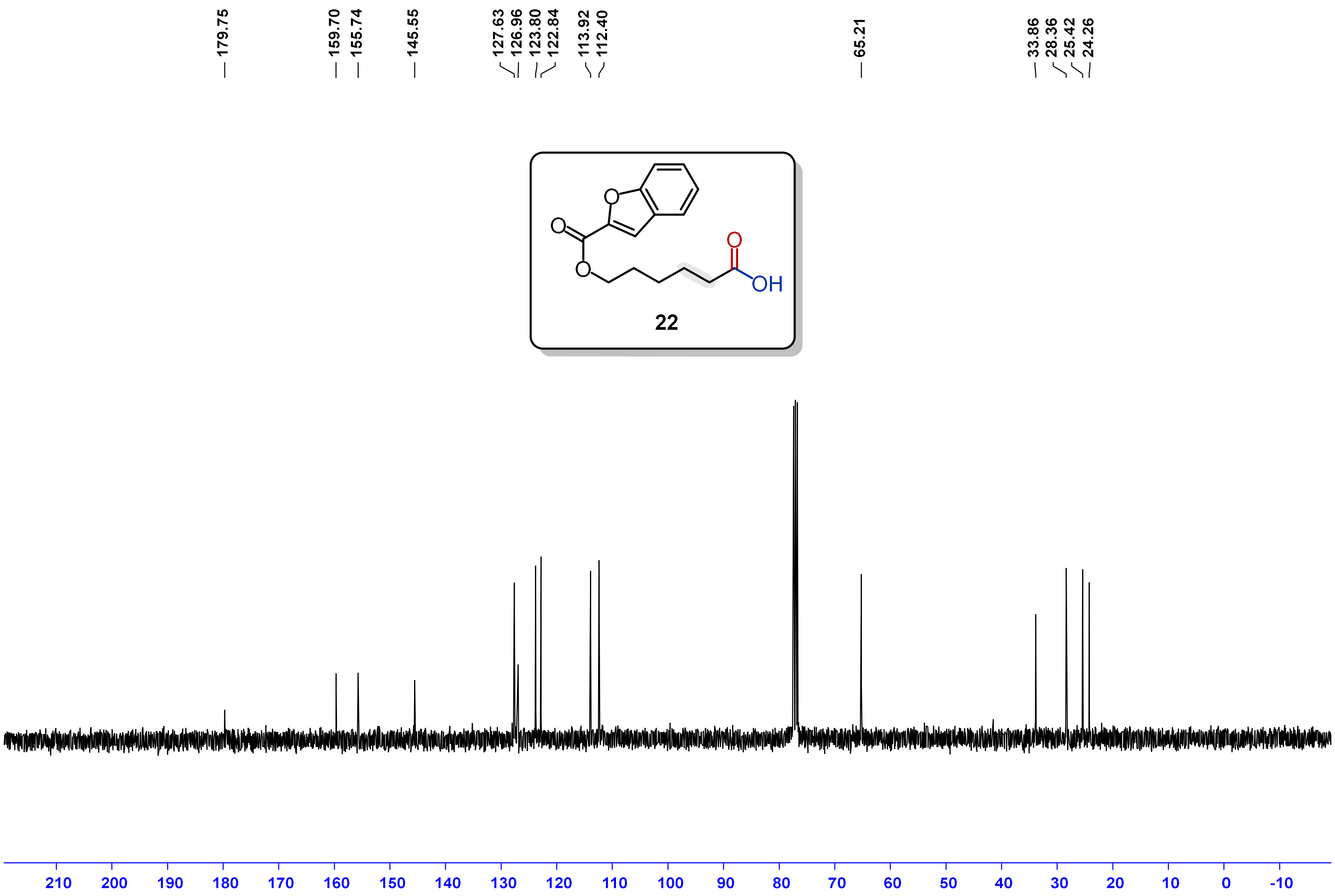

^{1}H NMR spectra for 23

lhc-x24z27-06.3.fid — 1H NMR (400 MHz, CDCl3)

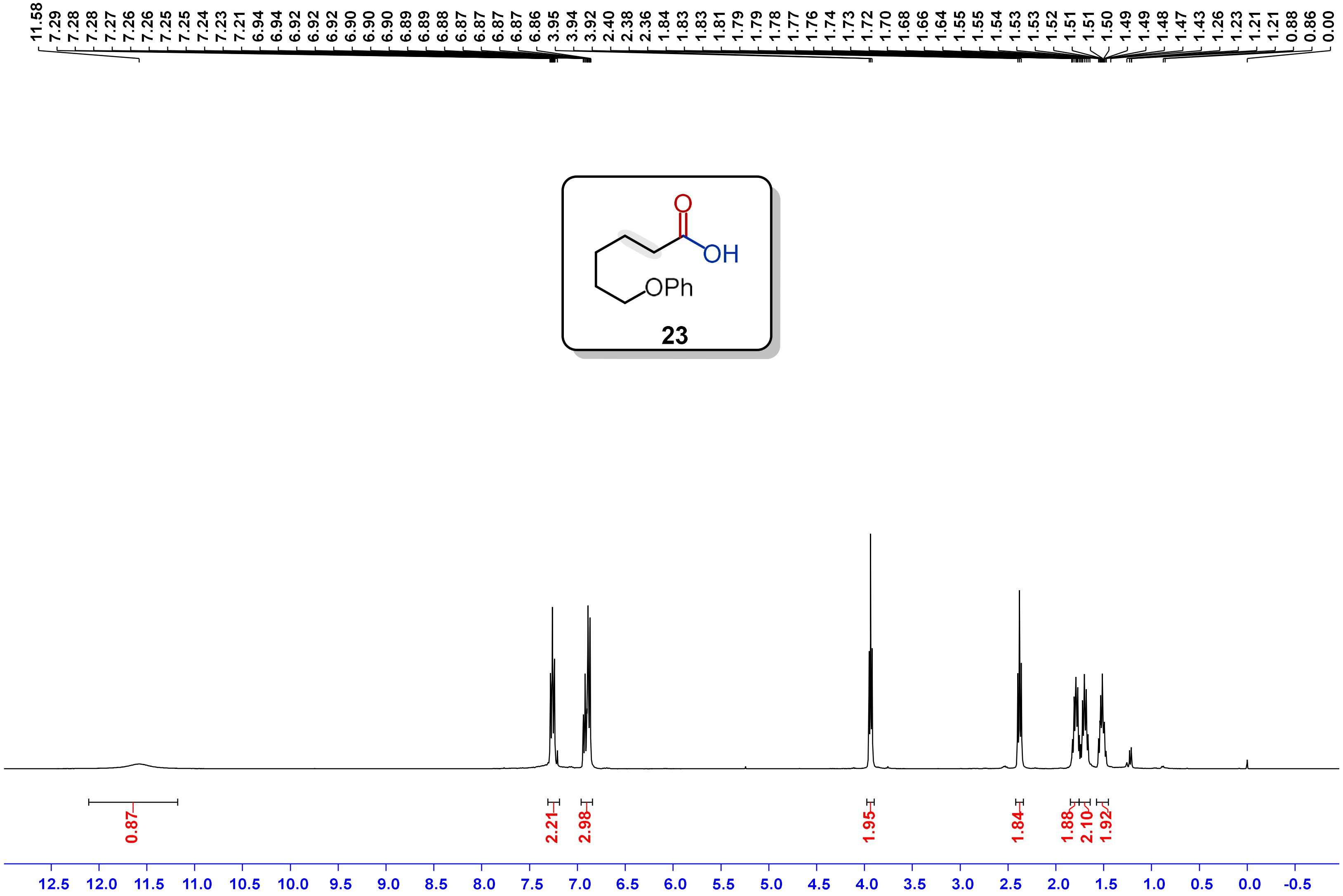

23

^{13}C NMR spectra for 23

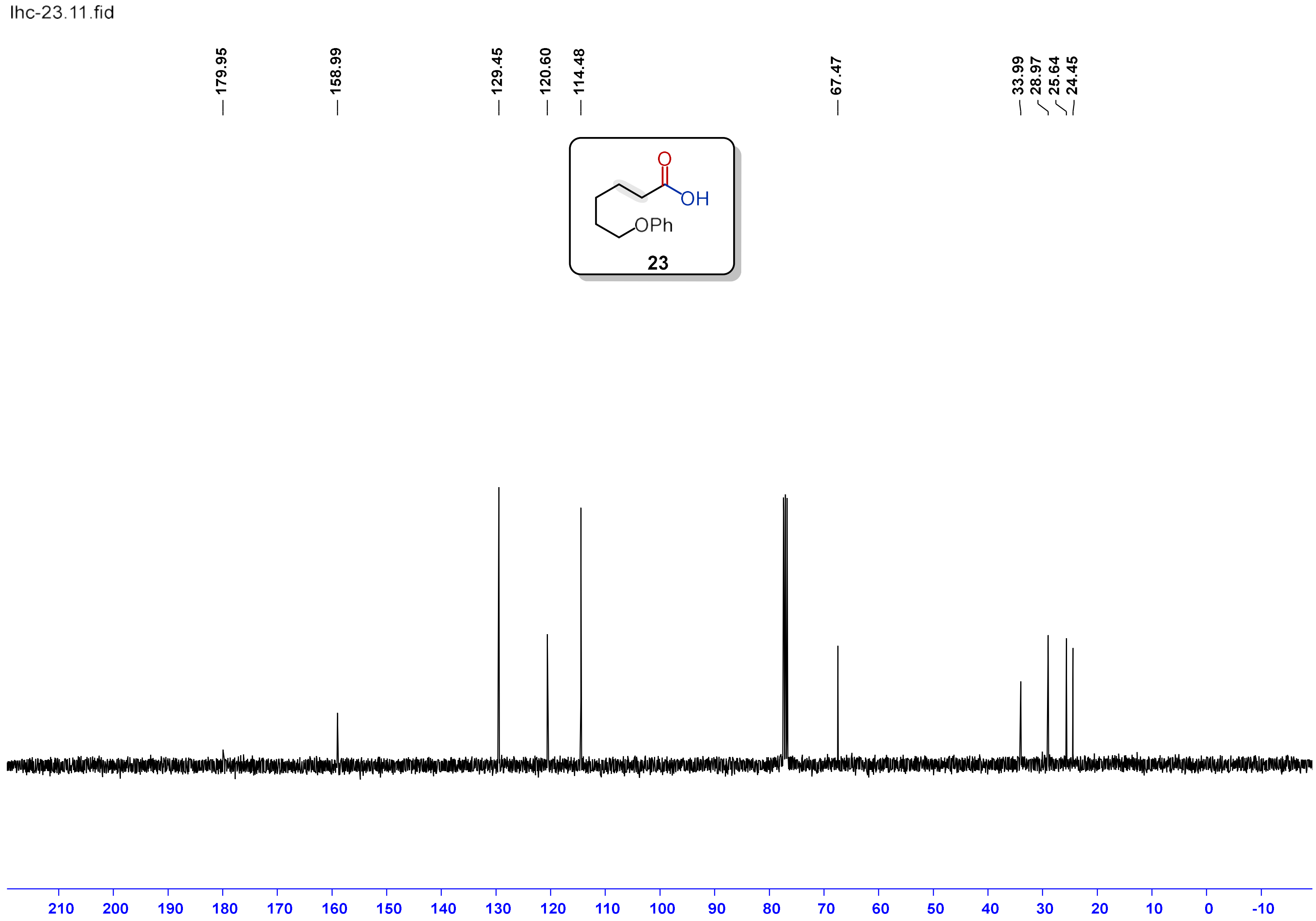

^{1}H NMR spectra for 24

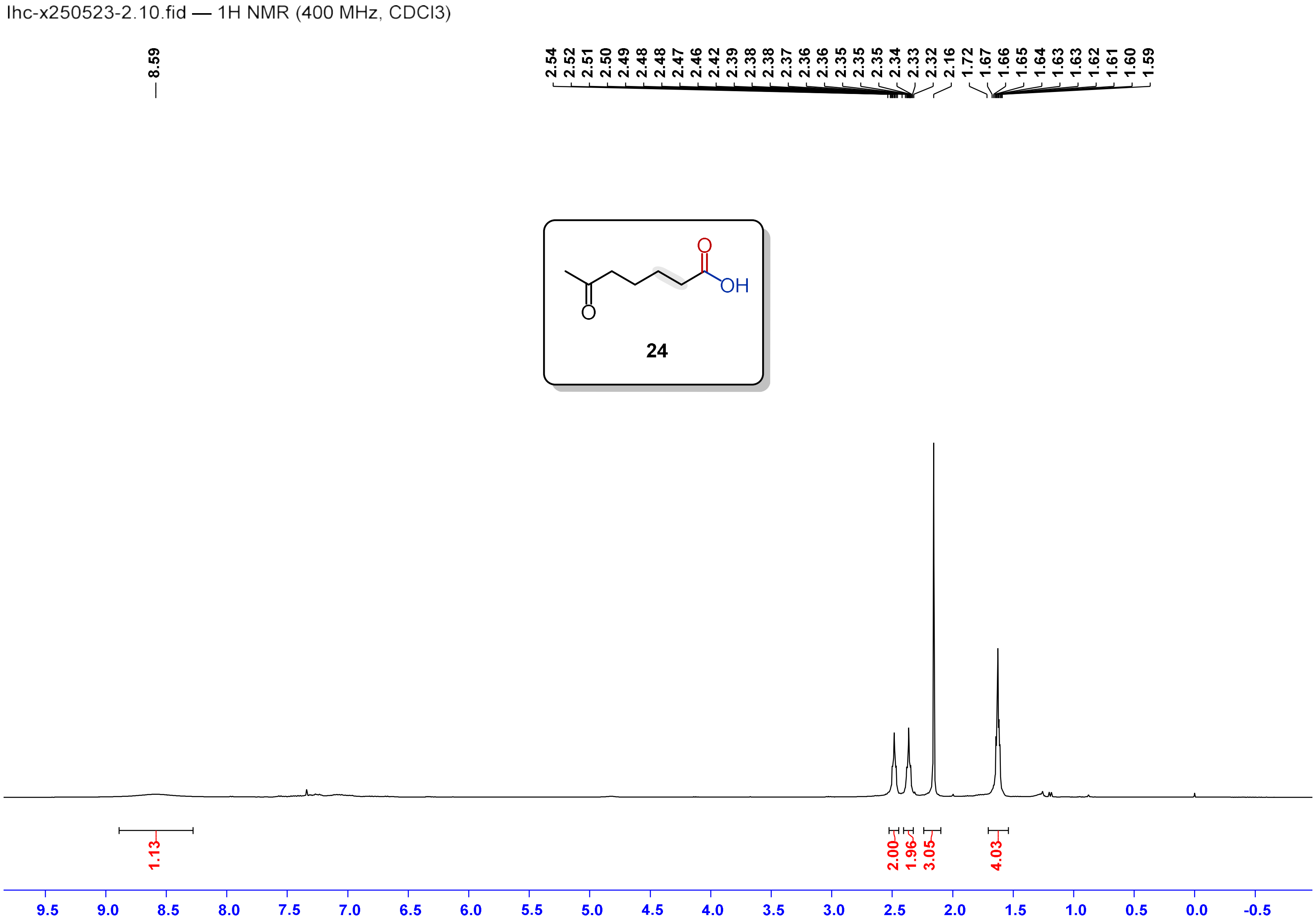

^{13}C NMR spectra for 24

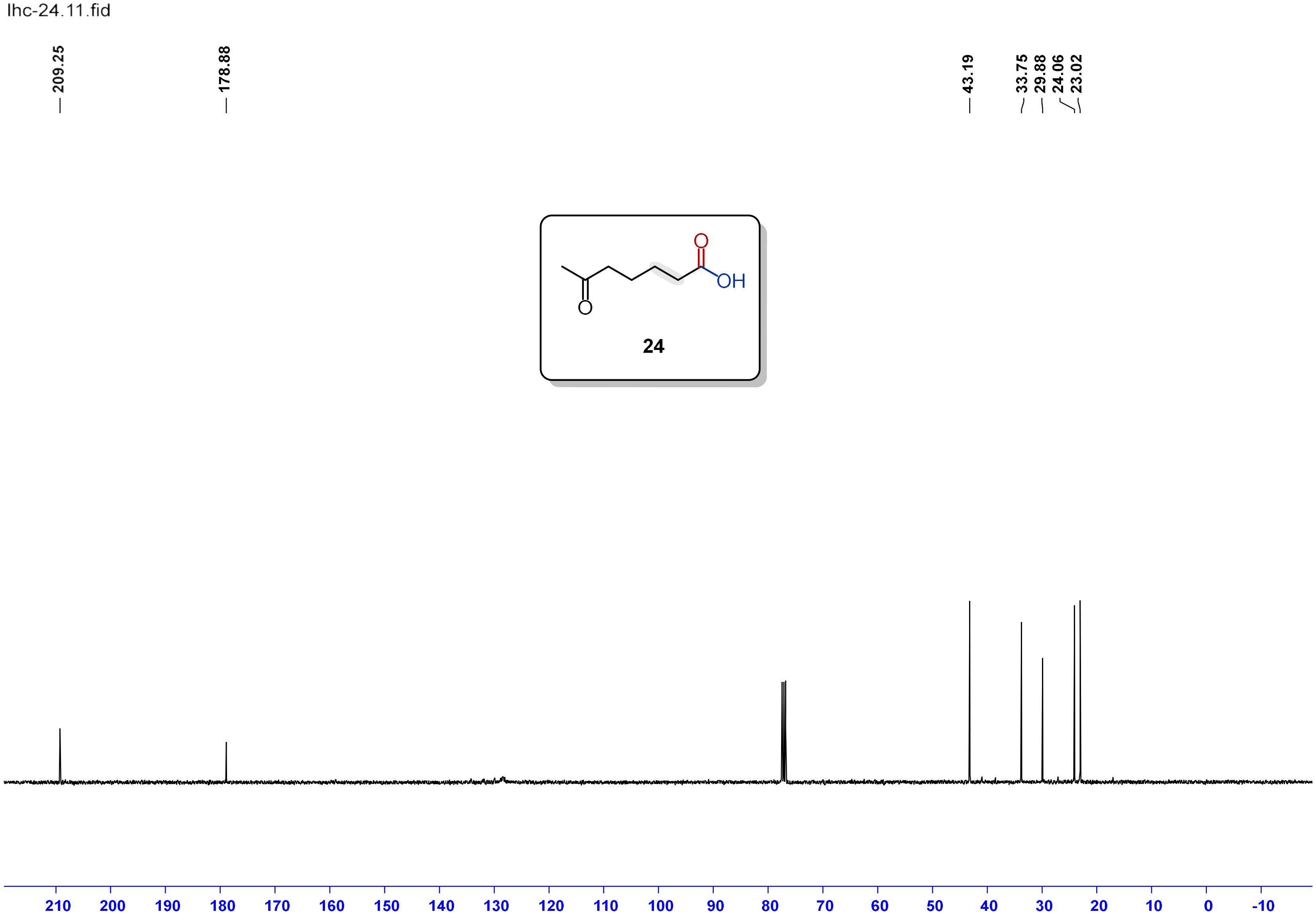

^{1}H NMR spectra for 25

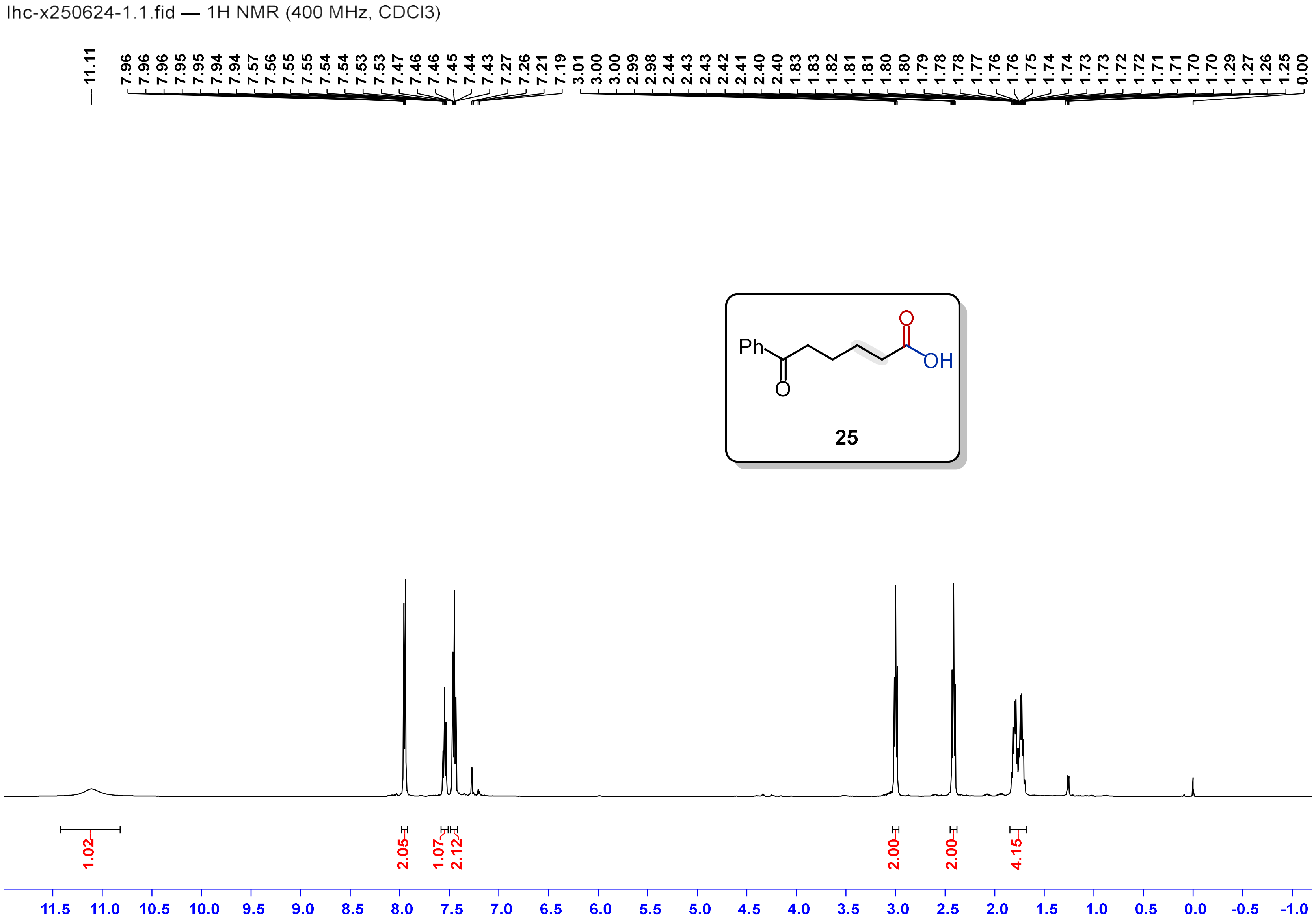

^{13}C NMR spectra for 25

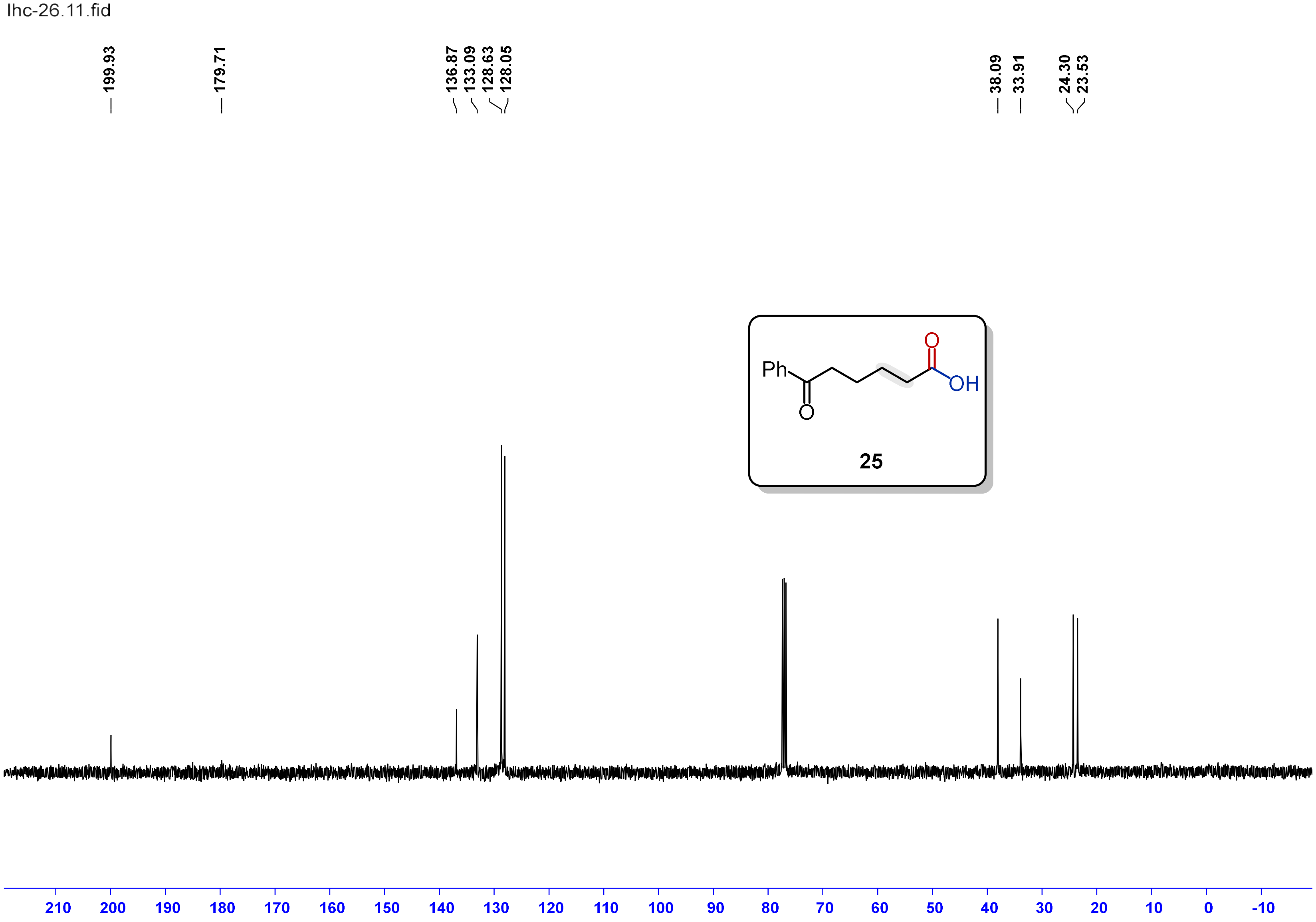

^{1}H NMR spectra for 26

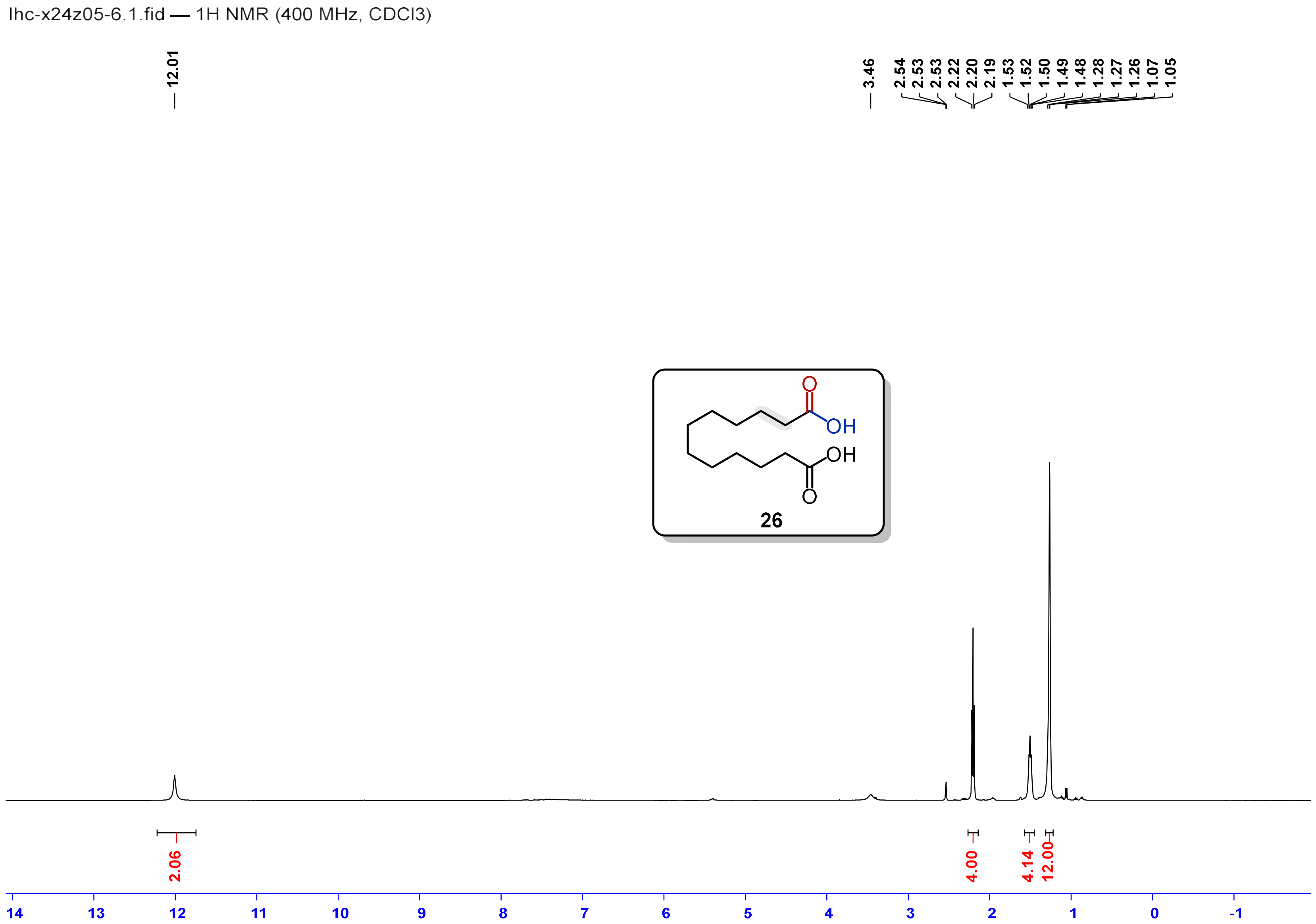

^{13}C NMR spectra for 26

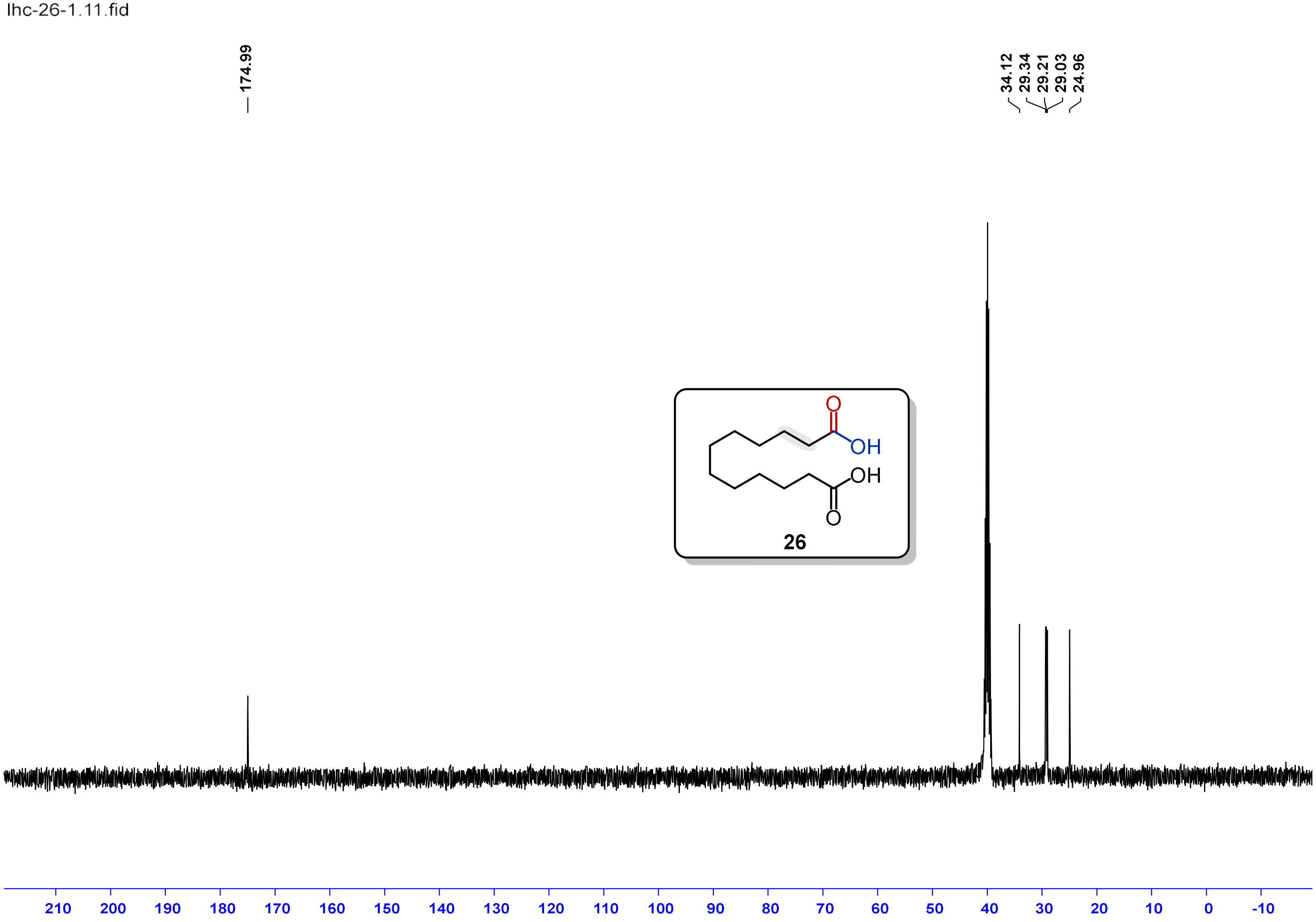

^{1}H NMR spectra for 27

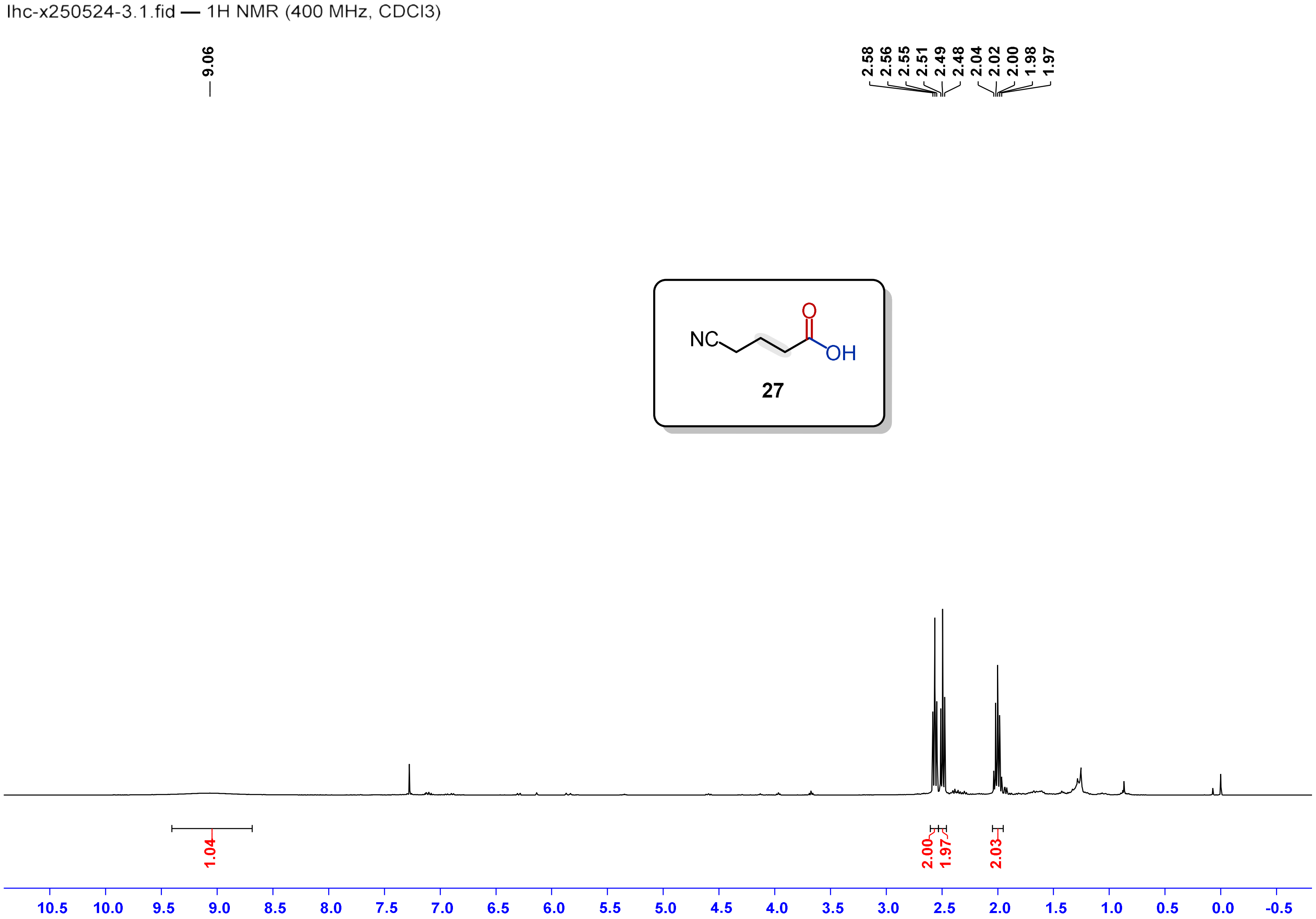

^{13}C NMR spectra for 27

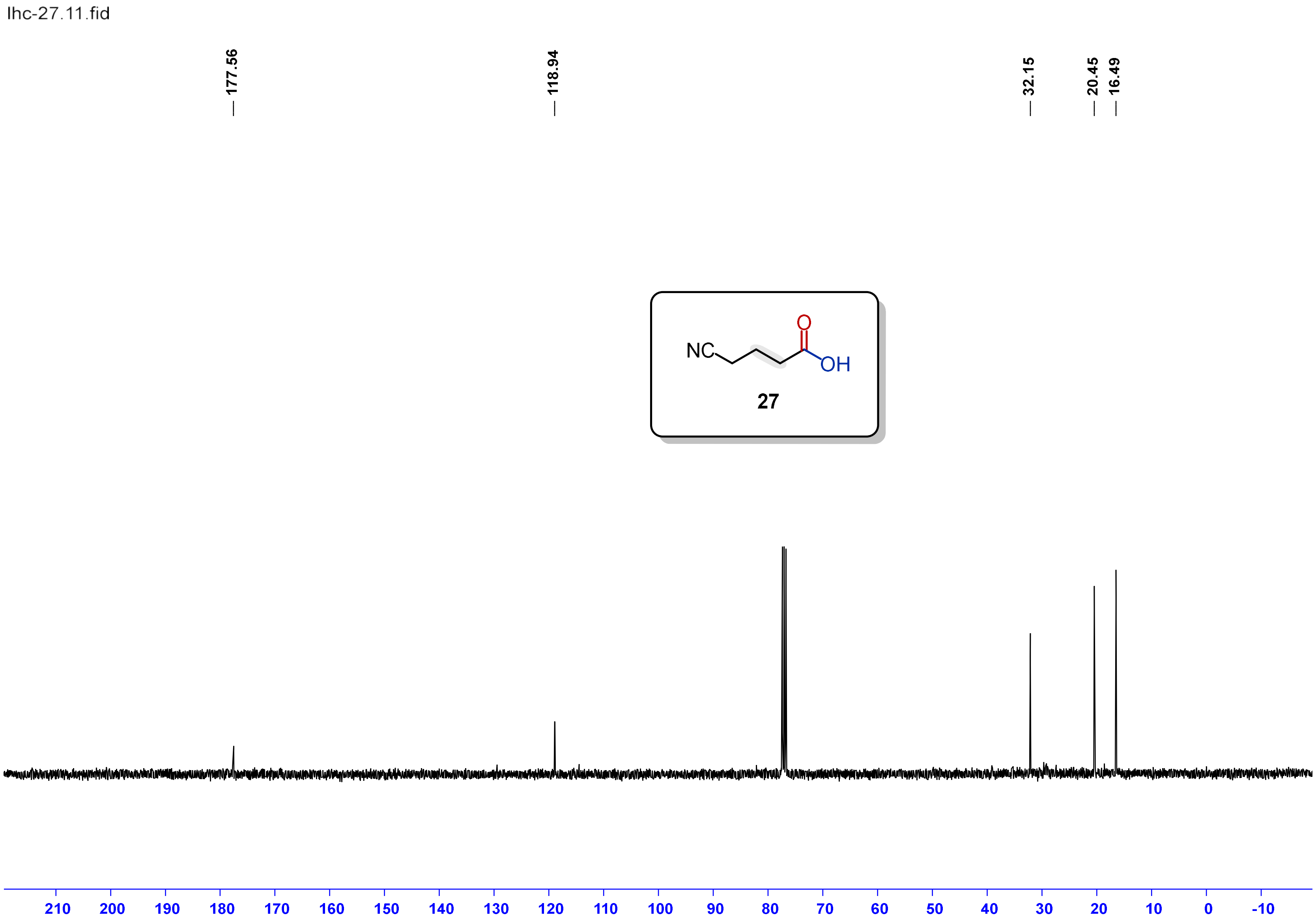

^{1}H NMR spectra for 28

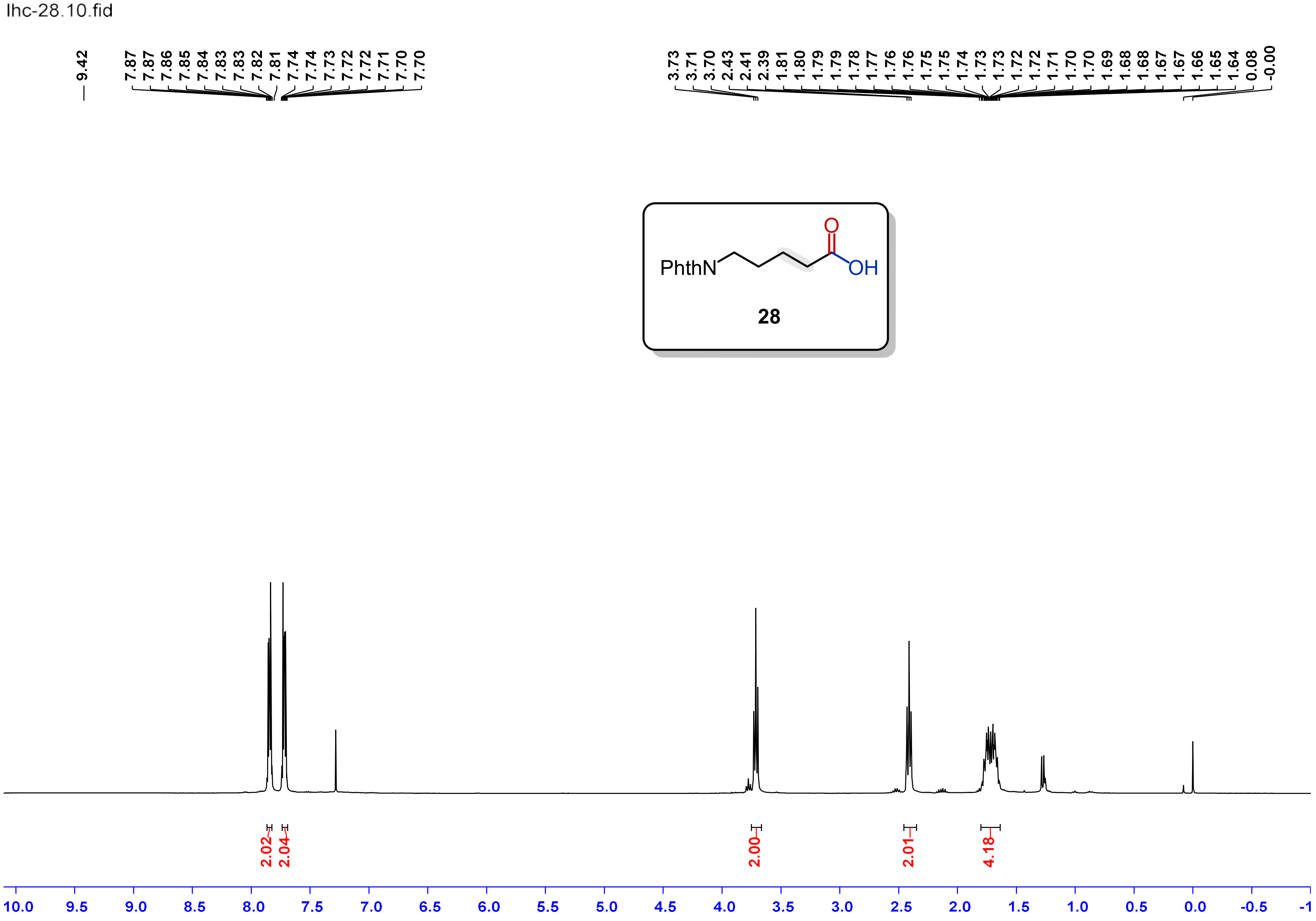

^{13}C NMR spectra for 28

lhc-28.11.fid

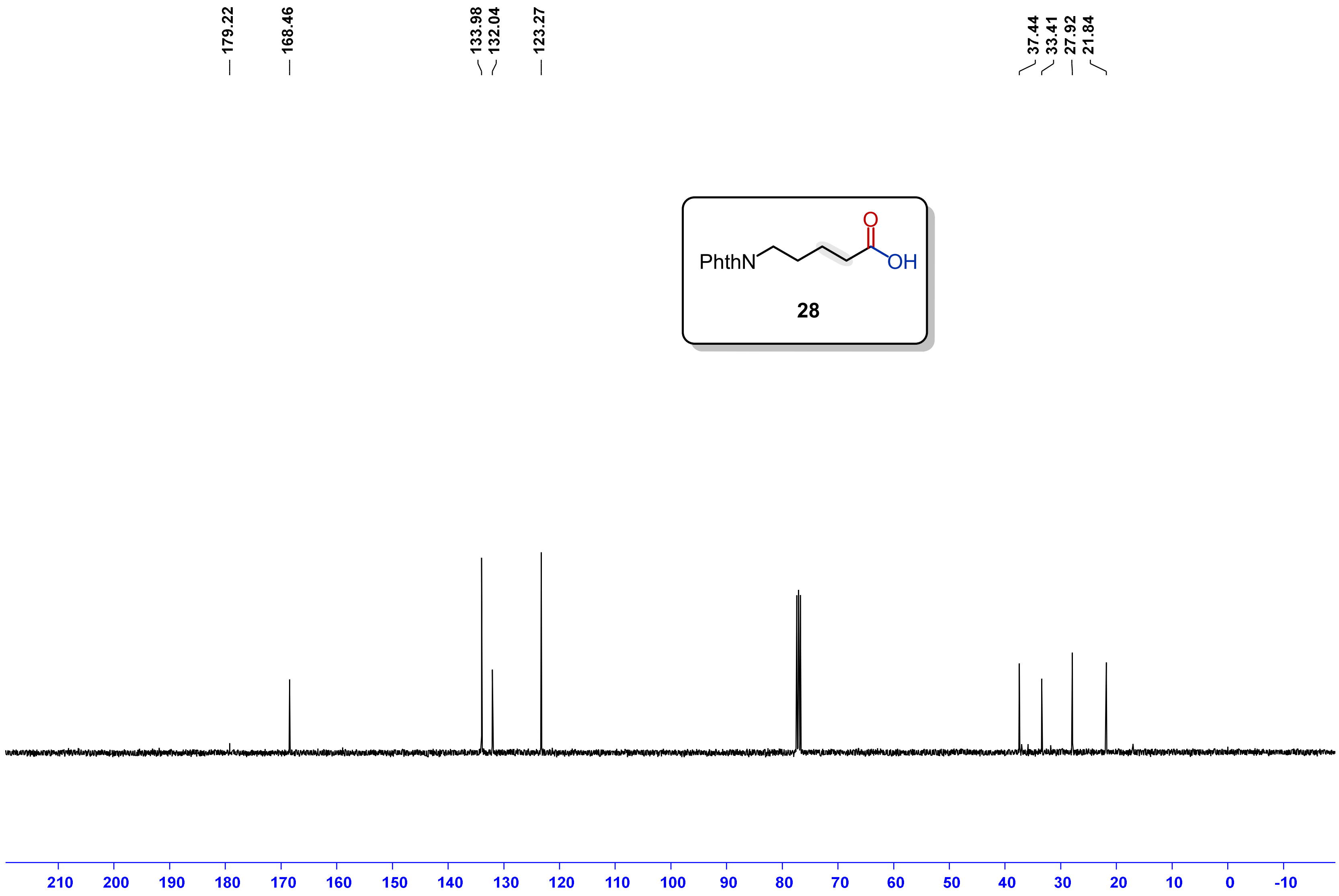

^{1}H NMR spectra for 29

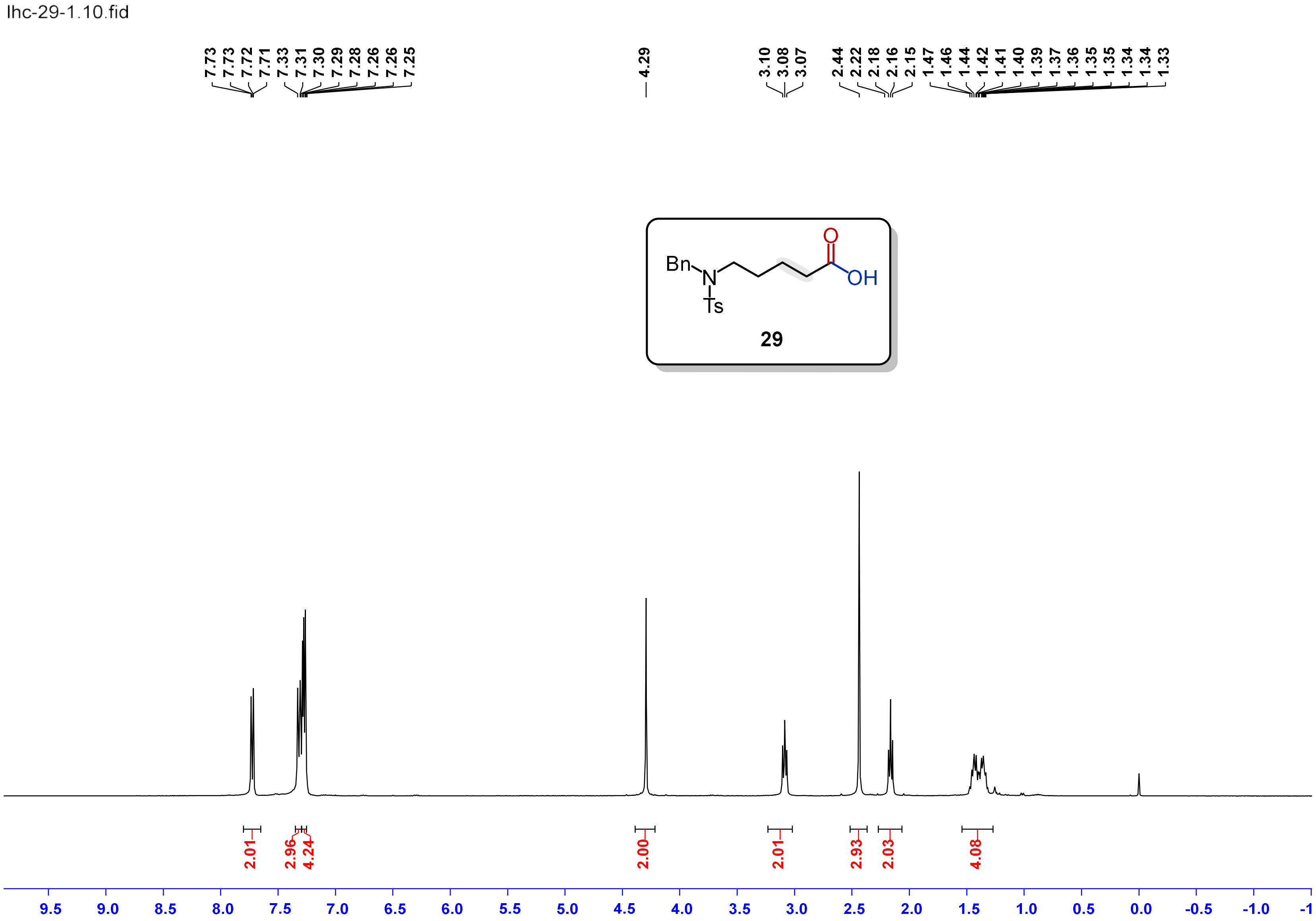

^{13}C NMR spectra for 29

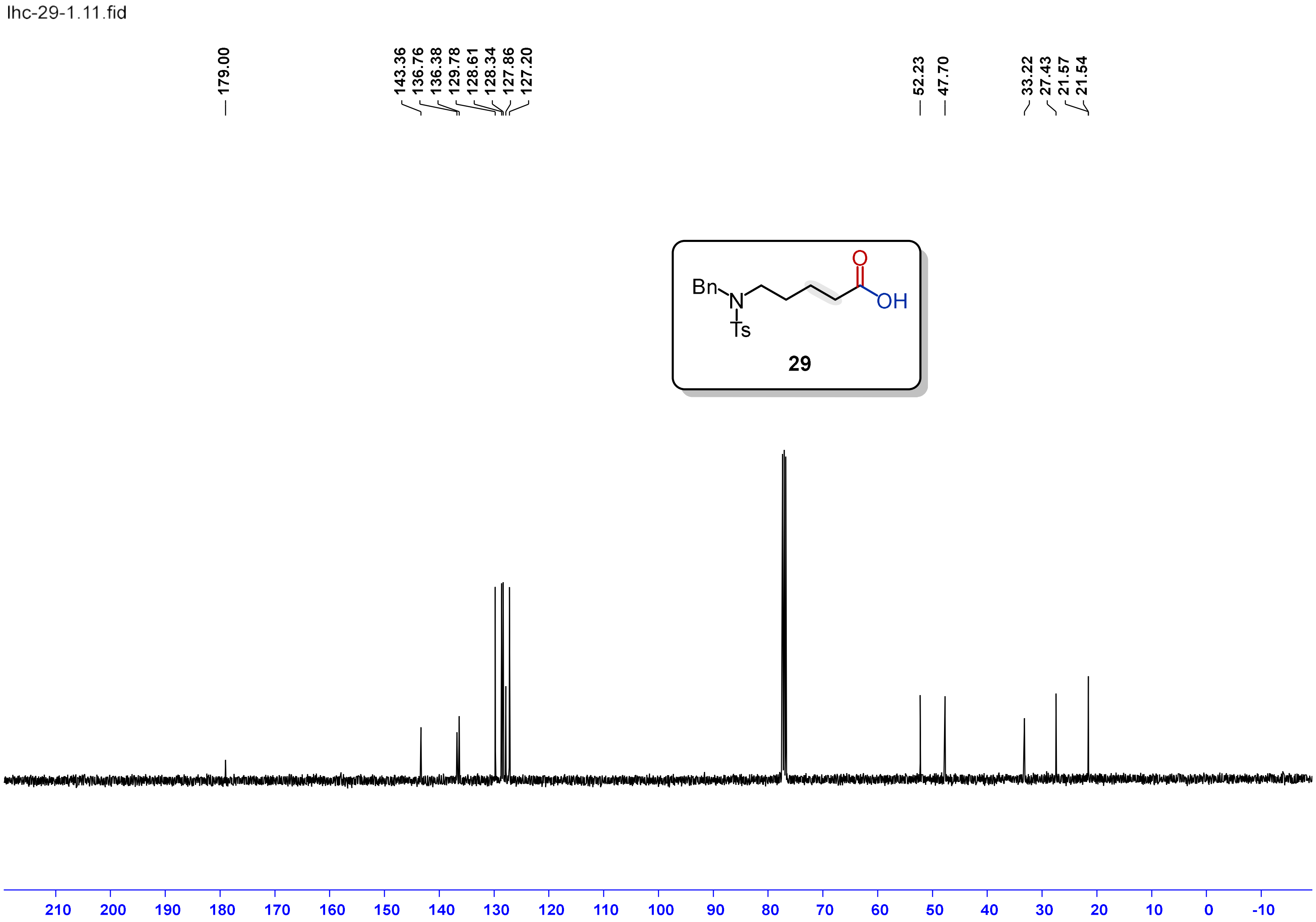

^{1}H NMR spectra for 30

lhc-30-2.1.fid

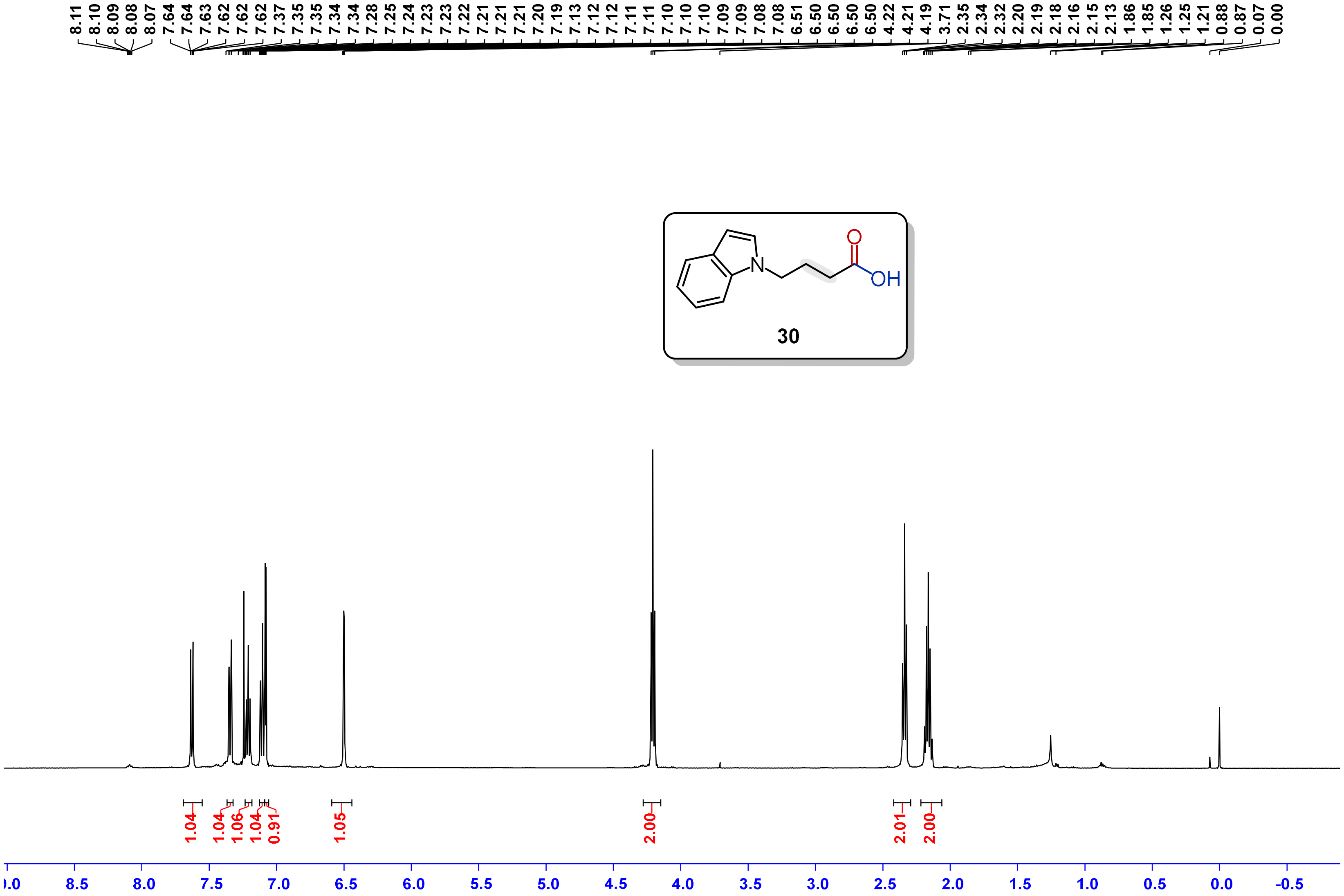

^{13}C NMR spectra for 30

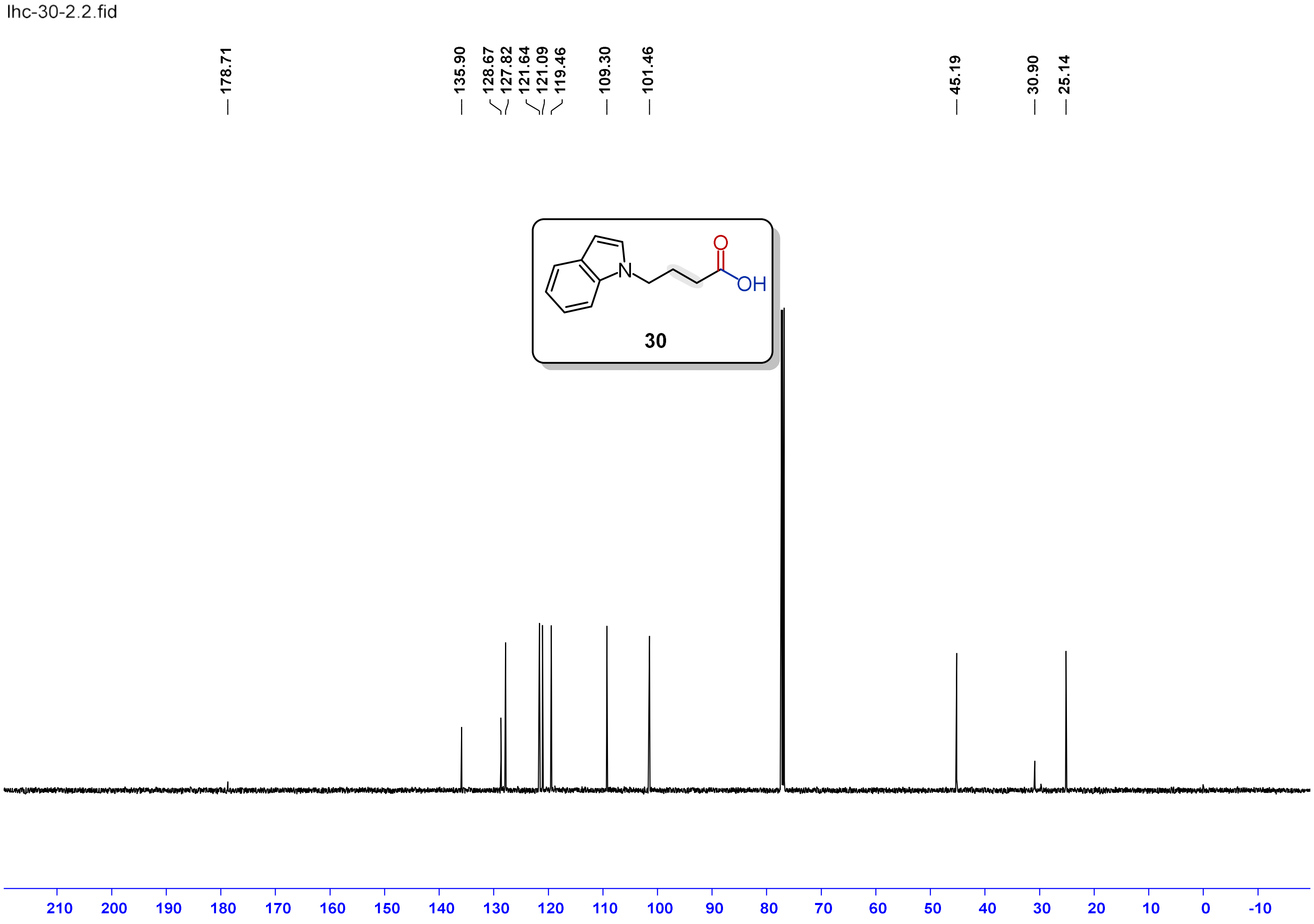

^{1}H NMR spectra for 31

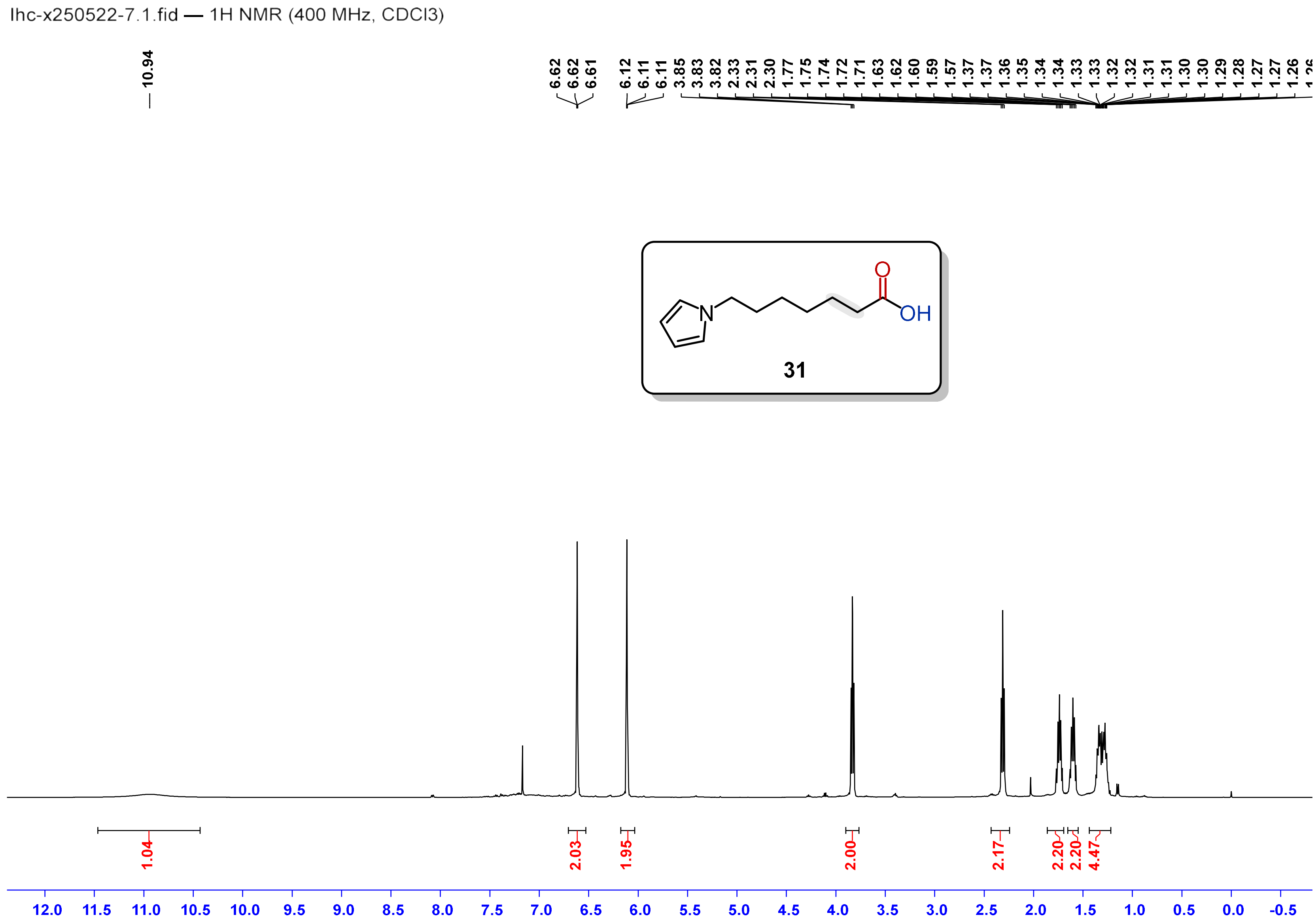

^{13}C NMR spectra for 31

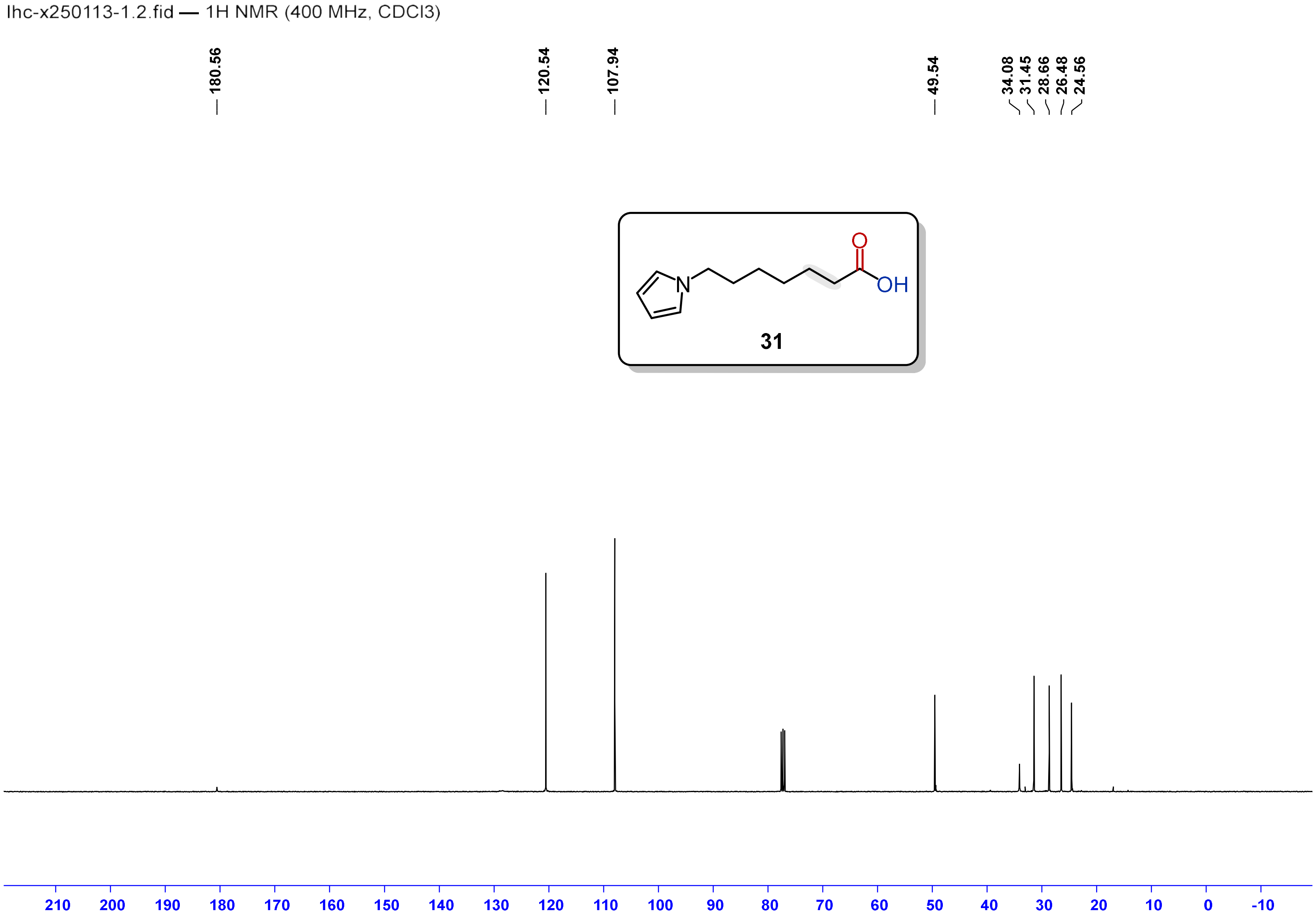

^{1}H NMR spectra for 32

lhc-32.10.fid

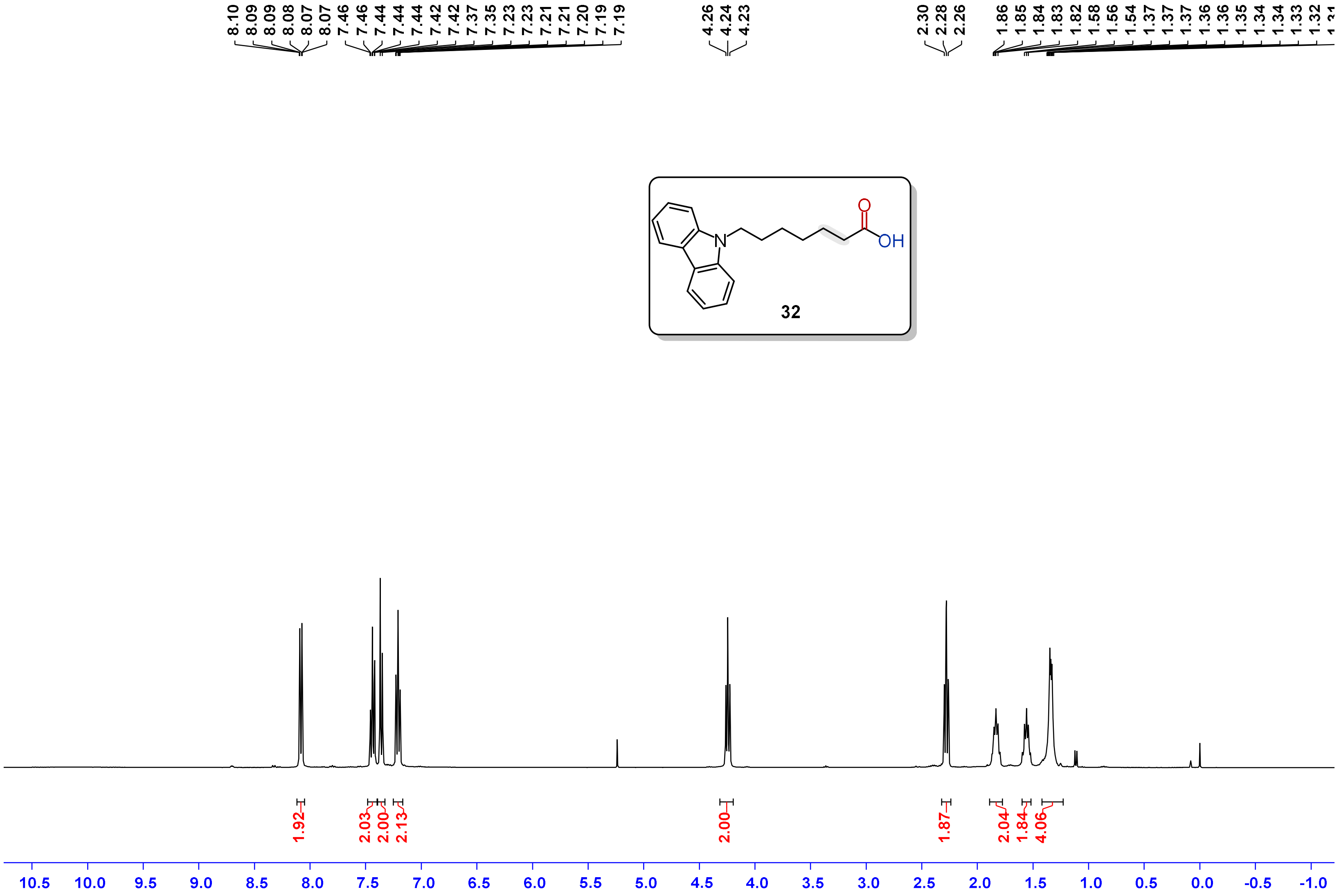

^{13}C NMR spectra for 32

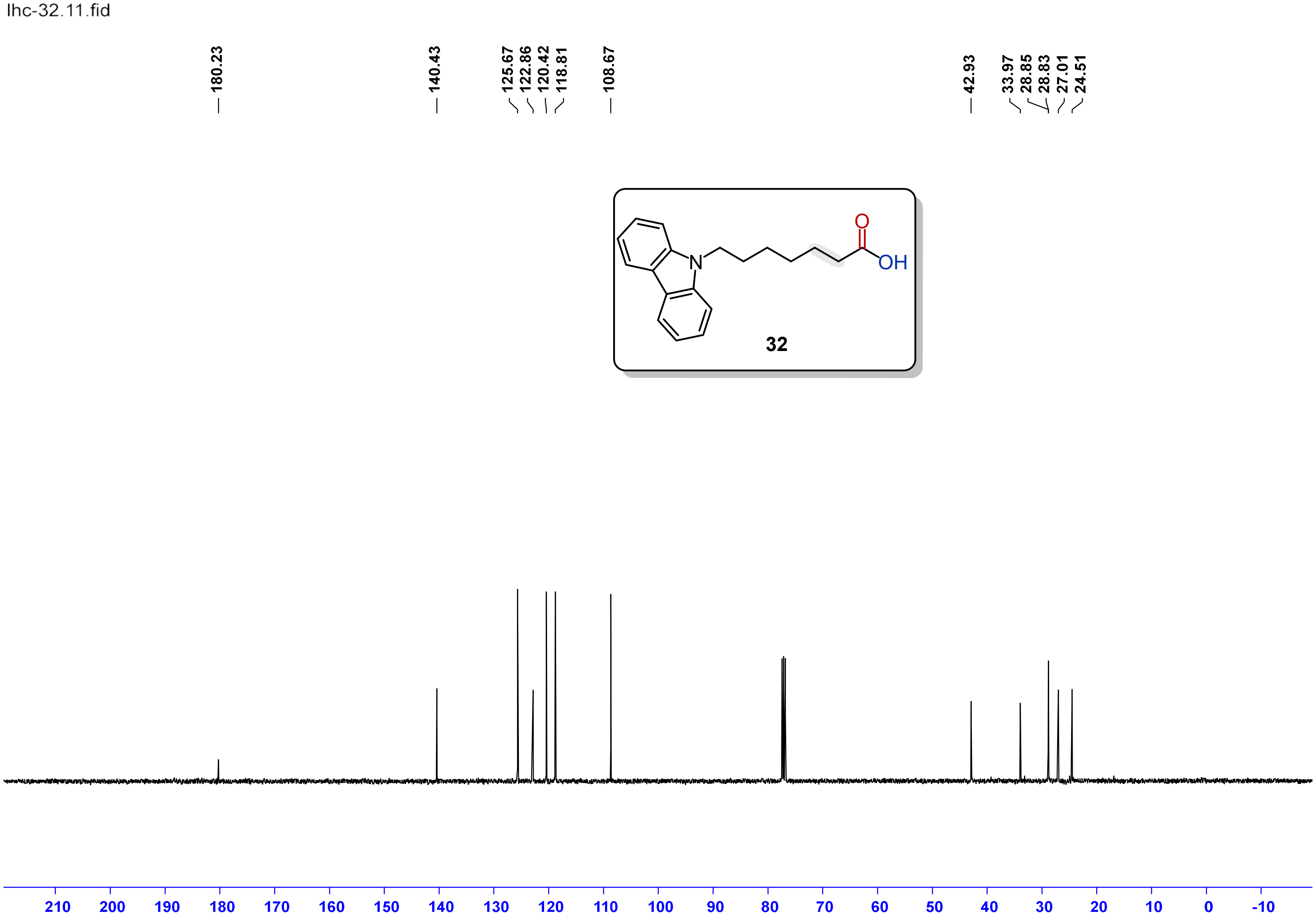

^{1}H NMR spectra for 33

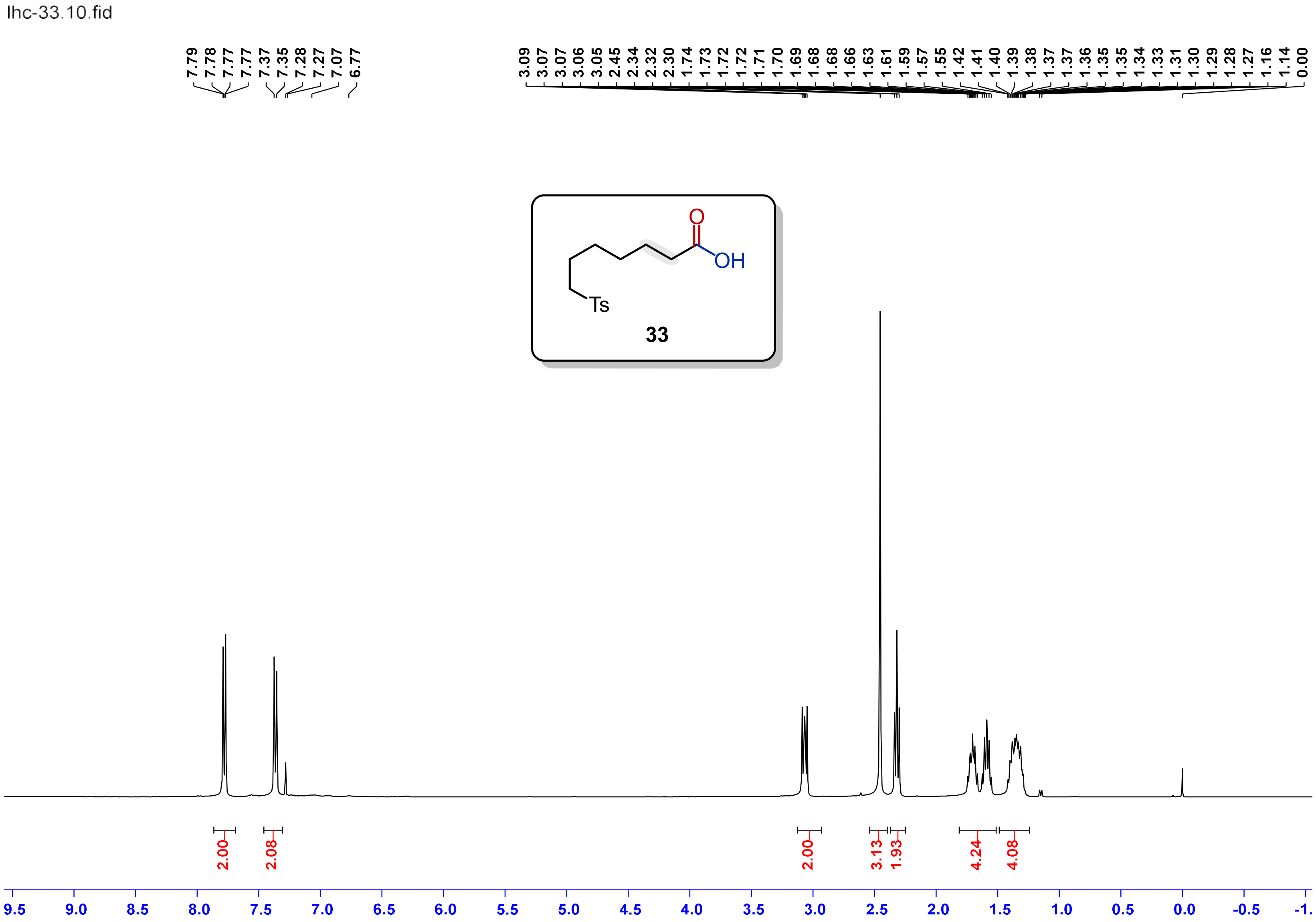

^{13}C NMR spectra for 33

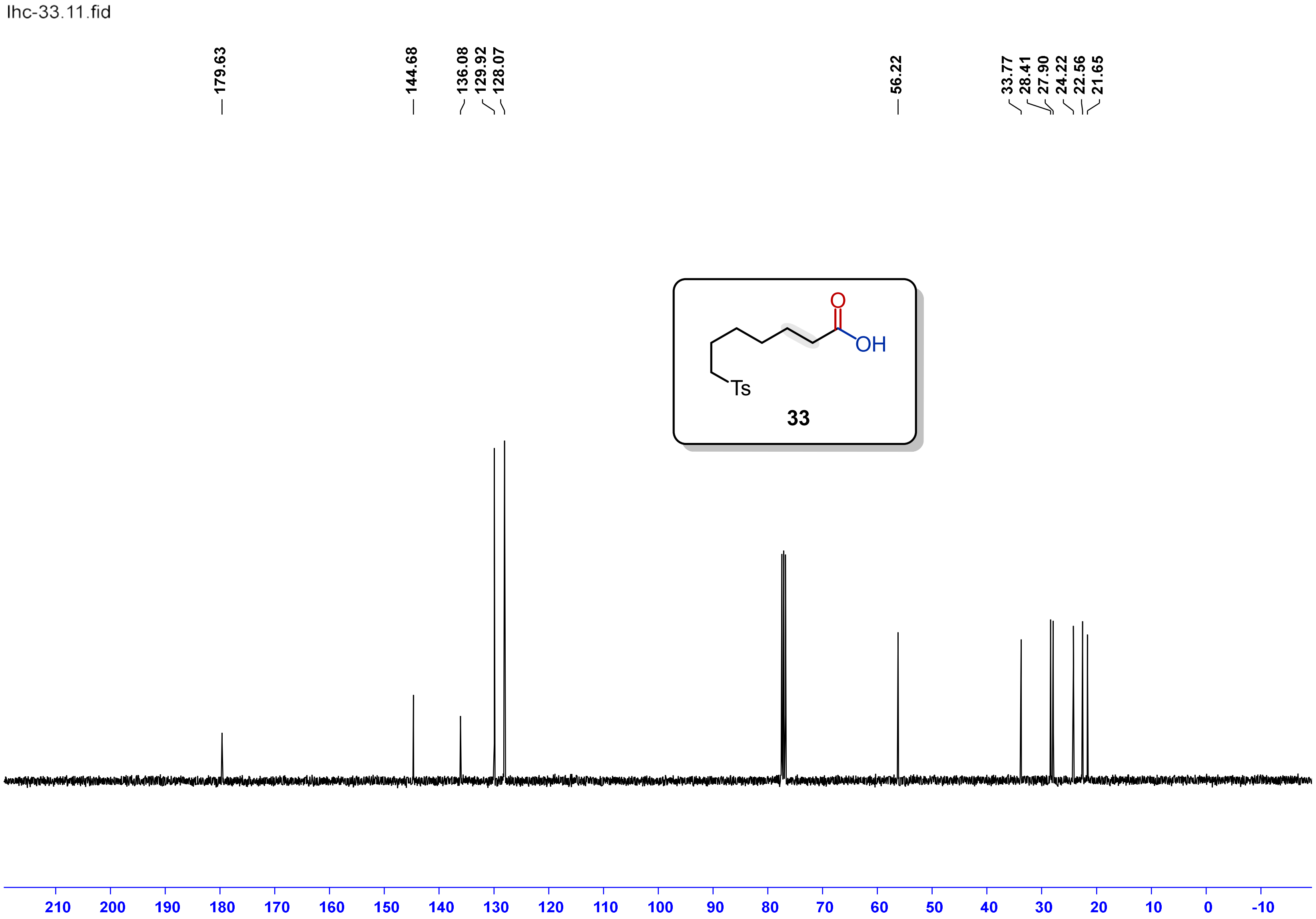

^{1}H NMR spectra for 34

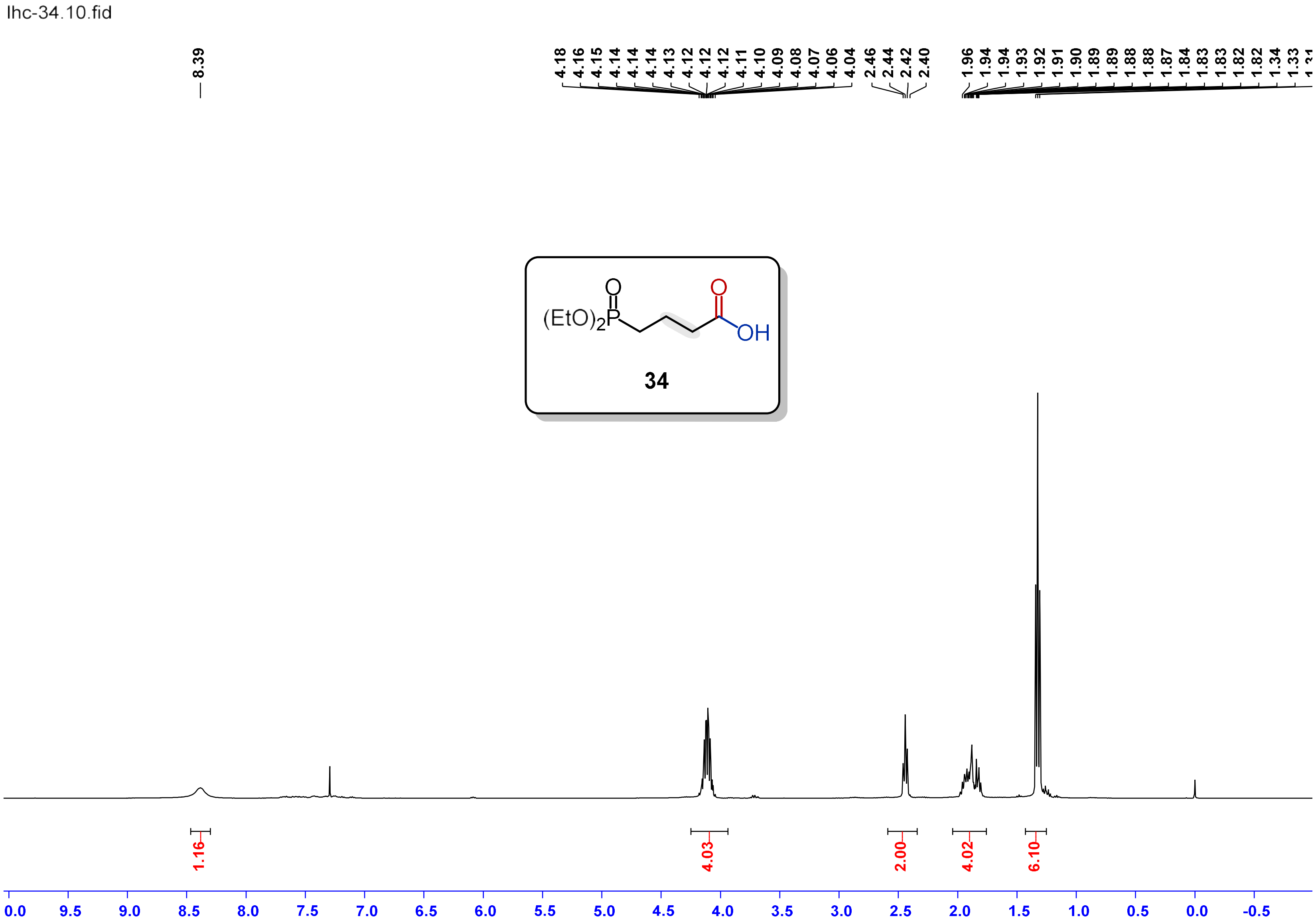

^{13}C NMR spectra for 34

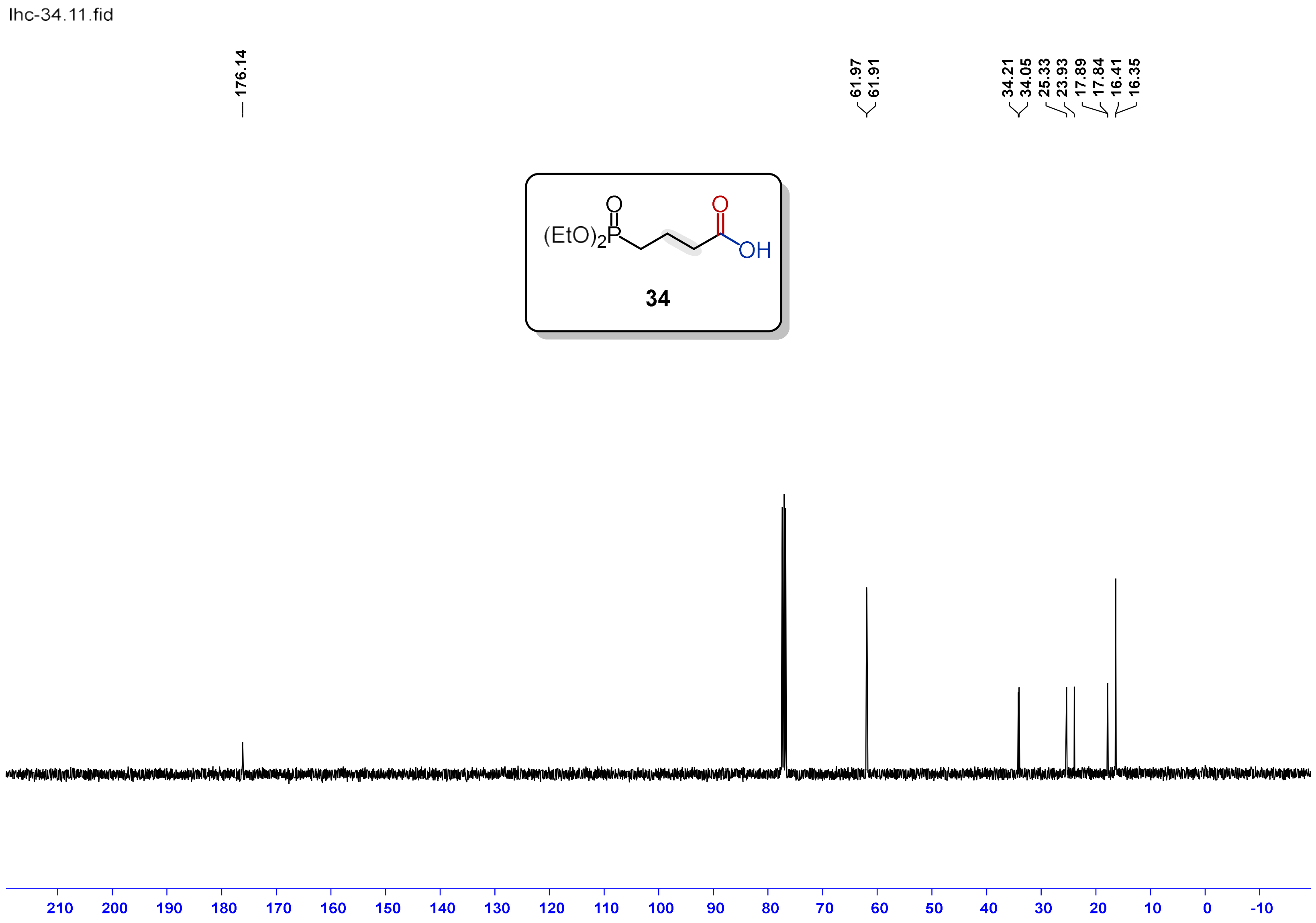

^{13}C NMR spectra for 34

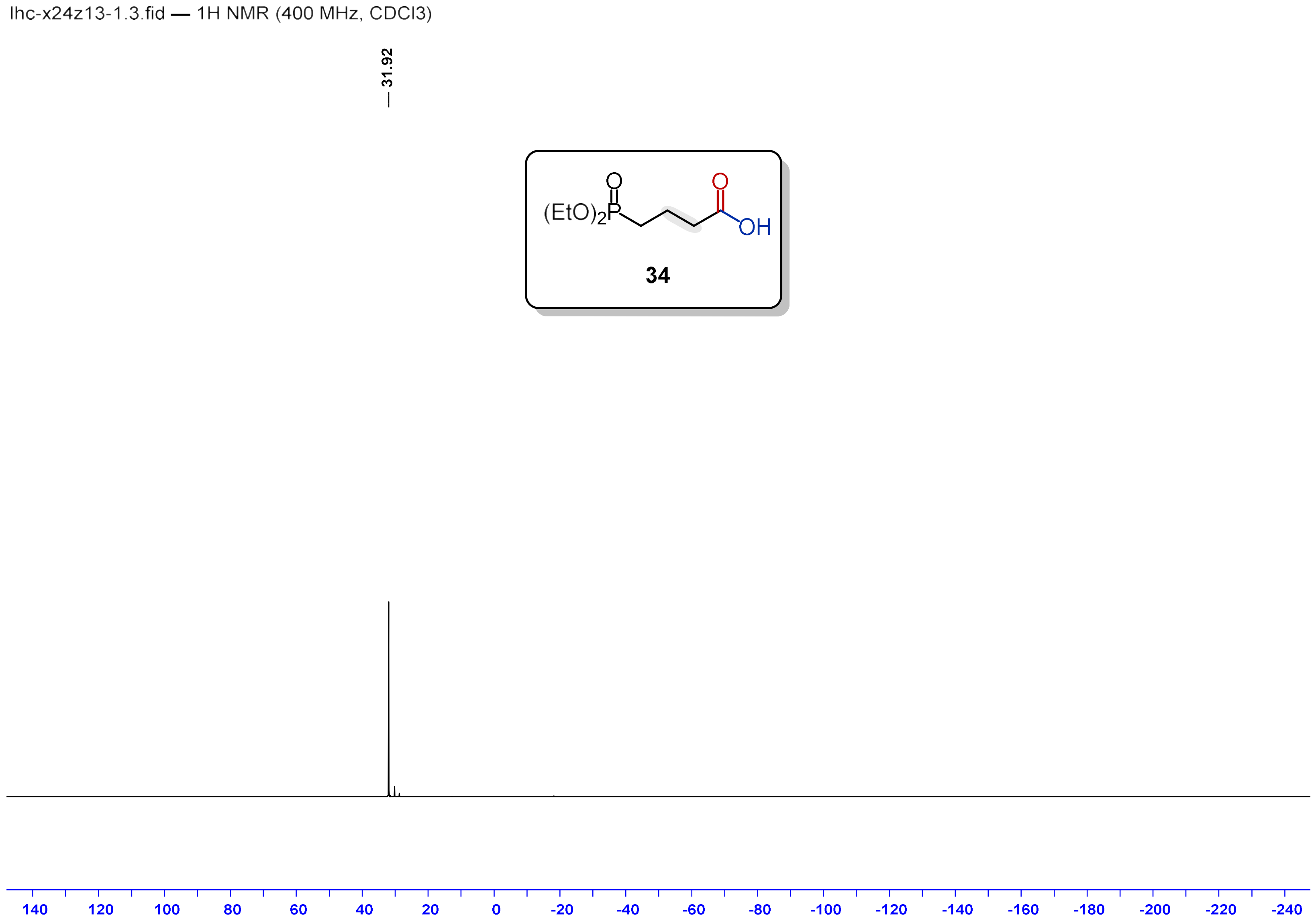

^{1}H NMR spectra for 35

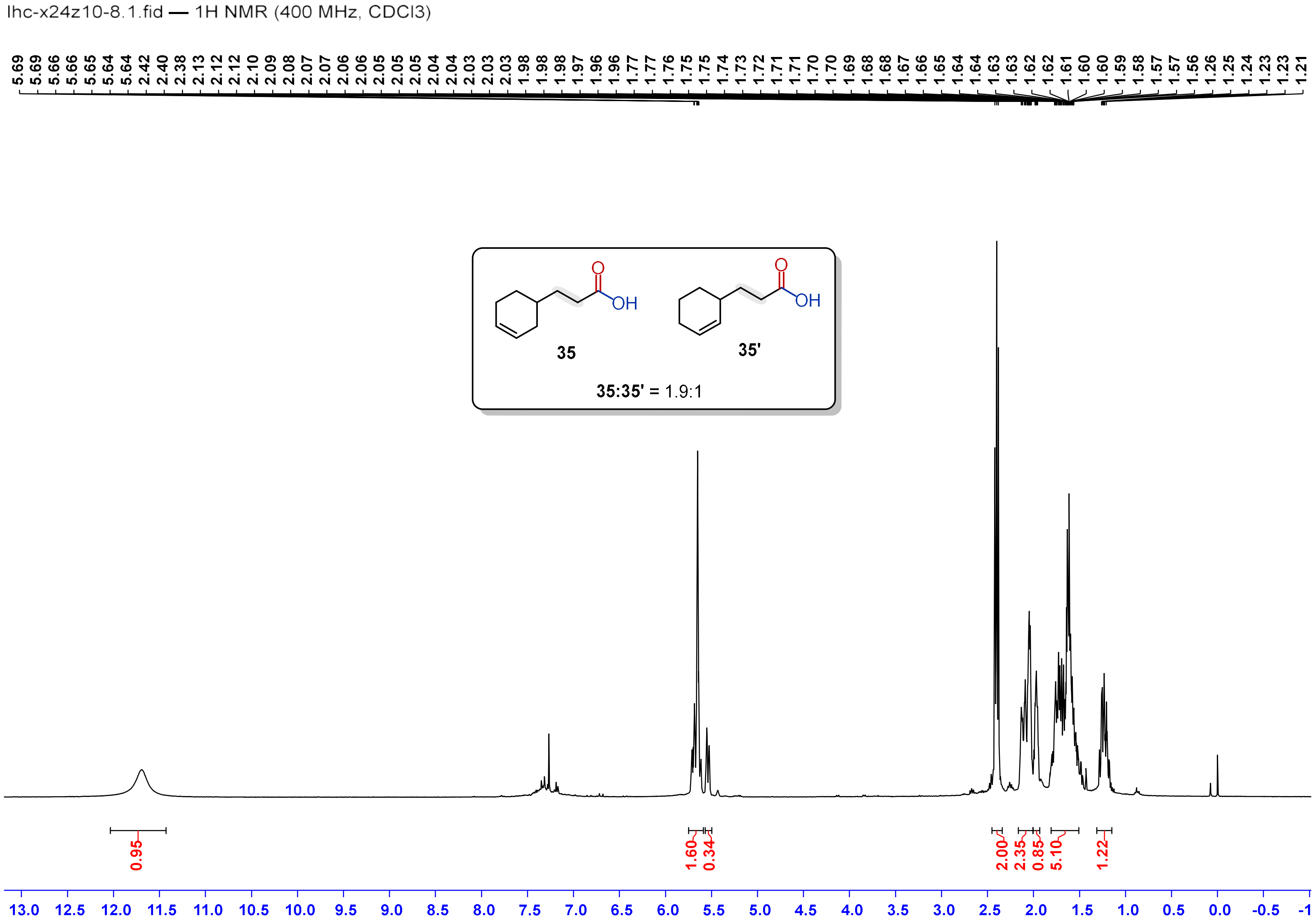

^{13}C NMR spectra for 35

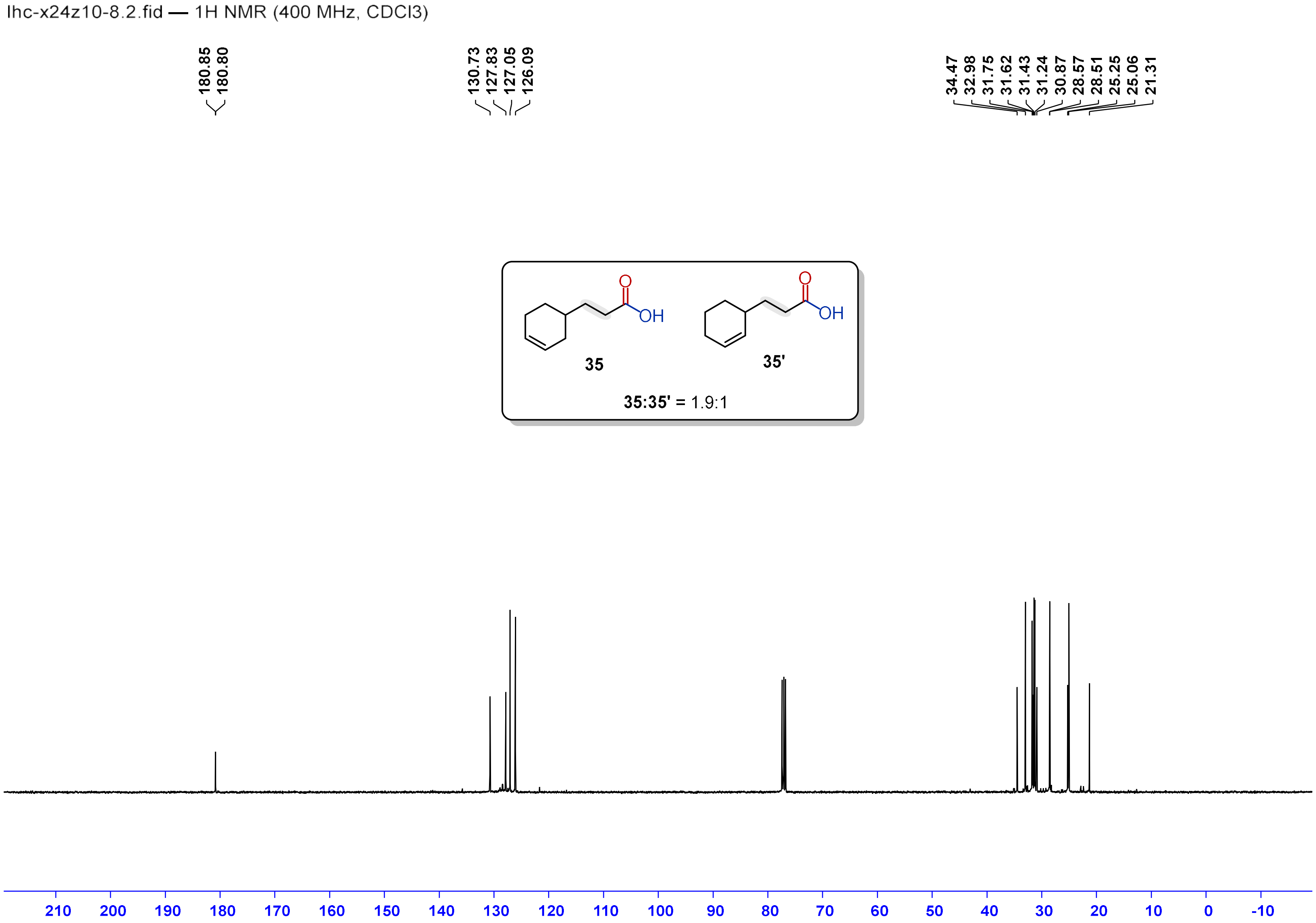

^{1}H NMR spectra for 36

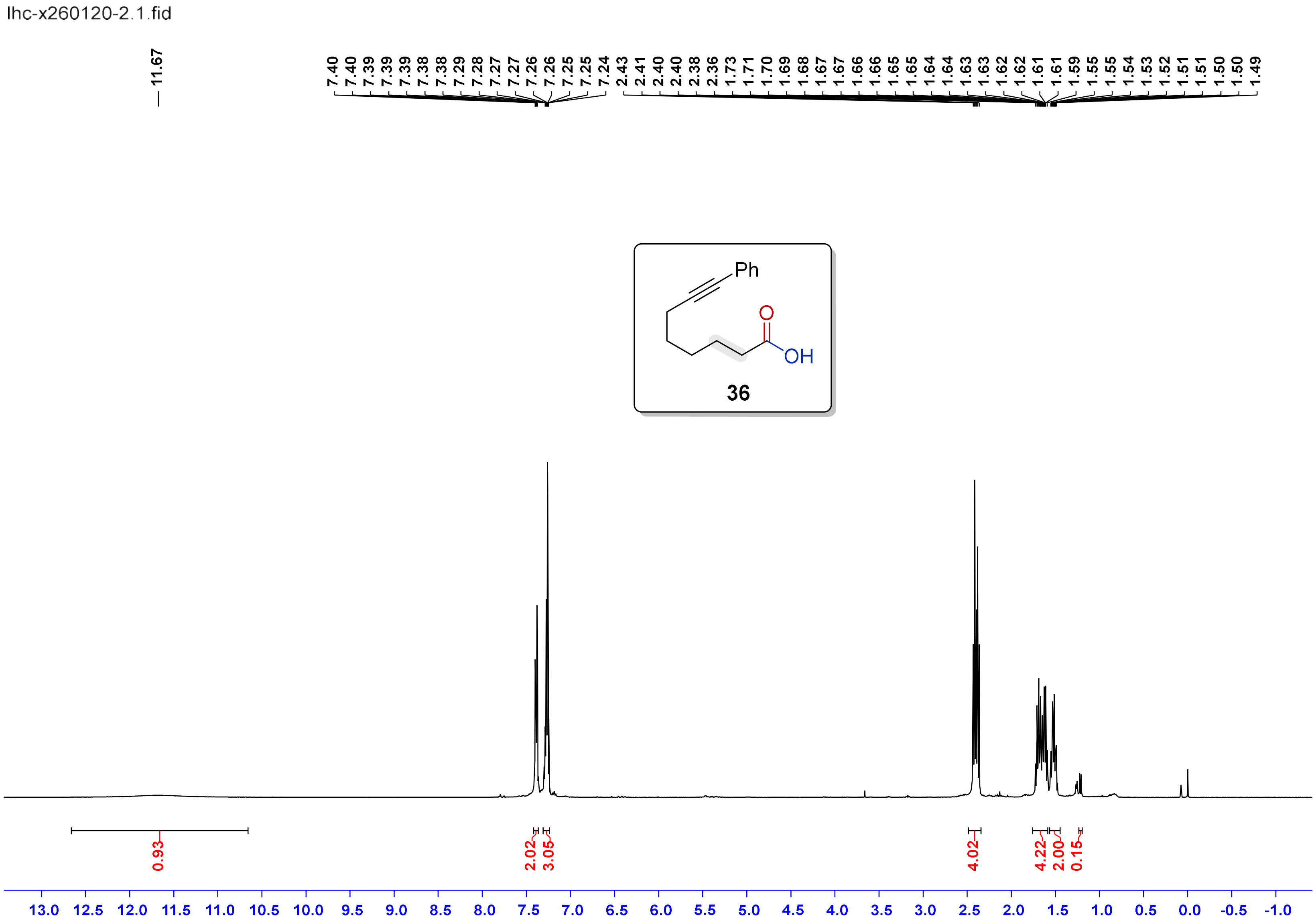

^{13}C NMR spectra for 36

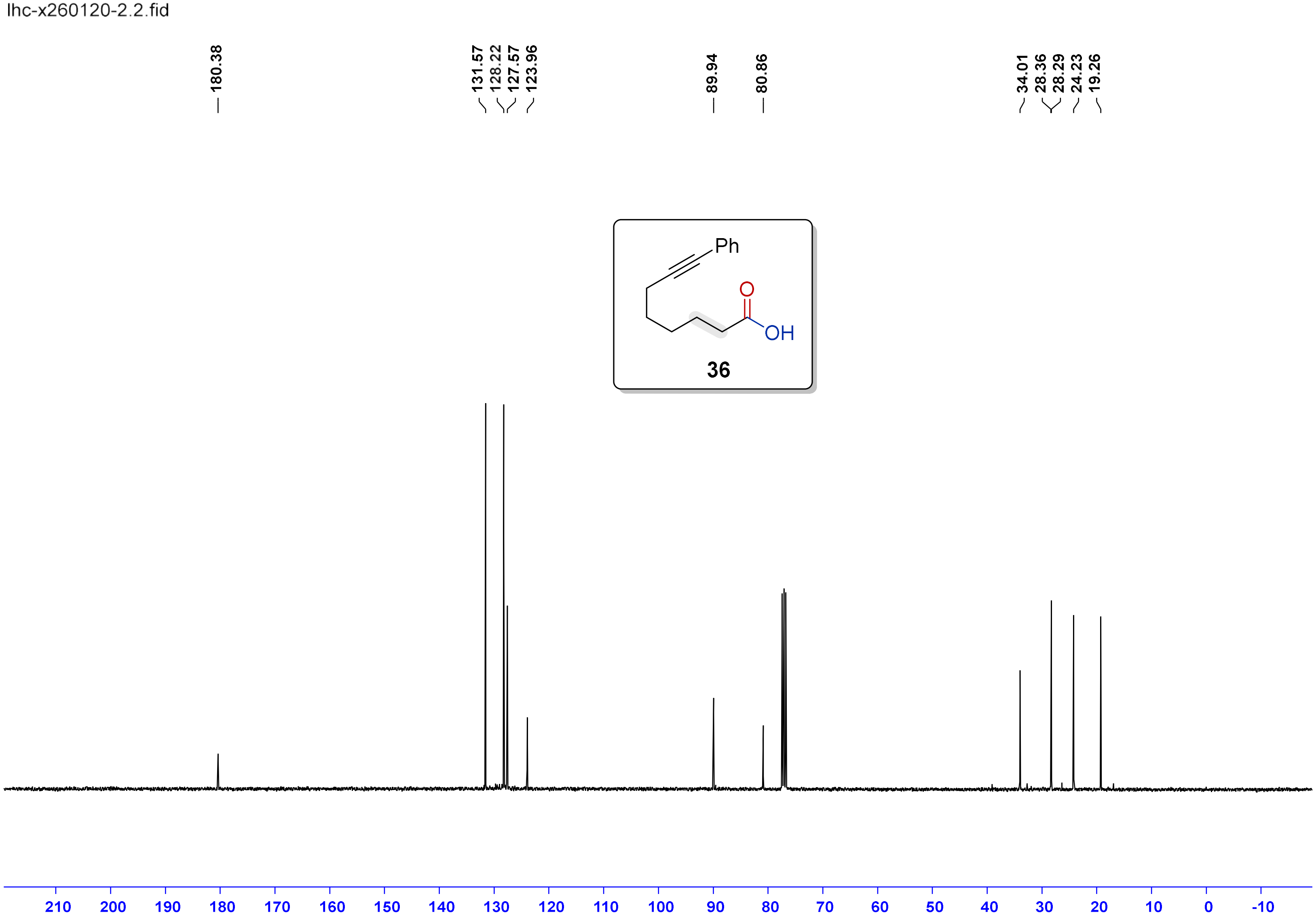

^{1}H NMR spectra for 37

lhc-x250523-5.10.fid — 1H NMR (400 MHz, CDCl3)

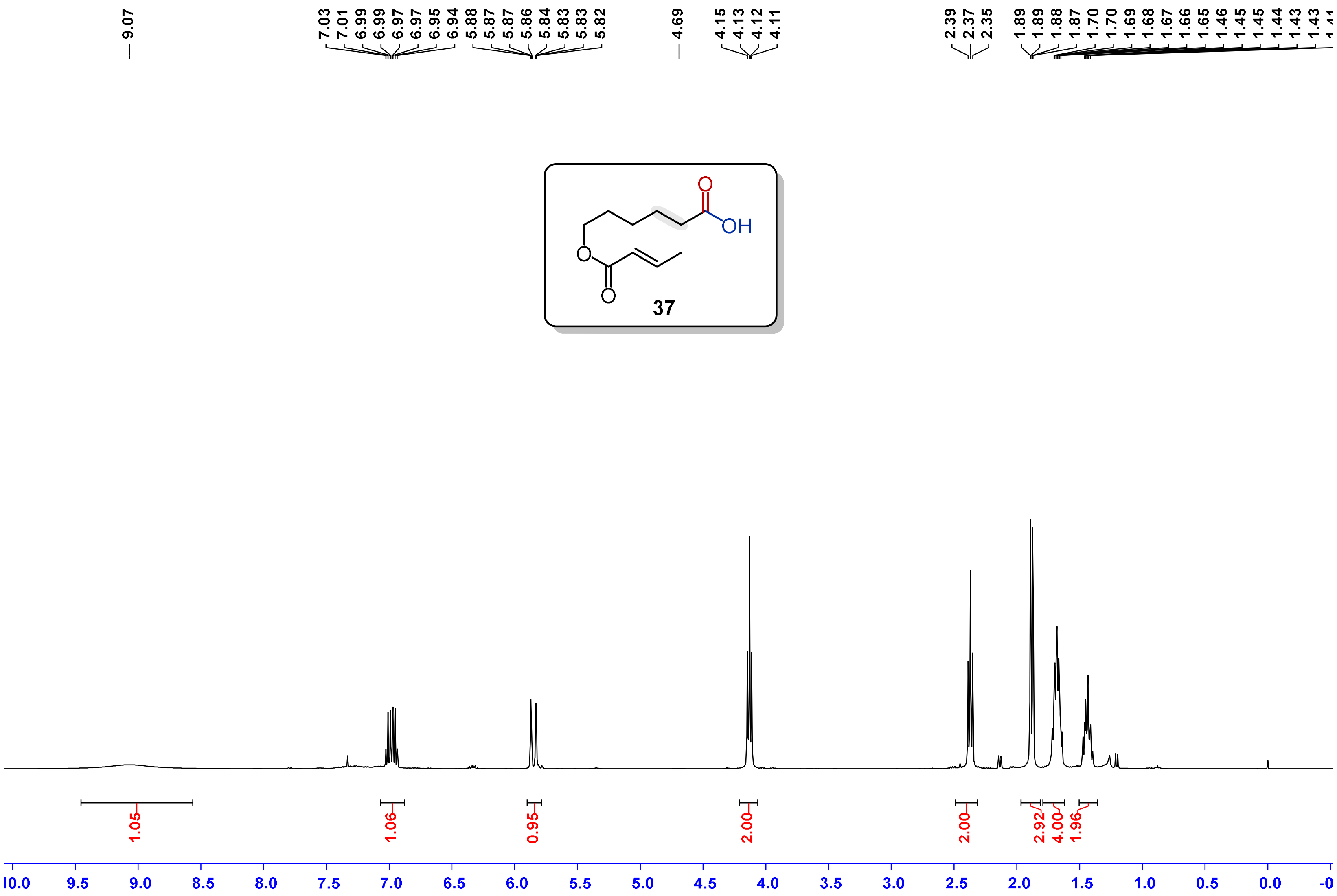

^{13}C NMR spectra for 37

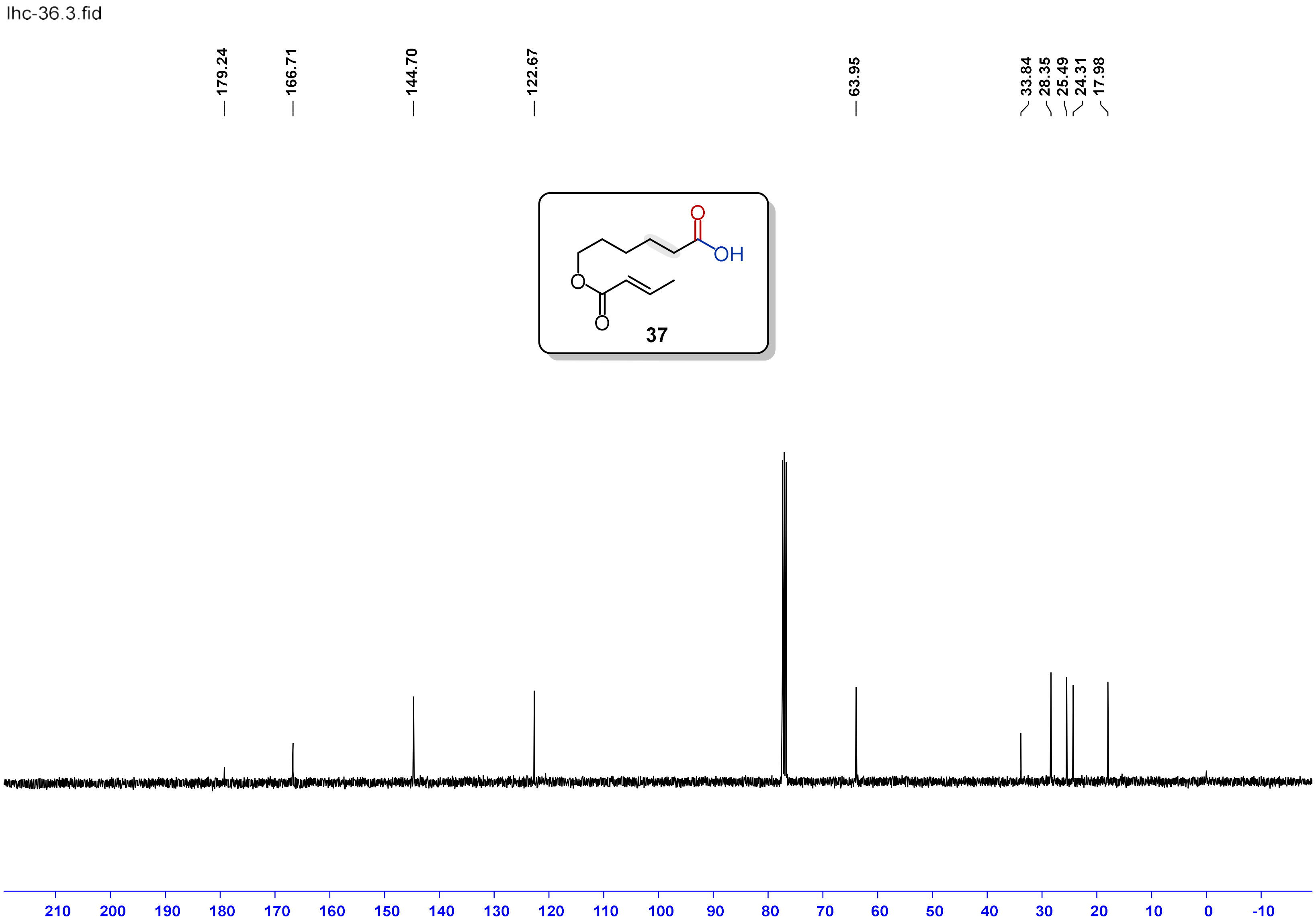

^{1}H NMR spectra for 38

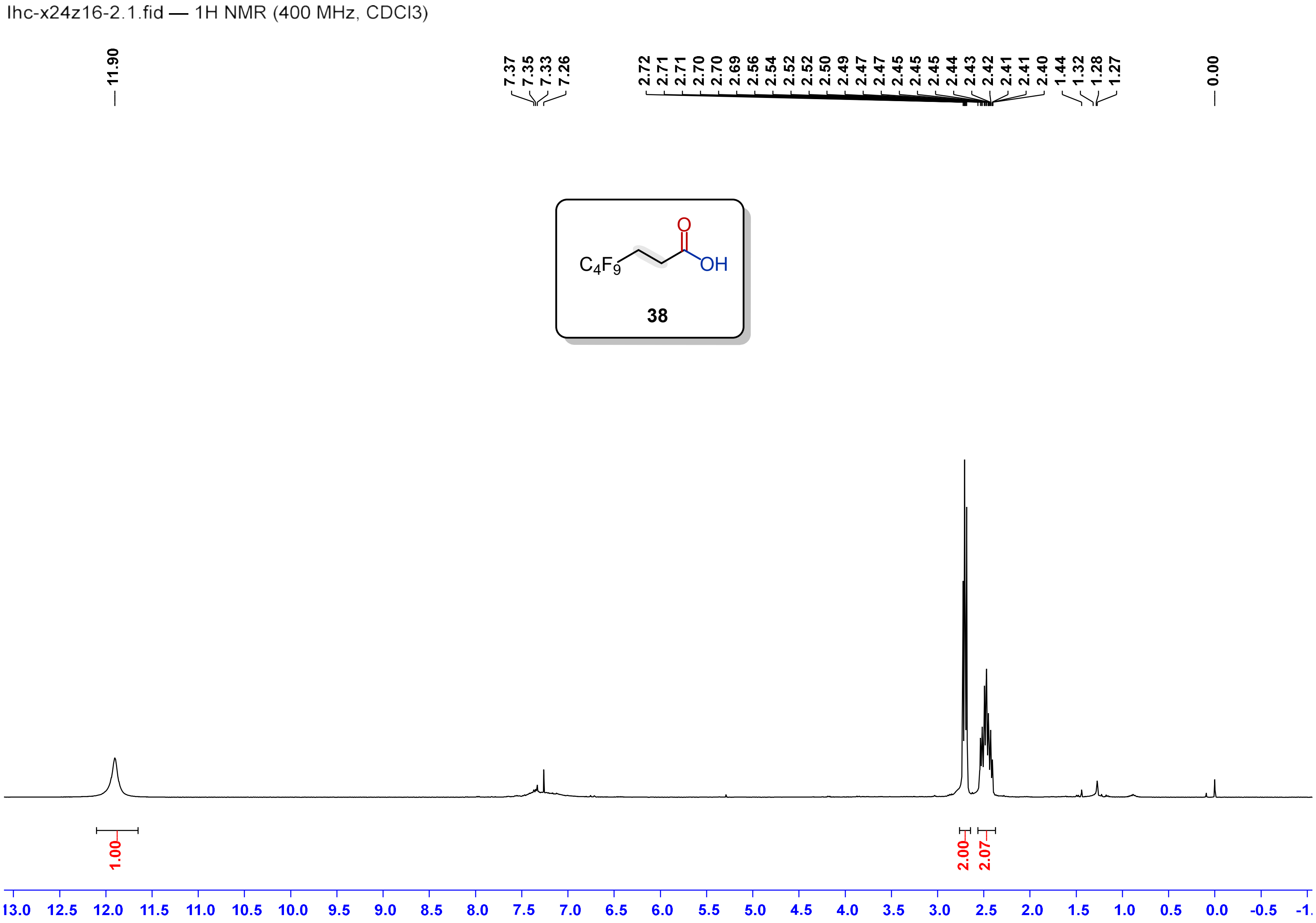

^{13}C NMR spectra for 38

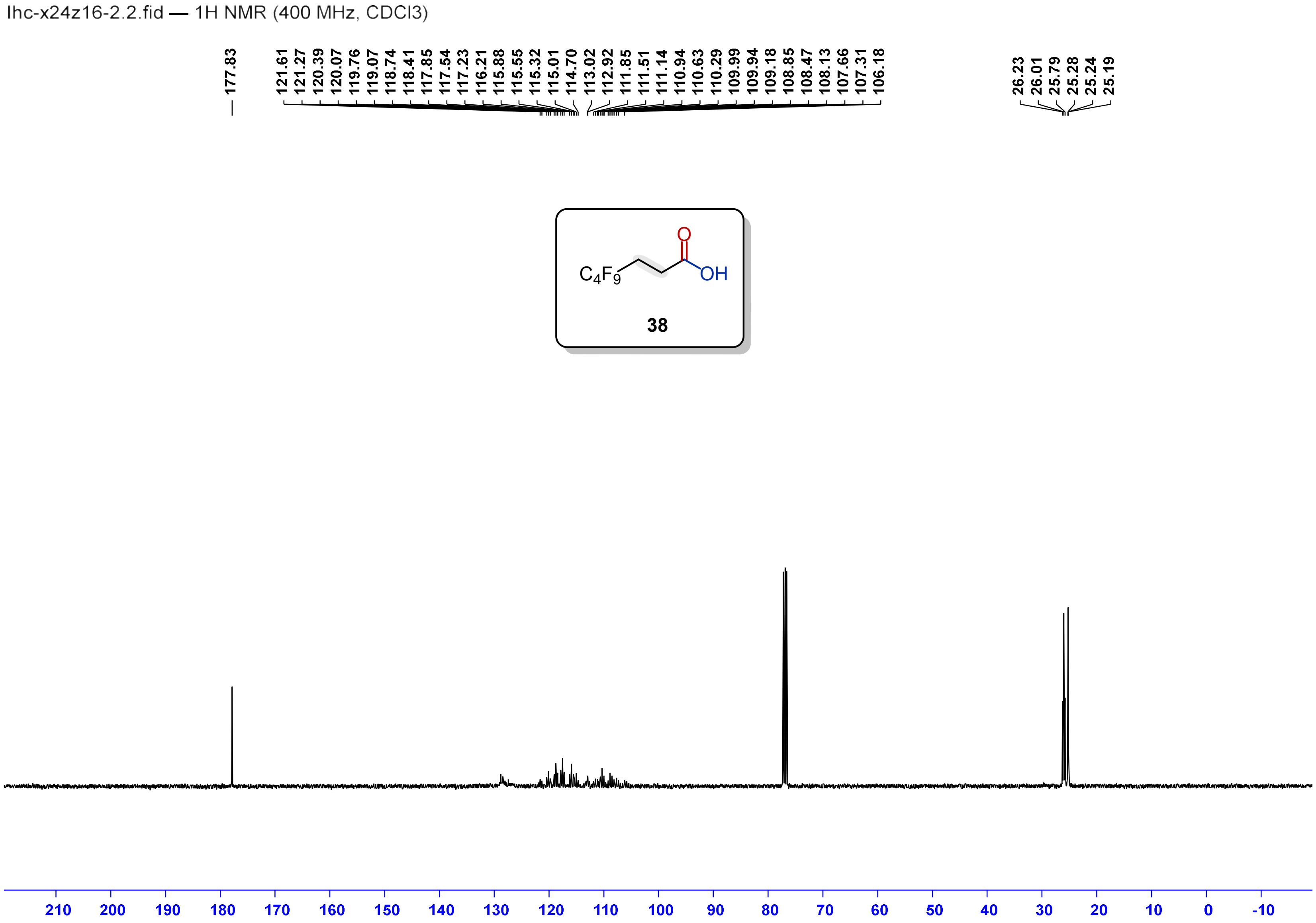

^{19}F NMR spectra for 38

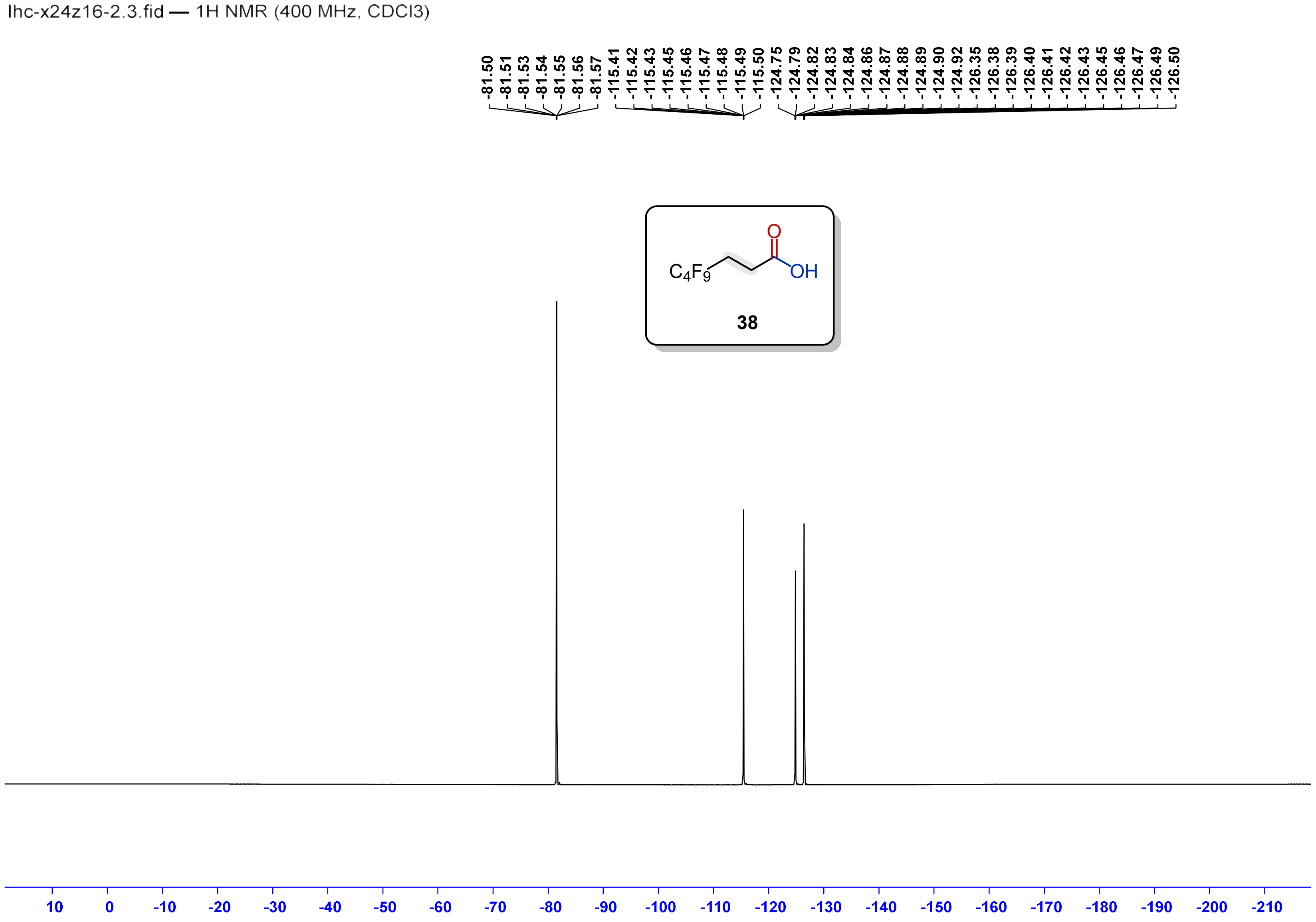

¹H NMR spectra for 39

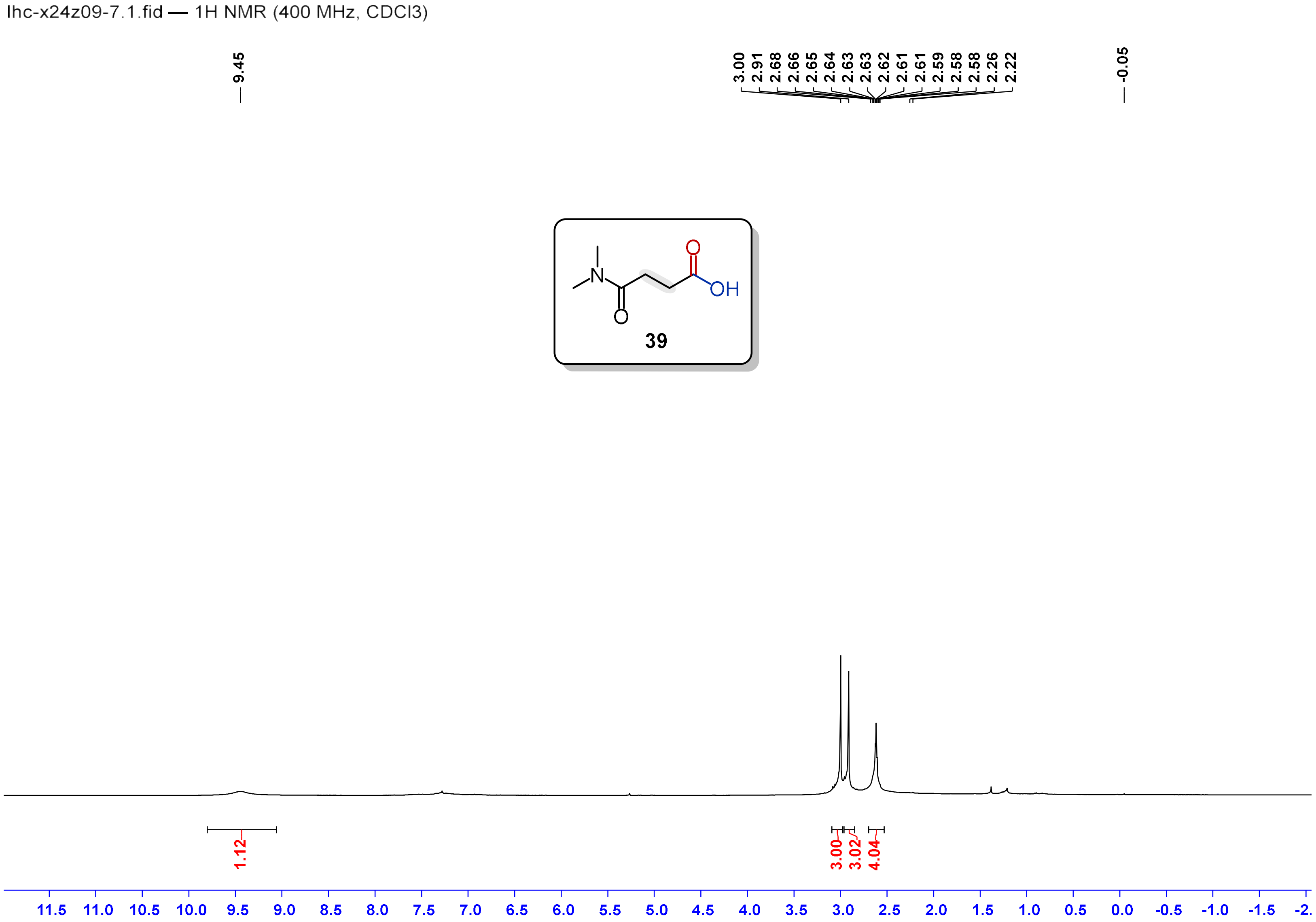

39

^{13}C NMR spectra for 39

lhc-x24z09-7.2.fid — 1H NMR (400 MHz, CDCl3)

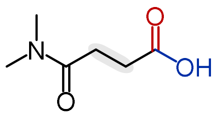

39

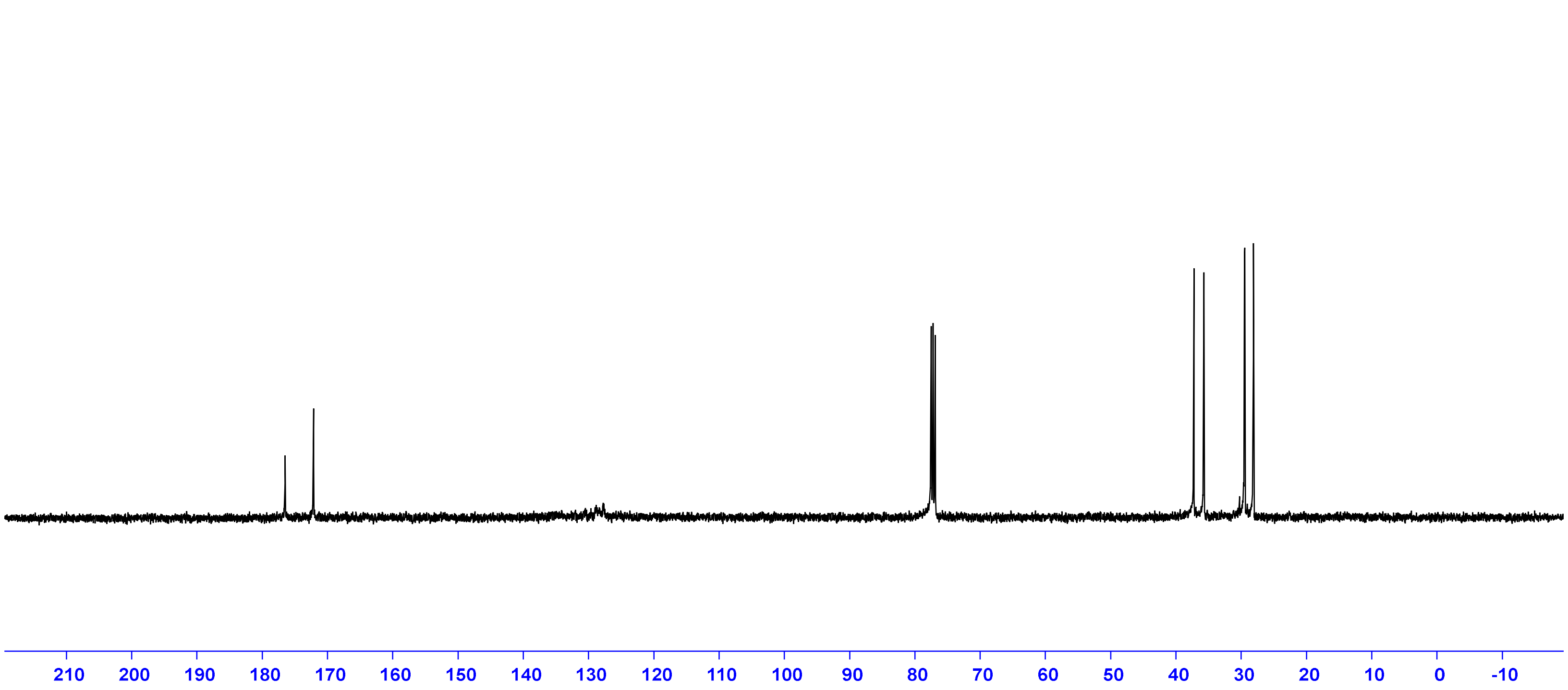

^{1}H NMR spectra for40

lhc-x24z25-1.1.fid — 1H NMR (400 MHz, CDCl3)

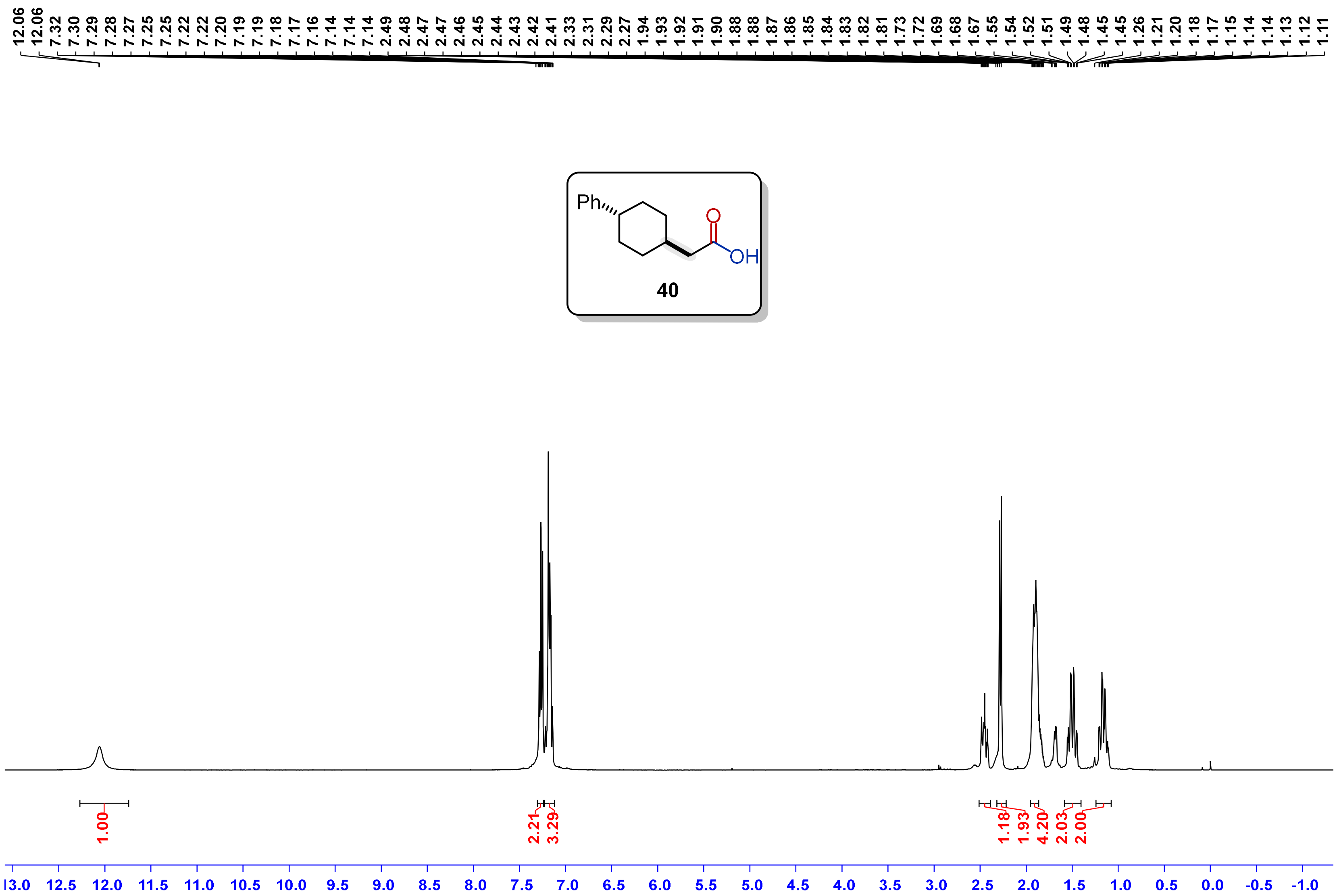

^{13}C NMR spectra for 40

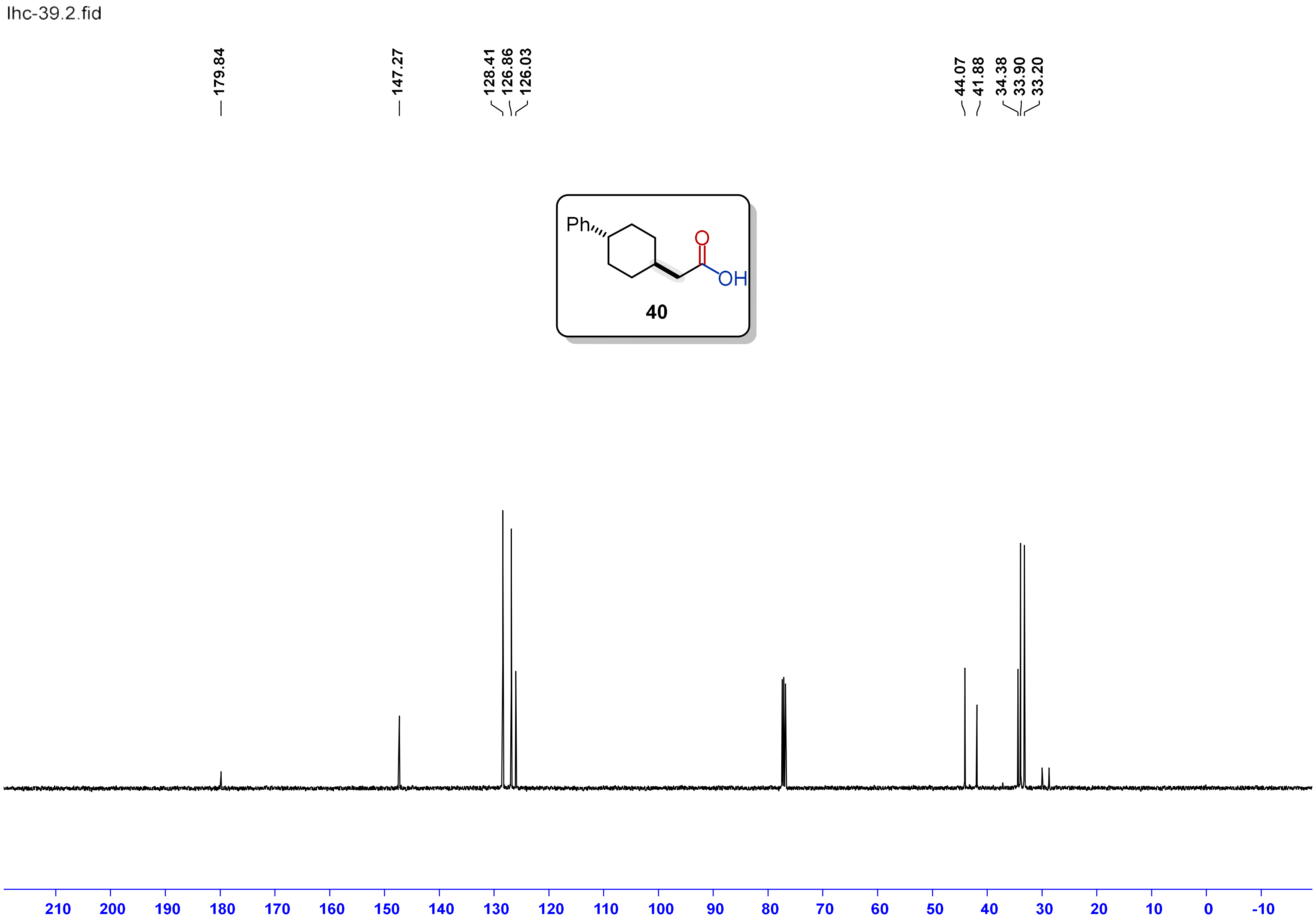

^{1}H NMR spectra for 41

lhc-x24z25-7.1.fid — 1H NMR (400 MHz, CDCl3)

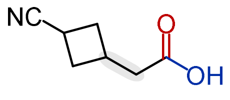

41

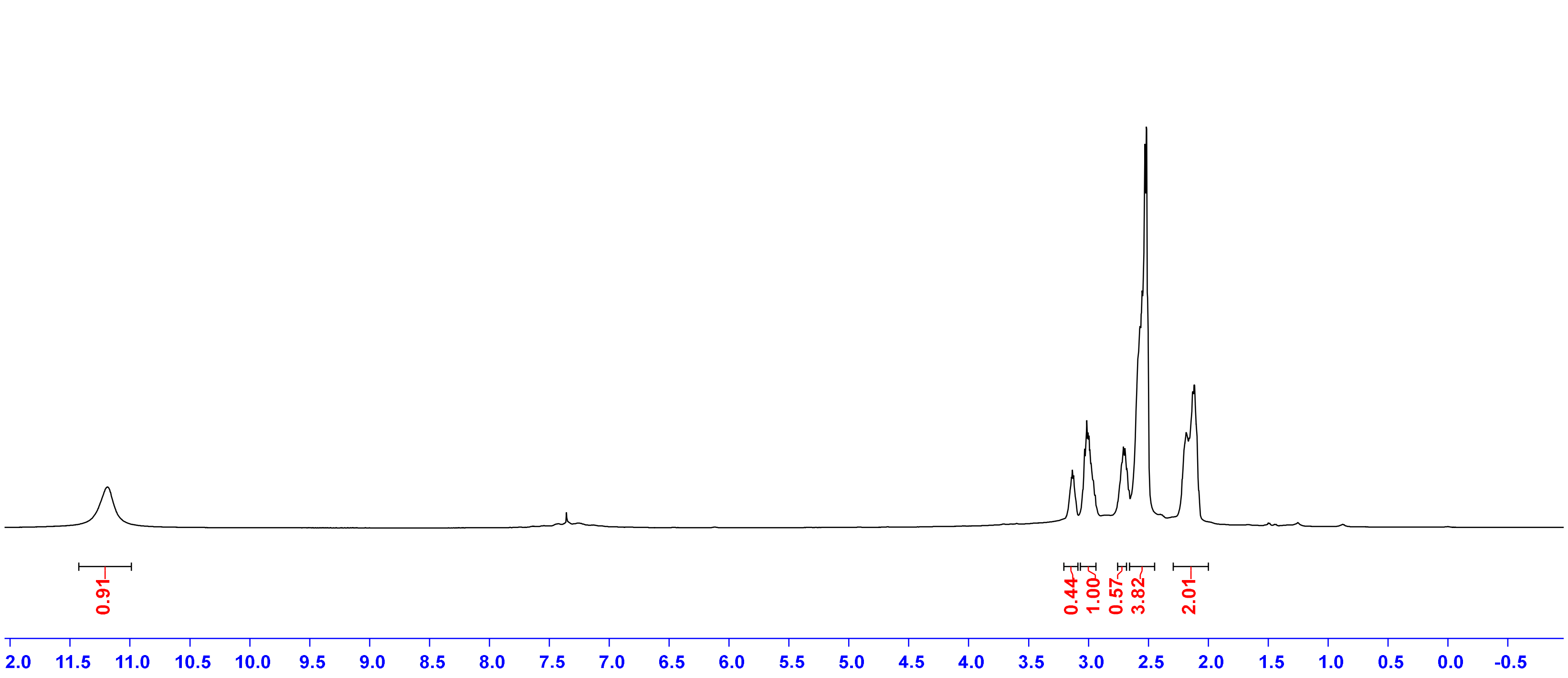

^{13}C NMR spectra for 41

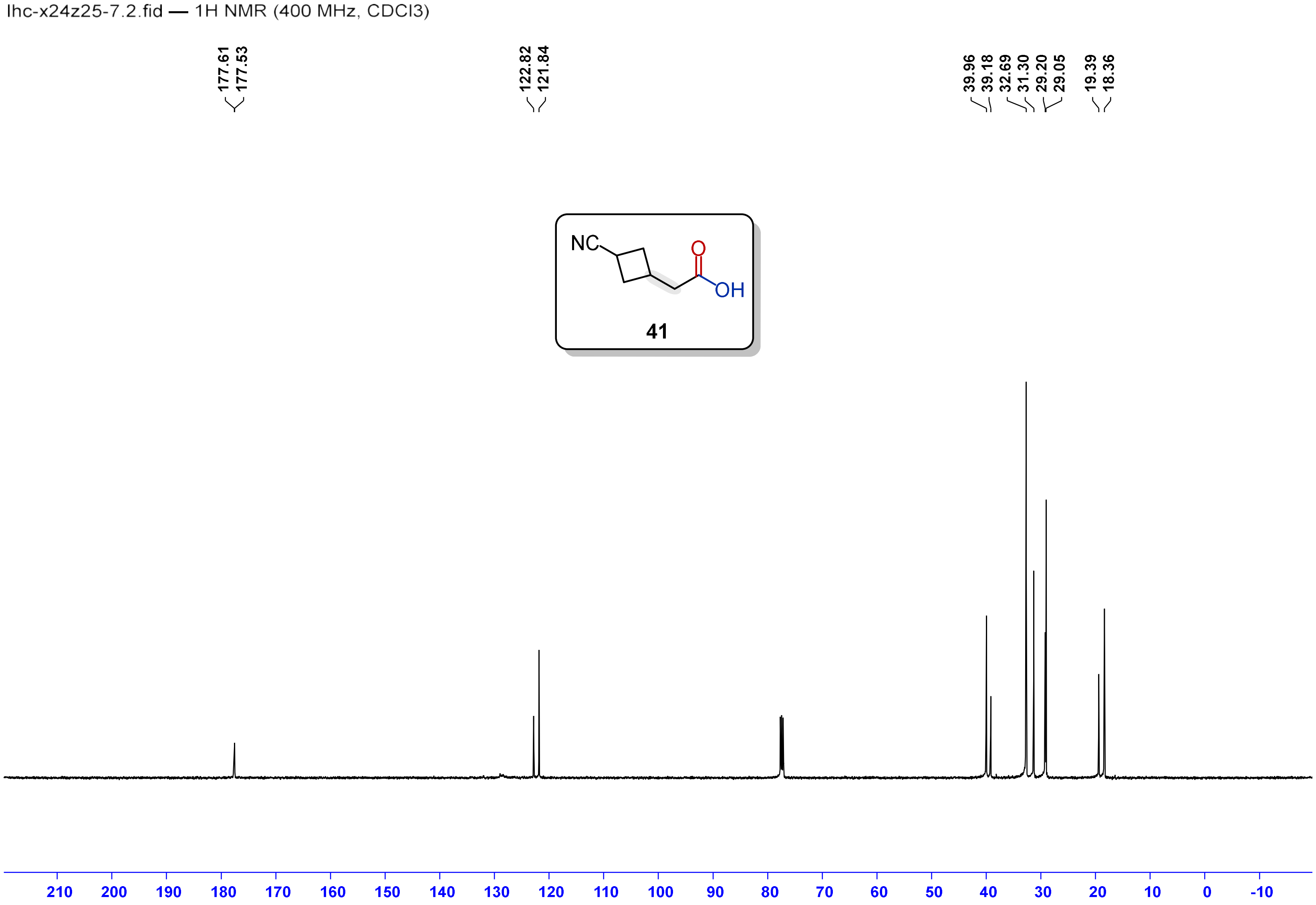

^{1}H NMR spectra for 42

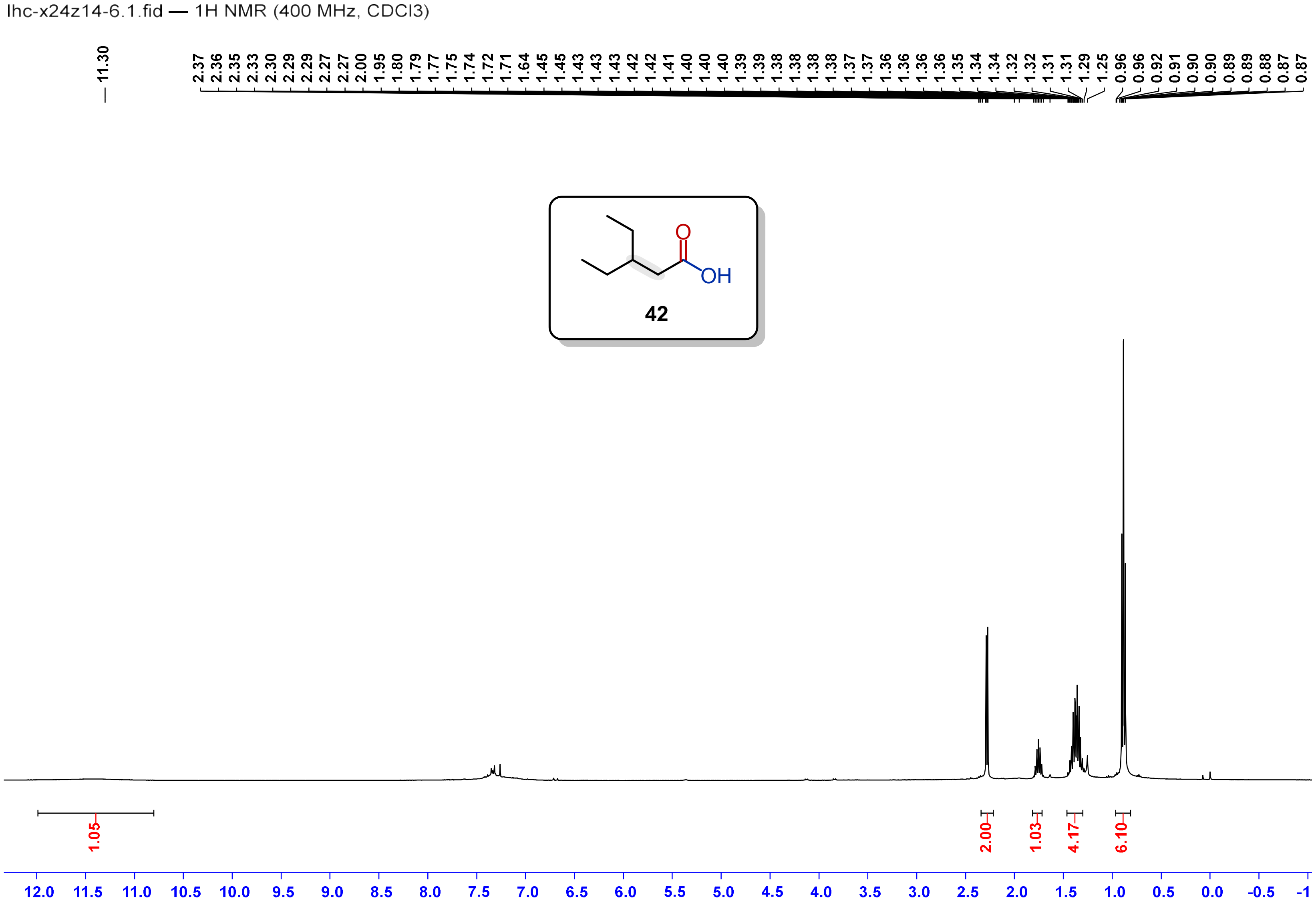

^{13}C NMR spectra for 42

lhc-x24z14-6.2.fid — 1H NMR (400 MHz, CDCl3)

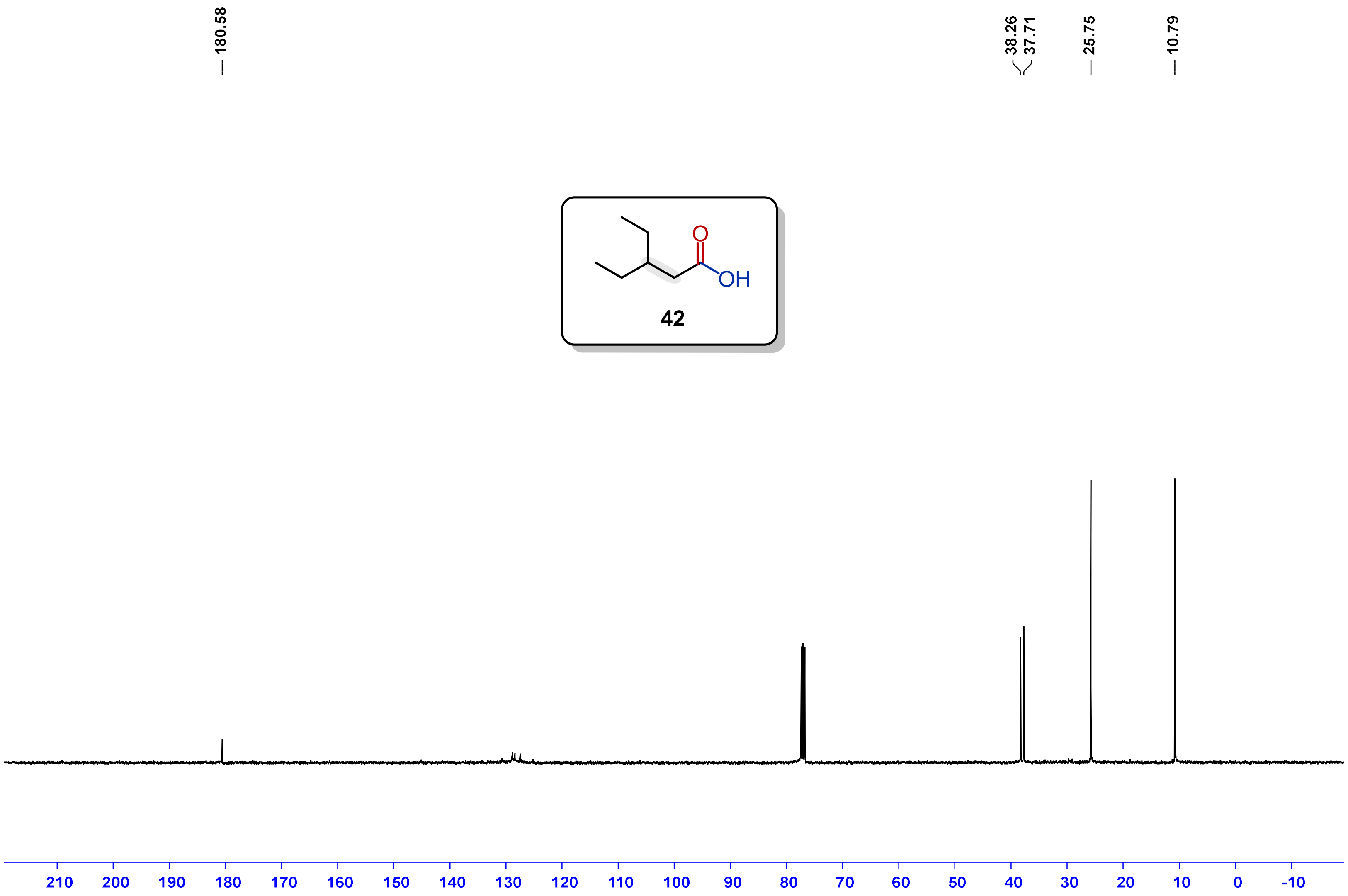

^{1}H NMR spectra for 43

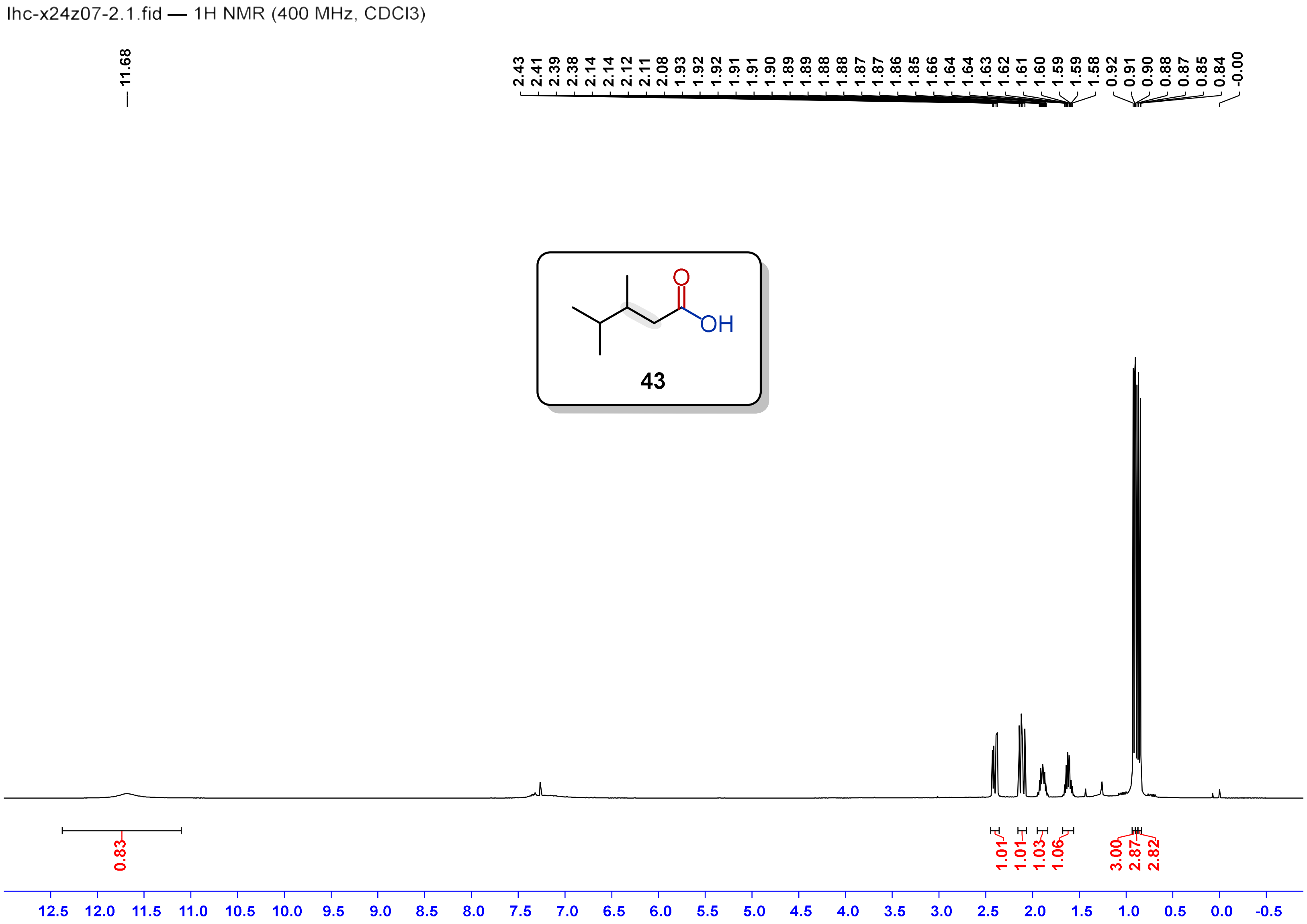

^{13}C NMR spectra for 43

lhc-x24z07-2.2.fid — 1H NMR (400 MHz, CDCl3)

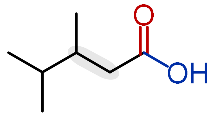

43

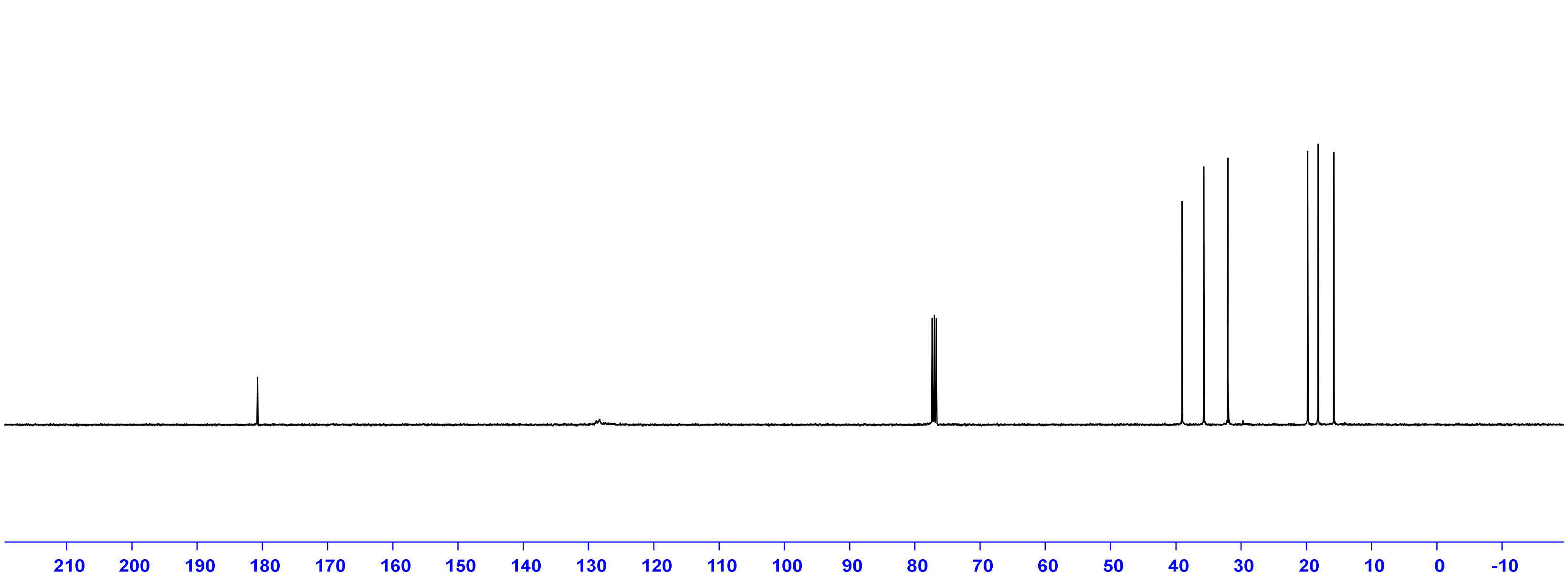

^{1}H NMR spectra for 44

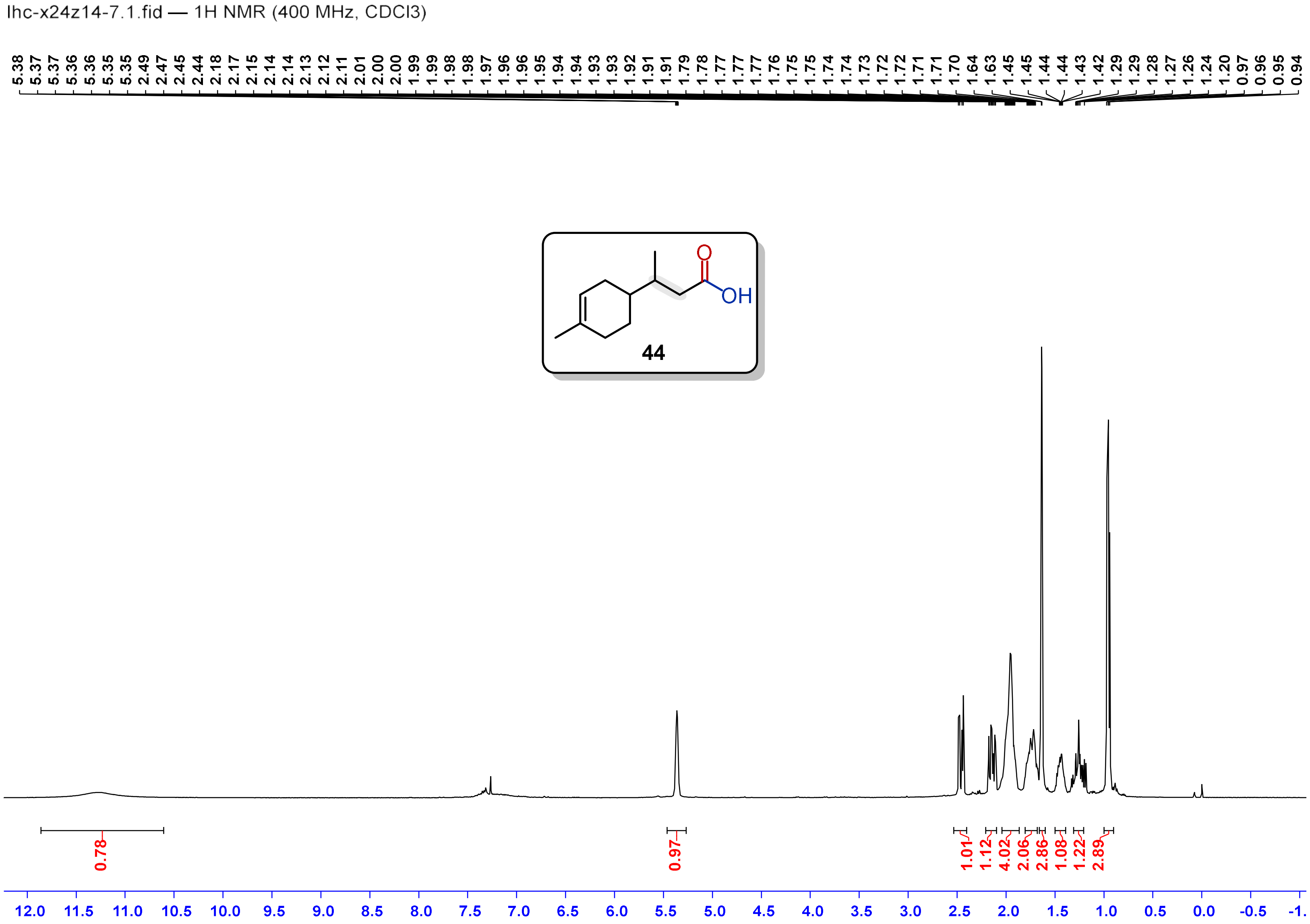

^{13}C NMR spectra for 44

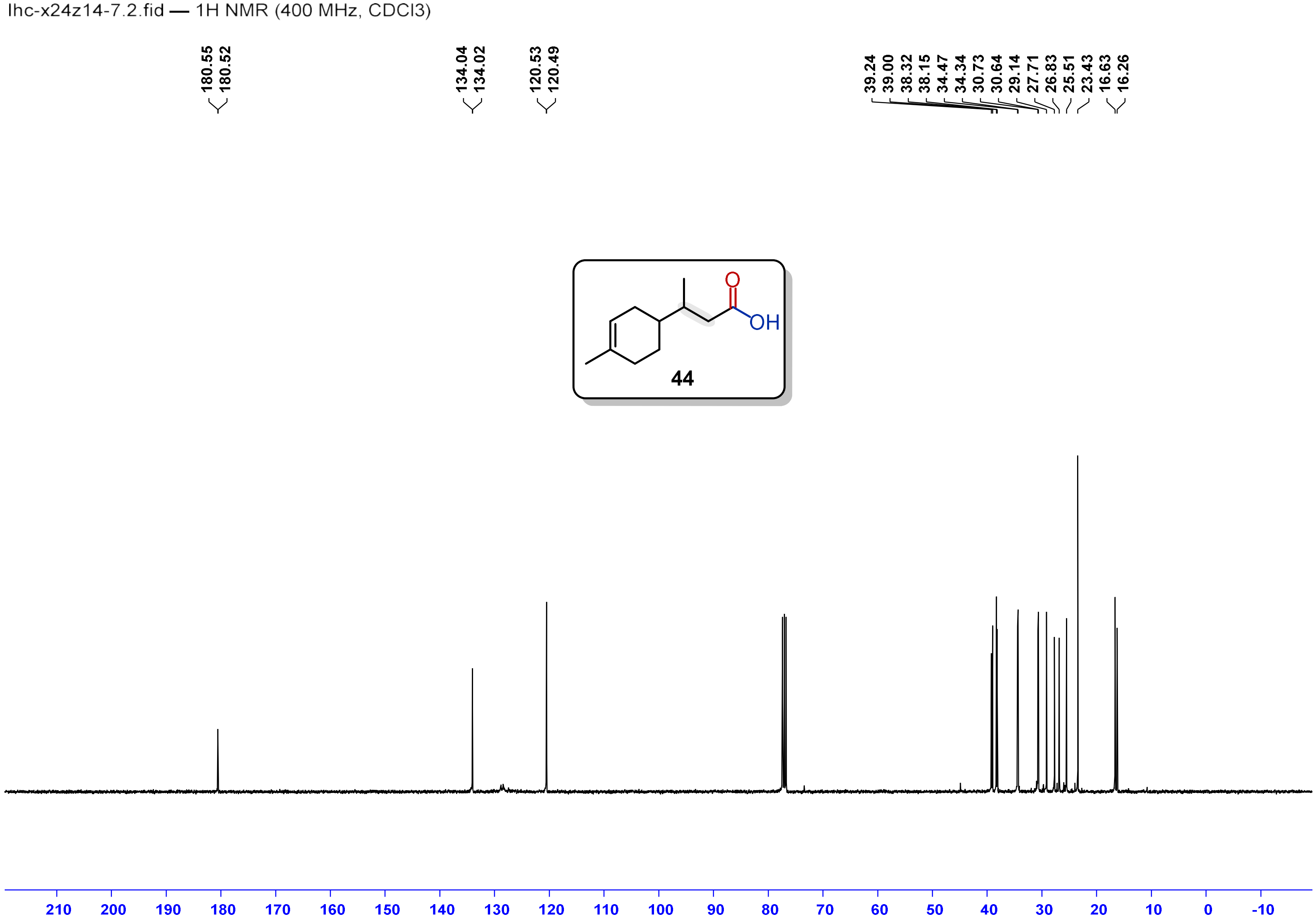

44

^{1}H NMR spectra for 45

lhc-x24z14-1-2.1.fid — 1H NMR (400 MHz, CDCl3)

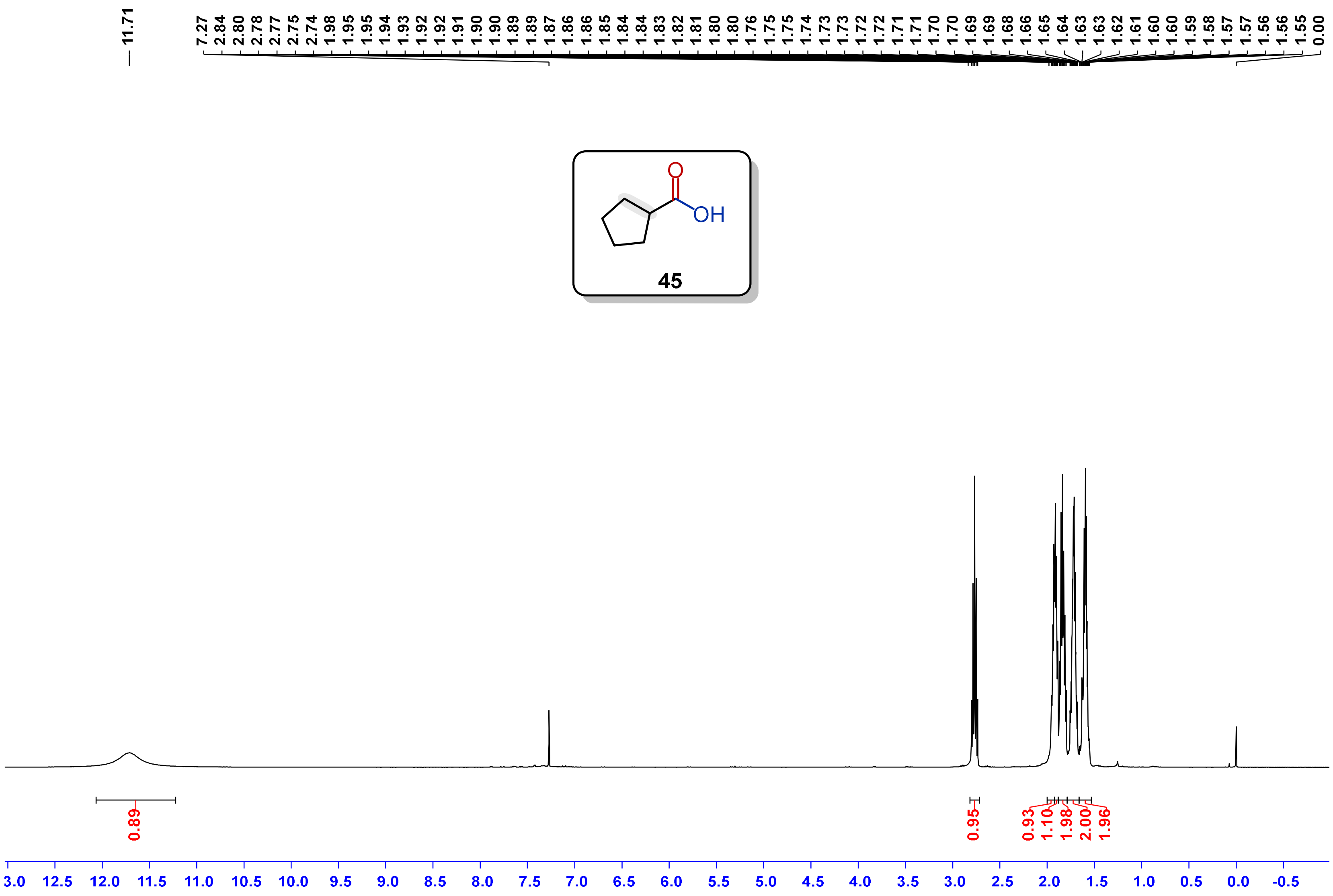

^{13}C NMR spectra for 45

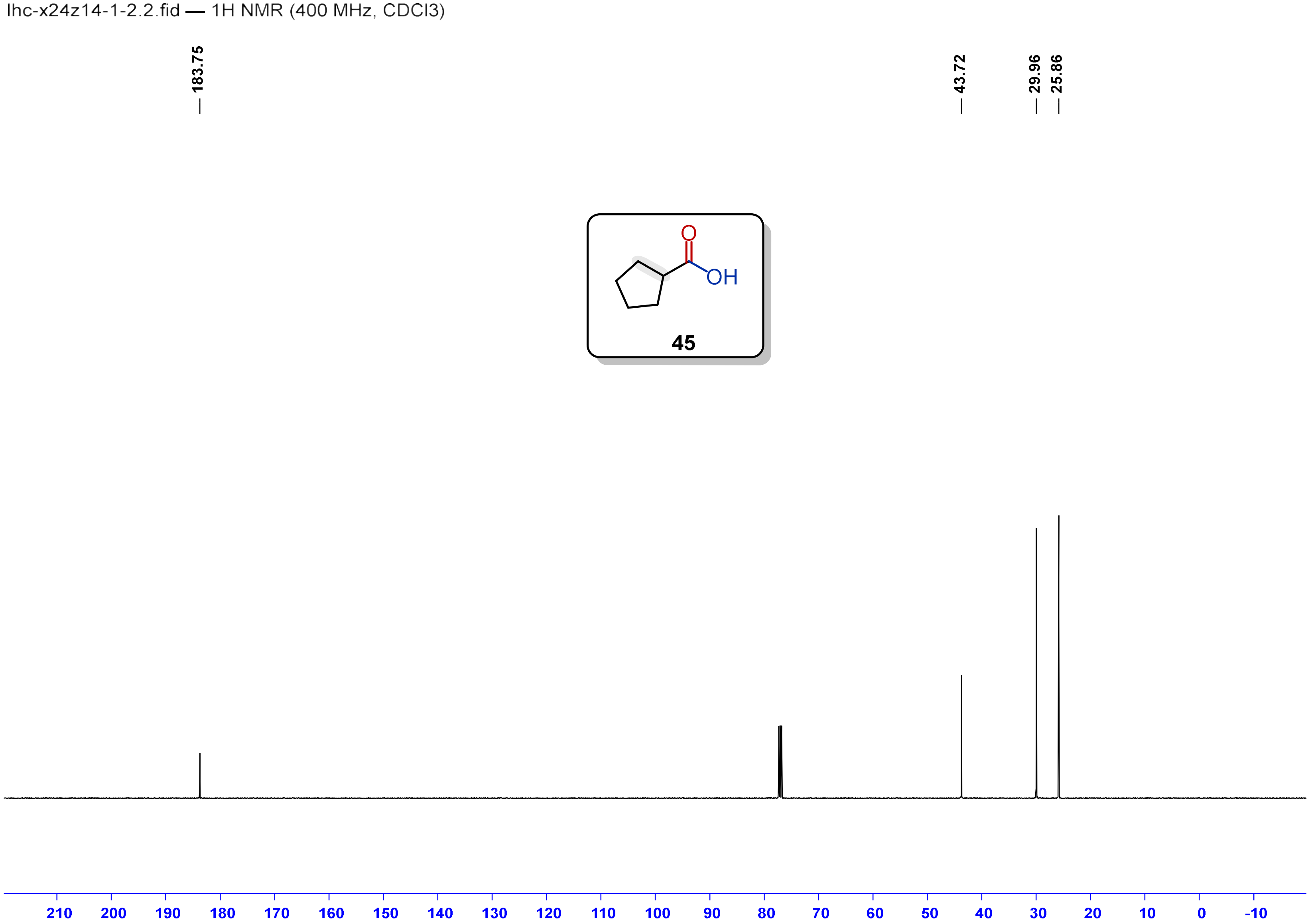

^{1}H NMR spectra for 46

lhc-x24z07-3-1.1.fid — 1H NMR (400 MHz, CDCl3)

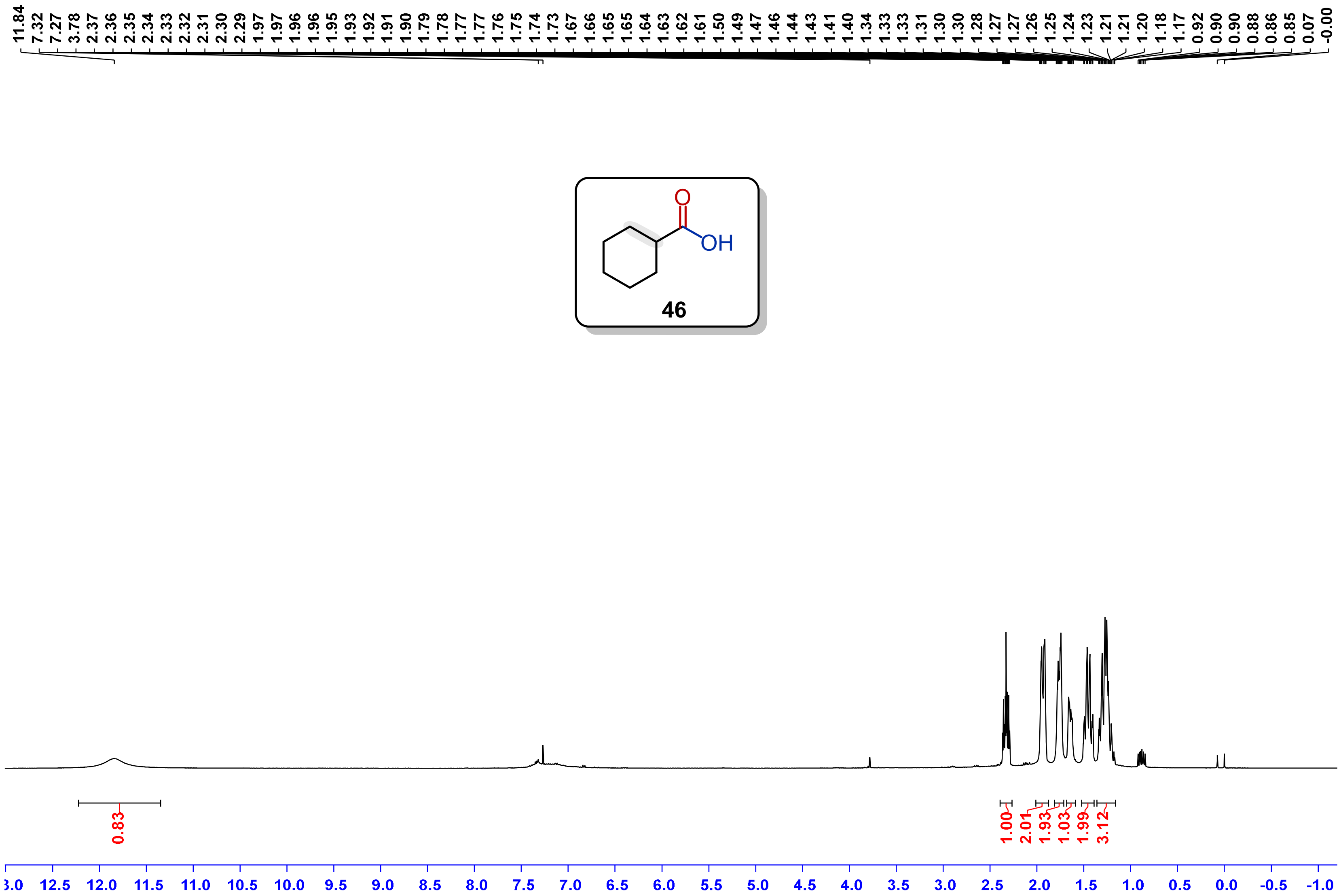

^{13}C NMR spectra for 46

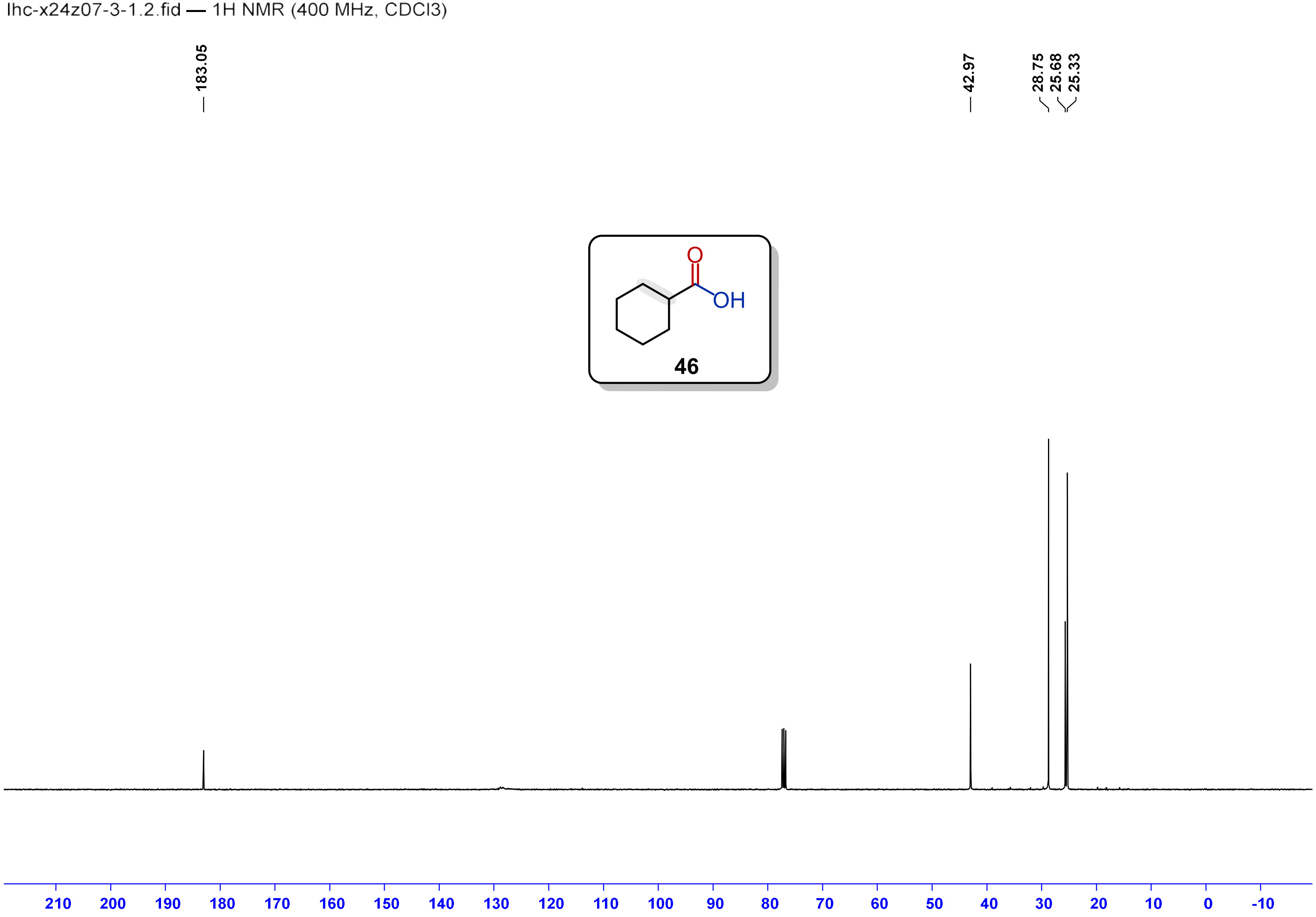

^{1}H NMR spectra for 47

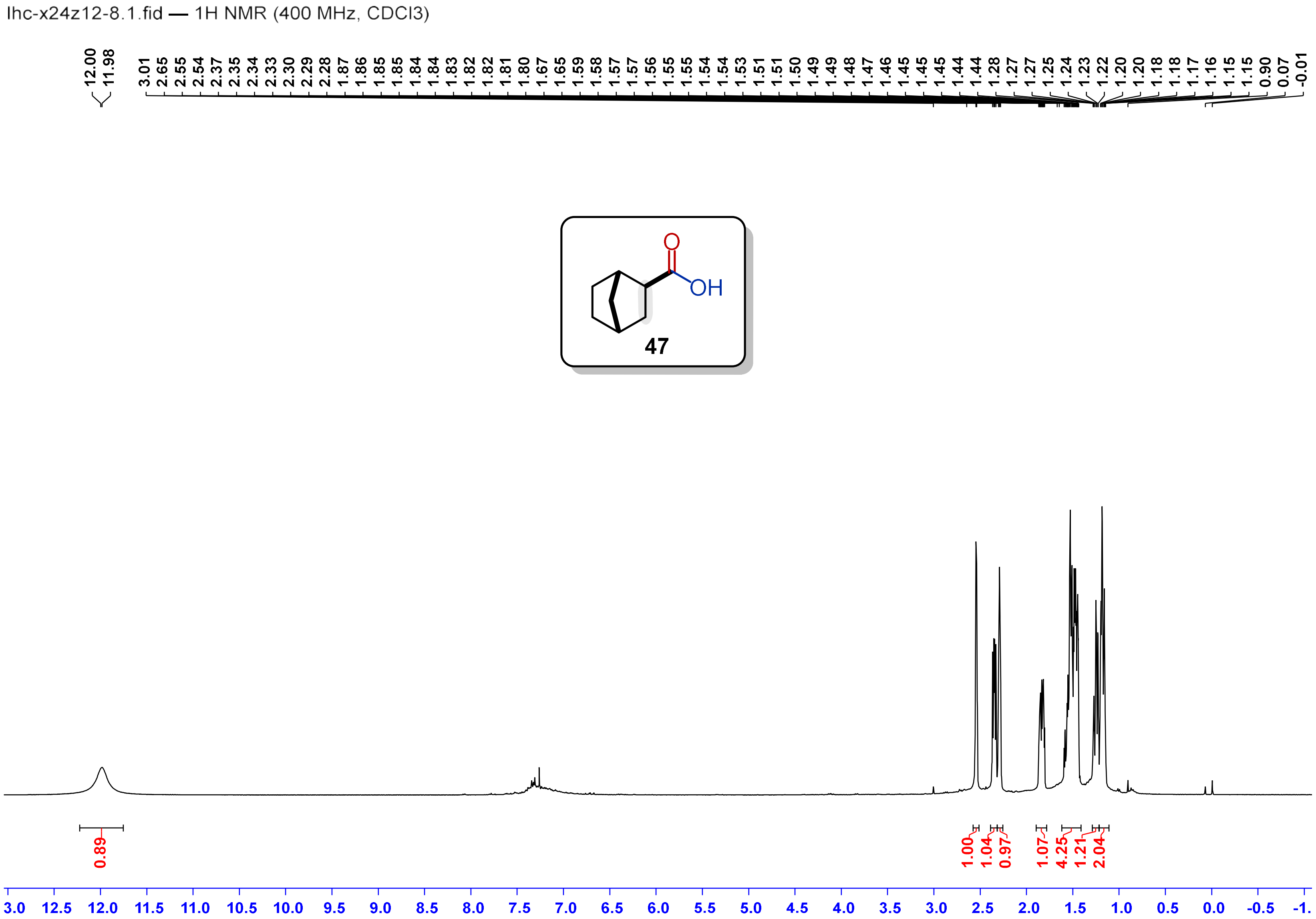

^{13}C NMR spectra for 47

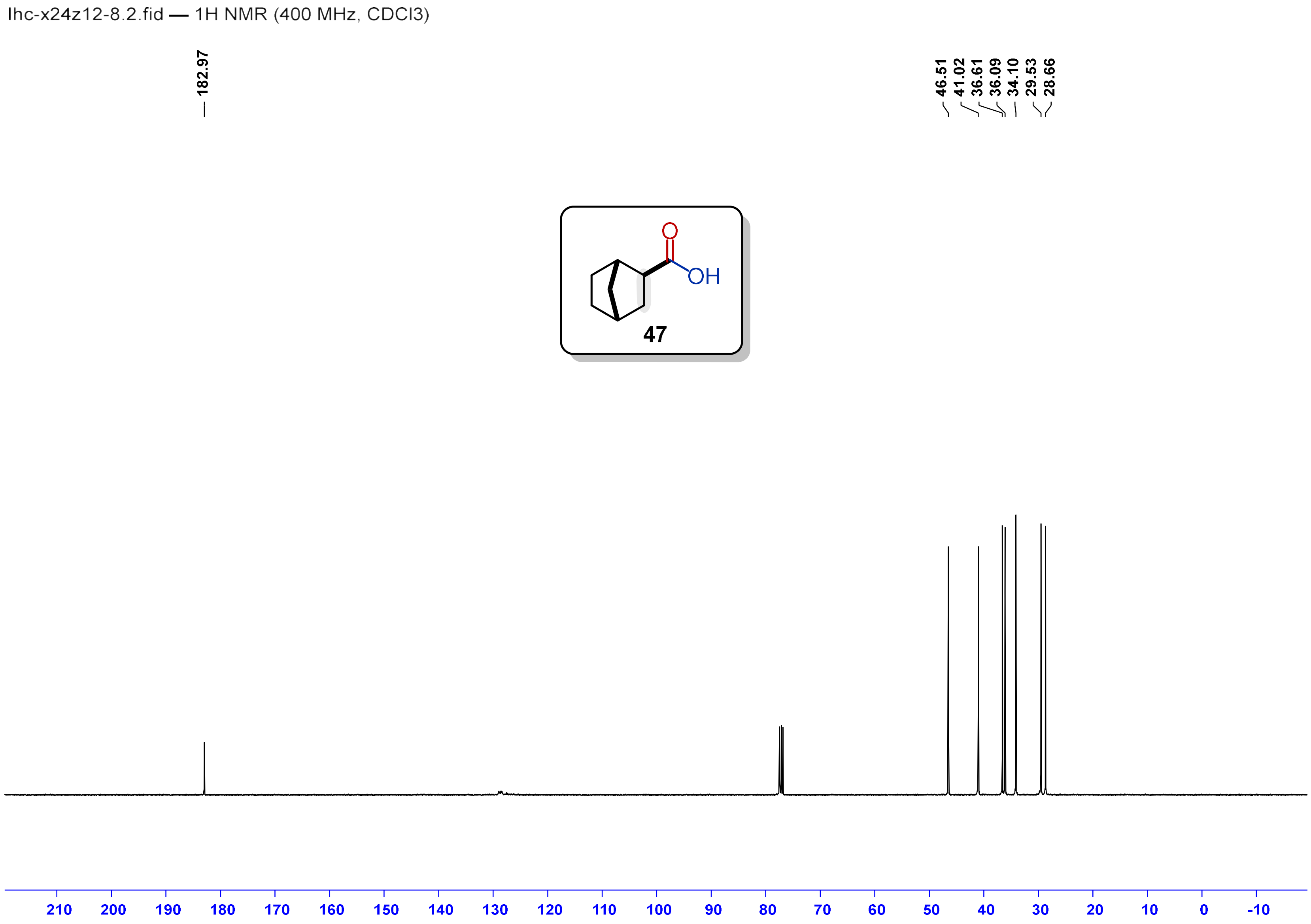

^{1}H NMR spectra for 48

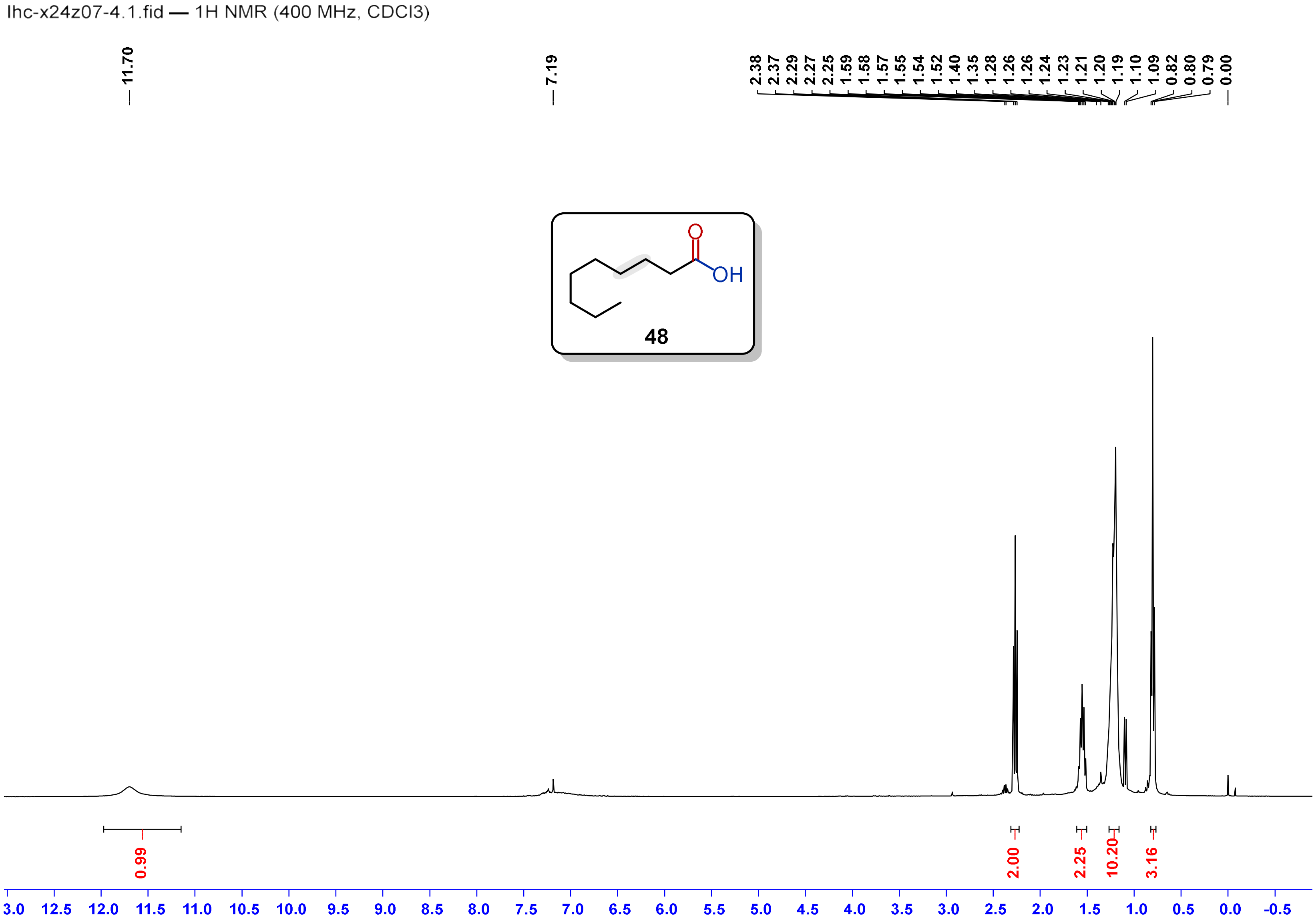

^{13}C NMR spectra for 48

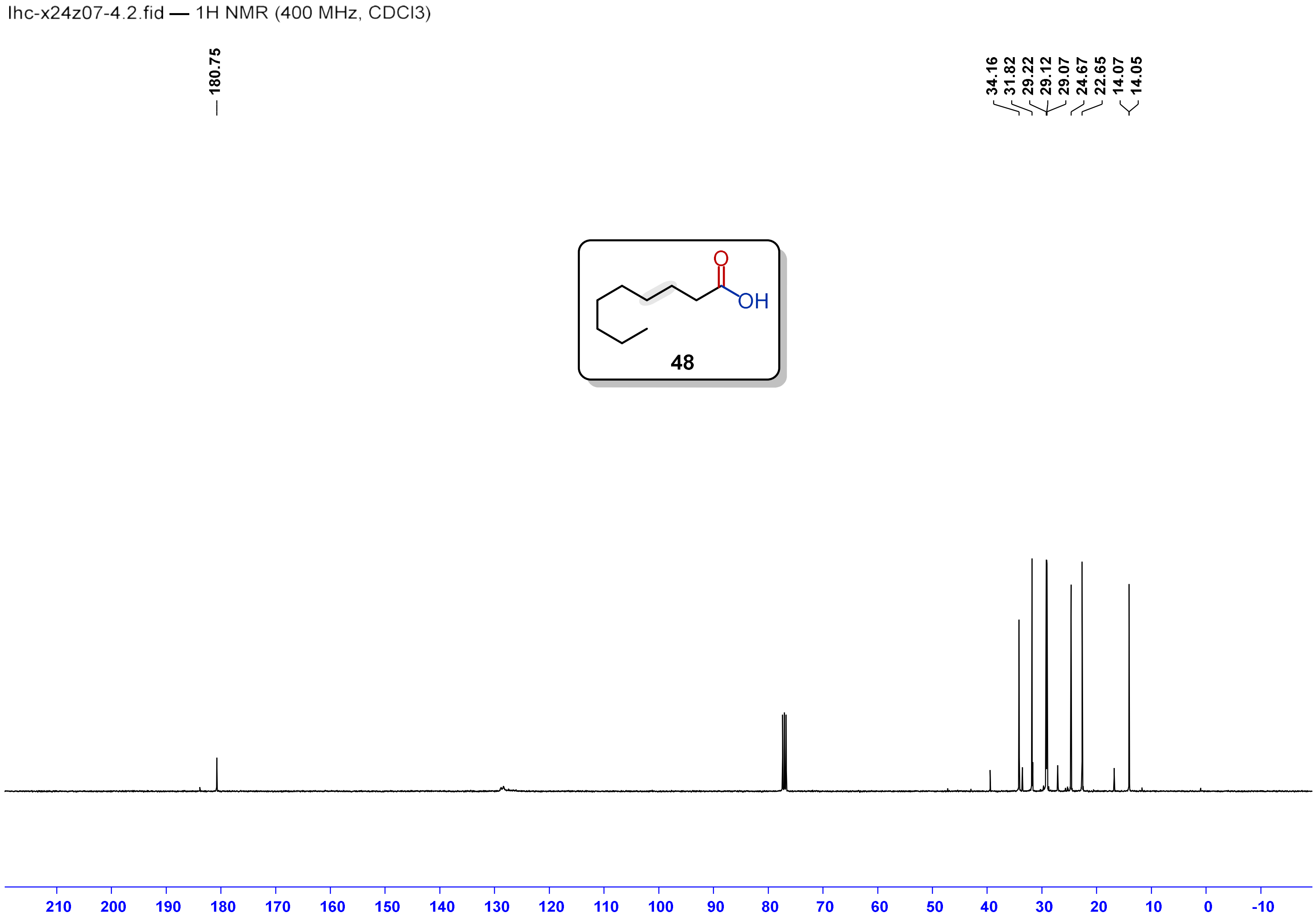

^{1}H NMR spectra for 49

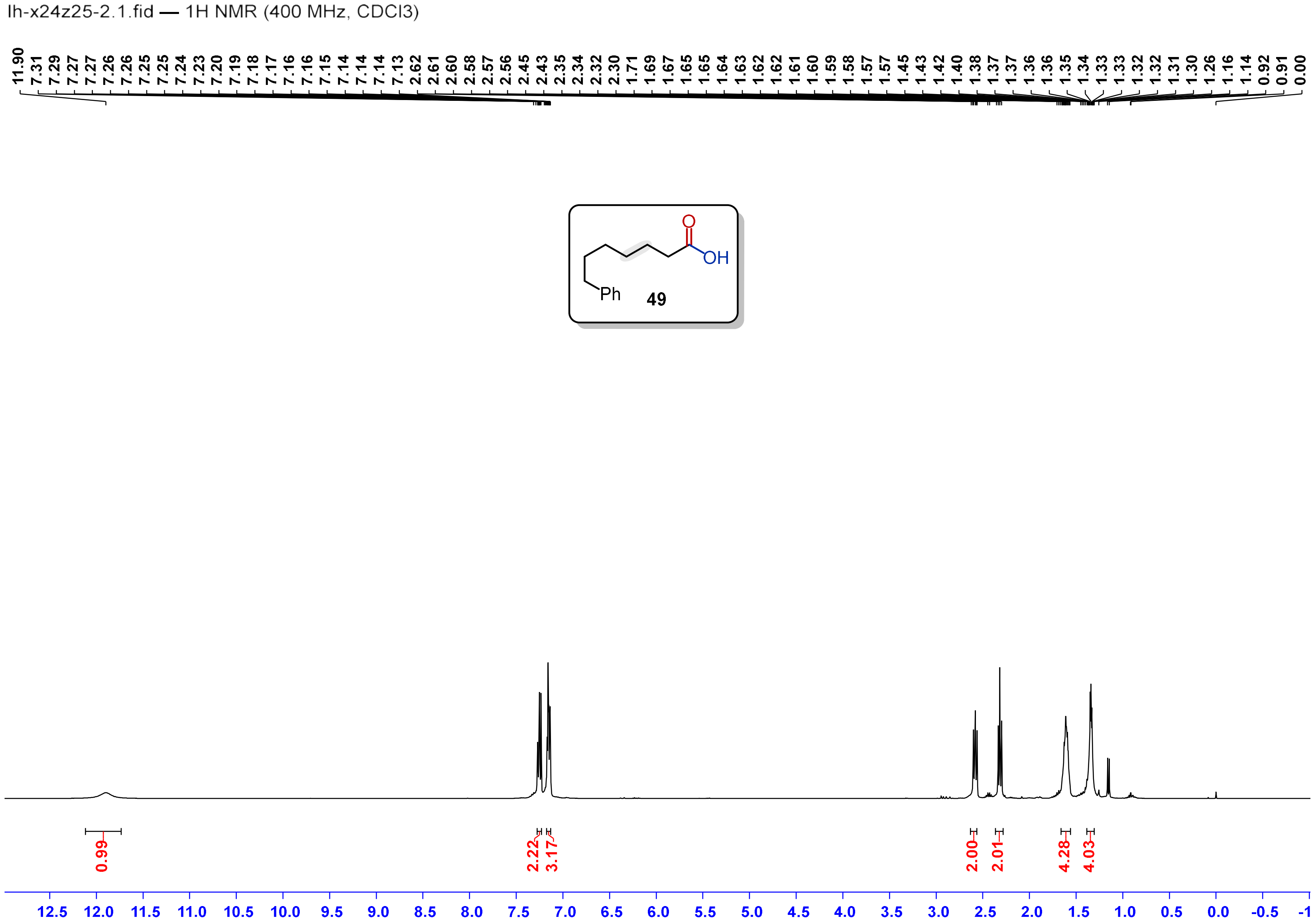

^{13}C NMR spectra for 49

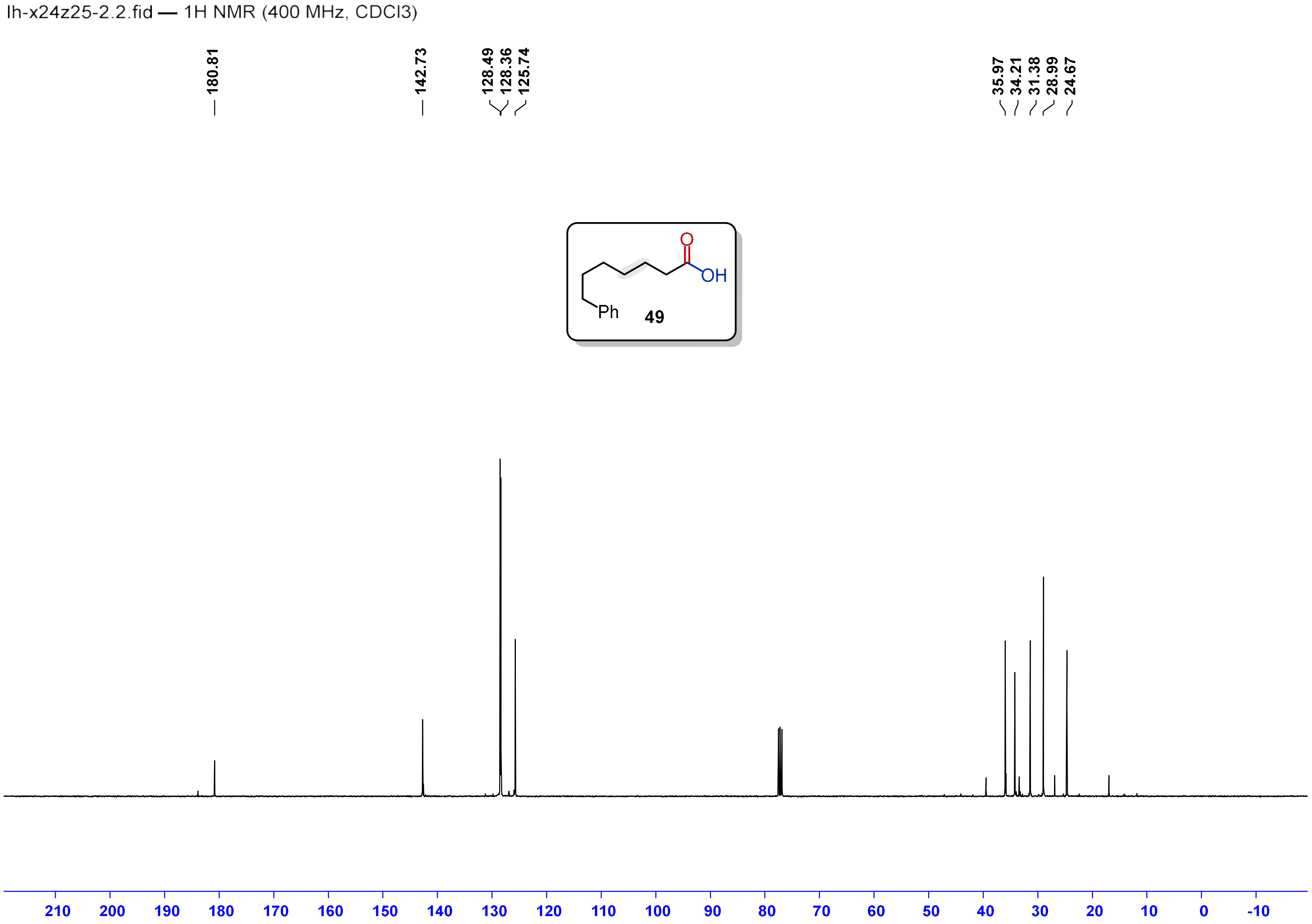

1H NMR spectra for 50

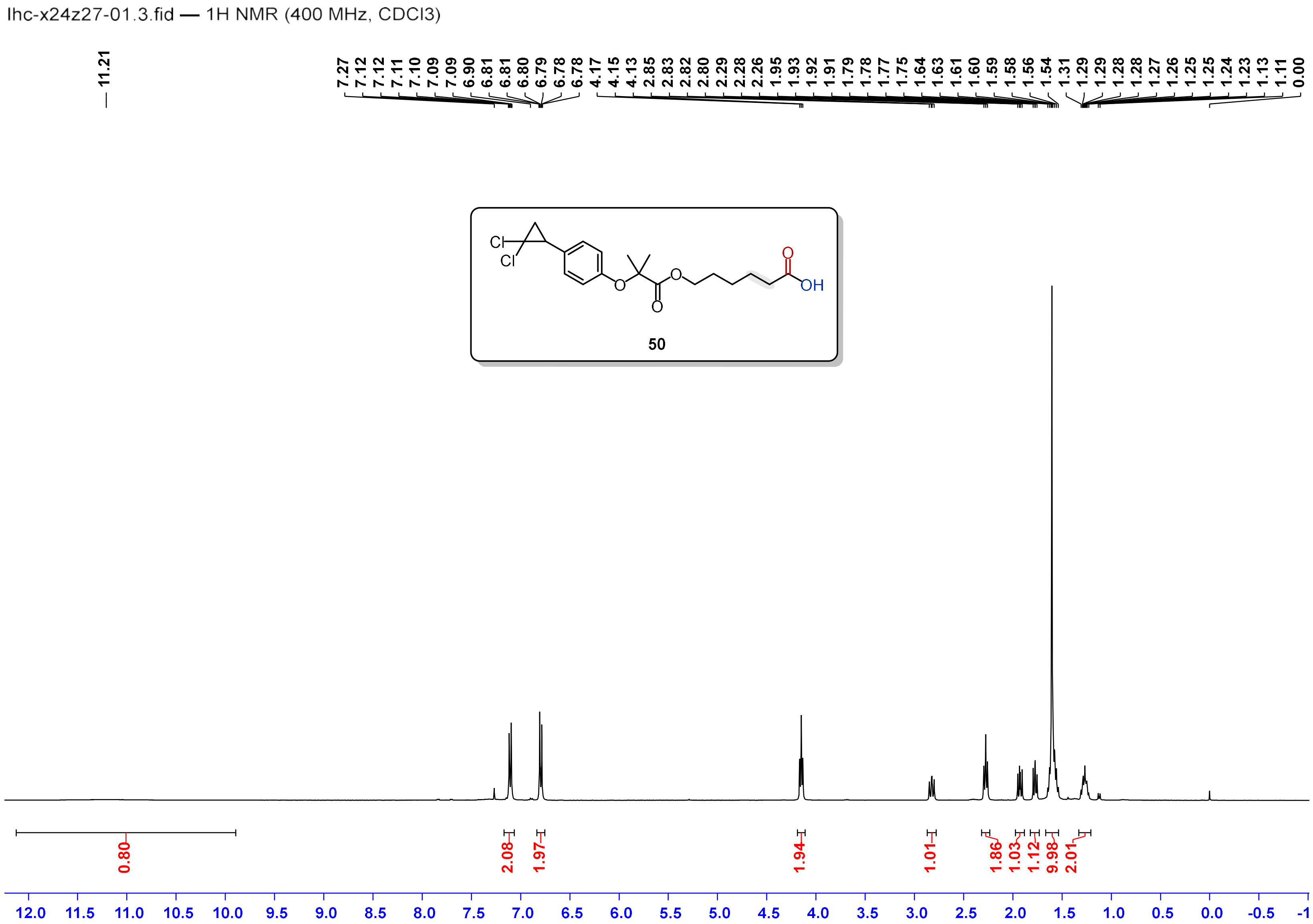

^{13}C NMR spectra for 50

lhc-x24z27-1.2.fid — 1H NMR (400 MHz, CDCl3)

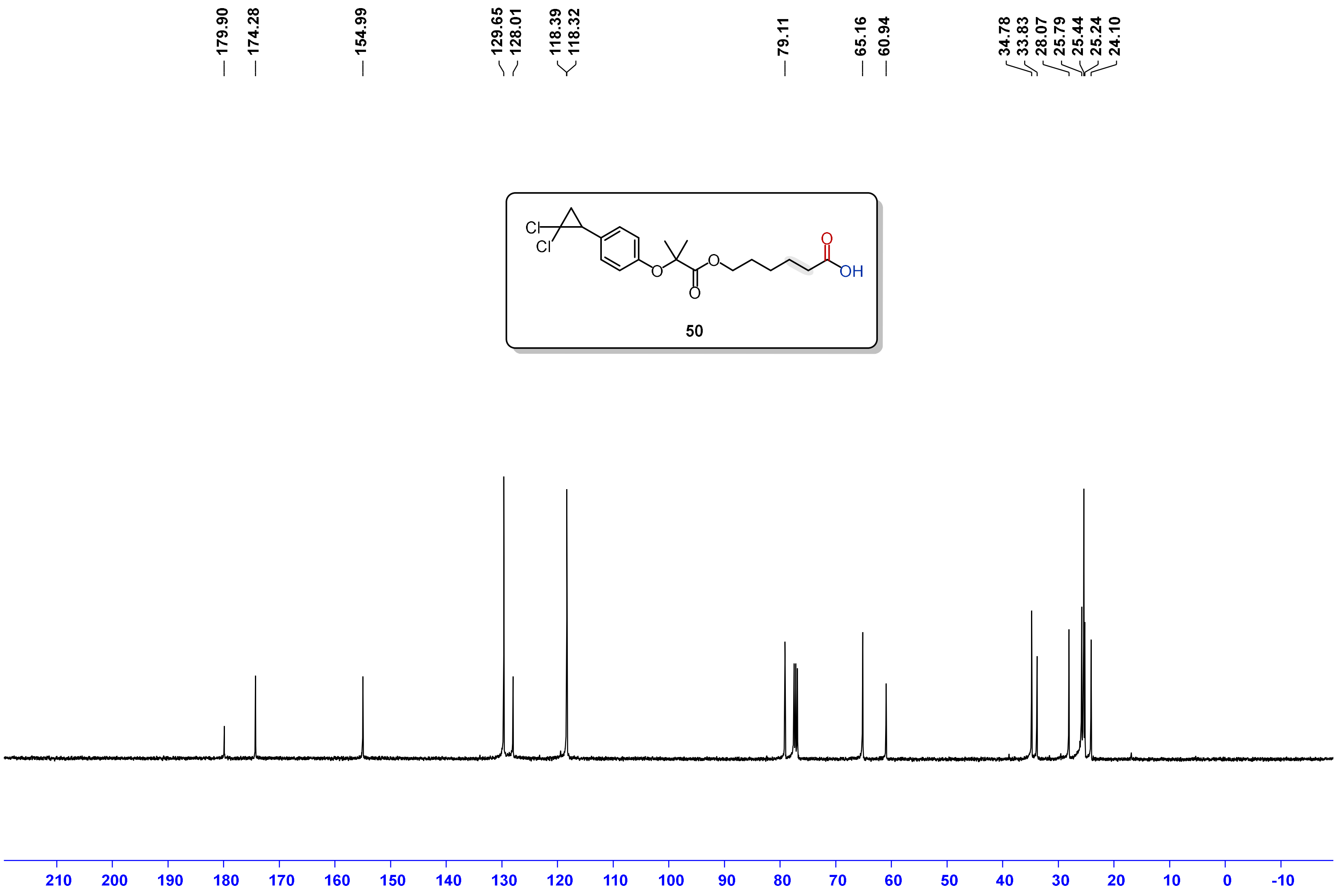

^{1}H NMR spectra for 51

lhc-x24z27-02.3.fid — 1H NMR (400 MHz, CDCl3)

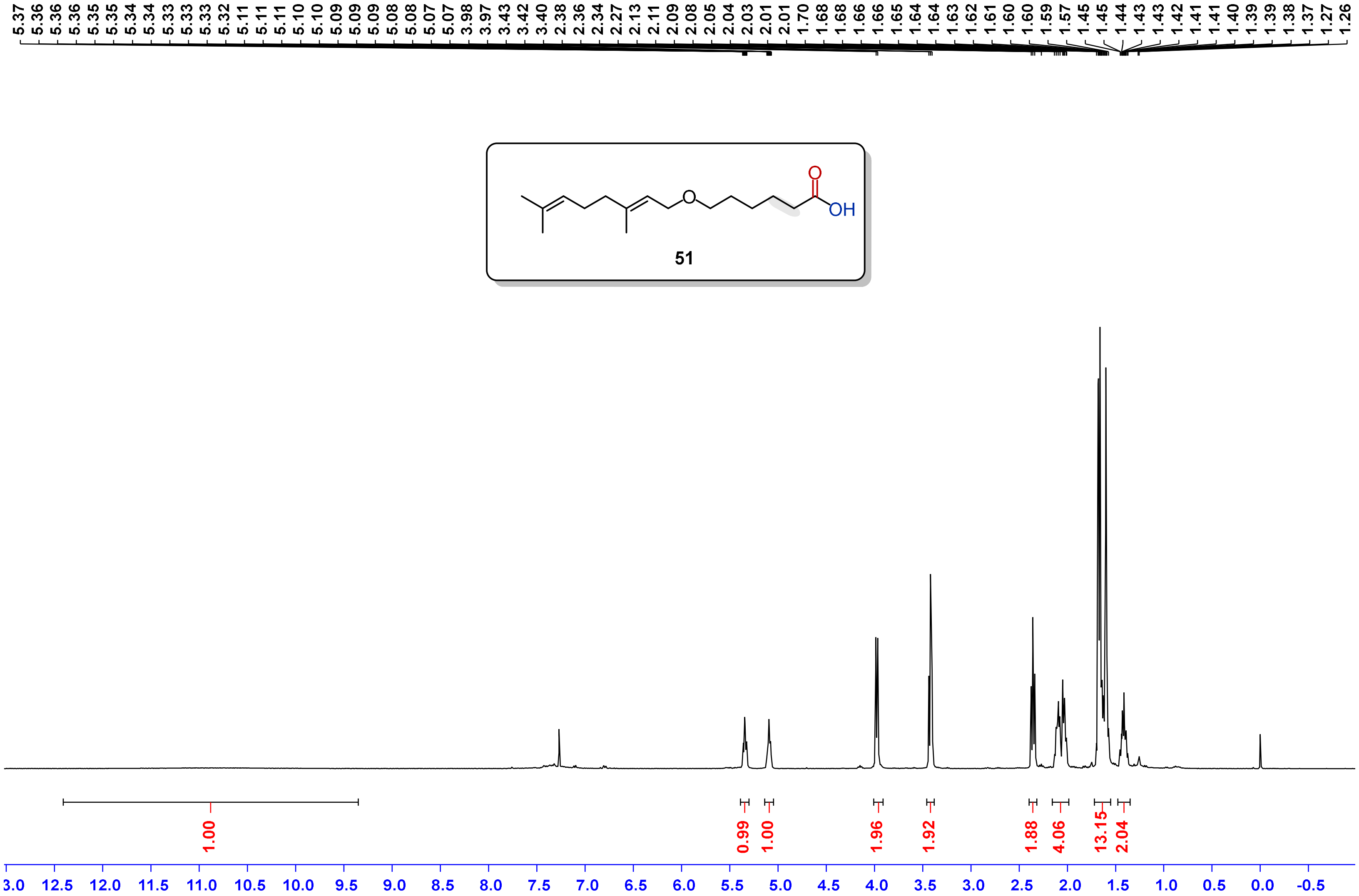

^{13}C NMR spectra for 51

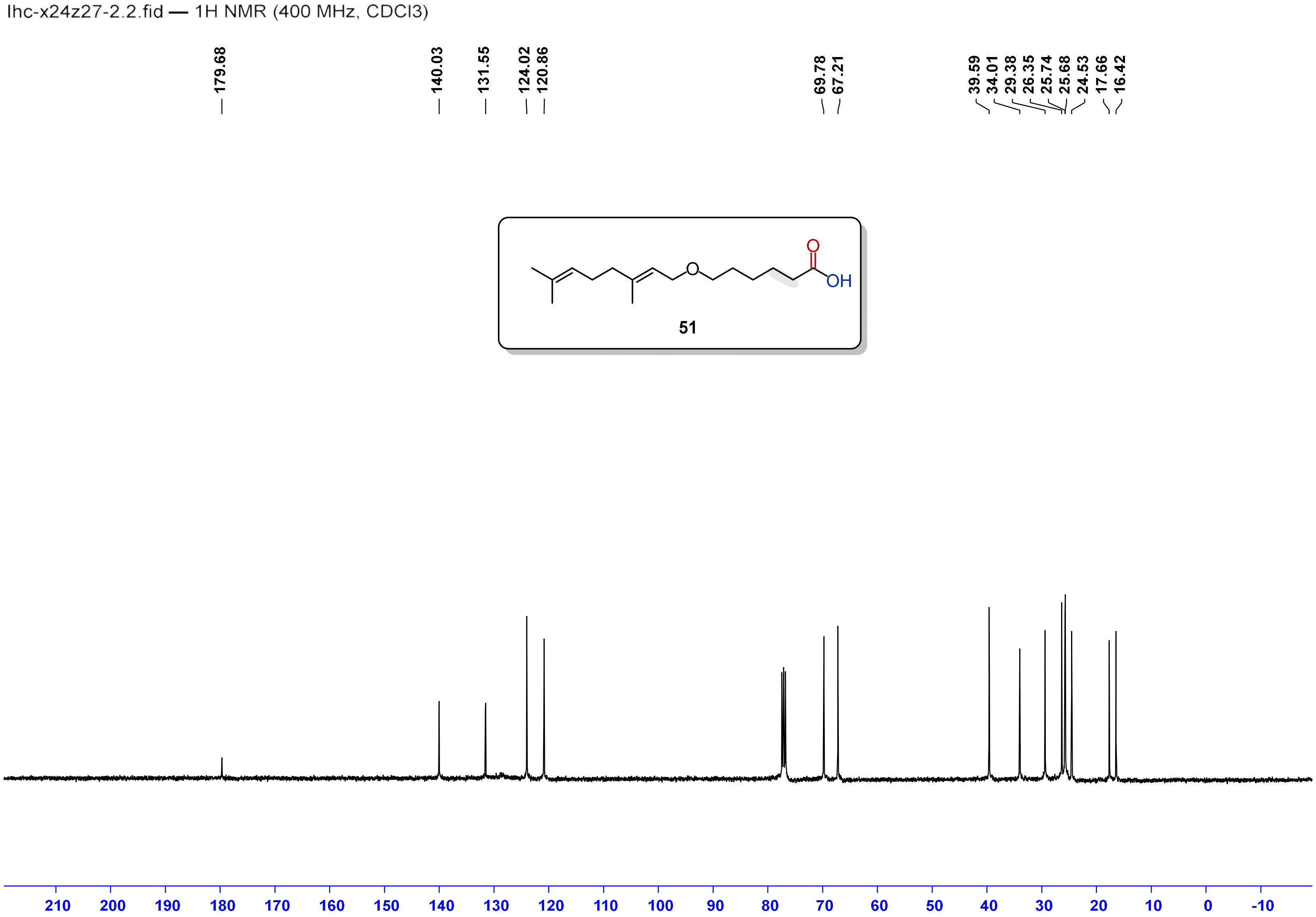

^{1}H NMR spectra for 52

lhc-x24z25-3.1.fid — 1H NMR (400 MHz, CDCl3)

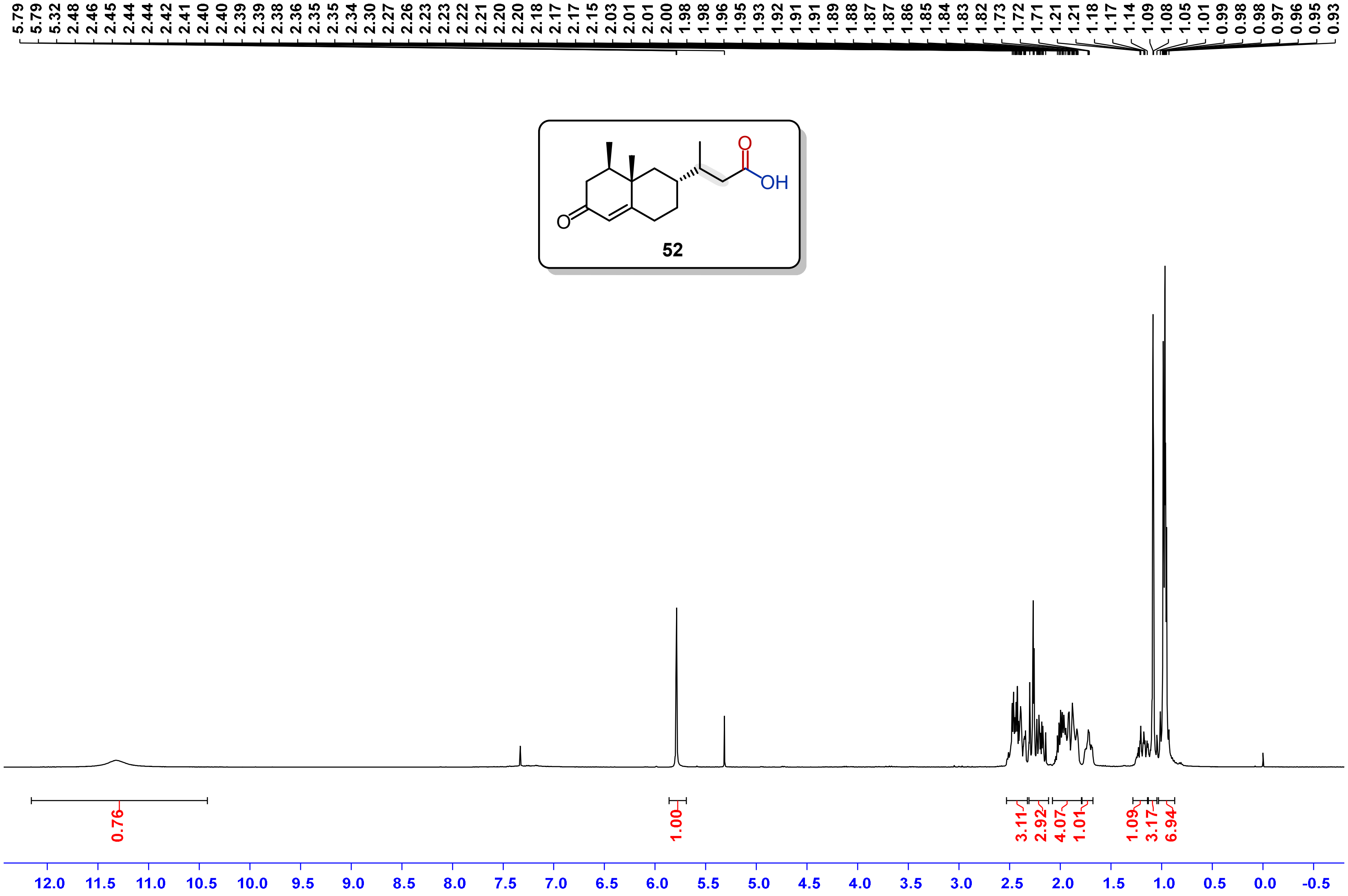

^{13}C NMR spectra for 52

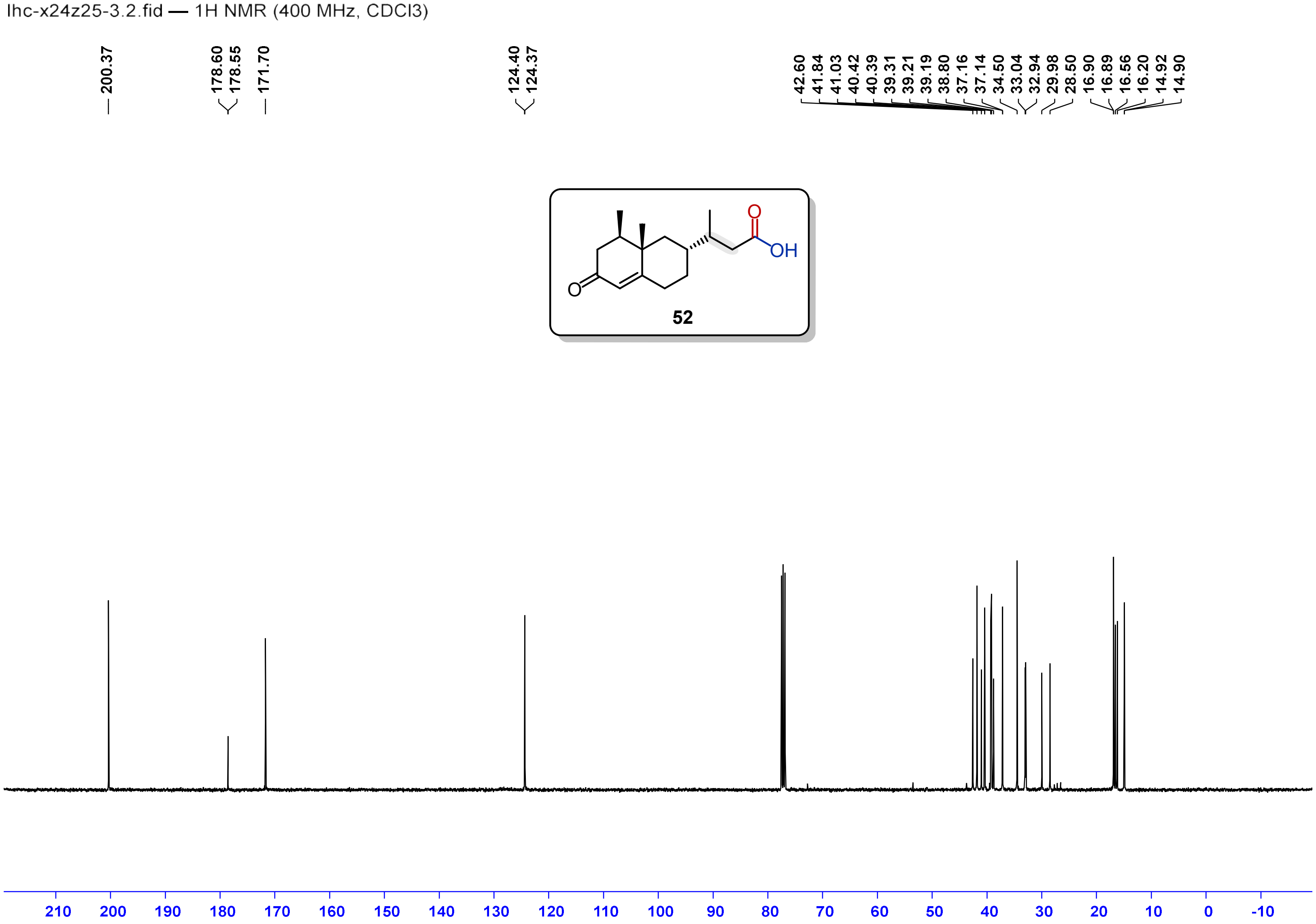

^{1}H NMR spectra for 53

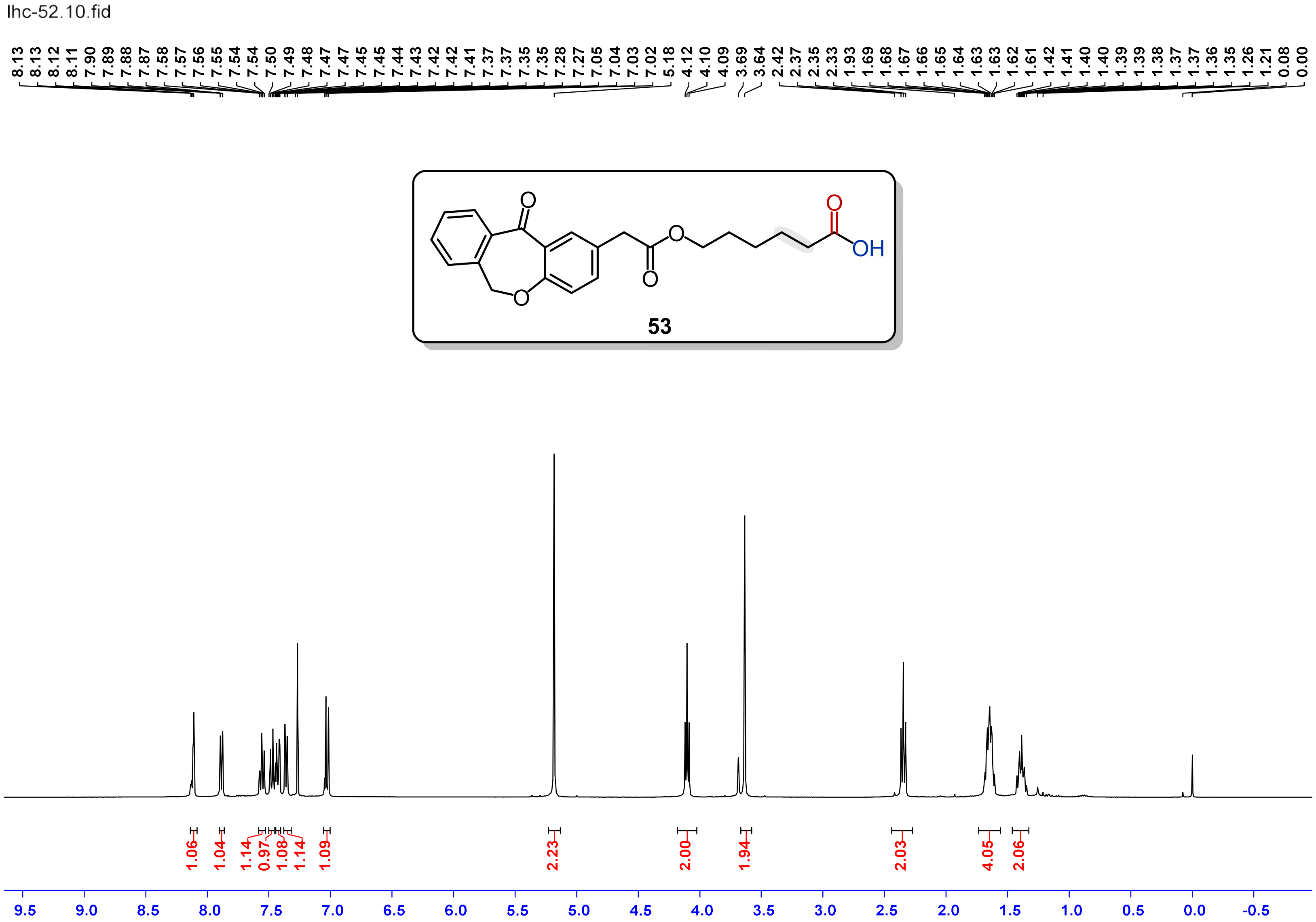

^{13}C NMR spectra for 53

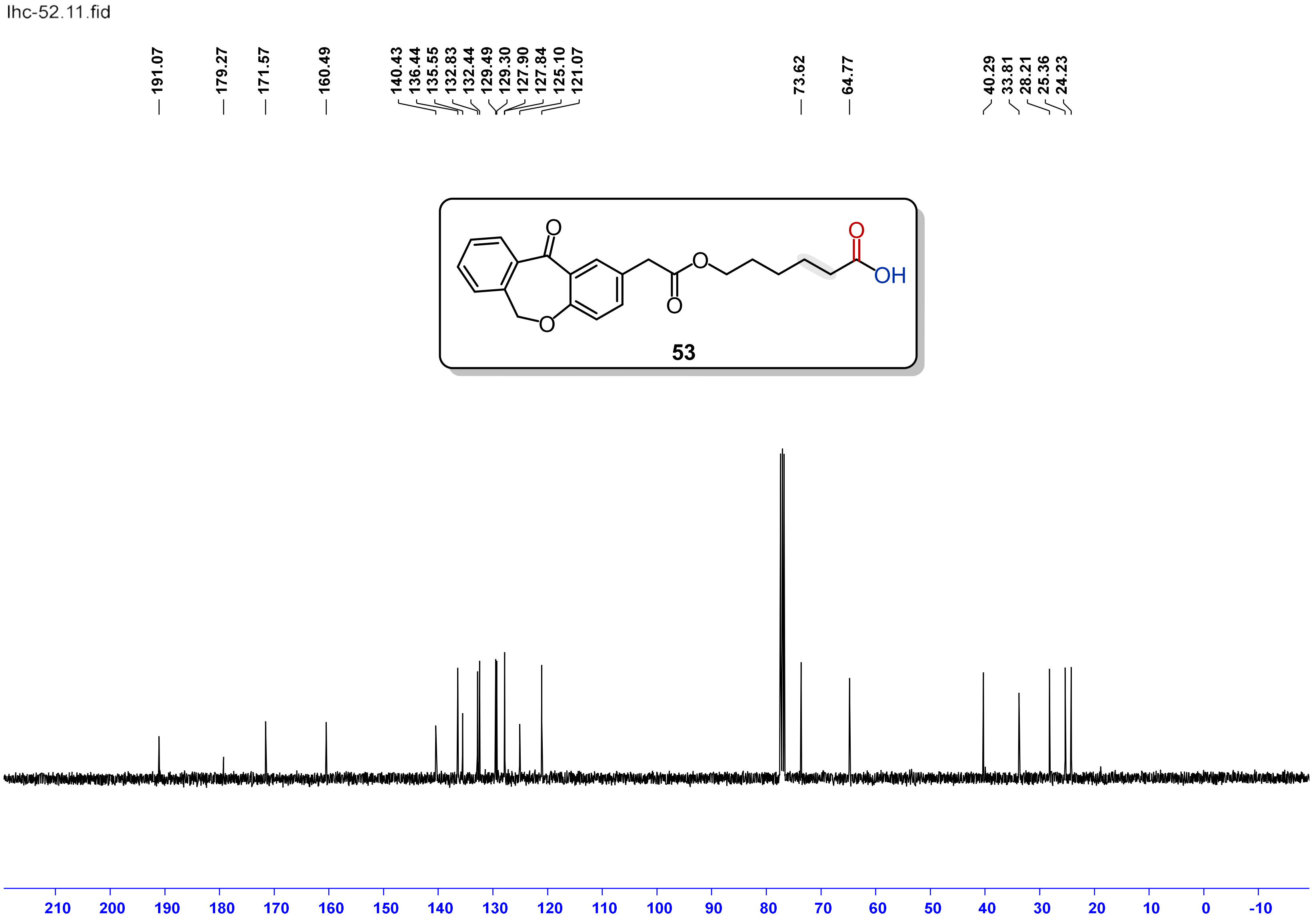

^{1}H NMR spectra for 54

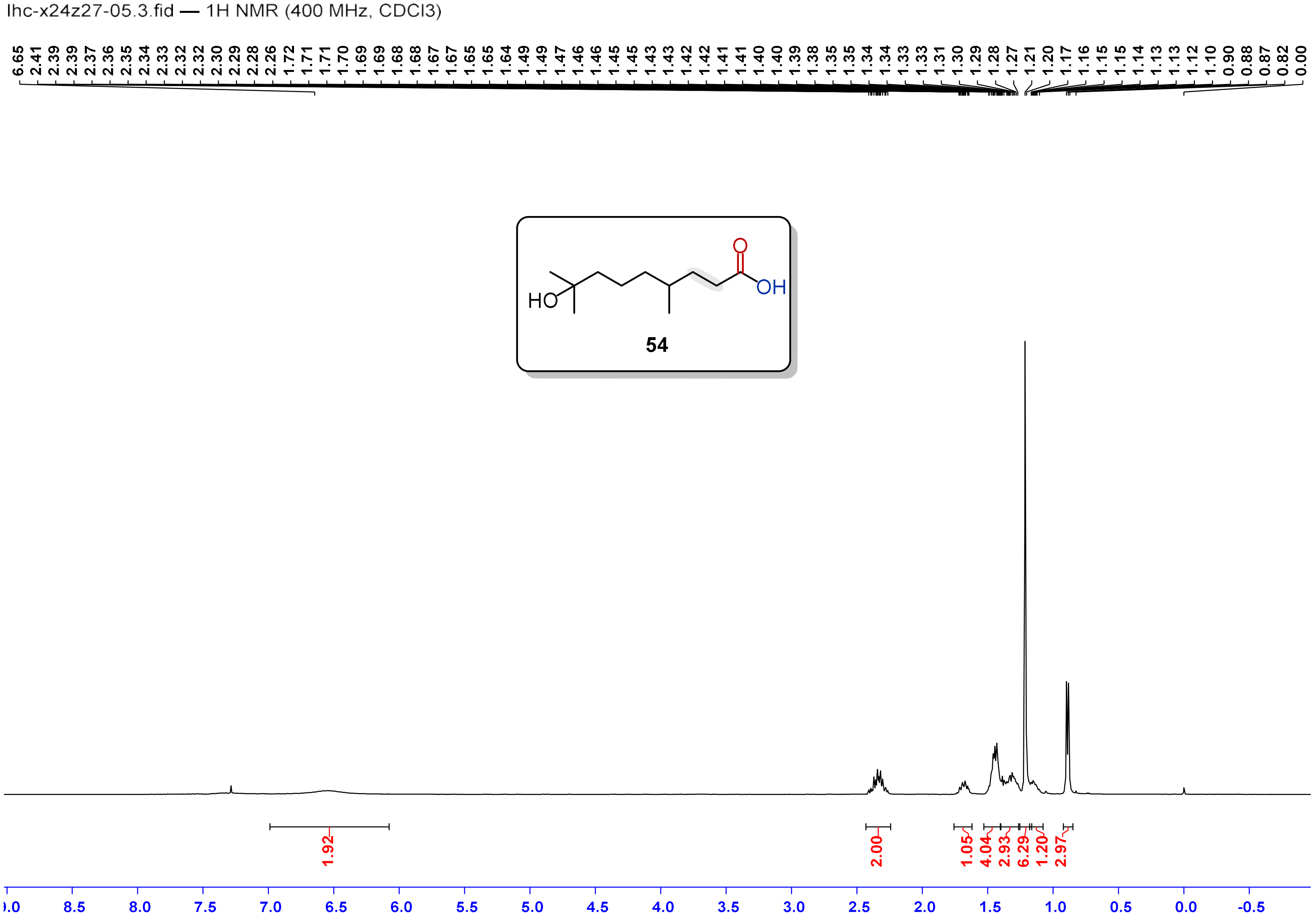

^{13}C NMR spectra for 54

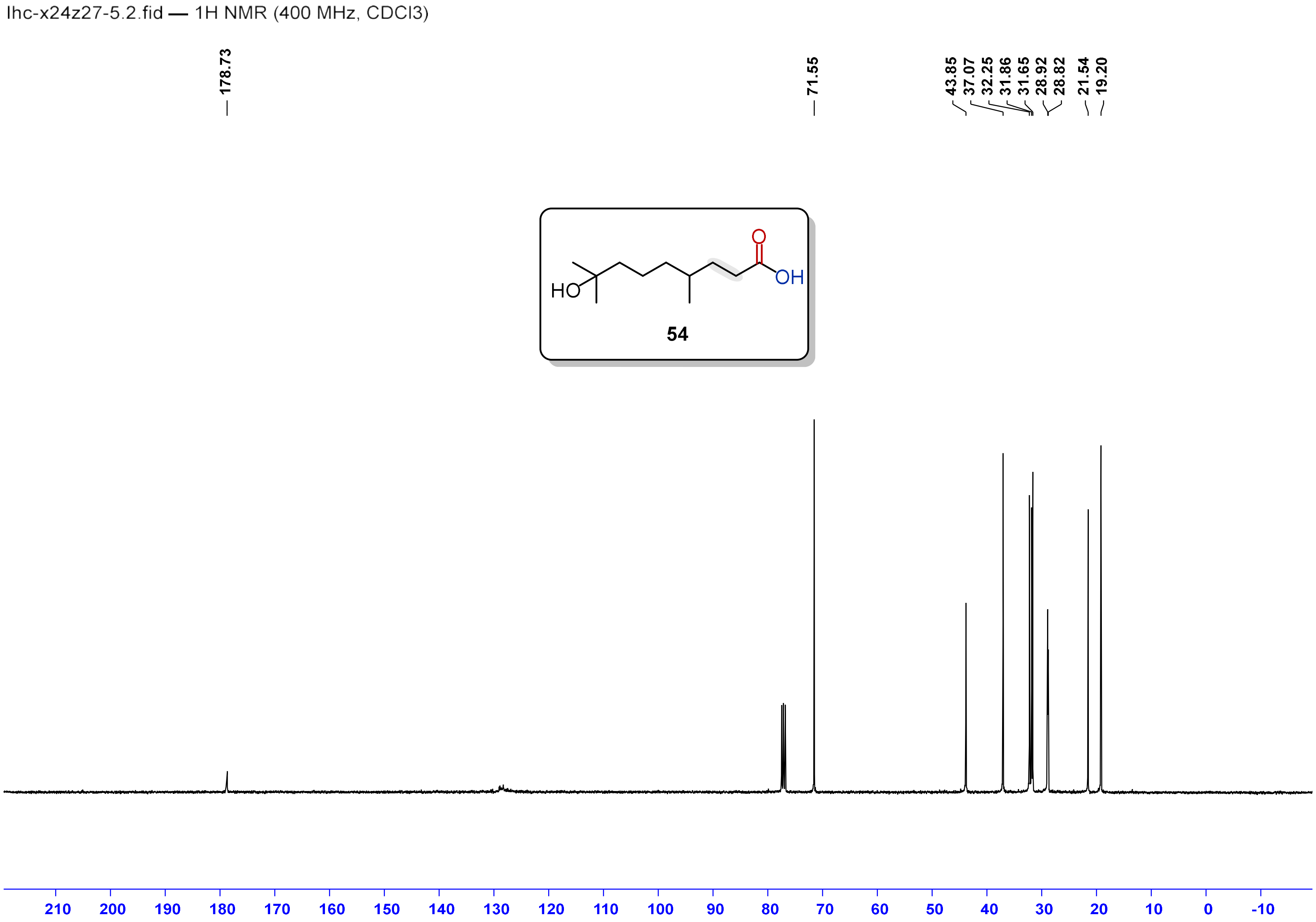

^{1}H NMR spectra for 55

lhc-x250524-2-1.1.fid — 1H NMR (400 MHz, CDCl3)

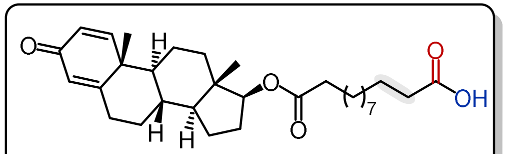

55

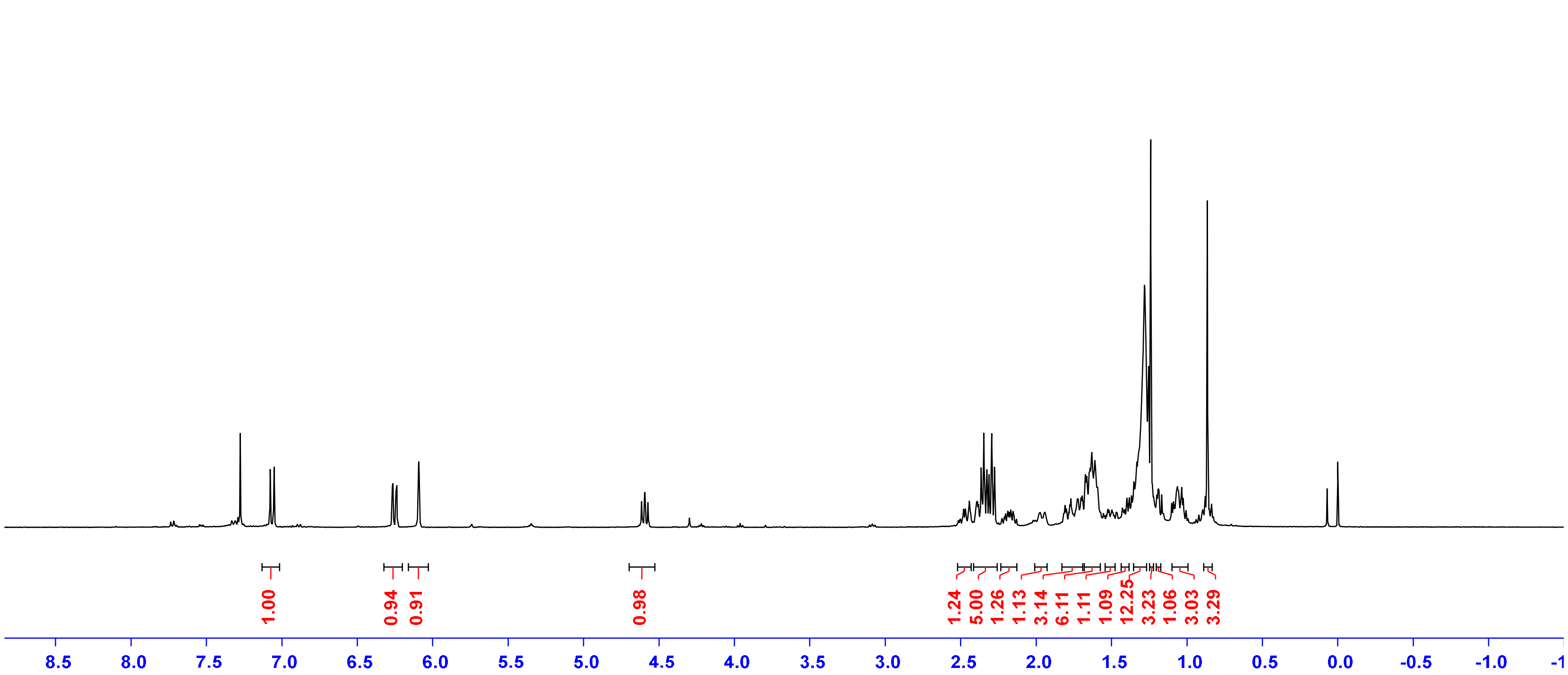

^{13}C NMR spectra for 55

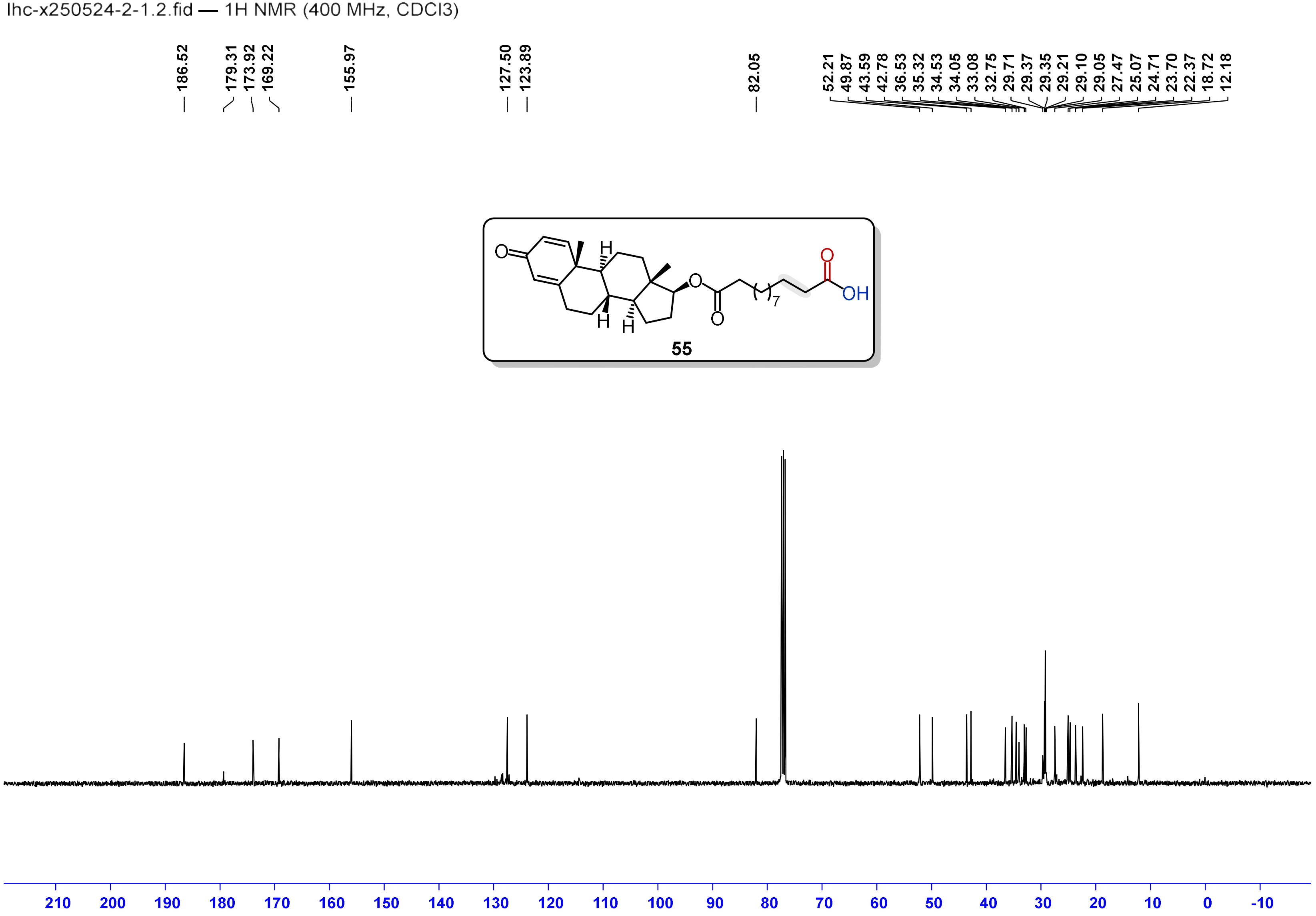

^{1}H NMR spectra for 56

lhc-x24z27-04.3.fid — 1H NMR (400 MHz, CDCl3)

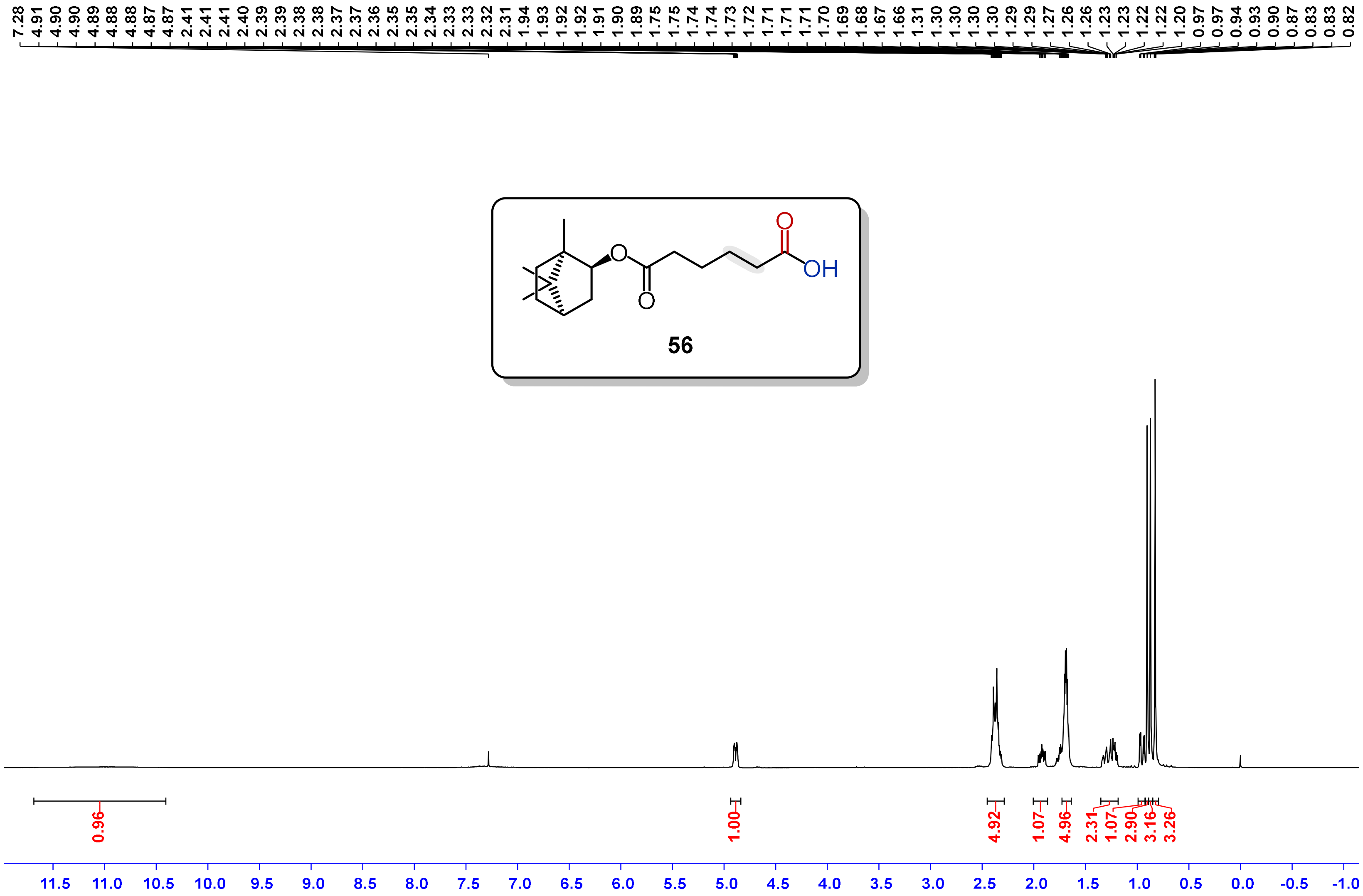

^{13}C NMR spectra for 56

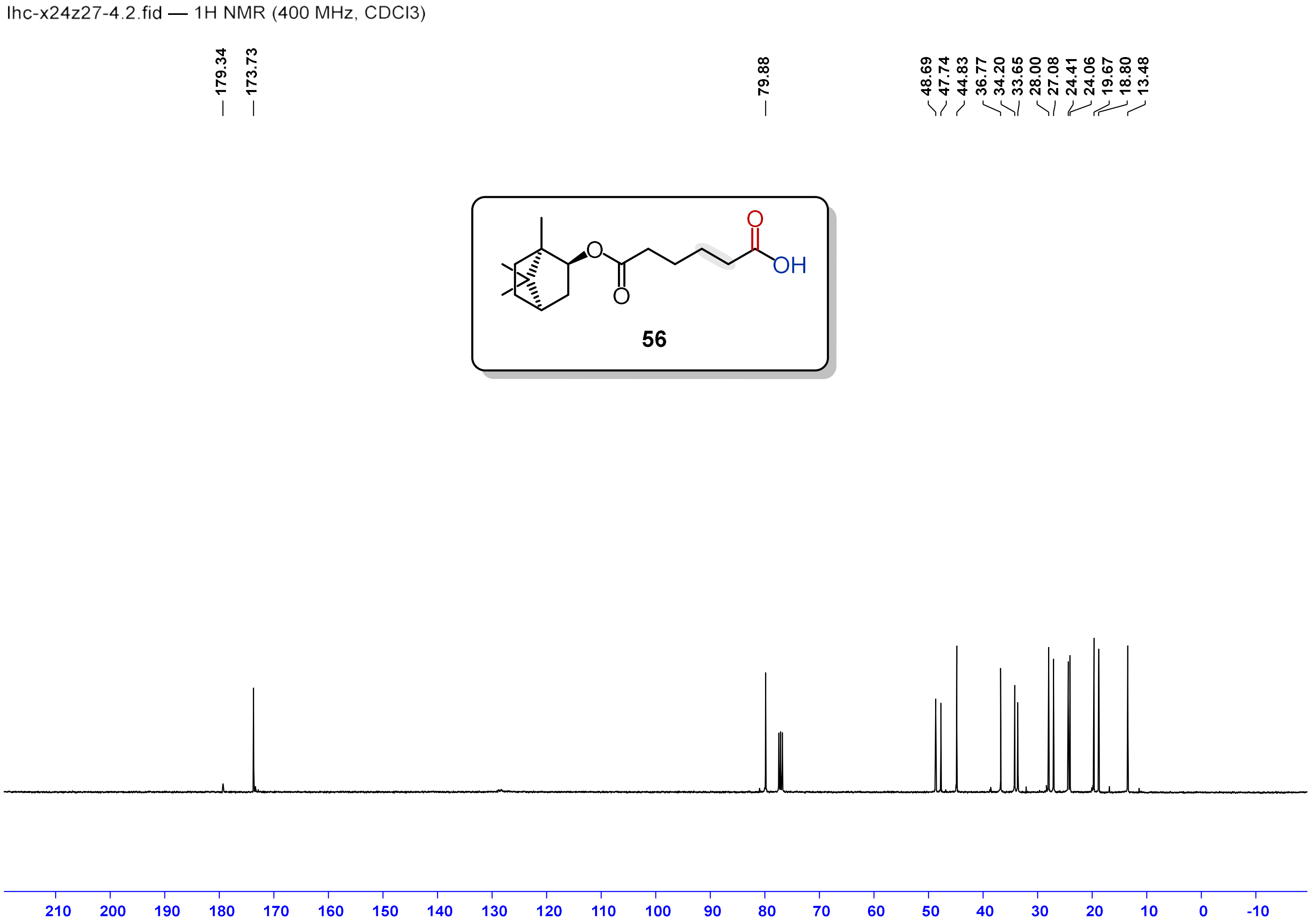

^{1}H NMR spectra for 57

lhc-x24z25-9.1.fid — 1H NMR (400 MHz, CDCl3)

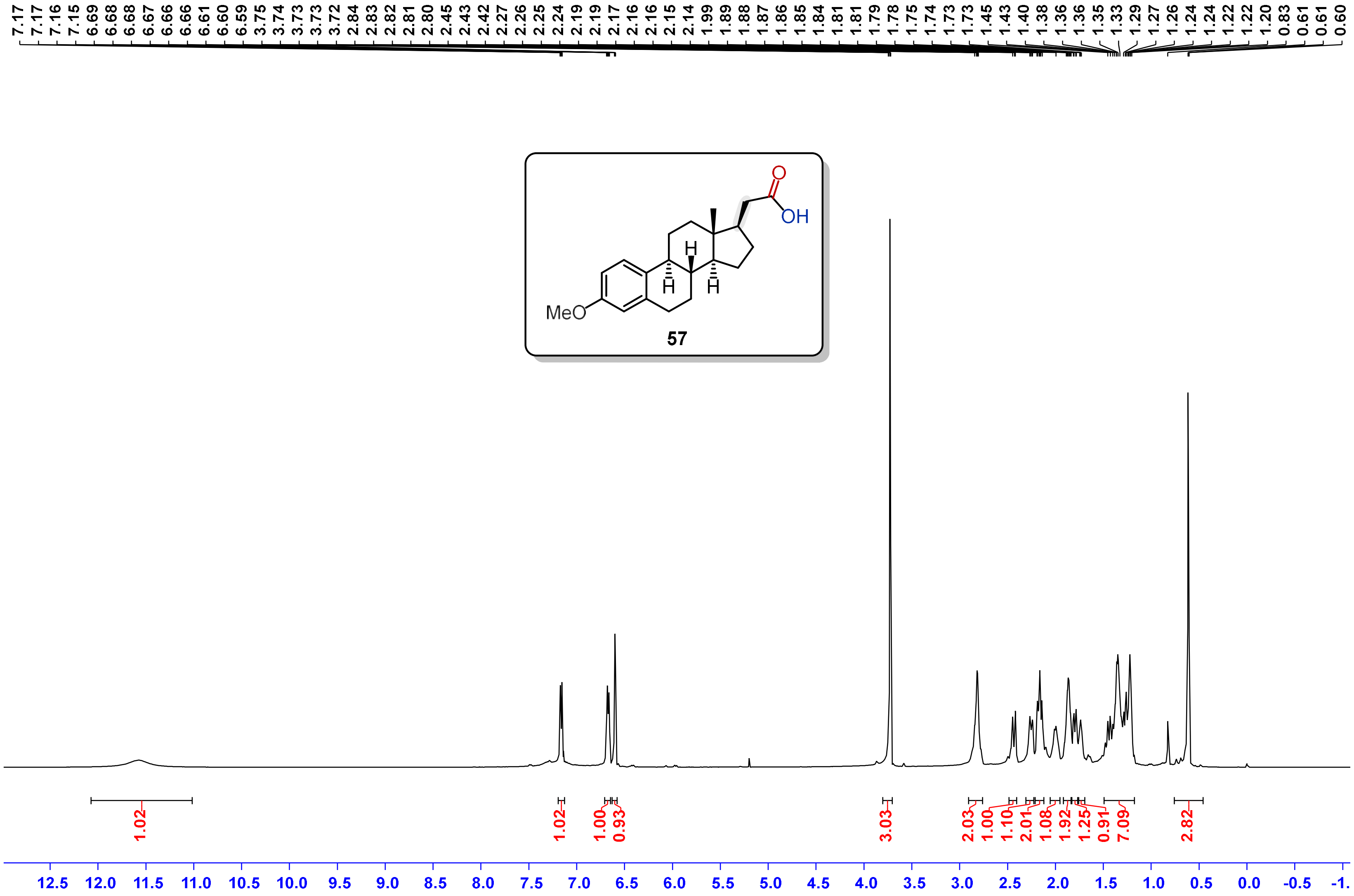

^{13}C NMR spectra for 57

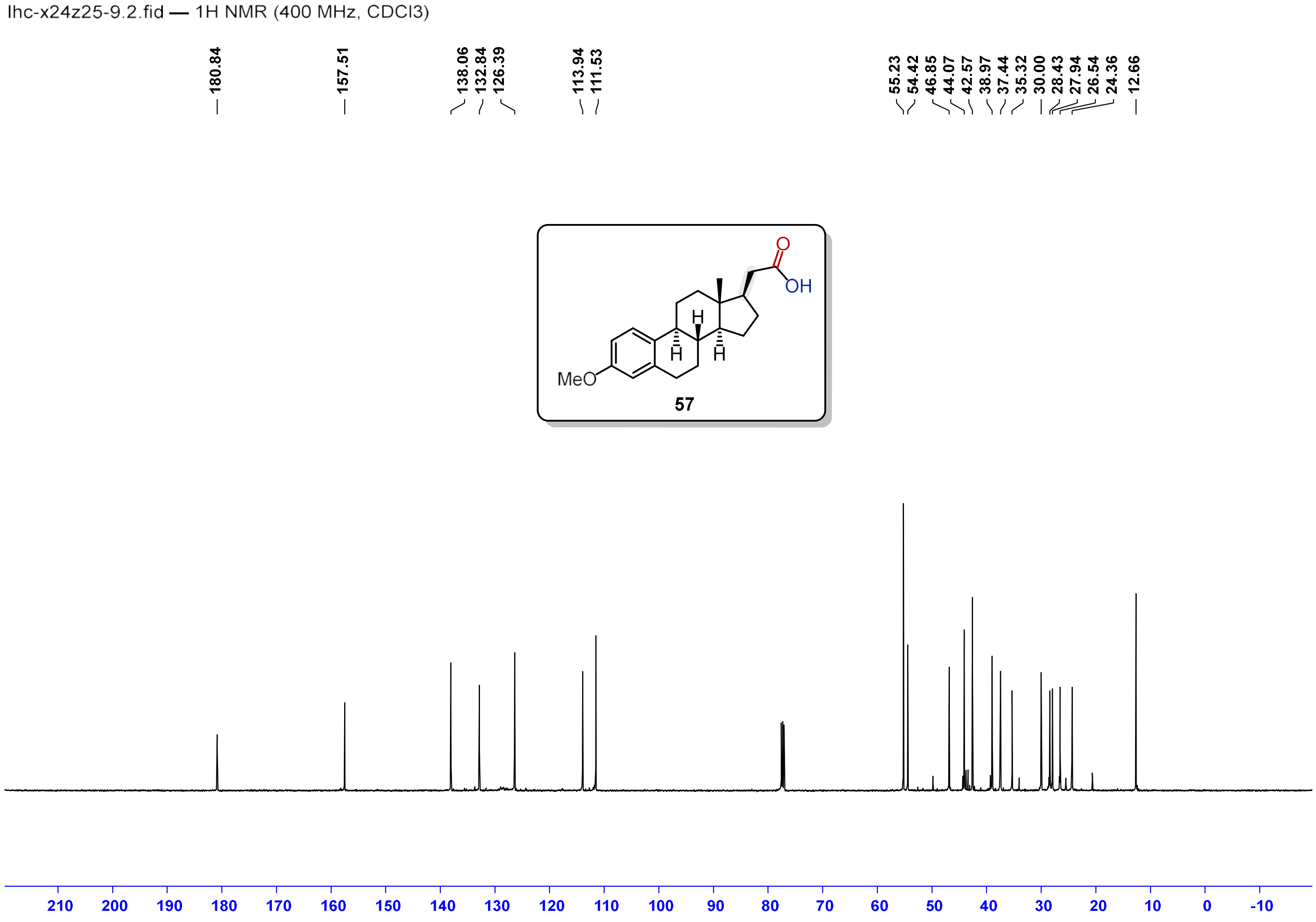

^{1}H NMR spectra for 58

lhc-x250518-1.1.fid — 1H NMR (400 MHz, CDCl3)

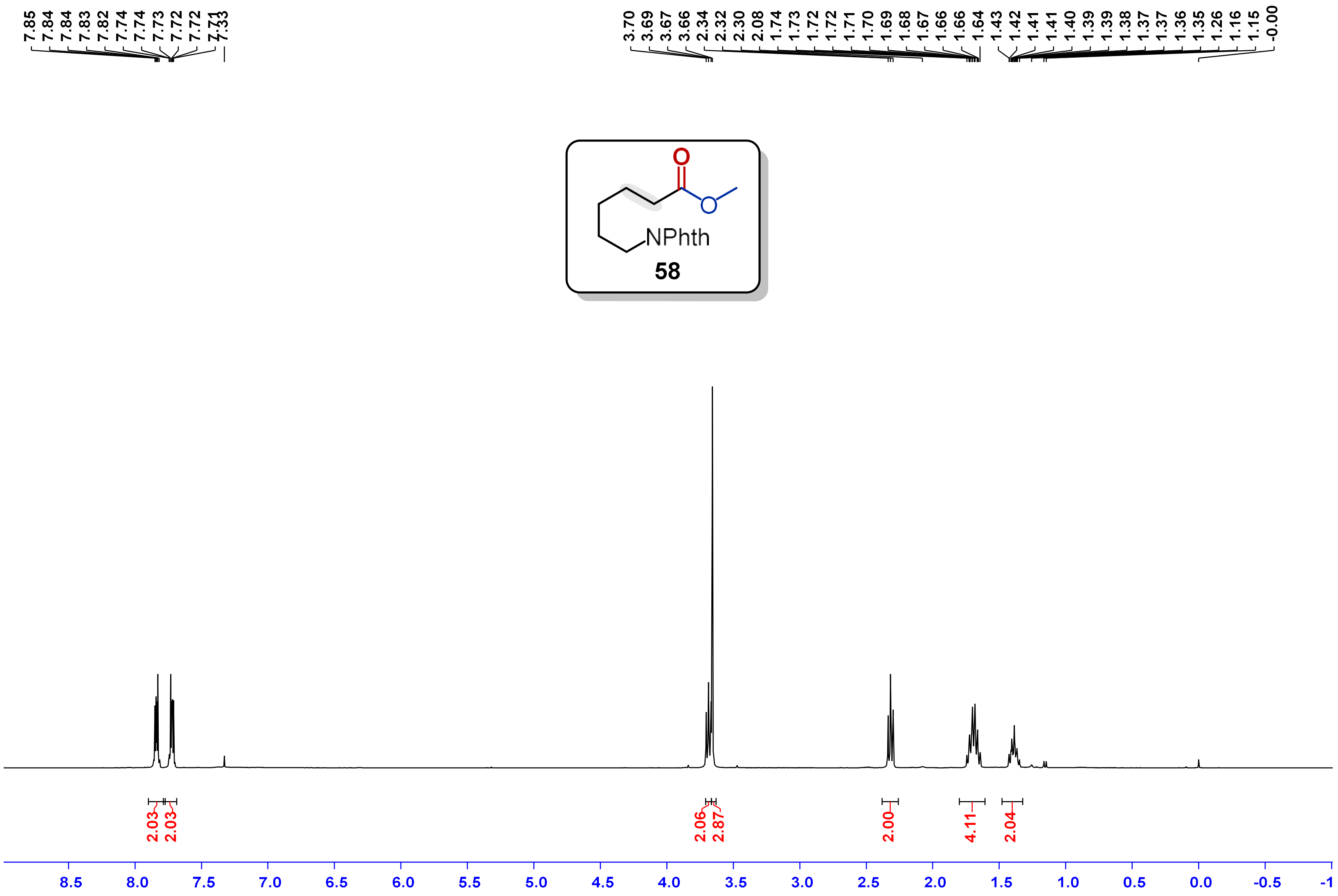

^{13}C NMR spectra for 58

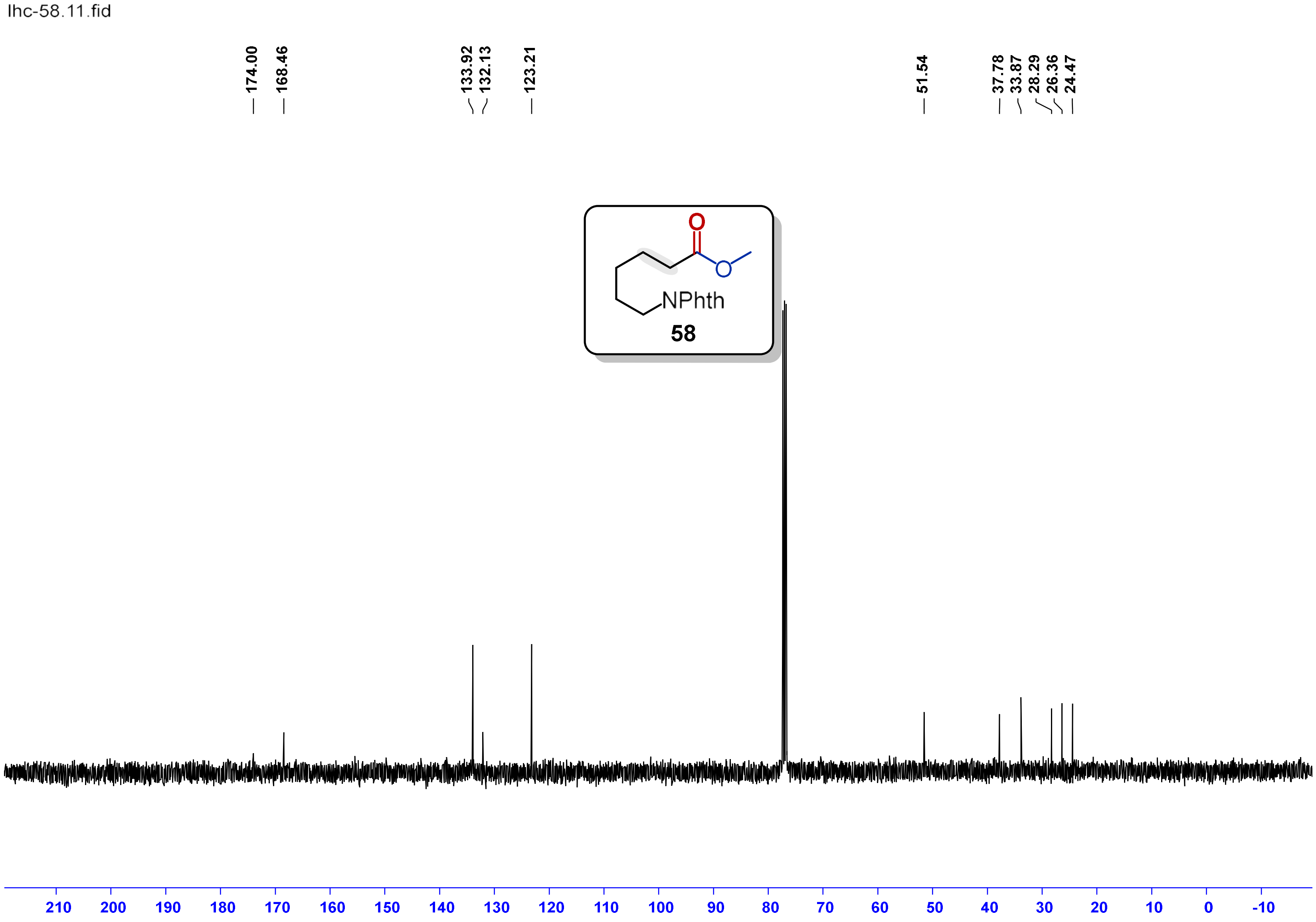

^{1}H NMR spectra for 59

lhc-x250518-2.1.fid — 1H NMR (400 MHz, CDCl3)

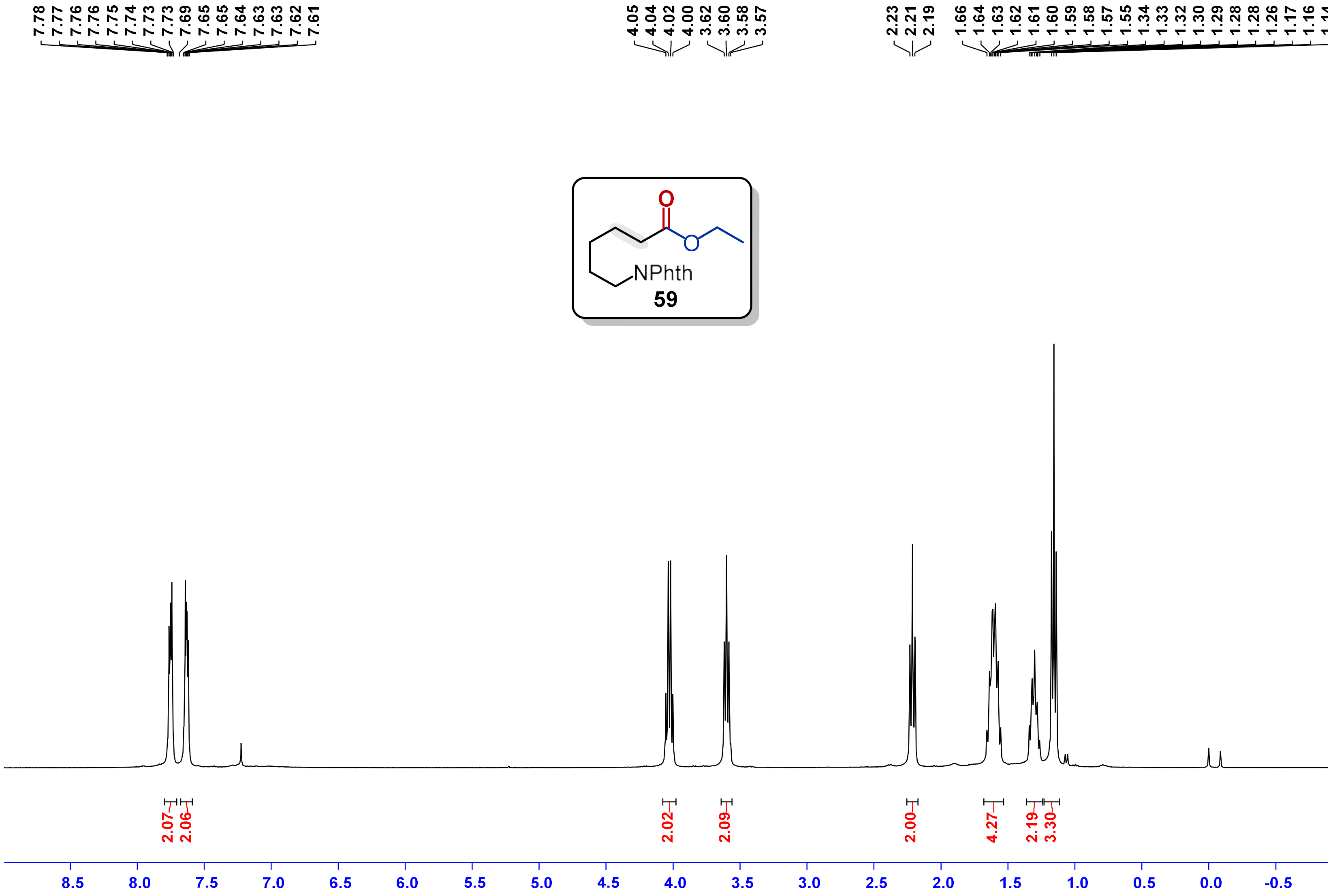

^{13}C NMR spectra for 59

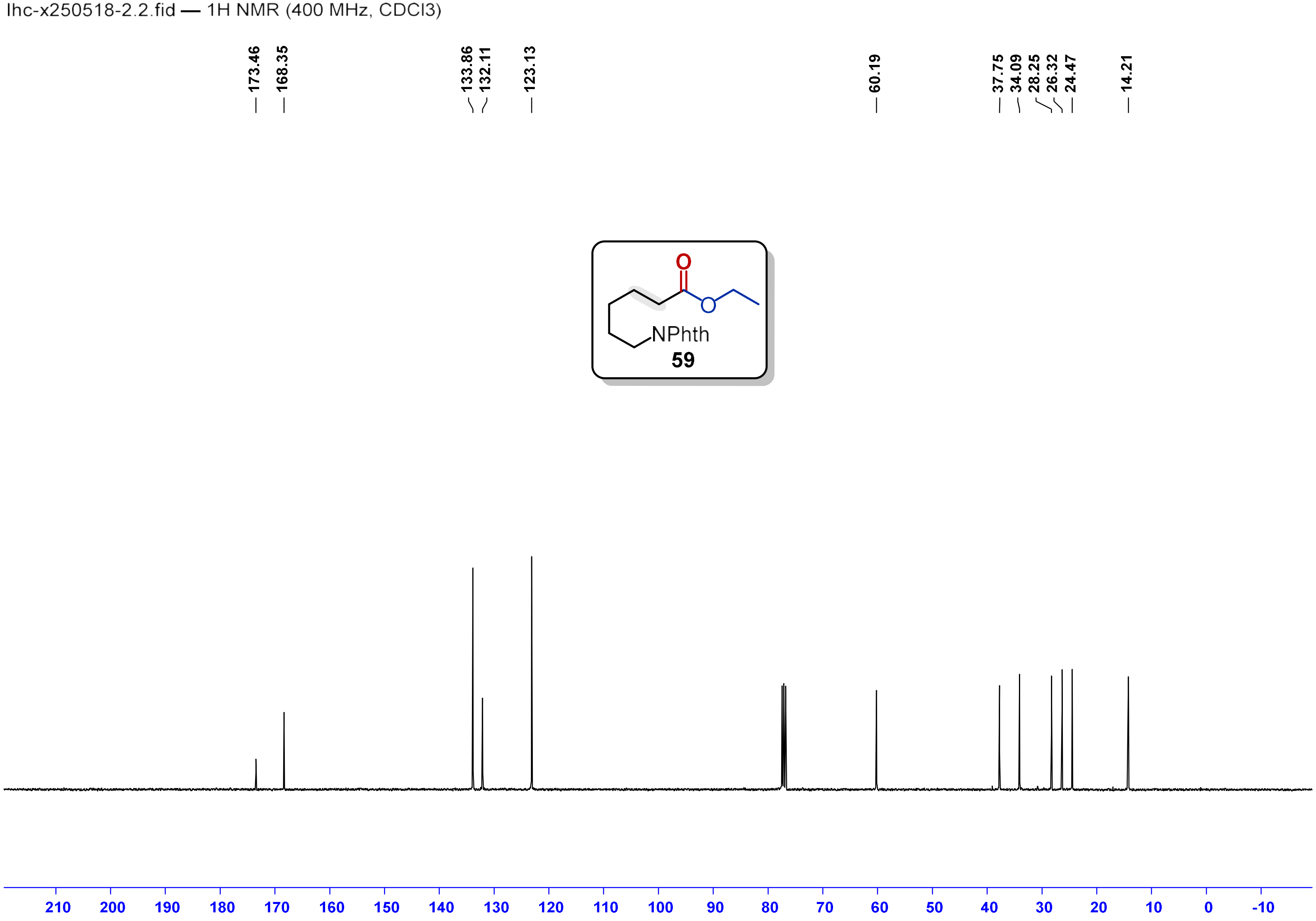

^{1}H NMR spectra for 60

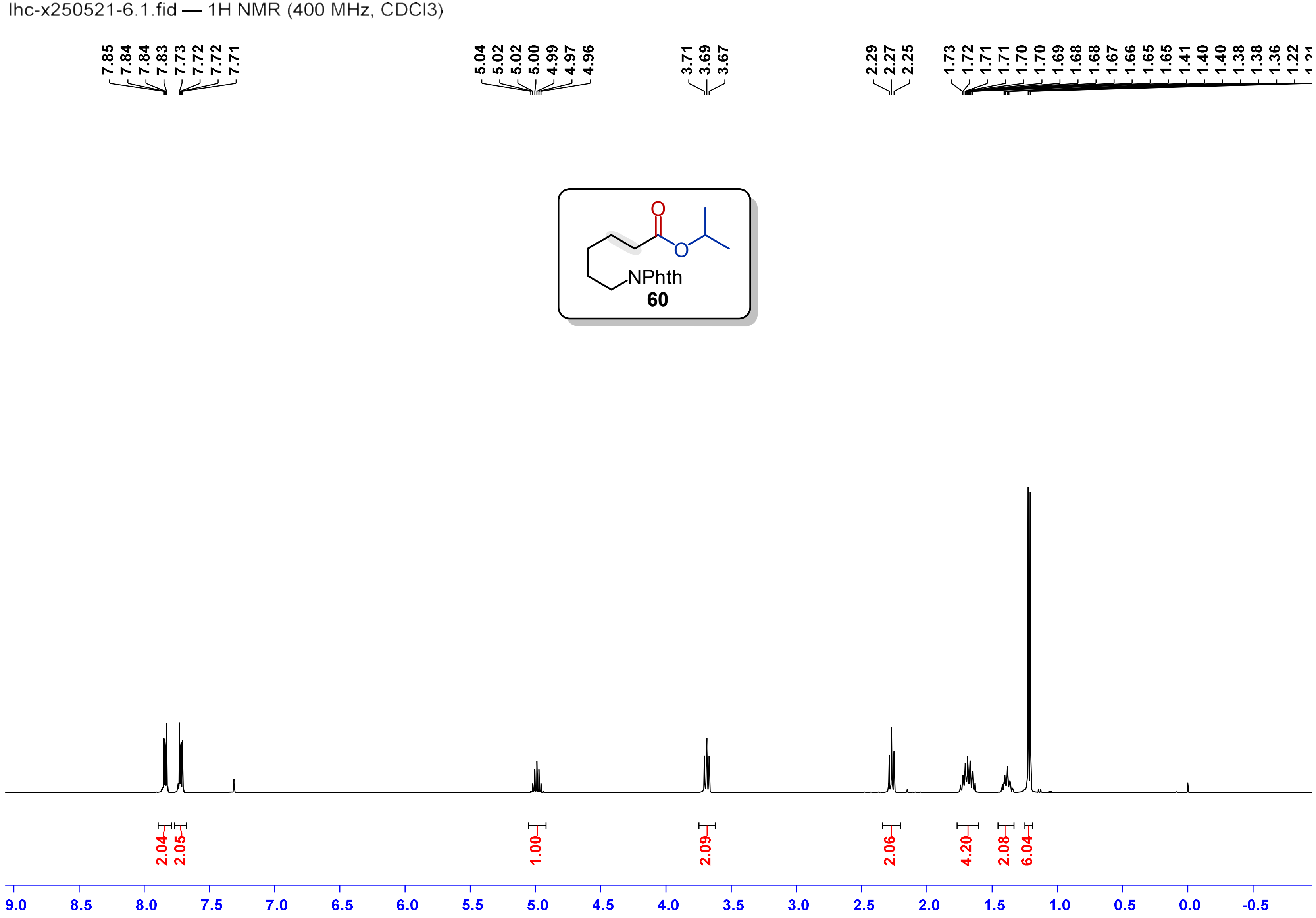

^{13}C NMR spectra for 60

lhc-x250521-6.2.fid — 1H NMR (400 MHz, CDCl3)

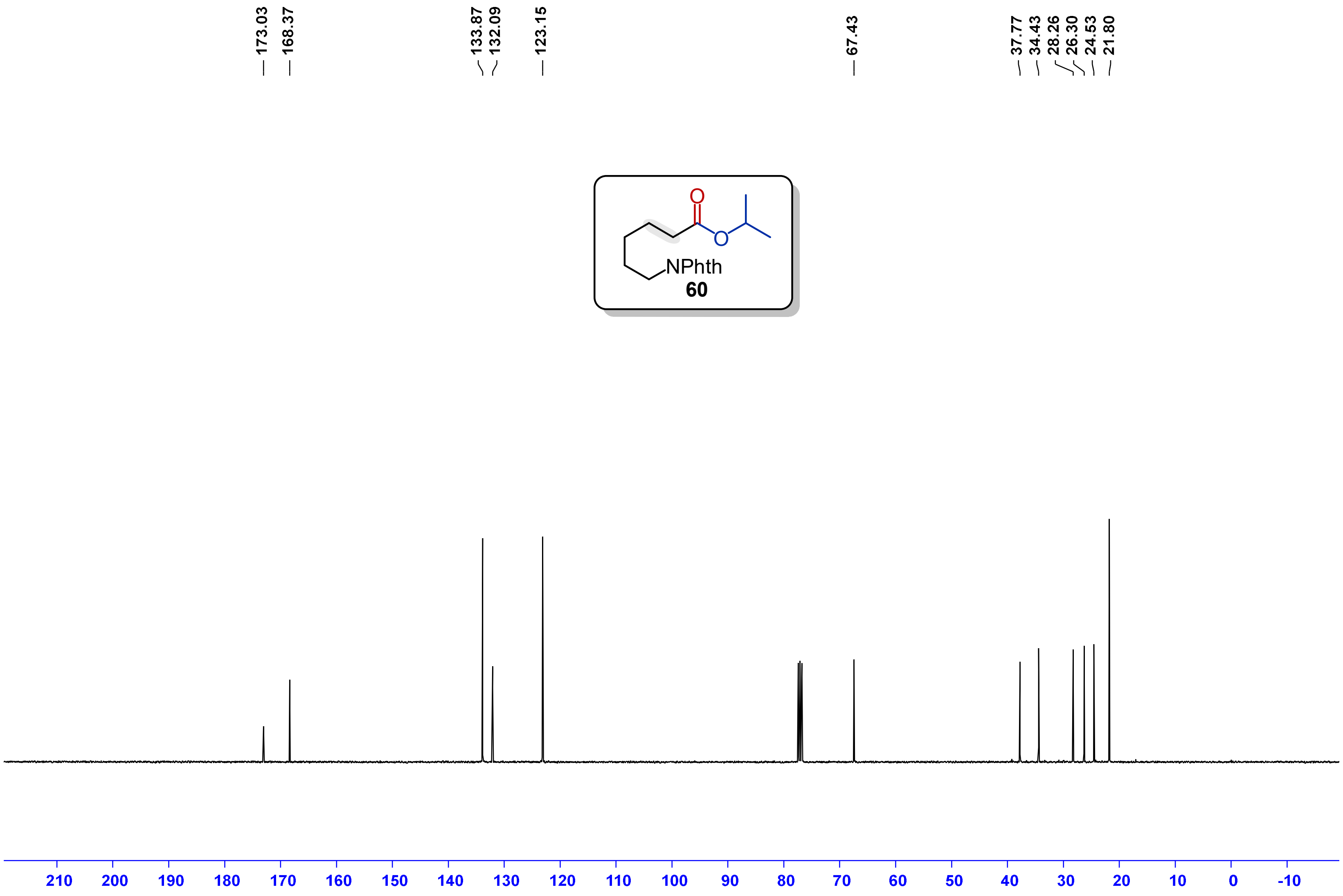

^{1}H NMR spectra for 61

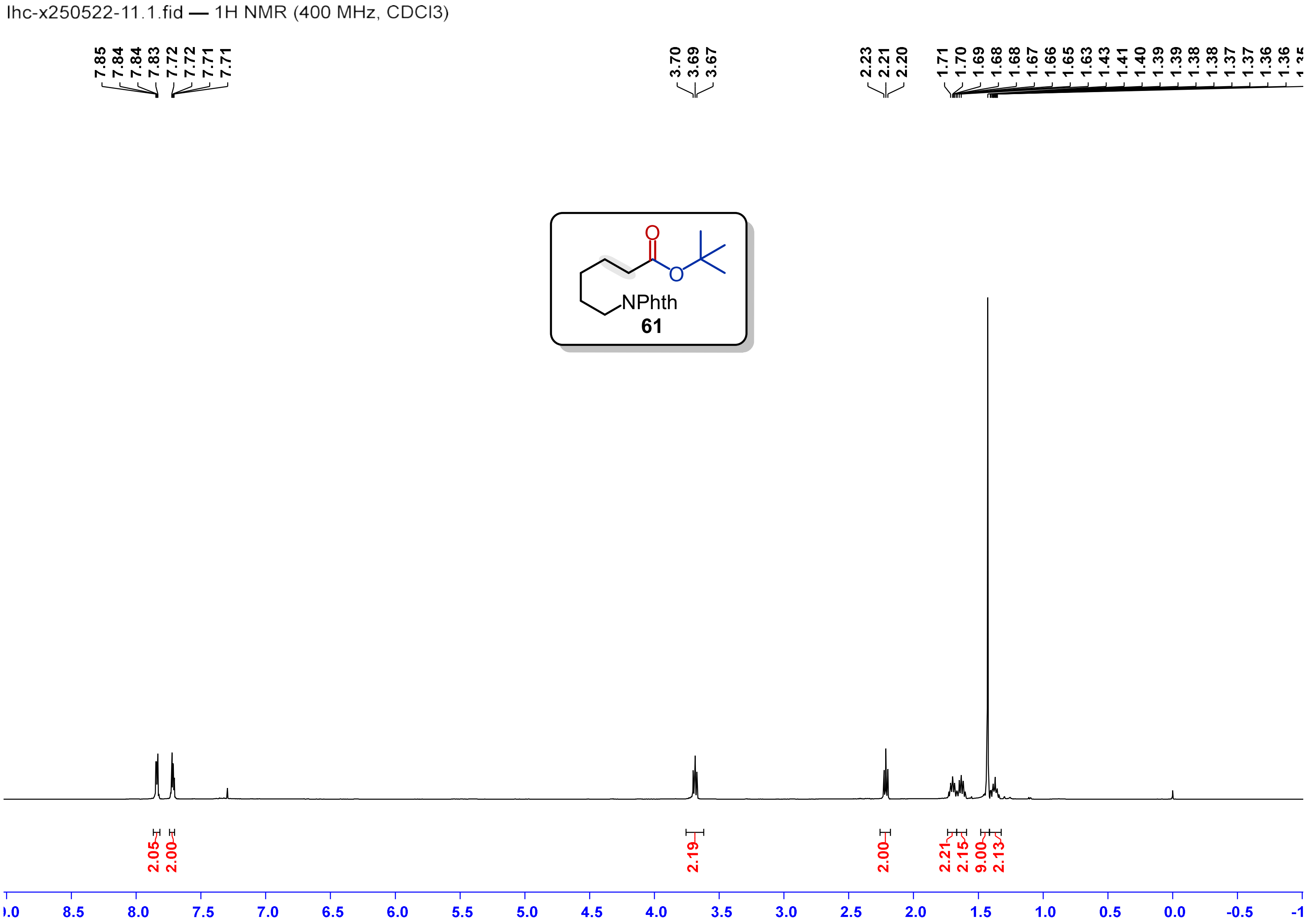

^{13}C NMR spectra for 61

lhc-x250522-11.2.fid — 1H NMR (400 MHz, CDCl3)

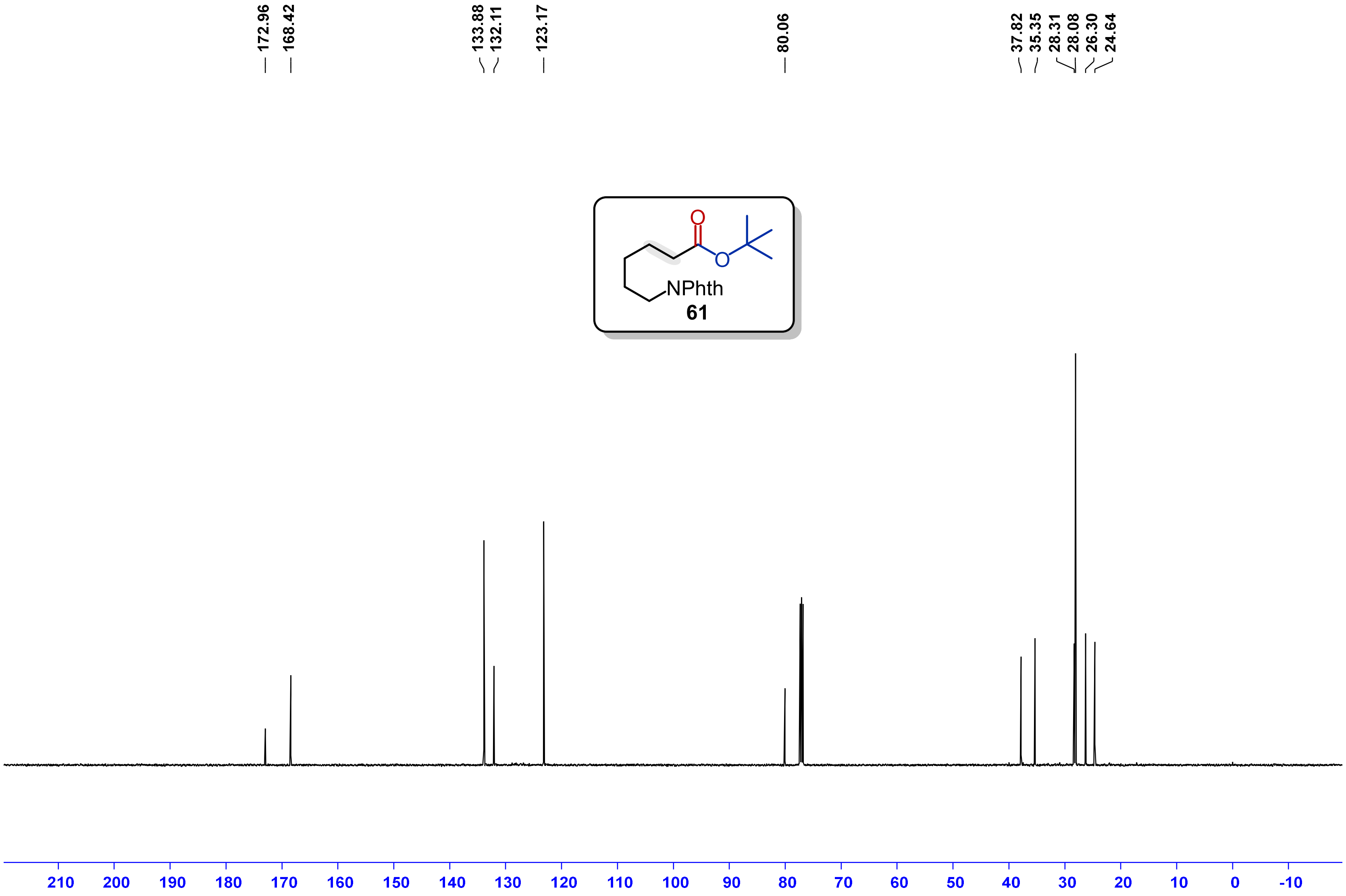

^{1}H NMR spectra for 62

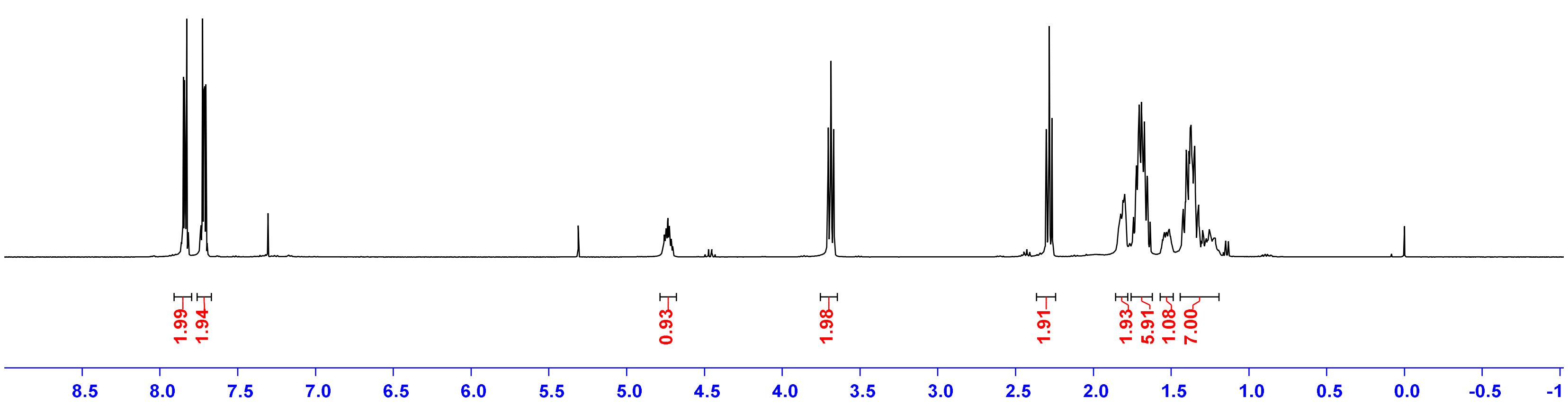

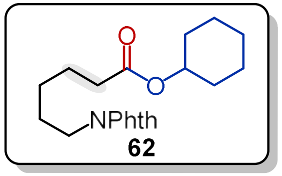

^{13}C NMR spectra for 62

lhc-x250520-7.2.fid — 1H NMR (400 MHz, CDCl3)

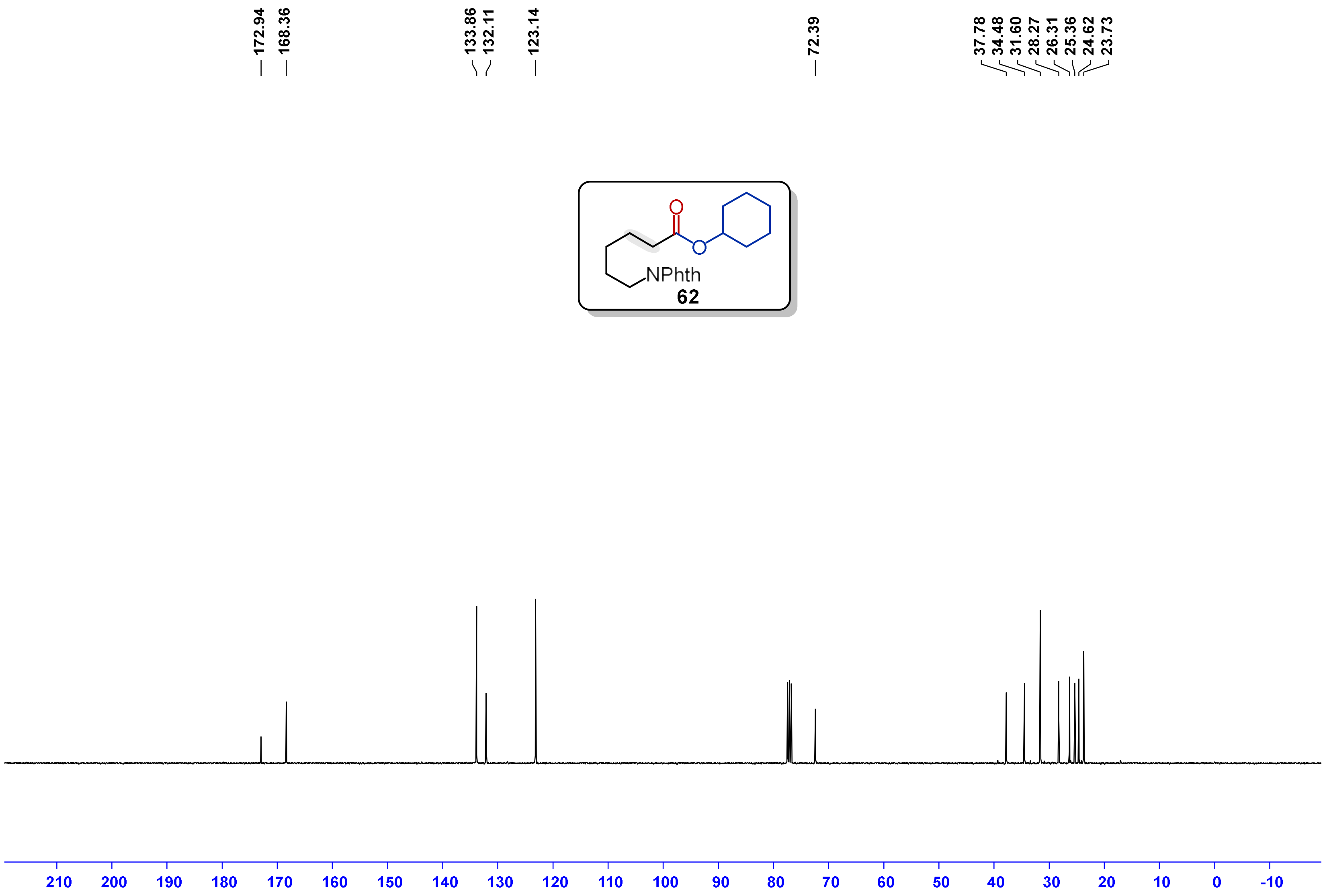

^{1}H NMR spectra for 63

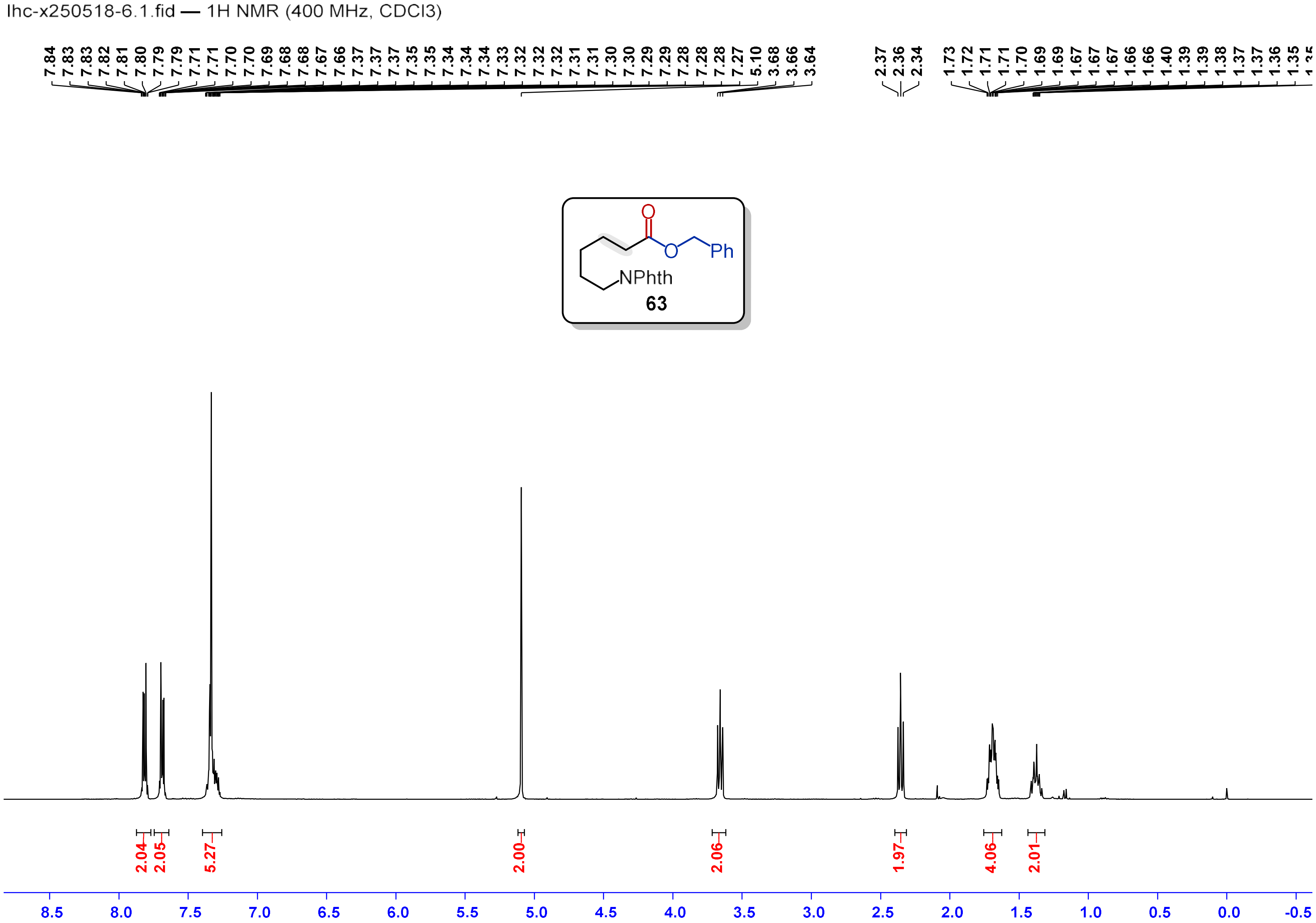

^{13}C NMR spectra for 63

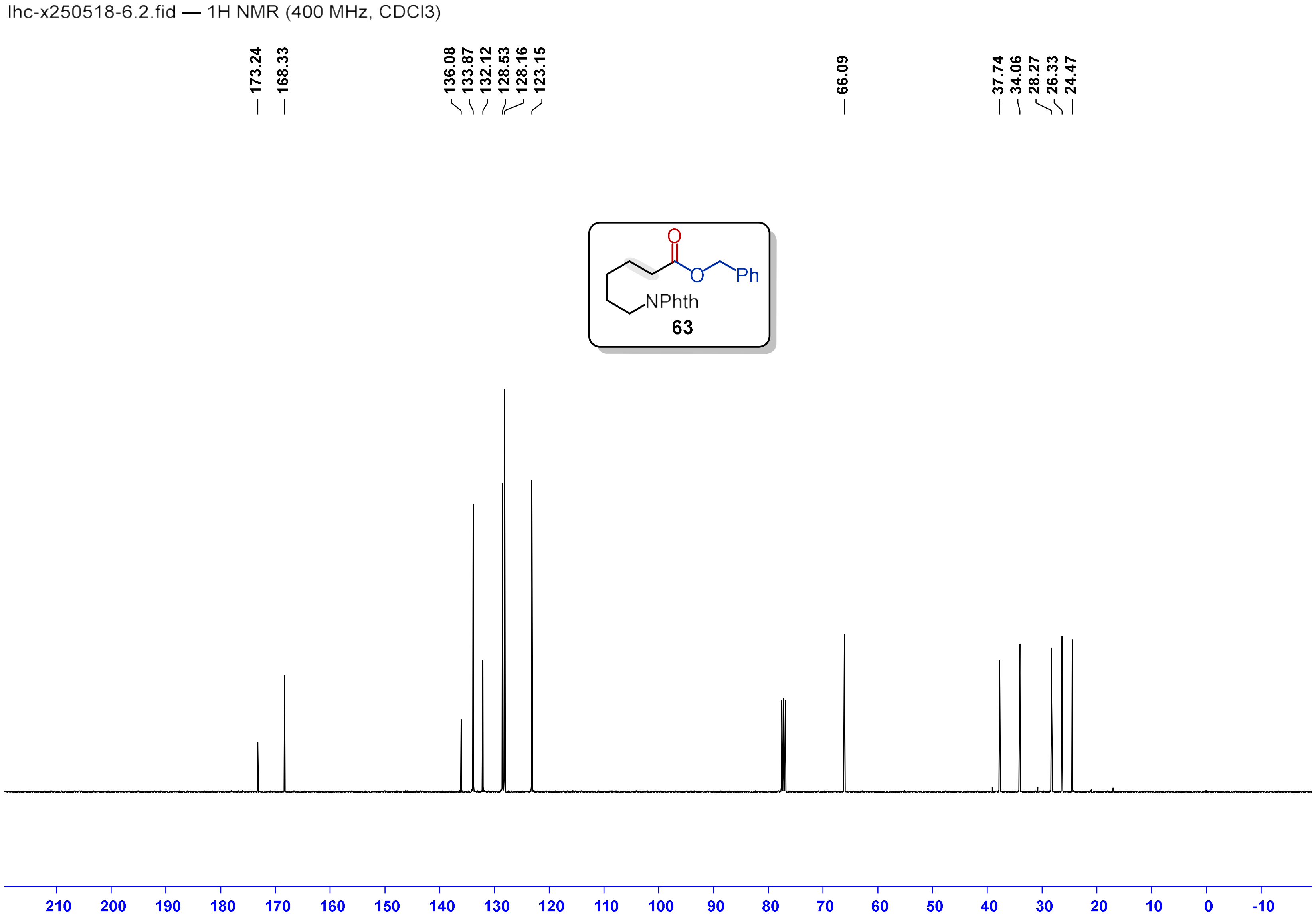

^{1}H NMR spectra for 64

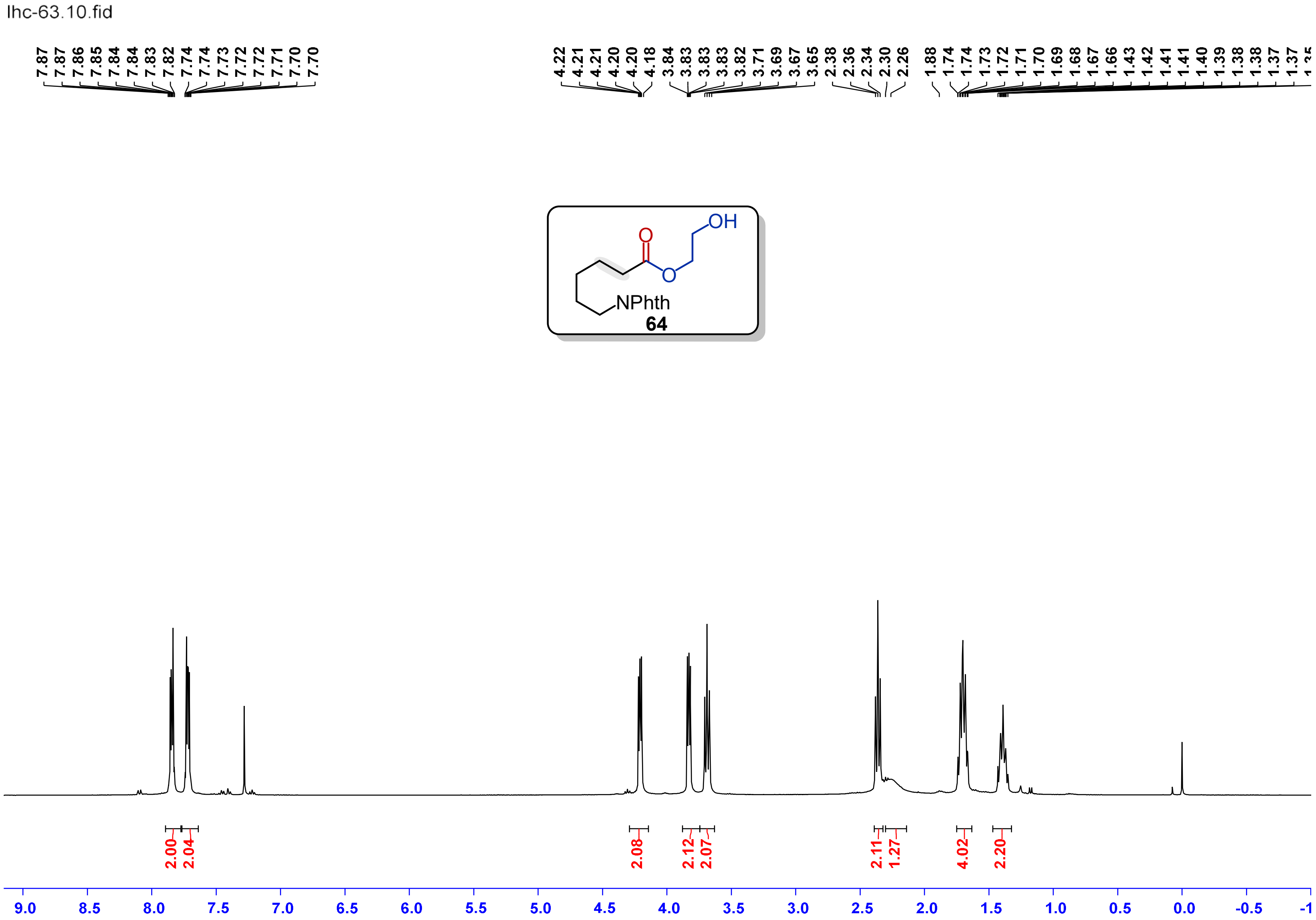

^{13}C NMR spectra for 64

lhc-63.11.fid

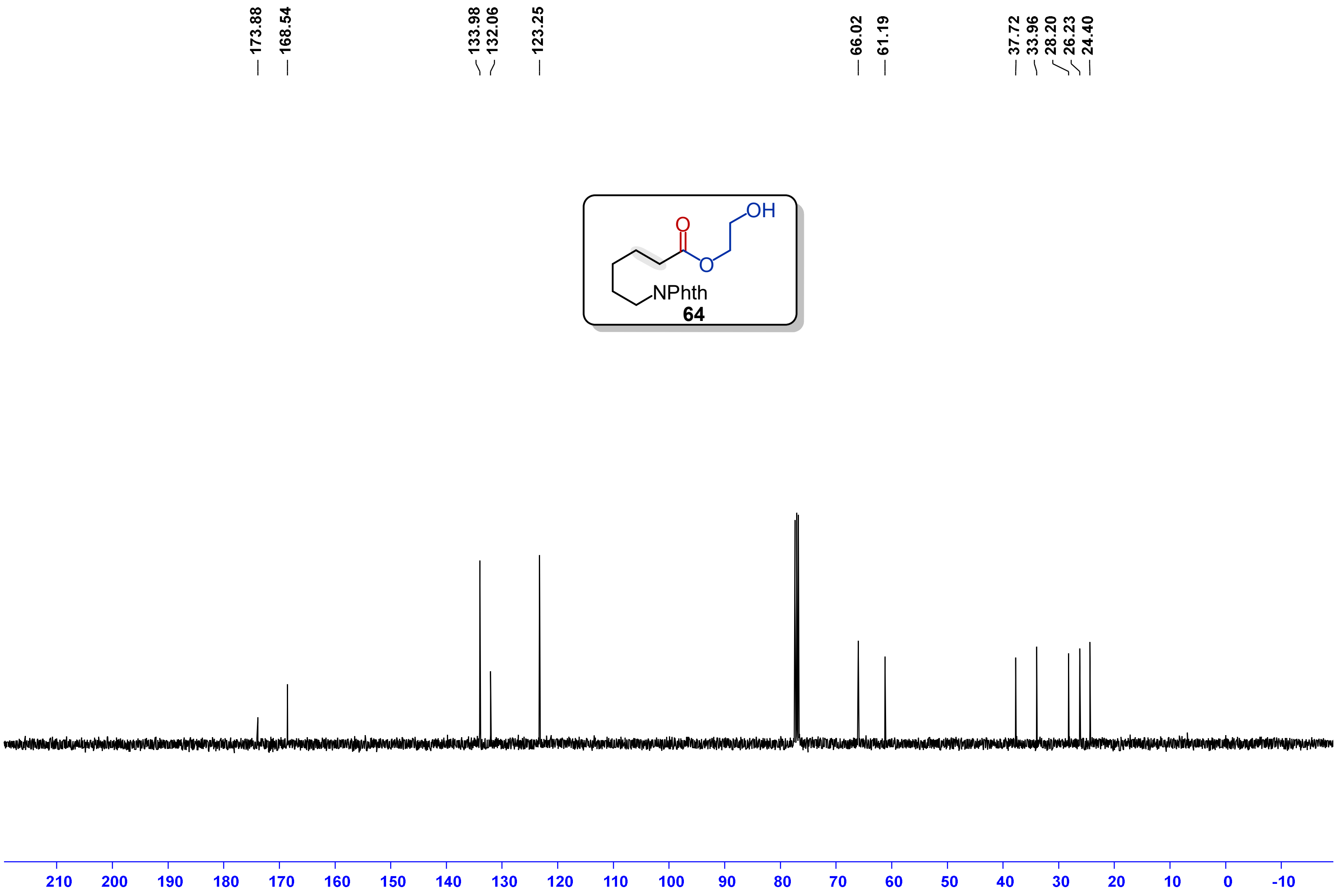

^{1}H NMR spectra for 65

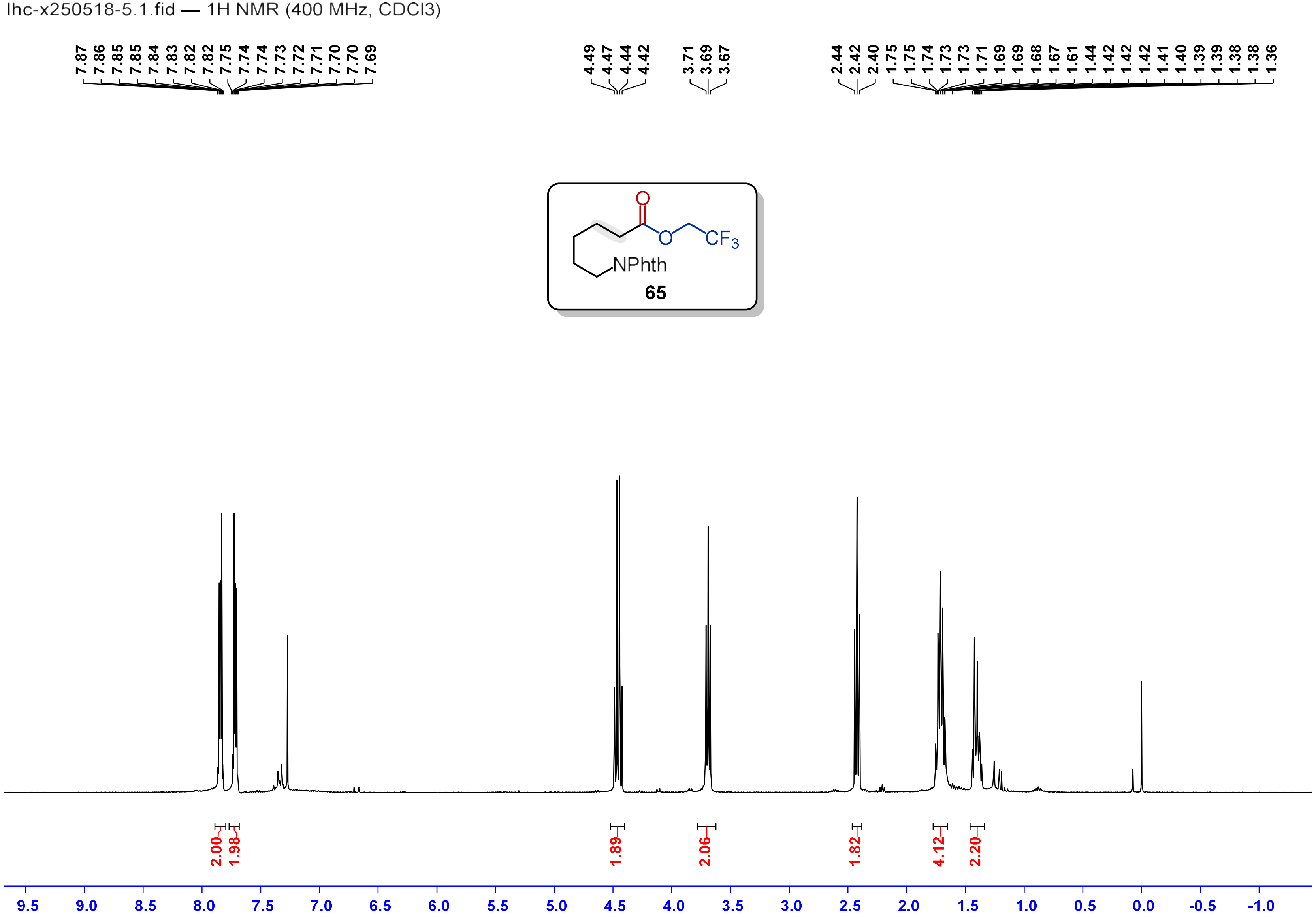

^{13}C NMR spectra for 65

lhc-x250518-5.2.fid — 1H NMR (400 MHz, CDCl3)

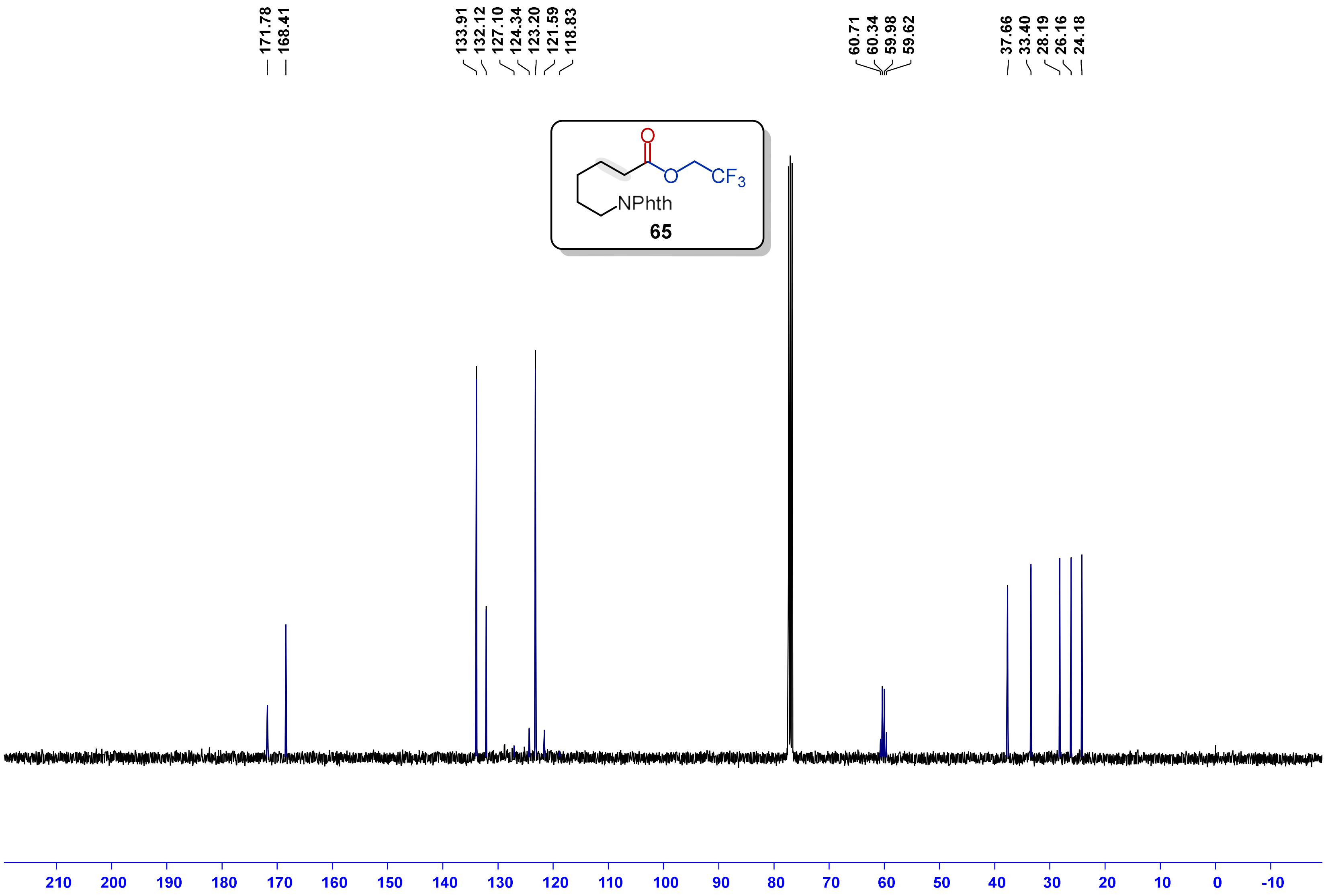

^{19}F NMR spectra for 65

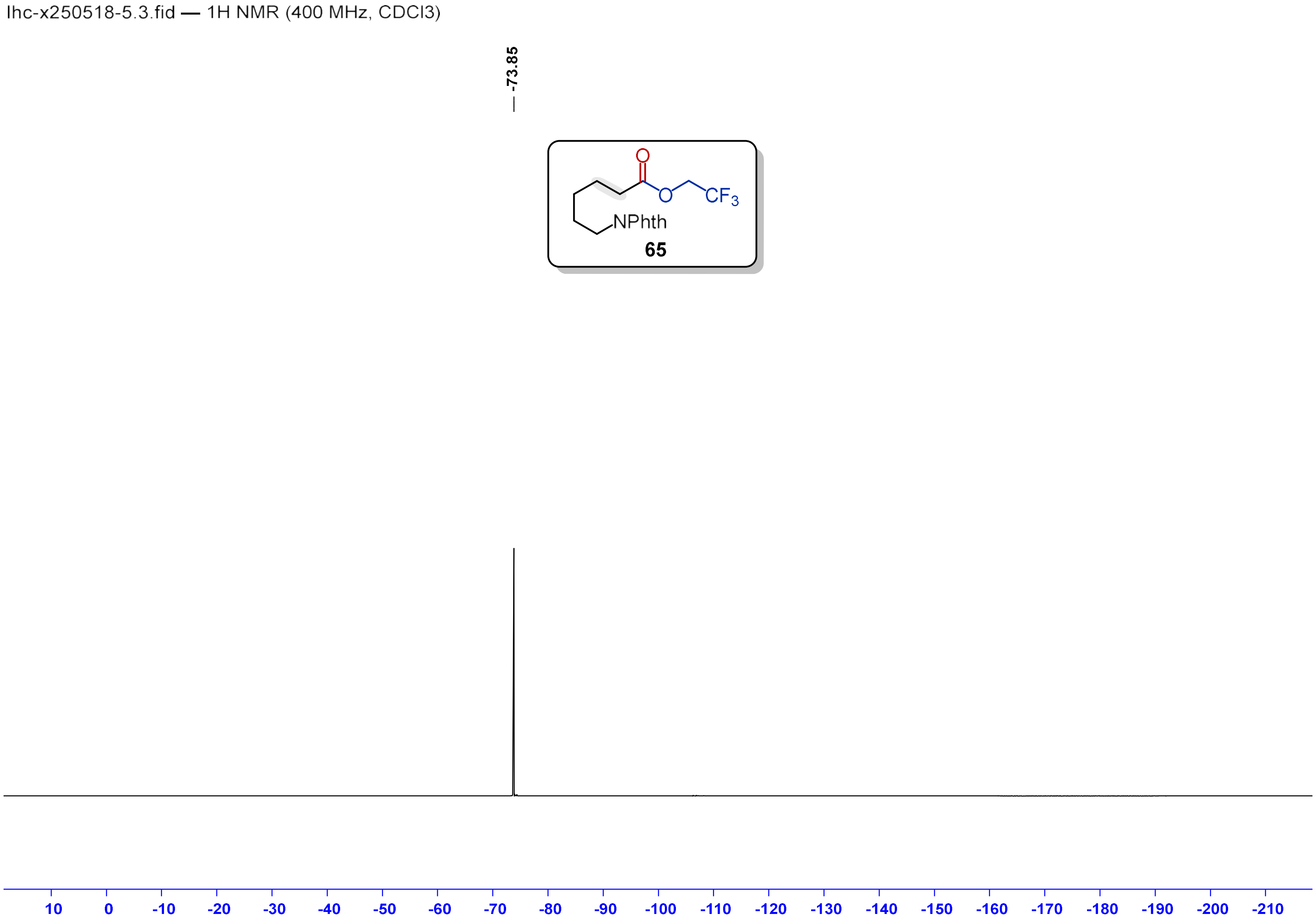

^{1}H NMR spectra for 66

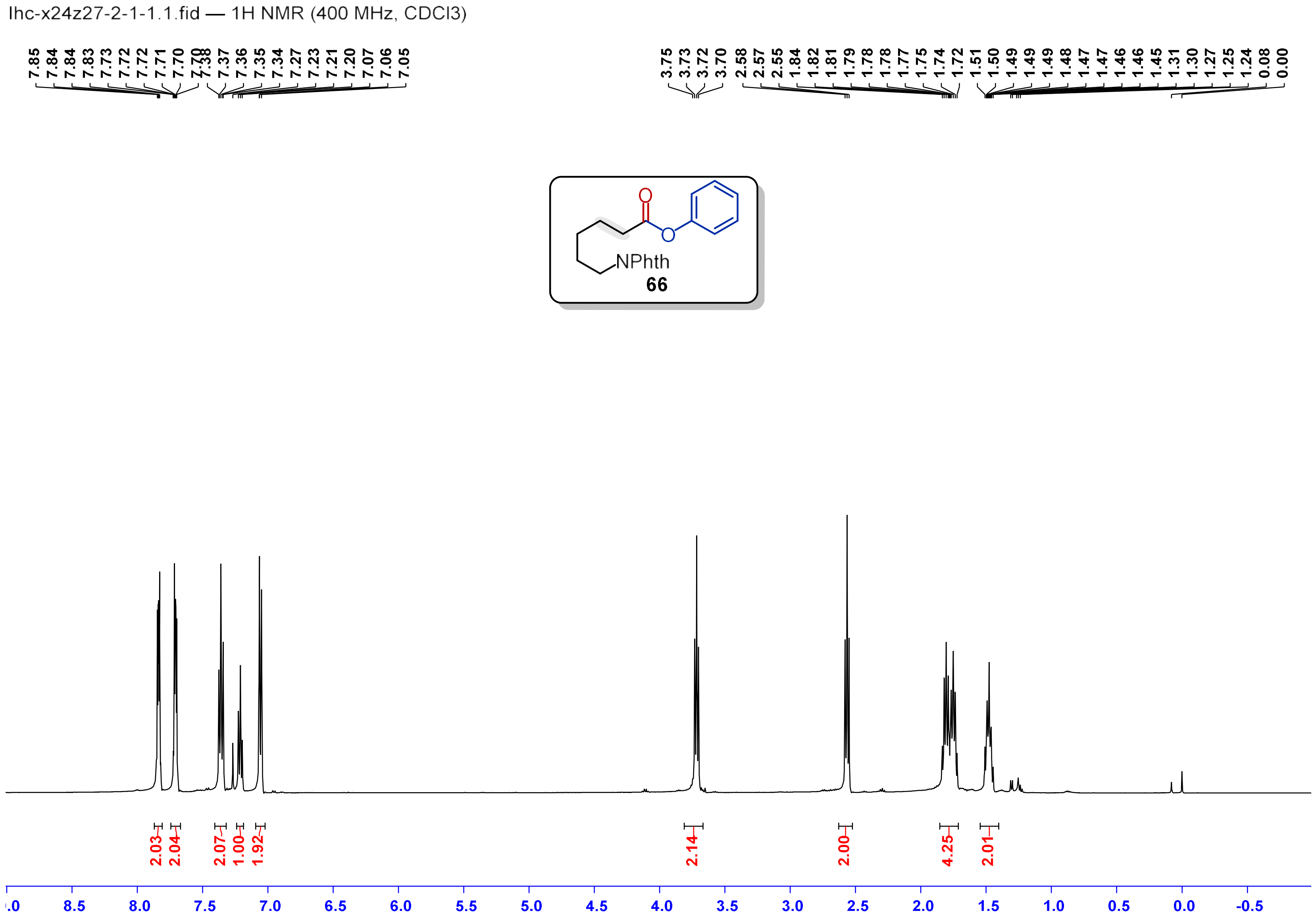

^{13}C NMR spectra for 66

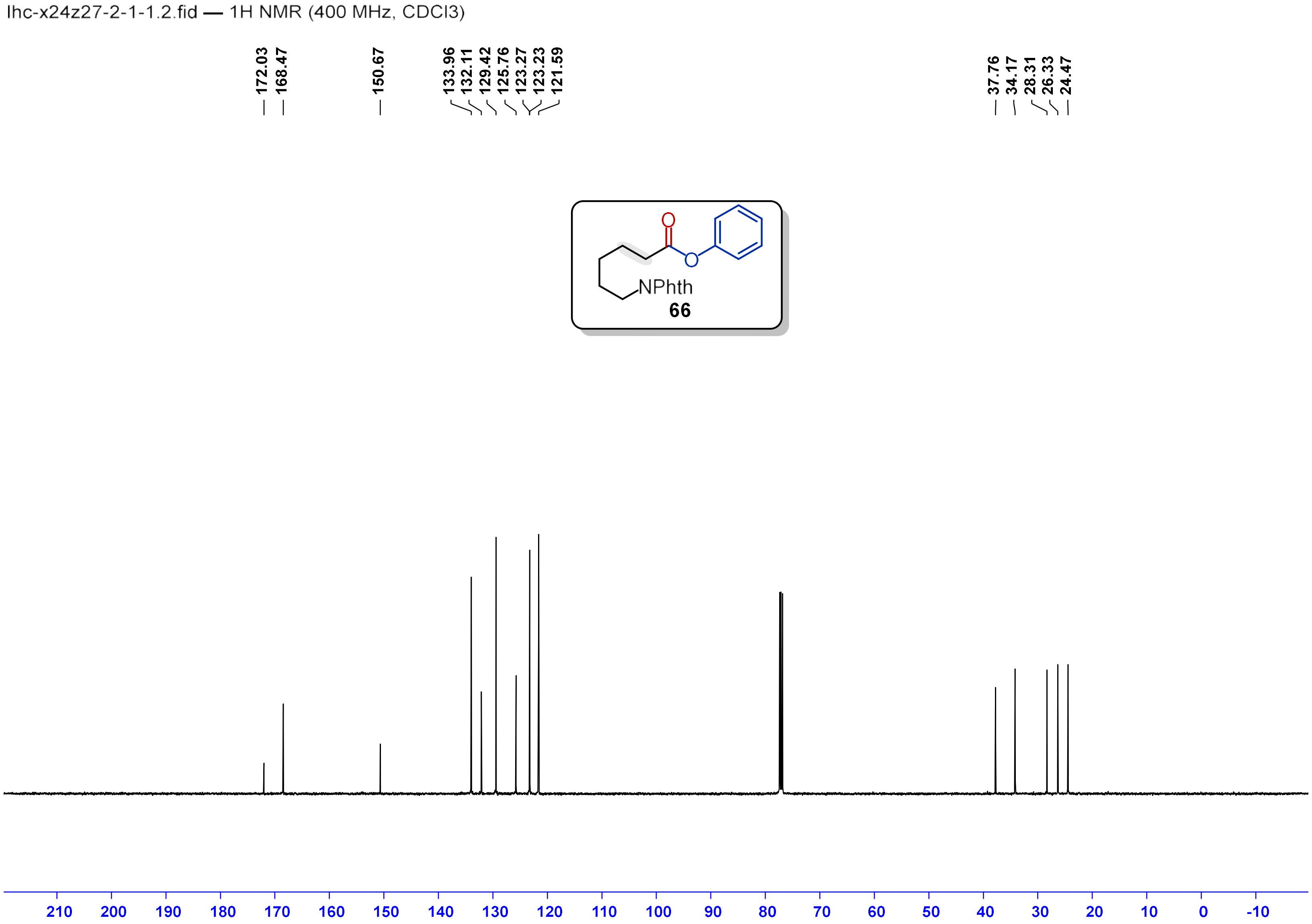

^{1}H NMR spectra for 67

lhc-66.10.fid

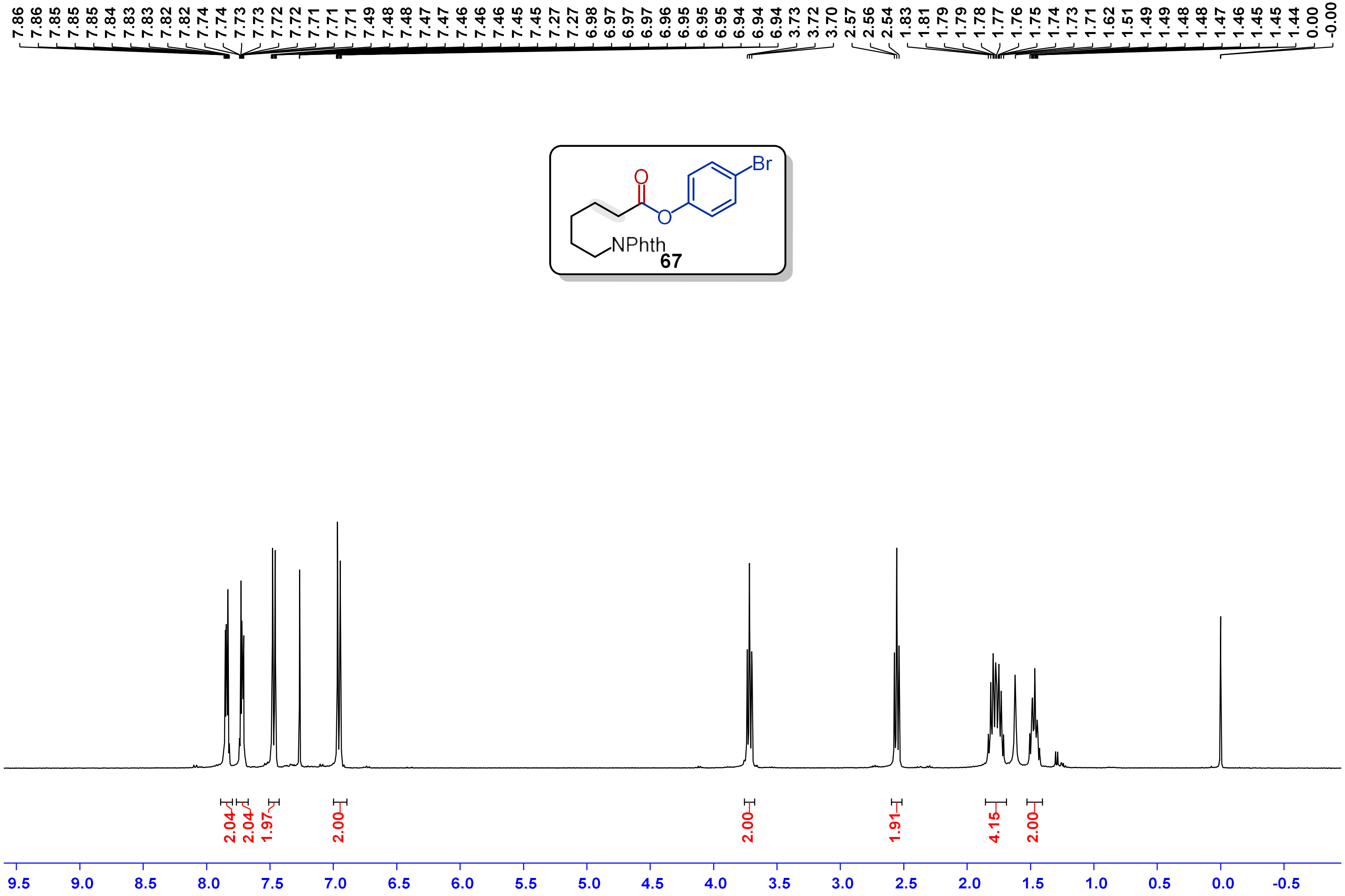

^{13}C NMR spectra for 67

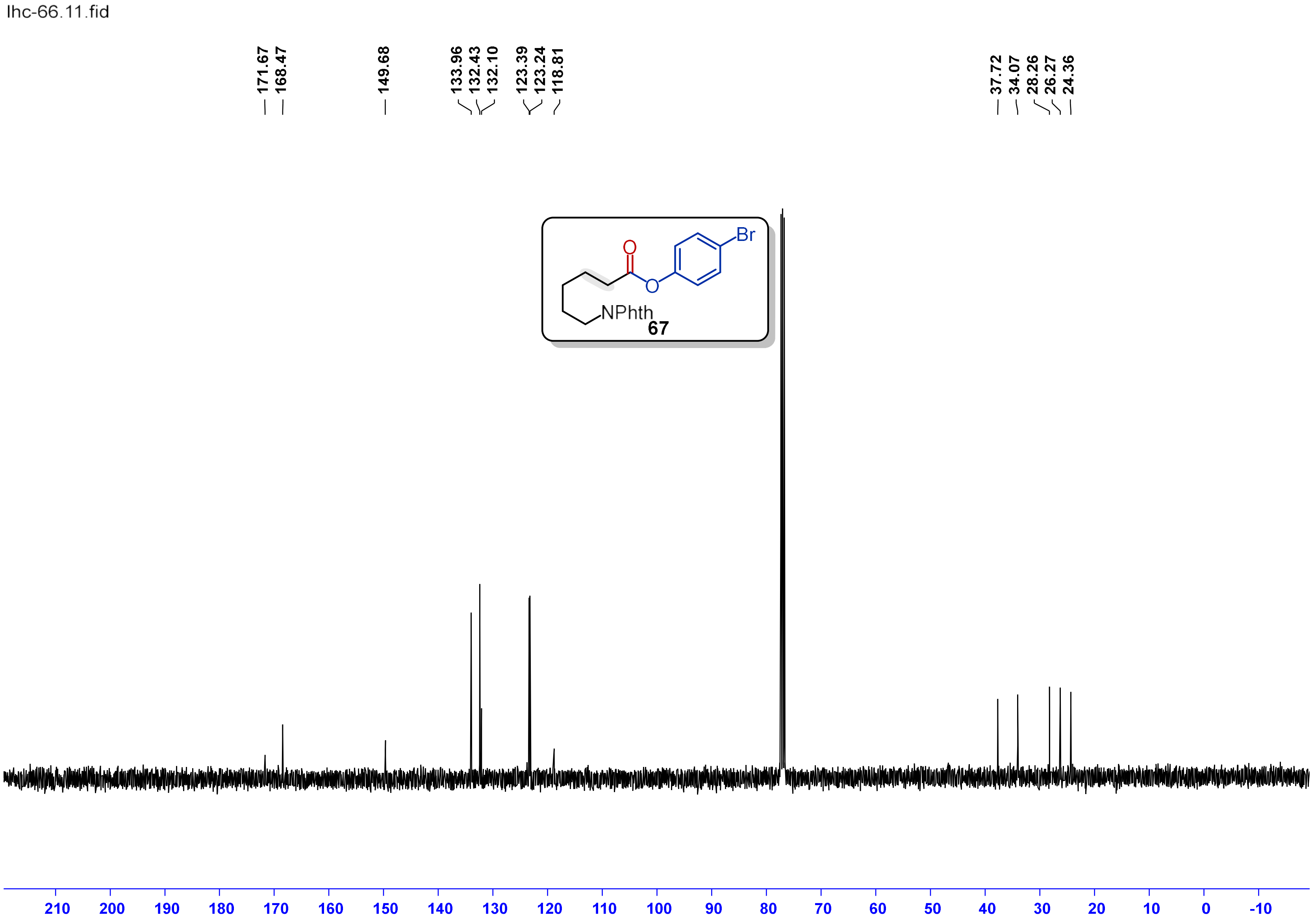

1H NMR spectra for 68

lhc-x25z26-5-1.1.fid — 1H NMR (400 MHz, CDCl3)

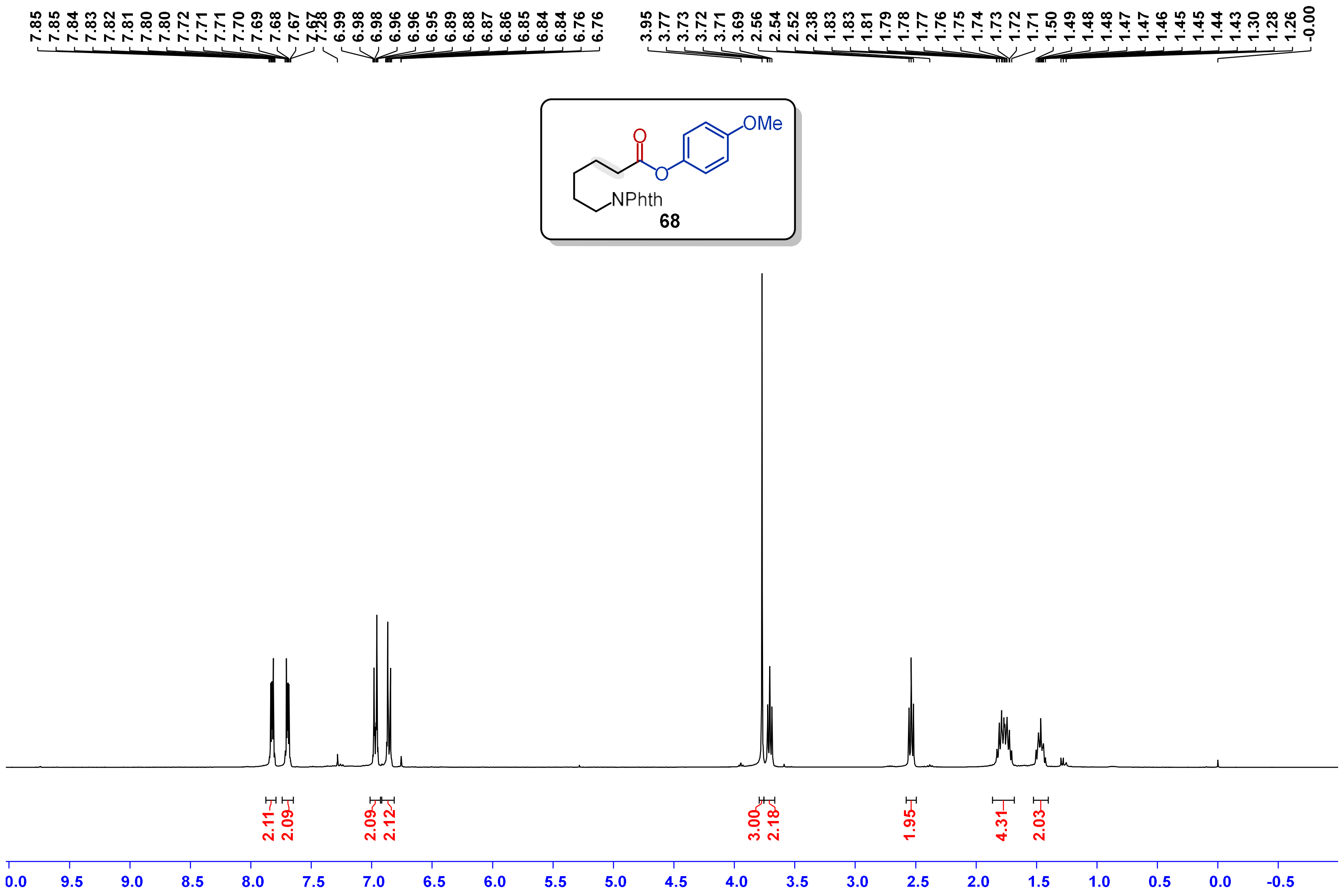

^{13}C NMR spectra for 68

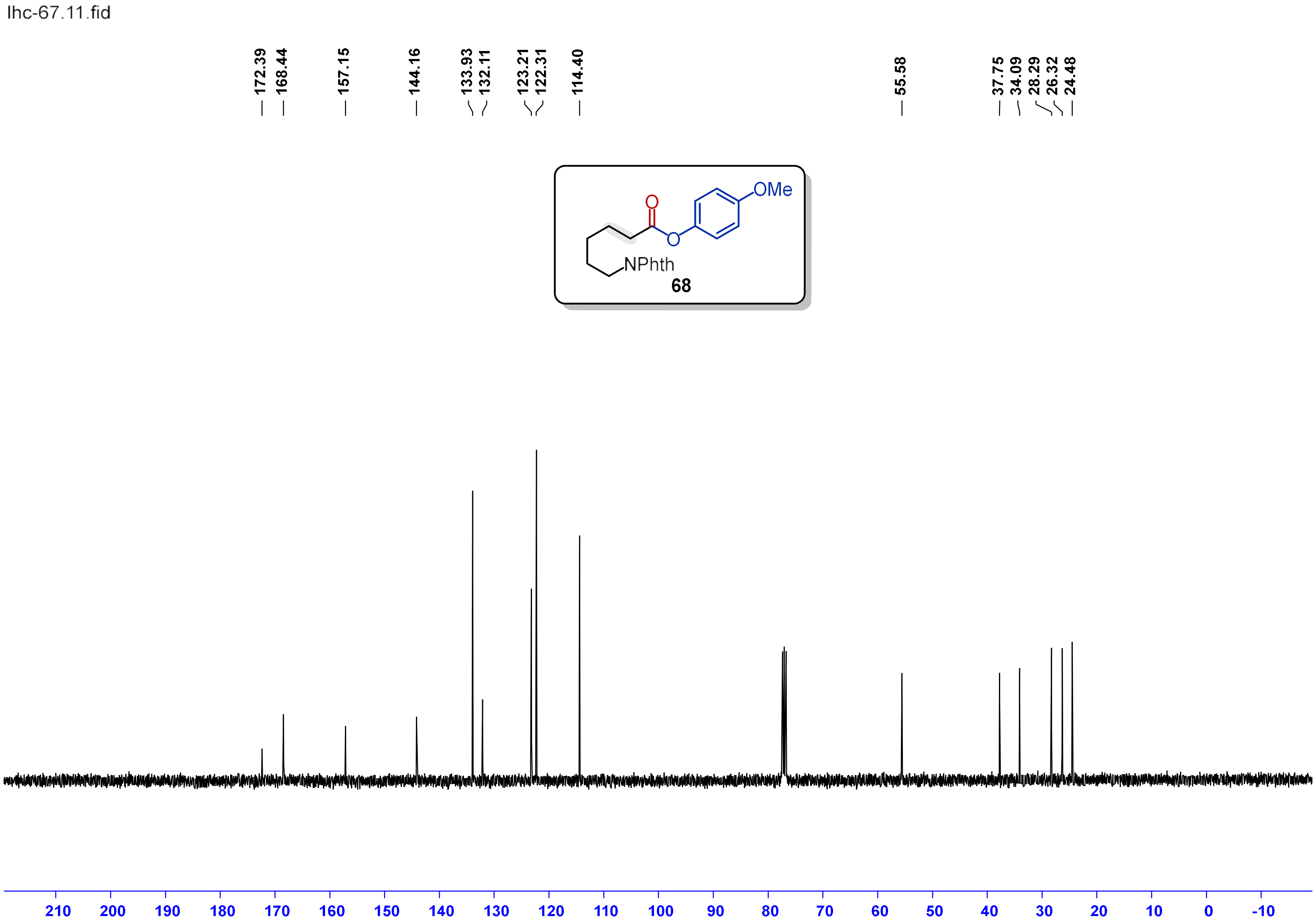

^{1}H NMR spectra for 69

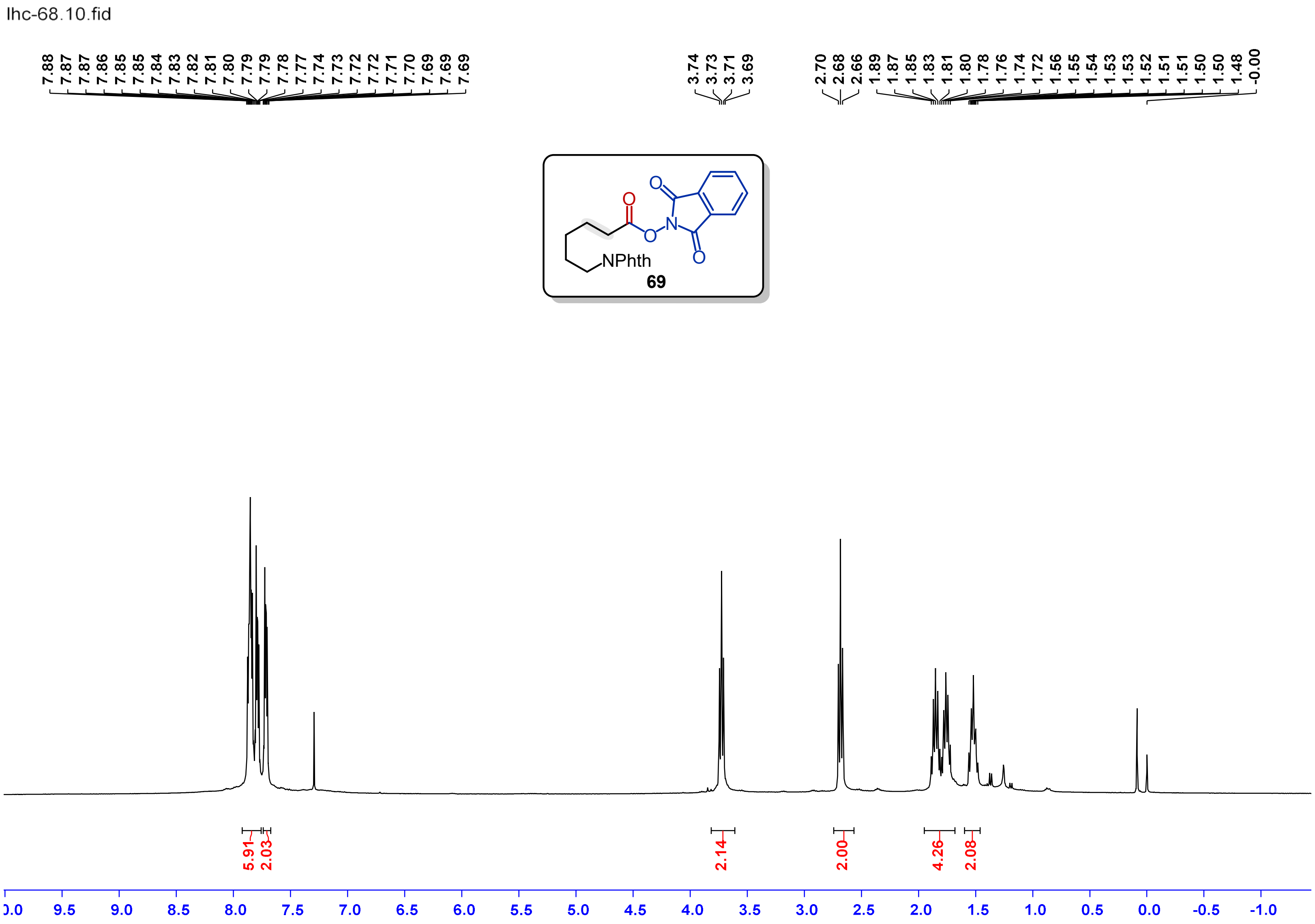

^{13}C NMR spectra for 69

lhc-68.11.fid

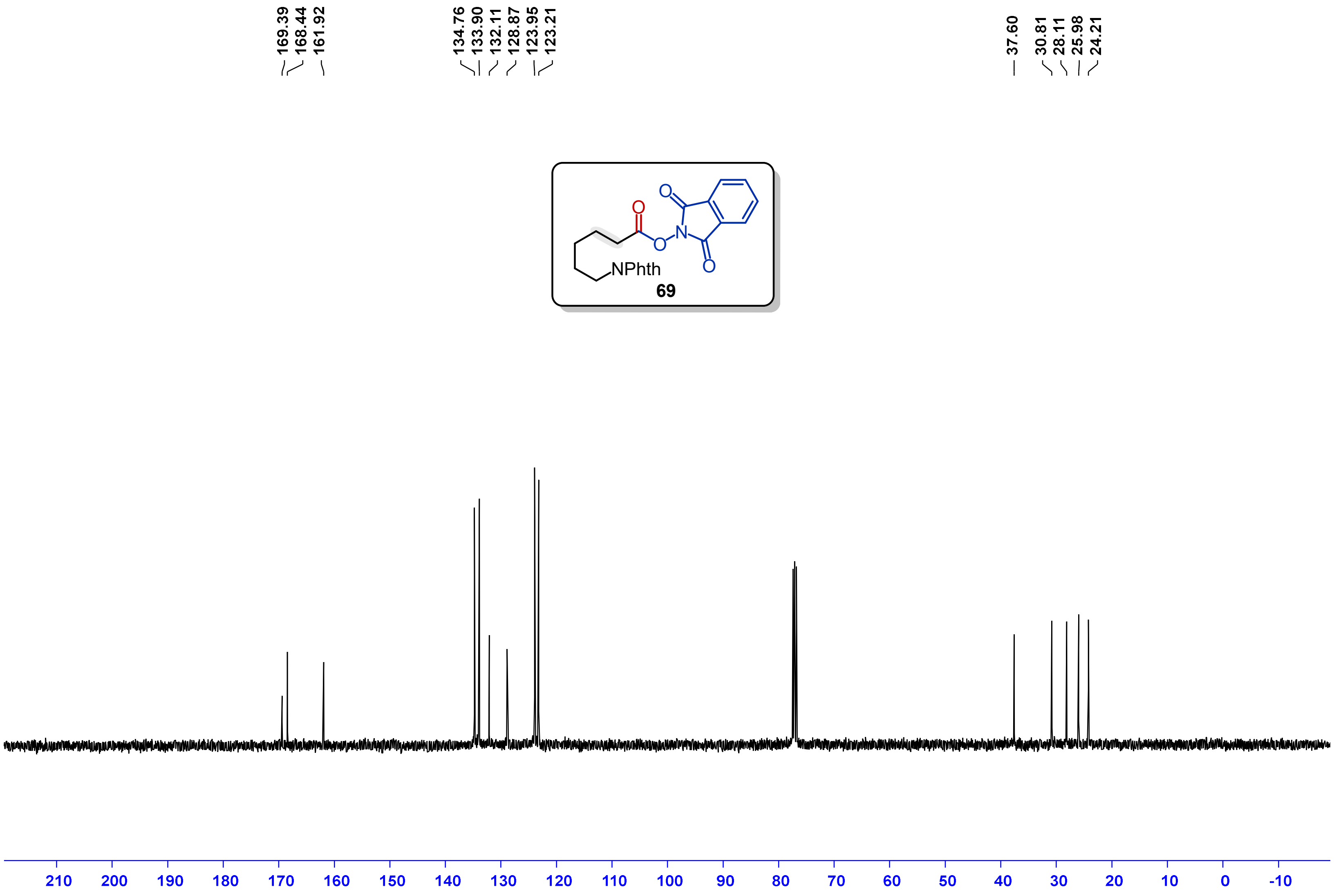

^{1}H NMR spectra for 70

lhc-x250309-4.1.fid — 1H NMR (400 MHz, CDCl3)

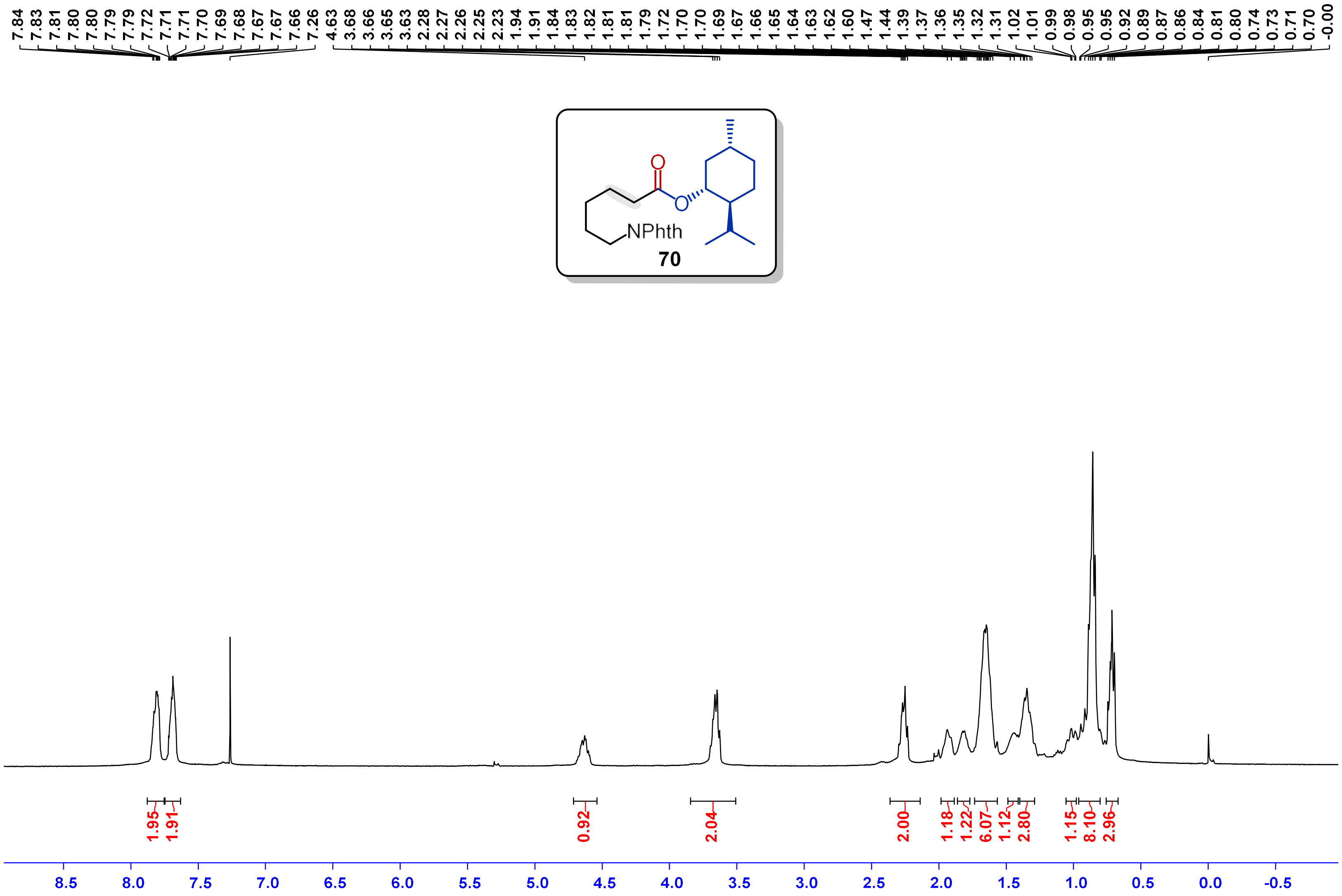

^{13}C NMR spectra for 70

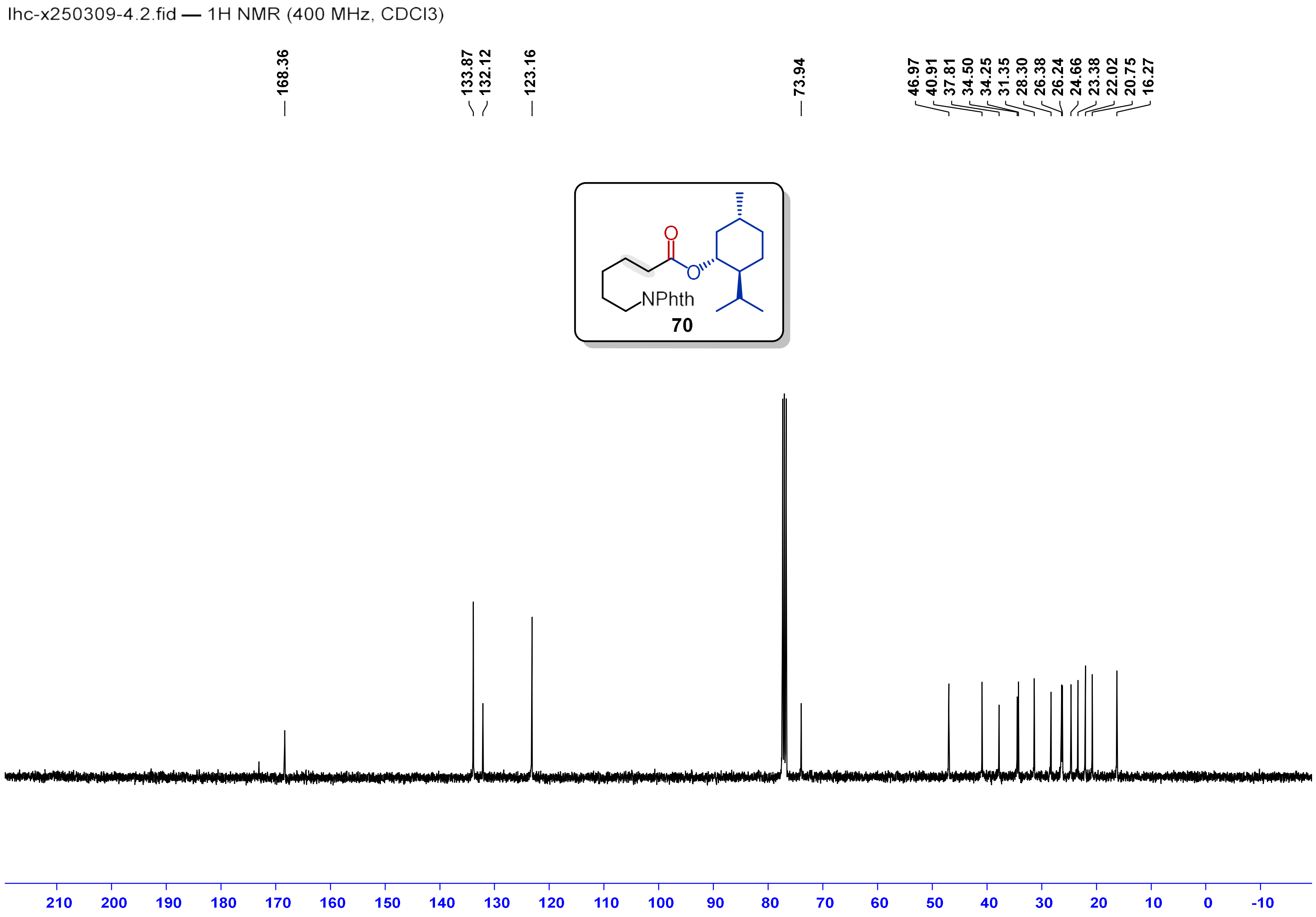

^{1}H NMR spectra for 71

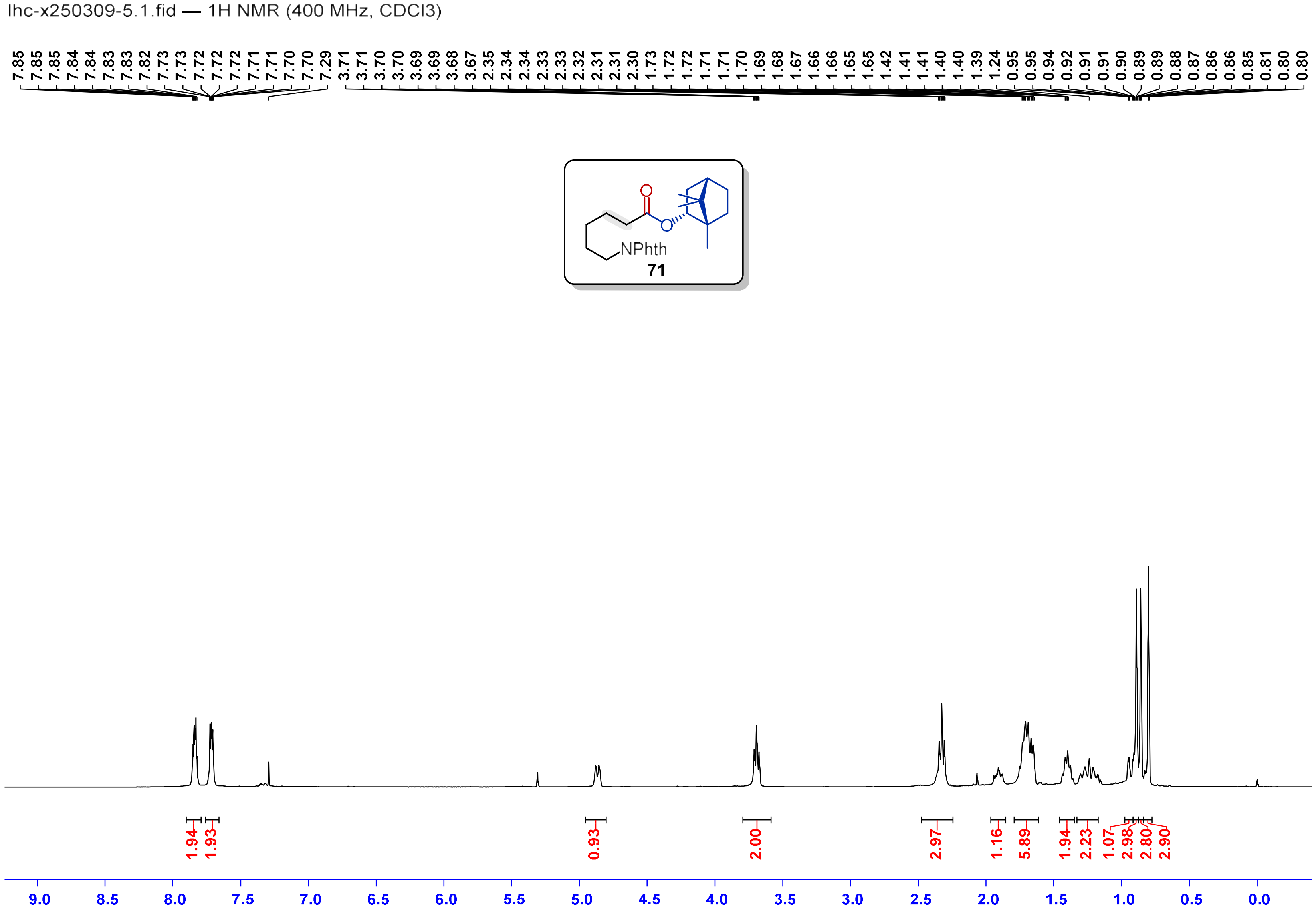

^{13}C NMR spectra for 71

lhc-x250309-5.2.fid — 1H NMR (400 MHz, CDCl3)

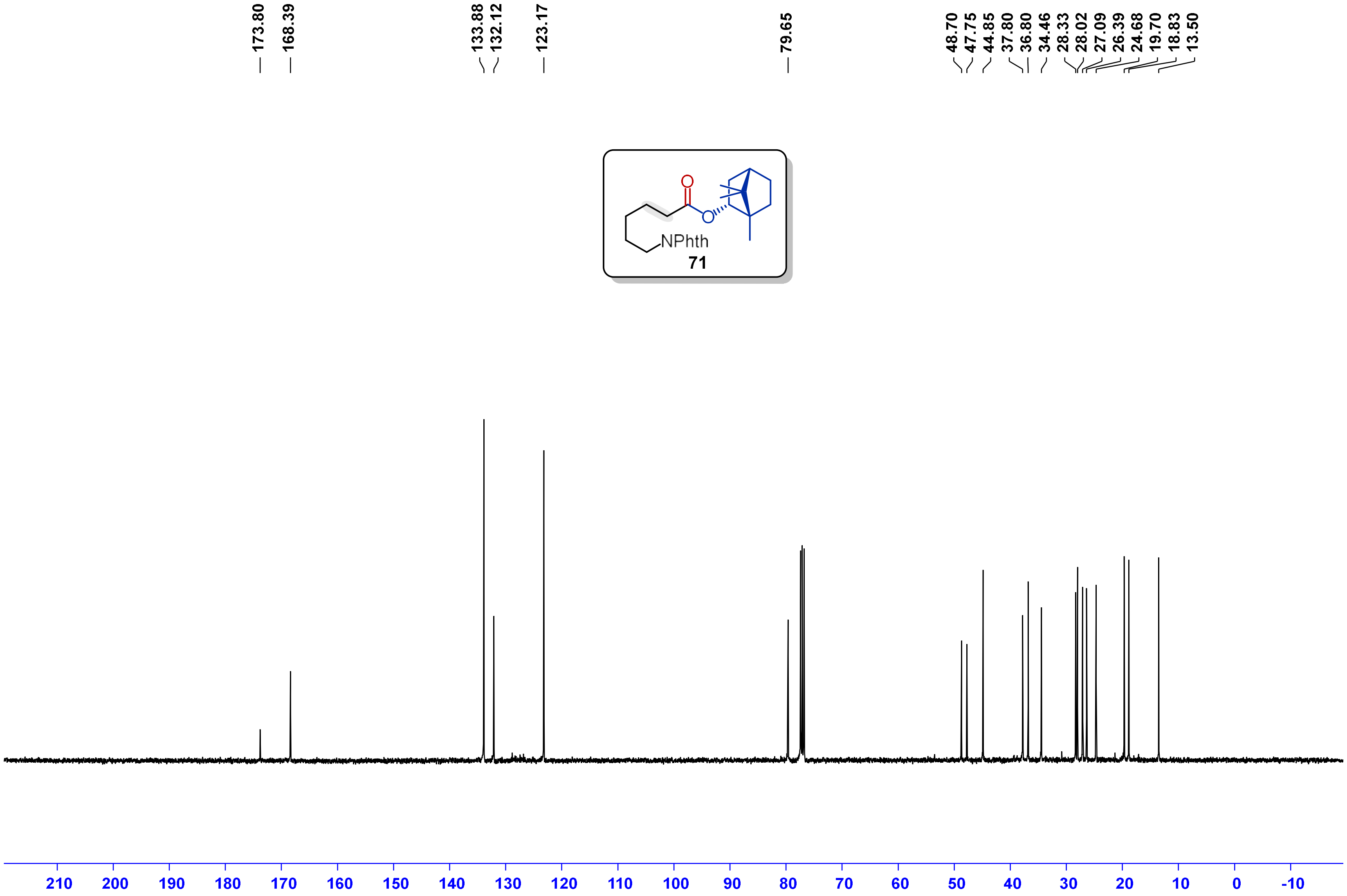

^{1}H NMR spectra for 72

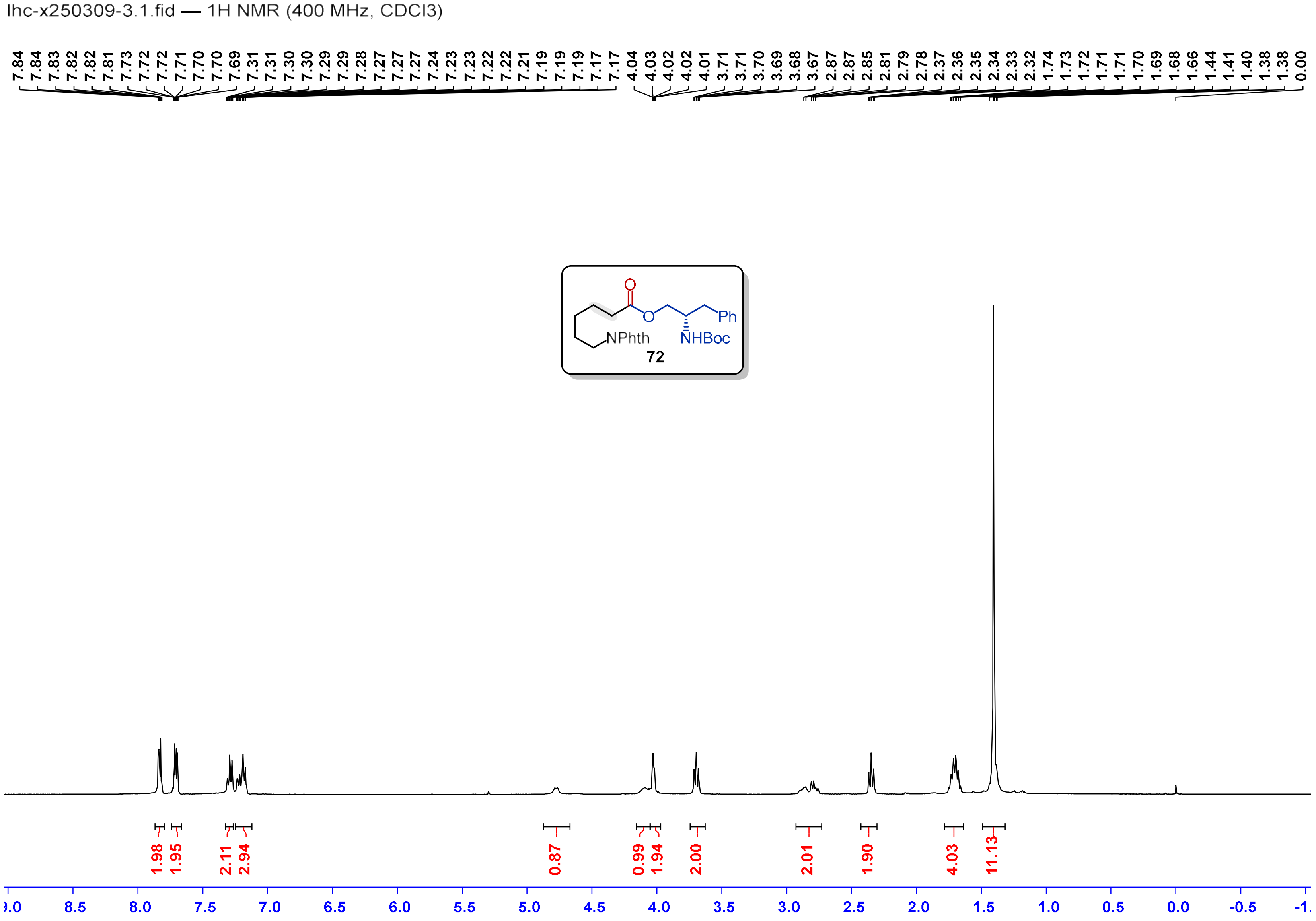

^{13}C NMR spectra for 72

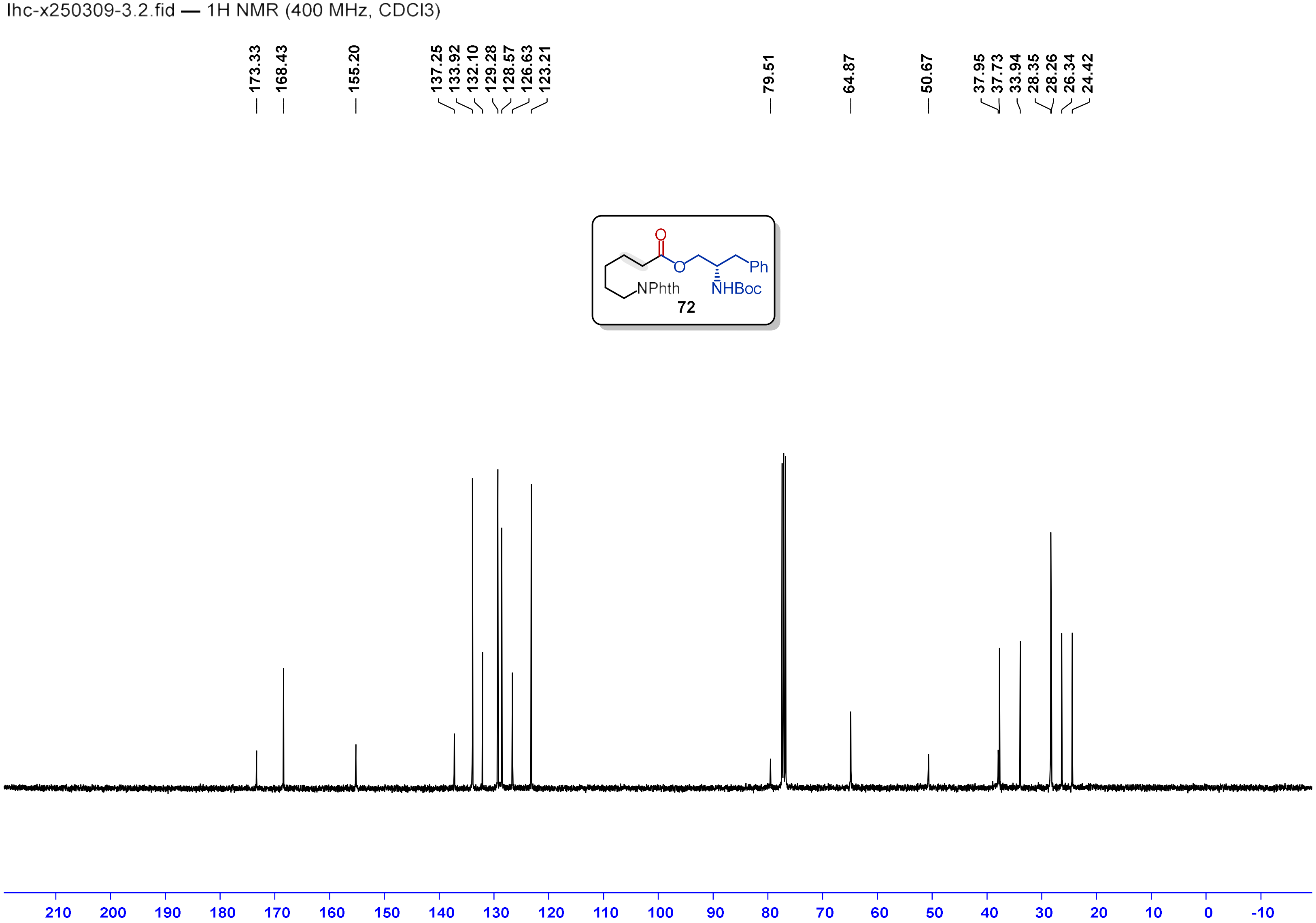

^{1}H NMR spectra for 73

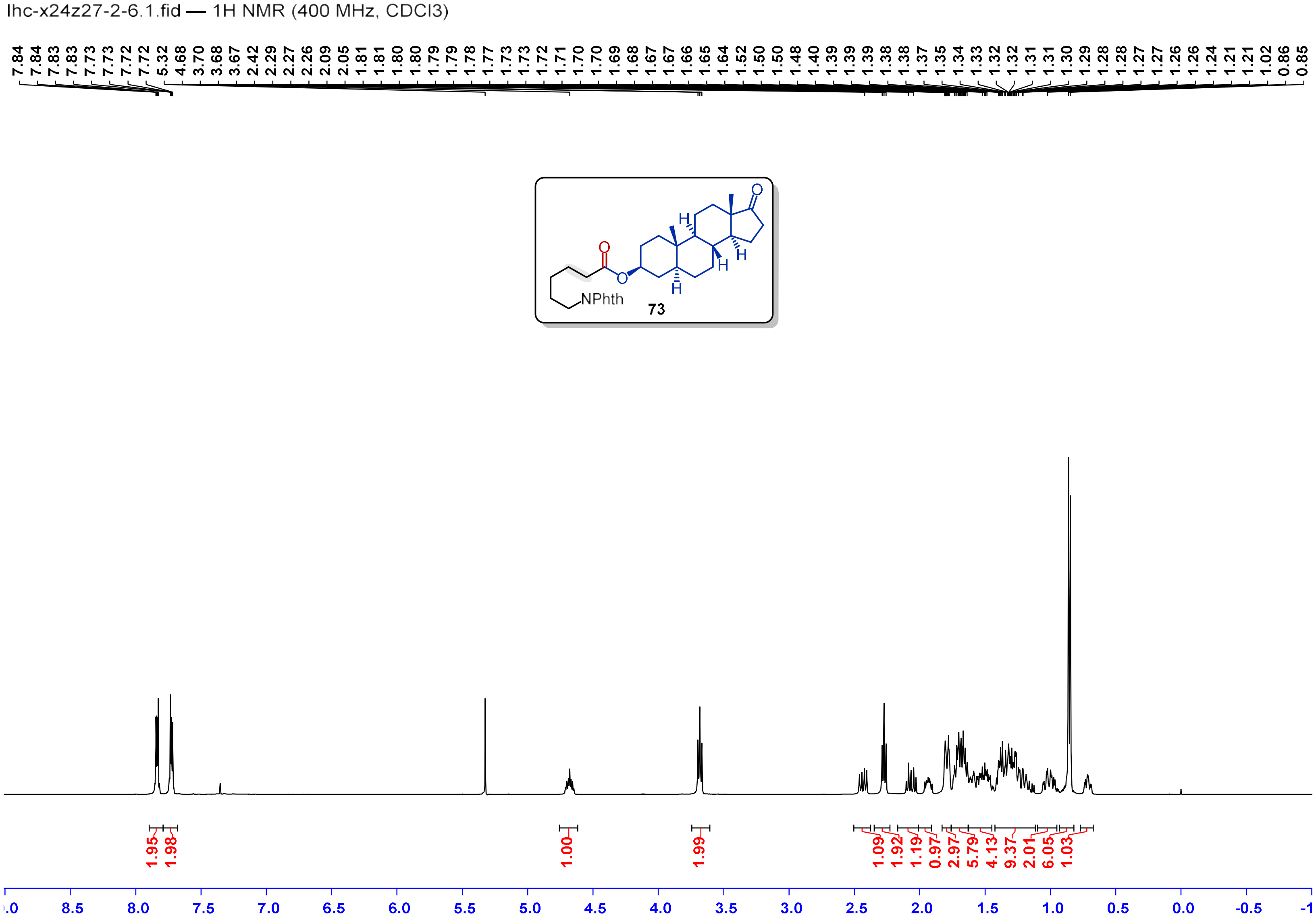

^{13}C NMR spectra for 73

lhc-x24z27-2-6.2.fid — 1H NMR (400 MHz, CDCl3)

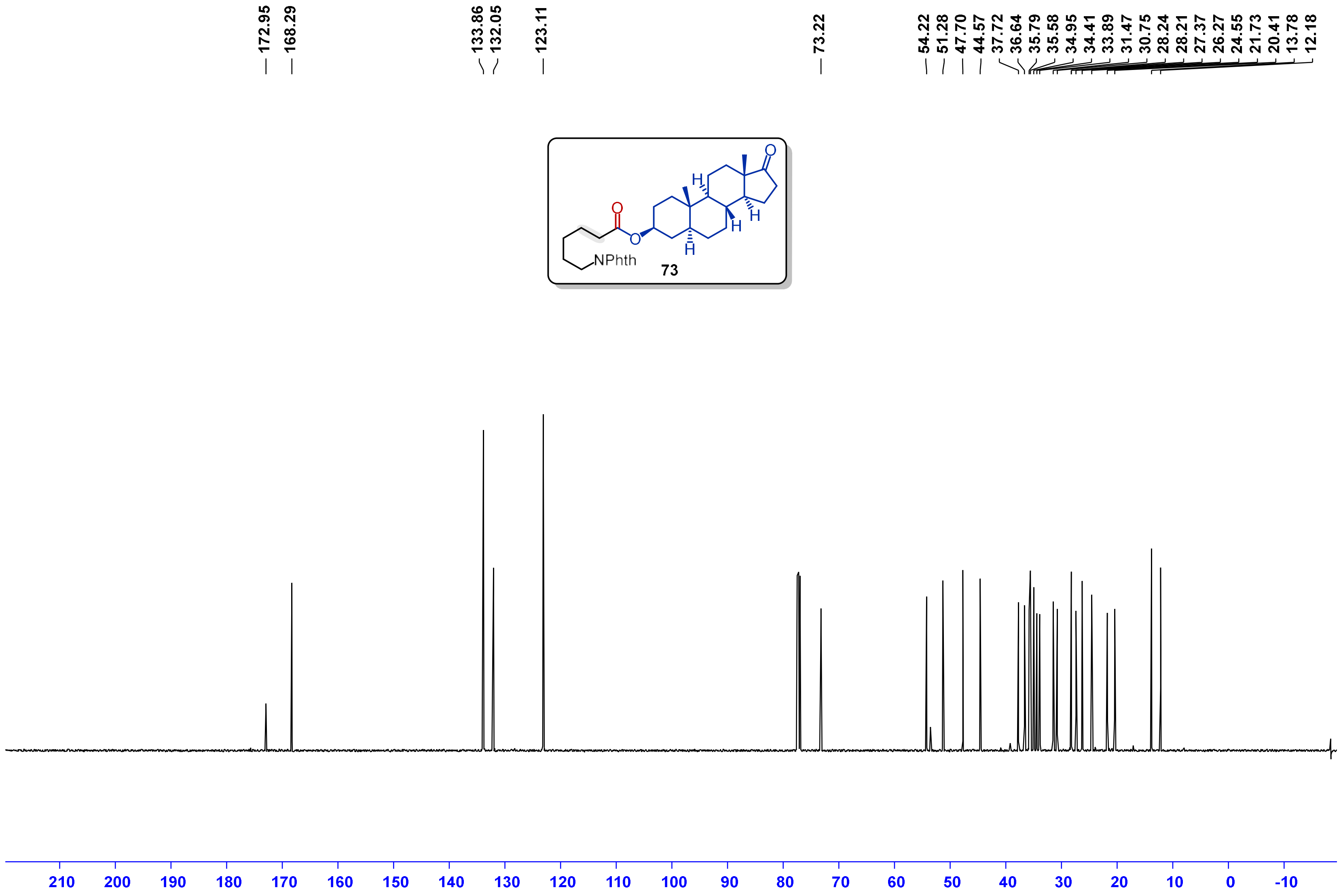

^{1}H NMR spectra for 74

lhc-x250330-1；73.1.fid — 1H NMR (400 MHz, CDCl3)

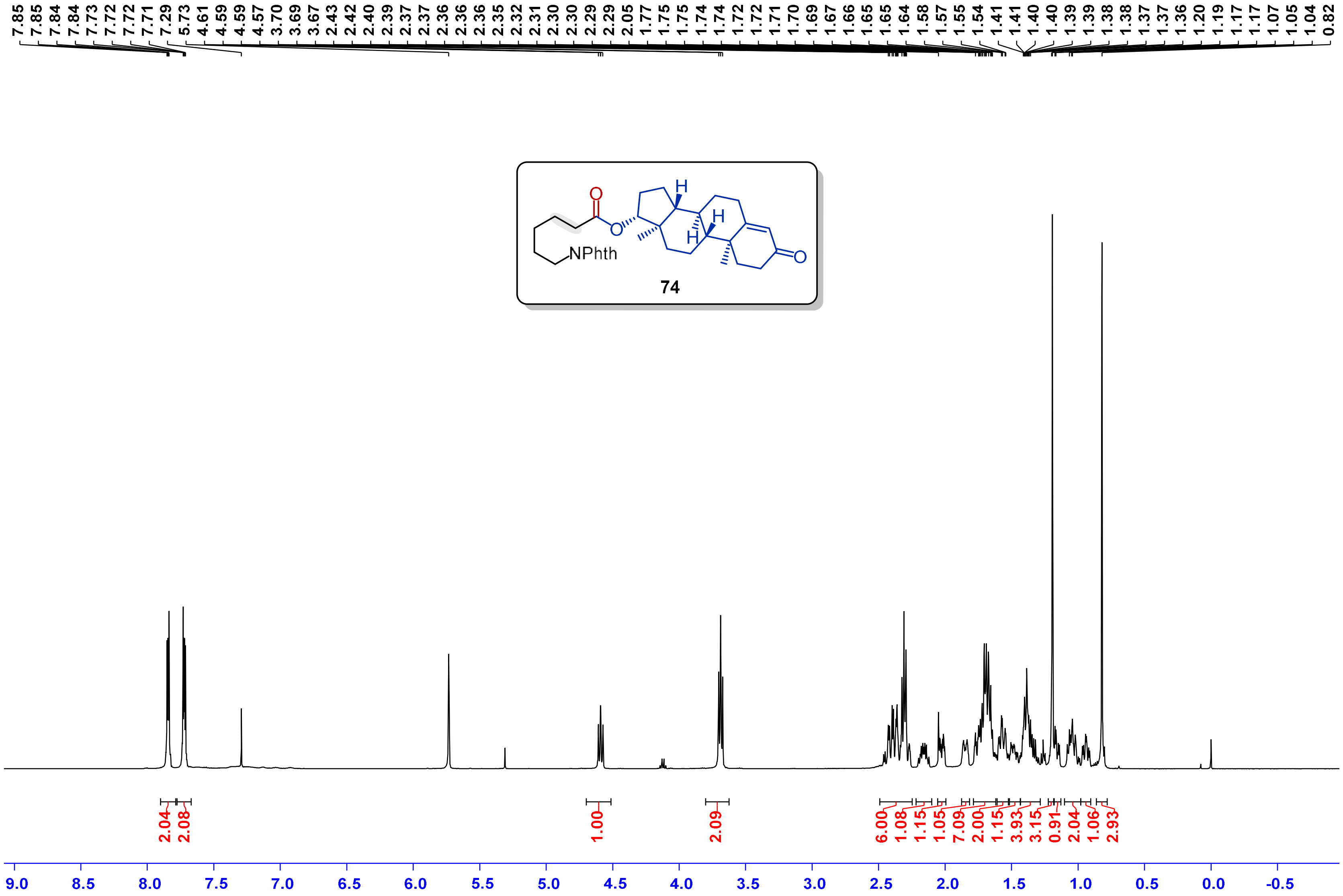

^{13}C NMR spectra for 74

lhc-x250330-1；73.2.fid — 1H NMR (400 MHz, CDCl3)

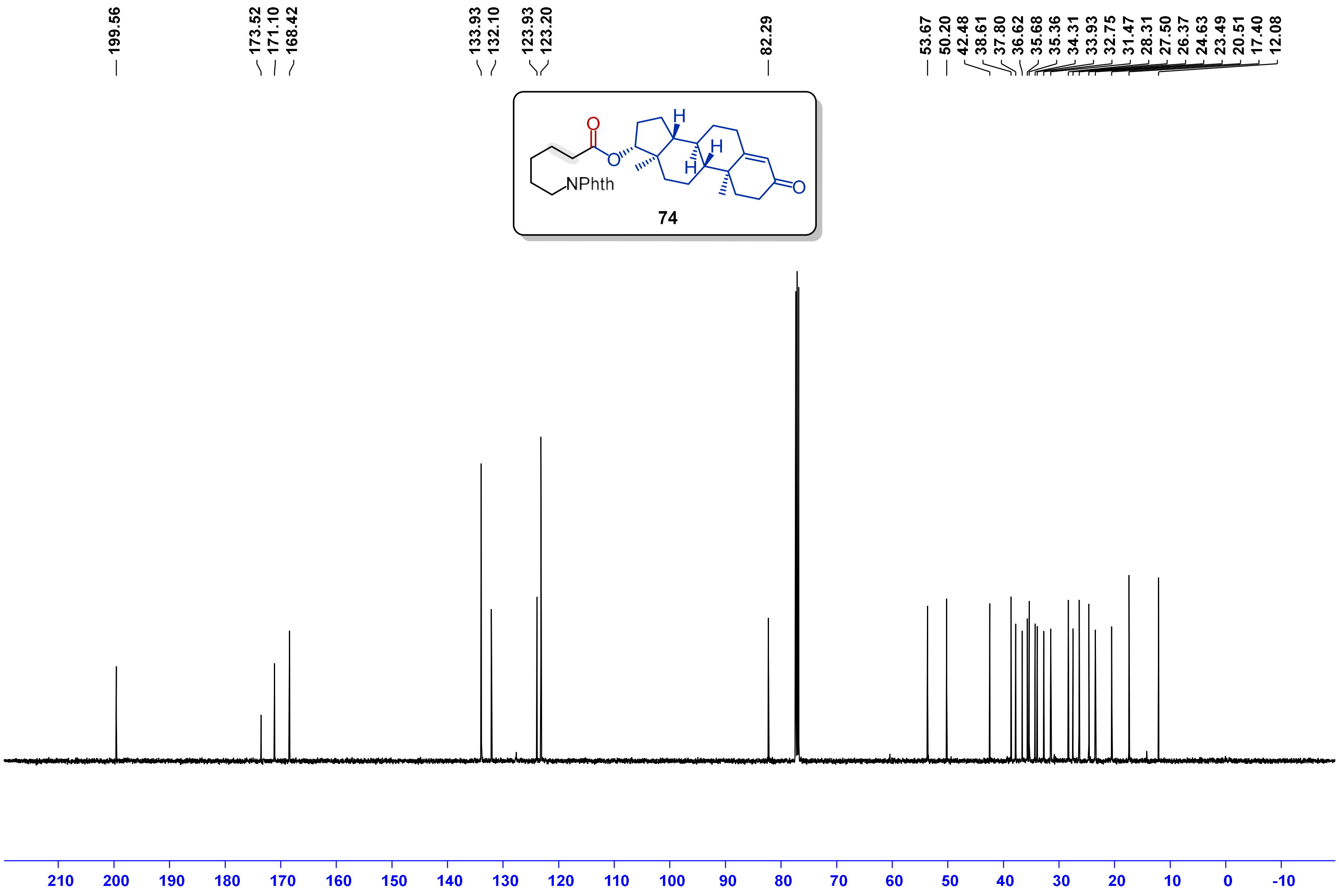

^{1}H NMR spectra for 75

lhc-x24z27-2-7.1.fid — 1H NMR (400 MHz, CDCl3)

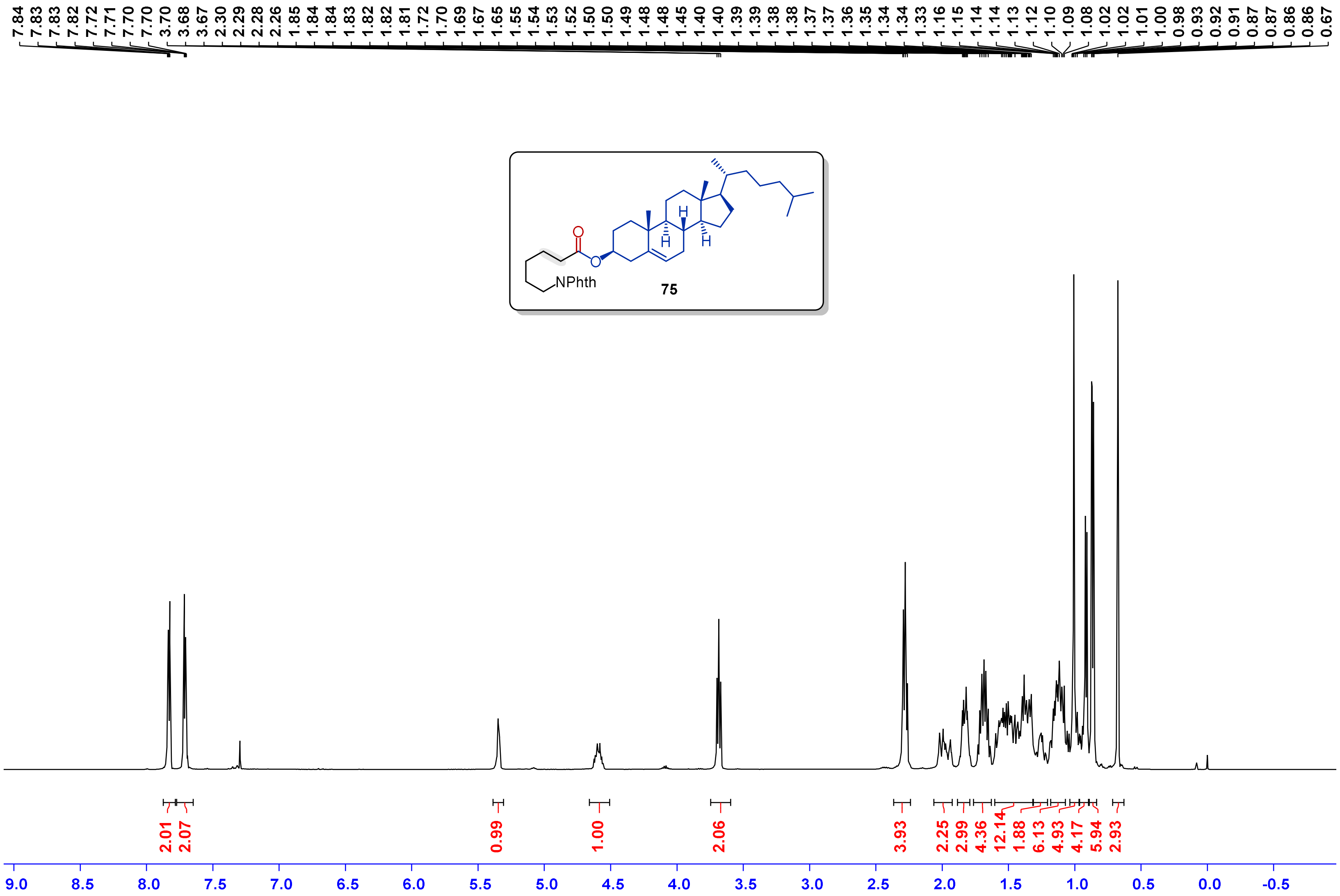

^{13}C NMR spectra for 75

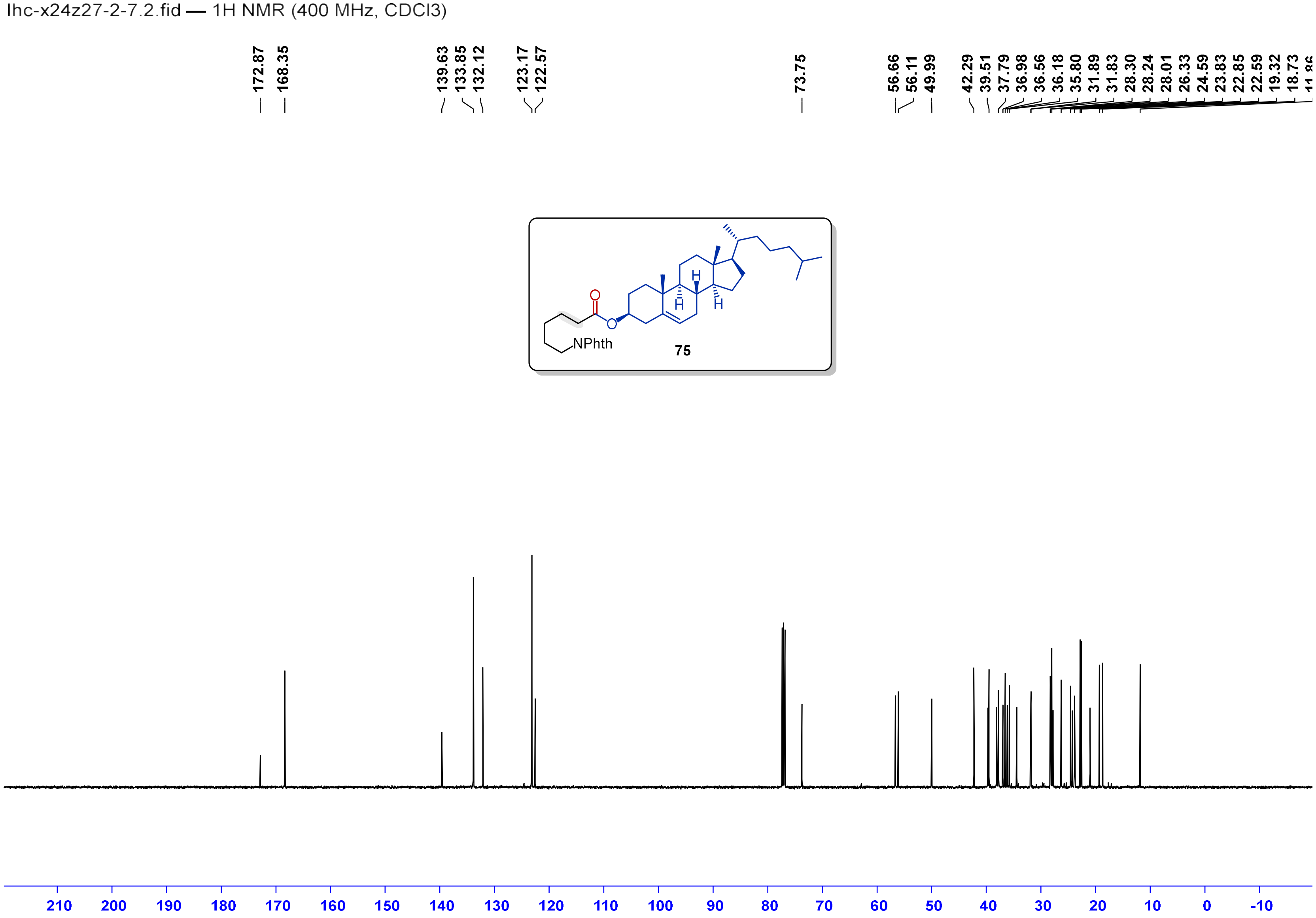

^{1}H NMR spectra for 76

lhc-x24z27-8.1.fid — 1H NMR (400 MHz, CDCl3)

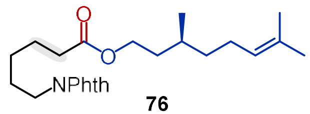

76

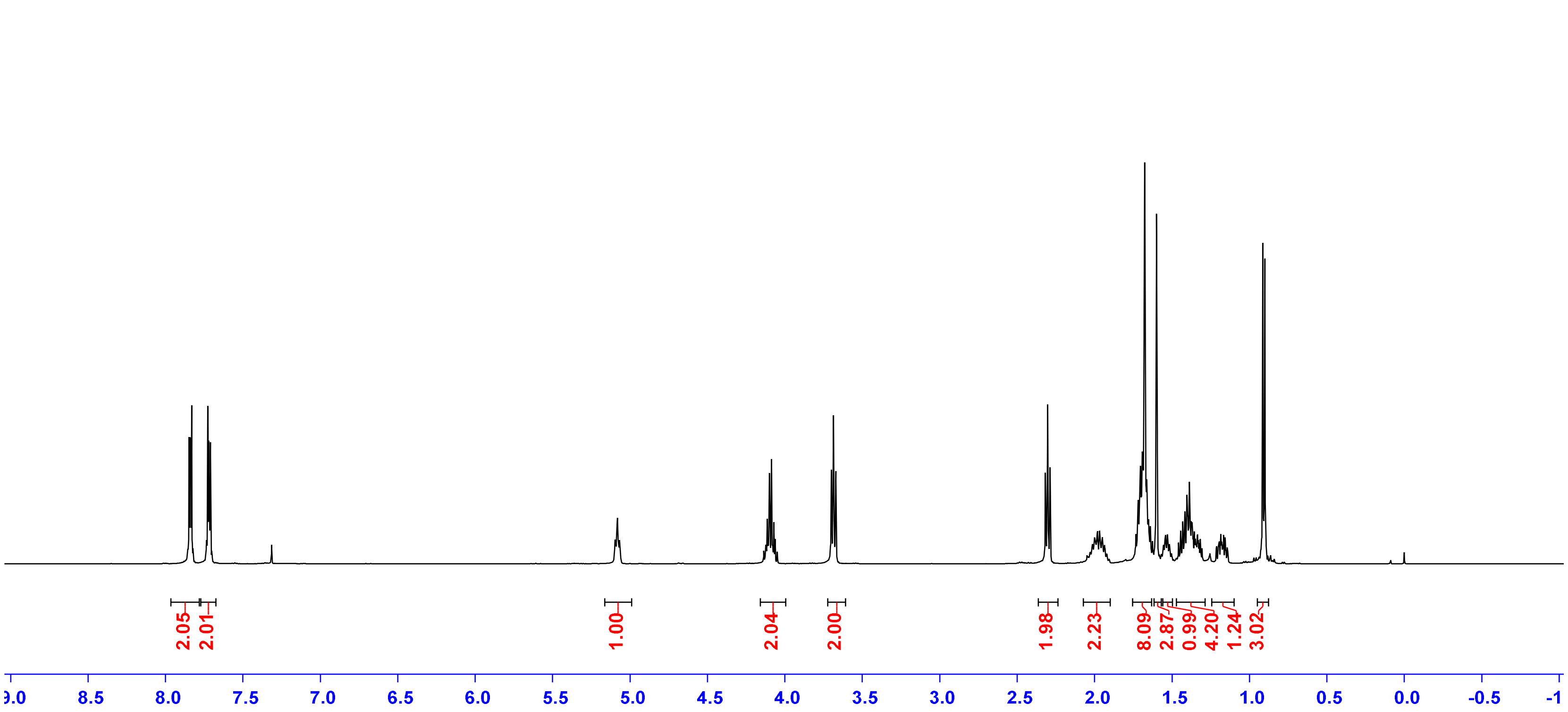

^{13}C NMR spectra for 76

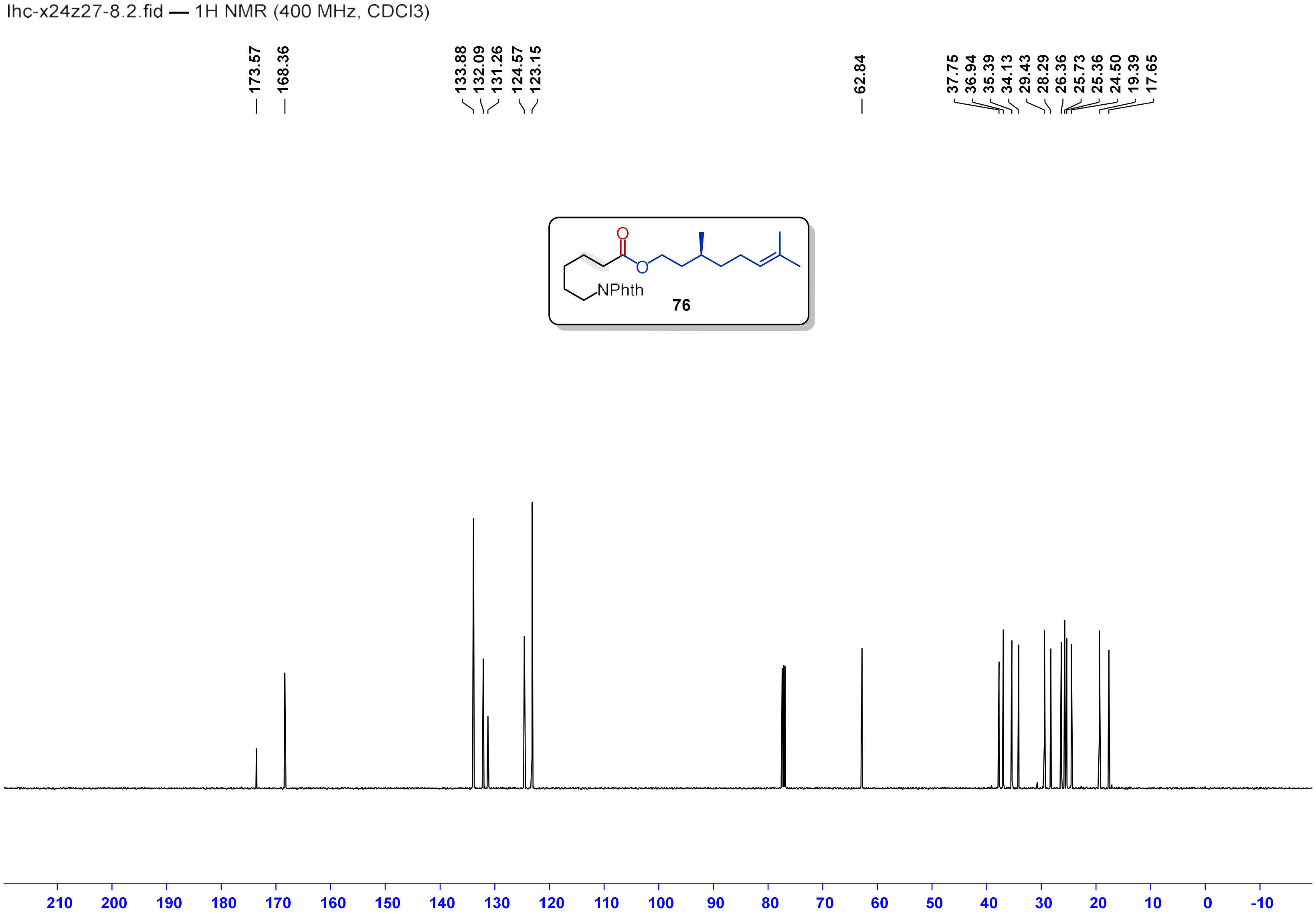

^{1}H NMR spectra for 77

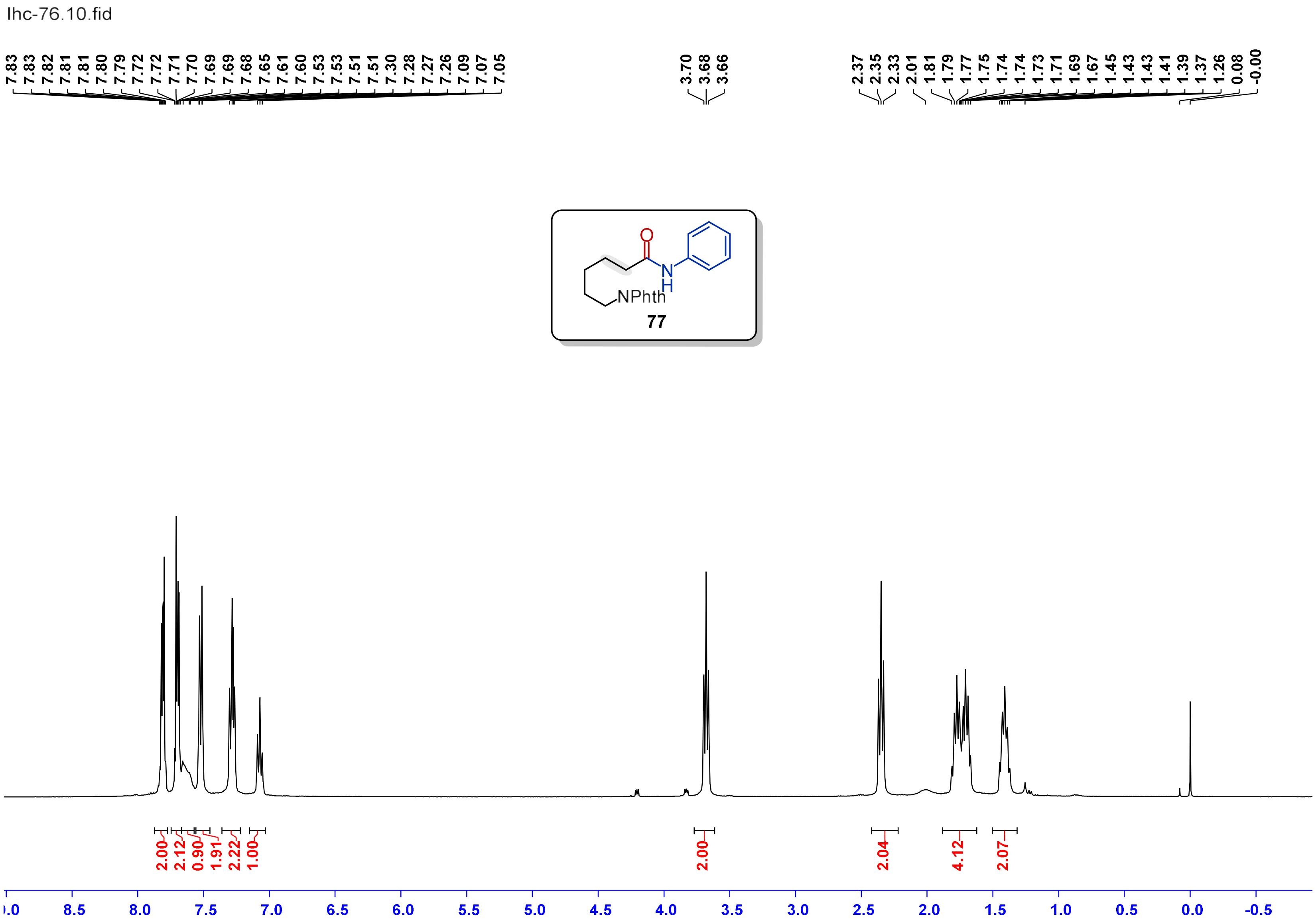

^{13}C NMR spectra for 77

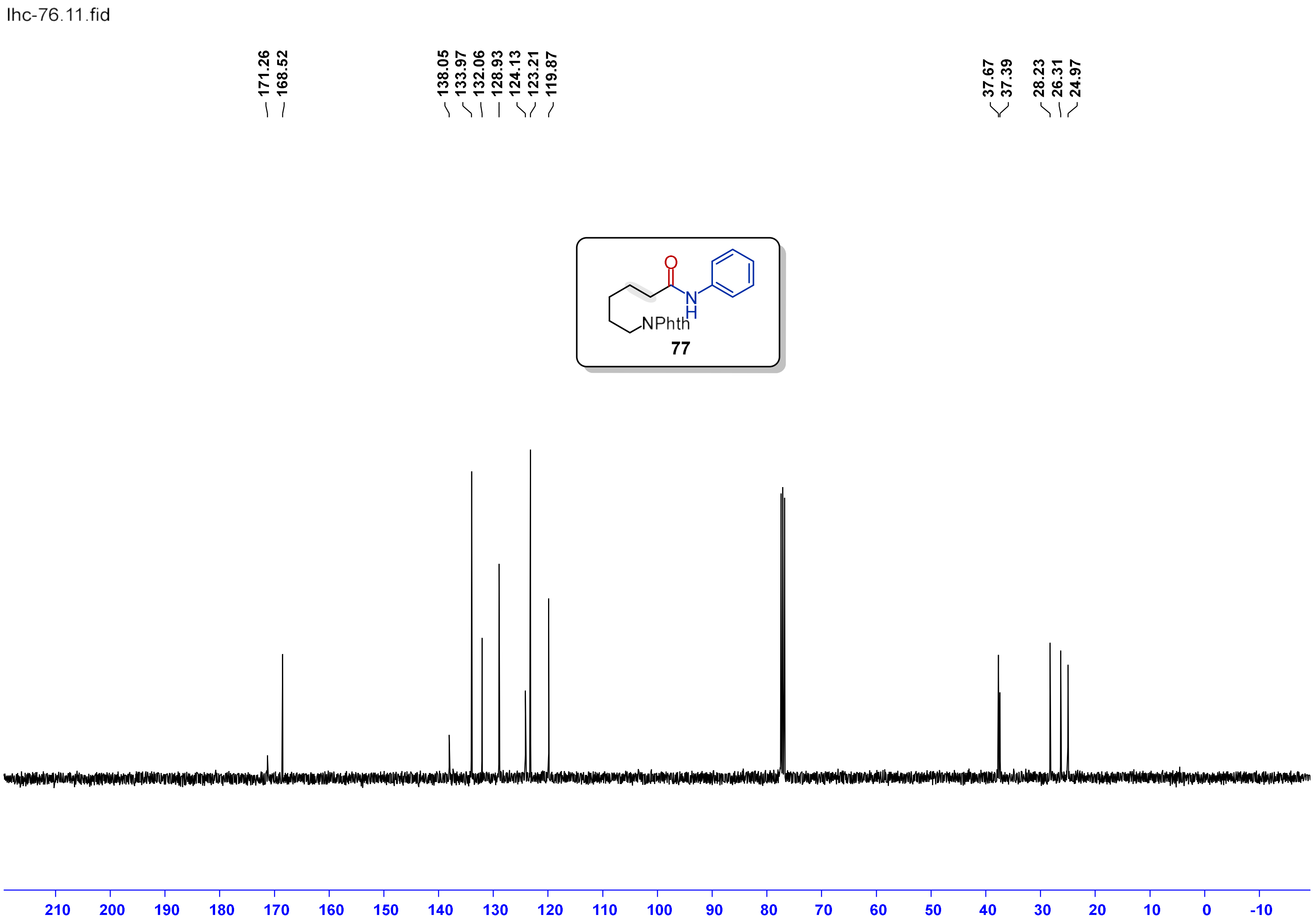

^{1}H NMR spectra for 78

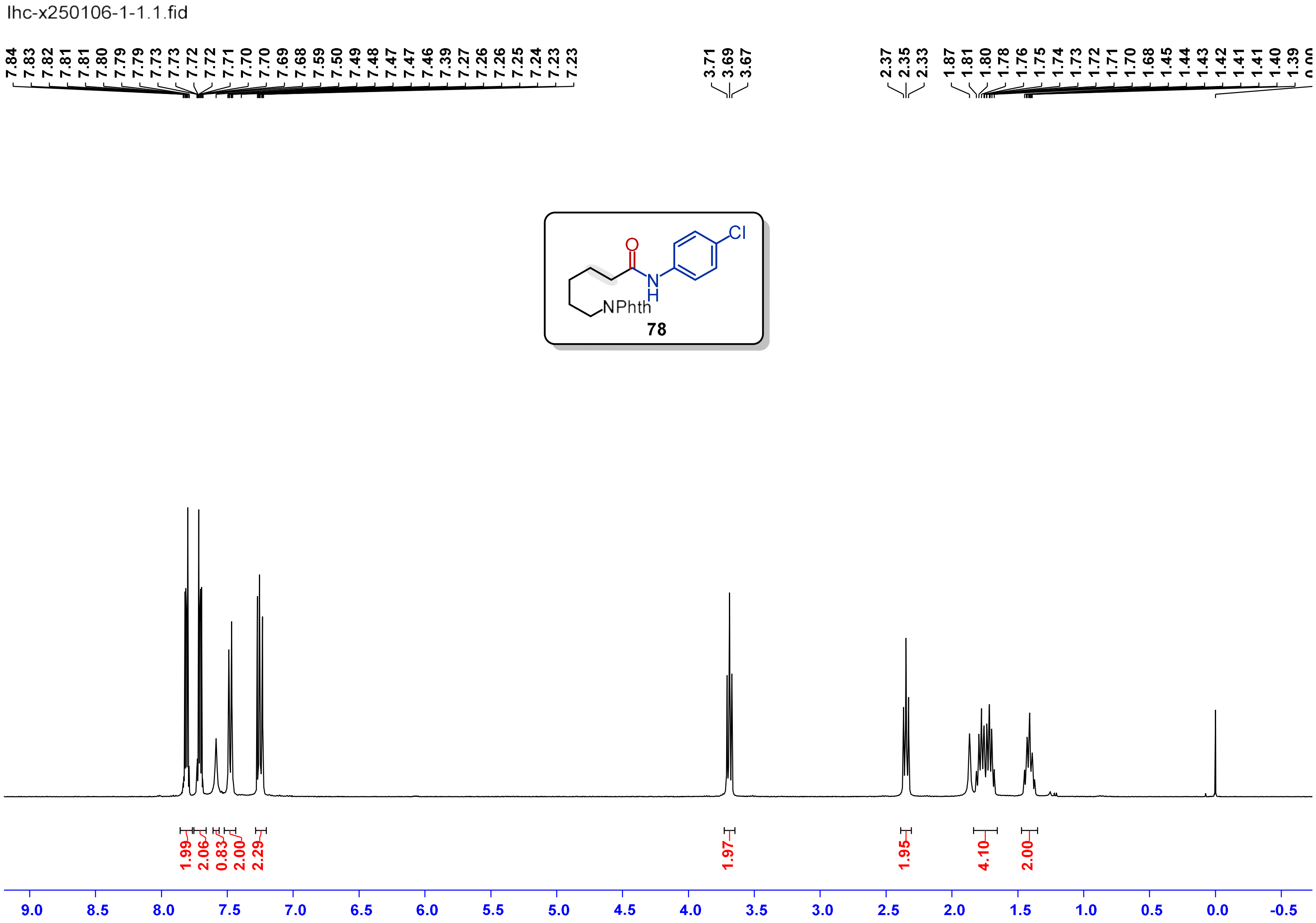

^{13}C NMR spectra for 78

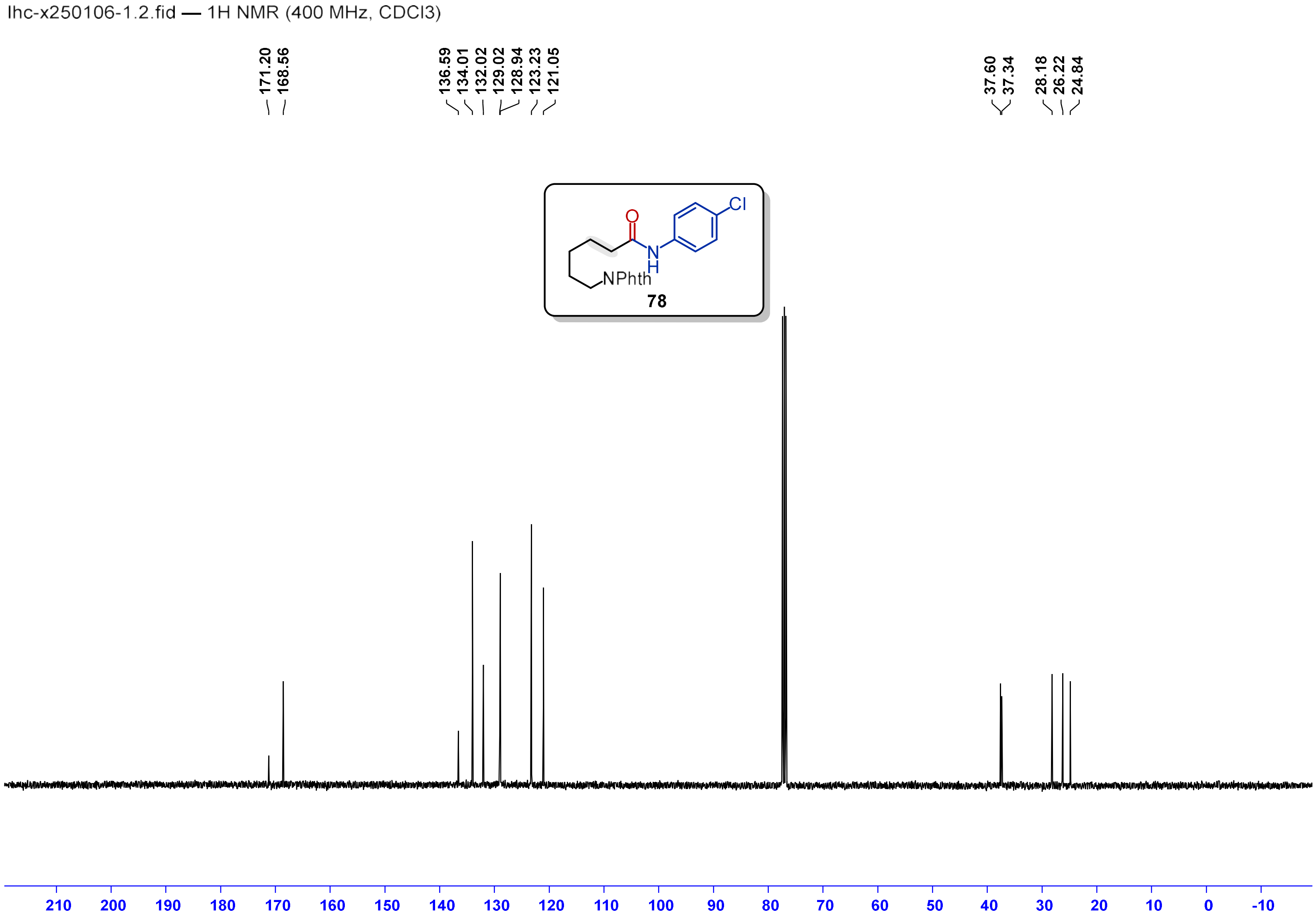

^{1}H NMR spectra for 79

lhc-x250106-2.3.fid — 1H NMR (400 MHz, CDCl3)

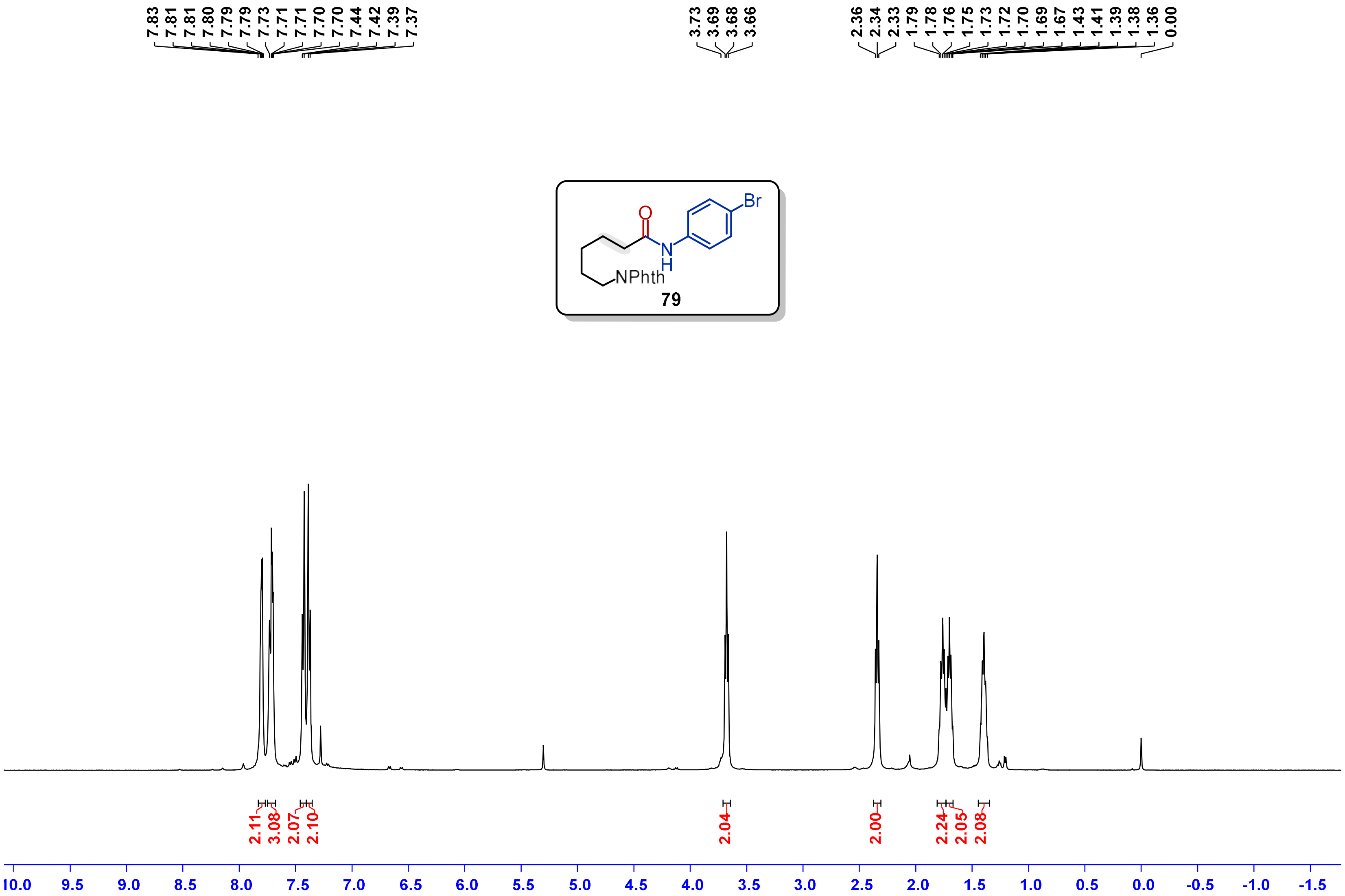

^{13}C NMR spectra for 79

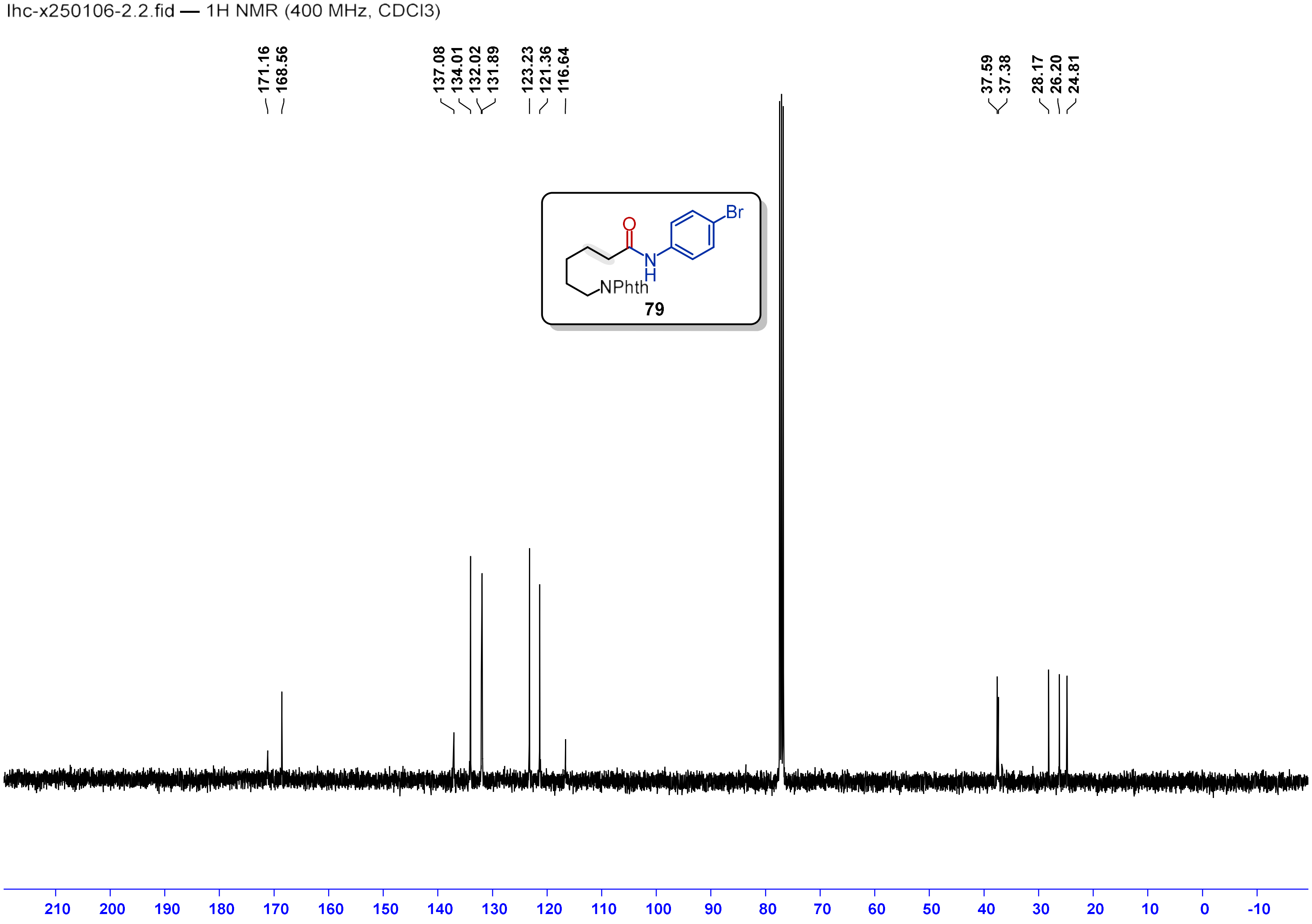

^{1}H NMR spectra for 80

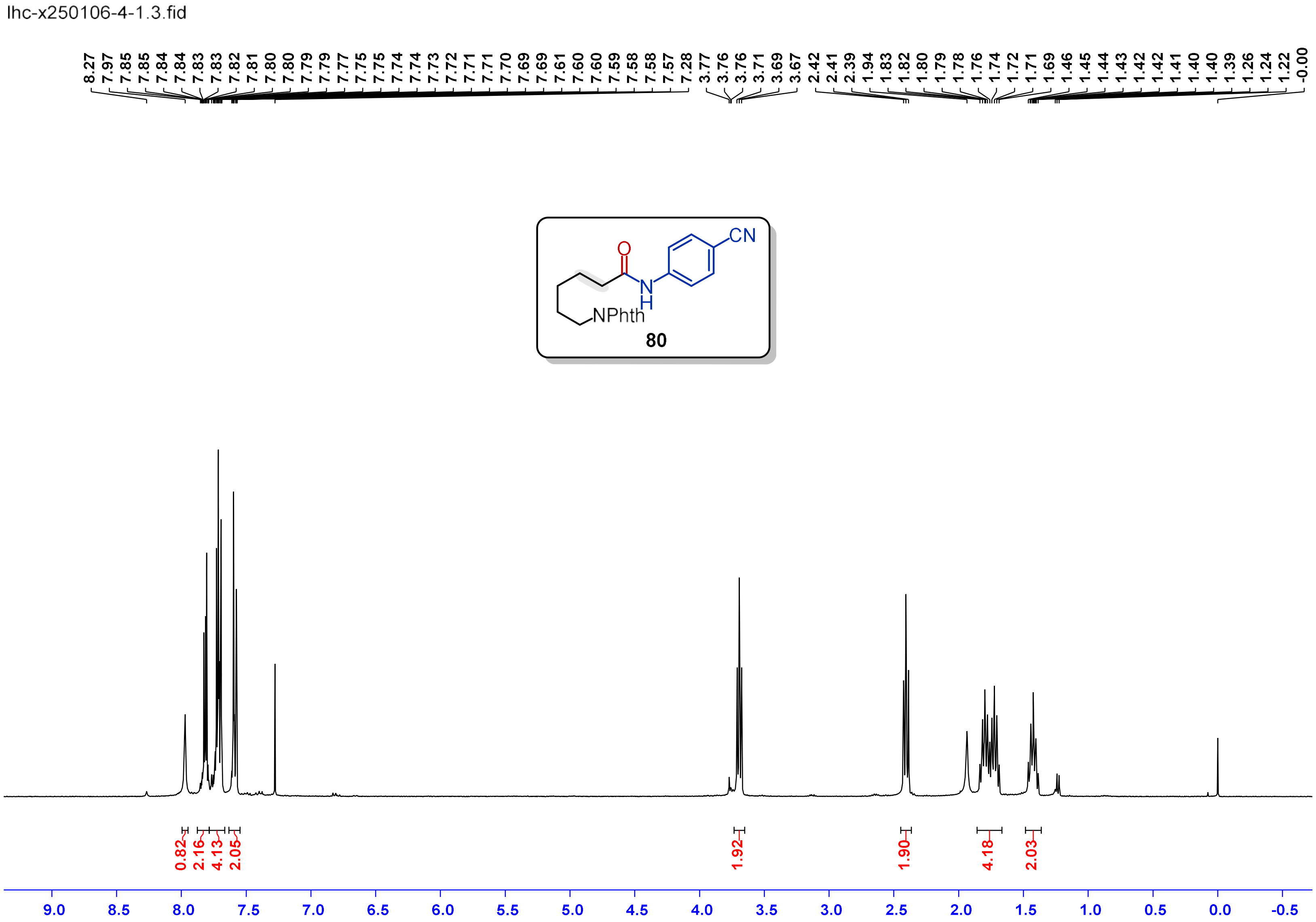

^{13}C NMR spectra for 80

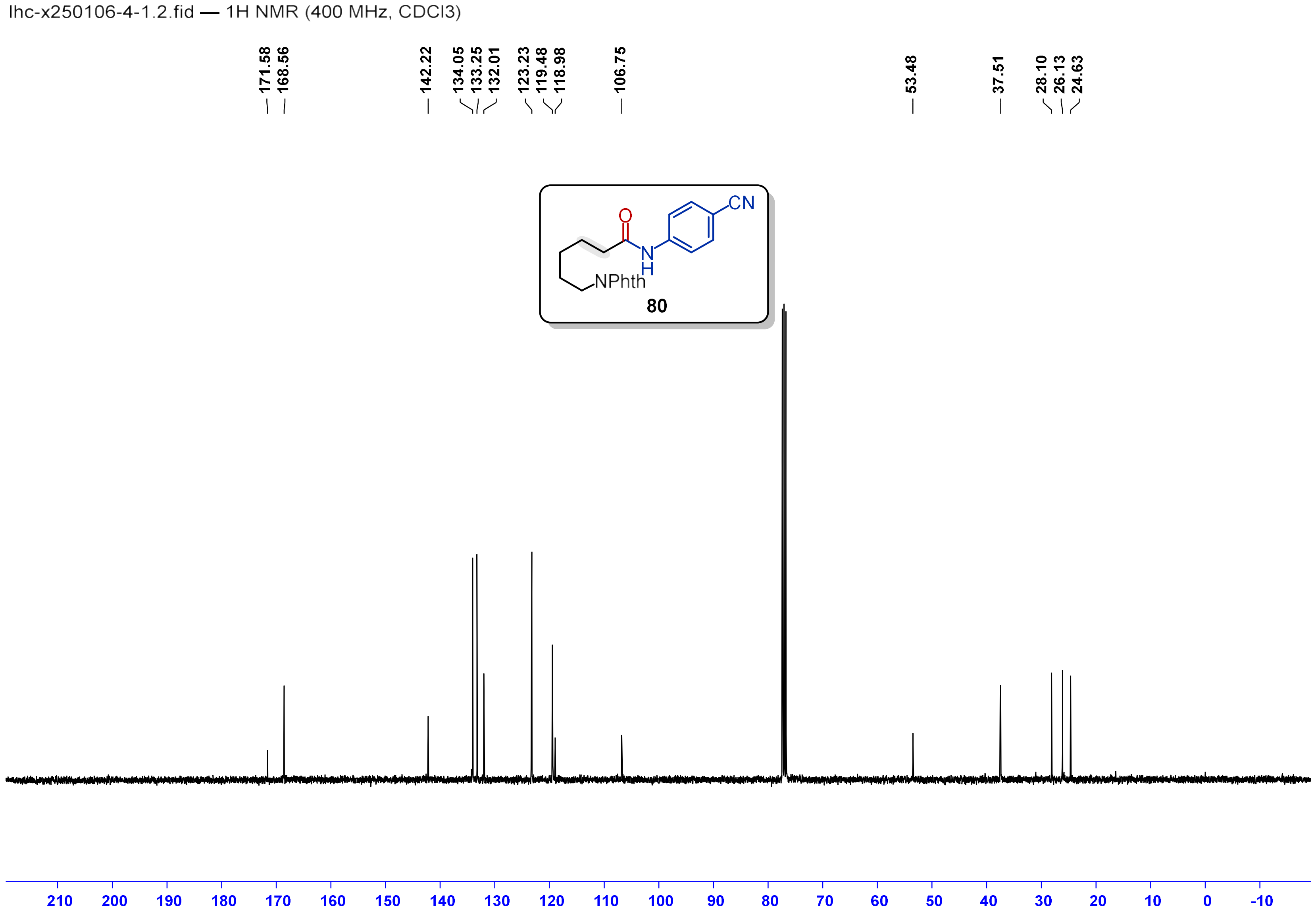

¹H NMR spectra for 81

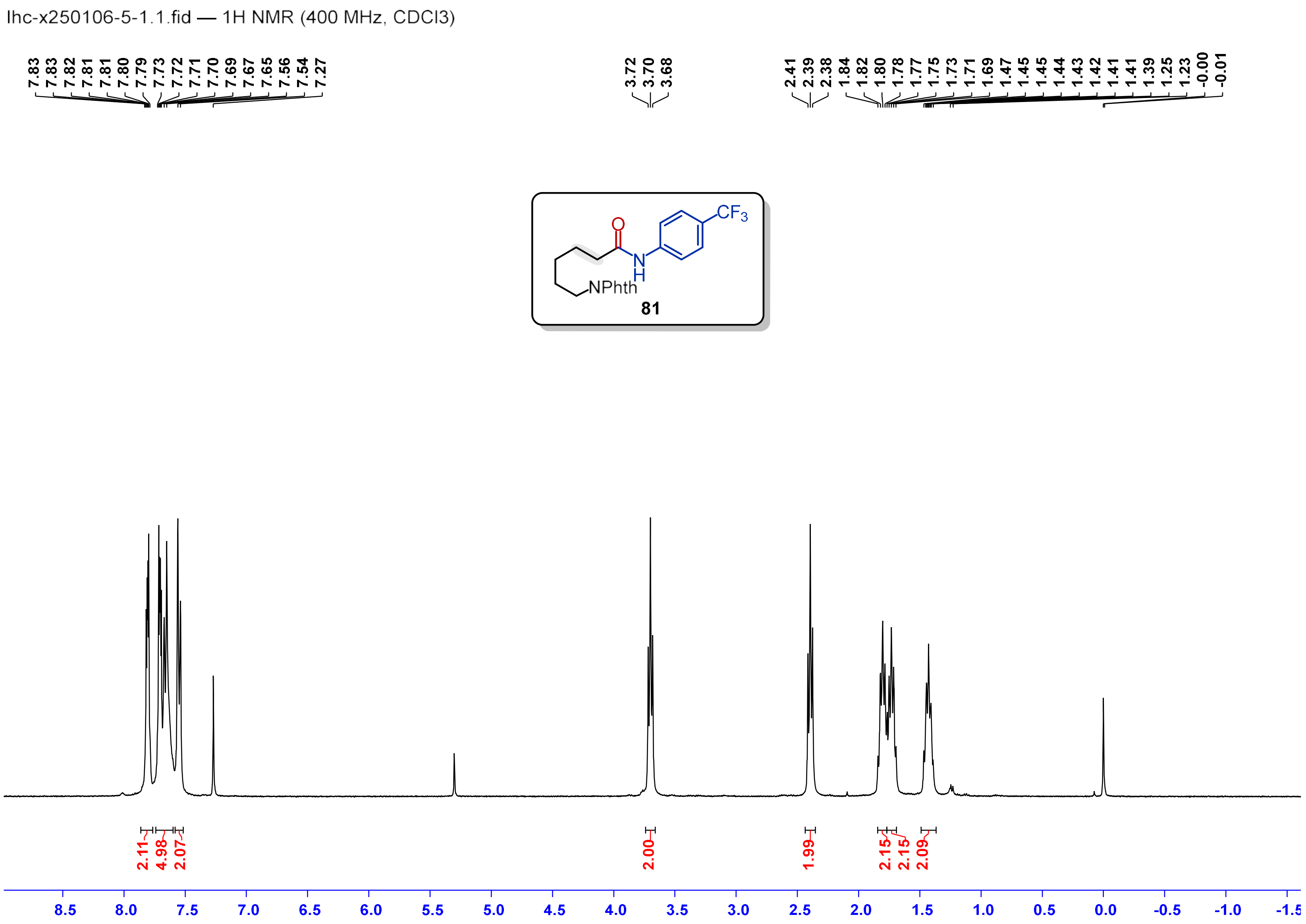

^{13}C NMR spectra for 81

lhc-x250106-5-1.2.fid — 1H NMR (400 MHz, CDCl3)

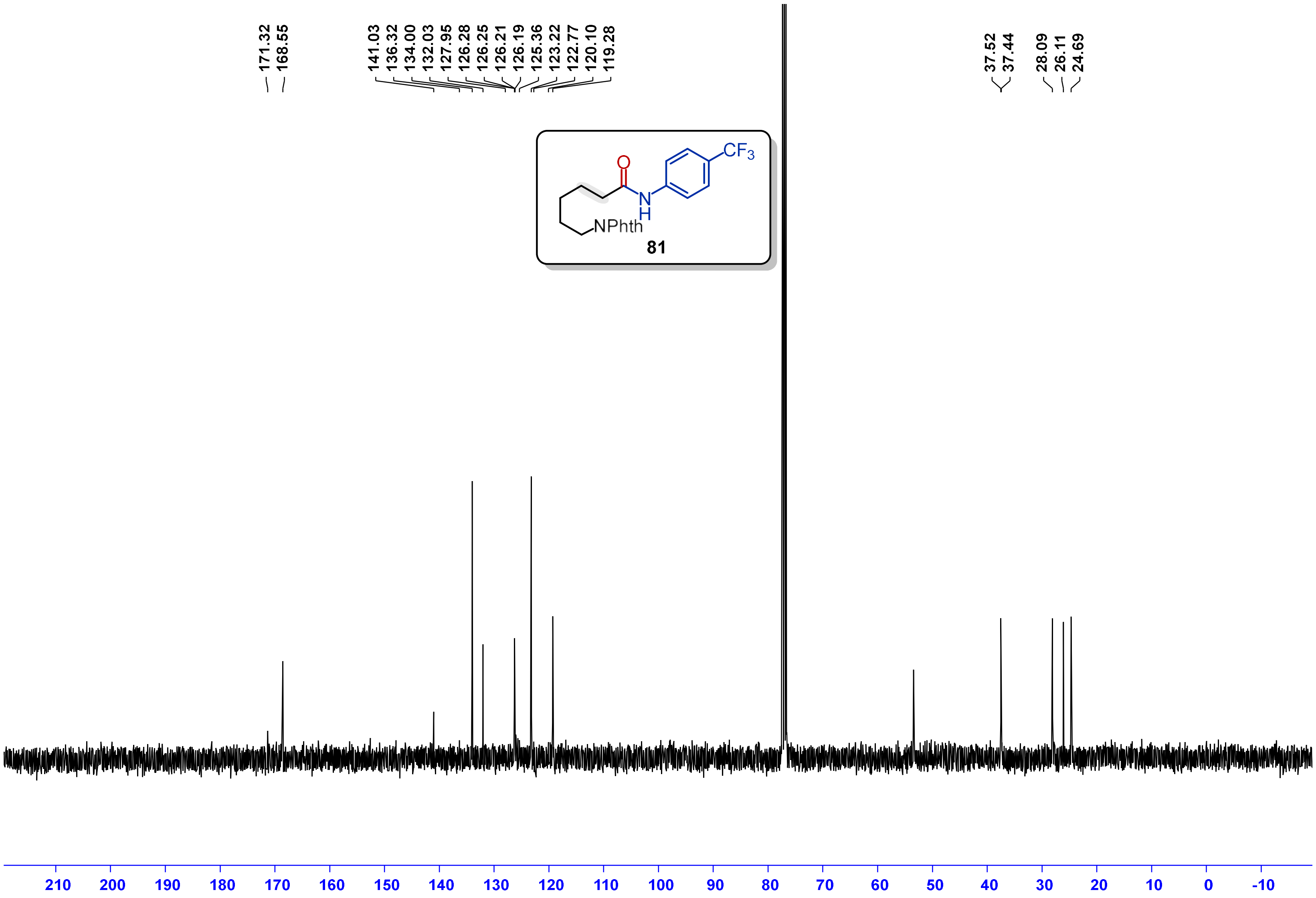

^{19}F NMR spectra for 81

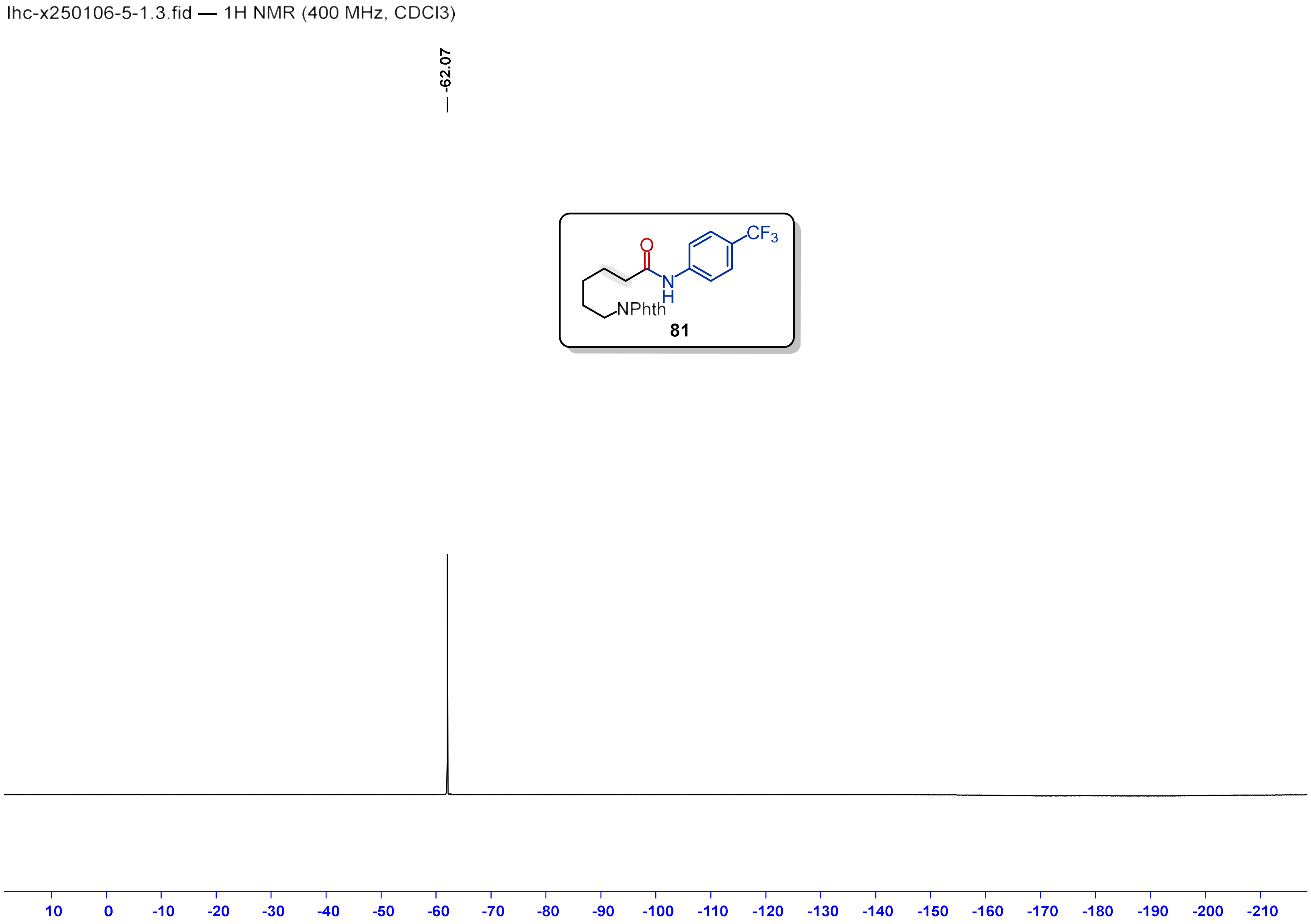

^{1}H NMR spectra for 82

lhc-x250106-3-1.1.fid — 1H NMR (400 MHz, CDCl3)

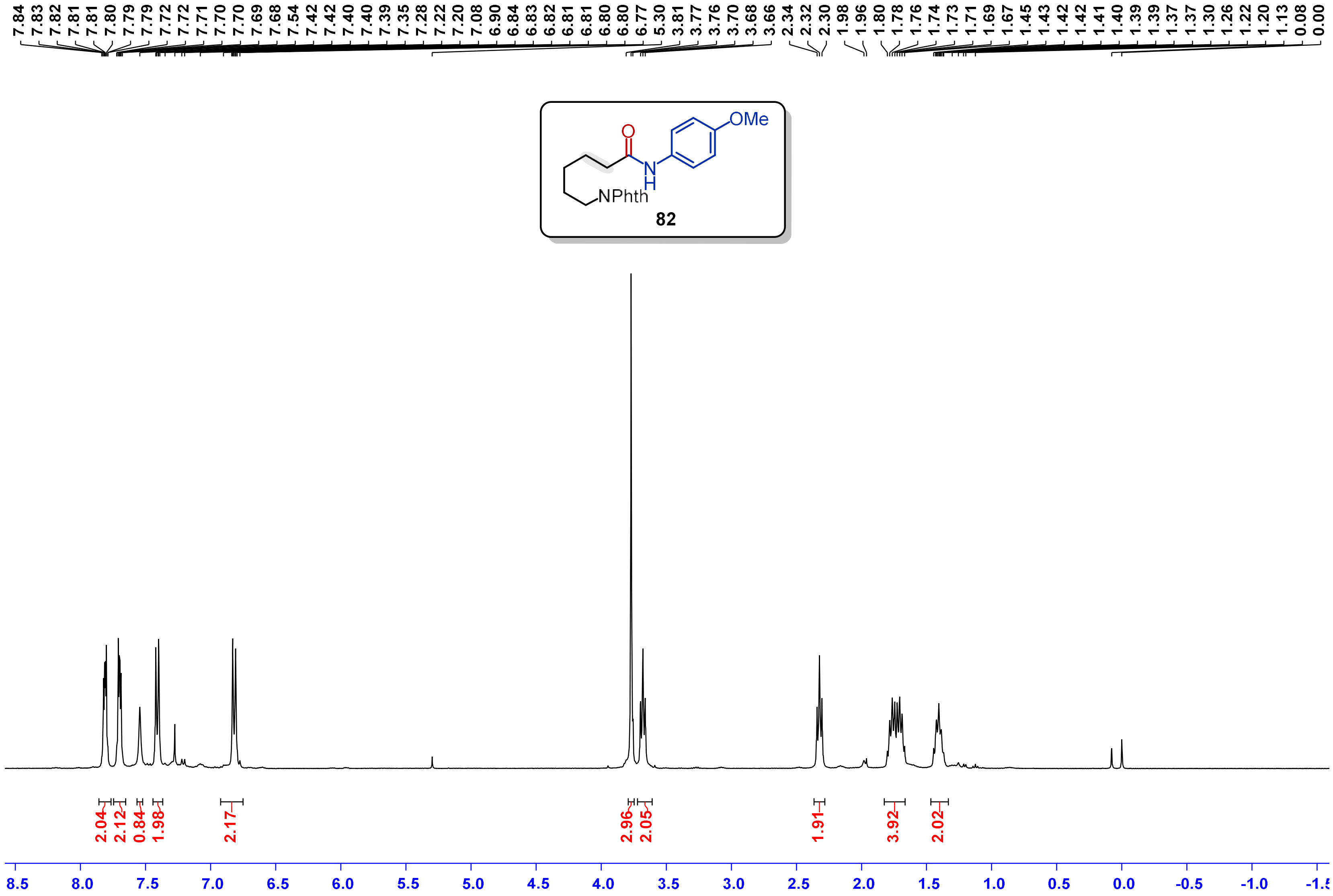

^{13}C NMR spectra for 82

lhc-x250106-3-1.2.fid — 1H NMR (400 MHz, CDCl3)

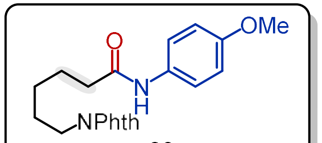

82

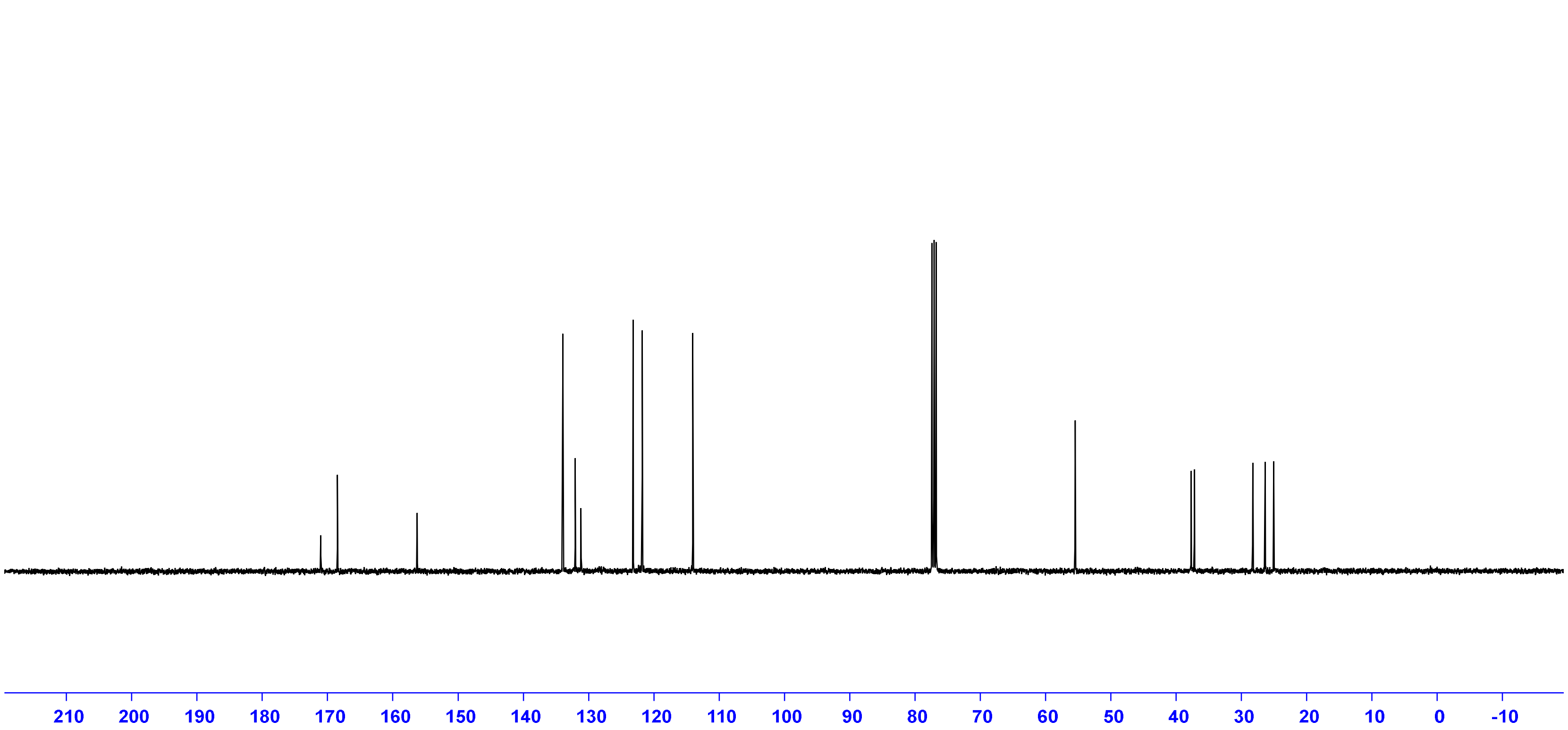

^{1}H NMR spectra for 83

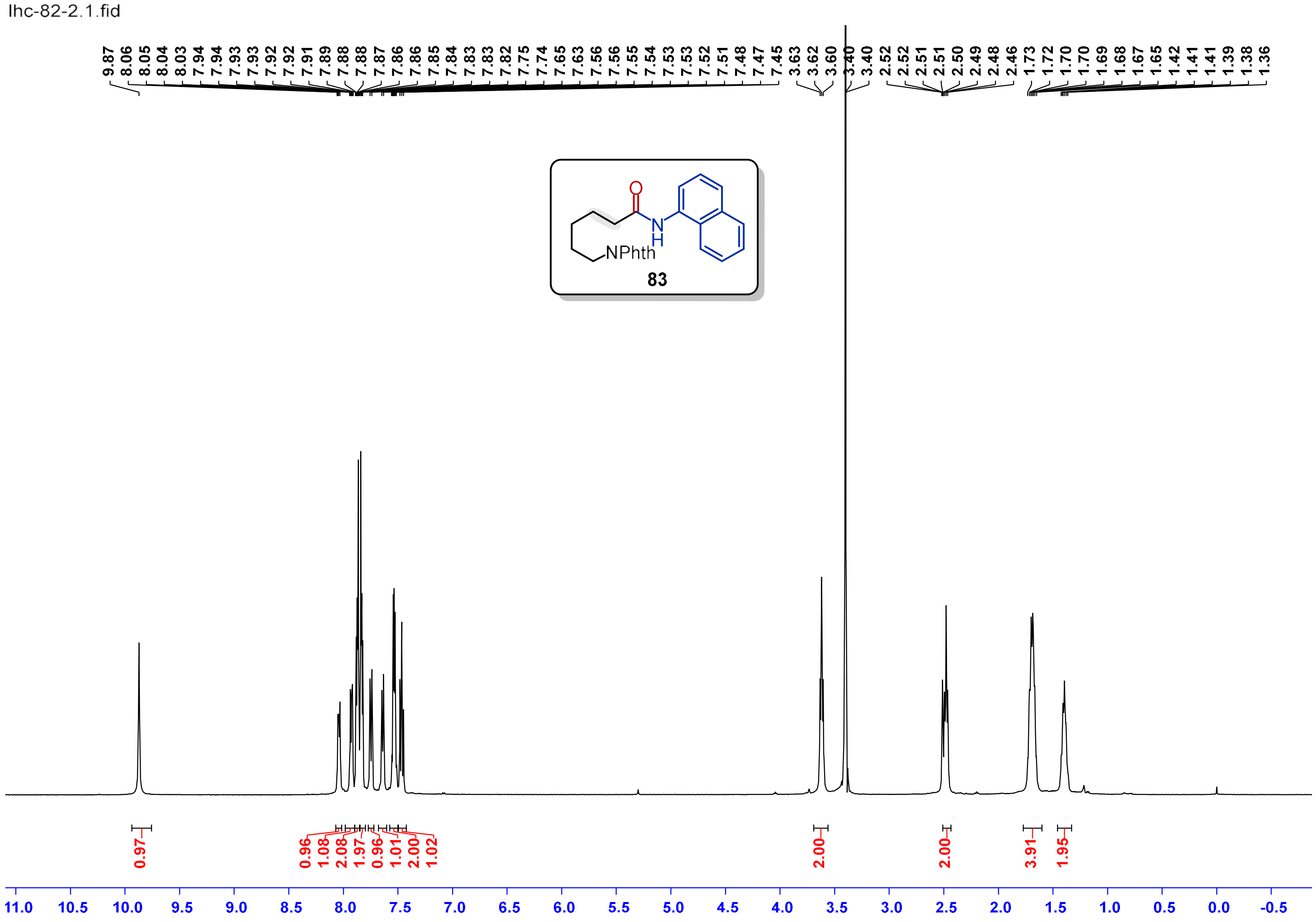

^{13}C NMR spectra for 83

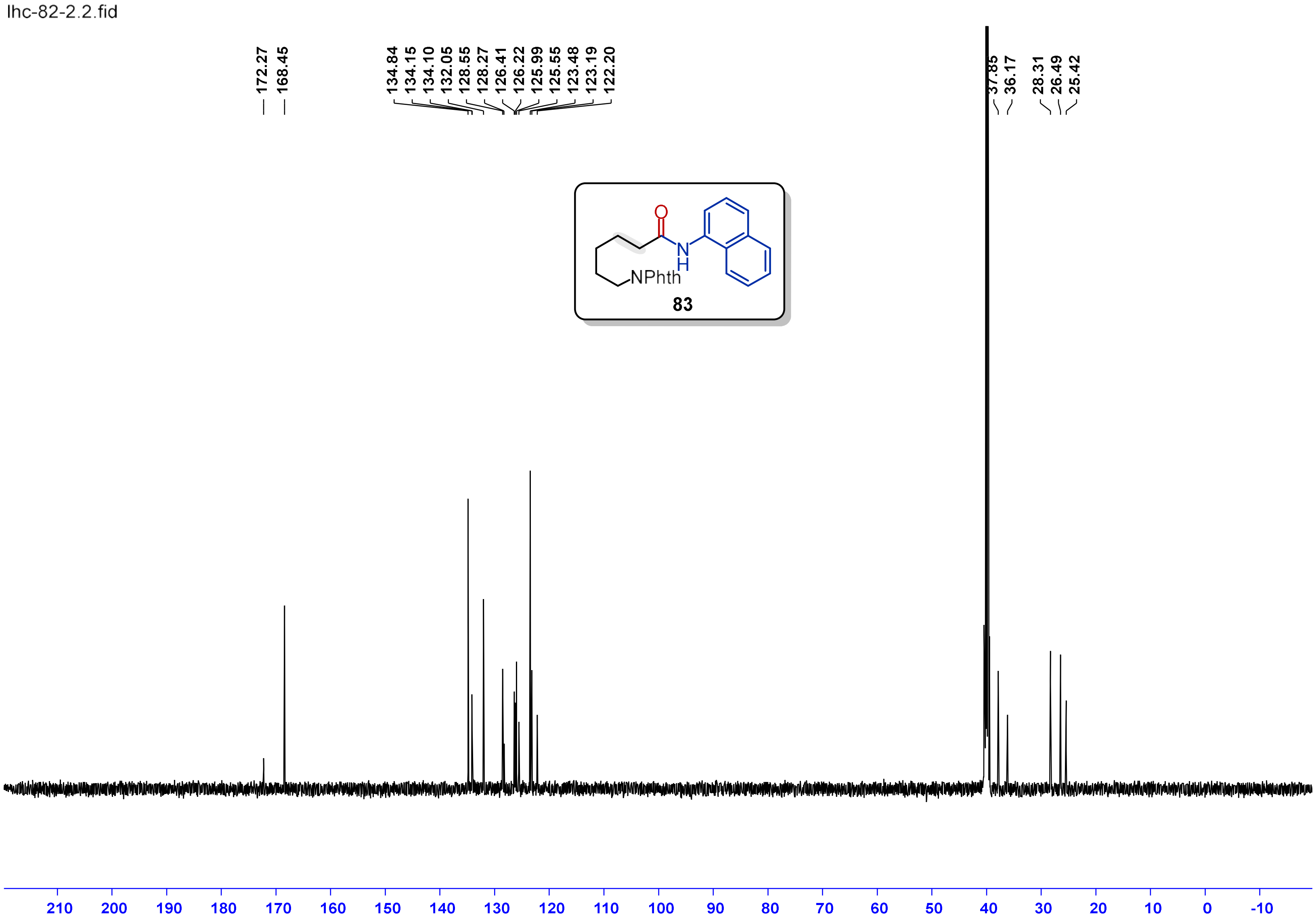

^{1}H NMR spectra for 84

lhc-84.10.fid

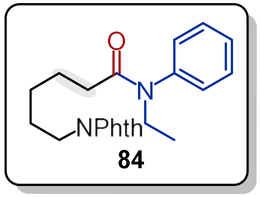

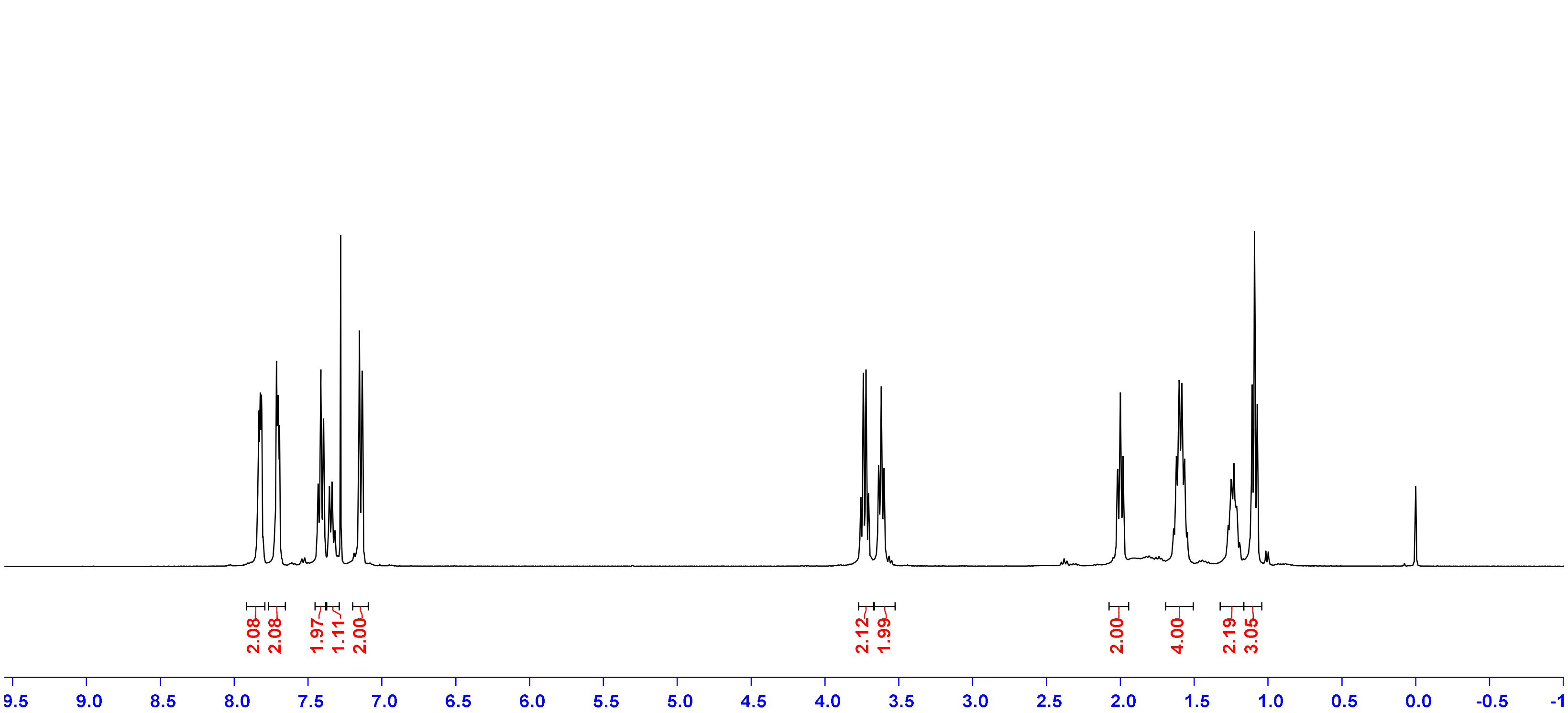

^{13}C NMR spectra for 84

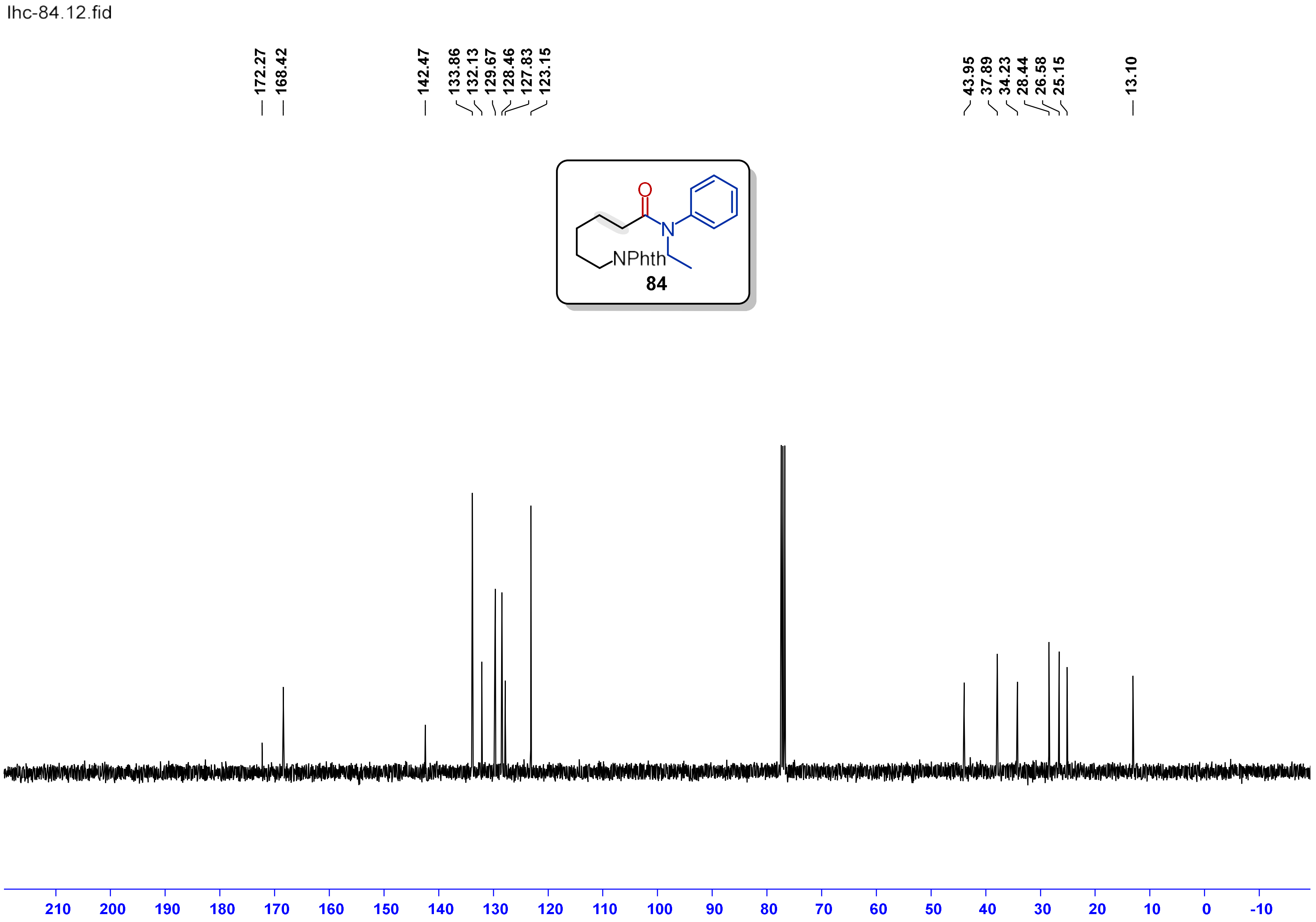

^{1}H NMR spectra for 85

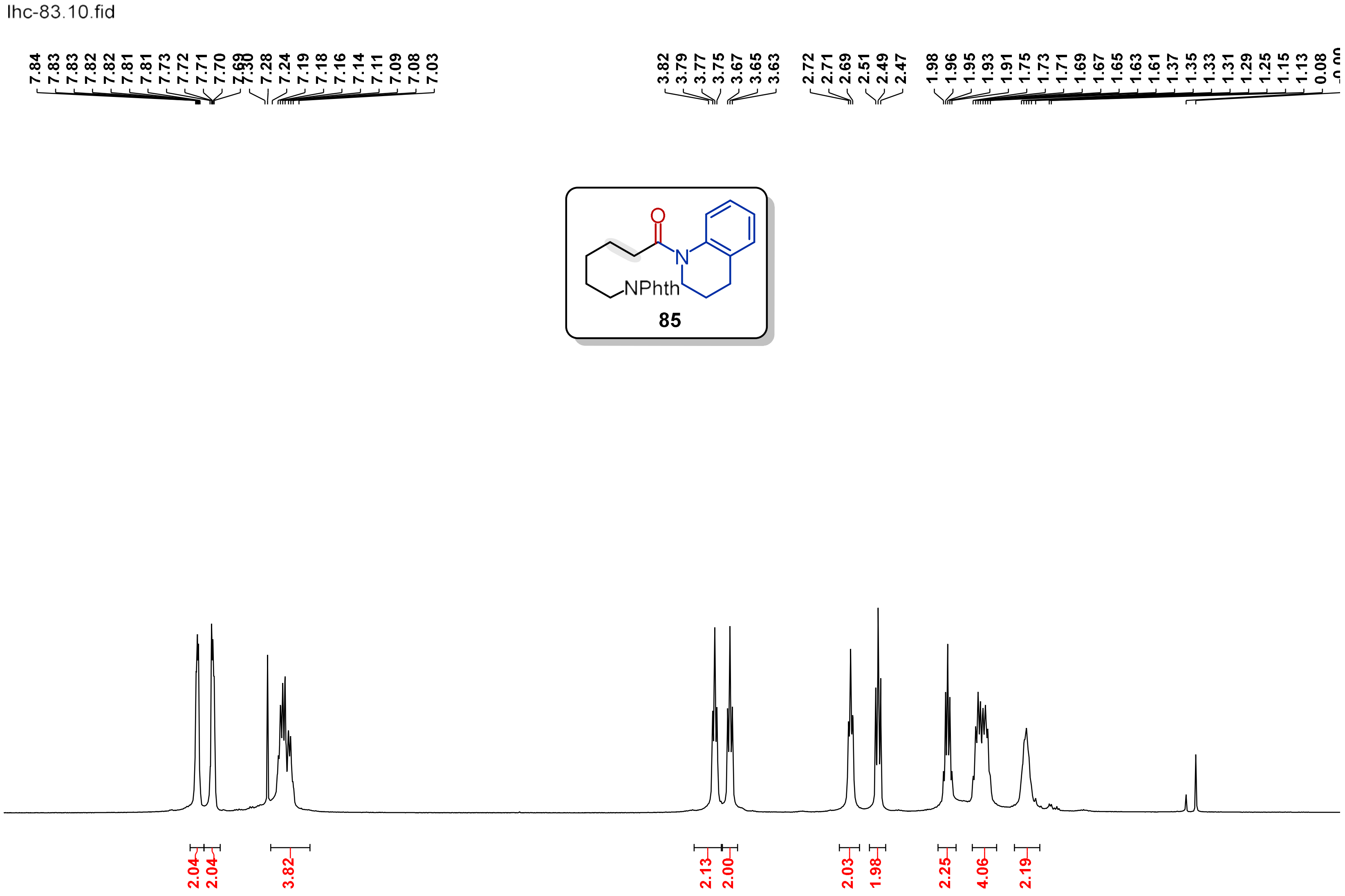

85

^{13}C NMR spectra for 85

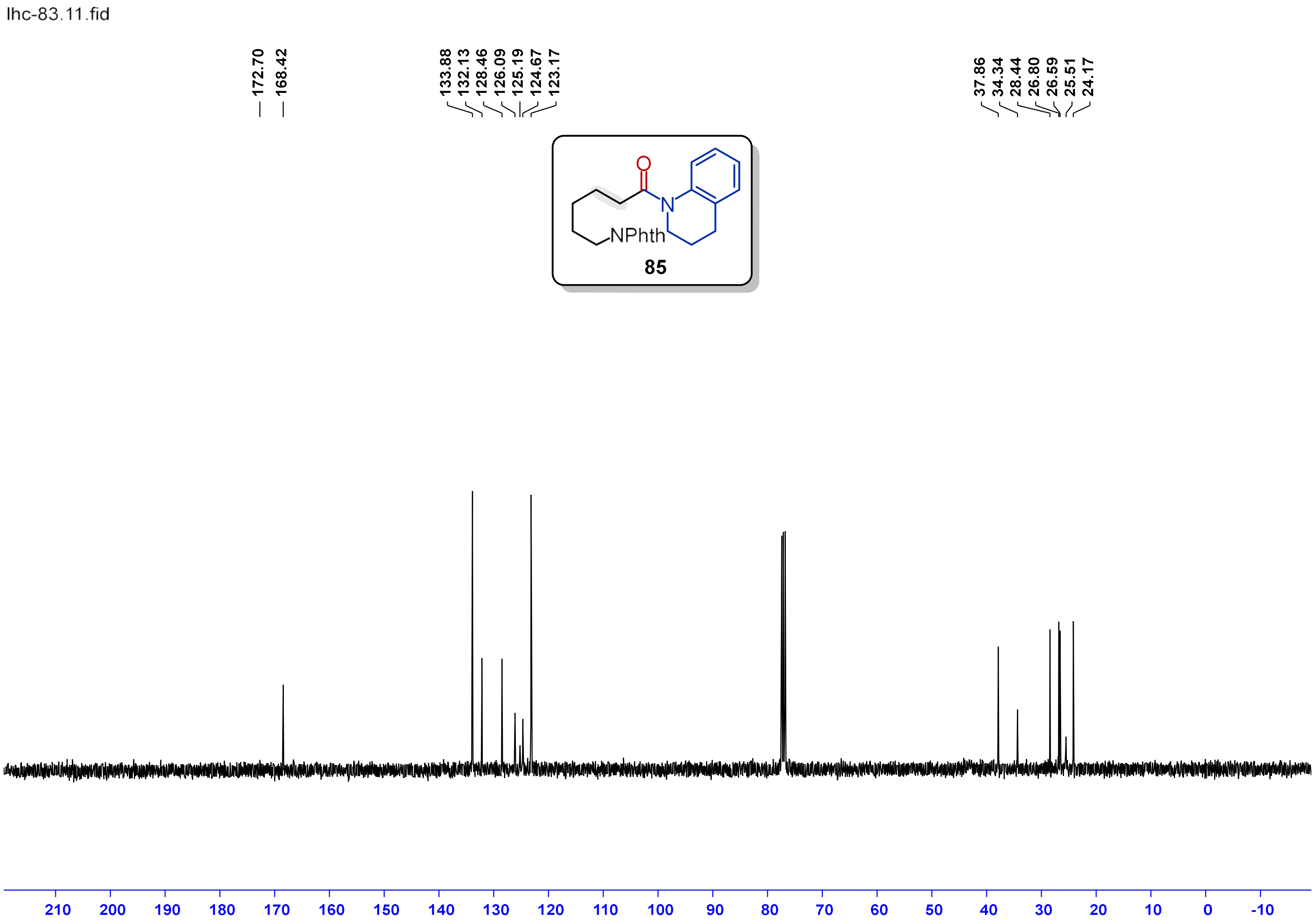

^{1}H NMR spectra for 86

lhc-x250107-1.3.fid — 1H NMR (400 MHz, CDCl3)

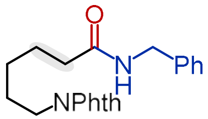

86

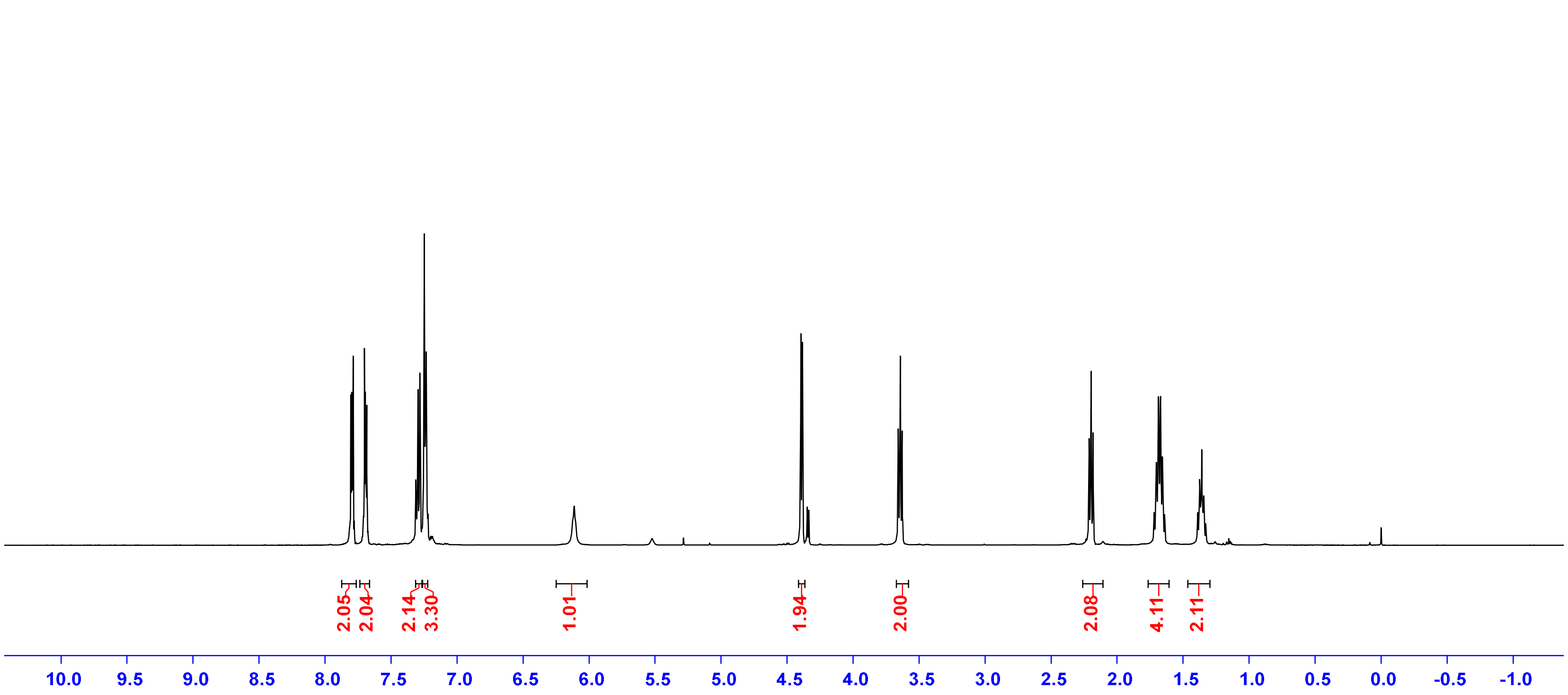

^{13}C NMR spectra for 86

lhc-x250107-1.4.fid — 1H NMR (400 MHz, CDCl3)

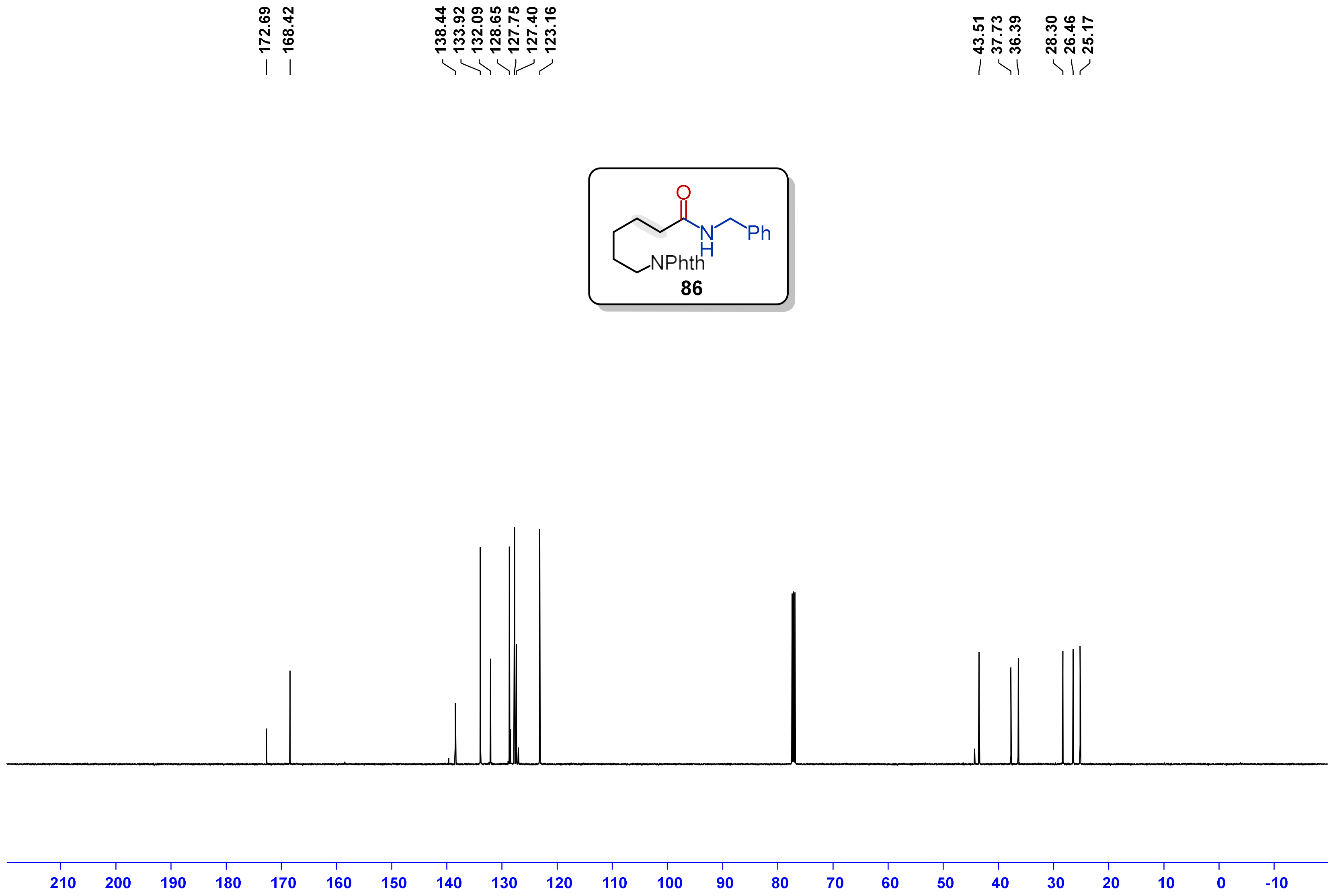

^{1}H NMR spectra for 87

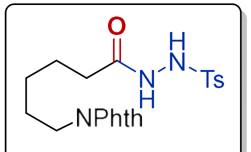

87

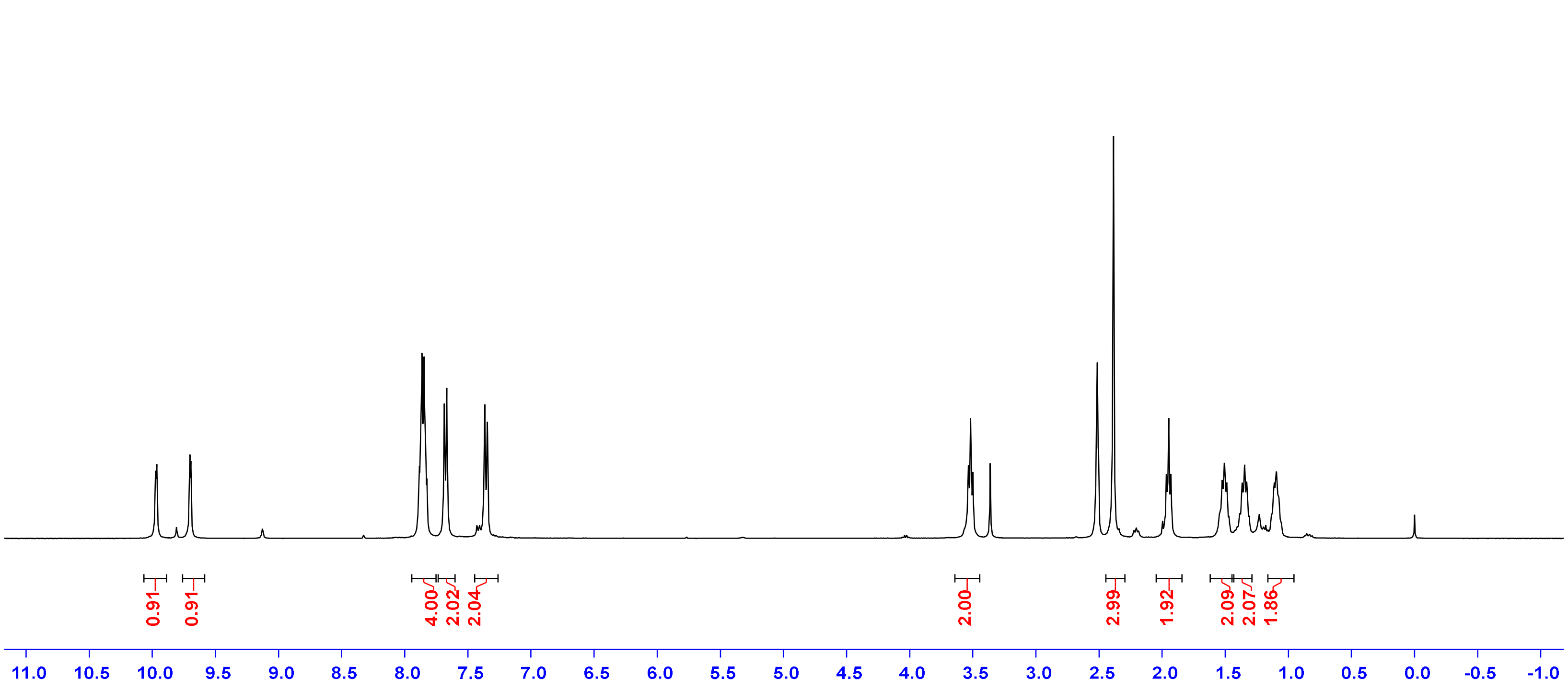

^{13}C NMR spectra for 87

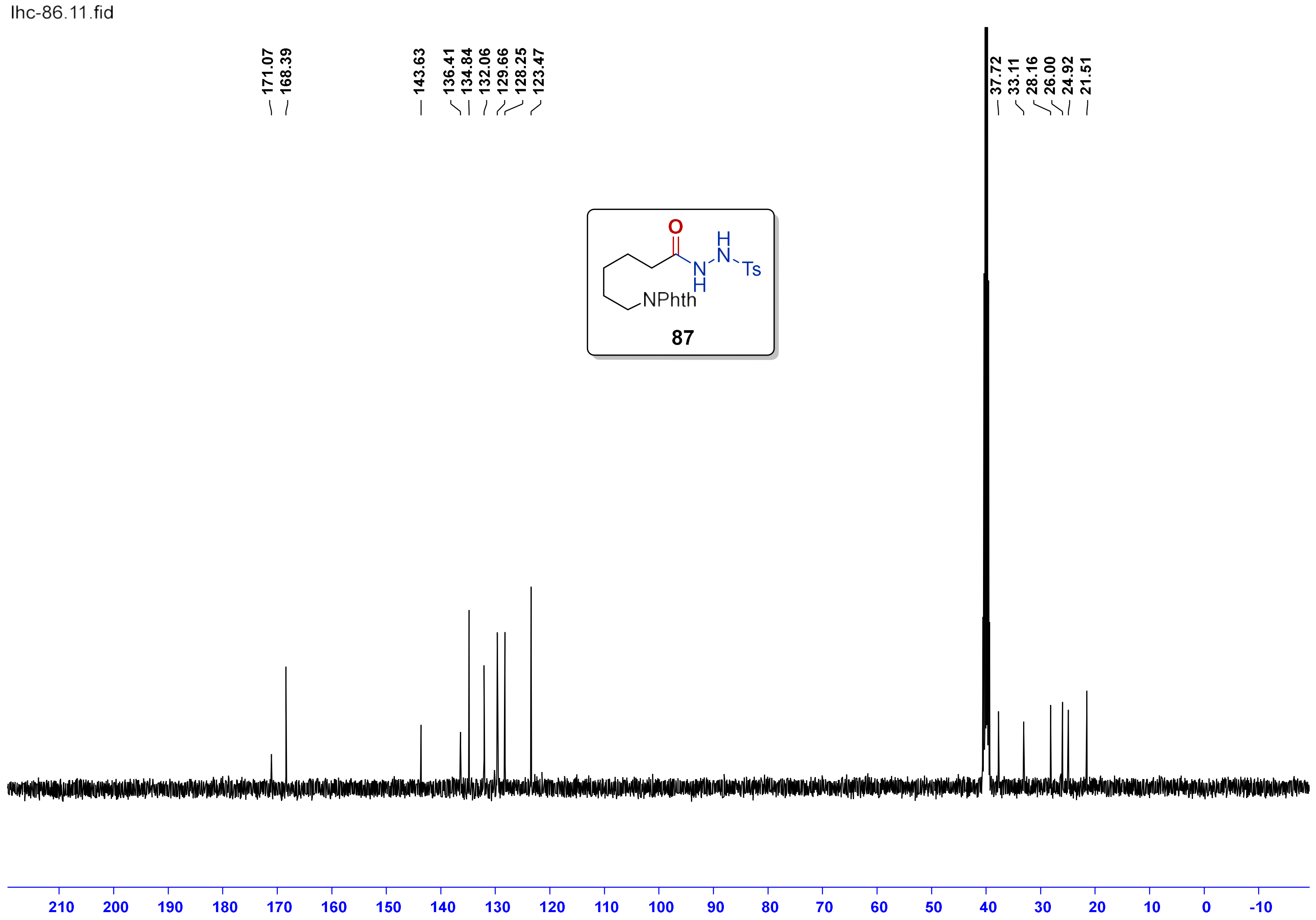

^{1}H NMR spectra for 88

lhc-x250309-7.1.fid — 1H NMR (400 MHz, CDCl3)

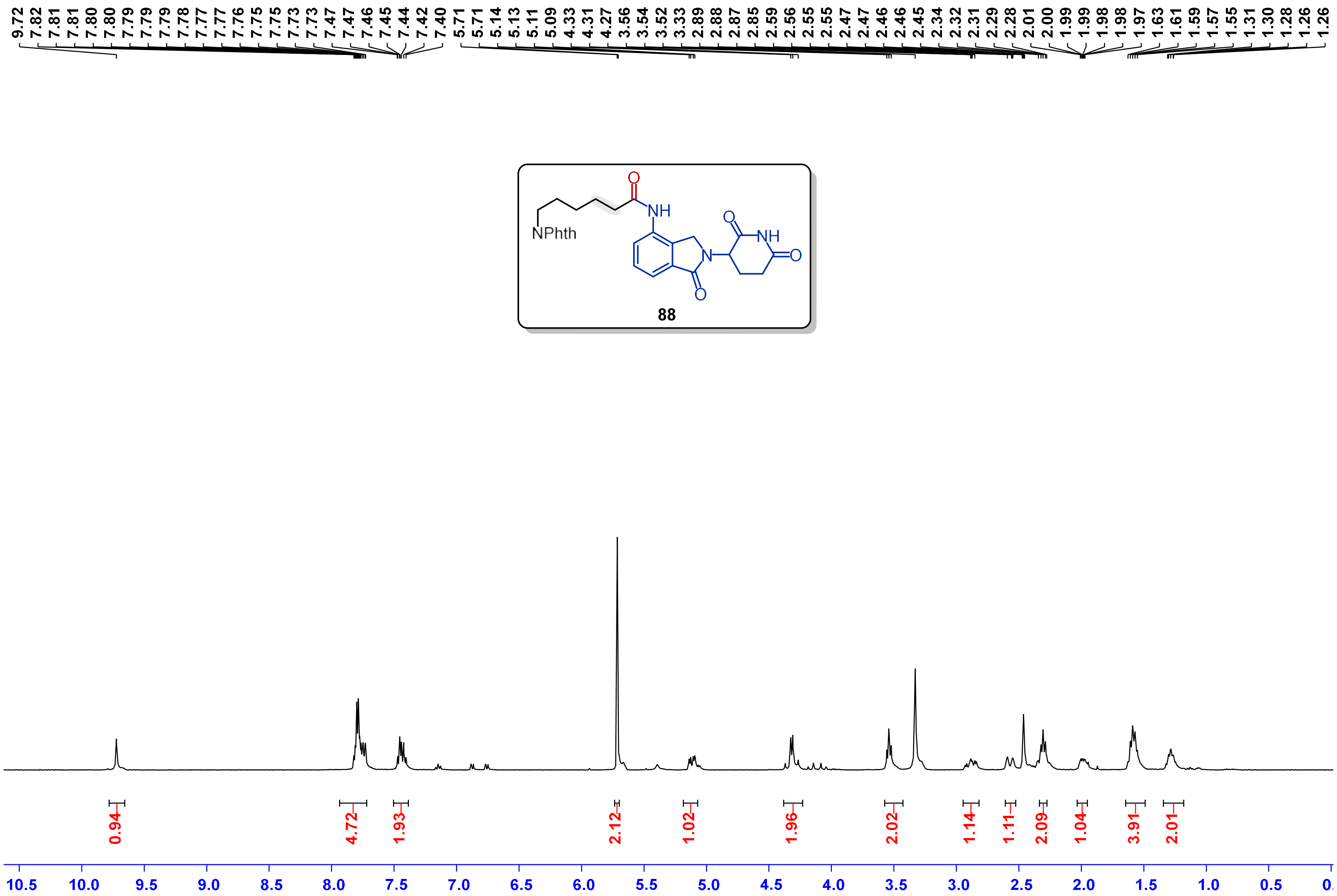

^{13}C NMR spectra for 88

lhc-x250309-7.2.fid — 1H NMR (400 MHz, CDCl3)

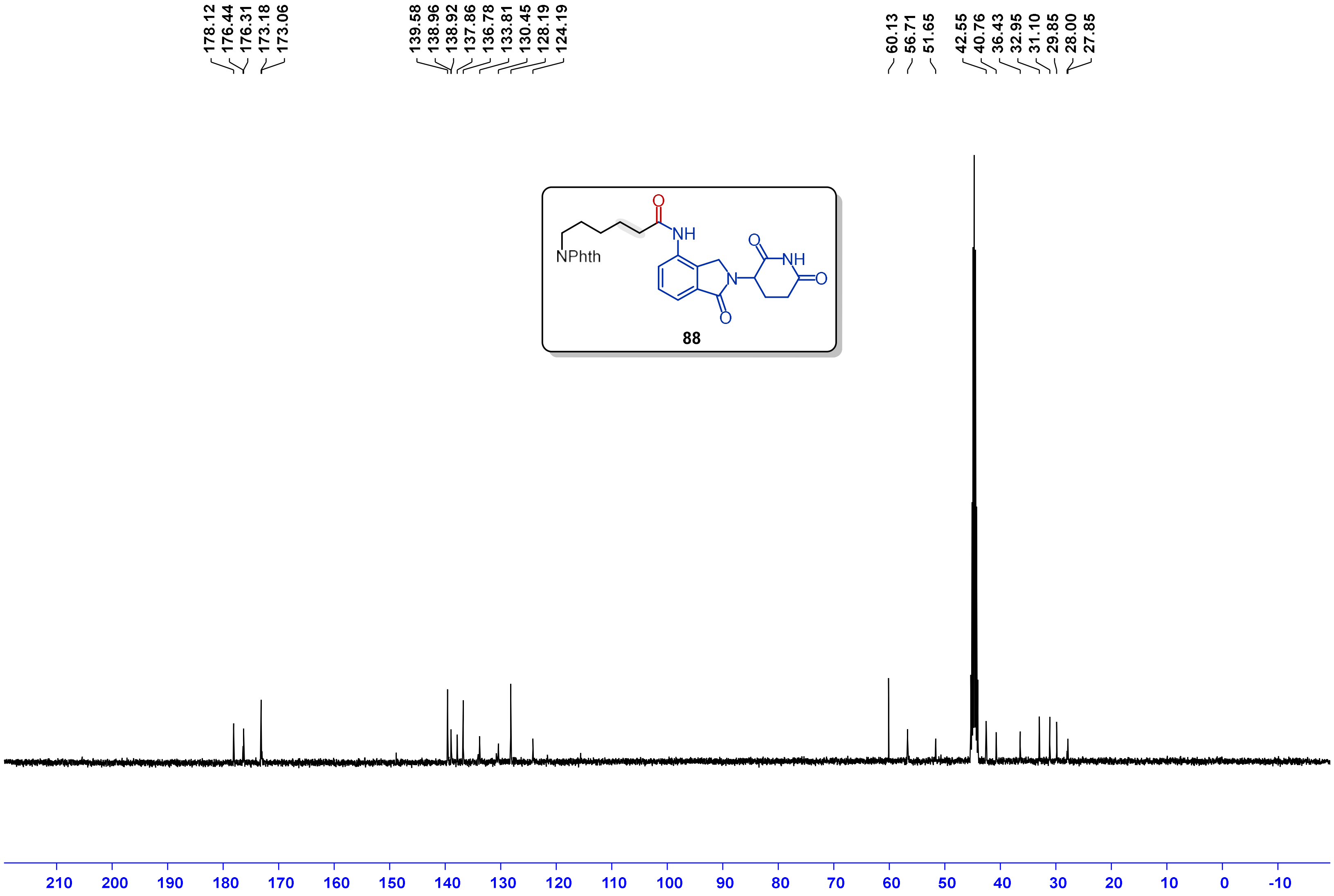

^{1}H NMR spectra for 89

lhc-x250518-7.1.fid — 1H NMR (400 MHz, CDCl3)

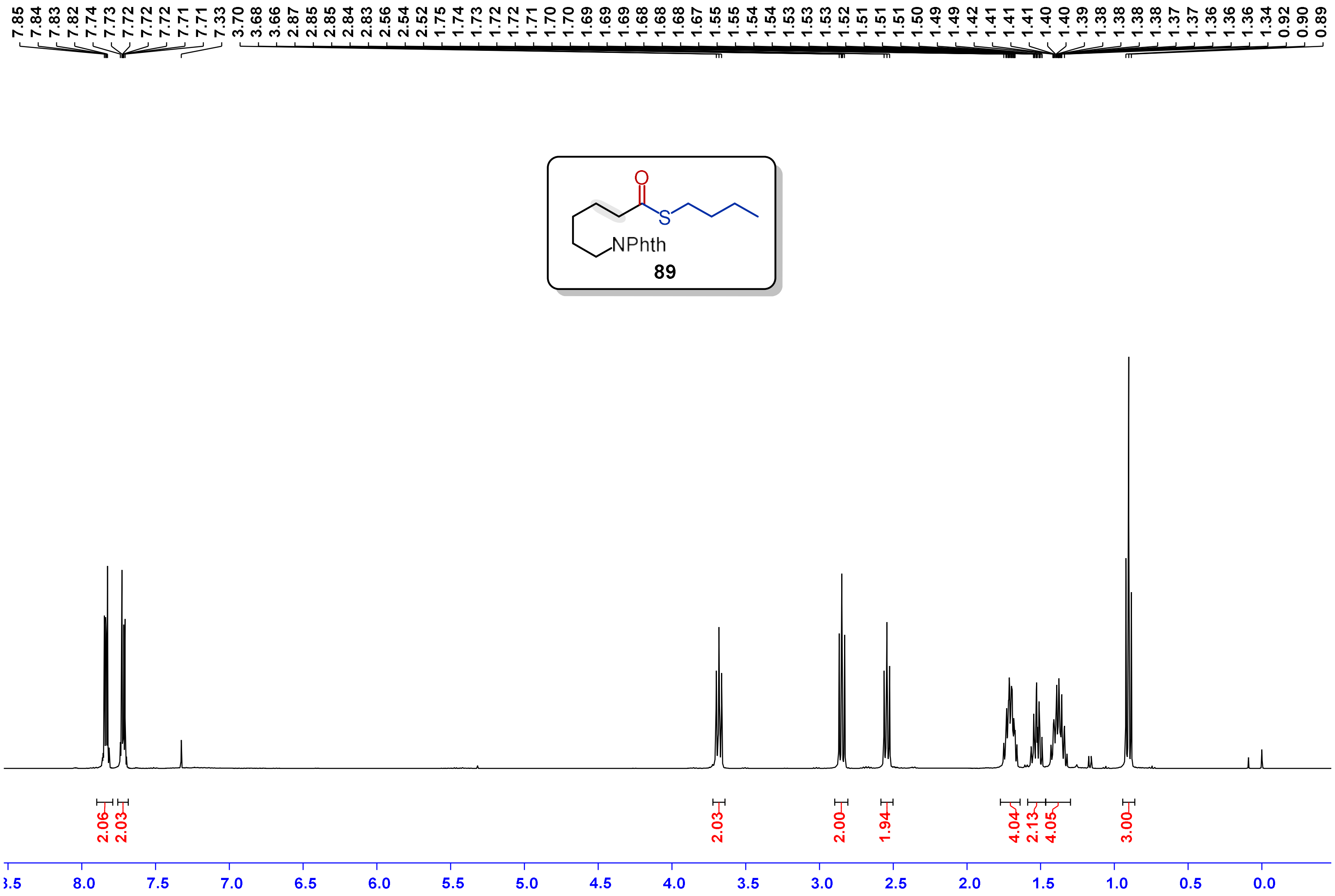

^{13}C NMR spectra for 89

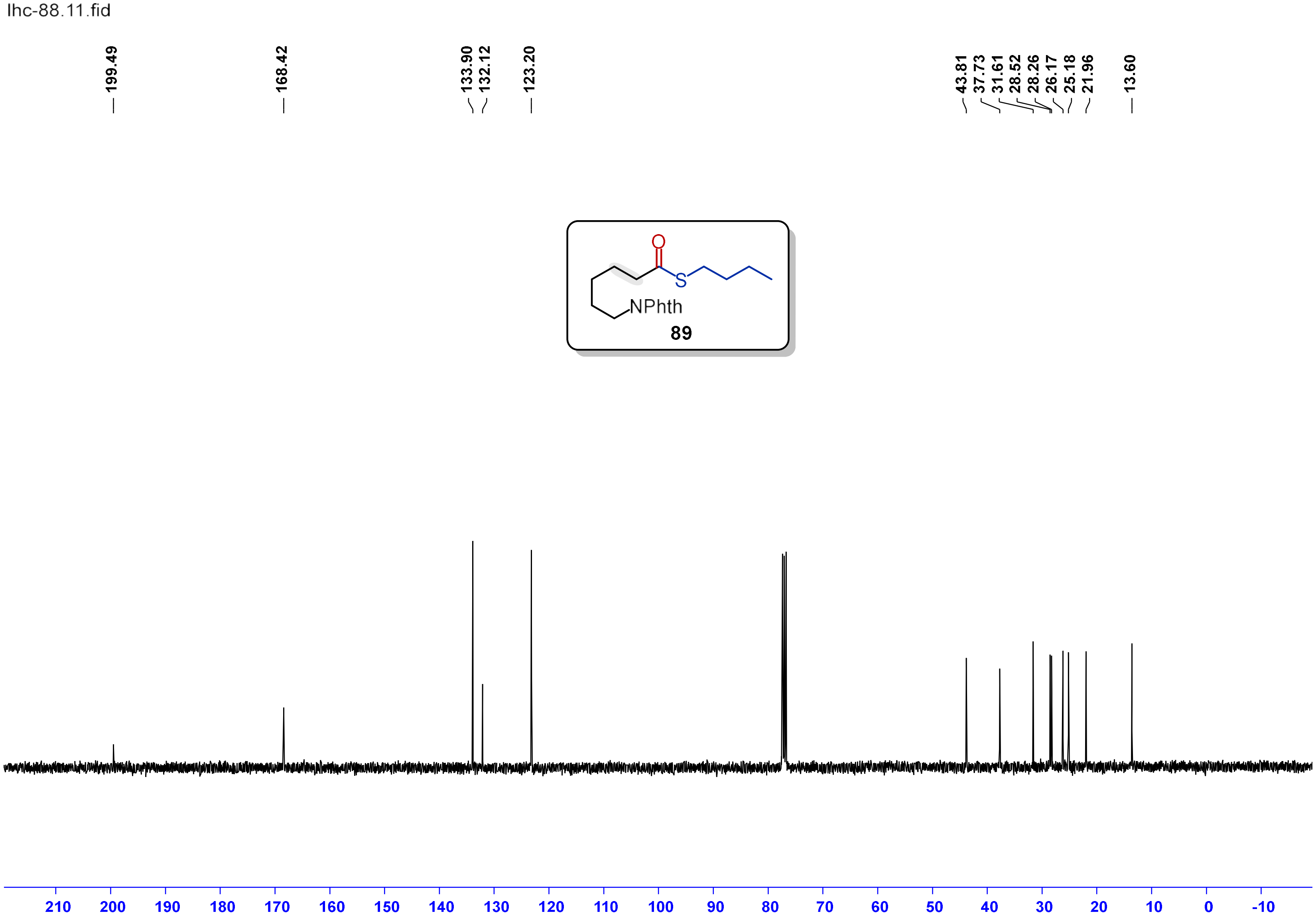

^{1}H NMR spectra for 90

lhc-x250518-8.1.fid — 1H NMR (400 MHz, CDCl3)

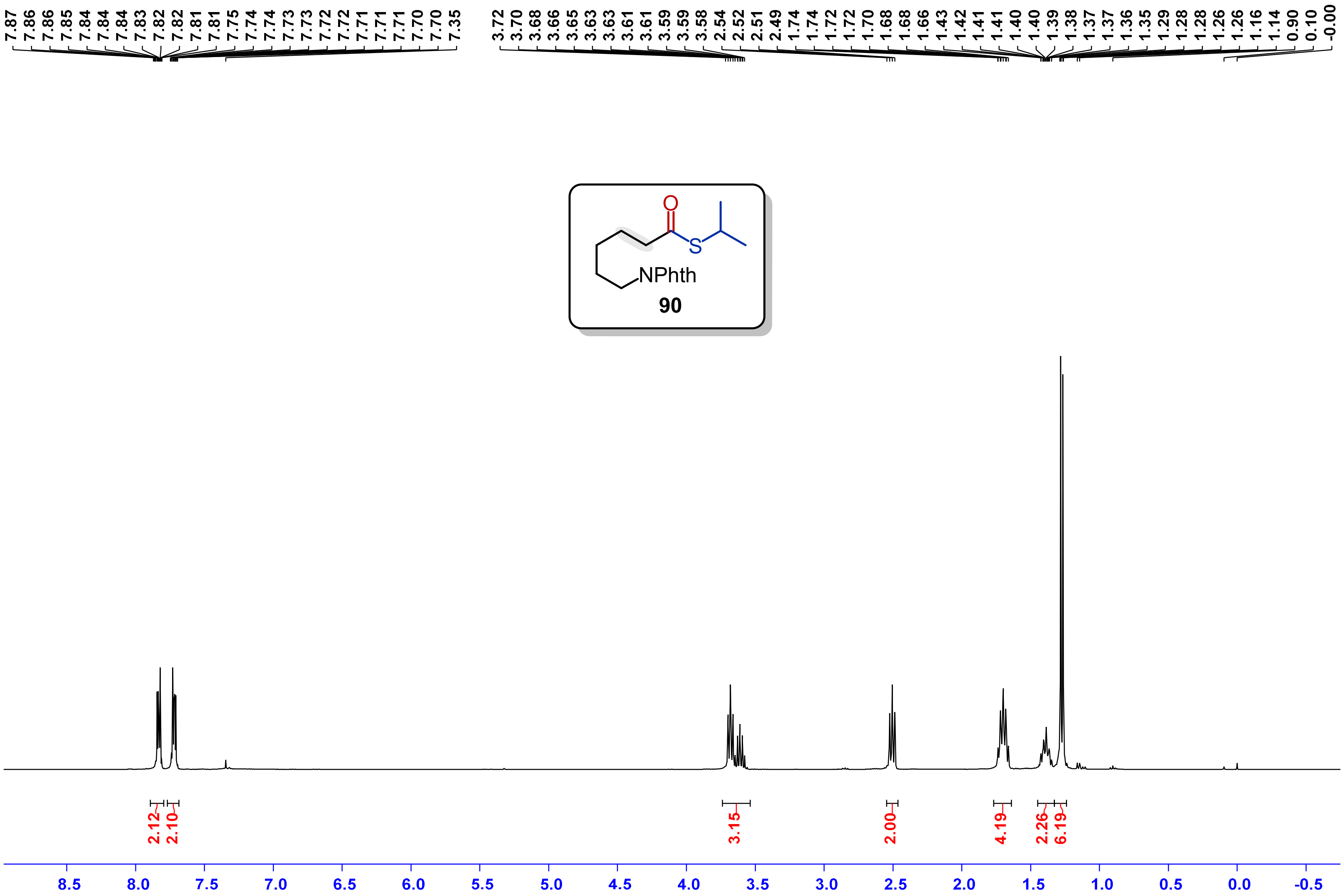

^{13}C NMR spectra for 90

lhc-x250518-8.2.fid — 1H NMR (400 MHz, CDCl3)

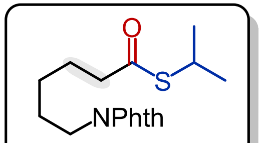

90

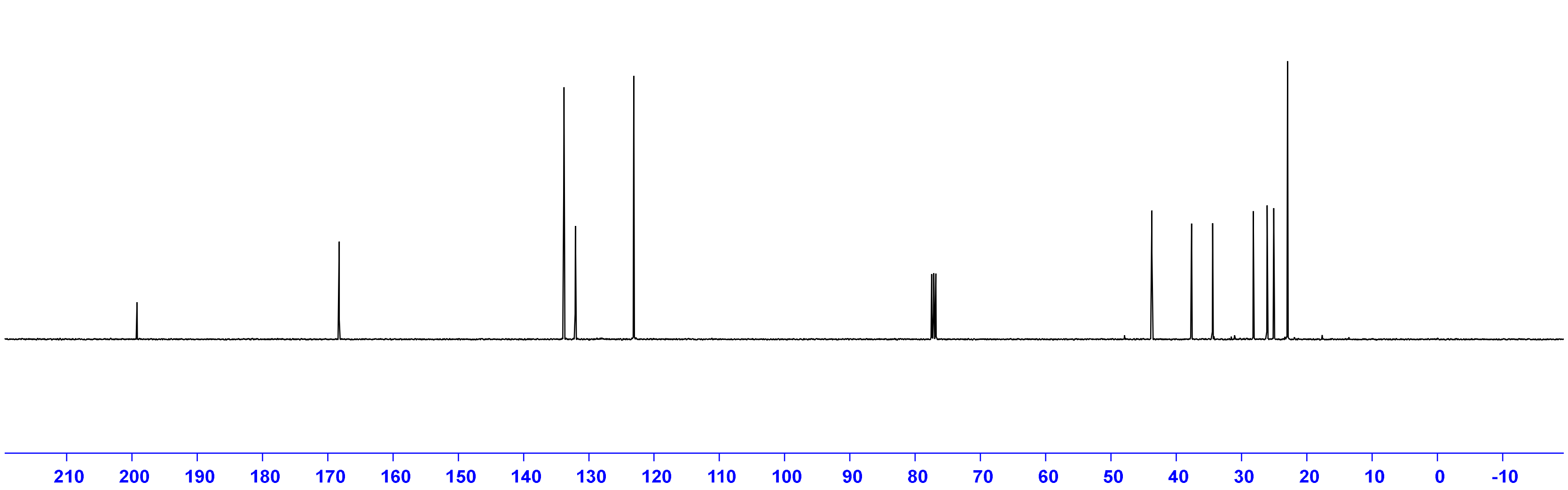

^{1}H NMR spectra for 91

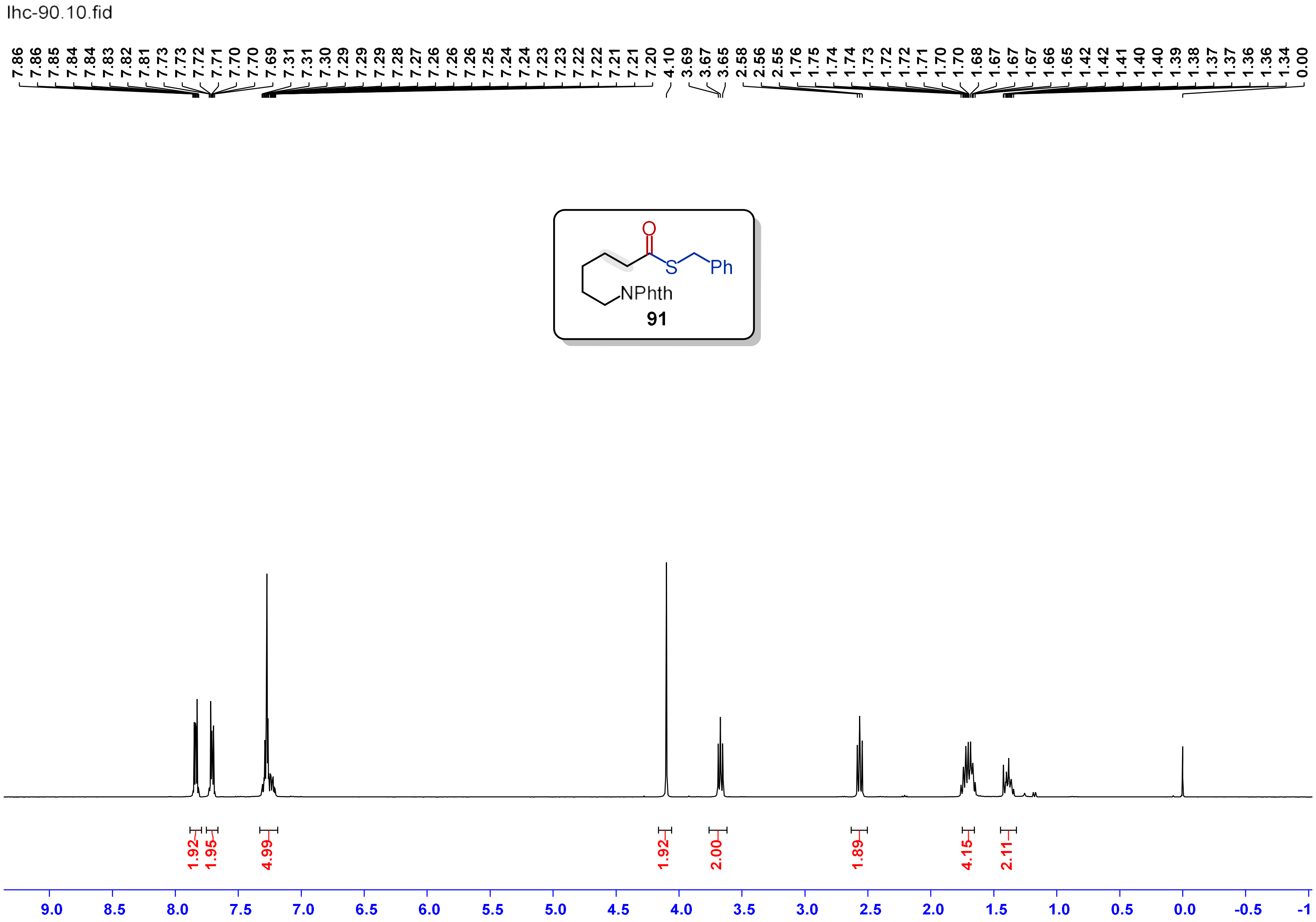

^{13}C NMR spectra for 91

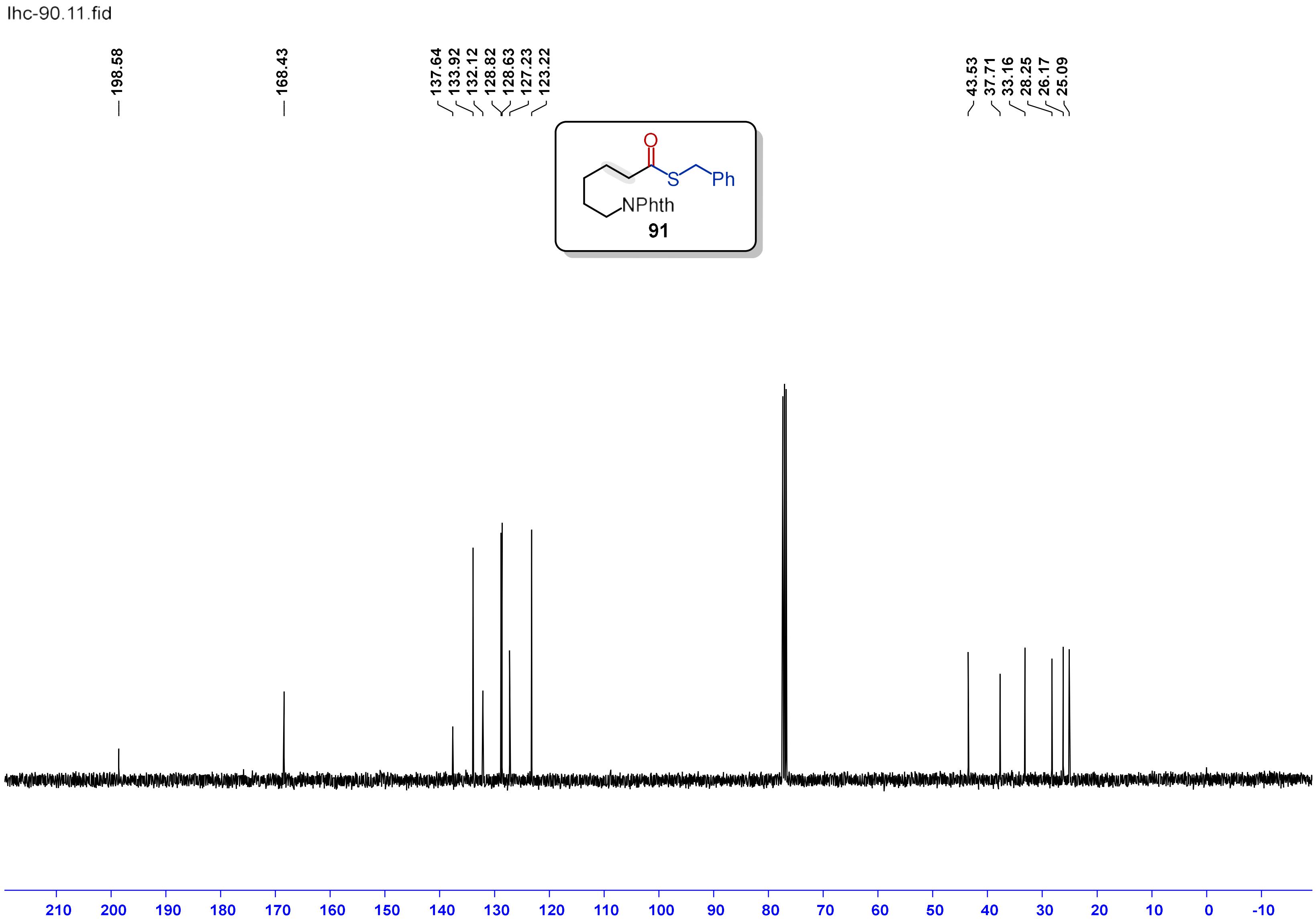

^{1}H NMR spectra for 92

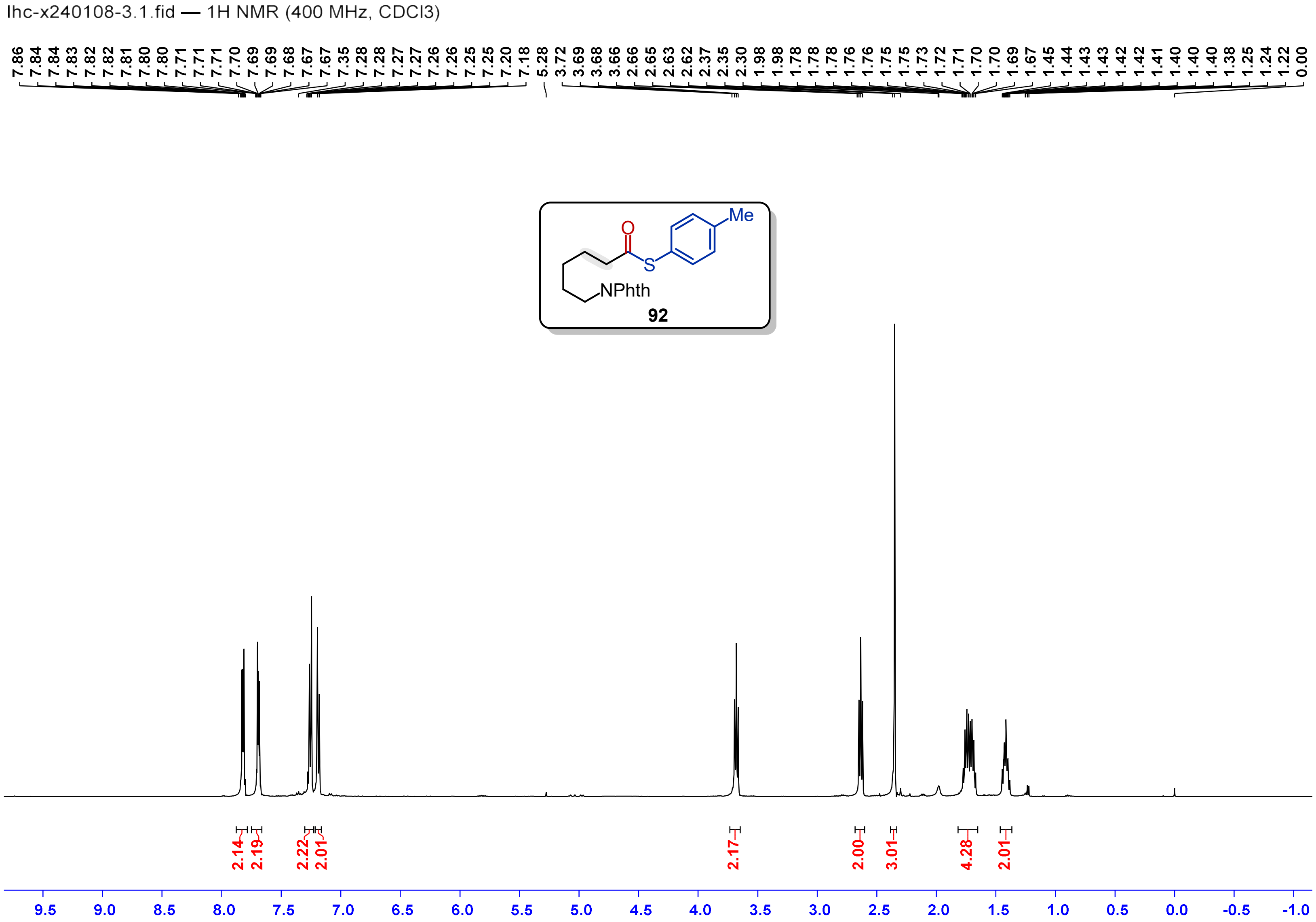

^{13}C NMR spectra for 92

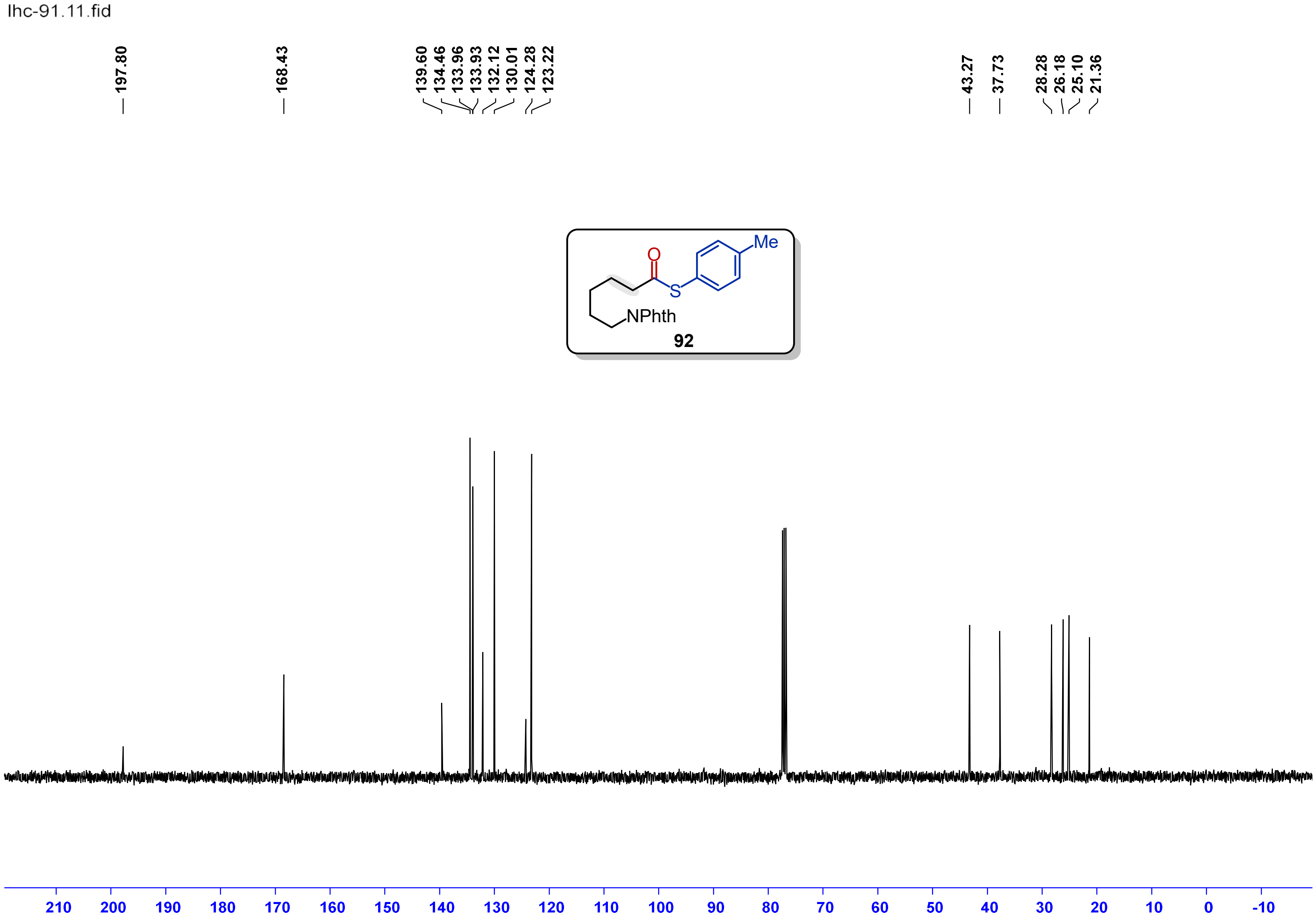

^{1}H NMR spectra for 93

lhc-x240108-6.1.fid — 1H NMR (400 MHz, CDCl3)

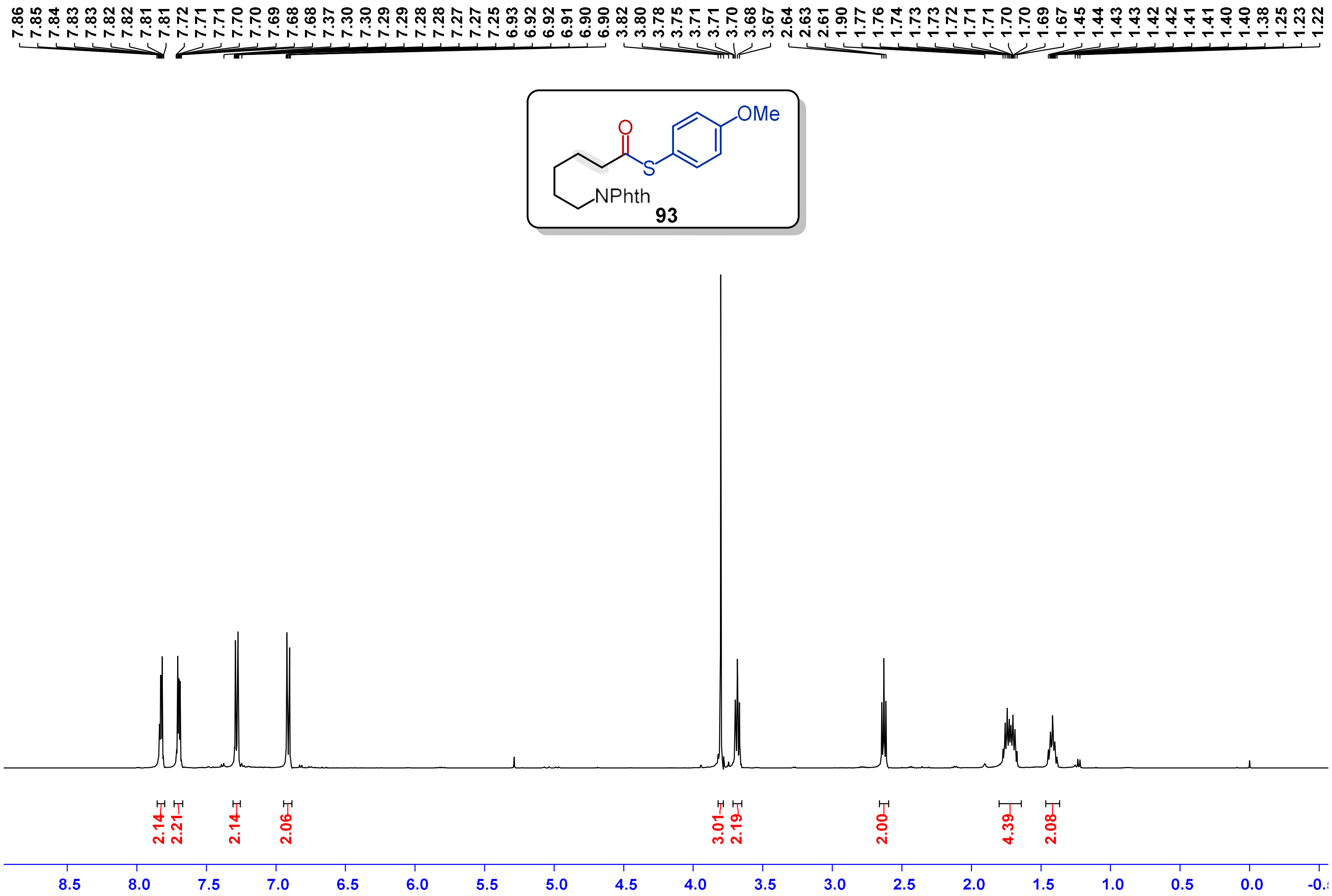

^{13}C NMR spectra for 93

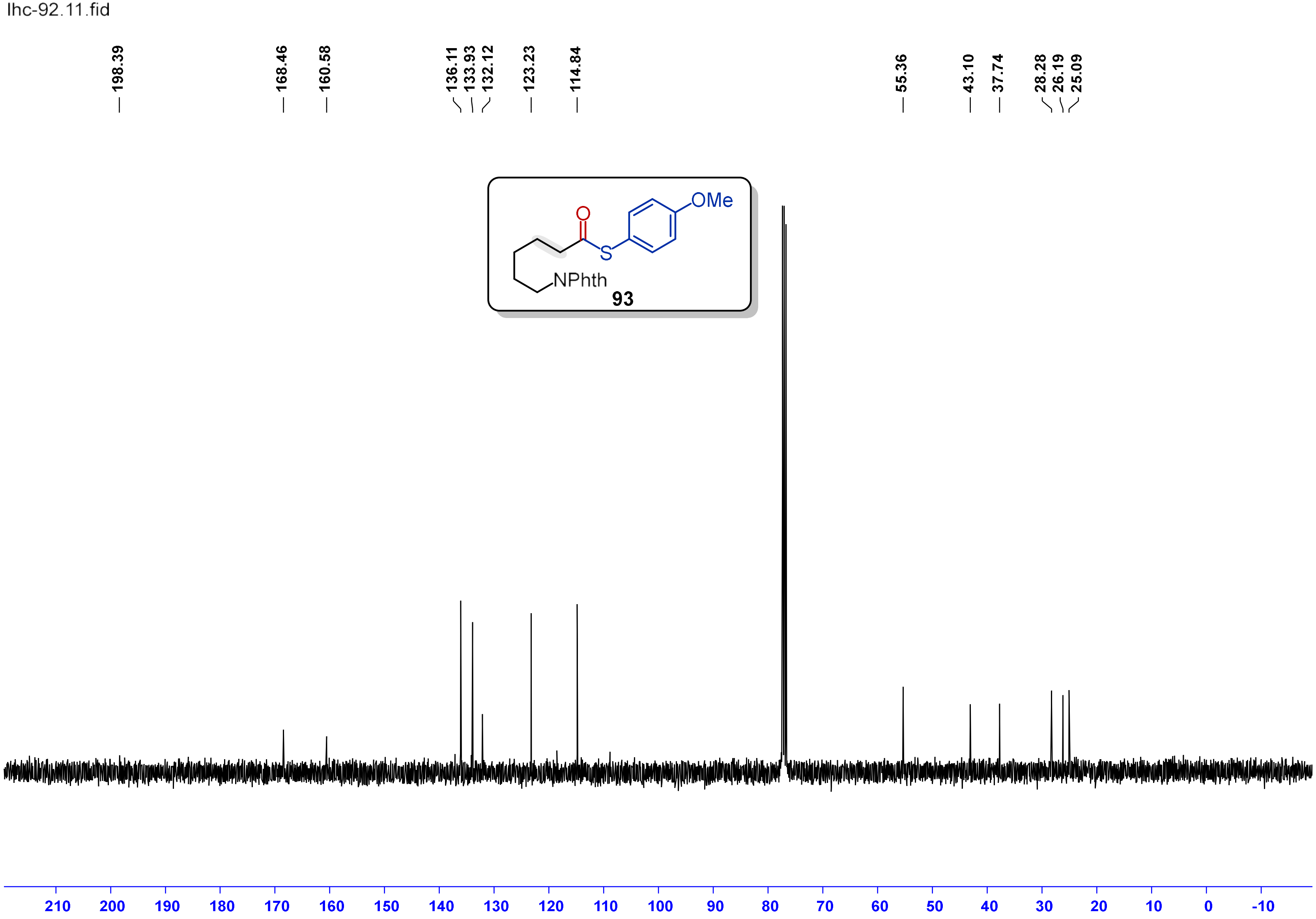

^{1}H NMR spectra for 94

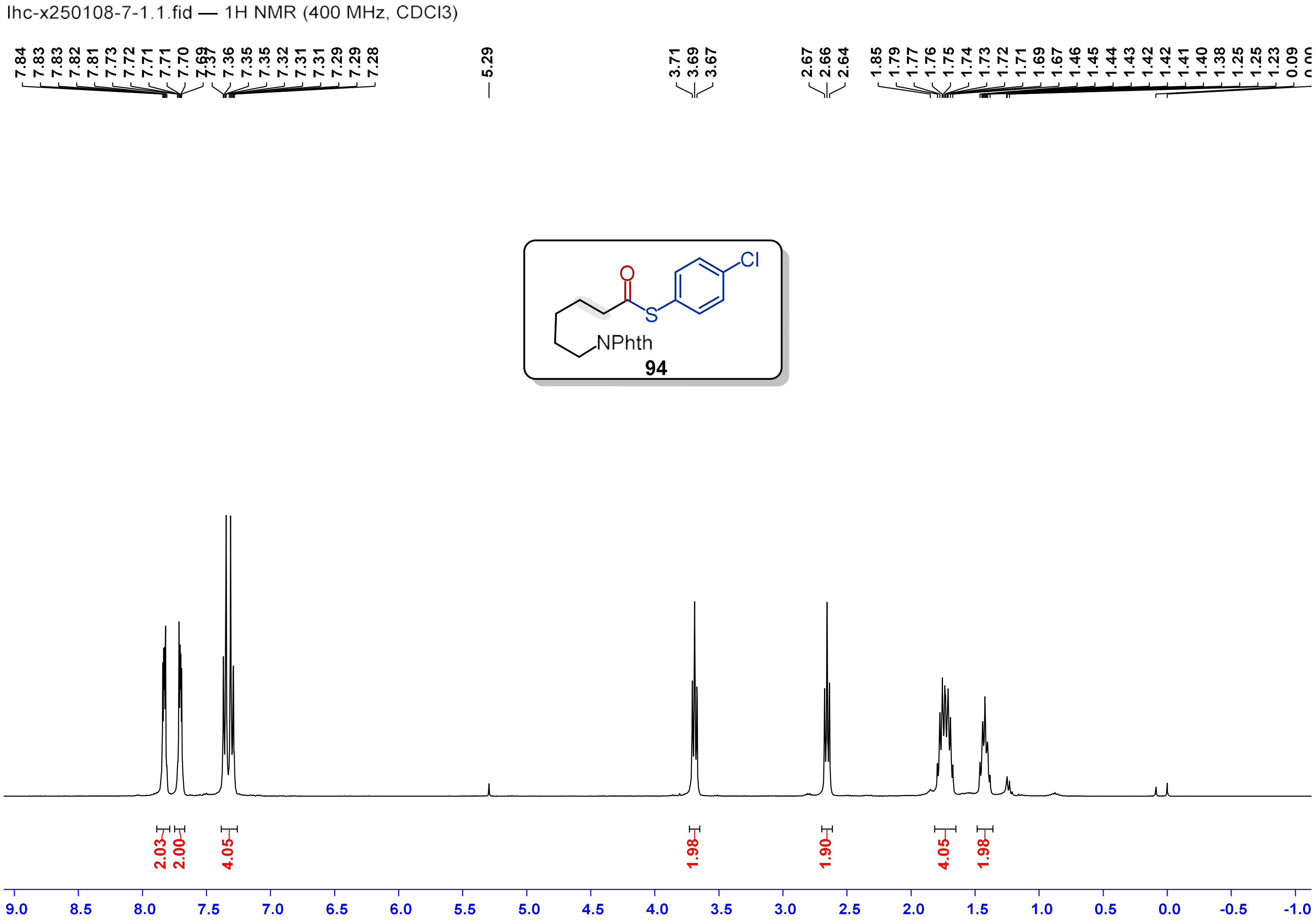

^{13}C NMR spectra for 94

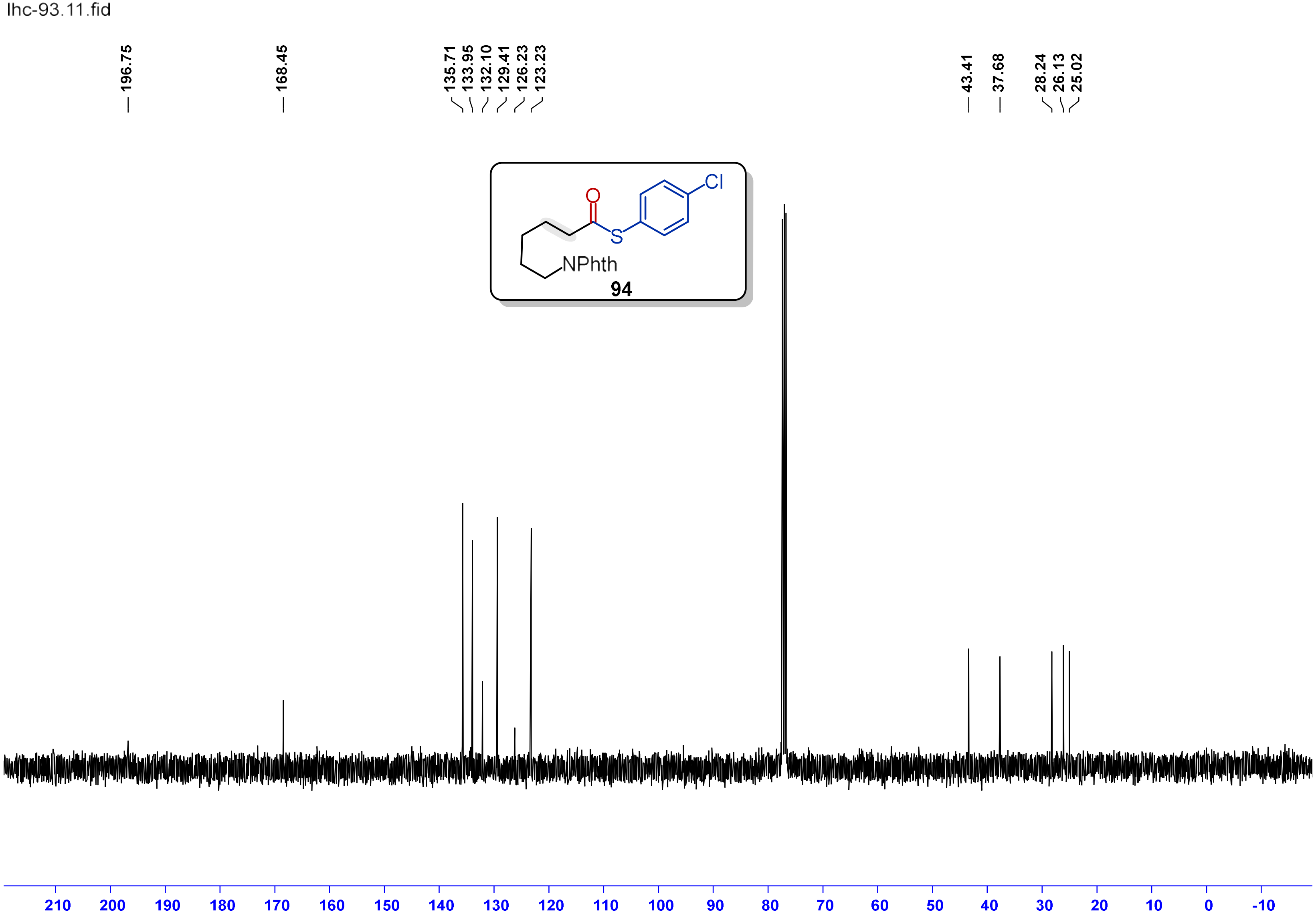

^{1}H NMR spectra for 95

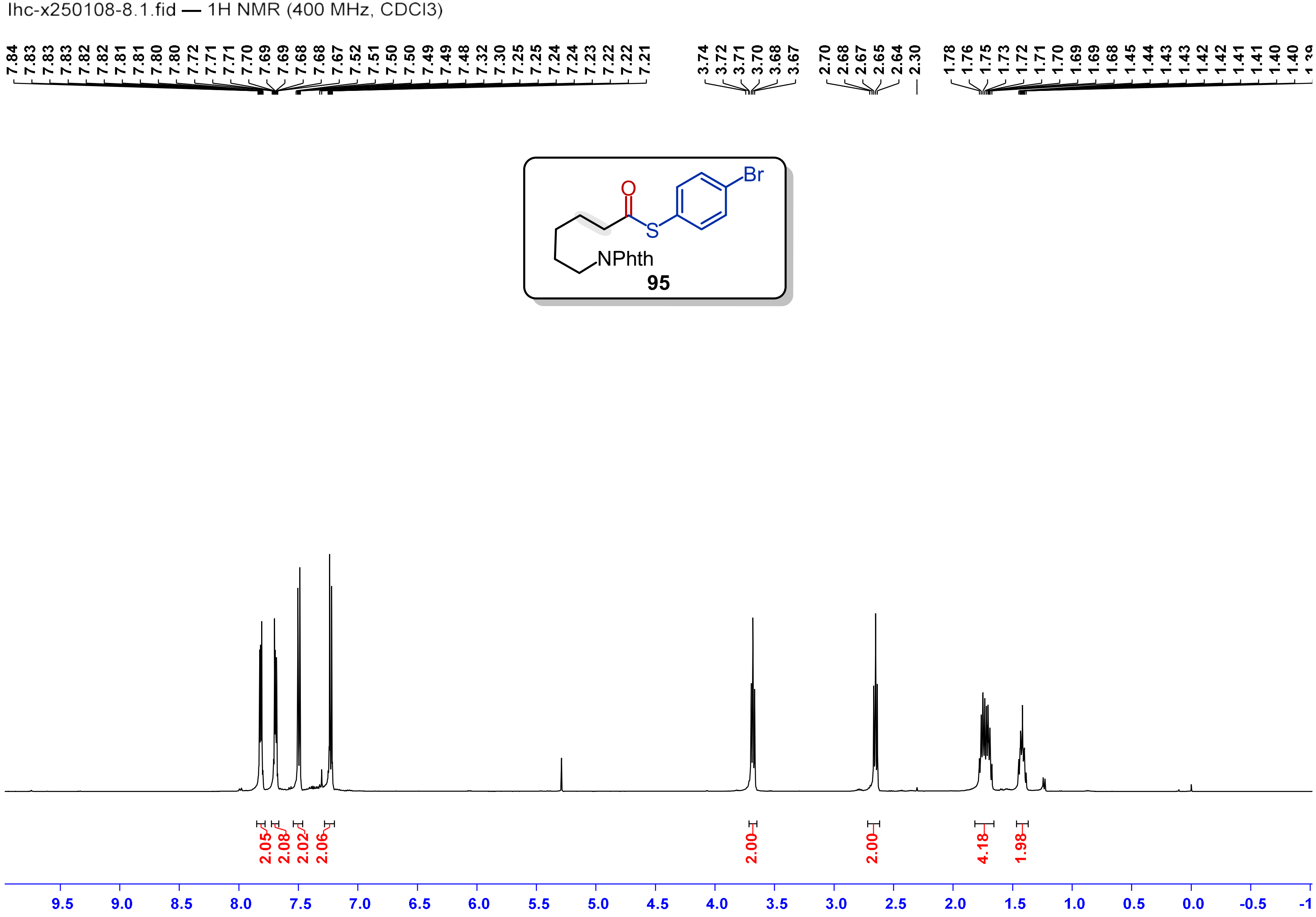

^{13}C NMR spectra for 95

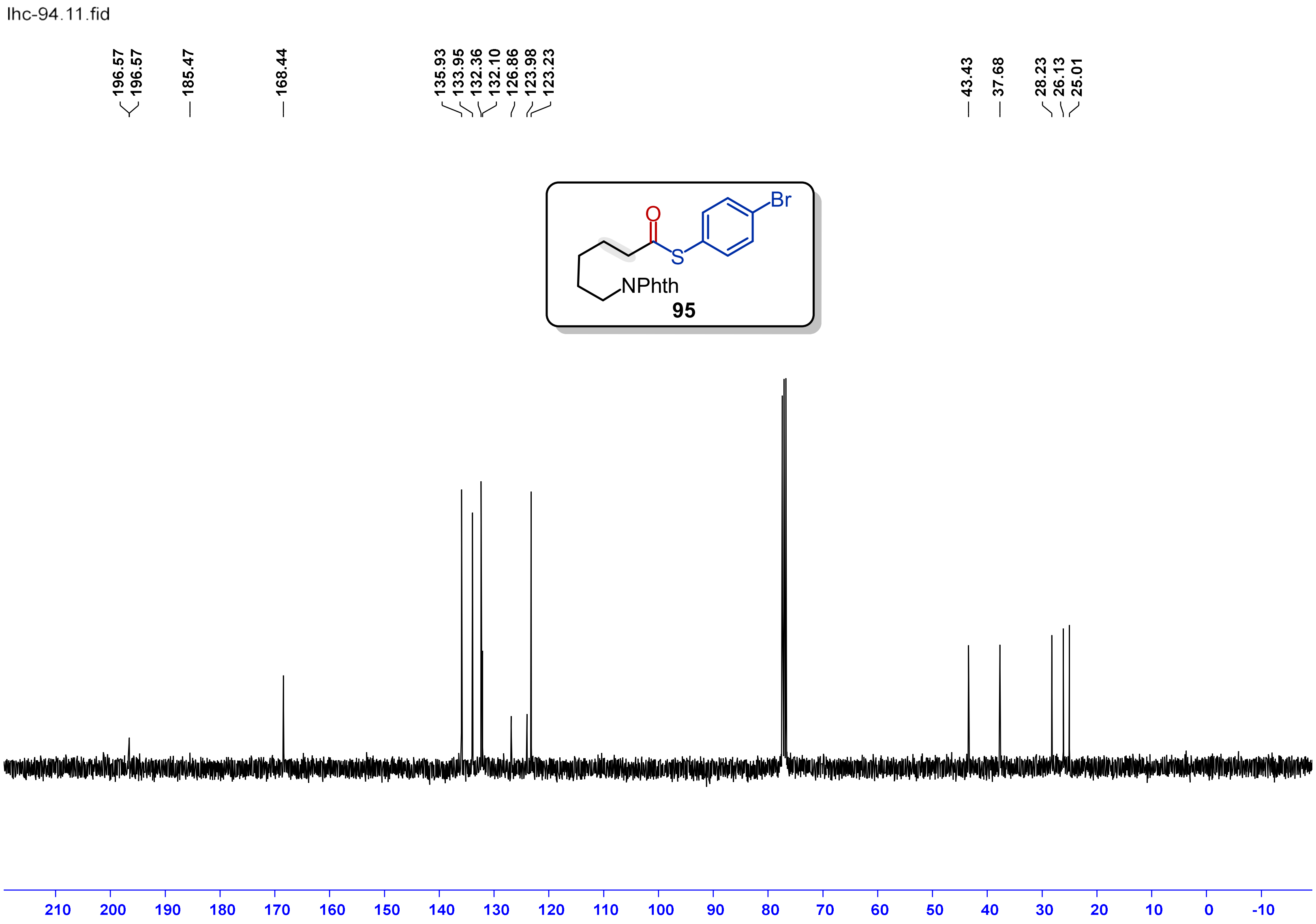

^{1}H NMR spectra for 96

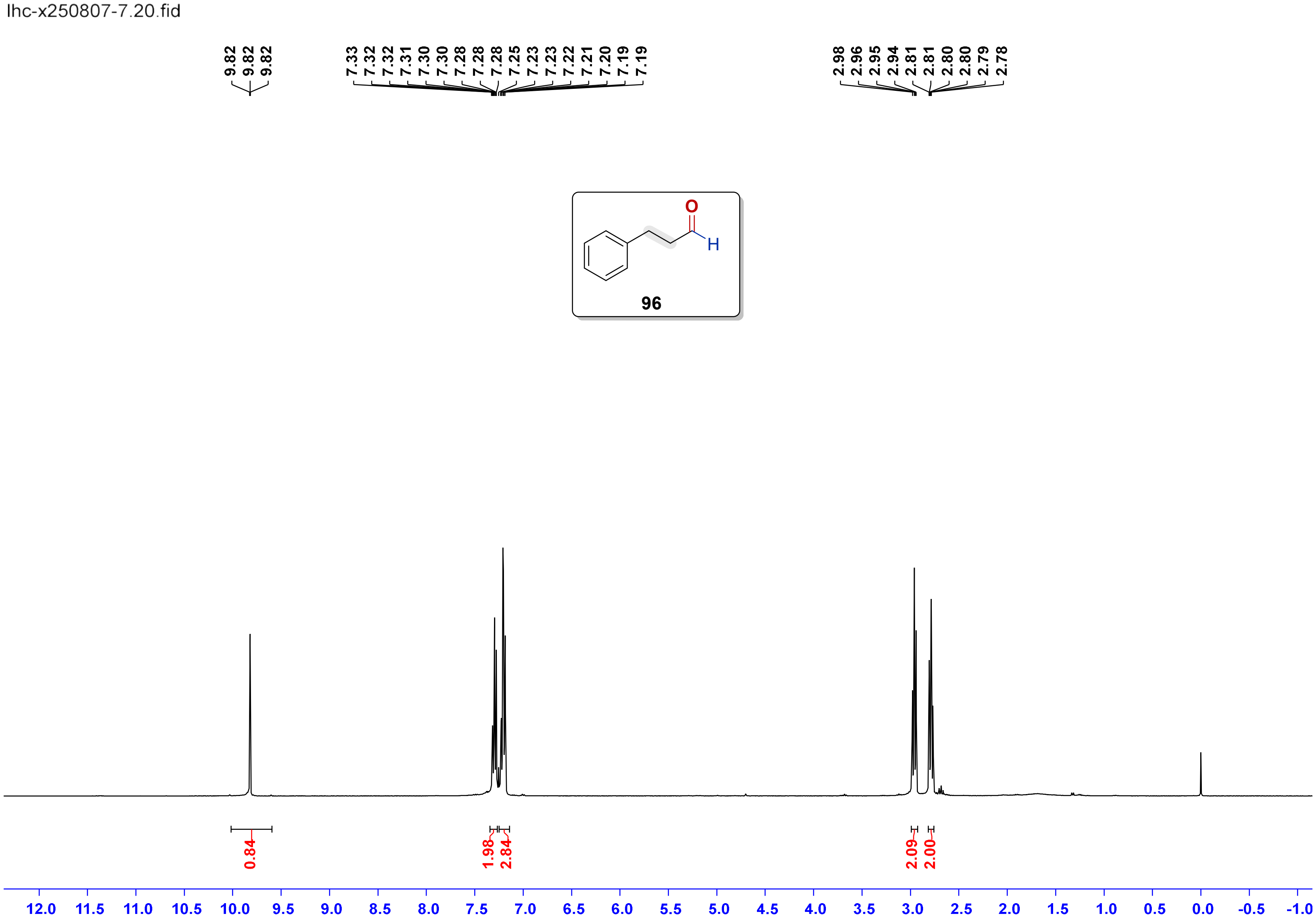

^{13}C NMR spectra for 96

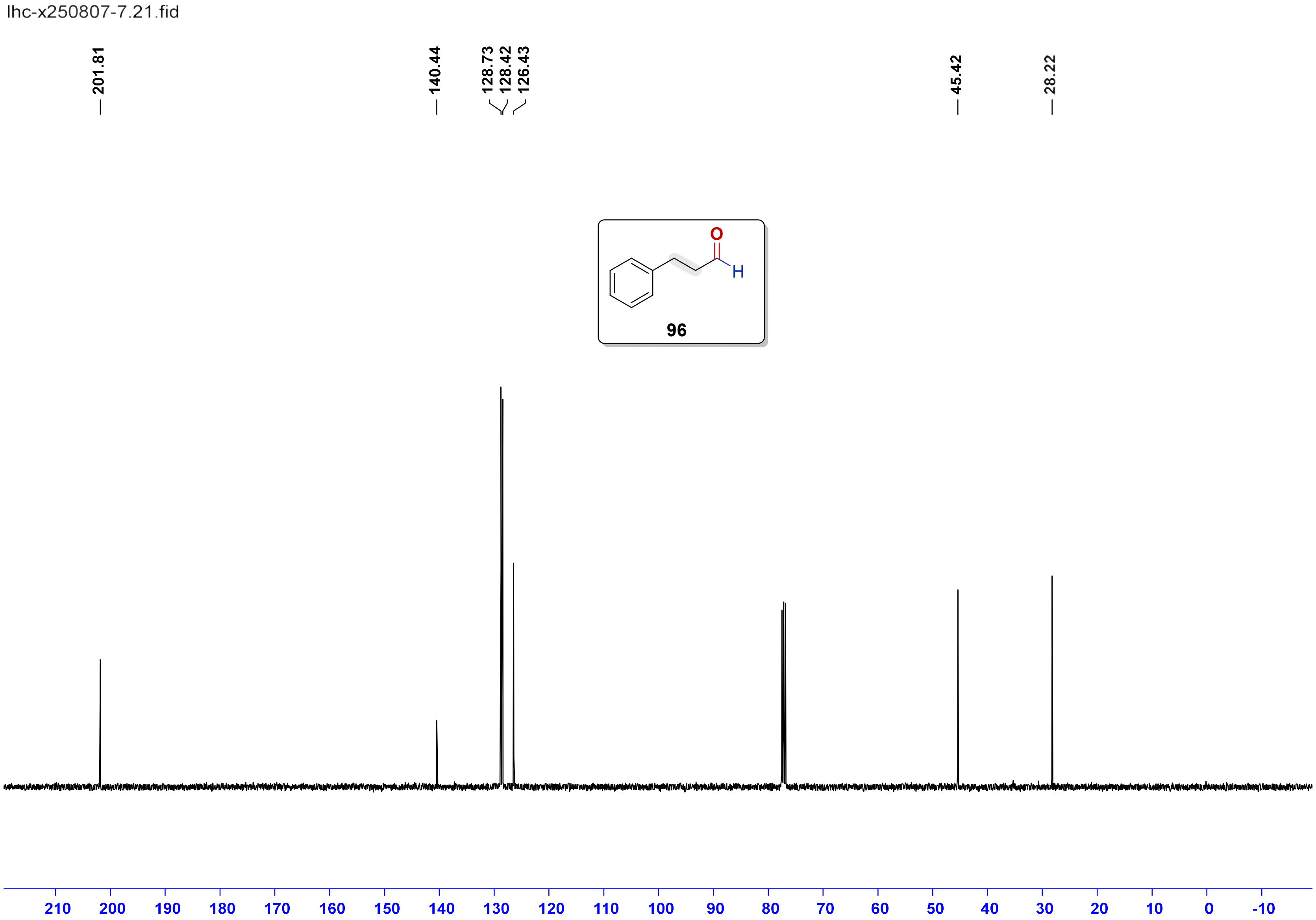

^{1}H NMR spectra for 97

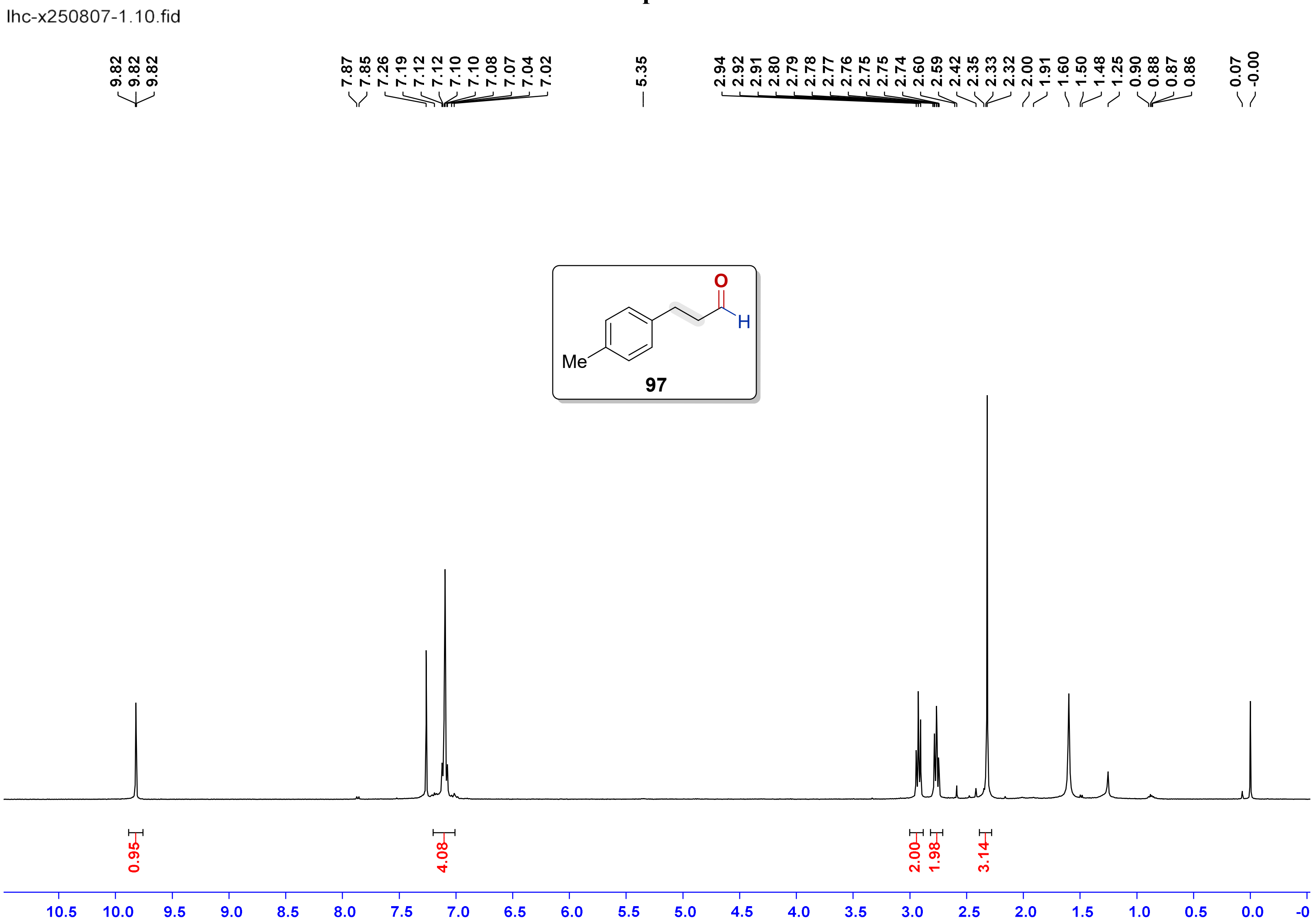

^{13}C NMR spectra for 97

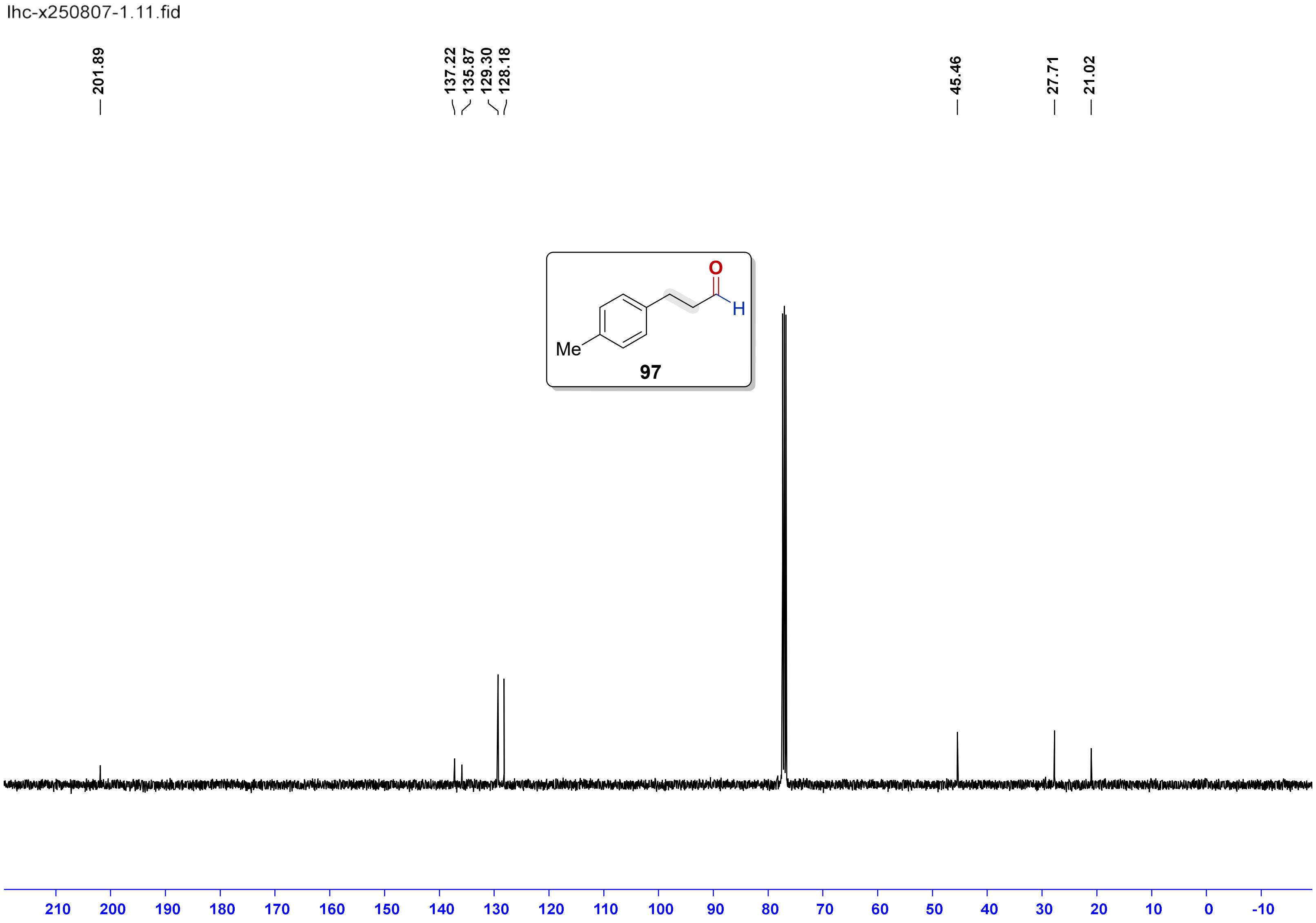

^{1}H NMR spectra for 98

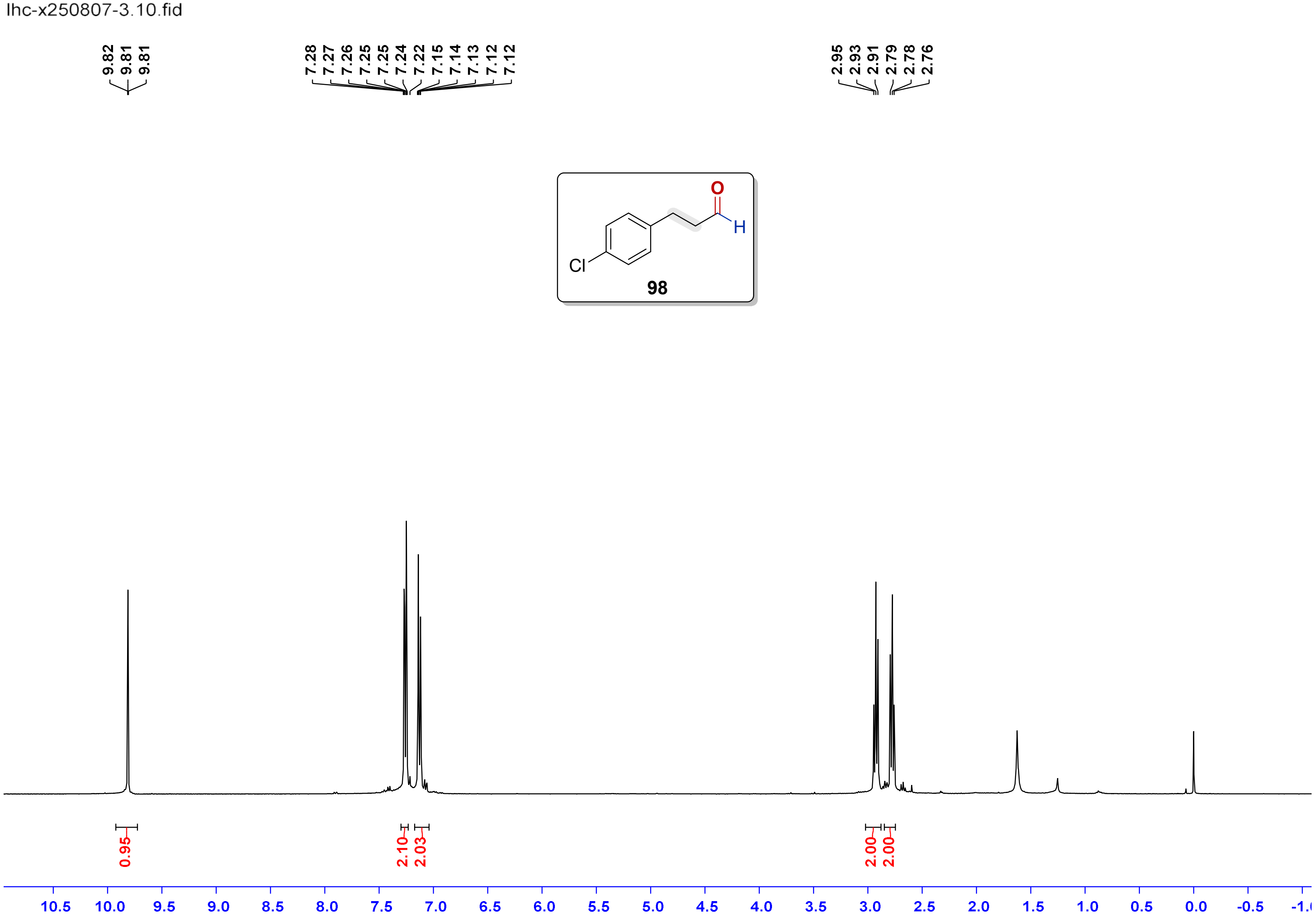

^{13}C NMR spectra for 98

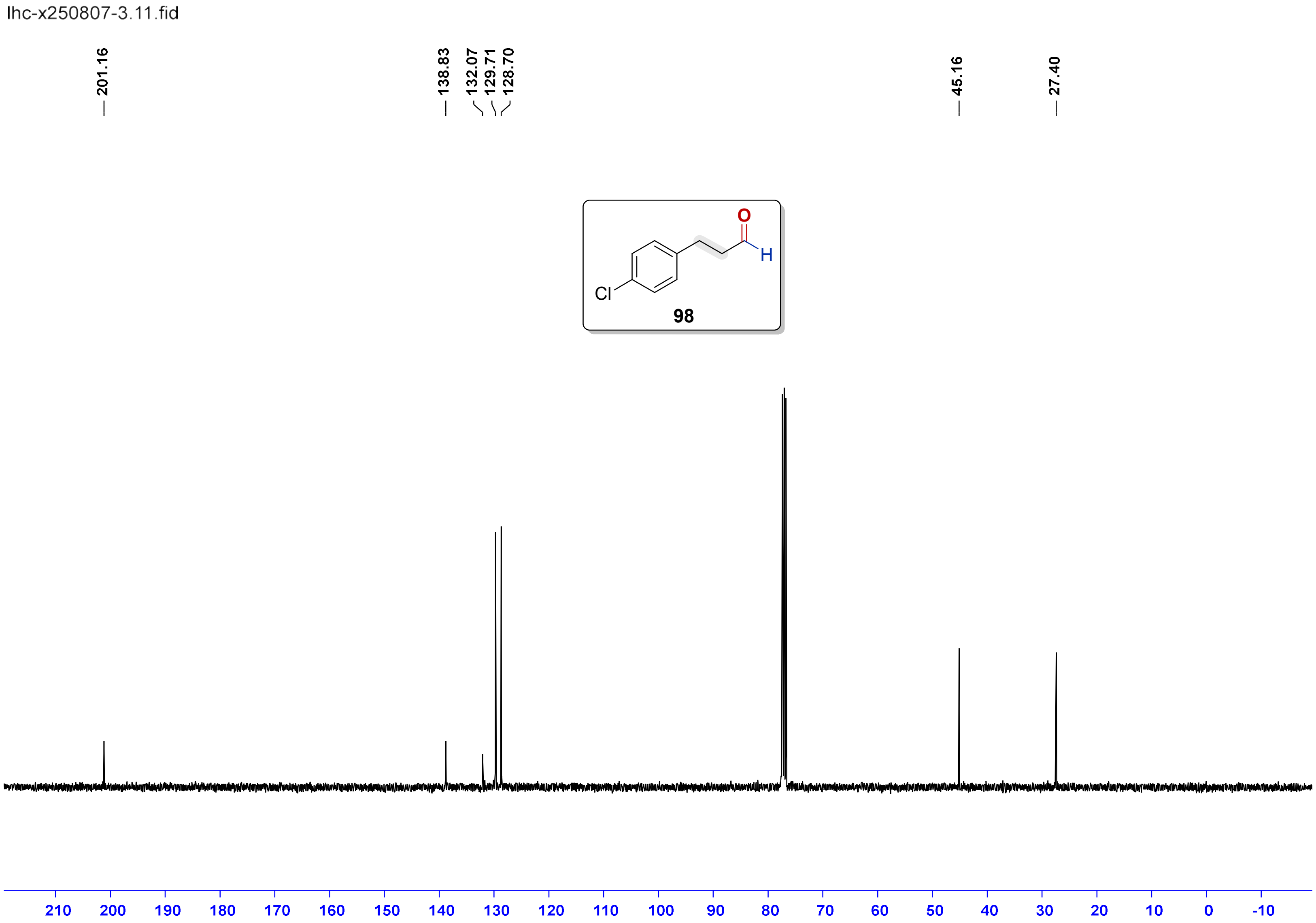

^{1}H NMR spectra for 99

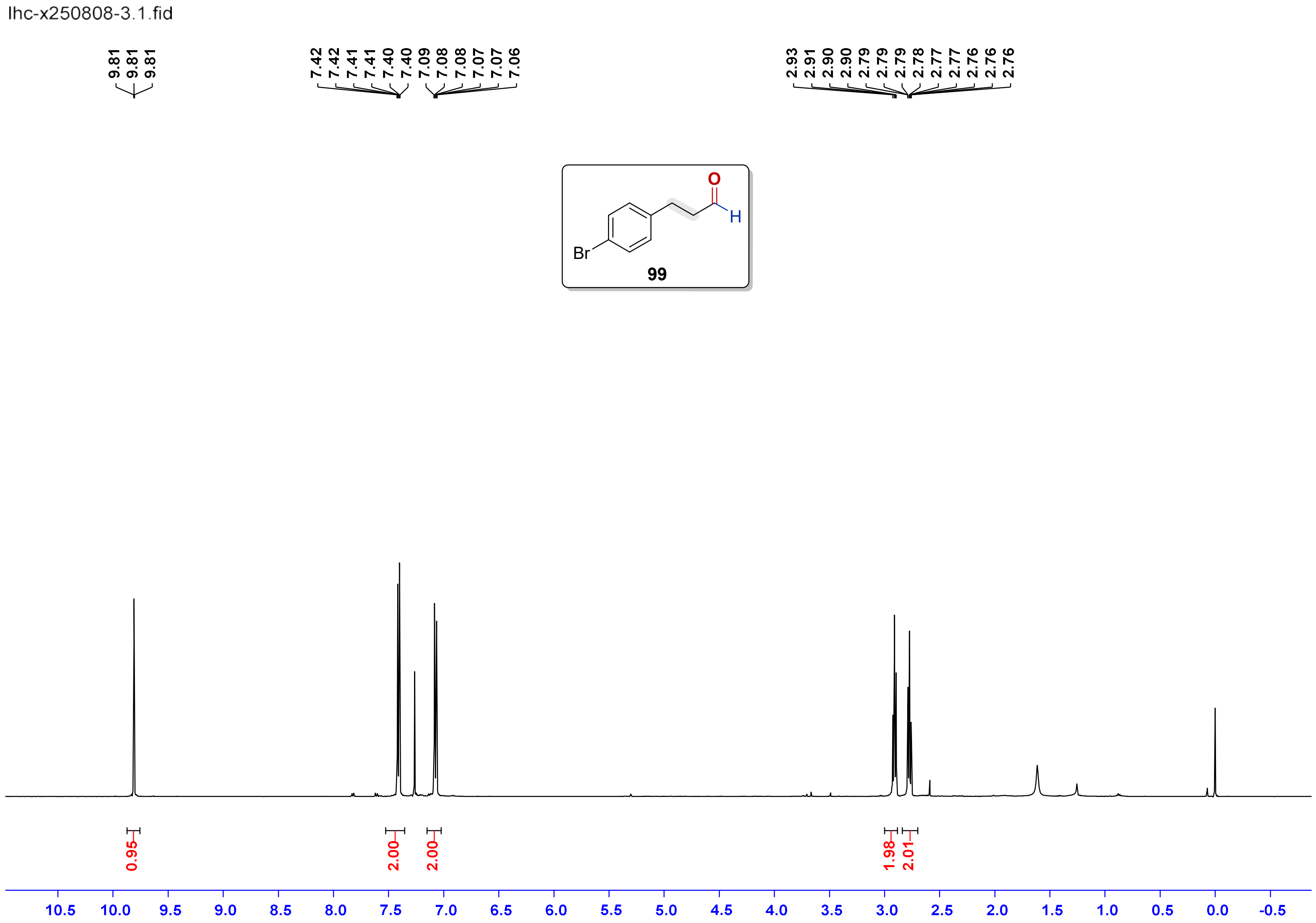

^{13}C NMR spectra for 99

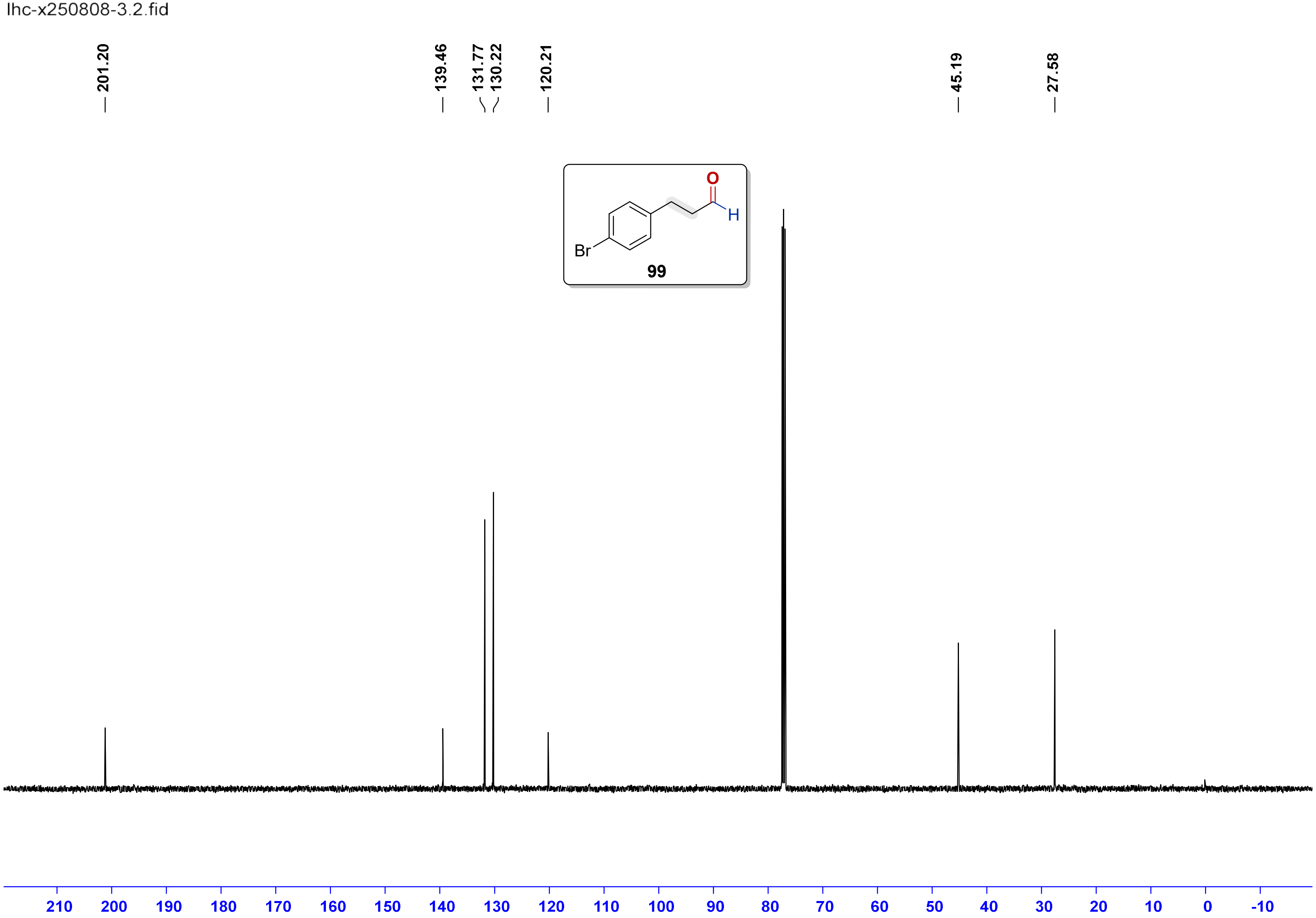

^{1}H NMR spectra for 100

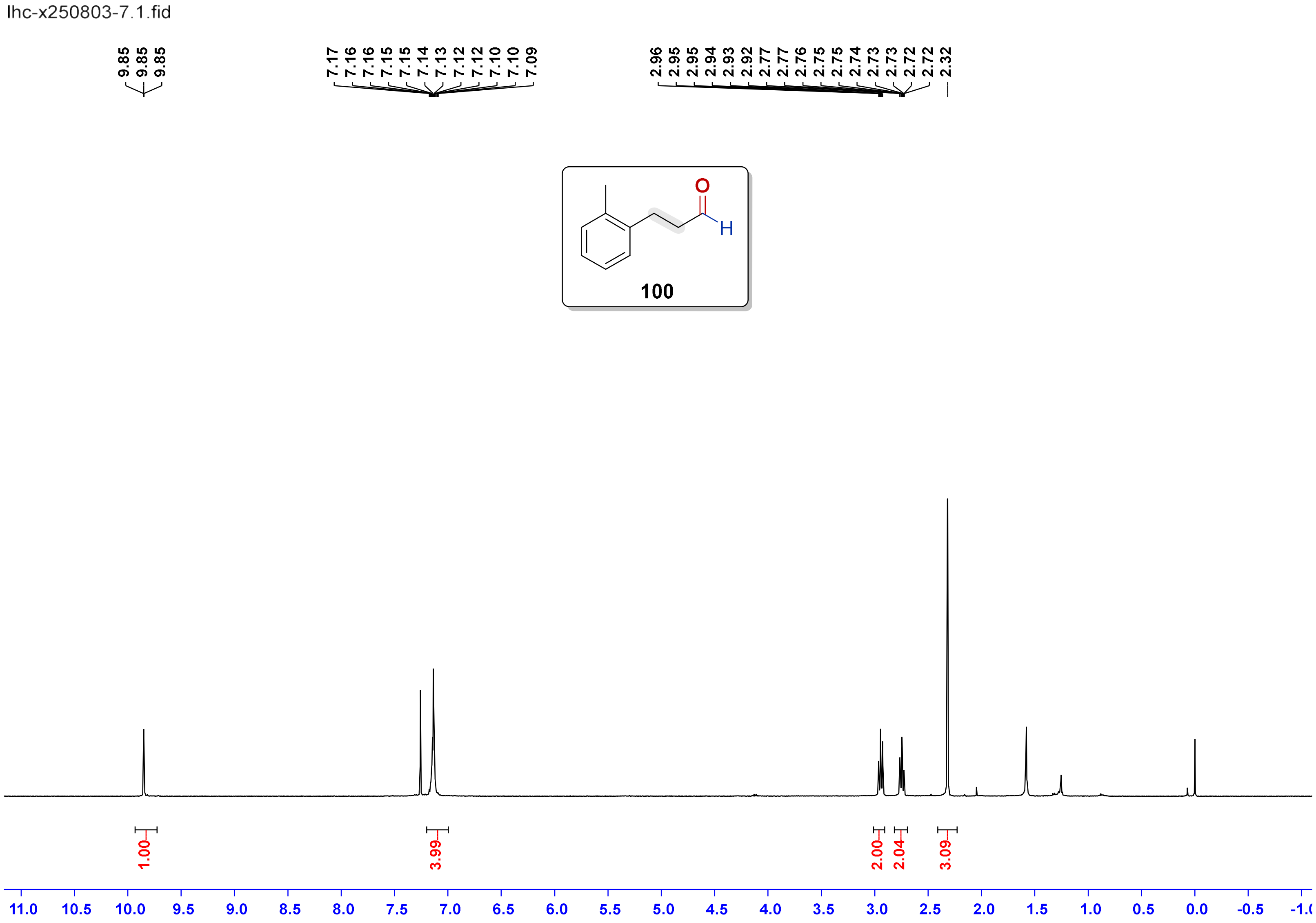

^{13}C NMR spectra for 100

lhc-x250803-7.2.fid

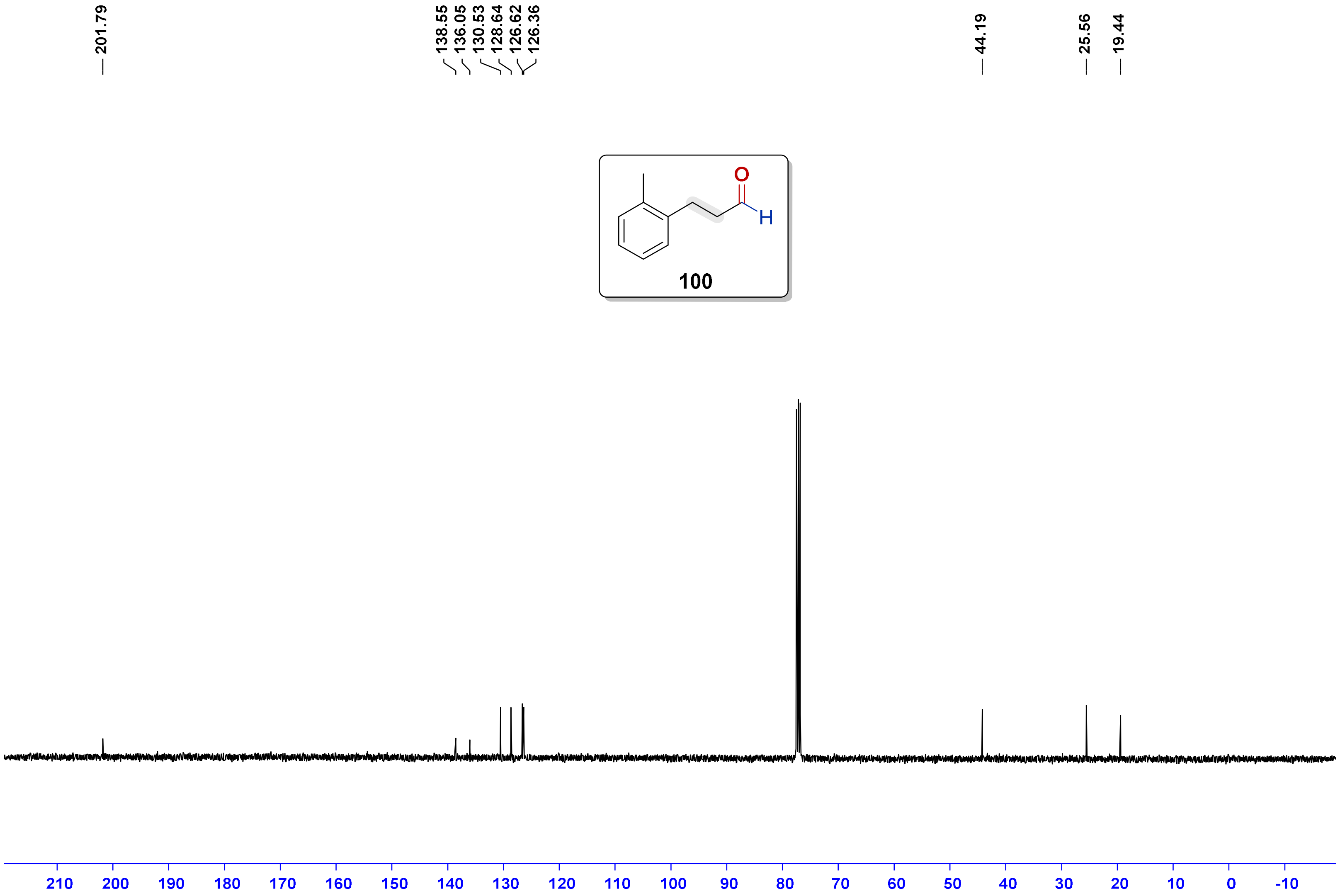

^{1}H NMR spectra for 101a

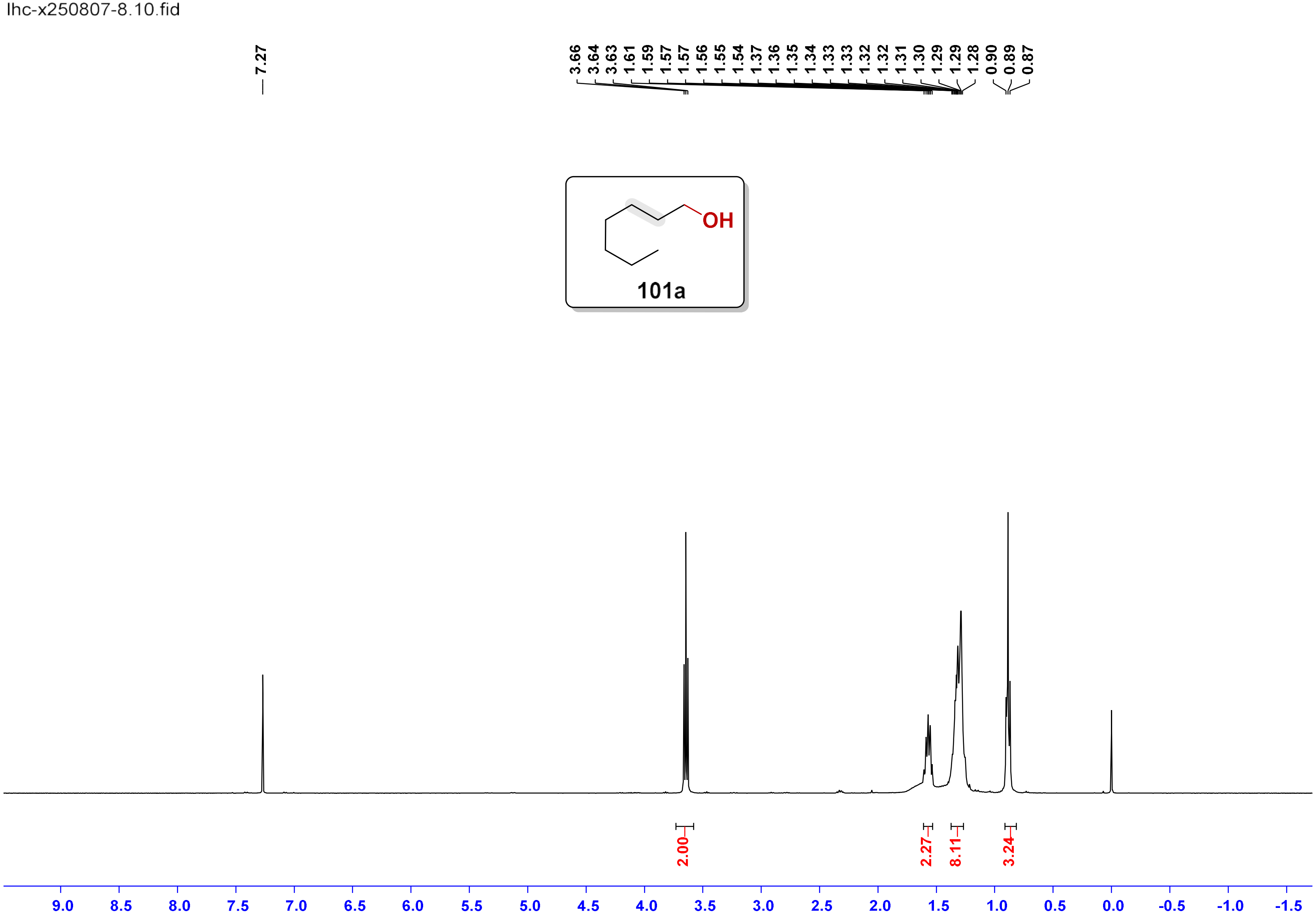

^{13}C NMR spectra for 101a

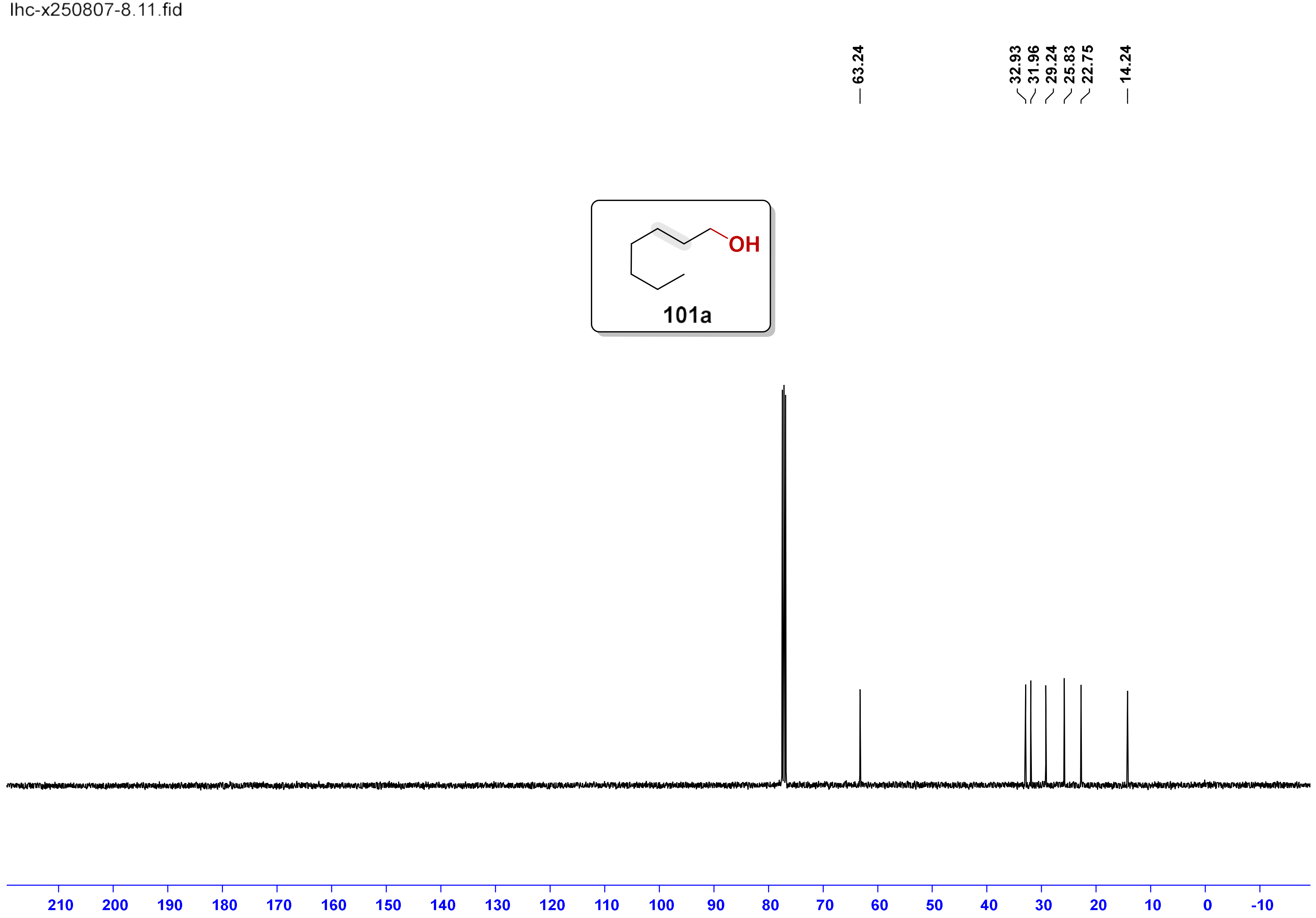

^{1}H NMR spectra for 102a

lhc-x250806-2.10.fid

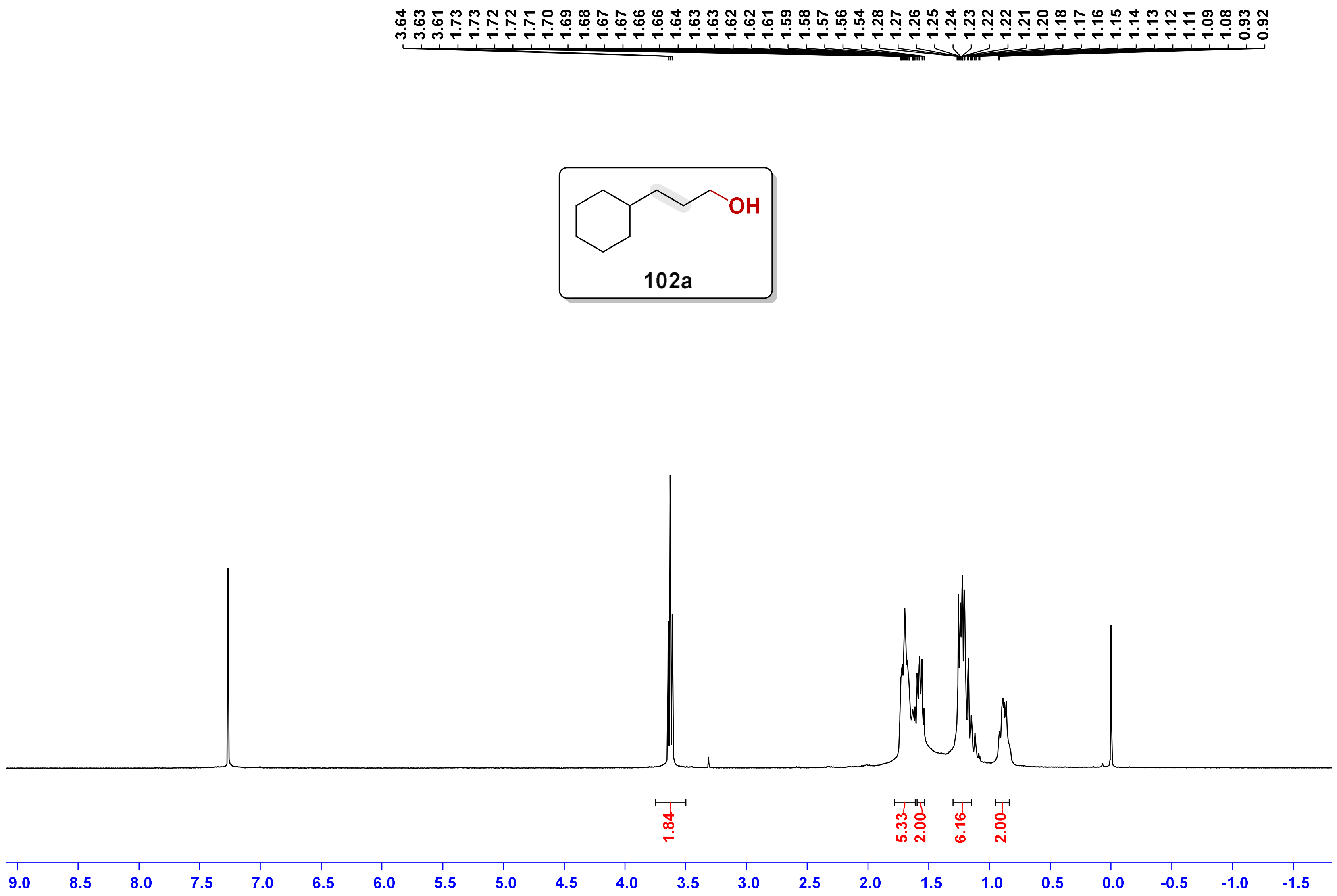

^{13}C NMR spectra for 102a

lhc-x250806-2.12.fid

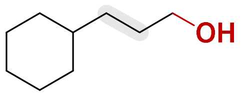

102a

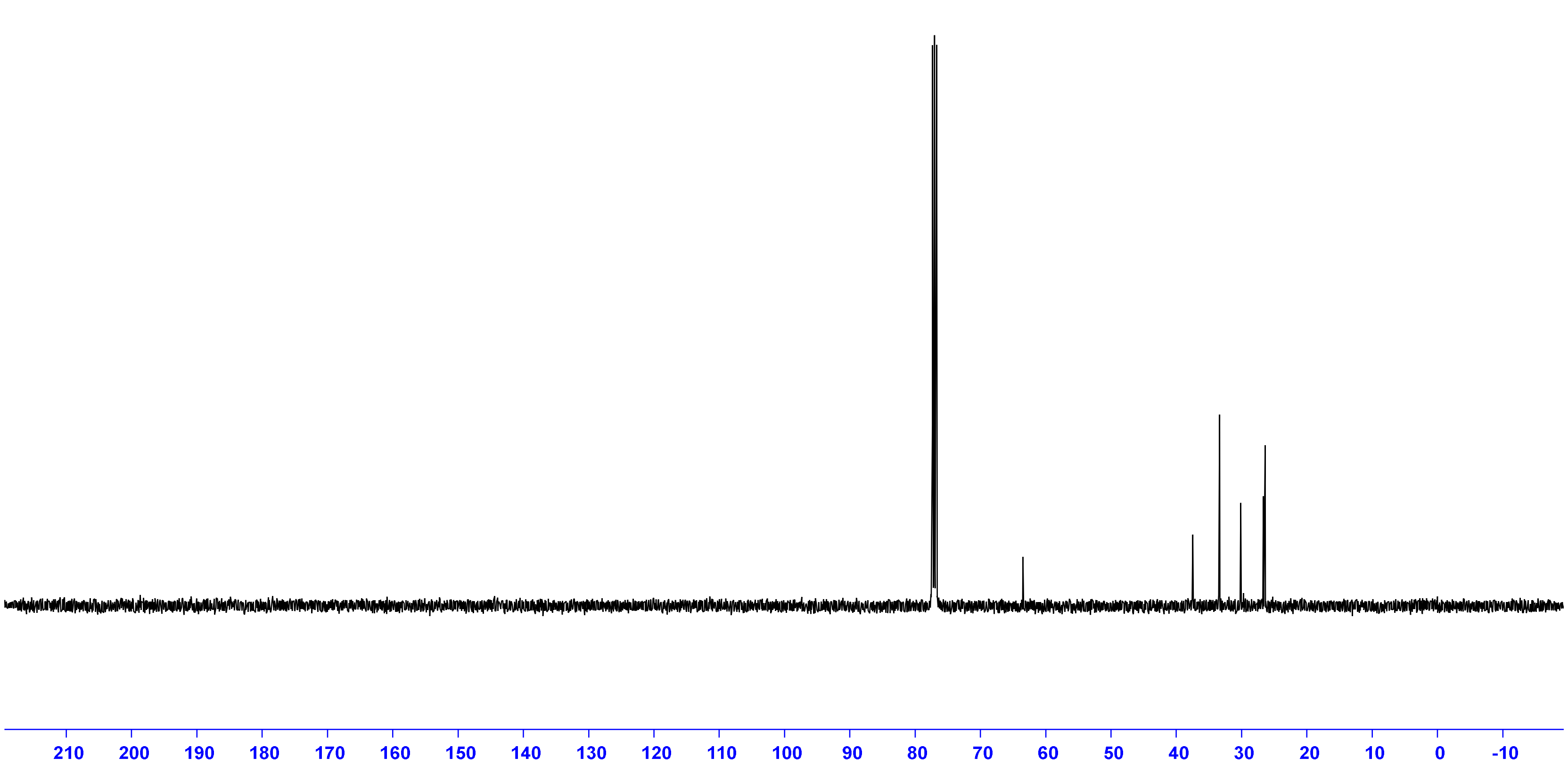

^{1}H NMR spectra for 103a

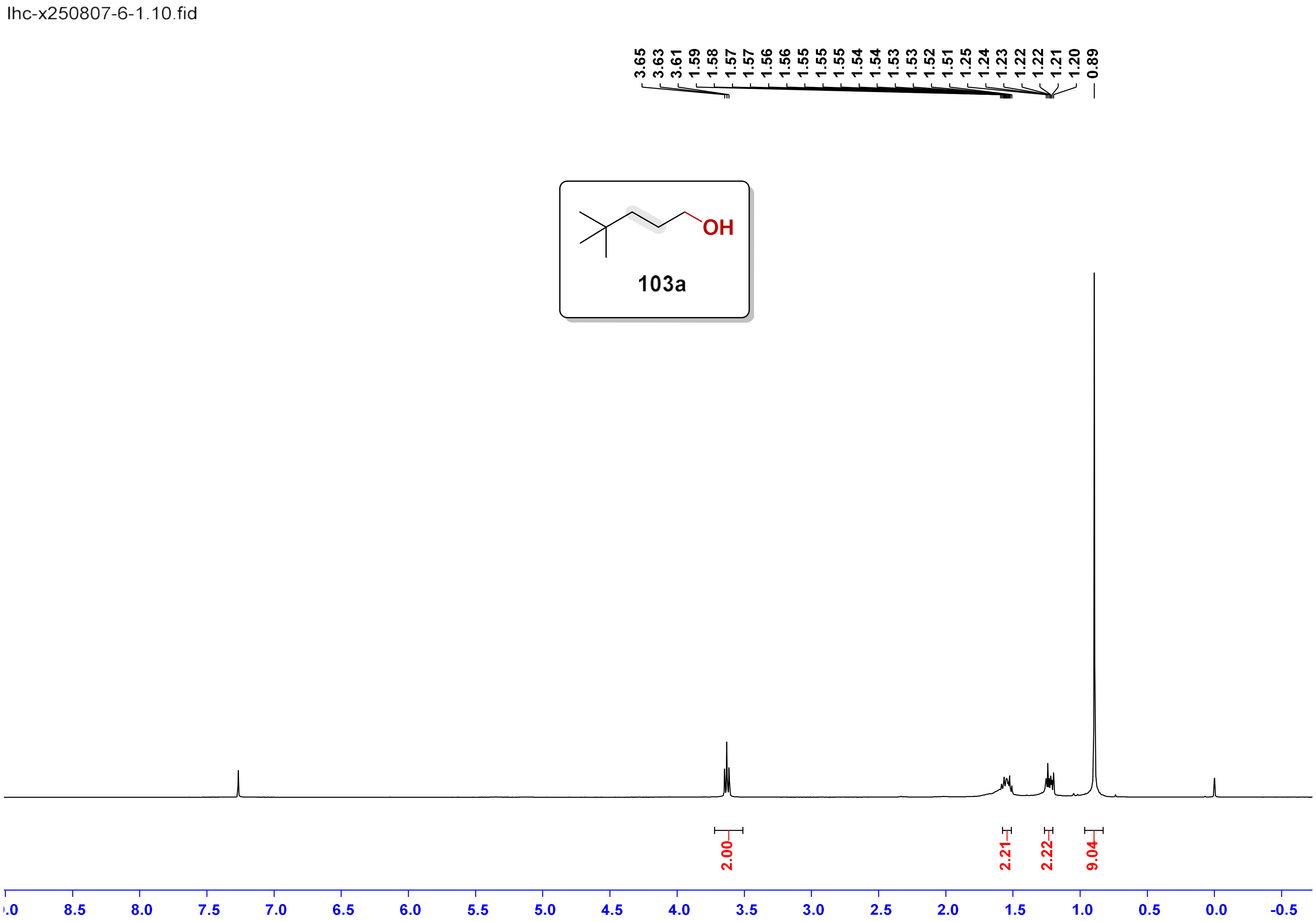

^{13}C NMR spectra for 103a

lhc-x250807-6-1.12.fid

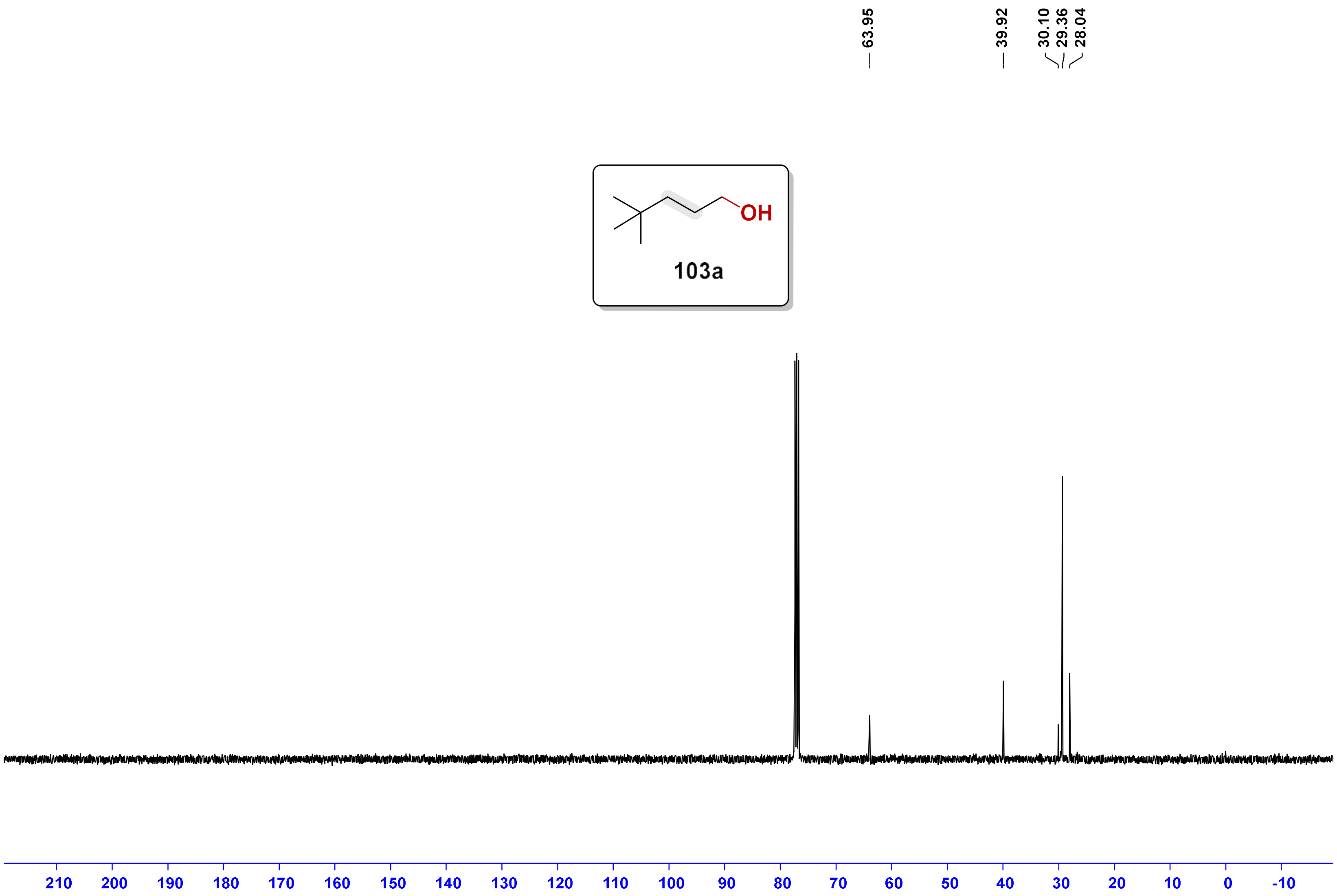

^{1}H NMR spectra for 104

lhc-x250803-2.1.fid

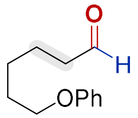

104

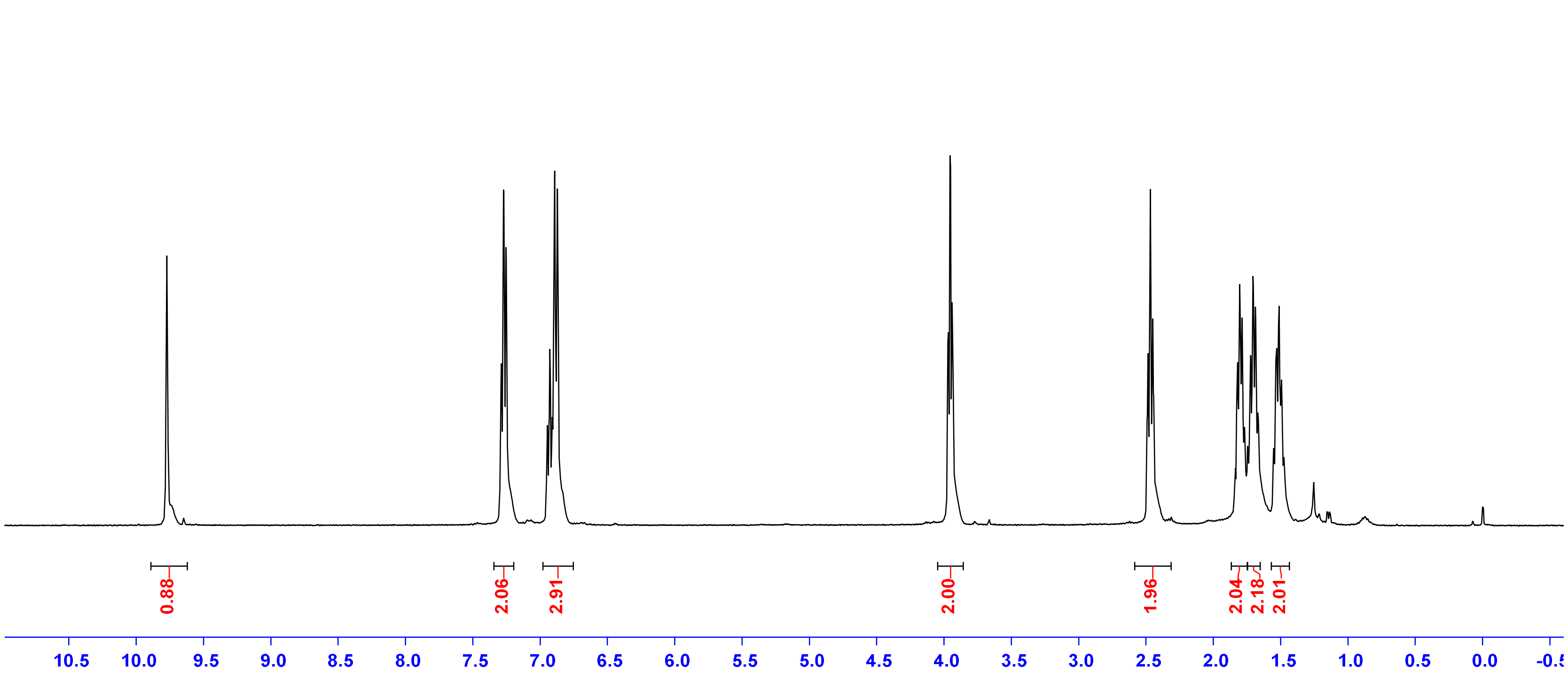

^{13}C NMR spectra for 104

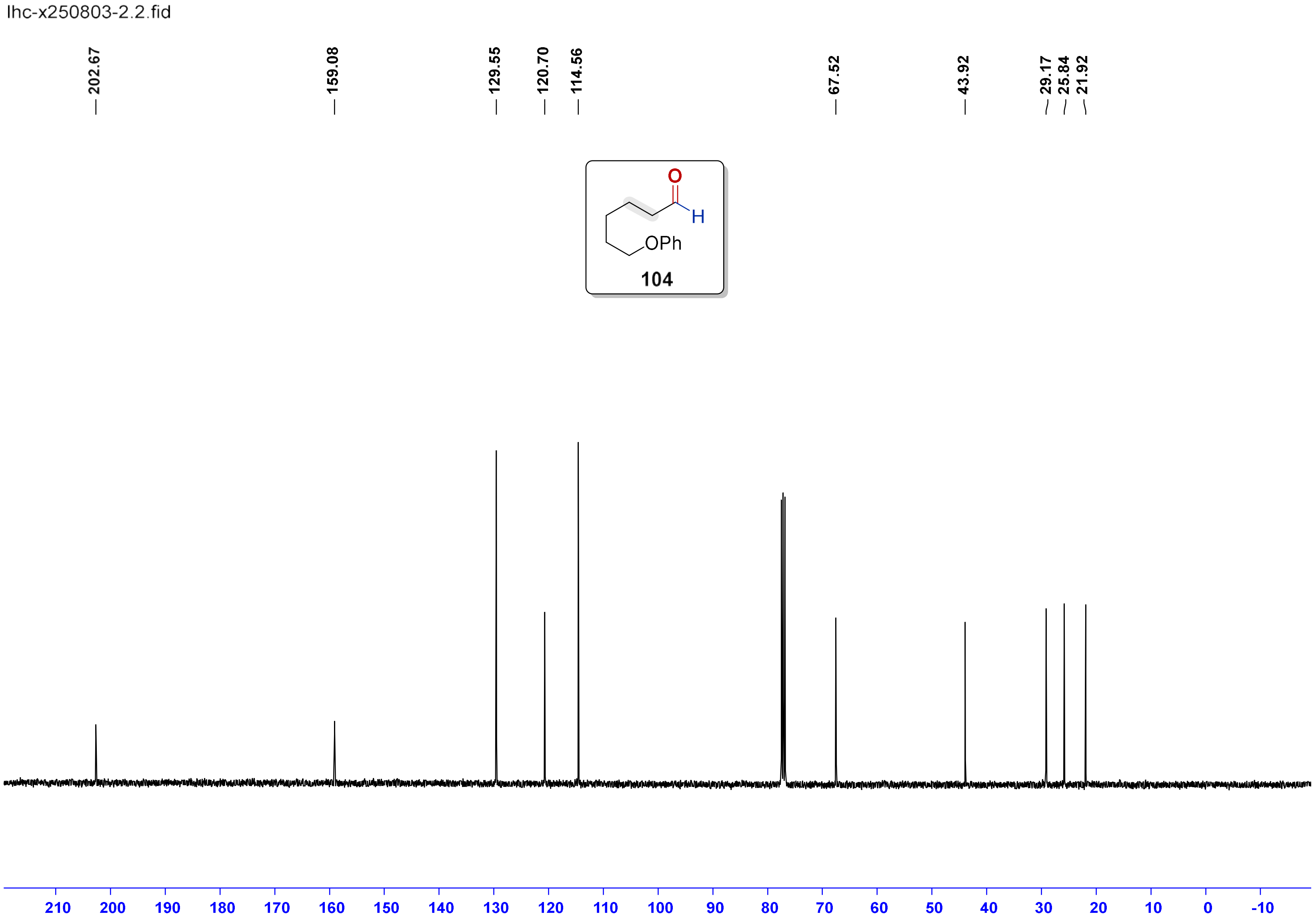

^{1}H NMR spectra for 105

lhc-x250804-3.1.fid

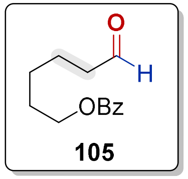

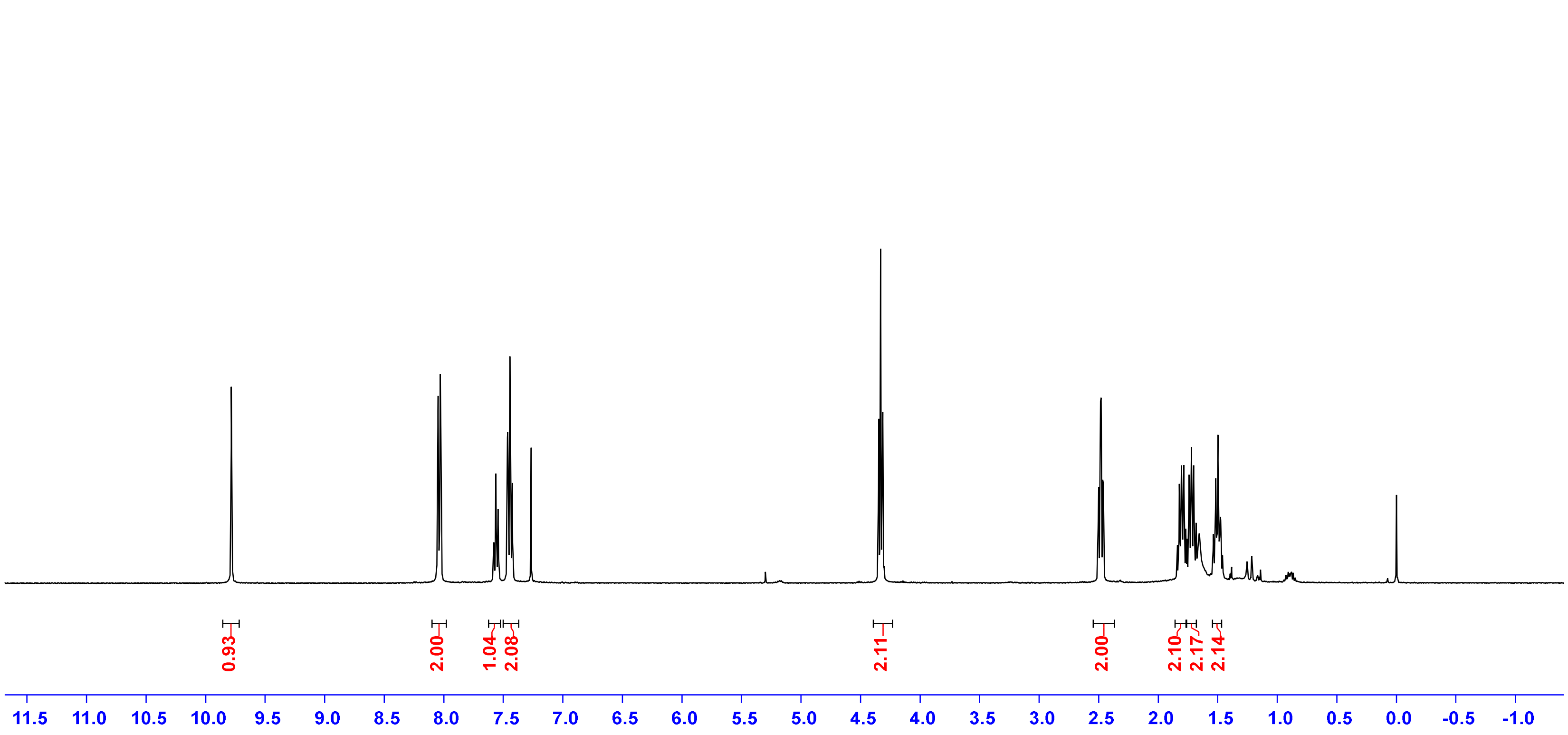

^{13}C NMR spectra for 105

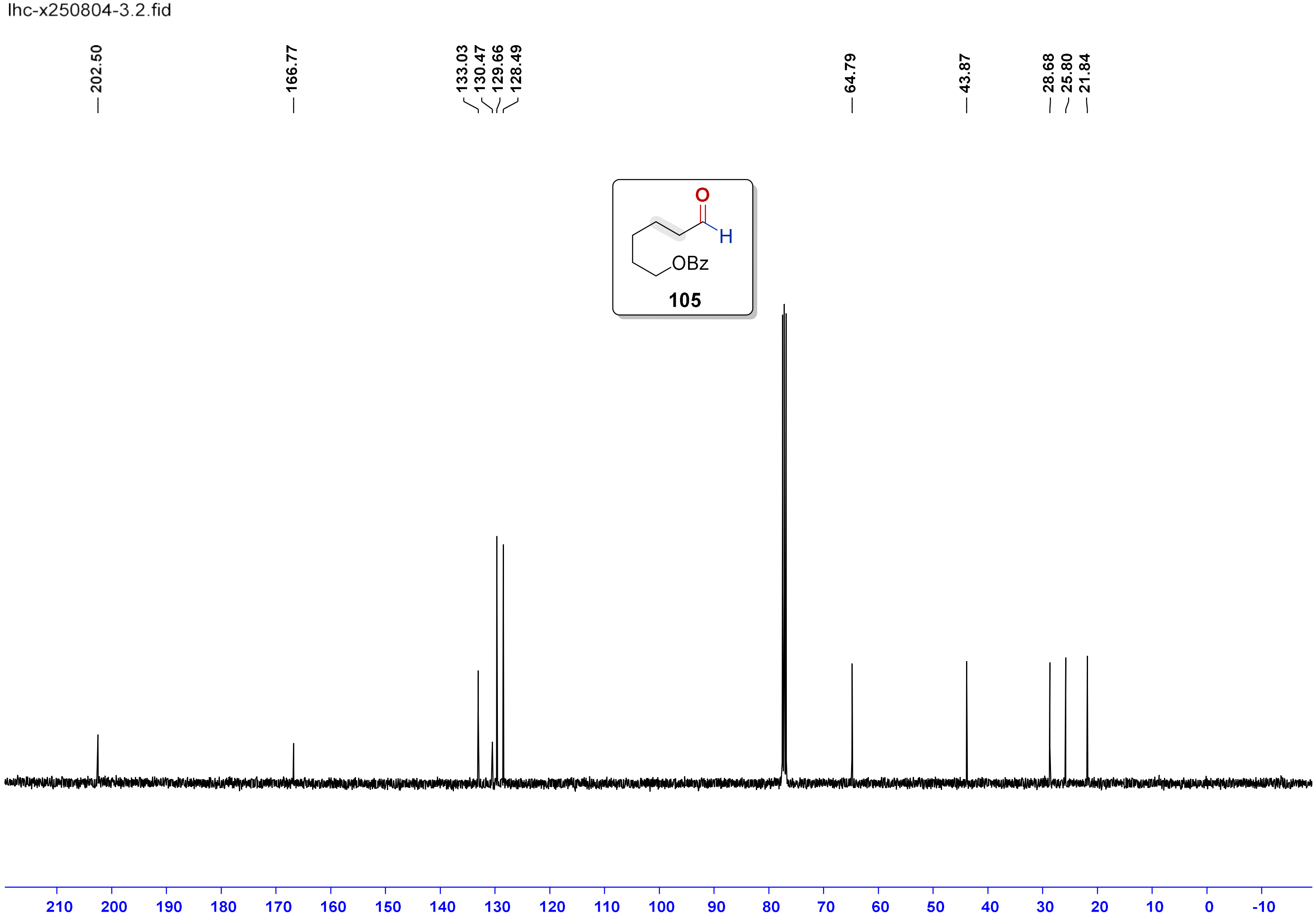

^{1}H NMR spectra for 106

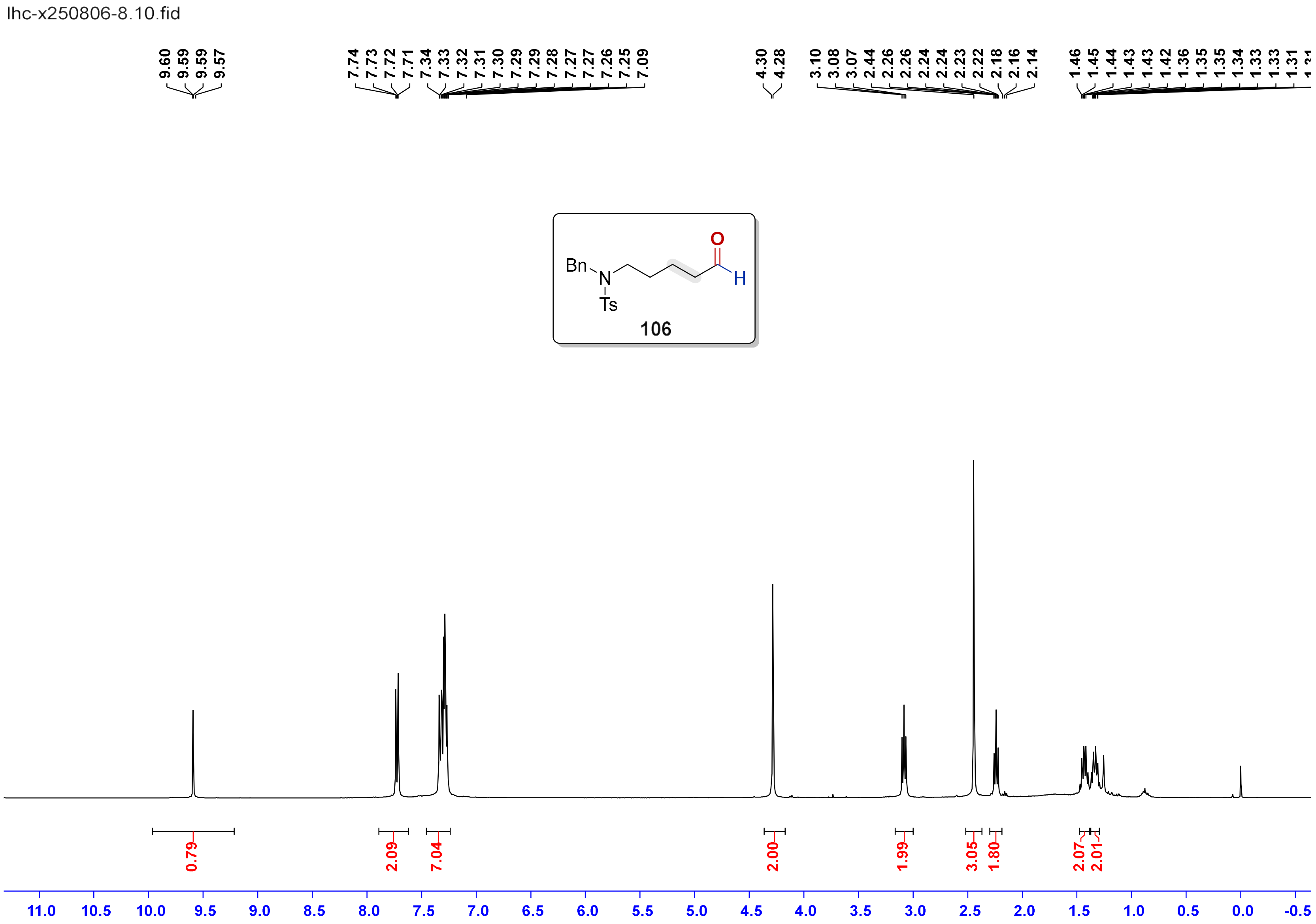

^{13}C NMR spectra for 106

lhc-x250812-2.2.fid

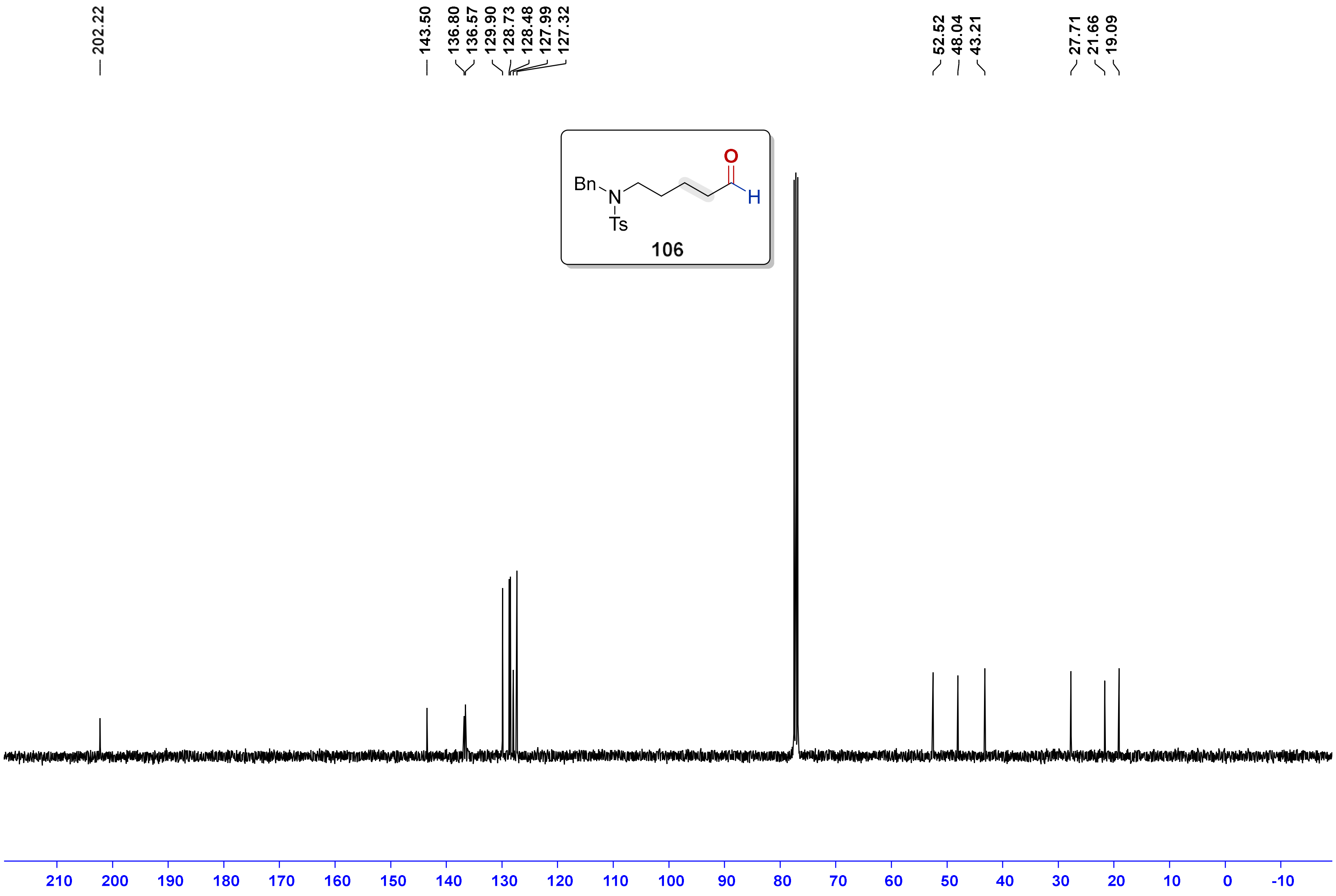

^{1}H NMR spectra for 107

lhc-x250731-3.1.fid

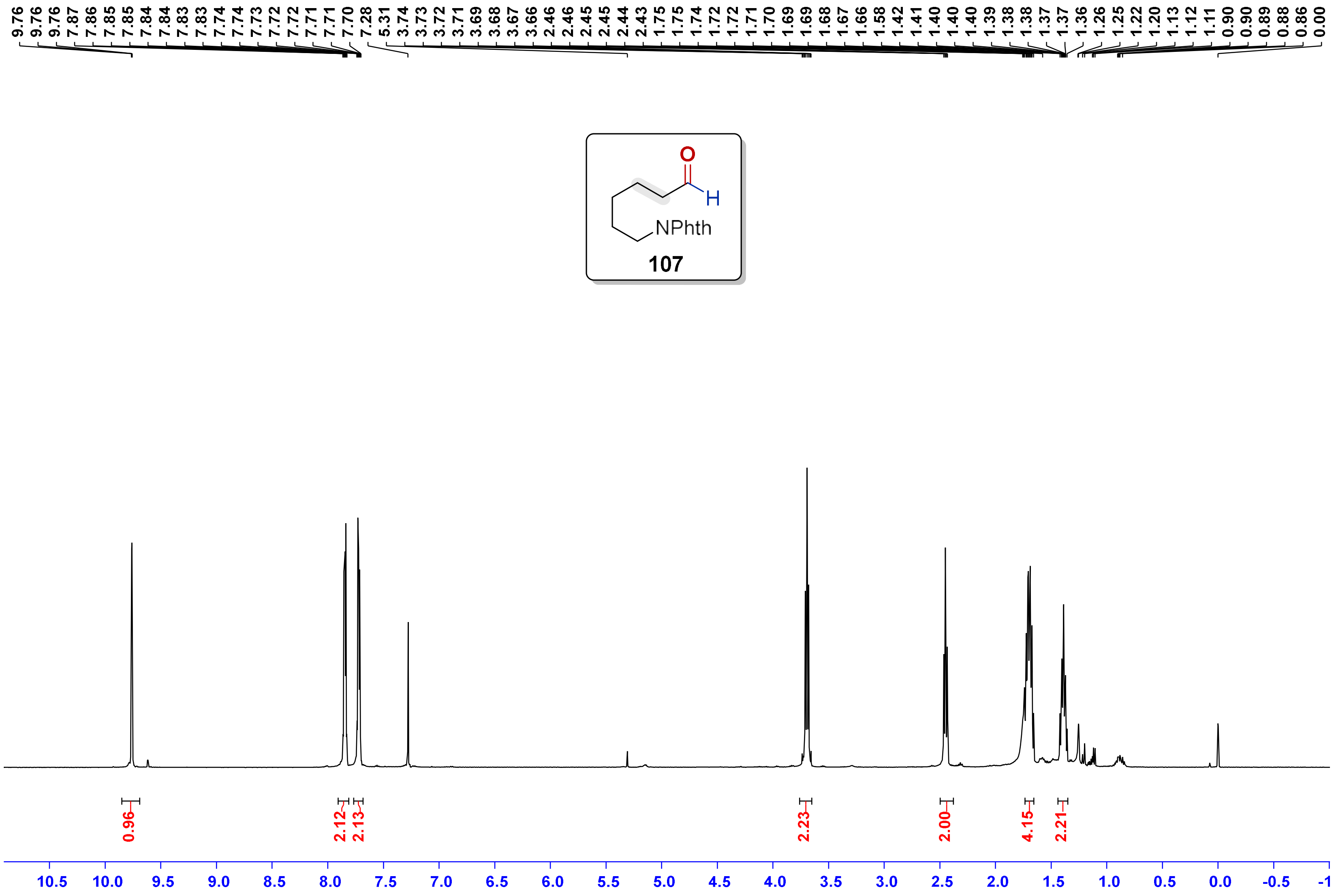

^{13}C NMR spectra for 107

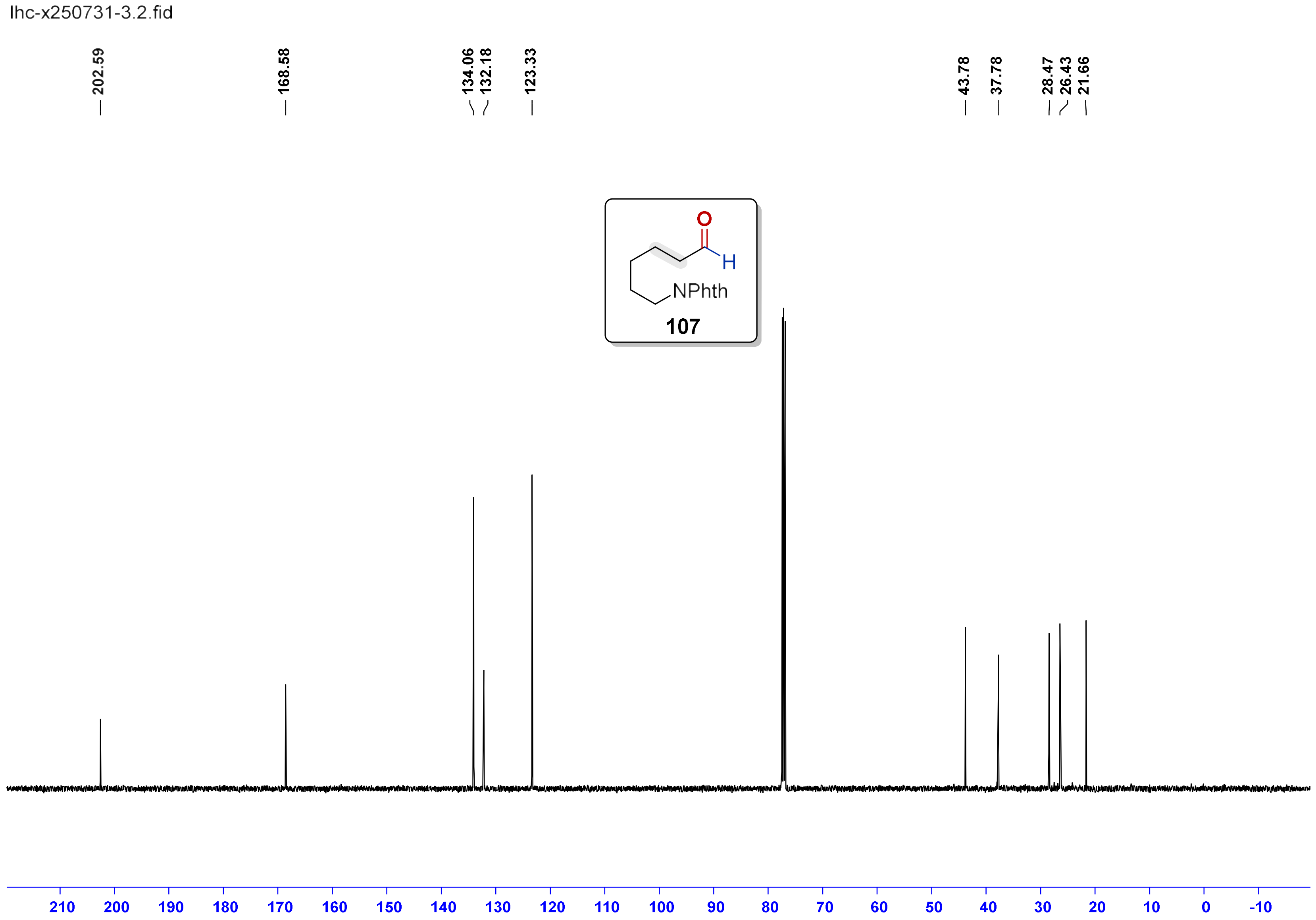

^{1}H NMR spectra for 108

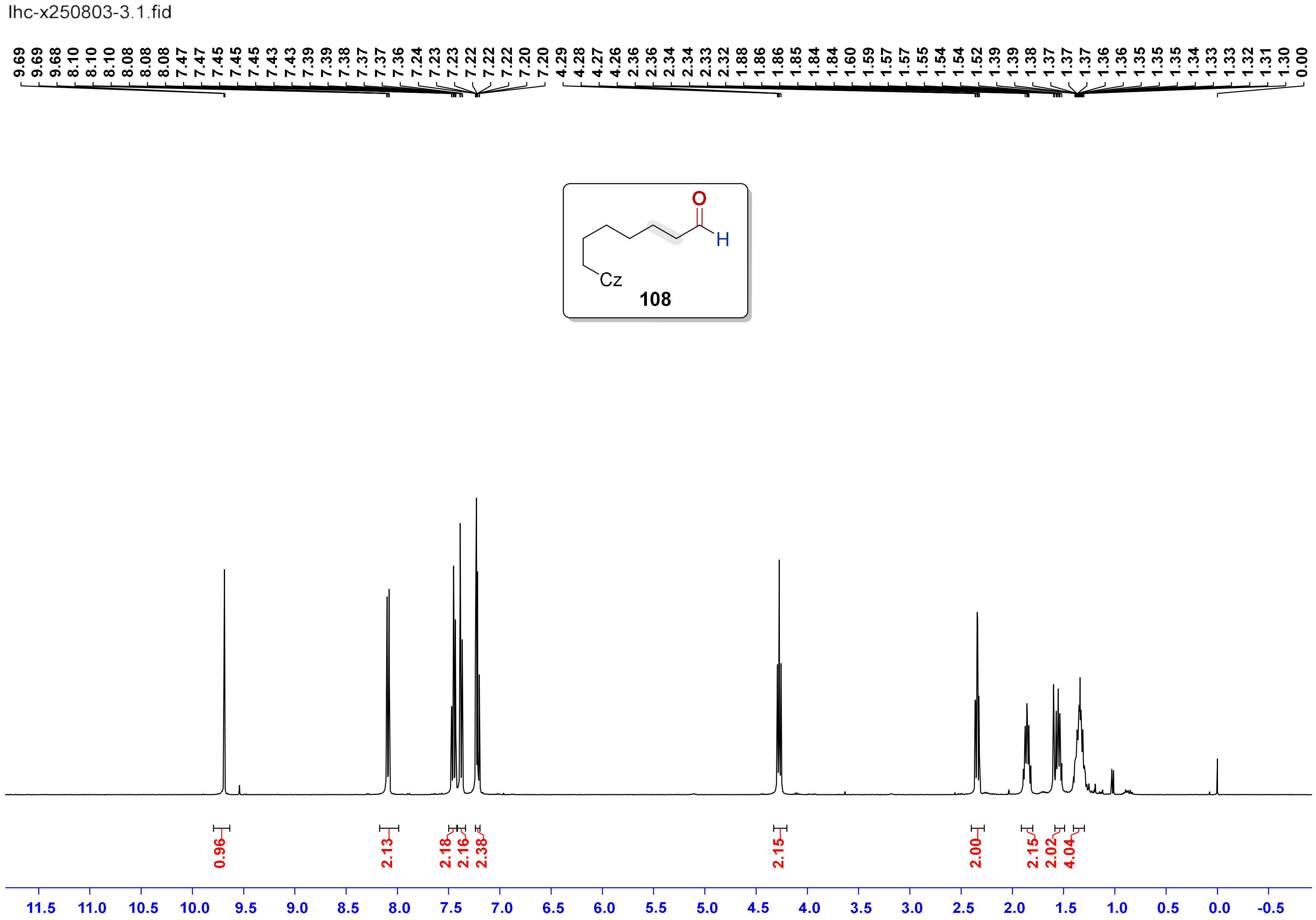

^{13}C NMR spectra for 108

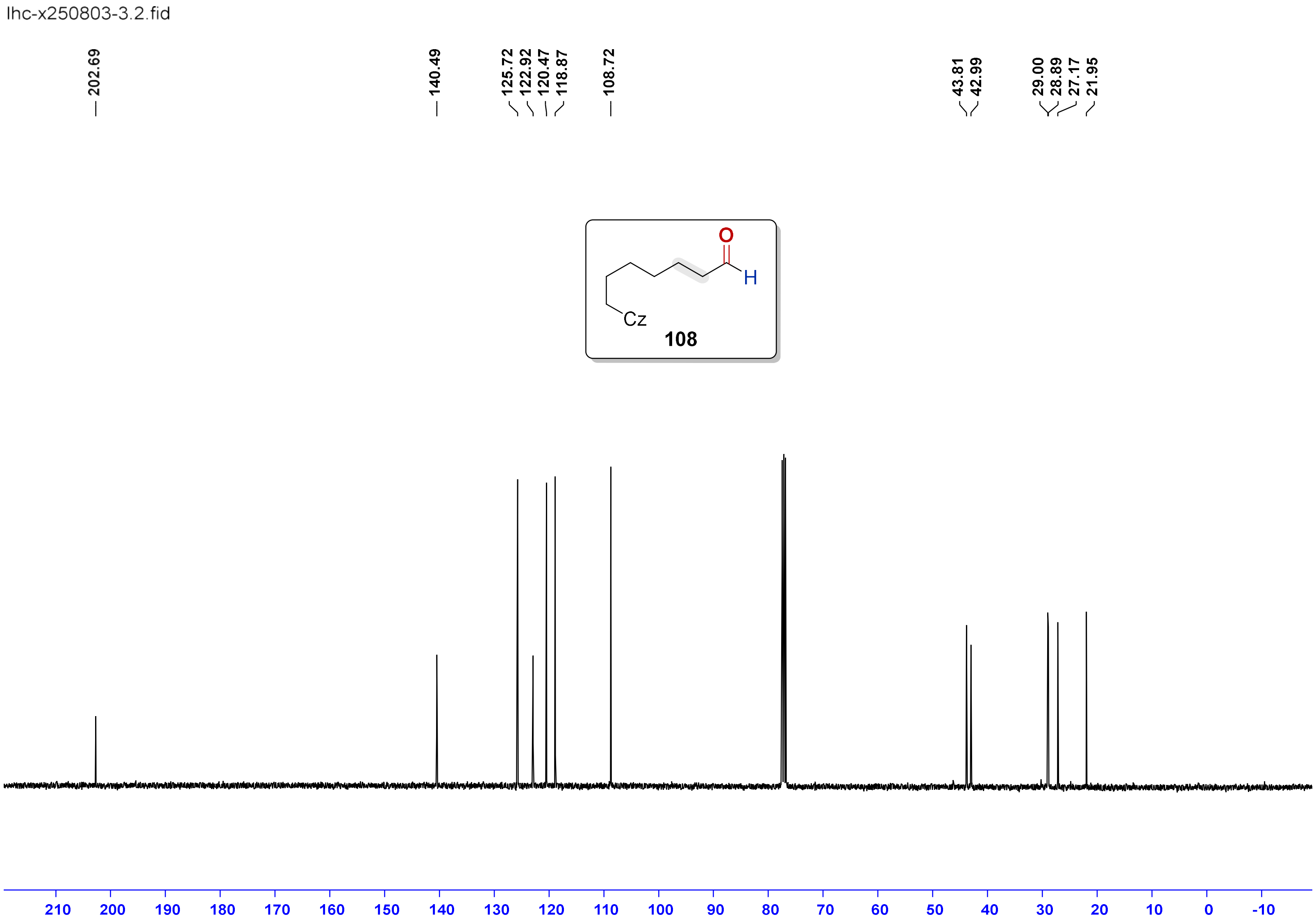

^{1}H NMR spectra for 109

lhc-x250730-2.1.fid

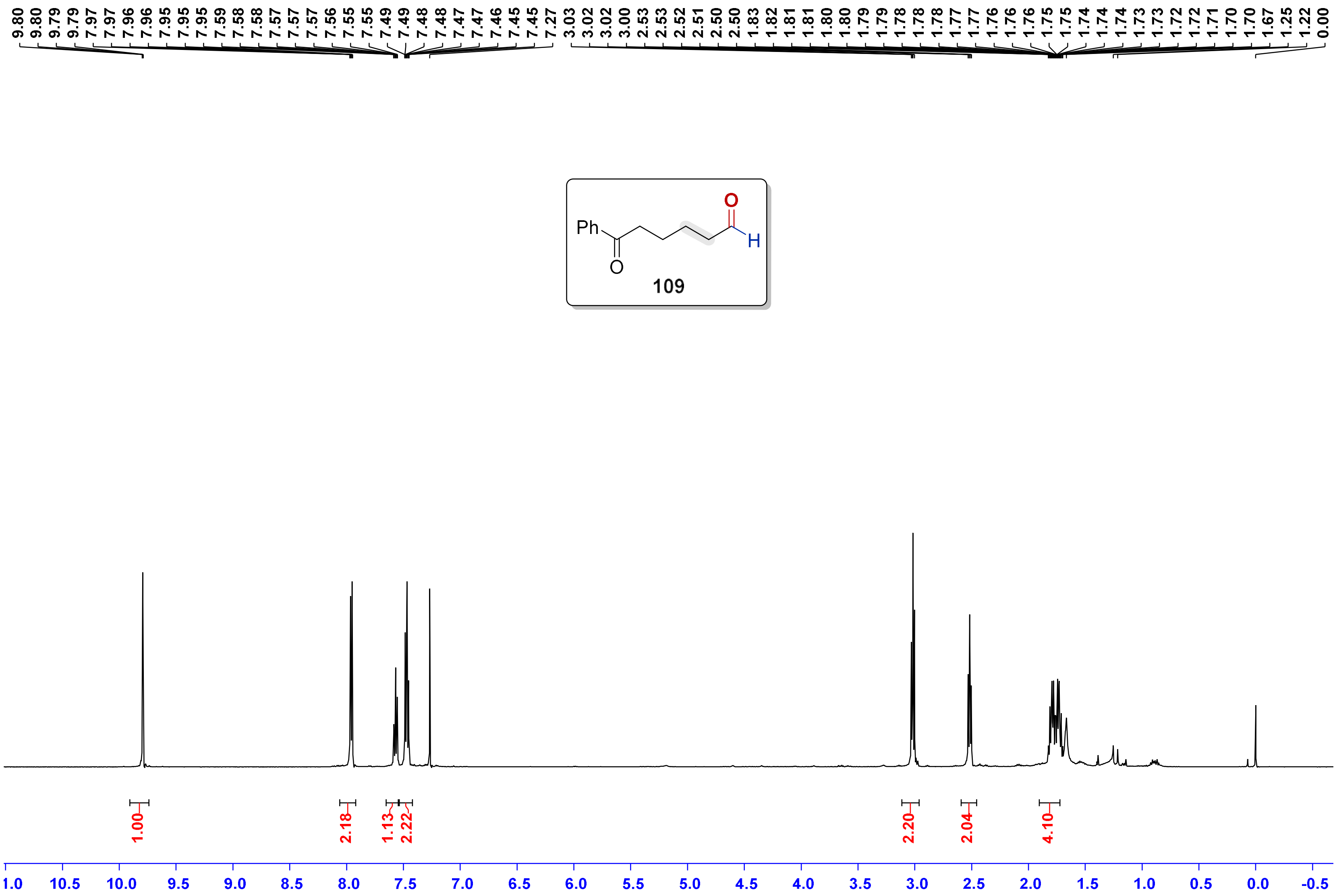

^{13}C NMR spectra for 109

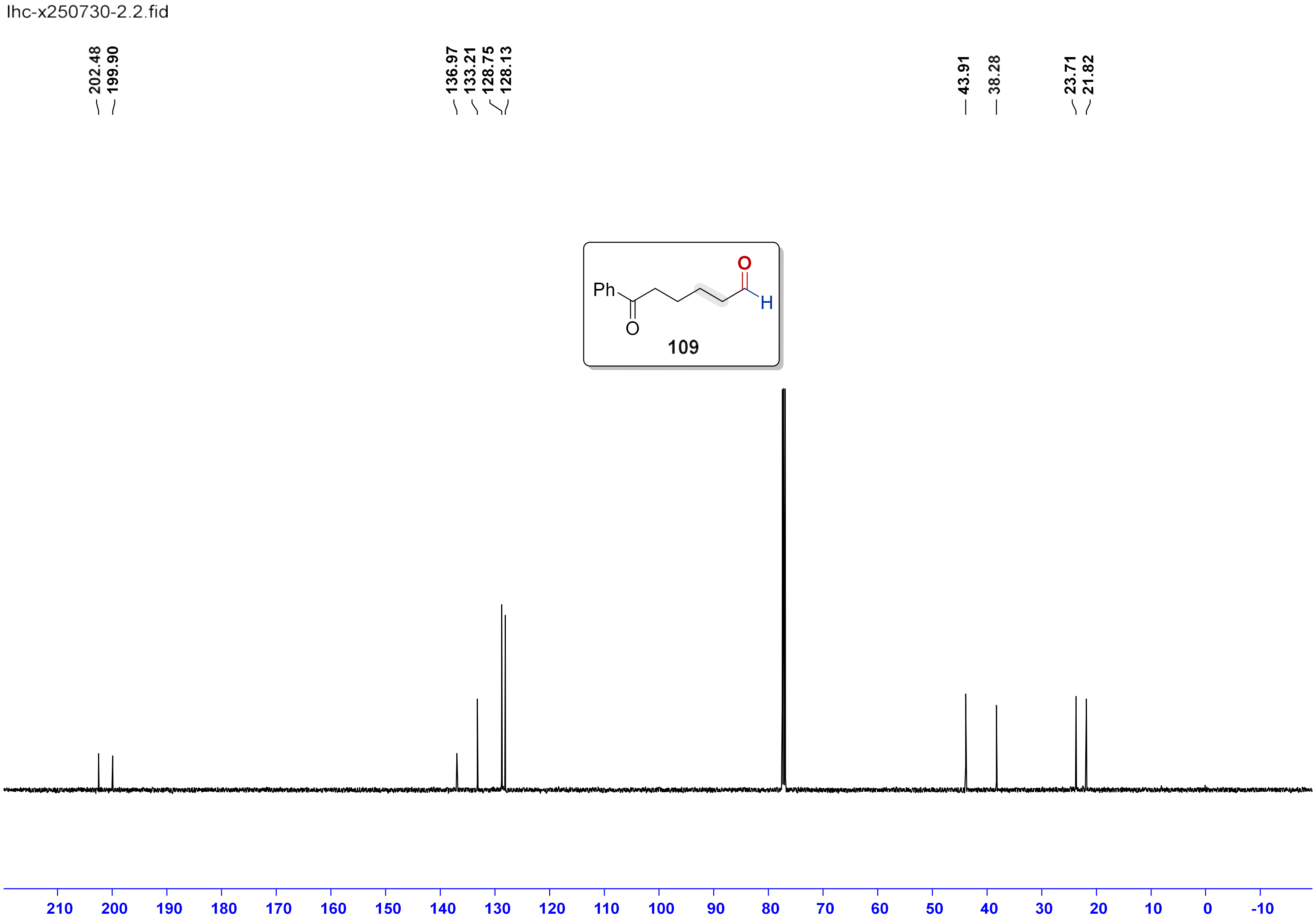

^{1}H NMR spectra for 110

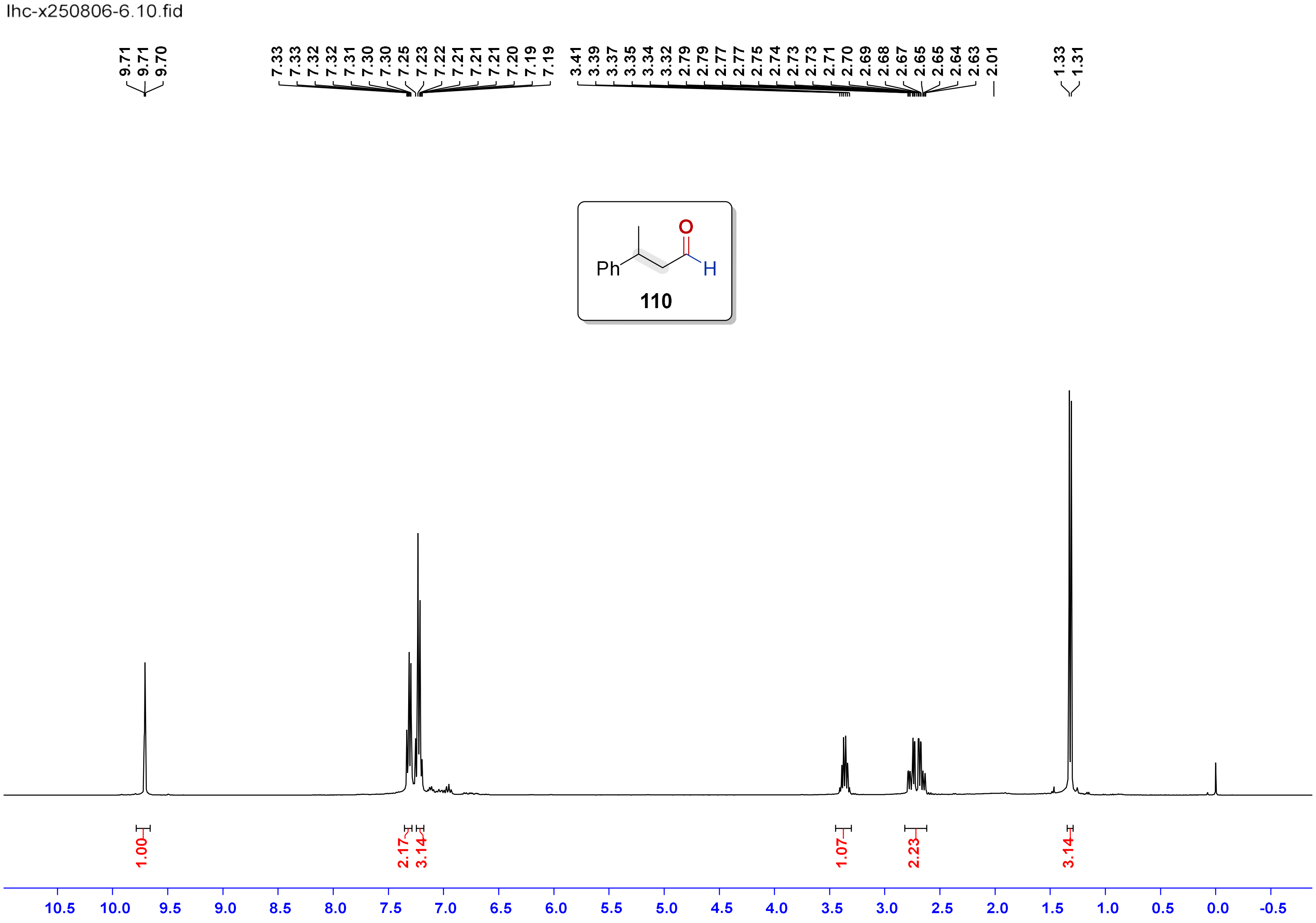

^{13}C NMR spectra for 110

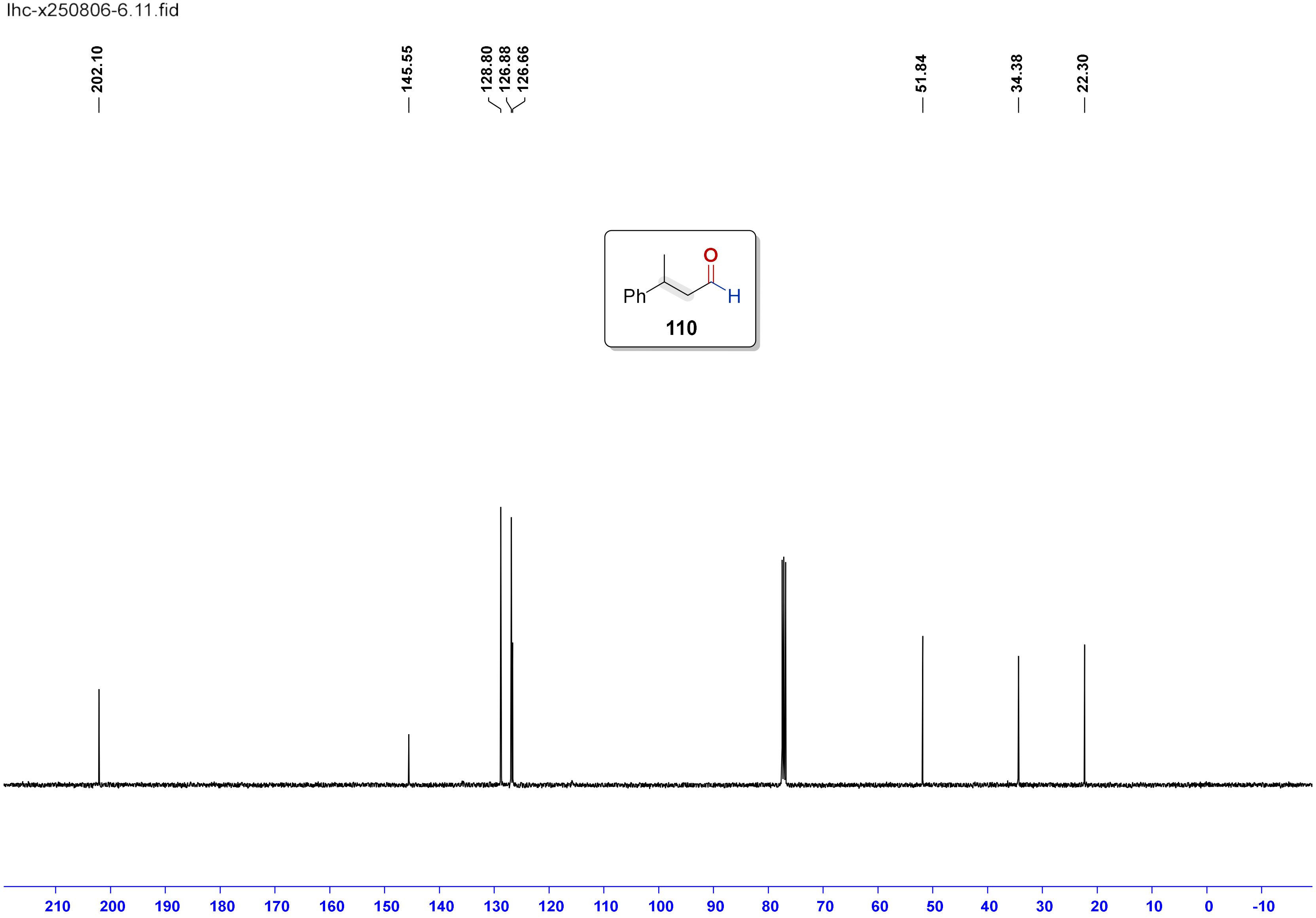

^{1}H NMR spectra for 111

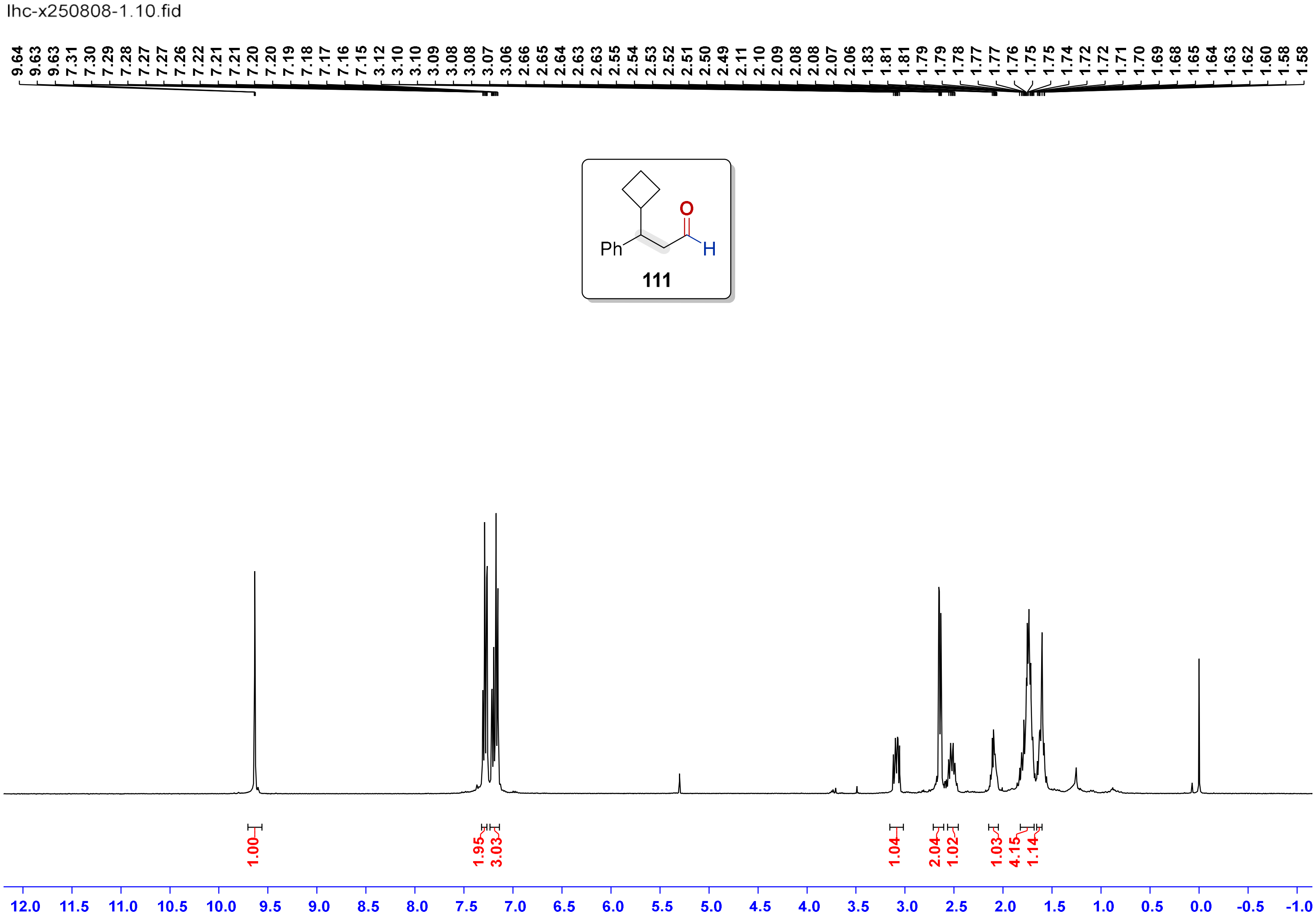

^{13}C NMR spectra for 111

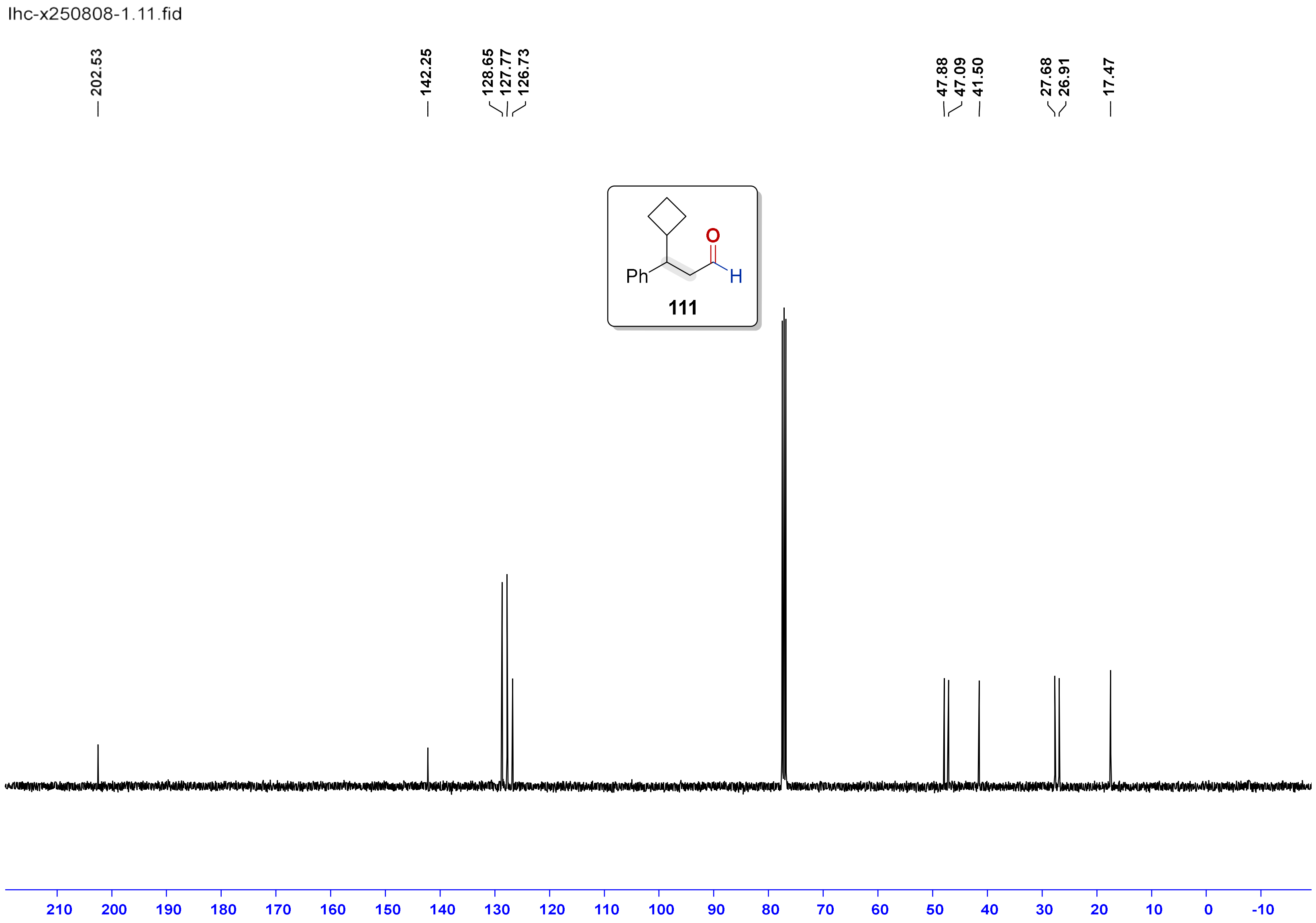

^{1}H NMR spectra for 112

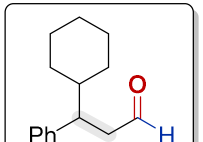

112

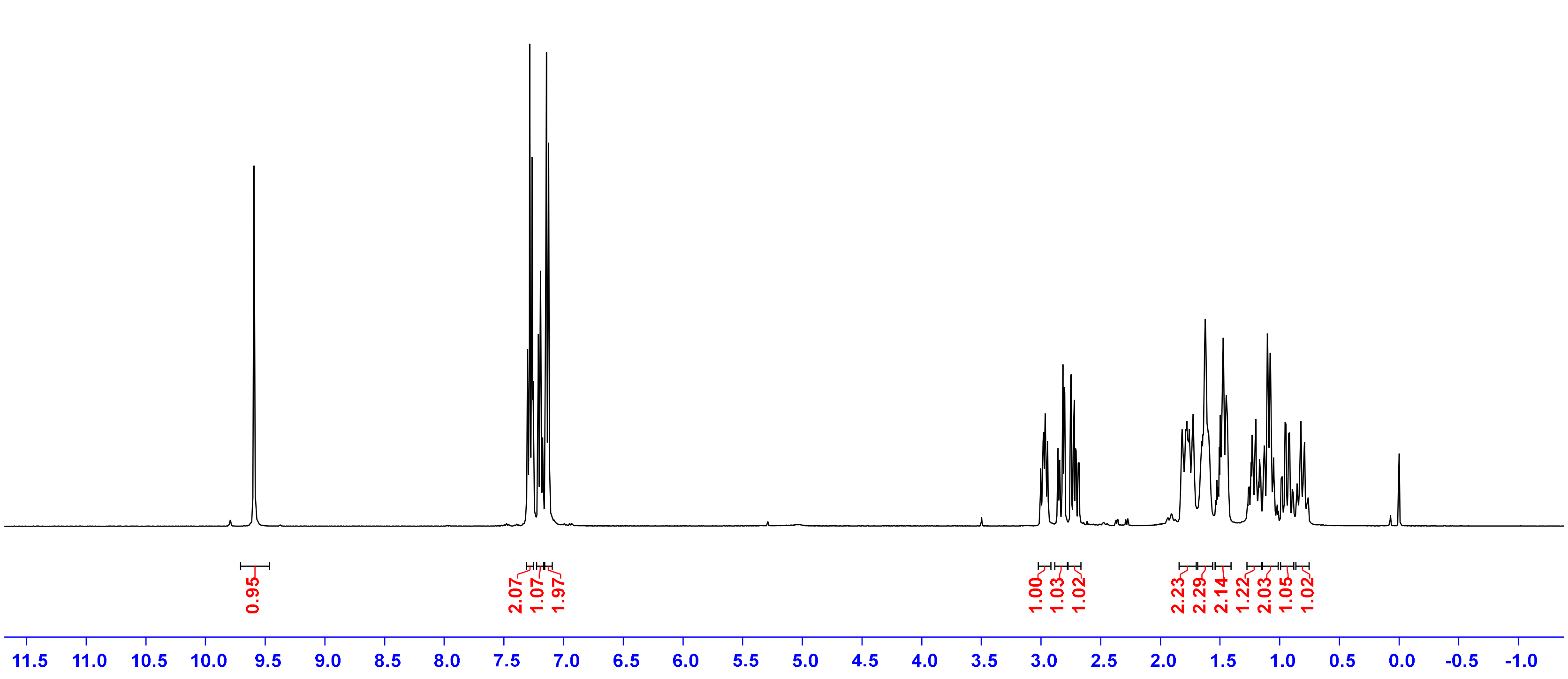

^{13}C NMR spectra for 112

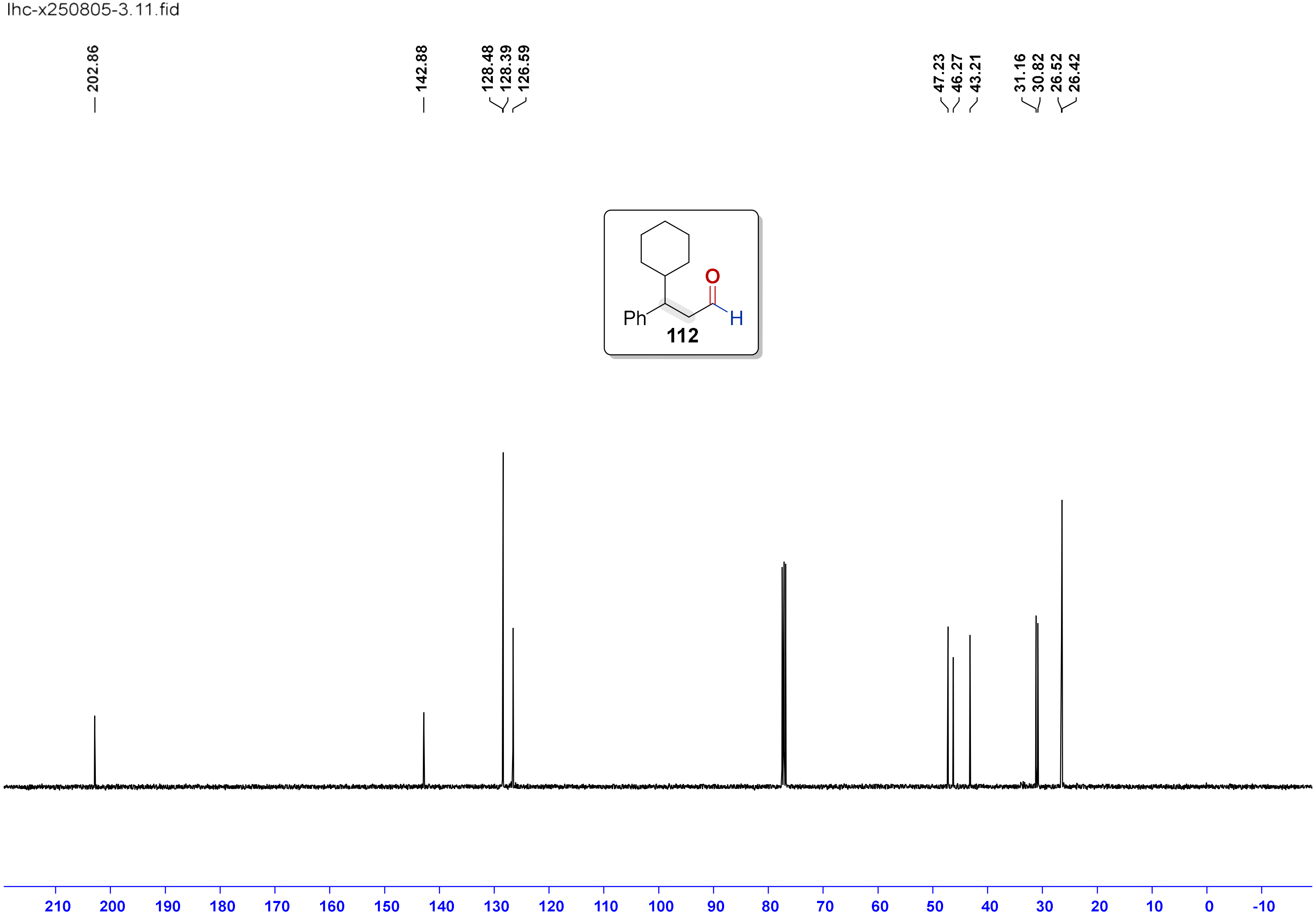

112

^{1}H NMR spectra for 113

lhc-x250808-2.10.fid

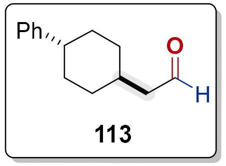

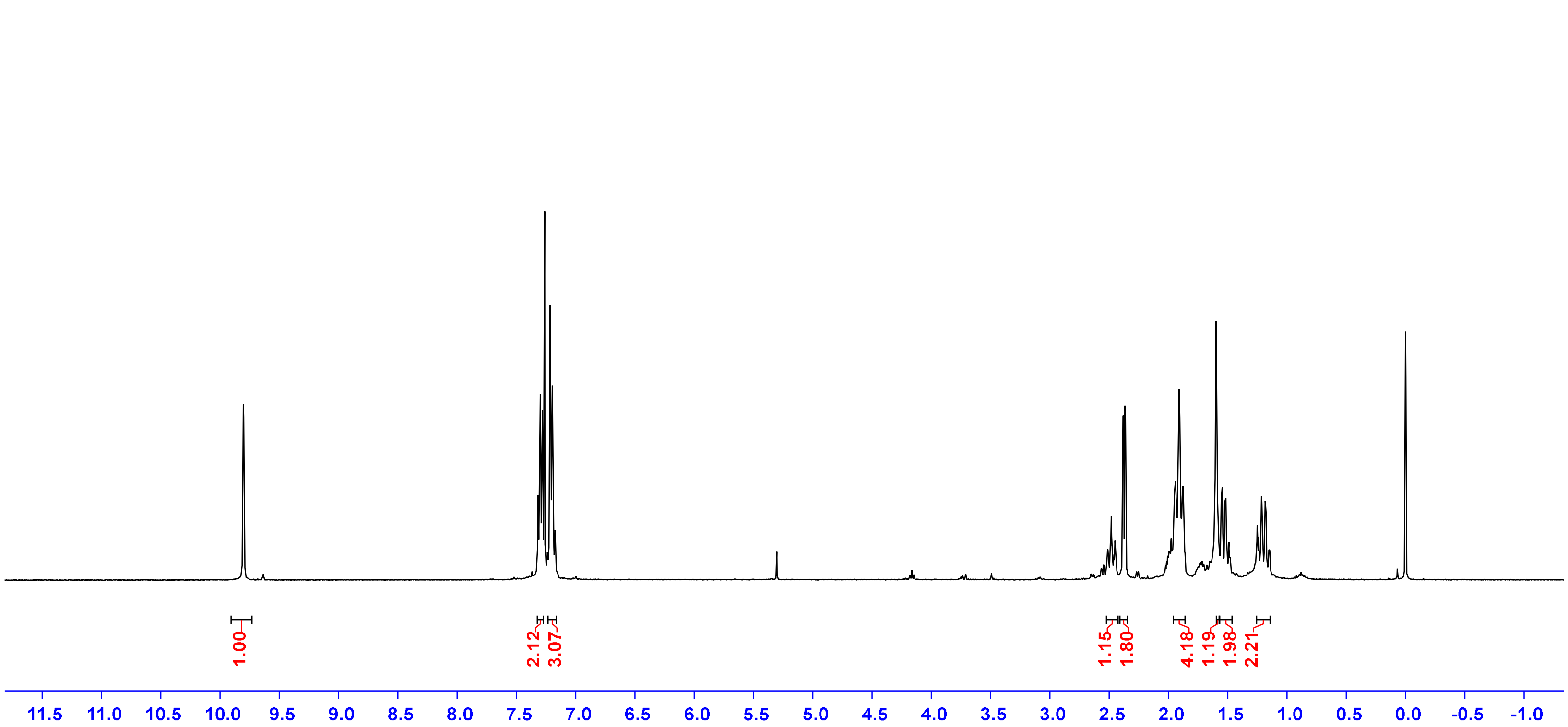

^{13}C NMR spectra for 113

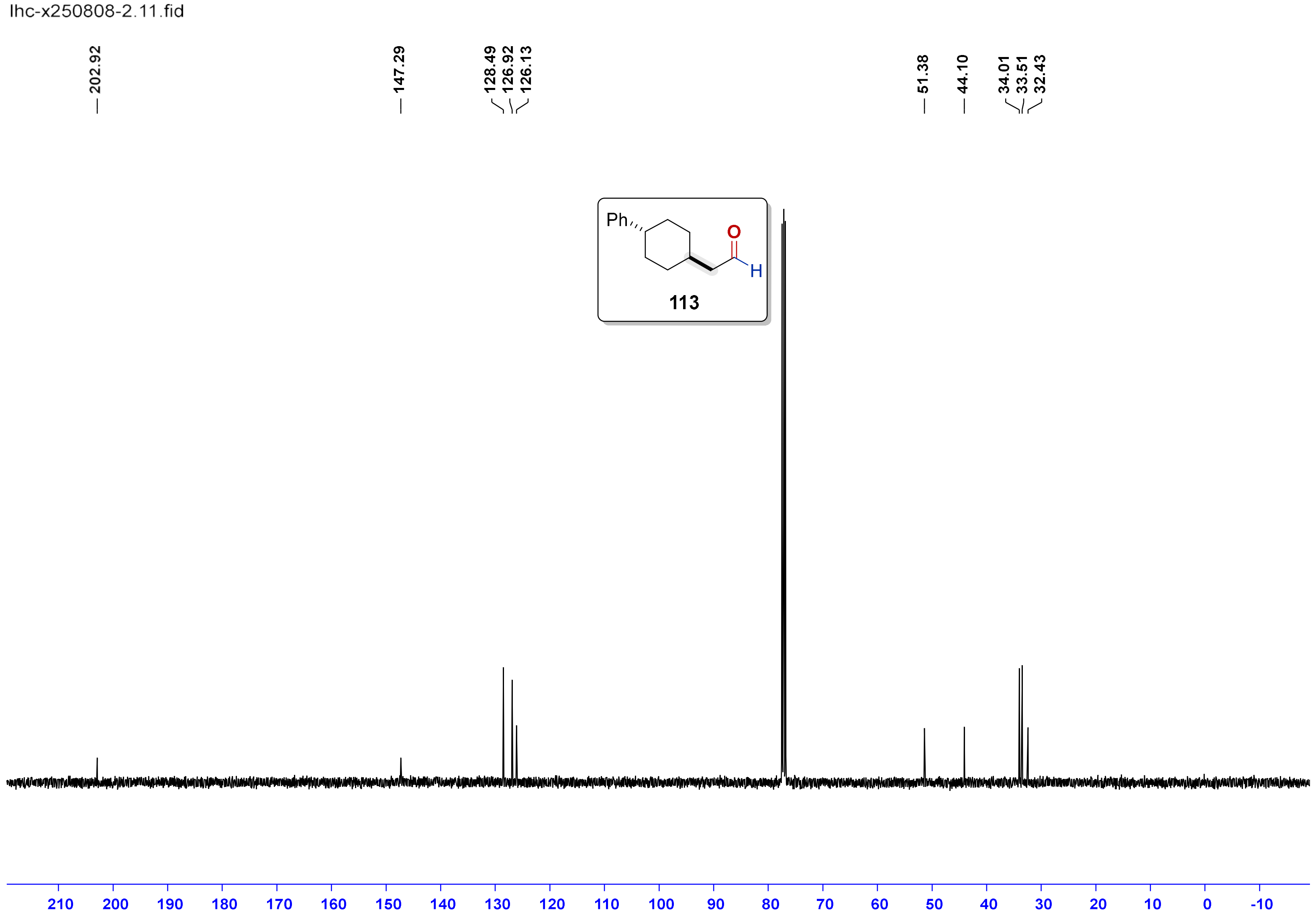

^{1}H NMR spectra for 114

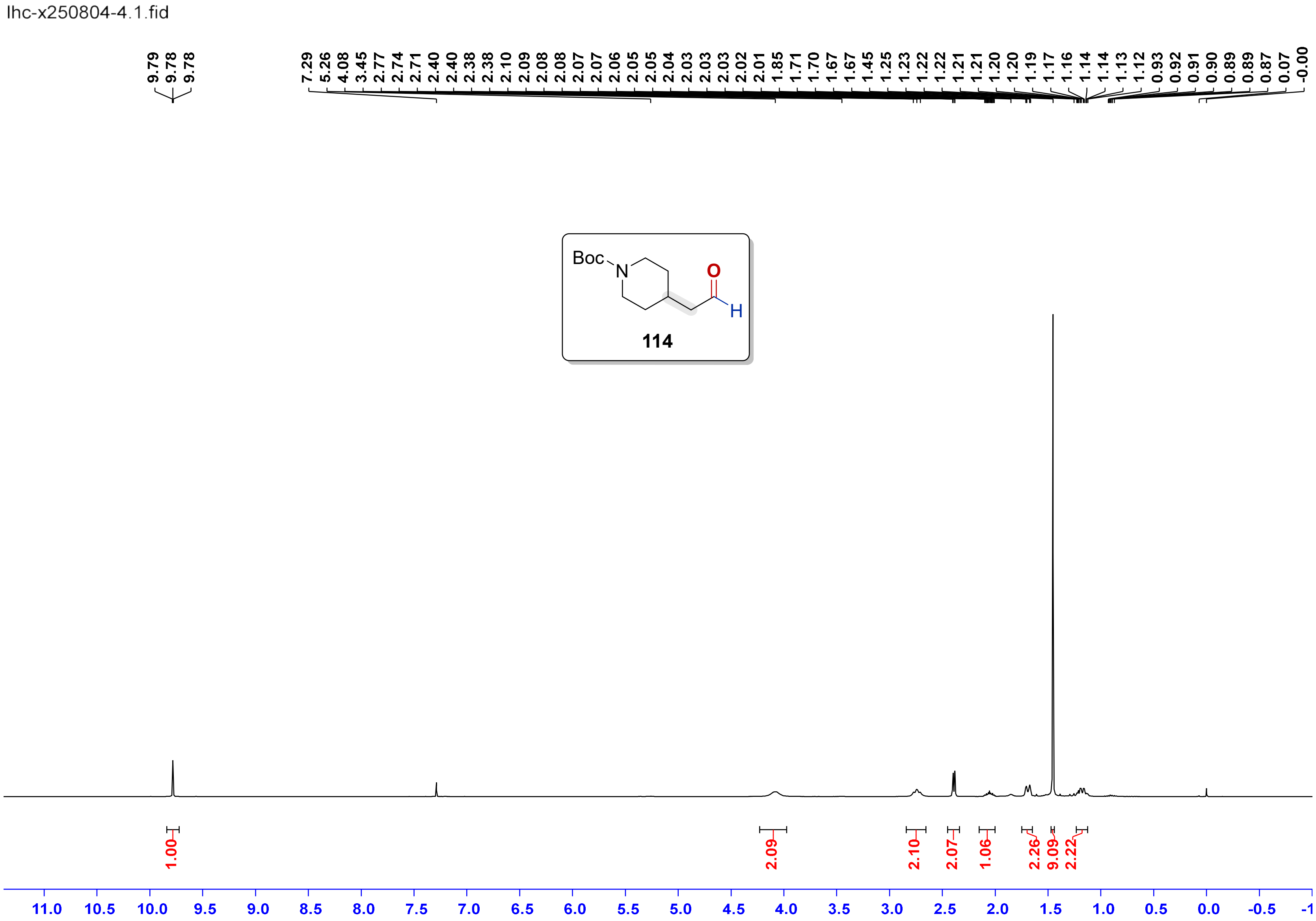

^{13}C NMR spectra for 114

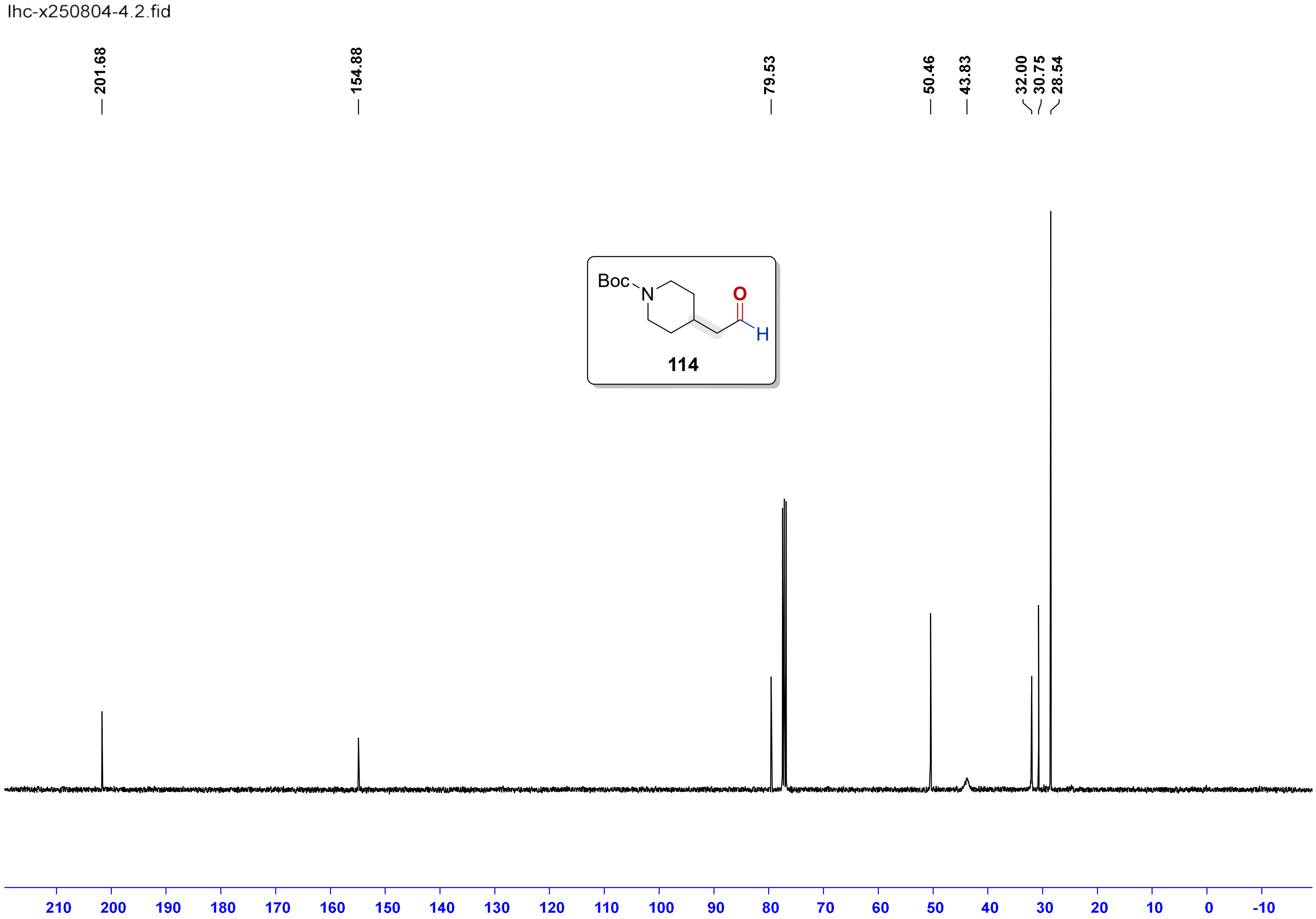

^{1}H NMR spectra for 115a

lhc-x250806-4.10.fid

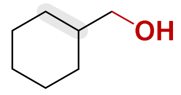

115a

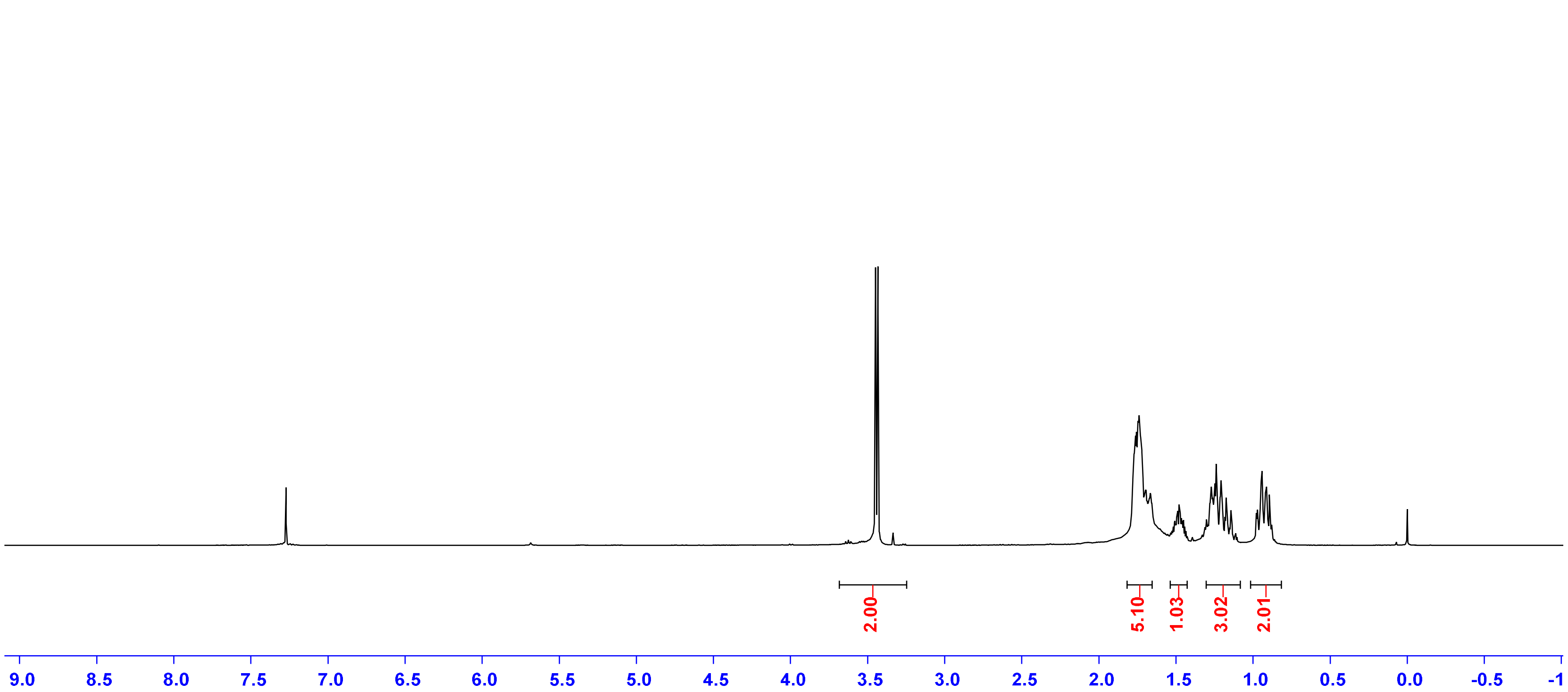

^{13}C NMR spectra for 115a

lhc-x250806-4.11.fid

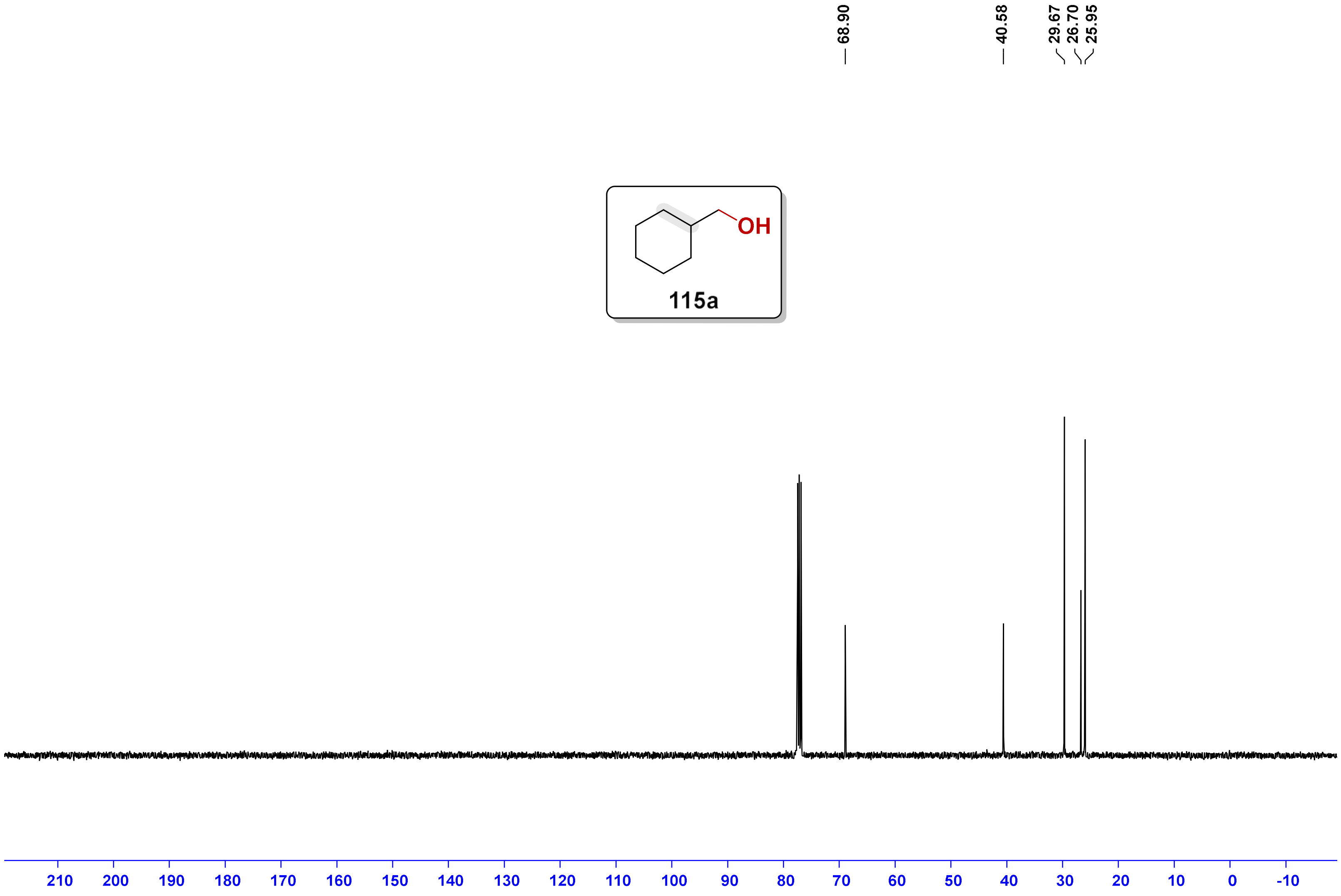

^{1}H NMR spectra for 116

lhc-x250803-10.1.fid

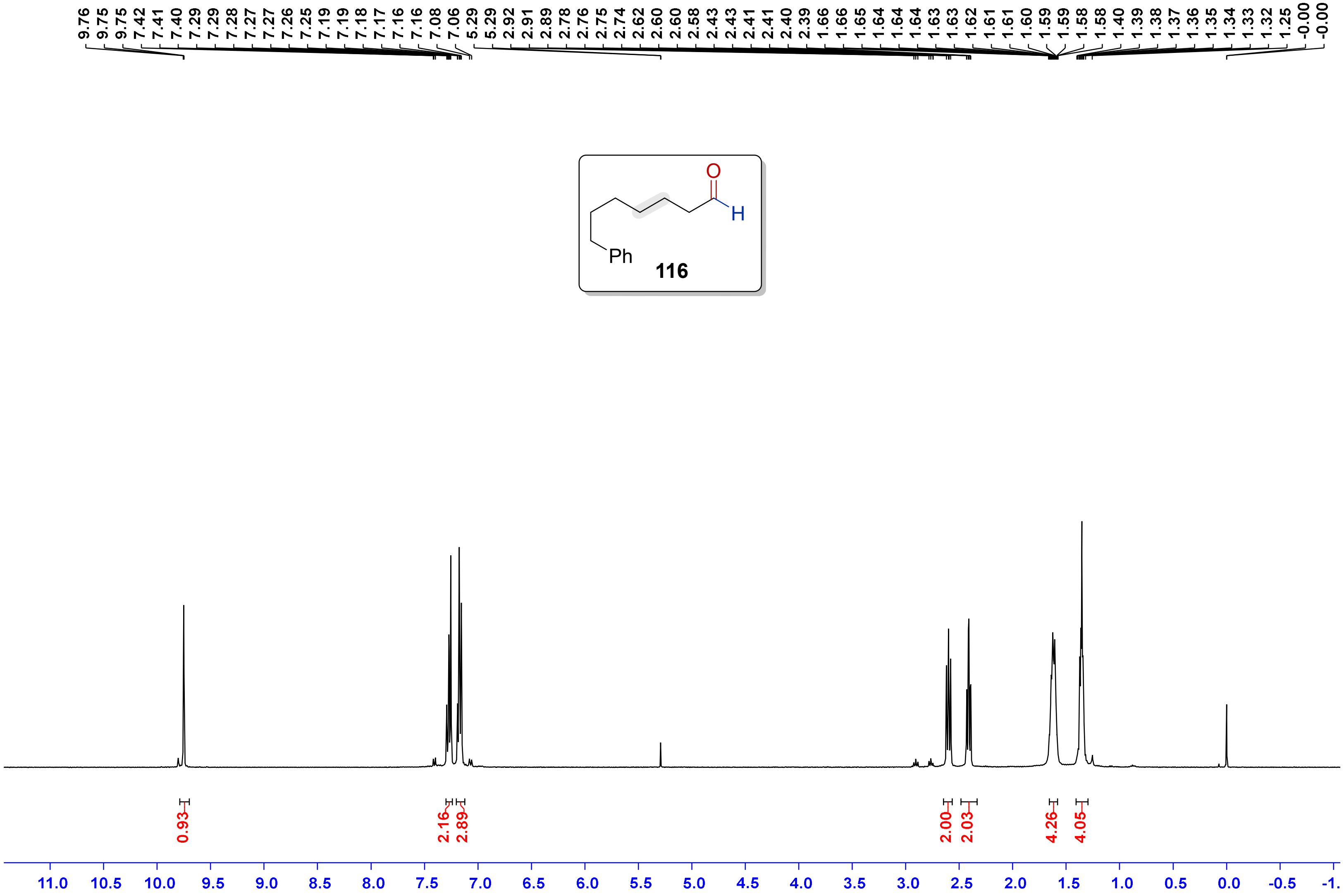

^{13}C NMR spectra for 116

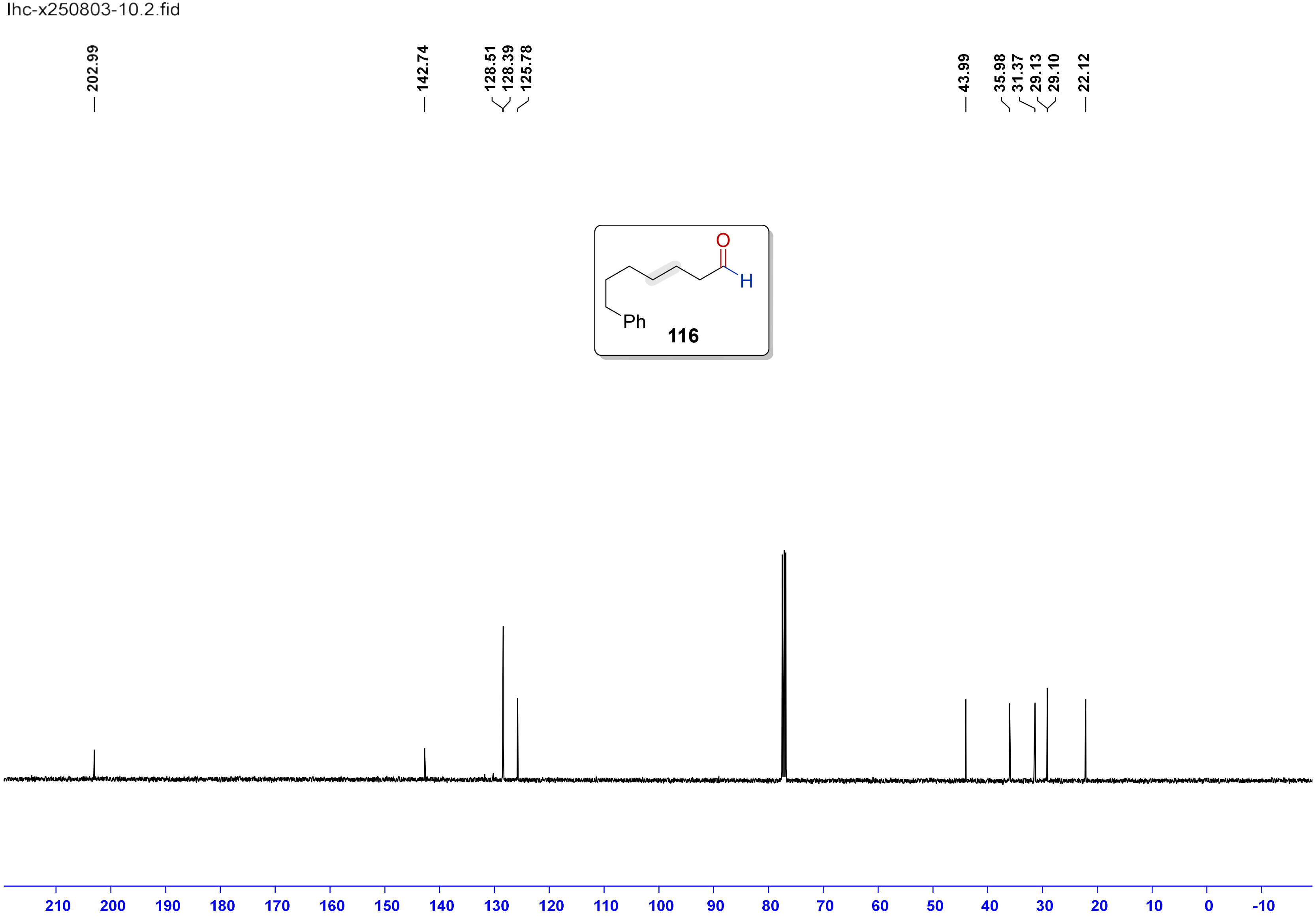

^{1}H NMR spectra for 117

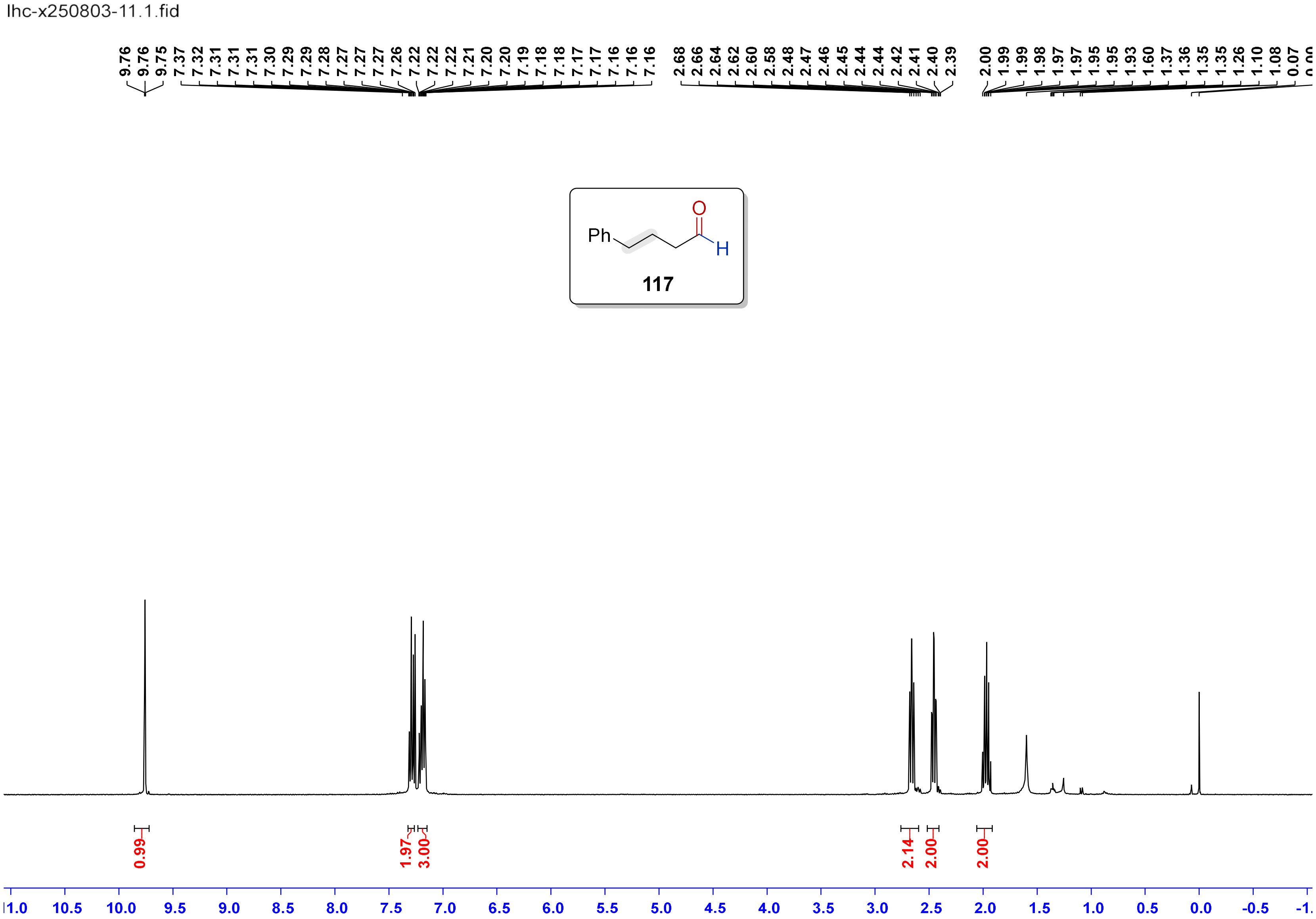

^{13}C NMR spectra for 117

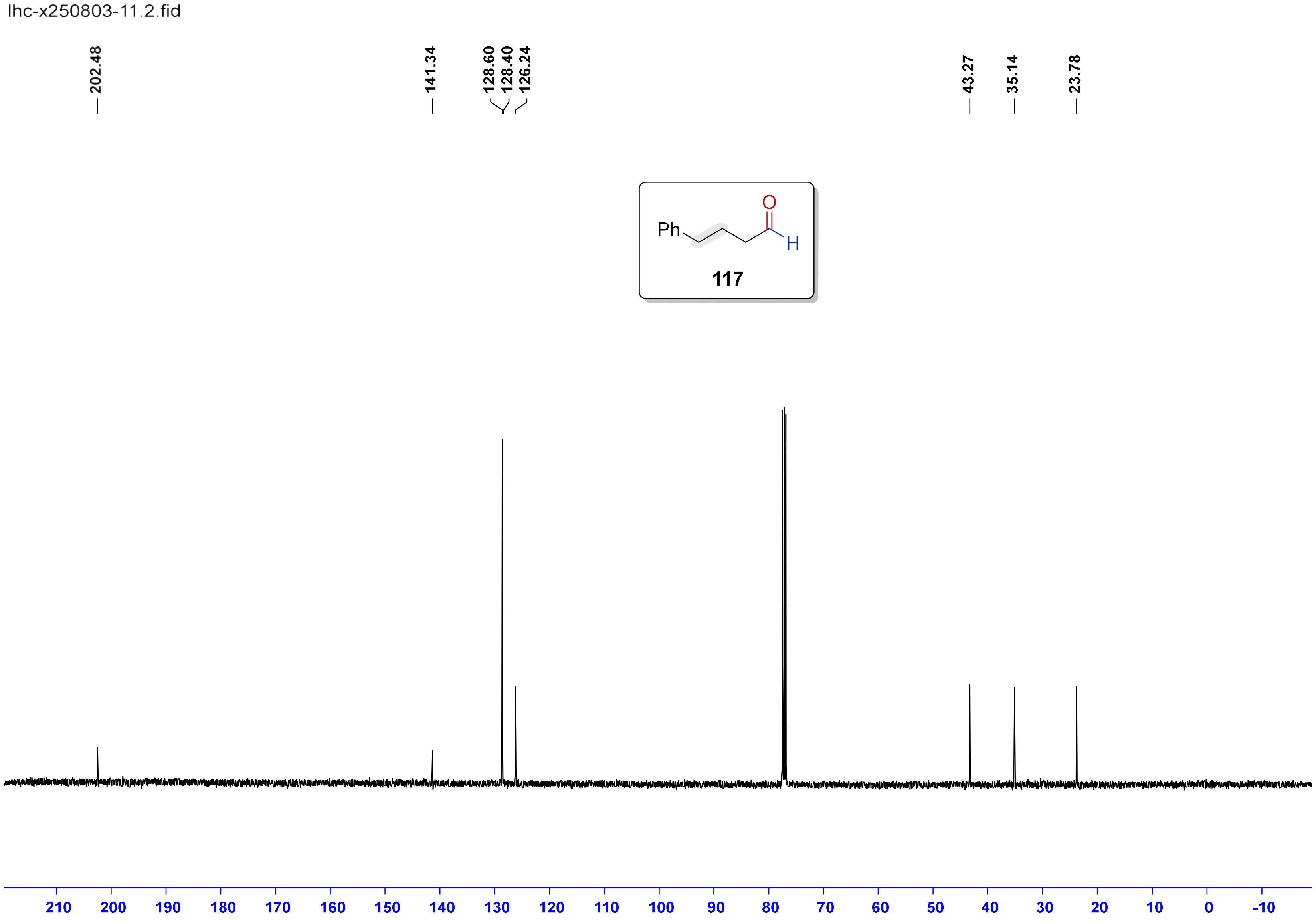

117

^{1}H NMR spectra for 118

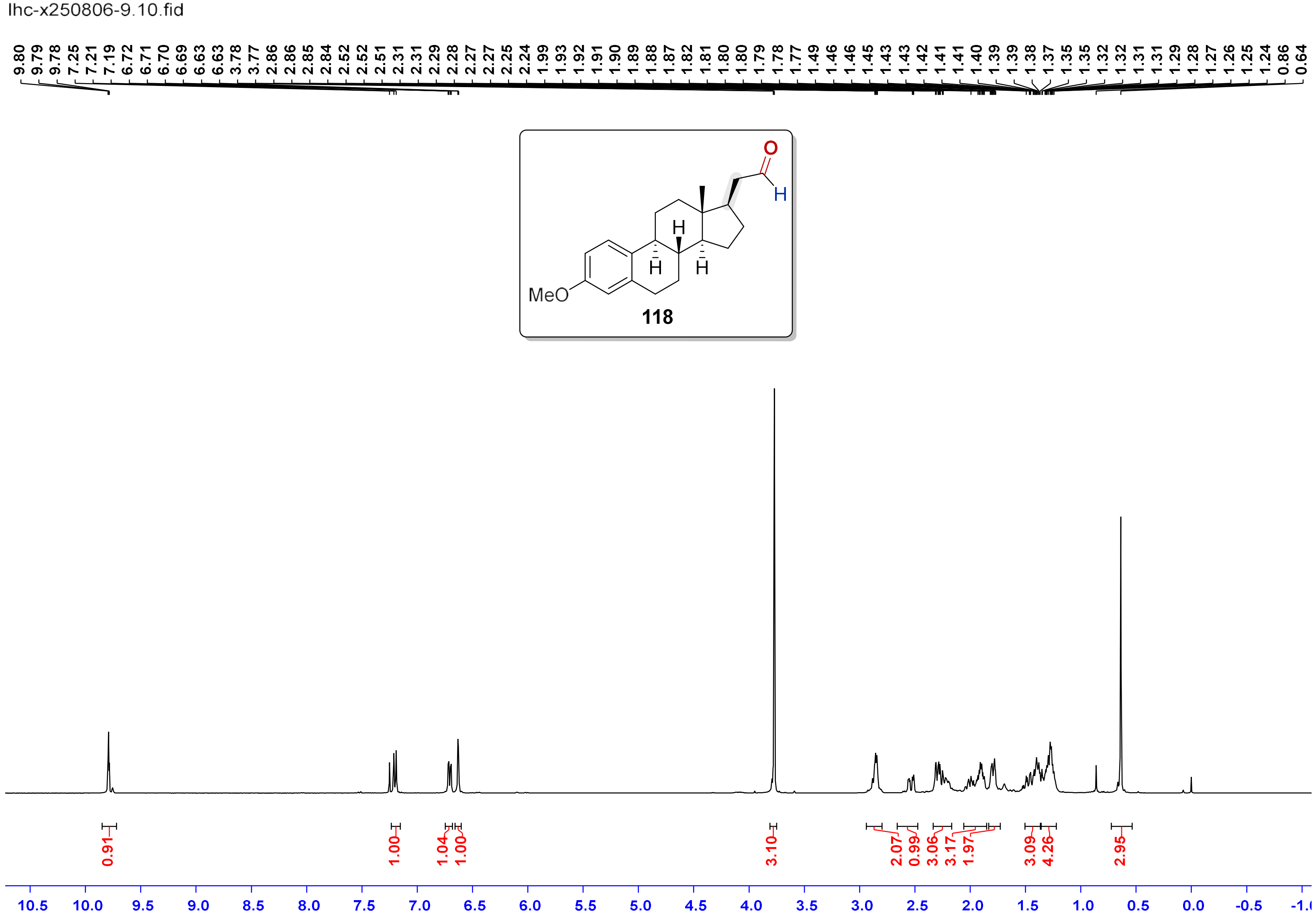

^{13}C NMR spectra for 118

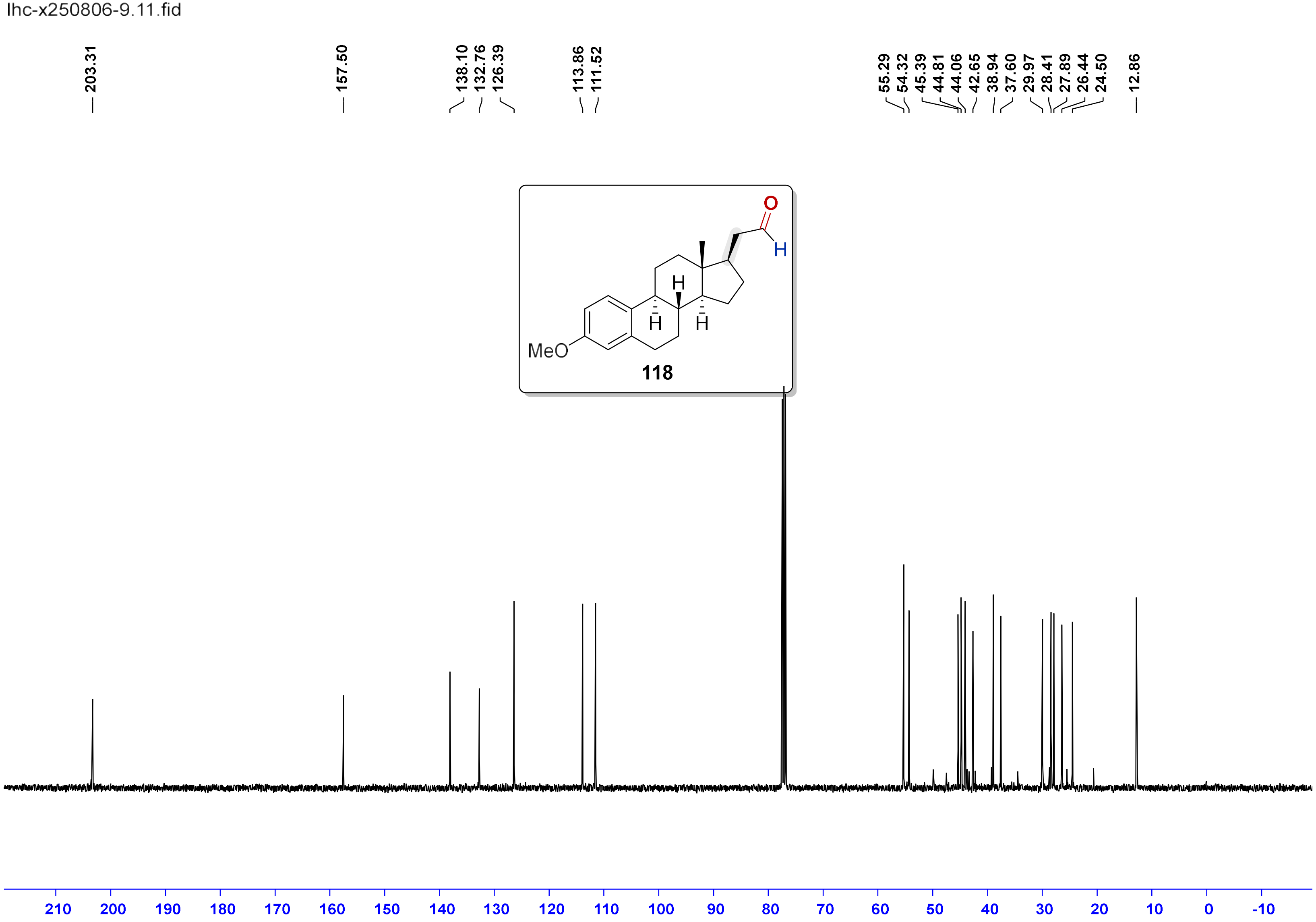

^{1}H NMR spectra for 120

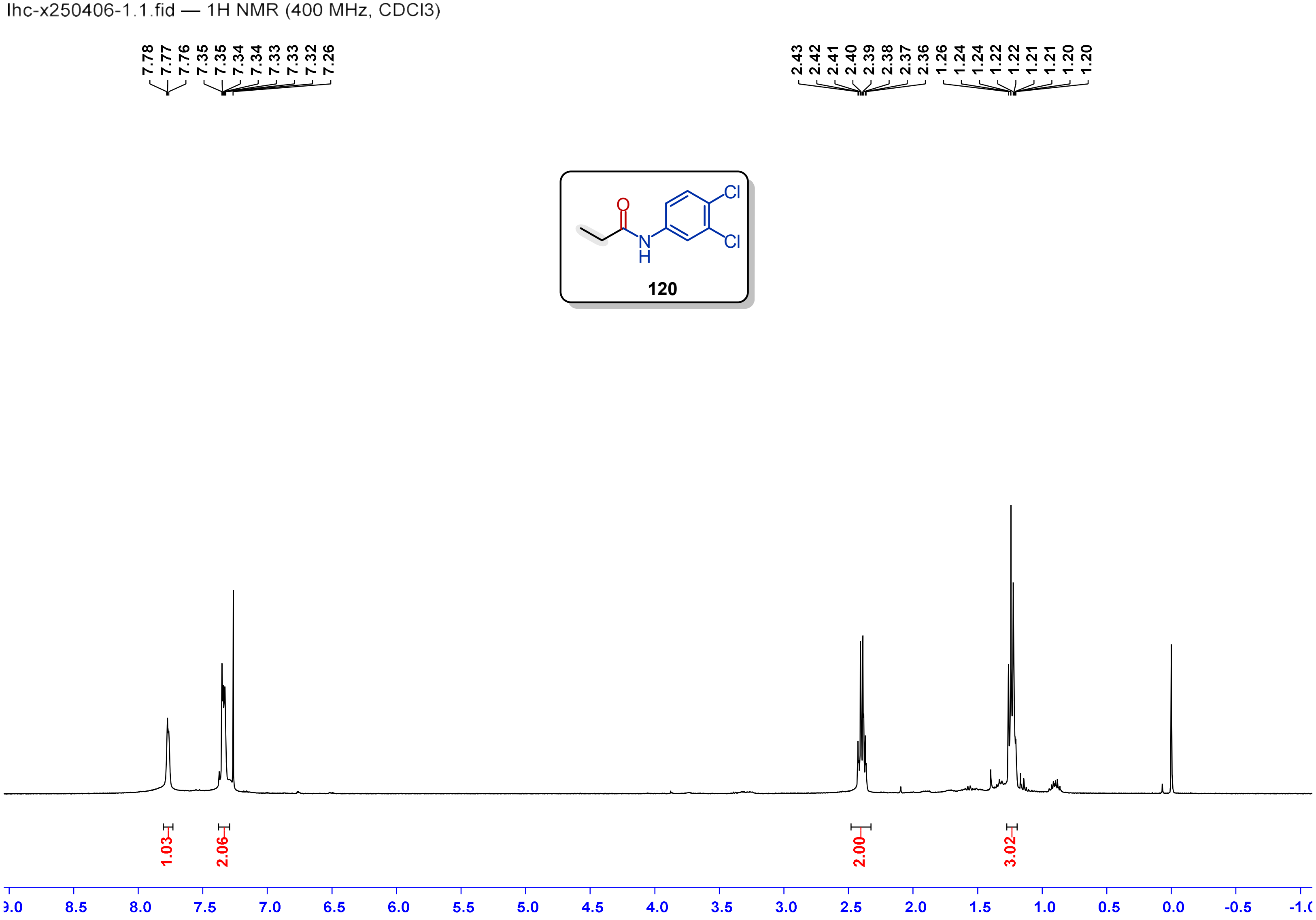

^{13}C NMR spectra for 120

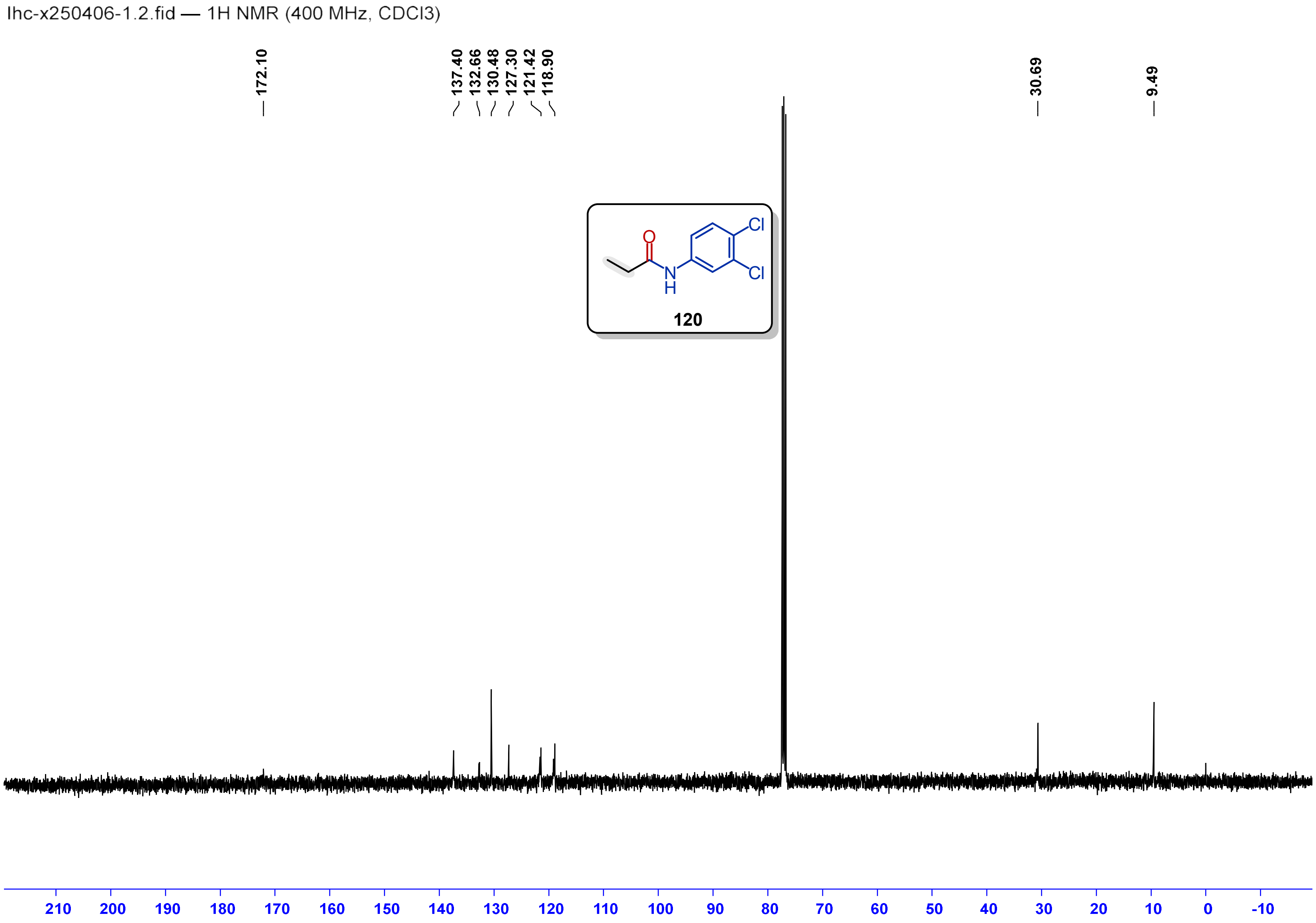

^{1}H NMR spectra for 121

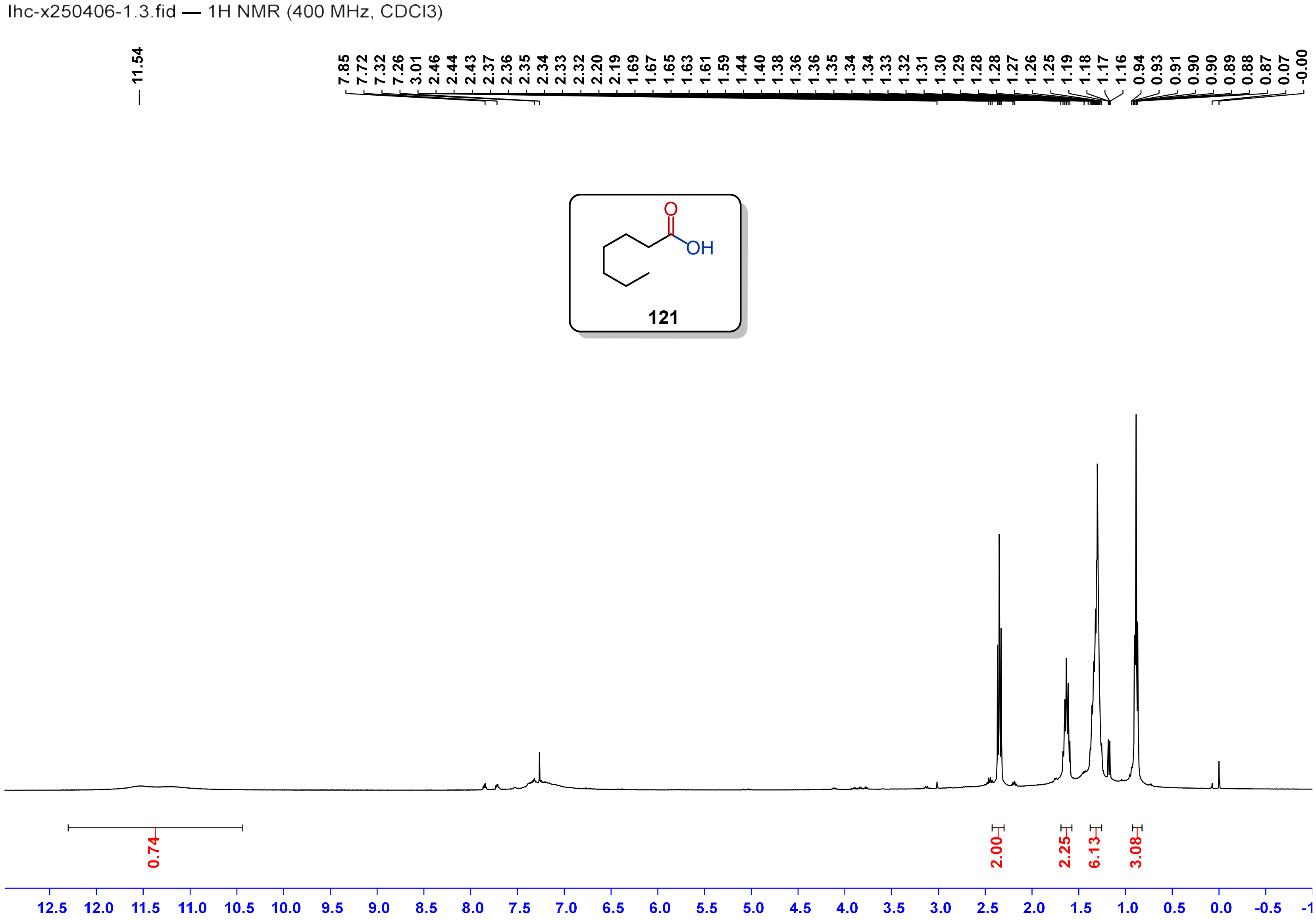

^{13}C NMR spectra for 121

lhc-x250406-1.4.fid — 1H NMR (400 MHz, CDCl3)

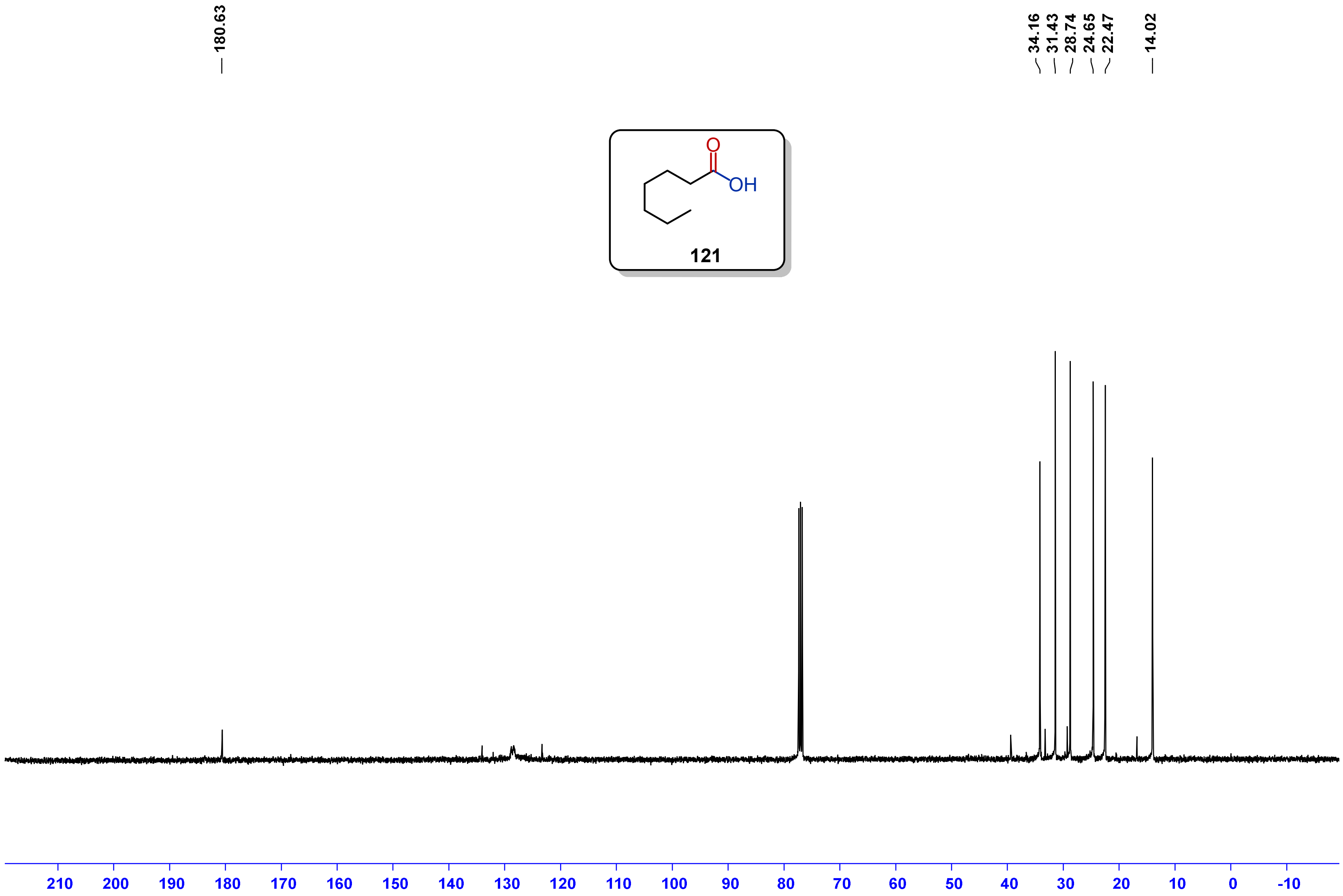

^{1}H NMR spectra for 122

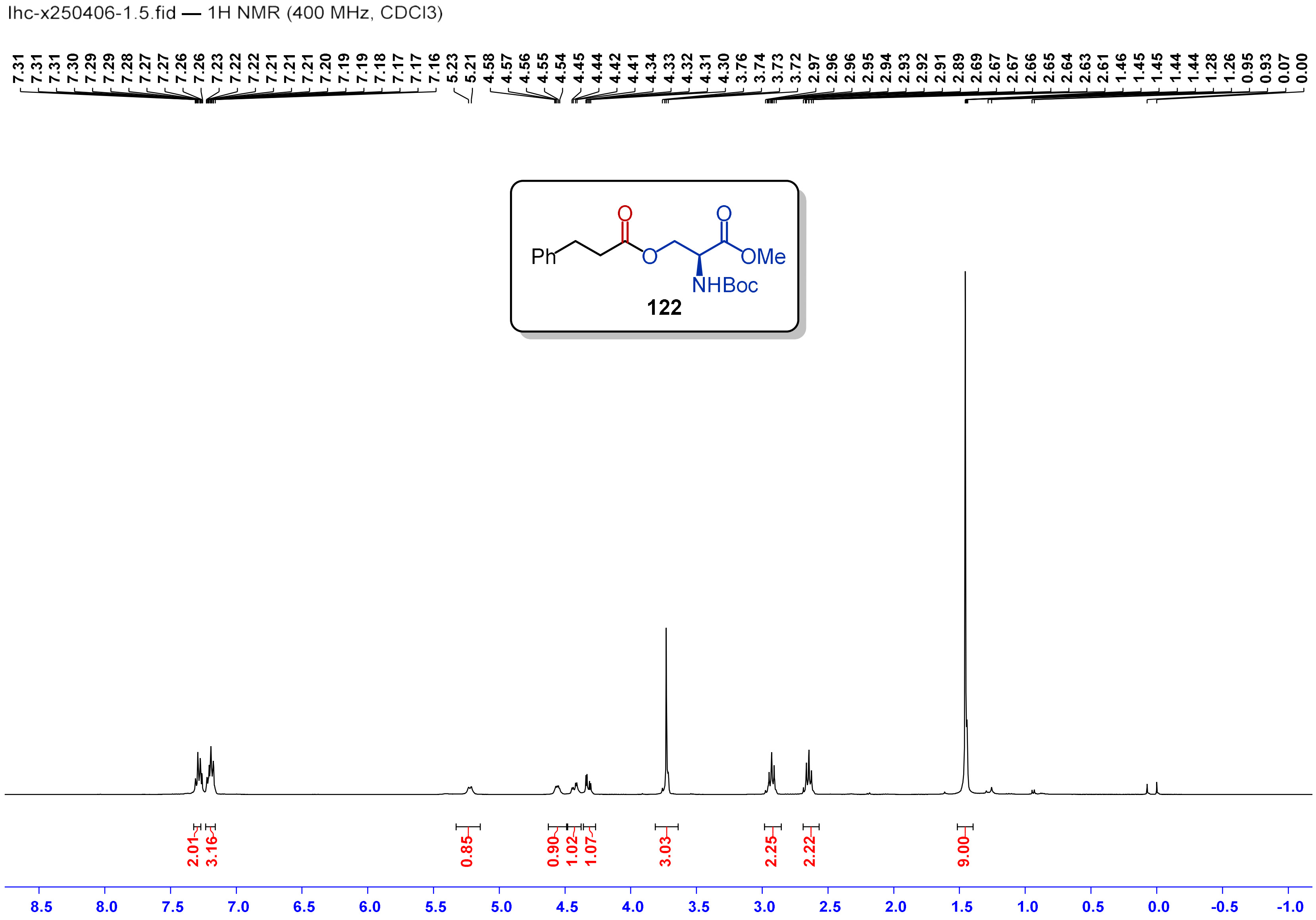

^{13}C NMR spectra for 122

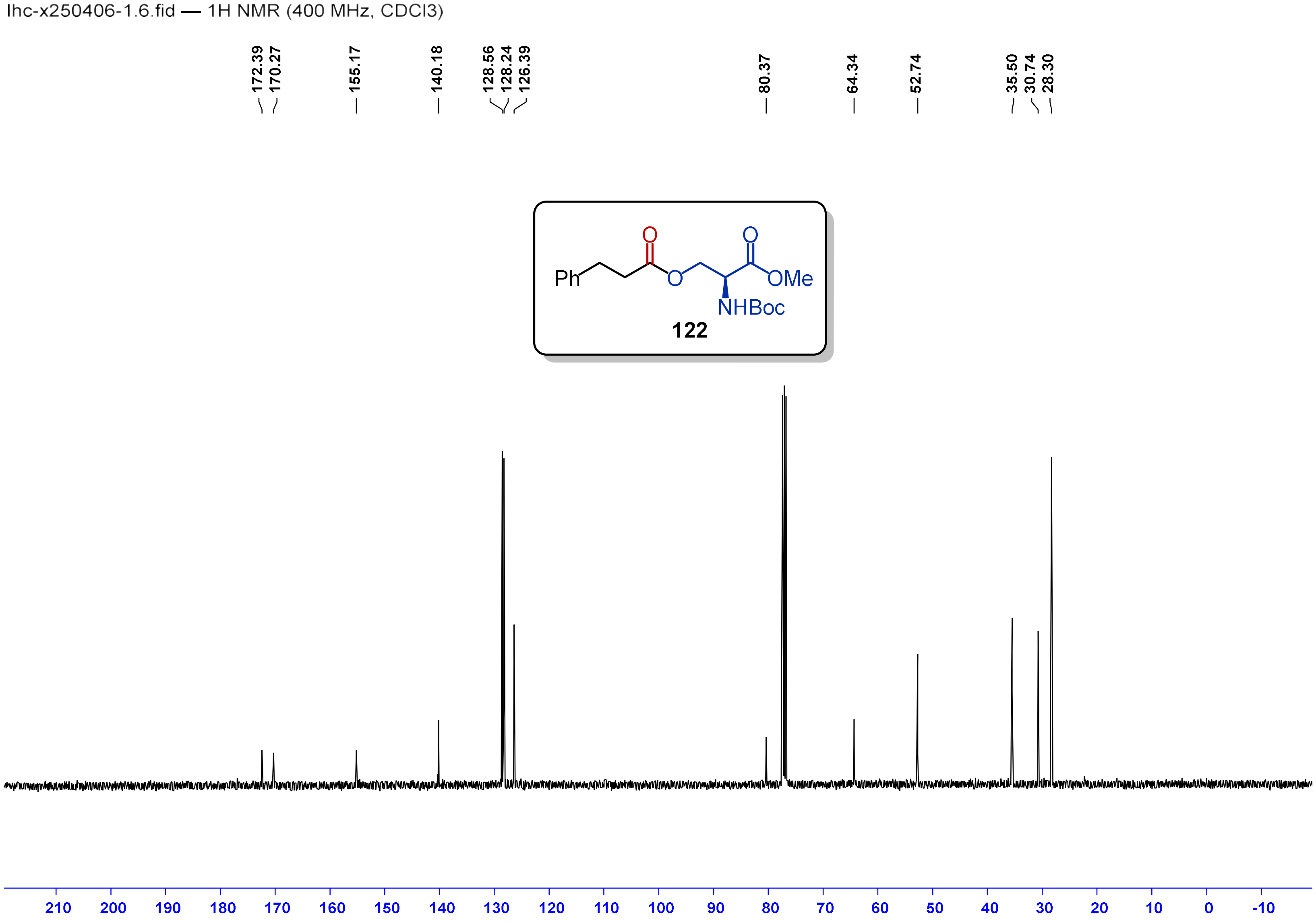

^{1}H NMR spectra for 123

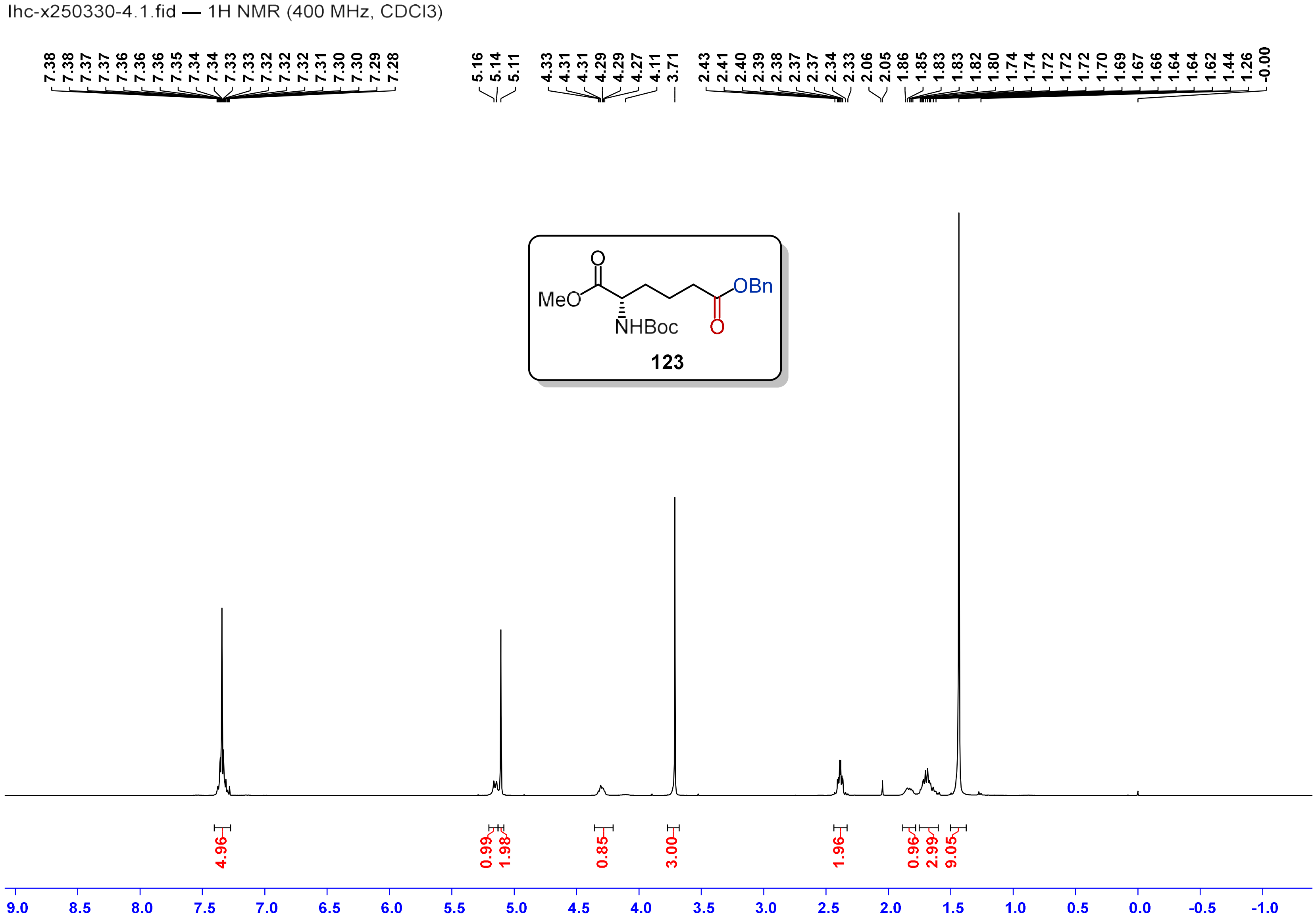

^{13}C NMR spectra for 123

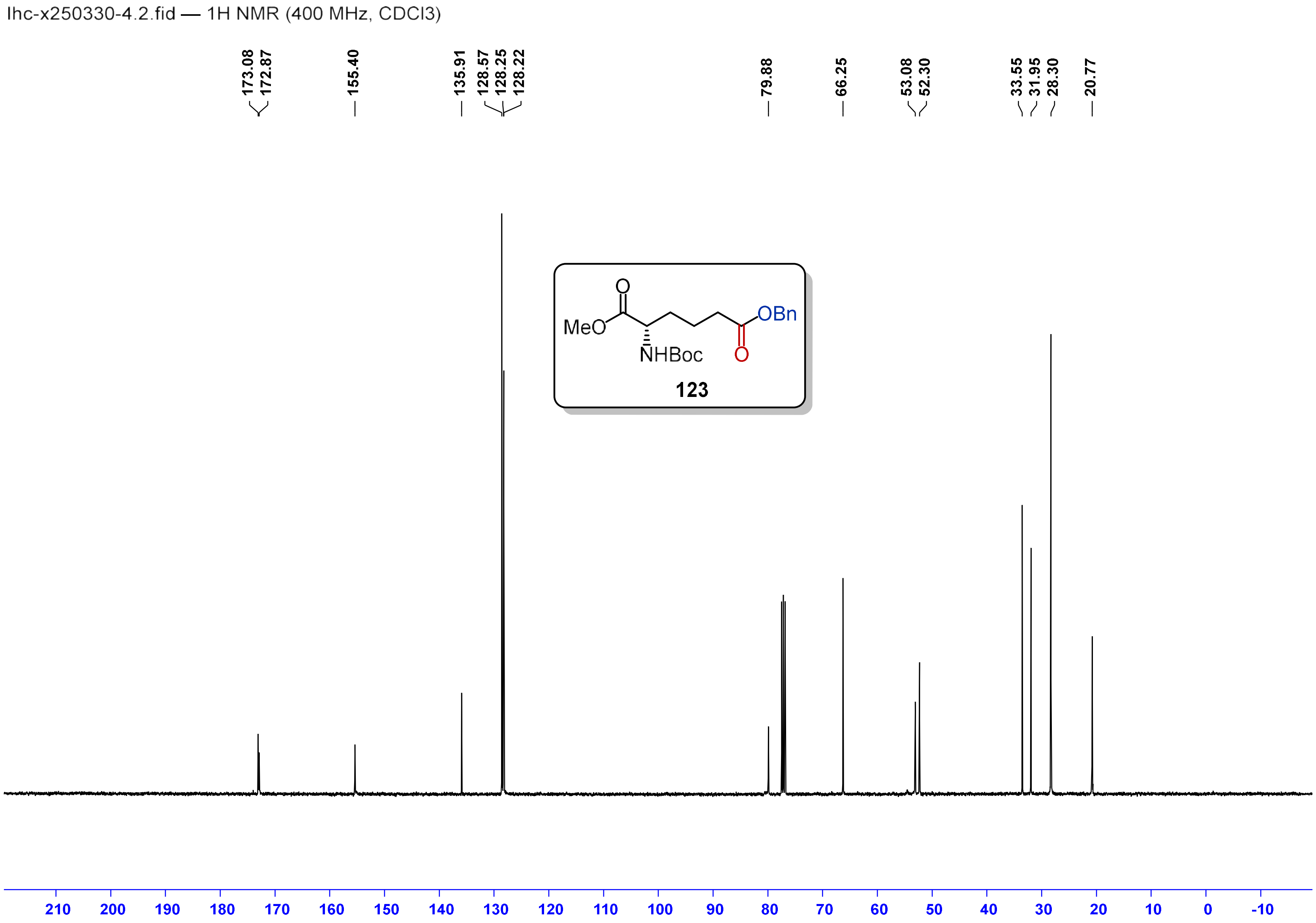

^{1}H NMR spectra for 124

lhc-x250420-1.1.fid — 1H NMR (400 MHz, CDCl3)

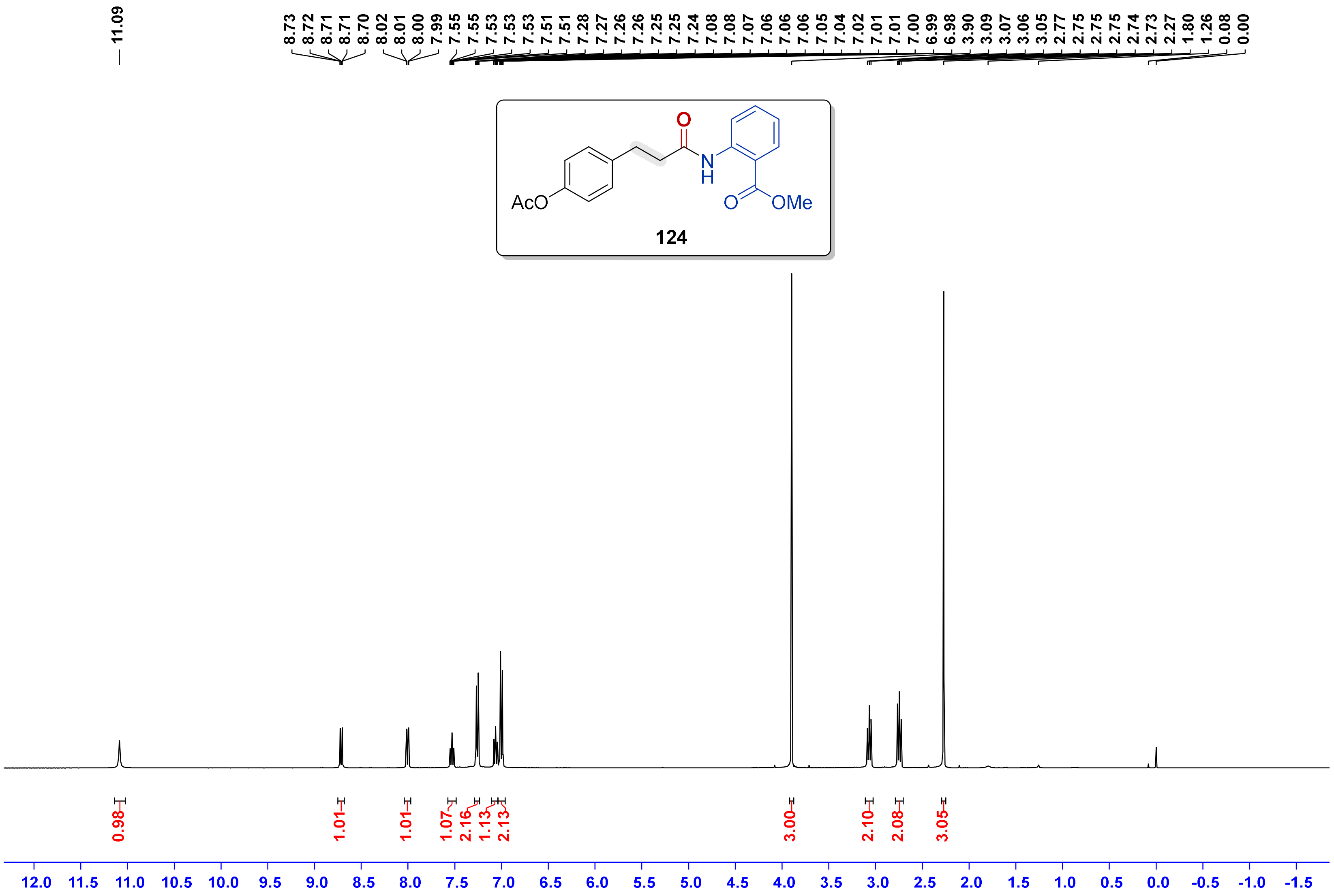

^{13}C NMR spectra for 124

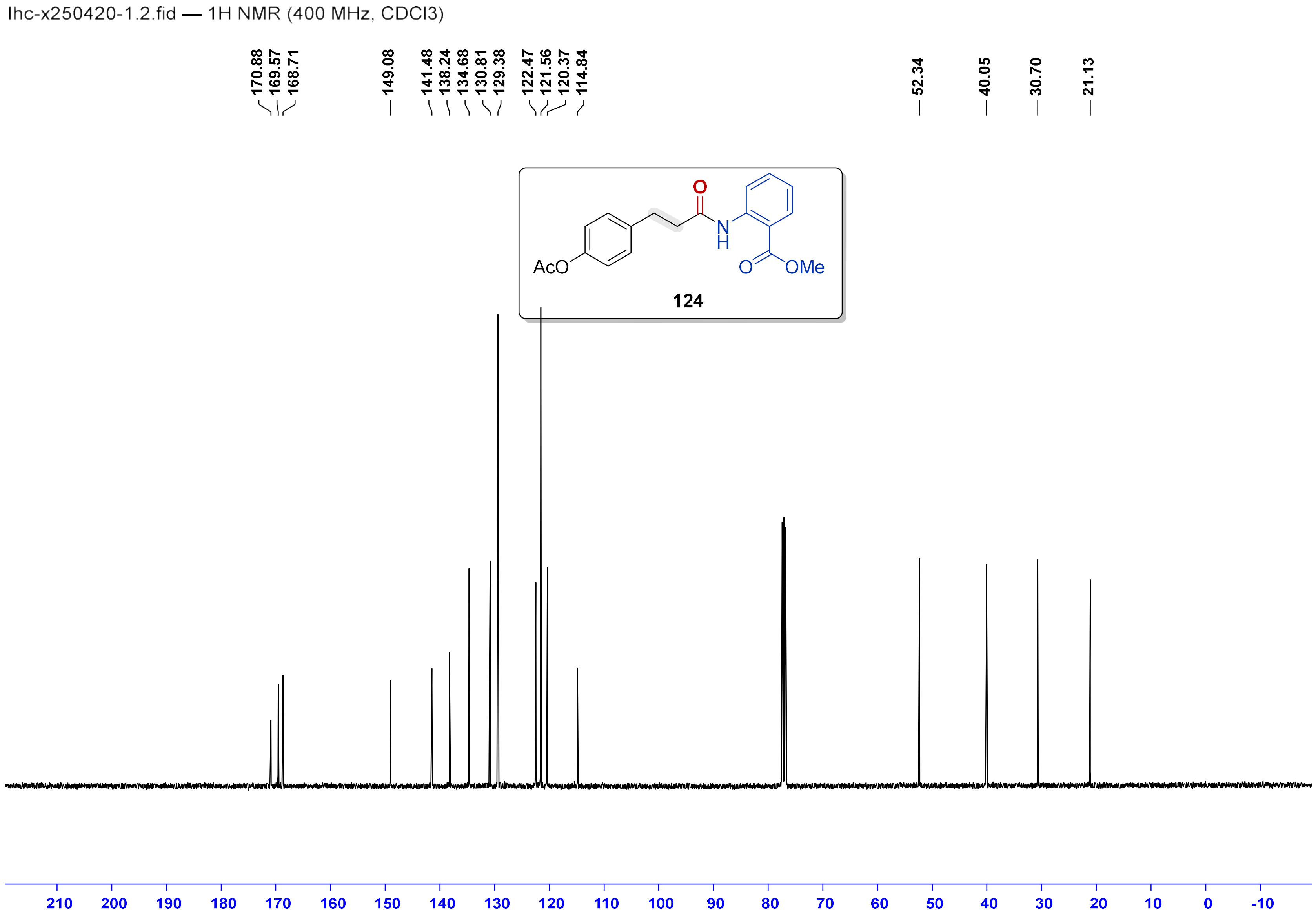

^{1}H NMR spectra for 125

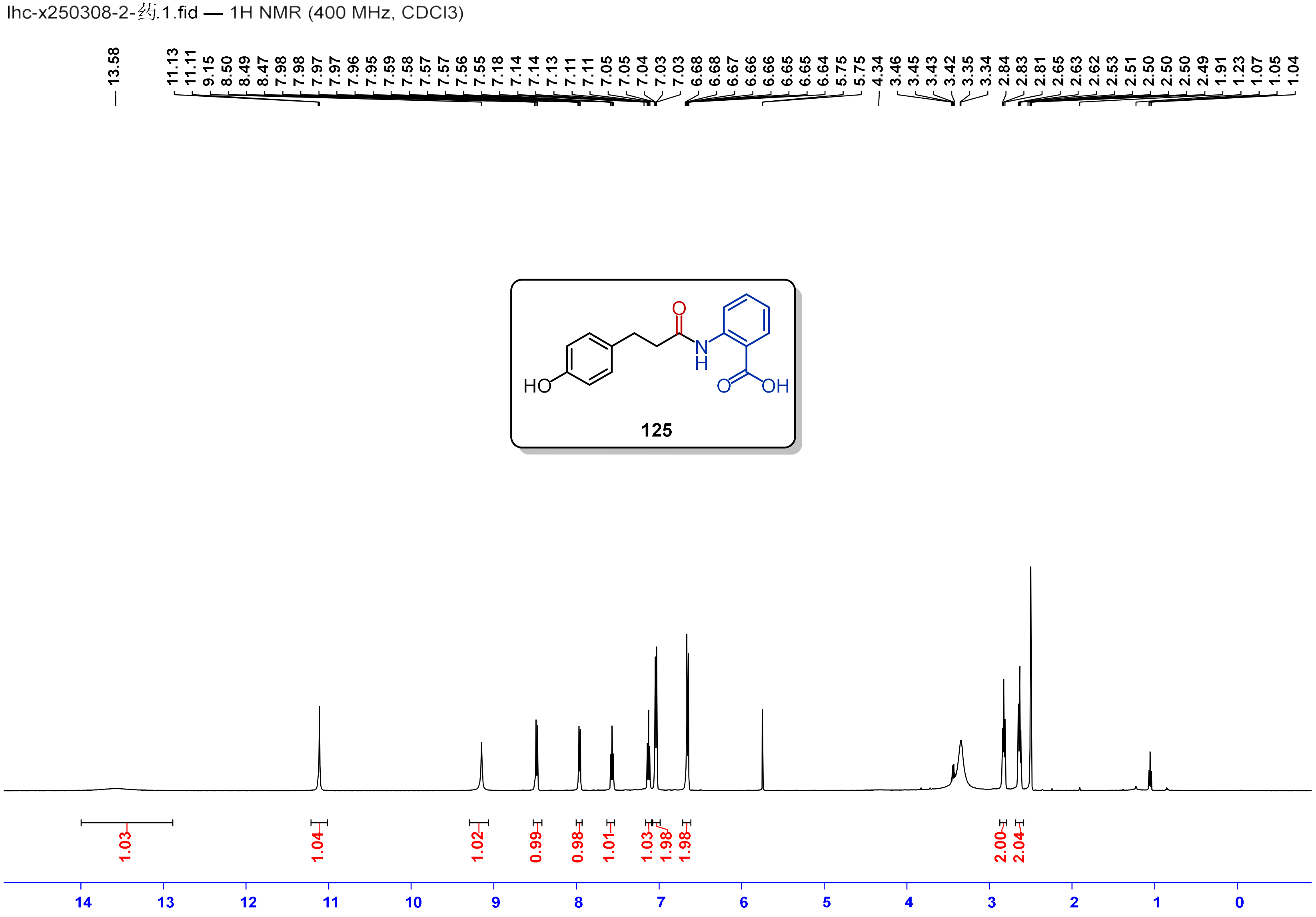

^{13}C NMR spectra for 125

lhc-x250308-2-药.2.fid — 1H NMR (400 MHz, CDCl3)

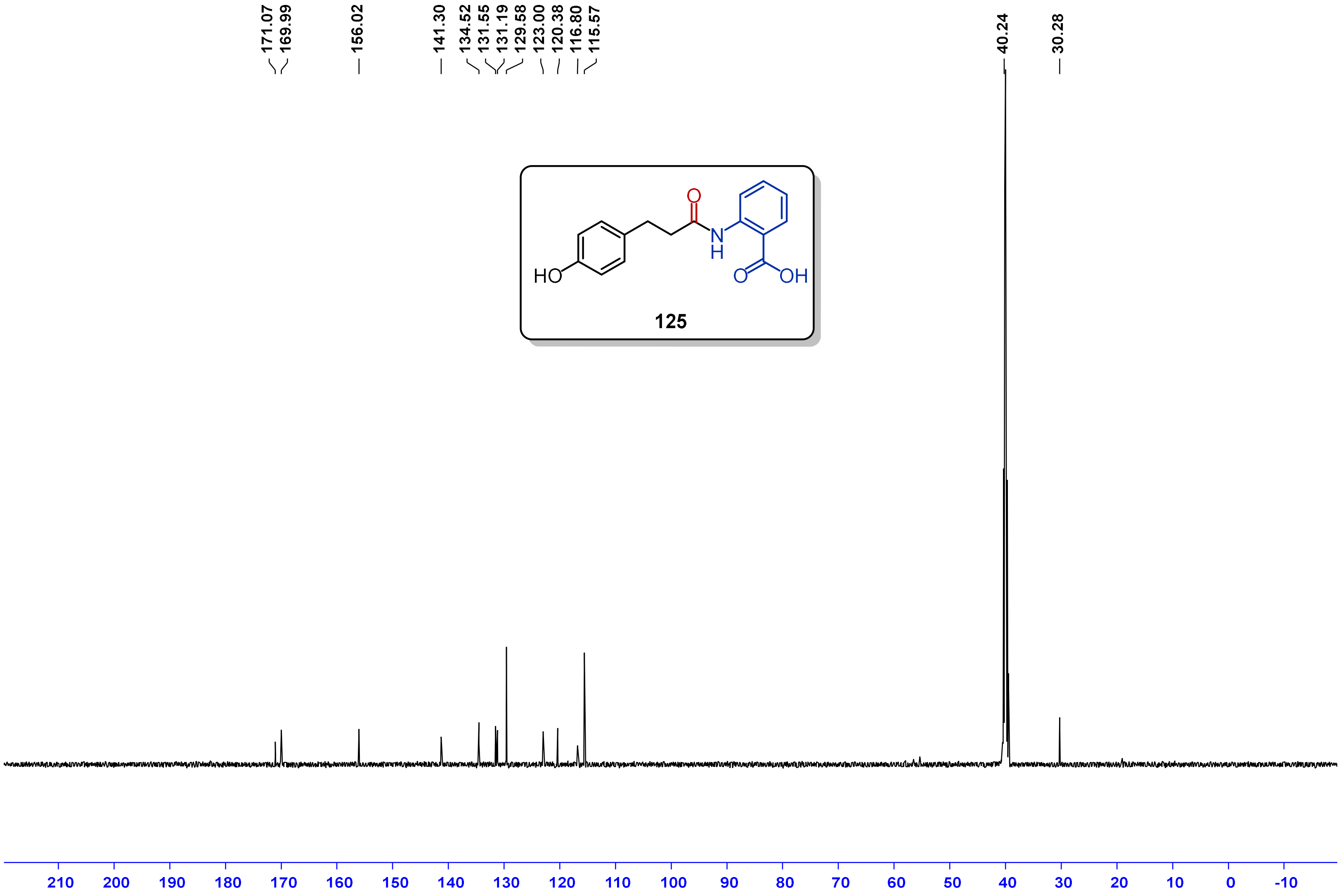

^{1}H NMR spectra for 126

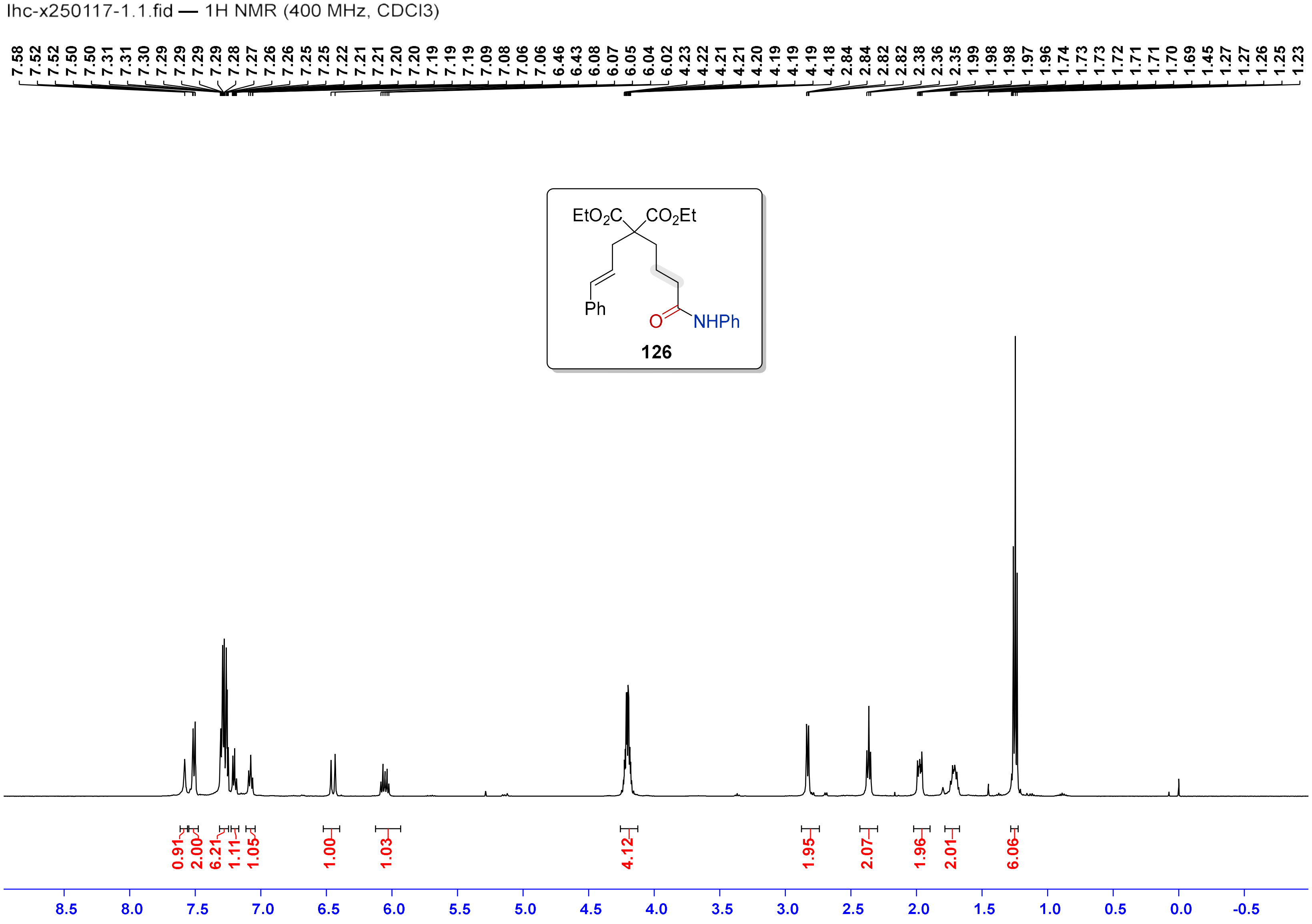

^{13}C NMR spectra for 126

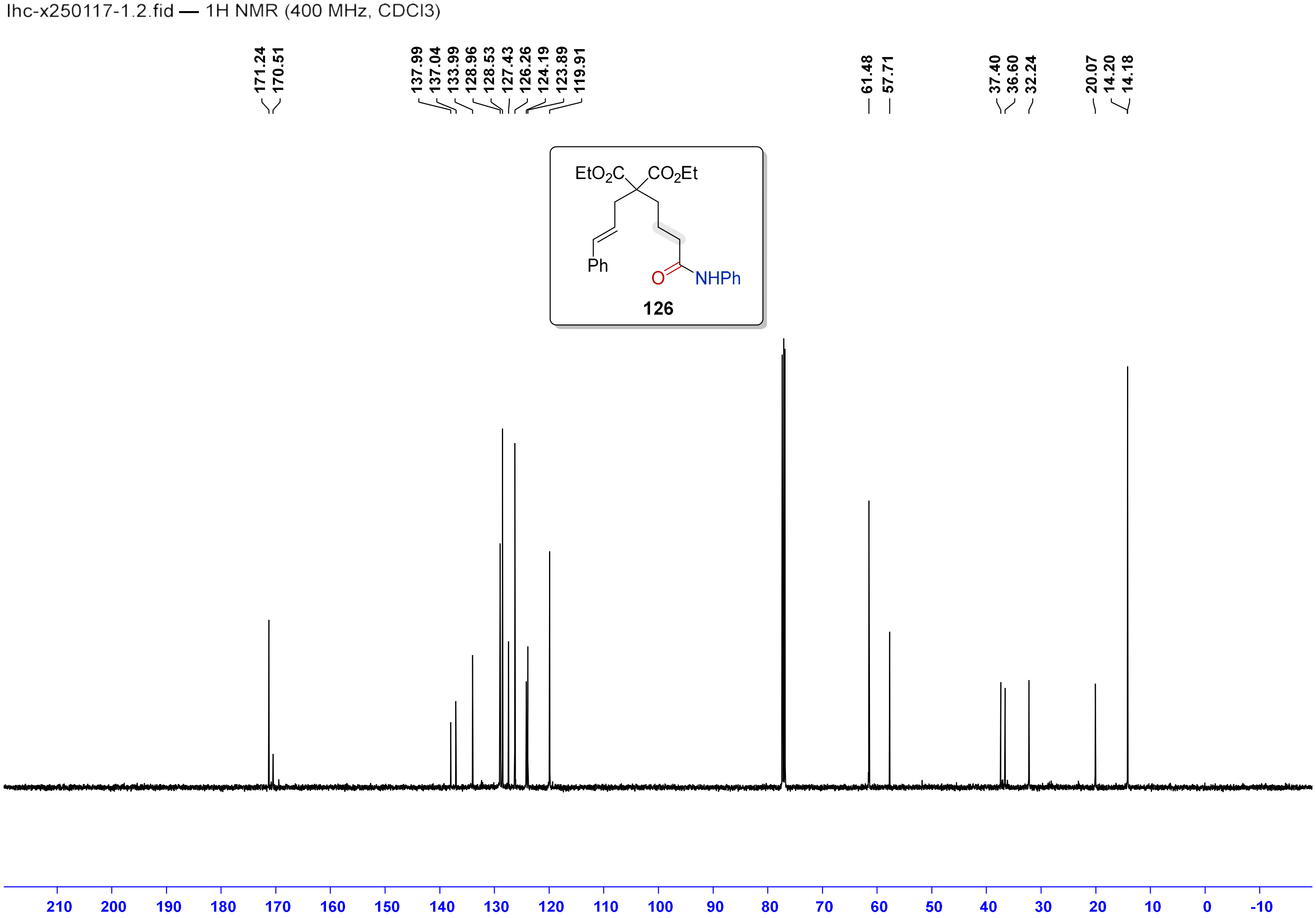

^{1}H NMR spectra for 127

lhc-x250113-3-2.1.fid — 1H NMR (400 MHz, CDCl3)

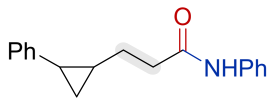

127

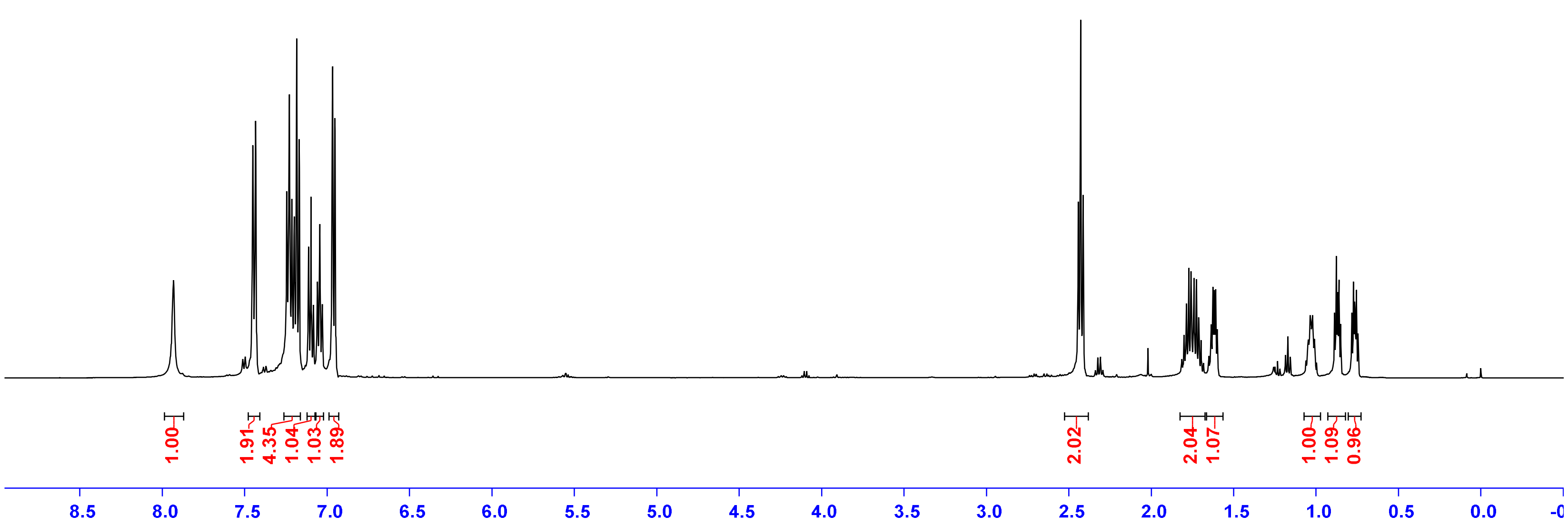

^{13}C NMR spectra for 127

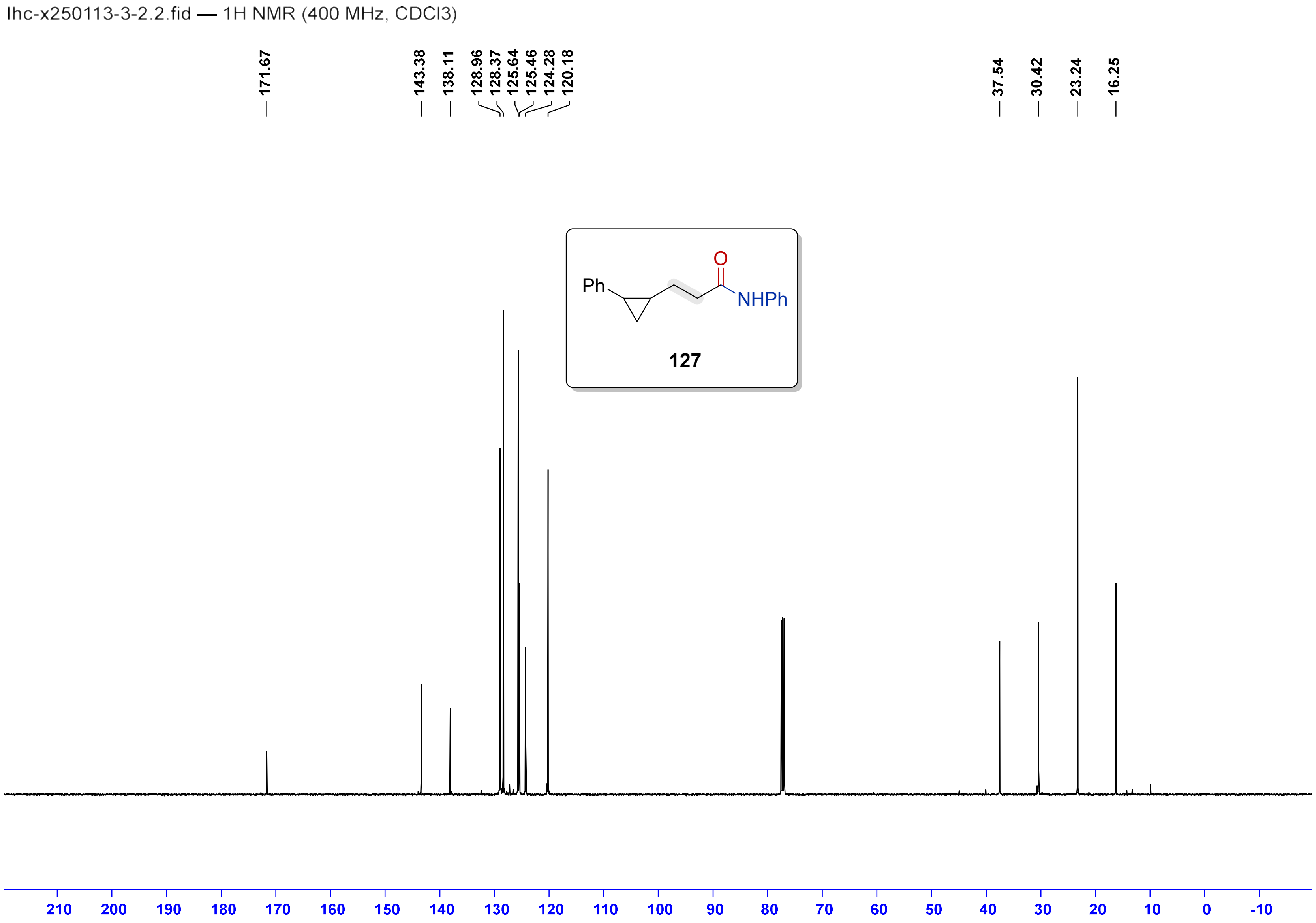

^{1}H NMR spectra for 128

lhc-x250113-3-1.1.fid — 1H NMR (400 MHz, CDCl3)

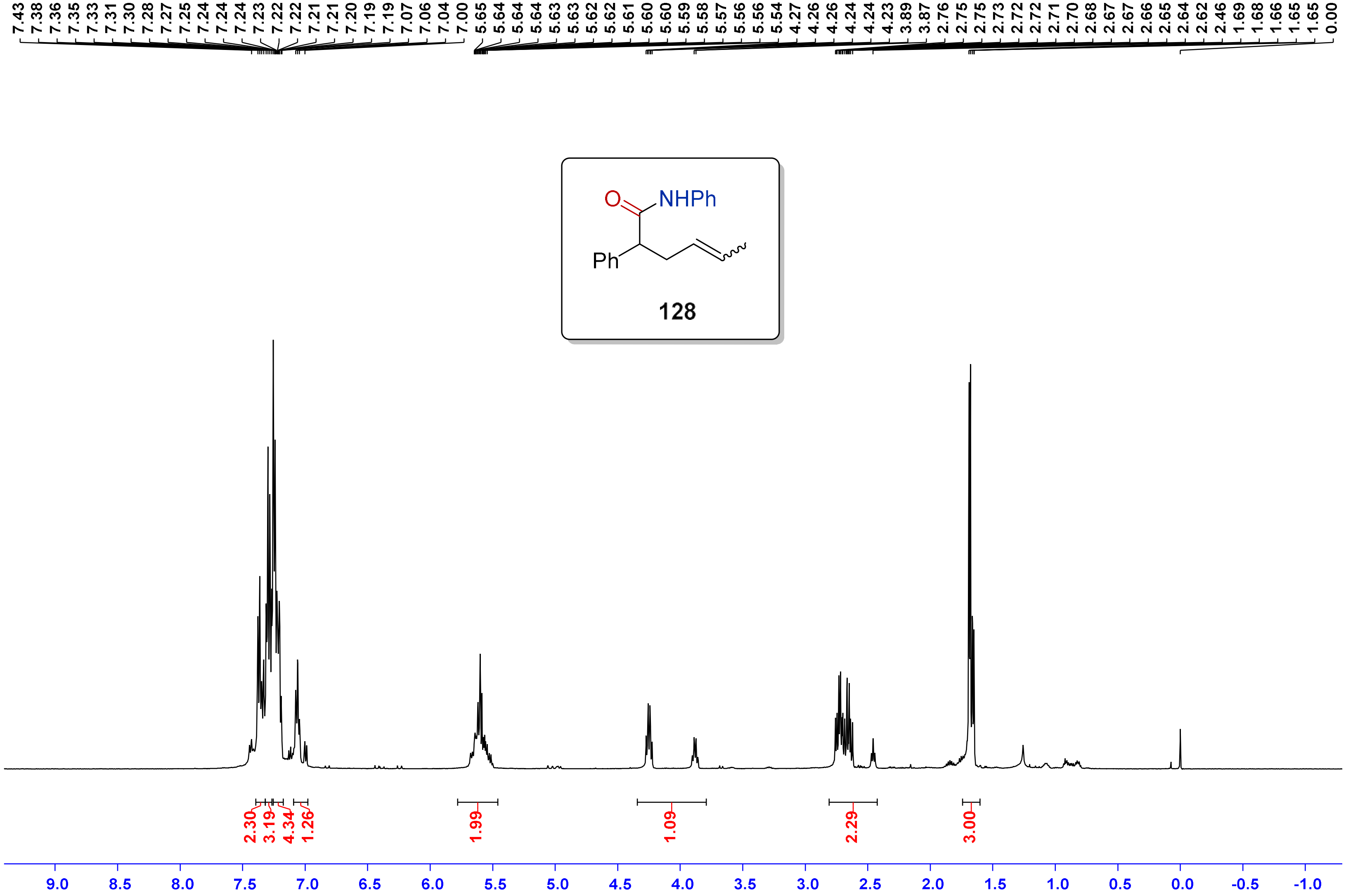

^{13}C NMR spectra for 128

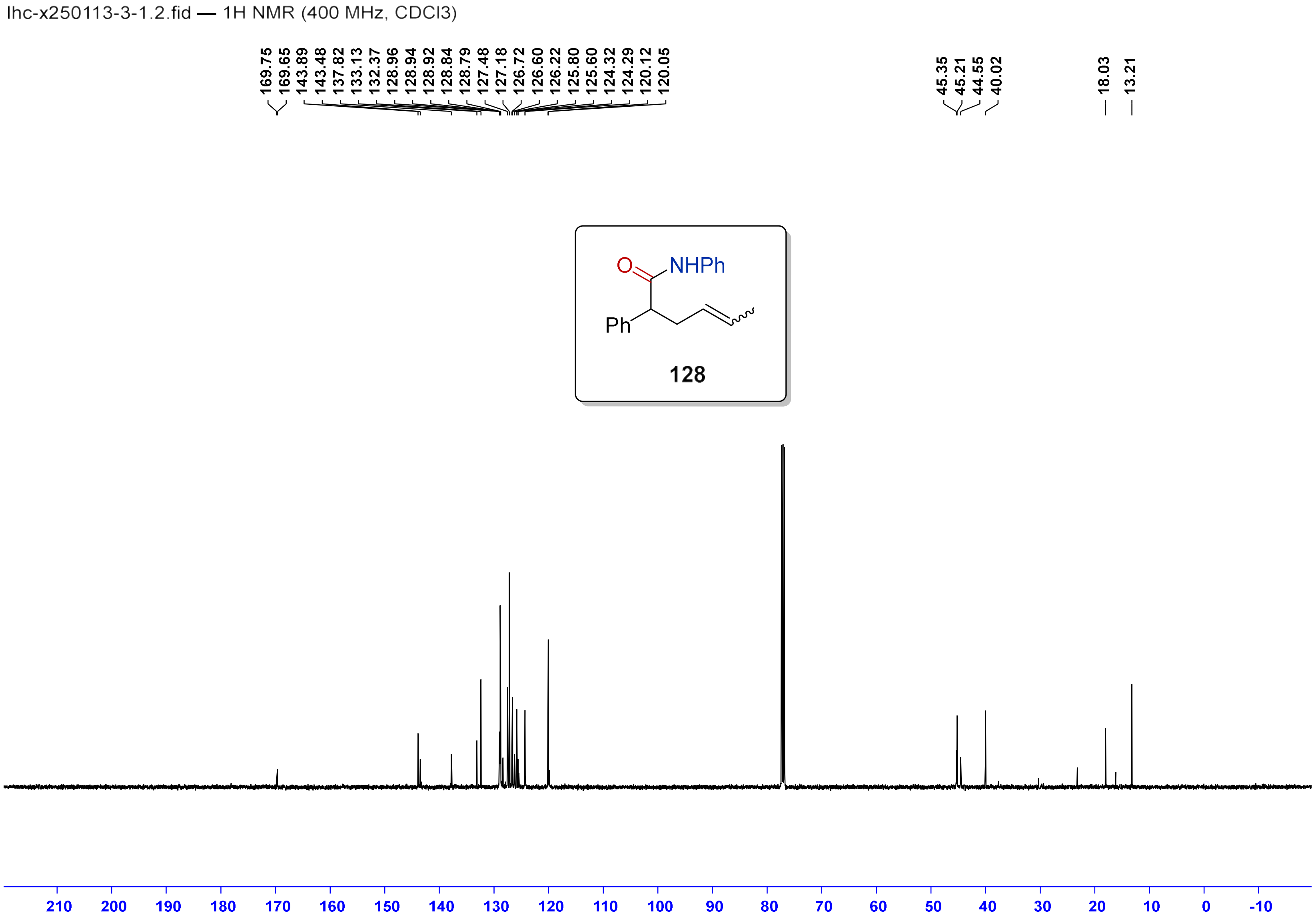

^{1}H NMR spectra for Pd-B

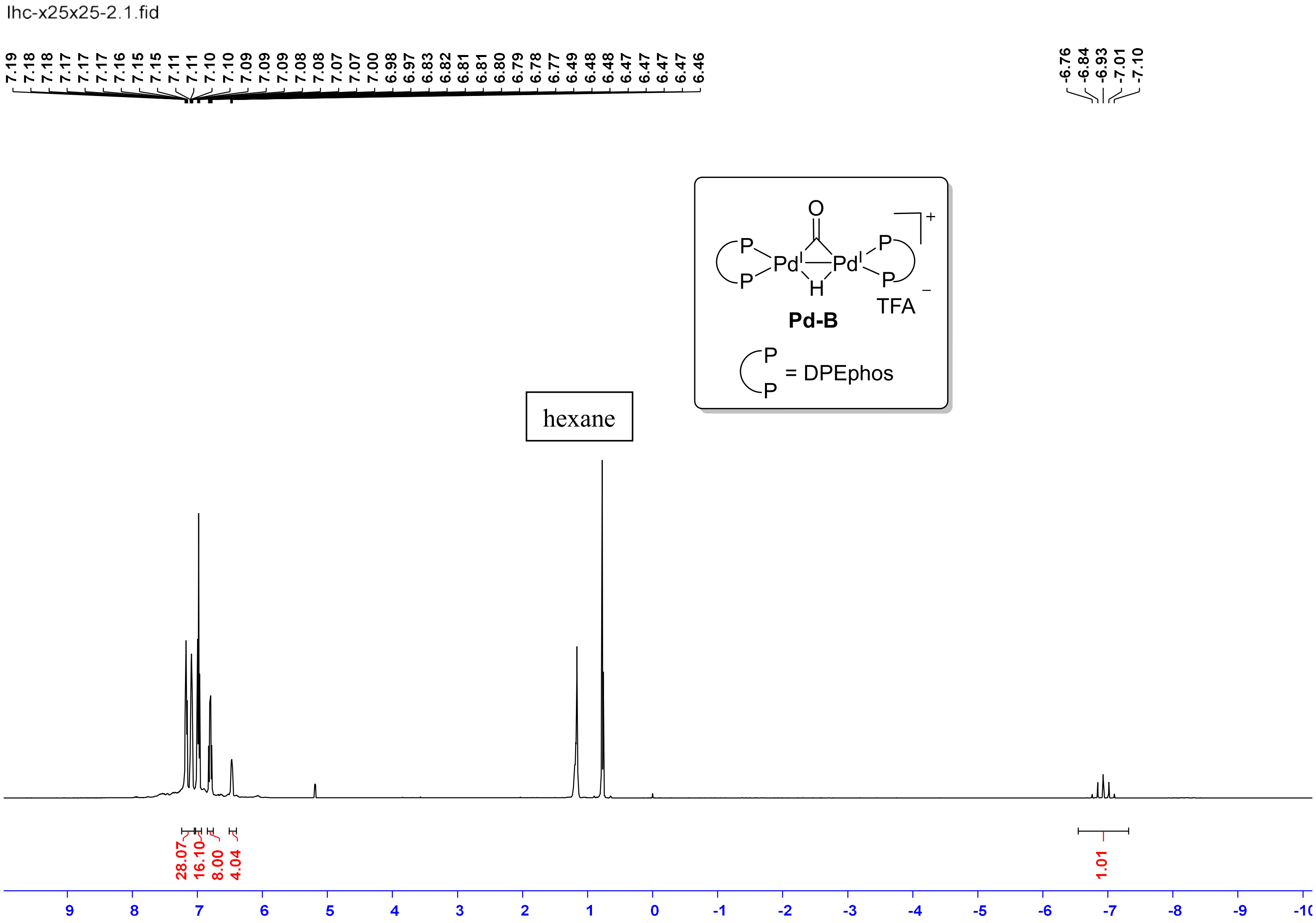

^{13}C NMR spectra for Pd-B

lhc-x25x25-2.2.fid

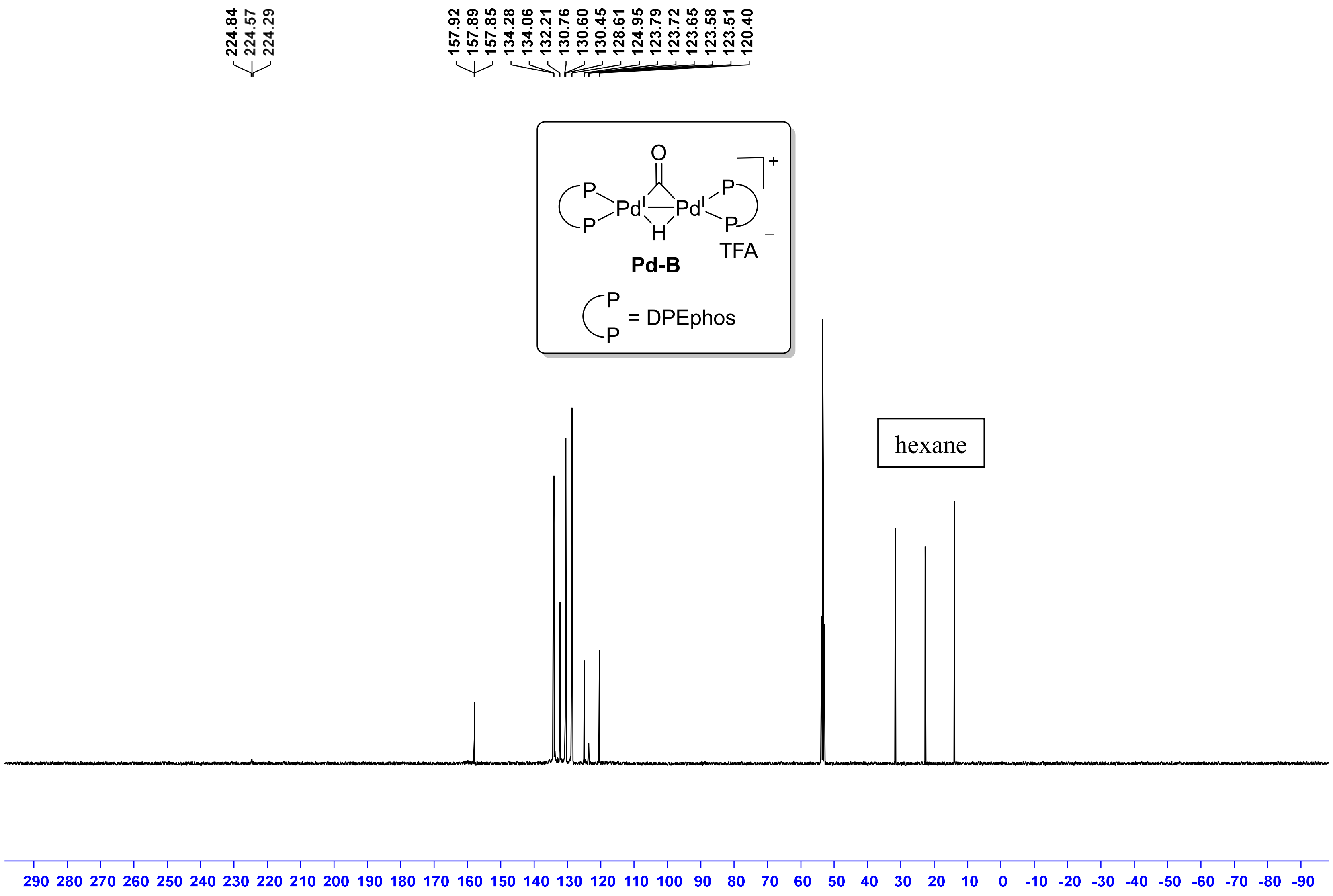

$^{31}P\{^1H\}$ NMR spectra for Pd-B

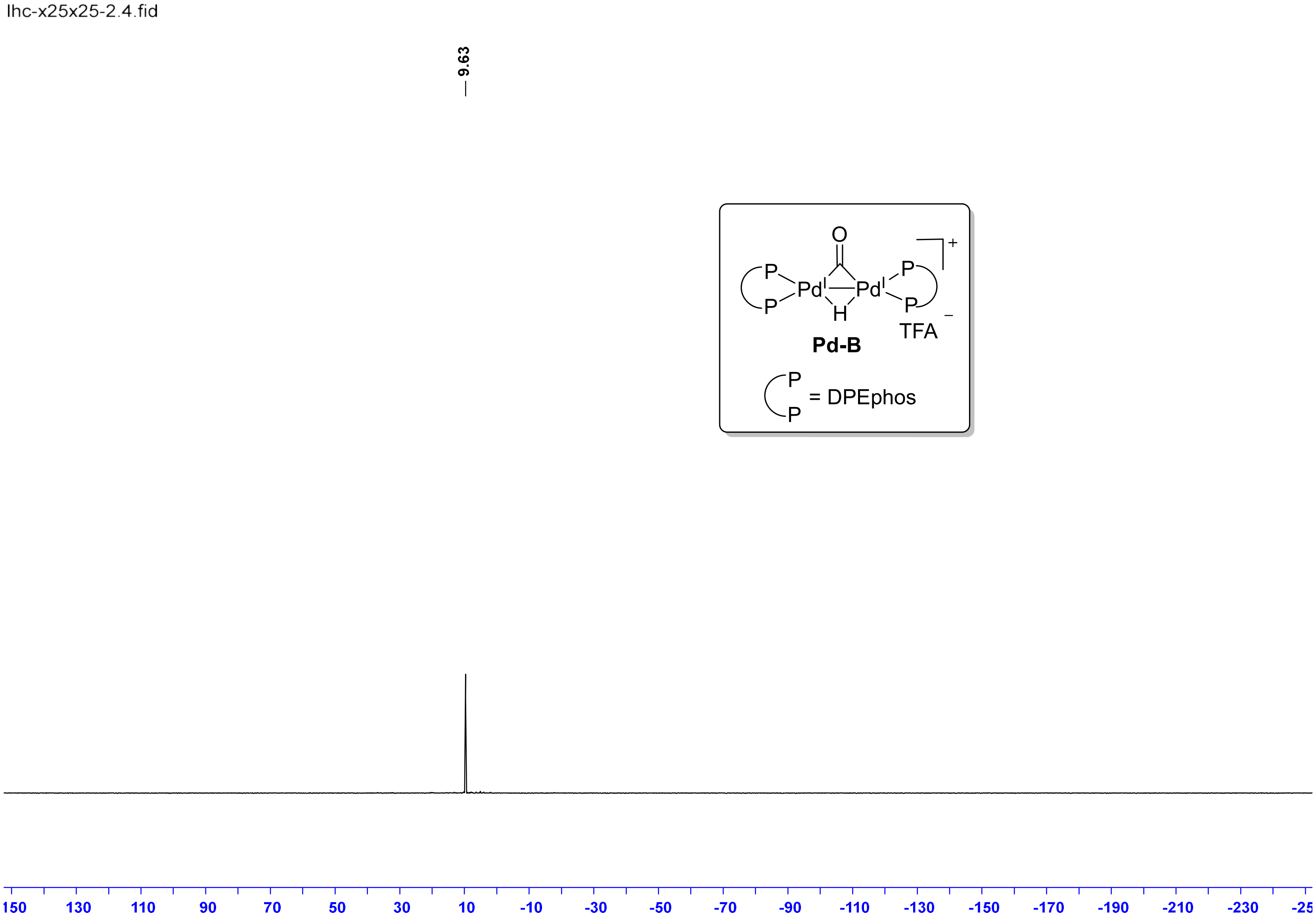

^{31}P NMR spectra for Pd-B

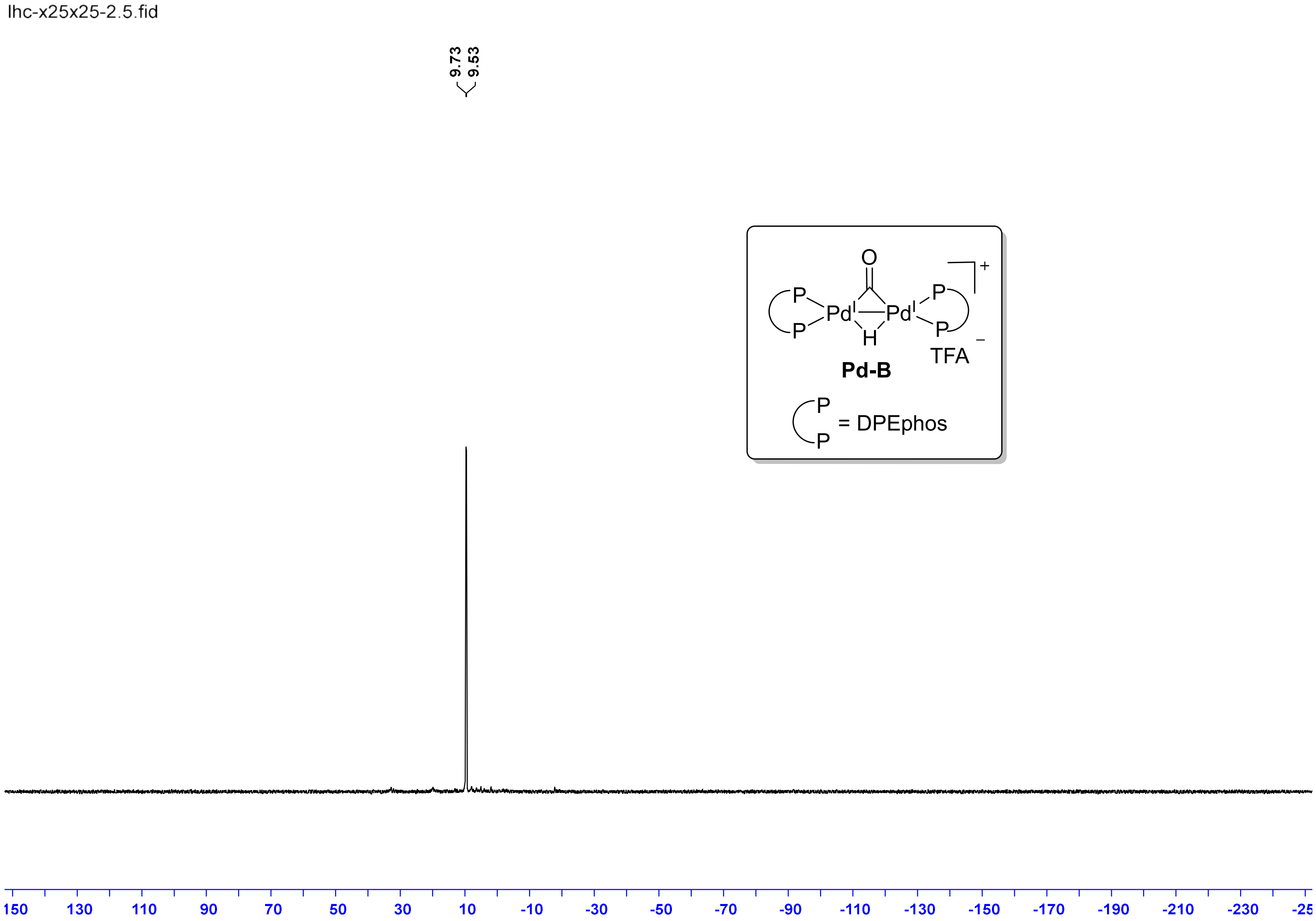

^{19}F NMR spectra for Pd-B

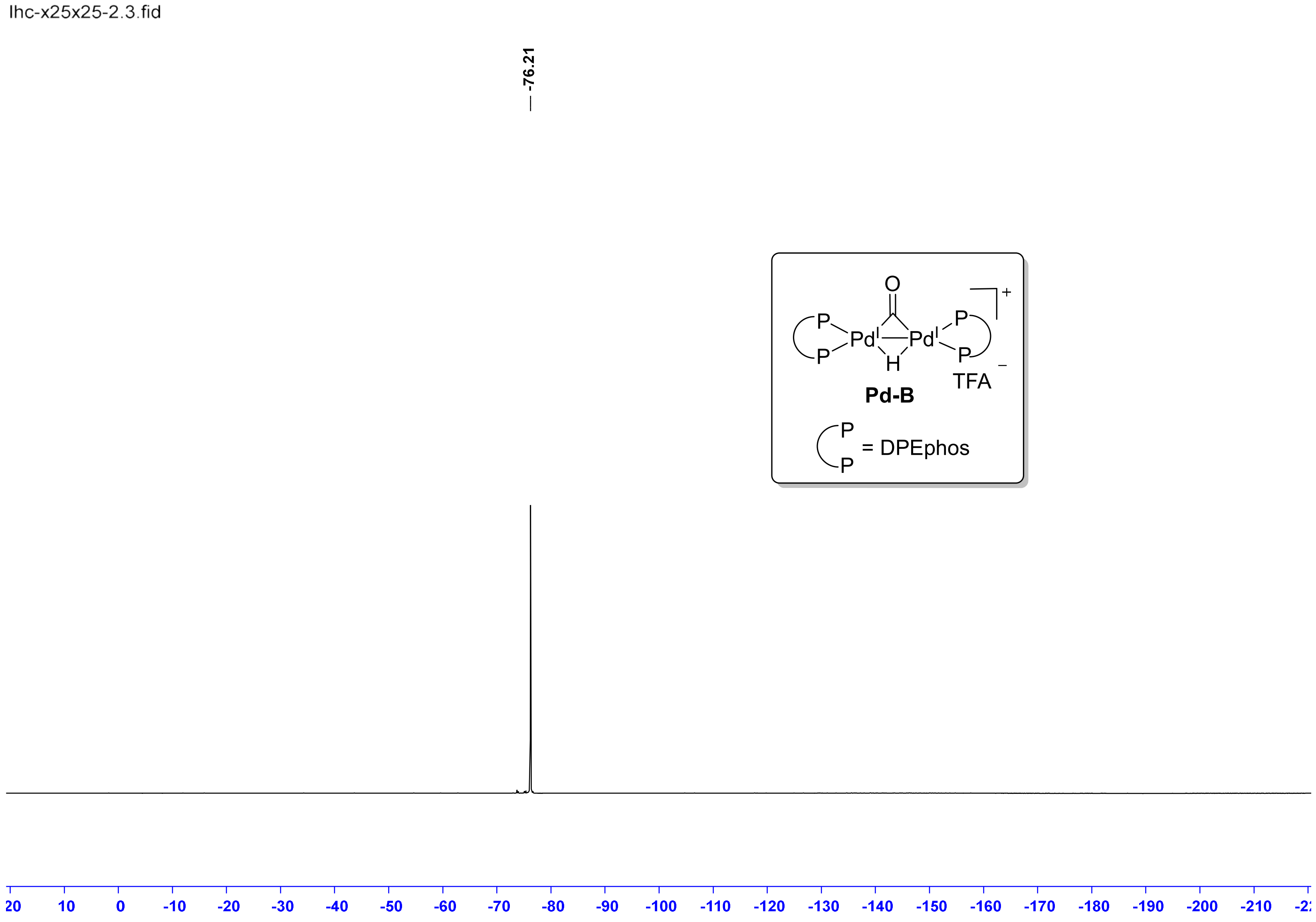

^{1}H-^{13}C HMBC NMR spectra for Pd-B

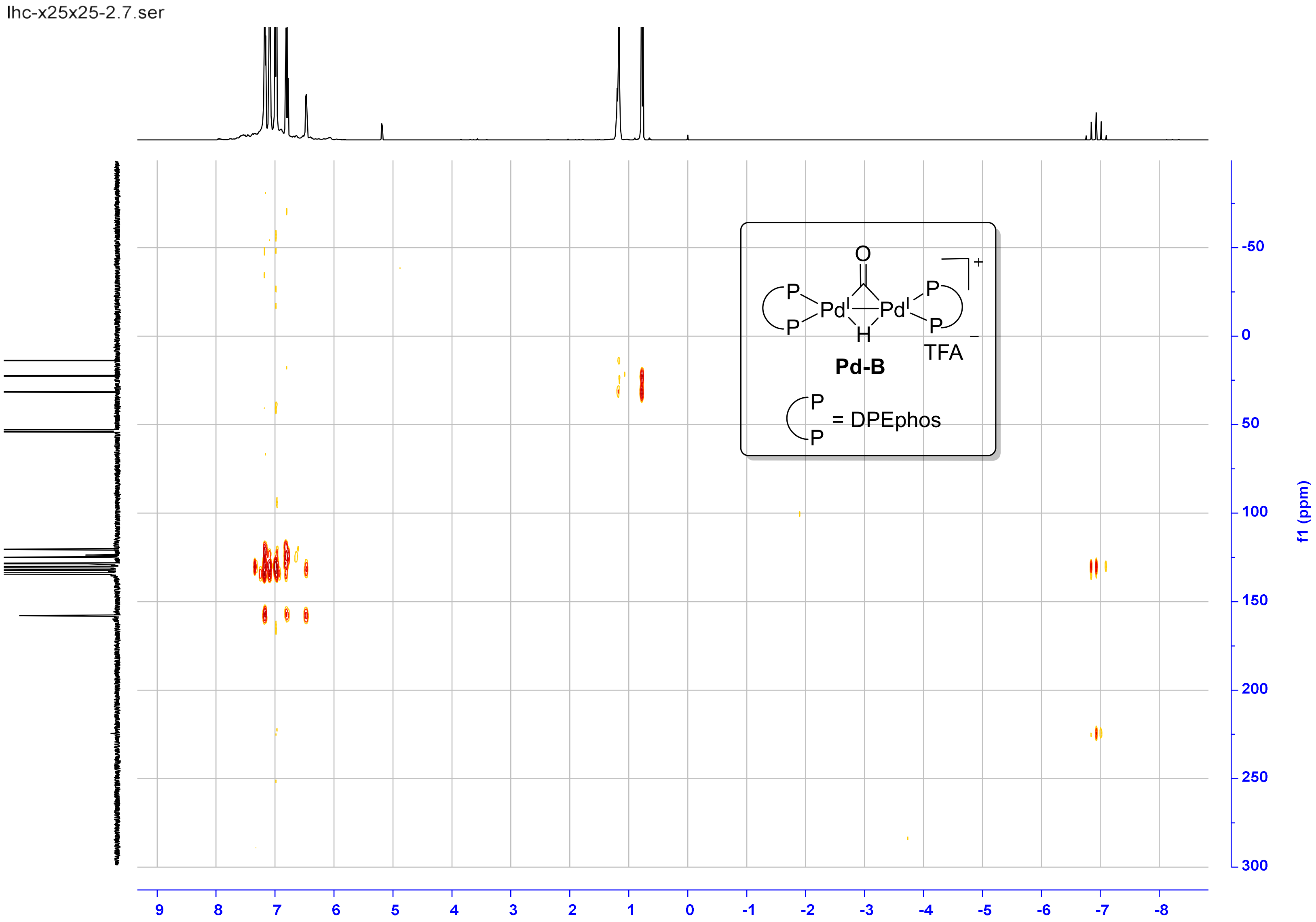

^{1}H NMR spectra for Pd-C

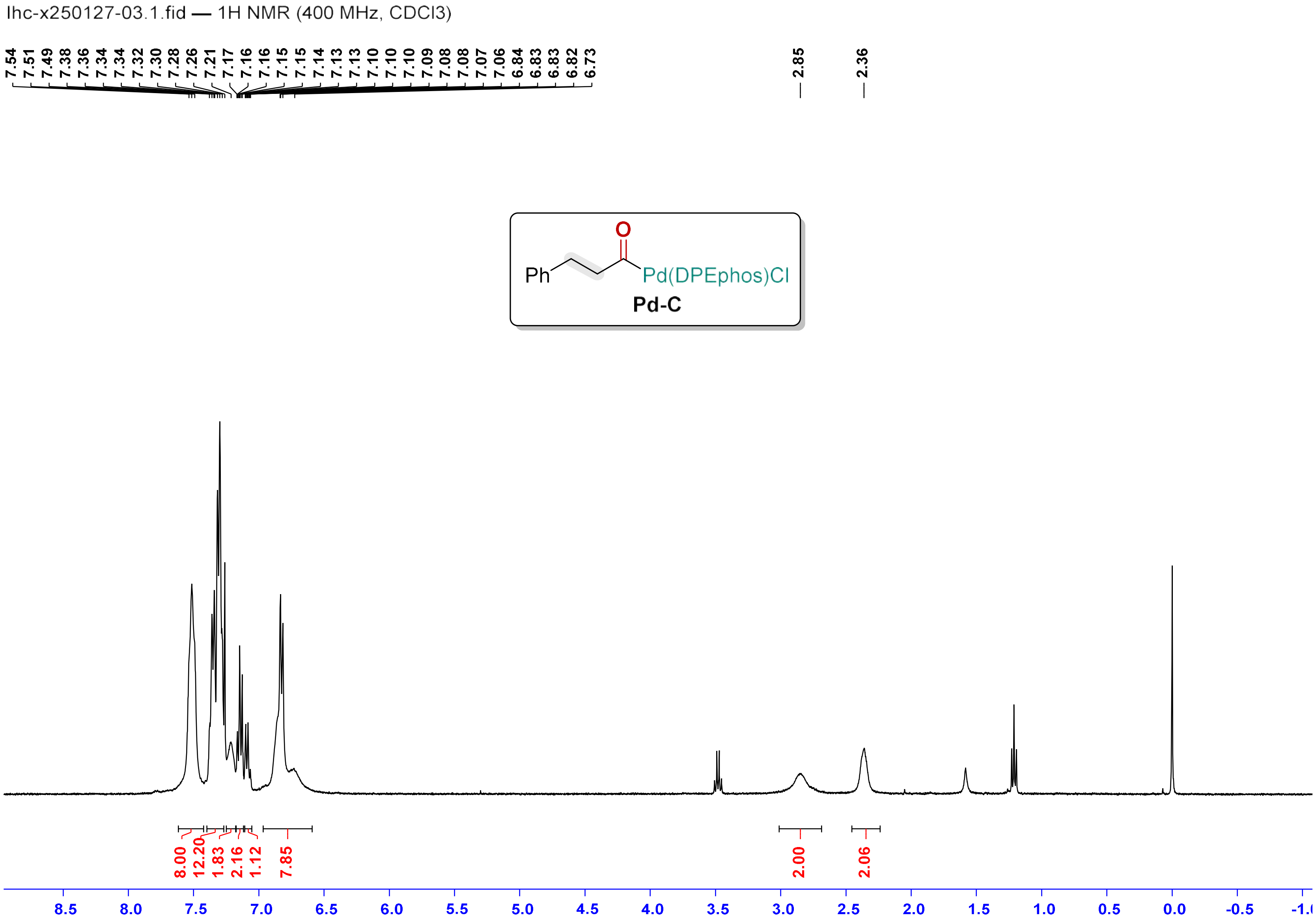

Pd-C

^{13}C NMR spectra for Pd-C

lhc-x250203-01.11.fid — 1H NMR (400 MHz, CDCl3)

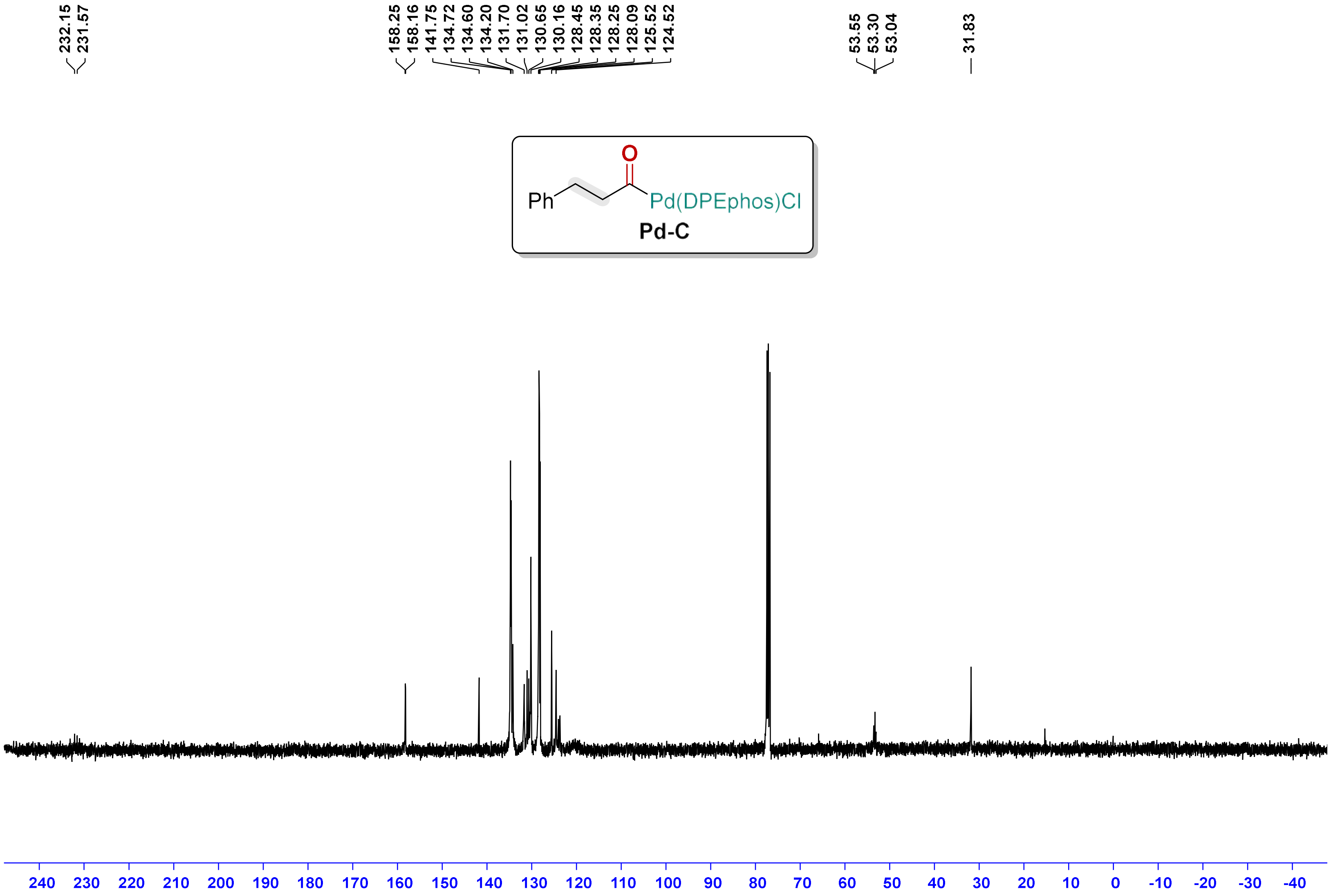

^{31}P NMR spectra for Pd-C

lhc-x250128-04-酰基钯2.fid — 1H NMR (400 MHz, CDCl3)

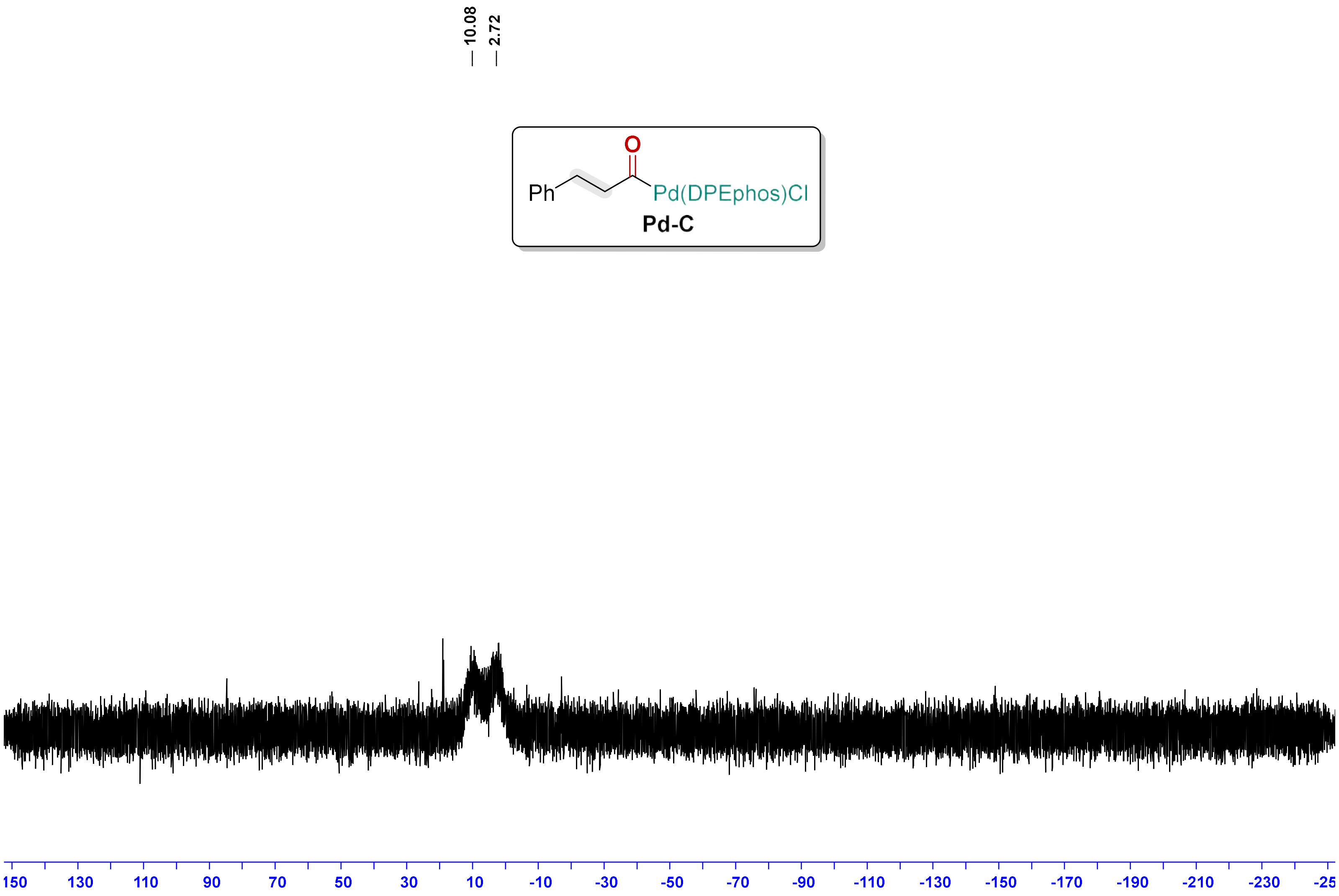

9. Copies of HPLC

HPLC for racemic 98

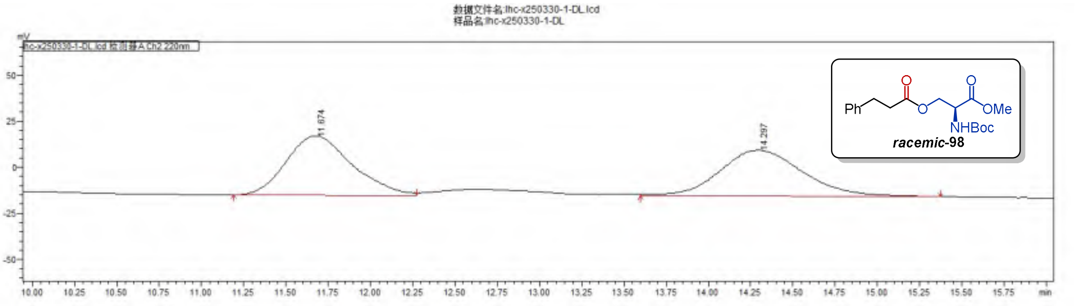

检测器A Ch2 220nm

eakNumbe	RetTime	Area	Height	Tab	Area%
1	11.674	865519	32053	V	50.241
2	14.297	857226	24910		49.759
总计		1722745	56963		100.000

HPLC for 98 produced under blue LED

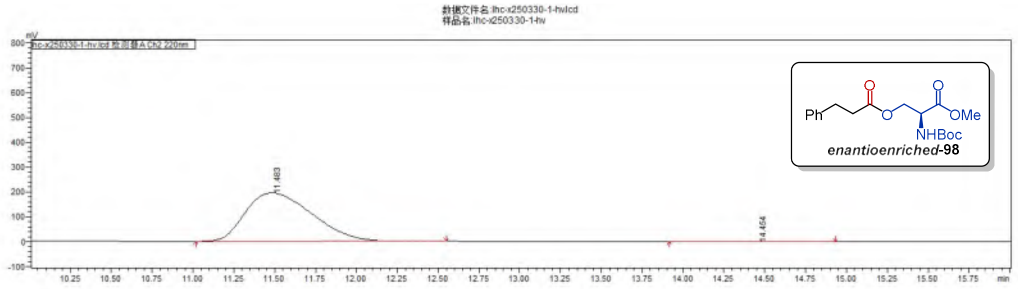

检测器A Ch2 220nm

eakNumbe	RetTime	Area	Height	Tab	Area%
1	11.483	5512152	194396	M	99.817
2	14.454	10127	337	M	0.183
总计		5522279	194733		100.000

HPLC for racemic 99

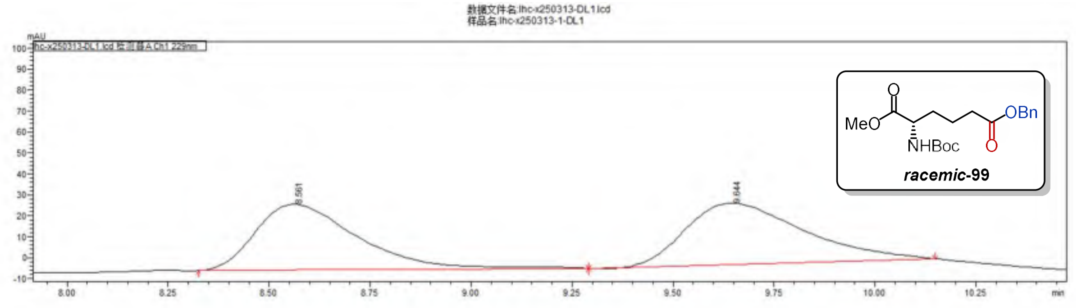

检测器A Ch1 229nm

eakNumbe	RetTime	Area	Height	Tab	Area%
1	8.561	550866	31377	M	49.783
2	9.644	555661	28388	M	50.217
总计		1106526	59765		100.000

HPLC for 99 produced under blue LED

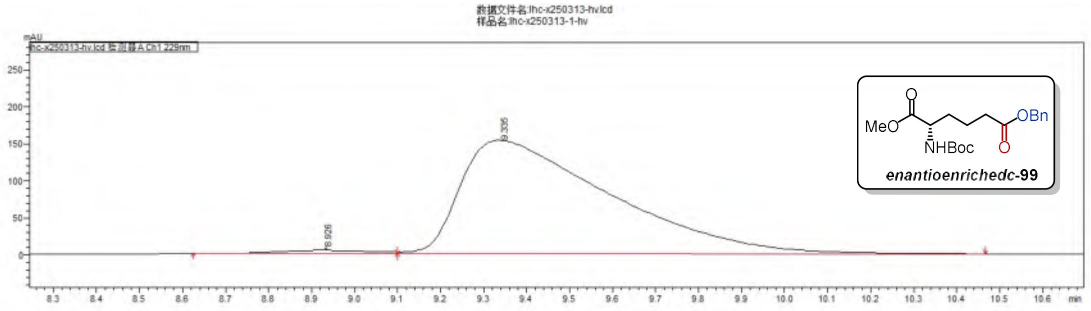

检测器A Ch1 229nm

eakNumbe	RetTime	Area	Height	Tab	Area%
1	8.926	62568	4165	M	1.665
2	9.335	3694560	153010	V M	98.335
总计		3757128	157175		100.000

HPLC for 99 produced under 120°C and 30 atm CO

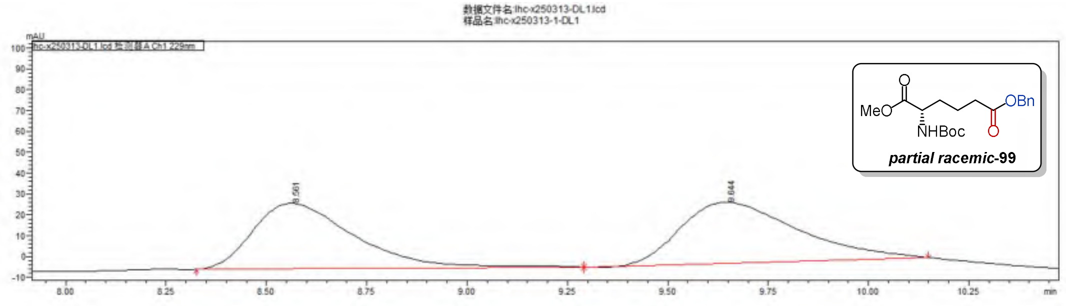

检测器A Ch1 229nm

eakNumbe	RetTime	Area	Height	Tab	Area%
1	8.795	384345	20997	M	43.298
2	9.662	503338	26881	V M	56.702
总计		887684	47878		100.000

10. References

[1] Y. Zhu, V. H. Rawal, *J. Am. Chem. Soc.* **2012**, *134*, 111-114.

[2] T. M. Konrad, J. A. Fuentes, A. M. Z. Slawin, M. L. Clarke, *Angew. Chem. Int. Ed.* **2010**, *49*, 9197-9200.

[3] S. J. Leiris, O. M. Khdour, Z. J. Segerman, K. S. Tsosie, J. Chapuis, S. M. Hecht, *Bioorg. Med. Chem.* **2010**, *18*, 3481-3493.

[4] S. Kobayashi, T. Kinoshita, T. Kawamoto, M. Wada, H. Kuroda, A. Masuyama, I. Ryu, *J. Org. Chem.* **2011**, *76*, 7096-7103.

[5] Z. K. Wickens, B. Morandi, R. H. Grubbs, *Angew. Chem. Int. Ed.* **2013**, *52*, 11257-11260.

[6] I. Fleischer, K. M. Dyballa, R. Jennerjahn, R. Jackstell, R. Franke, A. Spannenberg, M. Beller, *Angew. Chem. Int. Ed.* **2013**, *52*, 2949-2953.

[7] M. Vellakkaran, M. M. S. Andappan, N. Kommu, *Green Chem.* **2014**, *16*, 2788-2797.

[8] F. Buckingham, A. K. Kirjavainen, S. Forsback, A. Krzyczmonik, T. Keller, I. M. Newington, M. Glaser, S. K. Luthra, O. Solin, V. Gouverneur, *Angew. Chem. Int. Ed.* **2015**, *54*, 13366-13369.

[9] W. Ren, W. Chang, J. Dai, Y. Shi, J. Li, Y. Shi, *J. Am. Chem. Soc.* **2016**, *138*, 14864-14867.

[10] J. Lee, S. C. Shin, S. H. Seo, Y. H. Seo, N. Jeong, C. Kim, E. E. Kim, G. Keum, *Bioorg. Med. Chem. Lett.* **2017**, *27*, 237-241.

[11] Q. Meng, S. Wang, G. S. Huff, B. König, *J. Am. Chem. Soc.* **2018**, *140*, 3198-3201.

[12] P. Zhang, H. Shen, L. Zhu, W. Cao, C. Li, *Org. Lett.* **2018**, *20*, 7062-7065.

[13] C. Verrier, N. Alandini, C. Pezzetta, M. Moliterno, L. Buzzetti, H. B. Hepburn, A. Vega-Peñaloza, M. Silvi, P. Melchiorre, *ACS Catal.* **2018**, *8*, 1062-1066.

[14] W. Ren, J. Chu, F. Sun, Y. Shi, *Org. Lett.* **2019**, *21*, 5967-5970.

[15] R. Sang, P. Kucmierczyk, R. Dühren, R. Razzaq, K. Dong, J. Liu, R. Franke, R. Jackstell, M. Beller, *Angew. Chem. Int. Ed.* **2019**, *58*, 14365-14373.

[16] N. Ashush, R. Fallek, A. Fallek, R. Dobrovetsky, M. Portnoy, *Org. Lett.* **2020**, *22*, 3749-3754.

[17] G. M. Torres, Y. Liu, B. A. Arndtsen, *Science* **2020**, *368*, 318-323.

[18] X. Li, X. Yang, P. Chen, G. Liu, *J. Am. Chem. Soc.* **2022**, *144*, 22877-22883.

[19] J. Tu, H. Gao, M. Luo, L. Zhao, C. Yang, L. Guo, W. Xia, *Green Chem.* **2022**, *24*, 5553-5558.

[20] T. Yuan, Z. Wu, S. Zhai, R. Wang, S. Wu, J. Cheng, M. Zheng, X. Wang, *Angew. Chem. Int. Ed.* **2023**, *62*, e202304861.

[21] H. Joshi, D. Paul, S. Sathyamoorthi, *J. Org. Chem.* **2023**, *88*, 11240-11252.

[22] A. Kumar, R. Gupta, G. Mani, *Organometallics* **2023**, *42*, 732-744.

[23] F. Khamespanah, J. B. Gerken, D. S. Mannel, S. Nagy, B. Kimmich, S. S. Stahl, *Organometallics* **2024**, *43*, 1502-1510.

[24] P. Ghosh, S. Maiti, A. Malandain, D. Raja, O. Loreau, B. Maity, T. K. Roy, D. Audisio, D. Maiti, *J. Am. Chem. Soc.* **2024**, *146*, 30615-30625.

[25] E. Wheatley, H. Melnychenko, M. Silvi, *J. Am. Chem. Soc.* **2024**, *146*, 34285-

34291.
[26] Q. Zhang, H. Qian, S. Ma, *Chem.-Eur. J.* **2025**, *31*, e202404754.
[27] K. Zhang, J. Wang, W. Chen, M. Li, S. Jin, Z. Zuo, *Green Chem.* **2025**, *27*, 1023-1030.
[28] S. Inagaki, A. Sato, H. Sato, S. Tamura, T. Kawano, *Tetrahedron Lett.* **2017**, *58*, 4872-4875.
[29] C. Zhu, S. Lee, H. Chen, H. Yue, M. Rueping, *Angew. Chem. Int. Ed.* **2022**, *61*, e202204212.
